# Cytogenetic and Transcriptomic Analysis of Human Endometrial MSC Retaining Proliferative Activity after Sublethal Heat Shock

**DOI:** 10.3390/cells7110184

**Published:** 2018-10-25

**Authors:** Mariia A. Shilina, Tatiana M. Grinchuk, Olga V. Anatskaya, Alexander E. Vinogradov, Larisa L. Alekseenko, Artem U. Elmuratov, Nikolai N. Nikolsky

**Affiliations:** 1 Institute of Cytology, Russian Academy of Sciences, Tikhoretskay Ave 4, St. 194064 Petersburg, Russia; al.l.l@mail.ru (L.L.A.); nik.n.nikolsky@gmail.com (N.N.N.); 2 Institute of Biomedical Chemistry (IBMC) of Russian Academy of Sciences, 10 Building 8, Pogodinskaya Street, 119121 Moscow, Russia; artem@genotek.ru; 3 Medical Genetics Centre Genotek, Nastavnichesky Alley 17-1-15, 10510 Moscow, Russia

**Keywords:** human endometrial MSC, sublethal heat shock, karyotype, transcriptome, DNA repair, homologous recombination, genome instability, chromosomal breakages

## Abstract

Temperature is an important exogenous factor capable of leading to irreversible processes in the vital activity of cells. However, the long-term effects of heat shock (HS) on mesenchymal stromal cells (MSC) remain unstudied. We investigated the karyotype and DNA repair drivers and pathways in the human endometrium MSC (eMSC) survived progeny at passage 6 after sublethal heat stress (sublethal heat stress survived progeny (SHS-SP)). G-banding revealed an outbreak of random karyotype instability caused by chromosome breakages and aneuploidy. Molecular karyotyping confirmed the random nature of this instability. Transcriptome analysis found homologous recombination (HR) deficiency that most likely originated from the low thermostability of the AT-rich HR driving genes. SHS-SP protection from transformation is provided presumably by low oncogene expression maintained by tight co-regulation between thermosensitive HR drivers BRCA, ATM, ATR, and RAD51 (decreasing expression after SHS), and oncogenes mTOR, MDM2, KRAS, and EGFR. The cancer-related transcriptomic features previously identified in hTERT transformed MSC in culture were not found in SHS-SP, suggesting no traits of malignancy in them. The entrance of SHS-SP into replicative senescence after 25 passages confirms their mortality and absence of transformation features. Overall, our data indicate that SHS may trigger non-tumorigenic karyotypic instability due to HR deficiency and decrease of oncogene expression in progeny of SHS-survived MSC. These data can be helpful for the development of new therapeutic approaches in personalized medicine.

## 1. Introduction

Mesenchymal stromal cells (MSCs) are attracting growing attention due to their cytoprotective, anti-inflammatory, antifibrotic, antibacterial, and pro-regenerative properties, making them a promising tool in cell-based therapy [1,2,3,4,5]. Until recently, the sources for obtaining MSCs were bone marrow, adipose tissue, placenta, umbilical cord blood, muscle cells, amniotic fluid, and dental pulp [6,7,8].

The human endometrium is a dynamically-remodeling mucosa, undergoing 400 to 500 monthly cycles of morphologic and functional changes during reproductive life [9]. Each month, 4–10 mm of mucosal tissue grows within 4–10 days in the proliferative stage of the menstrual cycle under the influence of increasing circulating estrogen levels [10]. Desquamated (peeled off) endometrium contained in menstrual blood is an easily accessible and non-invasive source of human MSC [11,12,13].

Compared to other human MSCs, eMSCs shows higher vasculogenic, proliferative, and anti-inflammatory activity [14,15]. The first clinical trials of eMSC as a cell product followed by patient observation confirm the high efficacy of these cells in the treatment of cardiovascular diseases, arteriosclerosis, myodystrophy, and many other diseases [16,17,18,19,20,21,22]. However, the transplanted cells do not withstand the difficult microenvironment caused by local hypoxia, inflammation, hyperthermia, and oxidative stress [23]. For example, less than 1% of transplanted MSCs survived to the fourth day in an immunodeficient mouse heart [24]. Recent studies indicated that MSCs respond to heat stress (HS) by accelerated stress-induced premature senescence associated with persistent signals of double-stranded DNA breaks [25,26,27,28]. However, part of the population can overcome aging and continue proliferation even after severe stress, but in such cells, destabilization of the genome may occur [25,29]. Cells that escape senescence usually come into crisis, and are characterized by genome instability and death [30,31,32].

Genome instability is generally accepted as a transformation-enabling feature [33,34]. In contrast to other cell types, human MSC revealed no transformation despite prominent genome instability during long-term culturing [29,35,36,37,38]. The aim of this study was to analyze the progeny of eMSC surviving heat shock for their genetic stability and oncogenic potential. To address this question, we investigated karyotype stability in human endometrial MSC progeny that survived sublethal heat stress (sublethal heat stress survived progeny (SHS-SP)) 6 passages ago using G-banding of metaphase chromosomes, molecular karyotyping, and transcriptome analysis with particular emphasis on DNA repair system.

The design of the experiment was developed in our previous study where we showed that a temperature of 45 °C for 30 min is sublethal for human eMSC [25]. About 60% of all eMSC underwent premature senescence after the treatment. eMSC progeny that survived sublethal HS manifested stem cell properties of the parental cells, i.e., limited replicative life span and multilineage differentiation capacity [26]. In this study we performed a first investigation of the long-term effects of sublethal heat stress on human endometrial MSC genetic stability and the DNA repair system.

Similar results were obtained with the same temperature effect on eMSC. About 60% of all eMSC underwent premature senescence after the treatment. eMSC progeny that survived sublethal HS manifested stem cell properties of the parental cells, i.e., limited replicative life span and multilineage differentiation capacity [26]. A temperature of 45 °C for 30 min has been used in a large number of studies, since this treatment is not lethal, and effectively induces a cellular response to heat stress [25,26,27]. In this study, we performed the first investigation of the long-term effects of sublethal heat stress on human endometrial MSC genetic stability and the DNA repair system.

## 2. Material and Methods

### 2.1. Cell Culture

The cells were derived from the desquamated endometrium of the menstrual blood of a healthy donor, and characterized in the Institute of Cytology RAS [37,38]. The samples were processed in accordance with the ethical committee of the Institute of Cytology of Russian Academy of Sciences and the principles of the Declaration of Helsinki. The eMSC were cultured according to a standard protocol in DMEM/F12 medium (Gibco, Carlsbad, CA, USA) supplemented with 10% bovine fetal serum (HyClone, Logan, UT, USA), 1% antibiotic-antimycotic solution, and 1% GlutaMAX (Gibco, Carlsbad, CA, USA). Cells were passed at a density of 1:3–1:4 twice in week, using 0.05% trypsin/EDTA (Invitrogen, Grand Island, NY, USA) [38,39].

### 2.2. Immunophenotyping

To confirm the mesenchymal origin of the cultured cells, we performed immunophenotypic analysis on CD markers. A single cell suspension was obtained using 0.05% trypsin and EDTA (Invitrogen, USA). Cells (1 × 10^6^/mL) resuspended in PBS solution were treated with 5% fetal bovine serum. The cells were treated with FITC or phycoerythrin-labeled antibodies to CD34, CD44, CD105, HLA-DR (Beckman Coulter, Miami, FL, USA), CD73 (BD Biosciences, San Jose, CA, USA), and CD90 (Chemicon, Darmstadt, Germany), and assayed with flow cytometry.

### 2.3. Adipogenic Differentiation

For analysis of adipogenic differentiation, the cells (2 × 10^4^ cells/cm^2^) were sown on Petri dishes (Corning, Corning, NY, USA), covered with 0.1% gelatin (Sigma, Steinheim, Germany), and grown to 90% confluence. The cells were then incubated for 5 weeks in a medium composed of 10% FCS, 10 μg/mL insulin (Sigma, Steinheim, Germany), 1 μM dexamethasone (Sigma), 250 μM 3-isobutyl-1-methylxanthine (Sigma), and 200 μM indomethacin (MP Biomedicals, Solon, OH, USA). The medium was changed every 3 days. To detect fat deposition, the cells were fixed with 10% formaldehyde for 30 min. Fat drops were stained with Oil Red dye (Sigma, USA) according to the protocol of the manufacturer. Images were taken at 200× magnification. Control cells were cultured in growth medium without the addition of stimulating factors.

### 2.4. Osteogenic Differentiation

For induction of osteogenic differentiation, the cells (2 × 10^4^ cells/cm^2^) were sown in Petri dishes (Corning, USA) and covered with 0.1% with gelatin. When the cells reached 100% confluence, they were incubated in a differentiation medium for 5 weeks, with the medium being changed every 2 to 3 days. The differentiation medium was as follows: DMEM/F12 supplemented with 10% fetal calf serum (FCS, HyClone, Logan, UT, USA), 10 mM β-glycerophosphate, 100 μg/mL l-ascorbic acid 2-phosphate, and 100 nM dexamethasone. To detect mineralization (calcium deposits), the cells were fixed with ice-cold 70% ethanol, stained with Alizarin Red S (MP Biomedicals), and counterstained with hematoxylin (Sigma). Images were taken at 200× magnification. Control cells were cultured in growth medium without the addition of stimulating factors.

### 2.5. Sublethal Heat Shock (SHS)

eMSC were exposed to HS at passage 7. The cells (at a density of 50,000 per cup) were plated in 3 cm Petri dishes (Corning, USA) and cultivated for 48 h. Plates with the cells in subconfluent density were sealed with parafilm and heated at 45 °C for 30 min in a water bath, and were then returned to 37 °C in a CO_2_ incubator. HS cells were finally analyzed by karyotyping of G-banded chromosomes, molecular karyotyping, and transcriptome analysis.

### 2.6. Karyotype Analysis

The cells were seeded with a density of 14–15 × 10^5^ cells/cm^2^. After 24 h, half of the nutrient culture medium was replaced, thereby stimulating cells to divide. Mitostatic agent–colcemid (50 mg/mL) (Sigma, USA) was added for 1 h. Then, the medium was removed, and cells treated with 0.05% trypsin and centrifuged. The pellet was suspended and treated with 0.56% KCl hypotonic solution for about 1 h (the time was chosen experimentally). The cell suspension was centrifuged (1300 rpm), resuspended, and fixed by cold mix methanol with acetic acid at a ratio of 3:1. The fixative was changed three times, the total fixation time being 1.5 h. The fixed material was dropped onto cold and wet slides. The slides were air-dried for 1 week. Chromosomes were G-banded with Giemsa stain (Fluka, Saint Louis, MO, USA) after preliminary trypsinization. Metaphase plates with well-spread chromosomes were assayed under an AxioScop microscope (Carl Zeiss, Jena, Germany), objective 20×, 100×. Chromosomes were identified in accordance with the international nomenclature of human chromosomes [39] and the Atlas of Human Chromosomes [40]. The work was carried out at the population level. In each sample, we analyzed no less than 29 metaphase plates.

### 2.7. Molecular Karyotyping

Molecular karyotyping was performed using the HumanCytoSNP-12 kit (Illumina, San Diego, CA, USA) according to the manufacturer’s protocol. Cells were plated on a 6 cm Petri dish (Corning, USA) with a density of 300,000 cells each. After 72 h, the cells were lysed to isolate the DNA. The samples were hybridized on high-density oligonucleotide microchips containing 300,000 isothermal probes covering the non-recurring genomic and intergenic regions of the human genome. The scanning of finished samples was carried out with iScan (Illumina, San Diego, CA, USA). The results were processed using GenomeStudio Genotyping Module (Illumina) and BlueFuse software (Illumina, San Diego, CA, USA).

### 2.8. mRNA Expression Analysis by Next-Generation Sequencing

Sample preparation for NGS and sequencing on the Illumina platform were performed in Genotek company (Moscow, Russia). RNA was extracted using Purelink RNA Mini Kit (AMBION, Life Technologies, Carlsbad, CA, USA). After that, mRNA was extracted from total RNA using magnetic beads (Sileks, Sileks, MO, USA). cDNA libraries were prepared using NEBNext^®^ mRNA Library Prep Reagent Set for Illumina (New England Biolabs, Ipswich, MA, USA). In this approach, mRNA was fragmented, cDNA was synthesized, end repaired, and ligated to unique sequencing adaptors to form cDNA libraries. Dual indexing was performed by PCR with NEBNext Multiplex Oligos for Illumina (dual index primers set 1). Quality control of prepared libraries was made using Bioanalyzer 2100 (Agilent Technologies, Santa Clara, CA, USA). Sequencing of cDNA libraries was done on a HiSeq2500 (Illumina) in rapid run mode with read length 100 nt.

Next-generation sequencing was done by parallel measurement of three biological samples, both for control and SHS-treated eMSC. The NGS reads were trimmed using Trimmomatic software specially developed for Illumina NGS data, with default parameters [41]. The trimmed reads were mapped to canonical nonredundant human transcriptome presented in RefSeq database [42] using Bowtie 2 software [43,44]. Only the unambiguous mappings were counted.

The differential expression was determined as in the previous work [29]. The obtained counts were analyzed using the “limma” package (implemented in R environment) [45]. Taking into account the recommendations given in the limma manual, genes with counts below 10 in all probes were discarded. With this condition, 10,888 genes were found to be expressed either in SHS-SP or control (or both). The voom-quantile method was used for data normalization. The normalized data are provided in Table S1.

### 2.9. Gene Module Analysis

The biological processes and molecular pathways enriched in differentially-expressed genes were found to be similar to those described in previous works [46,47]. The biological processes were taken from GO database [48]. For each GO category (biological process), all its subcategories were collected using GO acyclic directed graphs, and a gene was regarded as belonging to a given category if it was mapped to any of its subcategories. As a source of molecular pathways, the NCBI BioSystems was used, which is the most complete compendium of molecular pathways from different databases (NCBI Resource Coordinators, 2016 [49]). The redundancy was removed by uniting entries with identical gene sets.

The contrast test was used for analysis of gene expression folds. In this test, the mean expression fold of genes belonging to each process/pathway is compared with the mean fold of the total gene set. For evaluation of two-tailed statistical significance of an obtained contrast between these folds, the 20,000 random samplings were taken from the total gene set (of a size equal to the number of genes in a process/pathway). This method is preferable to parametric or nonparametric tests, because normal distribution that is required for parametric tests is usually absent, whereas nonparametric tests can lose a considerable amount of information. The random-sampling test is distribution independent, and retains all information. The analysis of pathway enrichment in the switched-on and switched-off genes was done similarly to previous work [50]. The switched-on genes are those whose expression was not recorded in control cells but was above threshold in SHS-SP; the switched-off genes are the other way round. The hypergeometric distribution of probability (implemented in R environment) was used to determine the statistical significance of the ratio of observed to expected gene numbers in different pathways in the switched-on and switched-off gene samples. The expected number was calculated on the grounds of the number of pathway genes in total gene dataset (assuming random gene distribution across pathways). The adjustment of the obtained *p*-values for multiple comparisons in both the contrast test and hypergeometric test was done according to the method described by Storey and Tibshirani [51]. This procedure gives the q-value, which can be considered as false discovery rate (FDR).

To reveal the possible causes of SHS-SP karyotypic instability, we assessed the expression of gene modules containing in their title terms related to DNA repair, recombination, non-homologous end joining, DNA damage sensing and response, and DNA damage checkpoint.

### 2.10. Protein-Protein Interaction Network Construction and Analysis

The protein-protein interactions (PPI) were taken from the STRING server [52]. The PPI networks were also visualized using the STRING server. We analyzed protein-protein interaction networks for proteins encoded by genes demonstrating greater-than-2-fold expression differences between SHS-survived and control eMSC. To investigate the changes in the DNA repair system, we analyzed the network for ATM, ATR, and BRCA1 interacting genes. We chose these regulators because they are known as driving genes of homologous recombination, DNA double strand break and repair, and DNA damage sensing [53]. The induced and inhibited genes were analyzed separately at interaction confidence above 0.5 (which is slightly more than default STRING confidence, above 0.4).

### 2.11. PPI Network and Cluster Analysis

Biological networks are composed by subnetworks implicated in various biological processes. To identify the subnetworks, we applied K-means clustering [52] to the PPI network for DNA-repair-related genes. After the clustering, function annotation of clusters was made. The function annotation included GO (Gene Ontology) analysis and KEGG (Kyoto Encyclopedia of Genes and Genomes) pathway analysis. The Benjamini method was used to control the false discovery rate (FDR) to correct the *p* value.

### 2.12. The Detection of SA-Β-Galactosidase Activity

Evaluation of cell aging was carried out to identify the activity of the enzyme SA-β-Galactosidase. Cells (100,000 each) were plated on 3 cm Petri dishes (Corning, USA) and cultivated for 3 days. Then the medium was removed, cells were washed with PBS, and fixed with 4% formaldehyde solution. The staining was carried out using a senescence-galactosidase staining kit (Cell Signaling, Danvers, MA, USA) according to the manufacturer’s instructions. SA-β-Gal activity was detected by cell blue staining visualized under a light microscope.

## 3. Results

### 3.1. Characteristics of eMSC

**Figure 1:**
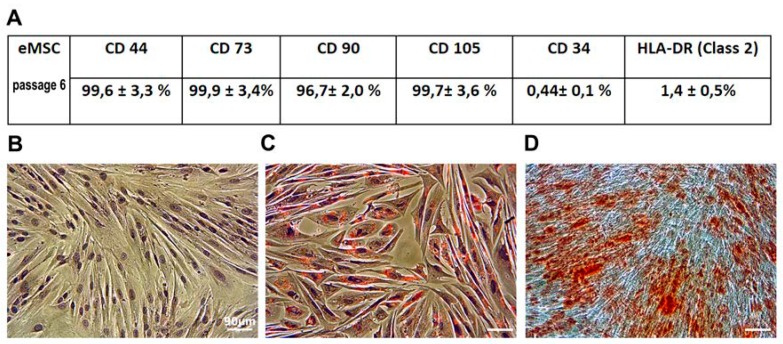
MSC CD marker expression (**A**) and capacity for differentiation into adipocytes (**C**) and osteoblasts (**D**), (**B**) Initial (control at the passage 6) cells were not subjected to differentiation stimuli. Ob: 10×, scale bar = 90 μm.

eMSC were isolated from the desquamated endometrium of the menstrual blood of a healthy donor, and had a fibroblast-like morphology. Flow cytometry analysis indicated that the eMSC were positive for CD44, CD73, CD105, and CD90, and negative for CD34 and HLA-DR surface markers, confirming that these cells show classical mesenchymal stem cell phenotype and demonstrate low immunogenicity (Figure 1A)

### 3.2. eMSC Differentiation In Vitro

To investigate eMSC capacity for mesodermal differentiation, the cells were induced to adipogenic and osteogenic differentiation. The phenotype of eMSC changed after incubation in an adipogenic-inducing medium for 21 days and an osteogenic-inducing medium for 28 days, correspondingly. The accumulation of lipid vacuoles was demonstrated by Oil Red staining. Calcium deposition was revealed with Alizarin Red (Figure 1B,D). The negative control cells were not stained by oil red and alizarin red after being cultured in the complete medium.

### 3.3. Karyotyping

#### 3.3.1. G-Banded Karyotype of Normal eMSC

**Figure 2:**
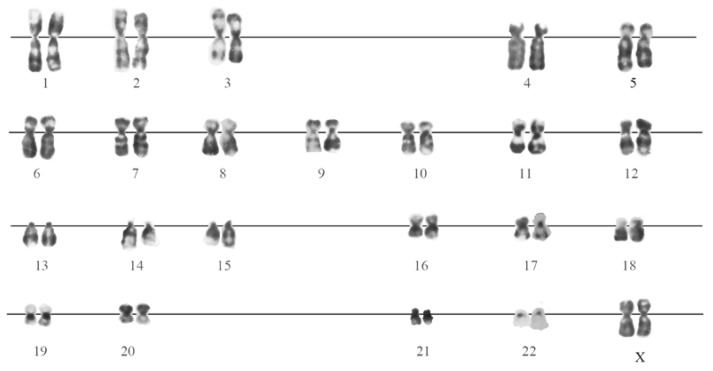
G-banded karyotype of normal eMSC, passage 13.

The karyotyping of eMSC cultured in normal conditions at the 13th passage, using differential chromosome G-banding, showed that most of the analyzed cells had a karyotype typical of normal human cells (Figure 2). Against this background, there were cells with abnormalities (below 10% in total), both in the number of chromosomes (monosomy or trisomy on some chromosomes), and in the karyotype structure (ectopic conjugation between chromosomes, isochromosomes).

#### 3.3.2. G-Banding of SHS-SP

**Figure 3:**
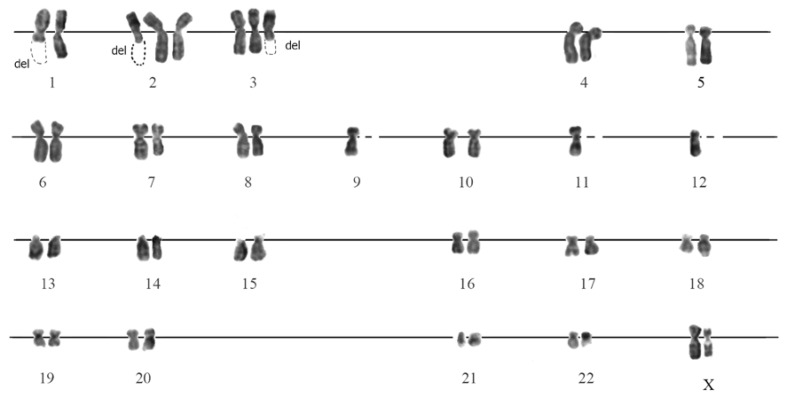
Karyotype of SHS-SP on the passage 6 after SHS (Table S2, metaphase plate N. 26). Totally SHS survived cells have gone through 13 passages. This figure illustrates near-centromere breakage of chromosomes 1, 2, 3; trisomy of chromosomes 2, 3; monosomy of chromosomes 9, 11, 12.

The karyotyping of SHS-SP after 6 passages after SHS (total 13 passages) revealed an outbreak of karyotypic instability in comparison with control cells: 80% of the analyzed cells had changes in the karyotype structure. These changes were associated with chromosomal breakages and a change in the copy of chromosomes (Figure 3). The breakages were of accidental nature and affected all the chromosomes of the set (Table S2).

#### 3.3.3. Molecular Karyotyping

**Figure 4:**
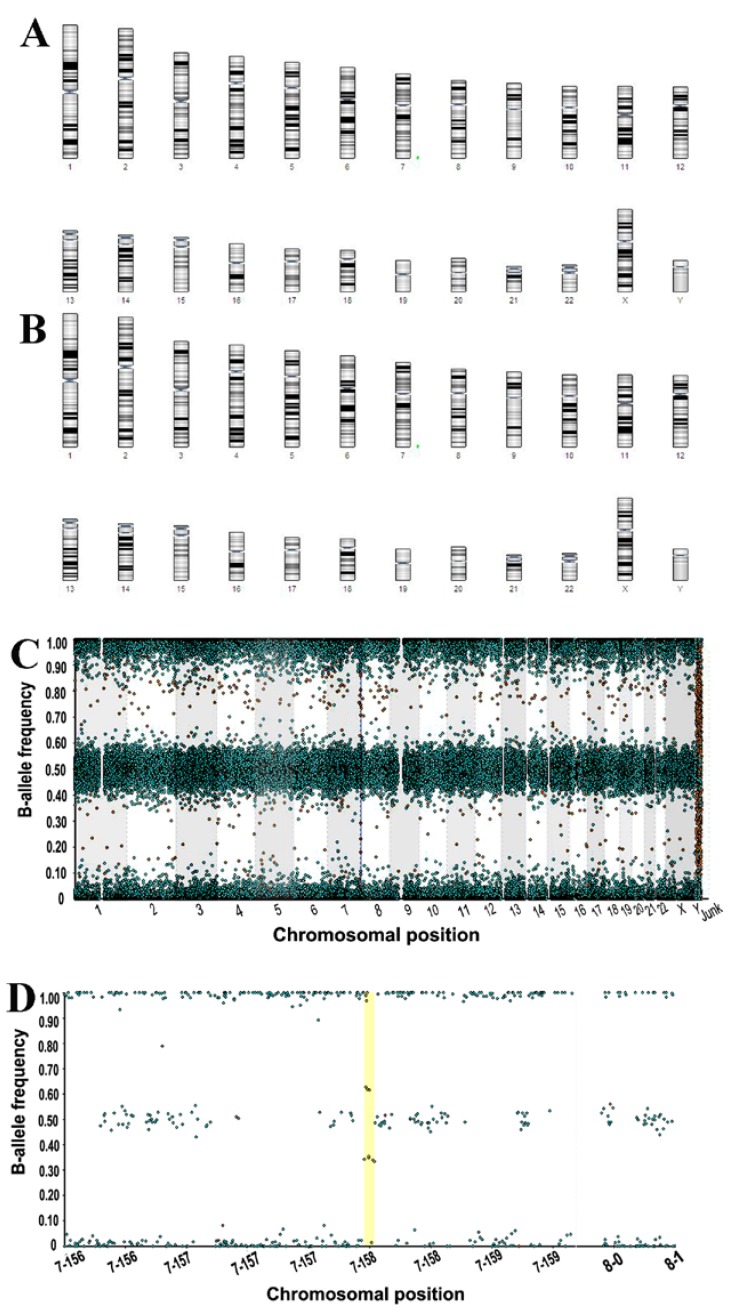
The results of molecular karyotyping of eMSC. (**A**) Karyotype of SHS-SP; (**B**) Karyotype of control cells; (**A**,**B**) scheme of genome profile with microduplication on chromosome 7; (**C**) entire genome profile with chromosome 7 microduplication; (**D**) chromosome 7 with microduplication; X on (**C**) length of all genome; X on (**D**) length of chromosome 7; Y on (**C**,**D**) B-allele frequency (BAF). BAF values range from 0 to 1: areas of homozygosity have BAF of 0 or 1; normal diploid regions have BAF of 0, 0.5, or 1; areas of allelic imbalance show intermediate values.

Molecular karyotyping of eMSC, performed at passage 13 for control cells and at passage 6 for cells after SHS (total 13 passages), revealed that 22 pairs of chromosomes did not differ in their genetic structure from those of the chromosomes of the normal human karyotypic set. The only exception was chromosome 7; in all analyzed cellular variants, microduplication was recorded in the locus 7q36.3 at 62,680 bp (62 kb) (Figure 4A,B).

### 3.4. Transcriptome Analysis

#### 3.4.1. Functional Enrichment Analysis of DNA-Repair-Related Gene Modules and Evaluation of Expression of Master DNA Repair Regulators

**Figure 5:**
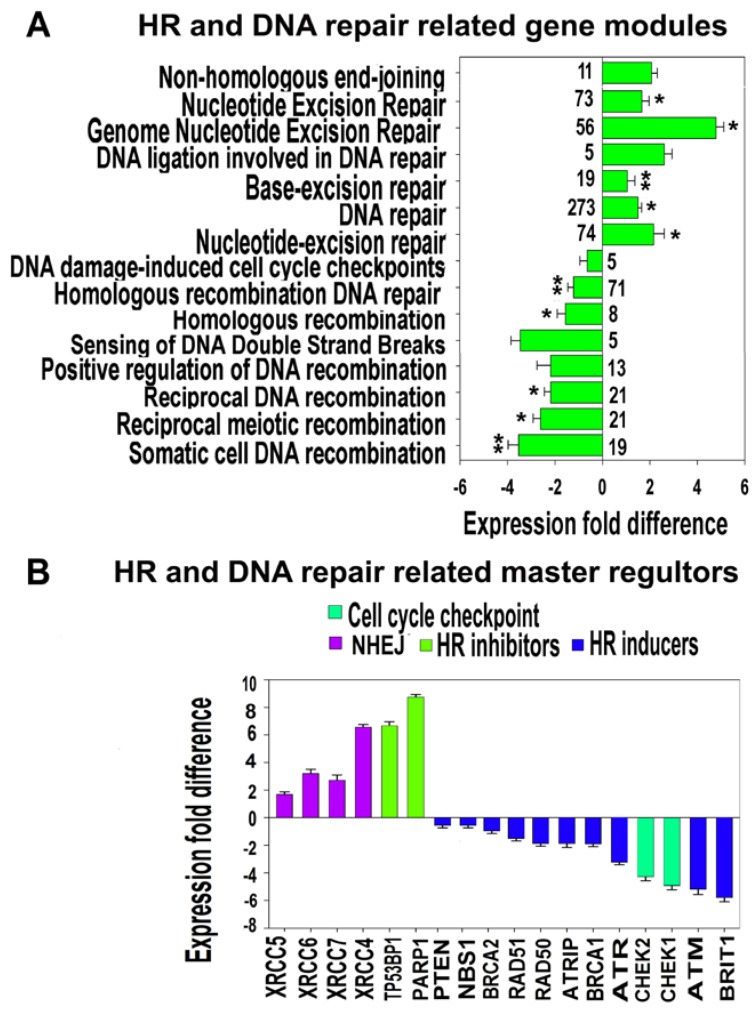
Expression difference of HR and NHEJ related gene modules and master regulators between control eMSC and sublethal heat shock survived progeny (SHS-SP). (**A**) GO biological processes and BioSystems pathways related to DNA repair that are significantly enriched in differentially expressed genes. ** *q* < 0.001; * *q* < 0.01; no asterisk–*q* < 0.01. The numbers near the bars indicate gene number in a module. The data were obtained with 10,888 genes. This figure illustrates the induction of pathways involved in excision repair and the impairment of pathways involved in recombination and DNA damage sensing and checkpoints. (**B**) Expression difference of HR and NHEJ in SHS-SP vs. control eMSC.

To understand the nature of SHS-SP genetic instability, we evaluated the expression of gene modules related to DNA repair, which contain in their titles the terms “repair”, “DNA repair”, “recombination”, “DNA double strand breaks”, “DNA damage” and “non-homologous end joining” (Figure 5). The results of functional enrichment analysis of differentially-expressed genes indicated that the modules implicated in excision repair, DNA damage checkpoint control, and non-homologous end joining (NHEJ) were induced, whereas the modules involved in homologous recombination (HR) and DNA damage sensing and DNA damage checkpoint were inhibited (Figure 5A, Table S3).

To further verify these results, we selected DNA repair genes that were switched on or off in SHS-SP vs. unheated cells. This selection enabled us to assess only the qualitative changes, and therefore, to make the analysis more rigorous. Overall, this selection yielded 132 activated and 84 suppressed genes, which is more than 65% of all (374) deregulated genes related to DNA repair. Gene module functional enrichment analysis of switched-on and -off genes confirmed the data obtained with the entire DNA repair gene list (Tables S4 and S5). The switched-on genes were related mainly to base and nucleotide excision repair, mismatched repair, and translesion DNA synthesis. The switched-off genes were associated with gene modules implicated in “Homologous recombination”, “ATM signaling”, “BRCA1, BRCA2” and “ATR in Cancer Susceptibility”, and “Double-strand break repair via nonhomologous end joining”, thus providing additional verification of our conclusions made on the basis of all DNA-repair-related gene analysis.

After identification of deregulated gene modules, we investigated the expression of their driving genes that were identified using the database from Peng, et al. [53]. Figure 5B indicates that the strongest effects were the downregulation of positive regulators of HR and DNA damage sensing and the induction of the negative regulators of HR and positive regulators of NHEJ (Figure 5B). Accordingly, the cell cycle checkpoint regulators (*CHEK1* and *CHEK2*) initiating cell cycle arrest to prevent damaged cells from progressing through the cell cycle were inhibited (Figure 5B).

#### 3.4.2. PPI Network Analysis for ATM, ATR and BRCA1 Interacting Genes

To obtain a comprehensive view of the effects of DNA repair system modification in SHS-SP, we investigated protein-protein interaction network for genes interacting with the master regulators of DNA damage sensing (*ATM, ATR)* and HR (*BRCA1*).

**Figure 6:**
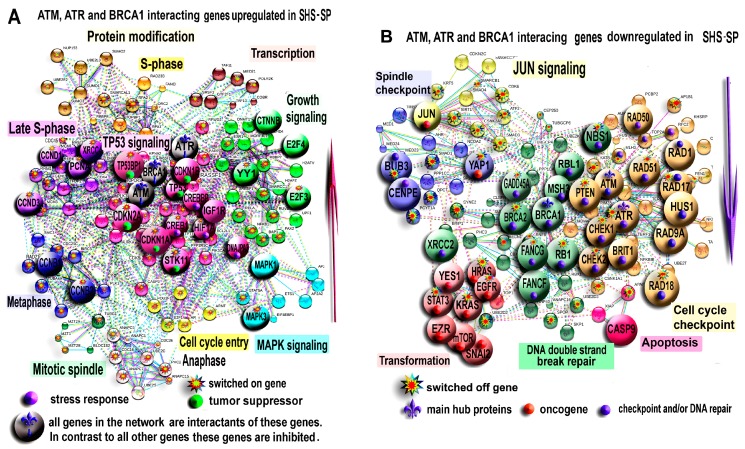
Protein-protein interaction network for ATM, ATR and BRCA1 interacting genes demonstrating expression difference above twofold between control eMSC and SHS-SP. The network for induced genes (**A**) and for inhibited genes (**B**). Large symbols show genes with above fivefold expression difference. Symbols marked with asterisks show switched-on and switched-off genes. The networks were constructed using STRING server (evidence view option) with interaction confidence above 0.5. Clustering was performed by MCL algorithm. The clusters are titled according to GO biological processes and KEGG molecular pathways enriched in cluster-comprising genes with *q* < 10^−4^. The network at (**A**) illustrates that SHS-SP show long-term induction of TP53 signaling and signaling related to first stages of cell cycle. The network at (**B**) indicates that SHS-SP downregulate signaling related to DNA repair, DNA damage checkpoint, spindle checkpoints and transformation.

Figure 6A illustrates the network for the induced genes. In the middle of the network, there is a large cluster of *TP53* regulated genes. The main *TP53* interacting genes are *CDKN2A (p19, ARF), CDKN1A* (*p21*), *CDKN1B* (*p27*), and *STK11*. This indicates that *TP53* expression increases as a result of stable co-regulation of tumor suppressors. The *TP53-*regulated cluster also contains a number of genes that are responsive to stress: *HIF1A*, *DNAJPA3 (HSP40)*, *IGF1R, CREB,* and *CREBBP*. The other clusters are related to the regulation of growth signaling and different cell-cycle phases, which confirms a substantial SHS-SP proliferation ability.

Figure 6B shows the network for inhibited genes. This network contains gene clusters related to DNA breakage sensing and DNA damage checkpoint (*ATM, ATR, HUS1, CHEK1, PTEN, BRIT1, RAD1,* and *9A,17,18*) and the clusters of genes regulating apoptosis (*CASP9, XIAP,* and *APAF*), homologous recombination and DNA double strand break repair (*BRCA1*, *BRCA2, FANCG, FANCF, RBL1, MSH3, RAD51, NBS1,* and *GADD45A*), and spindle assembly checkpoint (*BUB3* and *CENPE)*. There is also a tight cluster of transformation related genes (*HRAS, KRAS, STAT3, EZR, YAP1, EGFR, mTOR,* and *SNAI2*) and two clusters regulated by *JUN* and *YAP1* oncogenes. Thus, PPI network analysis indicated that despite clear hallmarks of compromised DNA damage sensing, HR deficiency DNA repair and checkpoint, SHS-SP maintain a higher expression of tumor suppressors and a lower expression of oncogenes compared to unheated cells.

#### 3.4.3. Characterizing of HR and DNA Repair Regulator Thermostability

**Figure 7:**
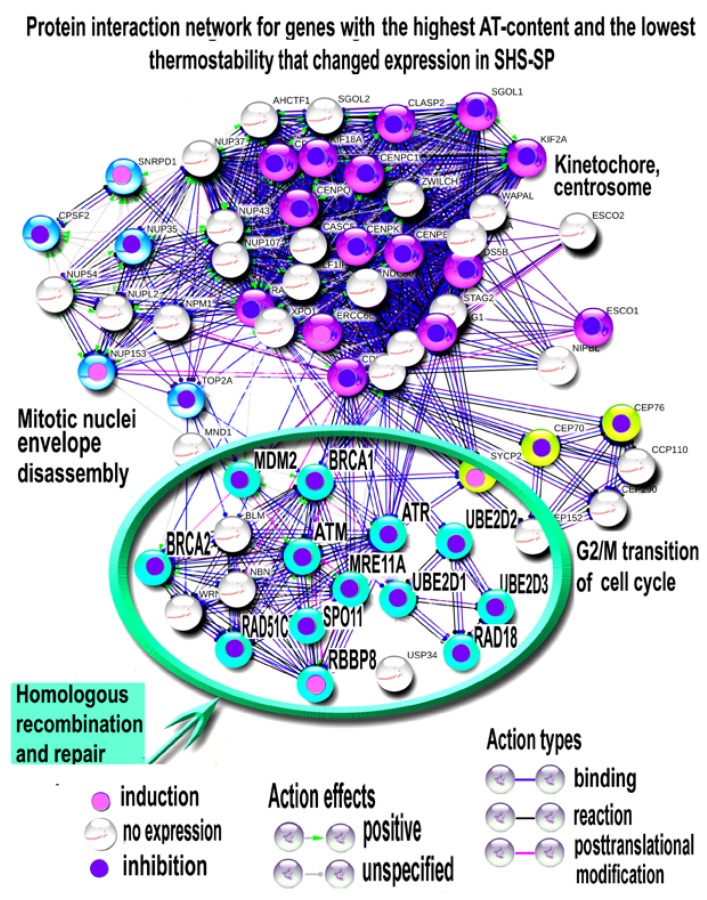
Protein interaction network for genes with highest AT-content and lowest thermostability that changed expression in SHS-SP. The network constructed with STRING server (molecular action option) at highest interaction (confidence > 0.9). Gene clusters were obtained with MCL clustering. The clusters are titled according to GO biological processes and KEGG molecular pathways enriched in cluster-comprising genes with *q* < 10^−4^. The figure illustrates the down-regulation of thermosensitive genes in SHS-SP vs. control and shows the particular decrease in expression of homologous recombination related genes.

We next investigated why HR and DNA damage checkpoint related genes show decreased expression after 6 passages after SHS, in contrast to other DNA repair pathways (Figure 5 and Figure 6). To address this question, we referred to the recent study on gene functional distribution according to their AT content that is inversely related to gene thermostability [50,54]. Specifically, we compared our results with the data describing core clusters of genes whose interactants are modularly enriched in genes with the lowest thermostability (AT-rich genes). Figure 7 shows genes from these clusters with marked DNA repair and HR related driver genes that changed expression in SHS-SP compared to unheated cells. It is clearly seen that of 12 genes with changed expression, 11 were downregulated and only 1 was upregulated (*p* < 0.0001, binomial test and *p* < 0.001 Mann-Whitney for the difference in expression change sign). It is important to underline that among these DNA-repair-related genes, there is one severe oncogene–MDM2 (TP53 antagonist) that decreased expression in SHS-SP compared to control (Figure 7). Thus, our results show that long-term downregulation of HR and DNA damage checkpoint drivers occurs because of changes in thermosensitive genes.

#### 3.4.4. PPI Network Analysis of Oncogenes and Thermosensitive DNA-Repair-Related Genes

**Figure 8:**
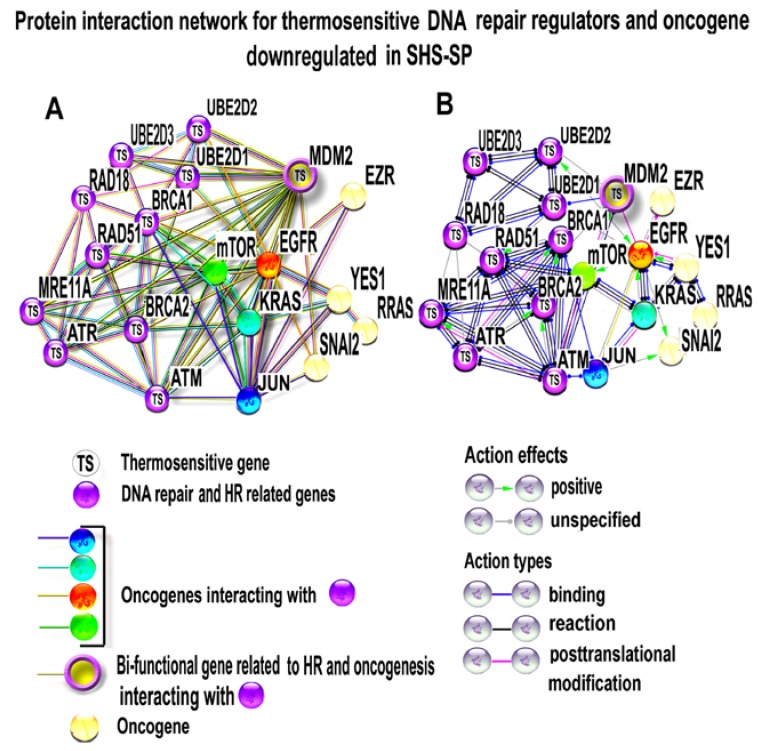
Protein interaction network for thermosensitive HR regulators and oncogenes that decreased expression in sublethal heat shock survived progeny (SHS-SP). The network constructed with ATM, ATR and BRCA1 interacting genes (presented also at Figure 2B). (**A**) evidence view illustrating multiple bonds between thermosensitive HR regulators and oncogenes. (**B**) molecular action view indicating that HR regulators and oncogenes are interconnected only with positive or unspecified interactions. The networks in (**A**,**B**) were constructed using STRING server at interaction confidence above 0.9. This figure illustrates a tight interaction between thermosensitive HR regulators and oncogenes confirming that SHS related HR inhibition also switches off severe oncogenes.

To answer the question of why heat-stress-related karyotype instability is accompanied by the decrease of oncogene expression, we investigated protein interactions between the down-regulated oncogenes shown in Figure 6B, including *EGFR*, *mTOR*, *JUN*, *HRAS*, *RRAS*, *YAP1*, *SNAI2*, and *EZR*, thermosensitive *MDM2* oncogene, and thermosensitive HR and DNA-repair-related genes that are also presented in Figure 6B and Figure 7. Figure 8A indicates that many thermosensitive HR and DNA-repair-related genes are connected with oncogenes. For example, oncogene *mTOR* connects HR related genes *MRE11A*, *BRCA1*, *ATM* and *ATR*, and *RAD51* with oncogenes *EGFR*, *KRAS*, *HRAS*, *JUN*, *MDM2*, and *YAP1*. The thermosensitive oncogene *MDM2* bridges DNA repair regulators *MRE11A*, *BRCA1*, *BRCA2*, *ATM*, *ATR*, *UBE2D1*, *UBE2D2* and *UBE2D3* with oncogenes *mTOR*, *JUN*, *KRAS* and *SNAI*. EGFR links DNA-repair-related genes *BRCA1, BRCA2, ATM, UBE2D1,* and *UBE2D2* with oncogenes *mTOR*, *MDM2*, *EZR*, *YES1, RRAS*, *SNAI2*, *KRAS* and *JUN*. KRAS joins DNA repair genes *BRCA1*, *BRCA2*, and *ATM* and oncogenes *mTOR*, *EGFR*, *MDM2*, *JUN* and *YAP1* (Figure 8A). Figure 8B illustrates type of molecular interactions between DNA repair drivers and oncogenes. It is clearly seen that all these interactions are of neutral or inducing nature (not inhibiting), indicating that DNA-repair-related genes and oncogenes can regulate each other. Thus, downregulation of HR thermosensitive genes can also decrease the expression of oncogenes. The master regulators of this interconnection are mTOR, MDM2 and EGFR, and KRAS.

#### 3.4.5. Comparative Analysis of Cancer-Related Gene Set Expression in SHS-SP VS. Unheated Cells and in hTERT Transformed Human Bone Marrow MSC VS. Control

**Figure 9:**
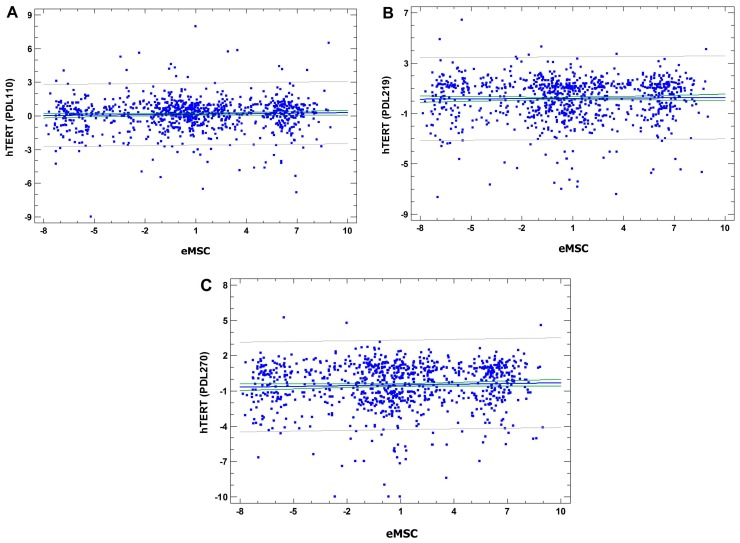
The absence of correlation between gene expression changes at various stages of hTERT-induced transformation in human bone marrow MSC (the data from Takeuchi, et al., 2015) and gene expression changes in SHS-SP. (**A**) the first stage (hTERT PDL110, *r* = 0.04, *p* = 0.2); (**B**) the second stage (hTERT PDL219, *r* = 0.02, *p* = 0.6); (**C**) the third stage (hTERT PDL270, *r* = 0.05, *p* = 0.2).

Next, we compared our results with the data on gene expression changes in human bone marrow mesenchymal stromal cells (hbm MSC) during different stages of hTERT-induced transformation [55]. The stages of transformation are indicated by population doubling level (PDL). The authors [55] designated the following stages: stage I (PDL 60–90), stage II (PDL 91–150), stage III (PDL 151–230), and stage IV (PDL 231–295). The comparison was based on 1731 genes that the authors designated as transformation-related in human mesenchymal stromal cells. Linear regression analysis revealed no statistically-significant correlation between gene expression changes in TERT transformed vs. control cells and in SHSSP vs. unheated cells at all three stages of transformation, thus confirming that SHS-SP are not transformed and show non-tumorigenic karyotypic instability (Figure 9A–C).

### 3.5. SHS-SP Replicative Aging Evaluation Using SA-β-gal Activity Detection

**Figure 10:**
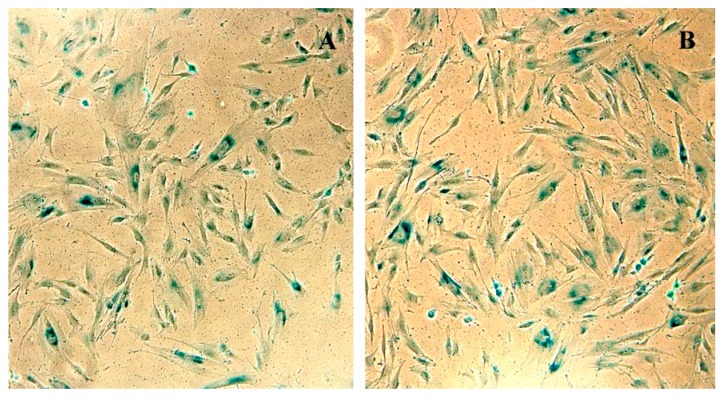
eMSC that survived sublethal heat shock (SHS-SP) exhibit SA-β-galactosidase expression at passage 30. (**A**) X-gal staining of parental cells. (B) X-gal staining of cells that survived heat shock. Figure illustrates that both control and experimental cells enter replicative aging at late passages, and therefore, do not undergo immortalization.

The SHS-SP entered replicative senescence after long-term cultivation (Figure 10); this can be seen from positive SA-β-galactosidase staining of both normal eMSC and SHS-SP at the 30-passage of cultivation. This result confirms that SHS-SP cells are not immortalized or transformed.

## 4. Discussion

The problem of possible spontaneous MSC transformation in culture is of great importance, since it concerns the genetic safety of the transplanted material. At the same time, this issue is still a matter of debate, especially in long-term follow-ups [56]. It is worthy of note that several articles describing the spontaneous transformation of human MSC in vitro were eventually withdrawn by the authors because of a revealed cross-contamination of cultures with transformed cells [57,58]. Thus, the problem is still open. In this regard, the evaluation of long-term physiological and genetic stability of human eMSC after severe (sublethal) heat stress is essential for the evaluation of possible safety margins.

In the framework of this problem, we investigated the progeny of human endometrial MSC that survived sublethal heat stress 6 passages ago. Before the experiment, we verified minimal criteria for multipotent mesenchymal stromal cells stated by the International Society for Cellular Therapy [59] in human eMSC cells (at passage 6) by CD marker expression and by the evaluation of osteogenic and adipogenic differentiation potential.

In this study, G-banding of SHS-SP revealed a burst of karyotypic instability caused by the breakage of chromosomal material and aneuploidy. The chromosomal instability was usually considered as a transformation-enabling feature [60,61]. However, now there are several points of view that stir debate on this topic; some authors believe that karyotypic disorders can provide a selective advantage and become a source of cellular transformation under certain circumstances [62,63,64,65,66,67,68], while others think that genetic and epigenetic changes occurring as a result of endogenous or exogenous stress can be considered as elements of cell adaptation to environmental changes [69,70,71]. Thus, random karyotypic alterations without selective advantage should not necessarily cause or promote transformation [35,67,72,73,74,75].

To address this issue, we performed molecular karyotyping and transcriptome sequencing. Molecular karyotyping revealed microduplication in chromosome 7 both in control and SHS-SP, which indicates that this is a donor-specific characteristic, and is not acquired as a result of SHS.

Thus, our data did not reveal differences in clonogenic microduplications between SHS-HP and control eMSC cells at molecular level, suggesting an absence of transformation related features in SHS-SP. Currently, we cannot compare the data on endometrial MSC with the data on other MSC types because of the absence of the literature concerning the problem.

Concerning the transcriptome analysis, it should firstly be noted that we made bulk transcriptome sequencing, which averages all variants of gene expression difference between control and SHS-SP in individual cells (in spite of heterogeneity in heated cells). However, cell heterogeneity in SHS-SP revealed by molecular and classical karyotyping shows no signs of clonal changes, i.e., it is random. This conclusion is based on the following observations. First, the results of molecular karyotyping of control cells and SHS-SP were identical. Second, in classical karyotyping, we never observed two identical chromosomal breaks; even in the same chromosome, they were in different locations. Third, if we consider the complex pattern of abnormalities in the whole chromosome set, it was also always unique. In contrast, the expression profiles show similar patterns between experiments (The consensus list of enriched pathways for triplicates is shown in Table S3). Therefore, the averaging of random cell heterogeneity implicated in bulk sequencing is quite reasonable. Moreover, we made an additional bioinformatic analysis, which can take into account possible extreme variants in individual cells (analysis of switched-on and -off genes). This test confirmed the conclusions made from the main part of analysis.

The analysis of transcriptome sequencing data indicated that SSH-SPs possess a modified DNA repair; this can be seen from the HR deficiency and inhibition of DNA damage checkpoint and sensing. It was recently shown that the immediate effects of heat stress on DNA repair include deprivation of virtually all its branches with a most severe disruption of HR, leading to HR deficiency (reviewed by Kantidze, et al., 2016 [28]). As a result, hyperthermia provokes the formation of multiple DSBs [76]. The long-term effects of hyperthermia on DNA repair in cultured mesenchymal stromal cells currently remains unstudied. Our data provided the first evidence that in SHS-SP, almost all systems of DNA repair recover after sublethal heat stress with the exception of HR and DNA damage checkpoint and sensing.

One possible explanation for this disruption of HR and DNA damage checkpoint and sensing can be a thermostability-dependent response to overheating that is governed by gene GC content. The GC content is related to a number of DNA physical properties [50,54]; the most relevant here is the lower thermostability of the AT-pair because of two hydrogen bonds between the bases versus three bonds in GC pair. Therefore, genes with high AT content are more heat stress sensitive than GC rich genes. Although the AT-rich genes generally show lower mutation probability than GC-rich genes [50], heat stress may be their Achilles’ heel. Indeed, the main regulators of HR and DNA damage checkpoint and sensing, including ATM, ATR, BRCA1, BRCA2, MRE11, RAD18 and RAD51, as well as DNA-repair-related oncogene MDM2, are among genes with the lowest thermostability in the entire genome due to high AT-content.

Moreover, the protein interaction enrichment analysis (PIEA) indicated that the interactants of these regulators are also enriched in genes with high AT-content [50], suggesting their aberrant functioning after heat stress. In our experiments, SHS-SP showed downregulation of all main AT-rich DSBs repair and sensing genes. Thus, our data provide evidence that heat stress may be considered as a genotoxic agent operating in human MSC through HR impairment. This result is in good agreement with numerous epidemiological and experimental studies evidencing that external hyperthermia and fever in pregnancy may cause neuronal, cardiac, and limb defects in humans and rodents [77,78]. These facts suggest that we are still far from having a complete understanding of the long-term genetic effects of hyperthermia on gene function.

Stem cell safety is one of the most important questions in biology and medicine. It was recently reported that SHS-SP do not show the hallmarks of cancer identified by Hanahan and Weinberg [29]. In this study, we checked for transformation-related features identified in hTERT transformed human bone marrow MSC with genetic instability in culture [55]. We used this study because gene function and cell phenotype depend on cell differentiation and transformation–related features like DNA instability, aneuploidy, and polyploidy [79,80,81,82,83,84]. For example, c-Myc functions as oncogene in diploid adult cells and as stemness inducer in embryonic stem cells and in polyploid cells Vazquez-Martin, et al. [85]. Accordingly, a detailed review of TP53 function in embryonic and adult stem cells provides evidence that in tissue-specific stem cells (i.e., in mesenchymal stem cells), TP53 triggers differentiation rather than apoptosis in response to stress [86]. Again, a series of recent articles indicated that TP53 induction in response to oxidative stress and overheating does not promote apoptosis, but rather, induces senescence [25,26,87]. Therefore, it was important to search for transformation traits that are MSC–specific.

From our results, SHS-SP do not show features common with initial or advanced stages of transformation identified in MSC [55], even despite genetic instability and HR deficiency. This result is in good agreement with the data obtained with genetically-unstable MSC of various origins after long-term cultivation and under stressful conditions [88,89]. Also, no neoplastic changes were registered in mesenchymal stromal cells of osteosarcoma patients after long-term culturing [90]. Thus, the absence of steps towards transformation is a common phenomenon for genetically-unstable MSC in general, and SHS-SP in particular.

Which pathways can provide transformation protection, and why do SHS-SPs maintain safe oncogene/tumor suppressor balance despite HR and genetic instability? Currently there is no answer to these questions. To shed light on the complicated interaction between genetic instability, HR deficiency, and impaired oncogene signaling, we investigated protein-protein interactions between thermosensitive HR regulators and oncogenes that decreased expression in SHS-SP. Surprisingly, we found that thermosensitive HR regulators are interconnected with several severe oncogenes, including mTOR, MDM2, EGFR and KRAS, and that one of these oncogenes (MDM2) is also AT-rich, and therefore, thermosensitive. It is known that MTOR, EGFR and MDM2 inhibitors also suppress HR and impair oncogene expression [91,92,93,94,95,96,97]. Thus, our data indicate that heat stress may trigger long-term HR deficiency accompanied by the suppression of mTOR, MDM2, EGFR, KRAS and other oncogenes, which contributes to heat-stress-associated karyotypic instability with non-tumorigenic properties.

## 5. Conclusions

In summary, our data indicate that the SHS-SPs are characterized by an outbreak of karyotypic instability. This instability has a random character that was confirmed by G-banding and molecular karyotyping. The instability is caused mainly by HR deficiency state. Apparently, the inhibition originates from the low thermostability of HR driver genes, which suggests that SHS may induce disruption of their expression. SHS-SP protection from transformation despite genetic instability is provided by safe oncogene/tumor suppression equilibrium. This equilibrium is probably maintained by a tight interconnection between thermosensitive HR and DNA-repair regulators and oncogenes mTOR, EGFR, MDM2 and KRAS, suggesting that HR deficiency inhibits the expression of these oncogenes. The transformation-related features previously identified in hTERT transformed MSC in culture were not found in SHS-SP, suggesting no traits of transformation in them. In the process of cultivation, these cells entered into replicative aging, thus confirming their mortality. Overall, our data indicate that despite the detected outbreak of karyotypic instability, SHS-SP are not subjected to oncogenic transformation and immortalization. These data can be helpful for the development of new therapeutic approaches in personalized medicine.

## Supplementary Materials

Supplementary Tables S1–S5 are available online at http://www.mdpi.com/2073-4409/7/11/184/s1.
