# Supplementary material for: Cytogenetic and Transcriptomic Analysis of Human Endometrial MSC Retaining Proliferative Activity after Sublethal Heat Shock
Source: Cells. 2018 Oct 25;7(11):184. doi: 10.3390/cells7110184 (PMC6262560; doi:10.3390/cells7110184)
Supplement: Supplementary file 1 [file cells-07-00184-s001.zip › cells-363926-supp/Supplementary Table 1.pdf]

Supplementary Table 1.

The limma voom-quantile normalized expression levels of control and SHS-SP eMSC (log2).

NCBI_id	control_1	control_2	control_3	SHS-SP_1	SHS-SP_2	SHS-SP_3
1	5.498944719	5.873346	5.124543452	5.55876787	5.588224375	5.529311366
2	-0.352108471	-0.352108	-0.352108471	7.085333103	4.927679215	2.770025327
14	5.749402114	5.5109155	5.987888748	7.119845432	6.606183222	6.863014327
16	8.507545809	4.9640136	6.735779686	6.932030641	5.539811681	6.235921161
19	5.759644951	5.9391922	6.118739492	-0.352108471	-0.352108471	-0.352108471
20	4.813611796	6.3968684	7.980124969	4.141880939	3.771324033	3.400767127
21	5.284974356	6.3585275	4.211421254	-0.352108471	-0.352108471	-0.352108471
23	4.947007189	4.9799919	4.914022459	6.822208827	6.841511354	6.802906299
27	8.375043057	10.663551	6.086534978	7.114489208	6.128734228	8.100244189
29	-0.352108471	-0.352108	-0.352108471	6.470706794	6.545950789	6.621194783
32	6.988736991	6.5851844	6.181631761	-0.352108471	-0.352108471	-0.352108471
36	9.985415816	8.1928158	9.089115811	8.201776207	7.642442186	7.922109197
37	-0.352108471	-0.352108	-0.352108471	8.926349609	7.389533528	8.157941569
47	1.392109249	3.136327	-0.352108471	3.151146871	3.177797522	3.12449622
48	6.830448955	6.502263	6.174077101	2.089170226	8.790664649	5.439917437
50	-0.352108471	-0.352108	-0.352108471	5.903760504	5.926961306	5.880559702
51	9.456984442	6.8163048	4.175625127	3.752620143	3.661624855	3.570629566
53	6.433318728	6.4453648	6.421272681	4.178899946	4.245214285	4.112585606
59	8.355311288	7.6994178	8.027364562	9.93141753	9.874056381	9.988778679
60	12.84789093	12.25982	13.43596189	14.64105918	14.46262557	14.28419196
71	11.09860229	10.766393	11.43081133	12.09160162	10.00423616	14.17896707
72	9.43473142	9.9468876	9.690809527	11.86516115	7.490422799	9.677791973
81	8.092283664	7.3887488	8.795818545	7.992216534	9.865303151	11.73838977
87	9.684451926	8.4537991	7.223146352	8.255643145	13.61139711	10.93352013
91	5.017136436	4.5152298	5.519043066	-0.352108471	-0.352108471	-0.352108471
93	6.651724224	4.6929891	2.734254014	4.704154739	5.416430737	5.060292738
100	-0.352108471	-0.352108	-0.352108471	6.056053999	5.531438525	6.580669473
101	5.754553399	5.3152588	6.193847962	-0.352108471	-0.352108471	-0.352108471
102	-0.352108471	-0.352108	-0.352108471	6.590036833	6.810957895	7.031878957
103	5.295280088	5.2849744	5.274668624	6.804723141	7.655057176	5.954389107
105	4.13408305	4.9140225	3.354143641	0.289051906	-0.352108471	0.930212283
114	-0.352108471	-0.352108	-0.352108471	2.351086241	3.255950465	1.446222016
115	-0.352108471	-0.352108	-0.352108471	5.980334028	5.351960992	6.608707064
118	6.801813774	6.8152071	6.788420481	7.387215402	7.407655215	7.366775589
123	5.853283936	5.2642518	5.55876787	3.32030891	7.178522138	11.03673537
128	-0.352108471	-0.352108	-0.352108471	9.979463217	4.759148467	7.369305842
132	6.332466669	3.7097133	8.955220043	5.052267685	7.335513206	2.769022165
133	10.26905022	8.1755796	9.222314905	10.692977	7.793854929	9.243415966
136	-0.352108471	-0.352108	-0.352108471	8.078823743	4.659489439	6.369156591
141	6.433318728	6.6296792	6.23695821	-0.352108471	-0.352108471	-0.352108471
142	-0.352108471	-0.352108	-0.352108471	8.375904556	8.392497989	8.359311122
143	6.212536209	6.802189	5.622883451	-0.352108471	-0.352108471	-0.352108471

146	7.334122775	7.3654996	7.302745947	-0.352108471	-0.352108471	-0.352108471
147	6.463674915	5.9872832	6.94006665	-0.352108471	-0.352108471	-0.352108471
148	5.95559053	6.6400194	7.324448256	-0.352108471	-0.352108471	-0.352108471
153	9.416826983	2.1319137	5.774370319	-0.352108471	-0.352108471	-0.352108471
156	5.463922025	5.5293114	5.398532684	5.494417177	5.494417177	5.494417177
157	6.061691422	7.3209677	6.691329572	4.23879782	6.190805163	8.142812505
158	-0.352108471	-0.352108	-0.352108471	4.388315565	3.245625856	2.102936146
160	5.420657276	3.9184181	2.416178924	7.995294036	8.245819749	7.744768323
161	3.987750338	3.1277379	2.267725412	7.340618328	6.915799893	6.490981459
162	7.54201368	7.2665096	7.817517777	9.518347022	6.74407332	3.969799619
163	5.940575529	7.0224724	6.481523945	8.622155348	6.343488979	7.482822164
164	6.767951819	8.5084058	5.027497824	6.588485208	6.889813725	6.739149467
165	7.360780676	8.273972	6.447589338	3.95724469	6.32531018	8.69337567
166	8.009623545	8.9052606	7.113986506	9.010141782	7.429082739	10.59120083
176	2.769242222	2.262153	2.515697587	-0.352108471	-0.352108471	-0.352108471
186	-0.352108471	-0.352108	-0.352108471	4.934492169	5.7359272	5.335209685
191	7.251757684	7.1096209	7.393894455	8.216996418	7.38669897	6.556401523
196	8.099059847	5.7302292	6.914644543	8.281500709	6.68898917	5.096477631
202	-0.352108471	-0.352108	-0.352108471	1.383520155	3.119148782	-0.352108471
203	5.993092449	6.7441214	6.368606935	5.93669218	6.694006421	7.451320663
204	5.958359385	6.6404746	5.276244216	8.496512176	7.054600764	5.612689352
205	-0.352108471	-0.352108	-0.352108471	0.874413692	-0.352108471	2.100935855
208	-0.352108471	-0.352108	-0.352108471	6.560204177	8.321844961	4.798563393
210	5.75668	6.3180937	6.879507361	5.233567424	3.886315413	6.580819434
211	1.996581495	3.3748827	2.685732087	4.570755367	2.124237496	-0.322280375
214	-0.352108471	-0.352108	-0.352108471	2.571154353	5.494417177	-0.352108471
215	-0.352108471	-0.352108	-0.352108471	5.55876787	7.797988557	3.319547183
216	4.730615771	4.617923	4.843308497	7.713028572	7.733387404	7.692669739
218	6.736748339	6.0307241	5.324699835	-0.352108471	-0.352108471	-0.352108471
219	7.195849524	5.1263858	9.265313239	6.344555562	5.656835755	7.032275369
220	6.536037335	6.7440015	6.640019393	-0.352108471	-0.352108471	-0.352108471
221	6.977475365	5.4283627	8.526588063	7.431698237	5.285920678	6.358809458
223	5.5220932	6.2248629	6.927632549	4.07228332	8.828322355	6.450302837
226	10.10924753	9.4304235	10.78807157	10.0301857	12.64585698	7.414514428
230	-0.352108471	-0.352108	-0.352108471	5.022802207	6.354443088	5.688622647
231	8.981447581	5.5568597	7.269153621	10.41406676	7.083829938	8.748948349
239	-0.352108471	-0.352108	-0.352108471	0.897715333	-0.352108471	2.147539136
258	-0.352108471	-0.352108	-0.352108471	2.252618212	2.655049751	1.850186673
262	9.209545952	6.2510559	7.730300925	-0.352108471	-0.352108471	-0.352108471
267	-0.26064198	3.1277379	6.516117731	9.112842831	7.295045874	5.477248917
269	-0.352108471	-0.352108	-0.352108471	1.964002179	0.808855576	3.119148782
271	4.021726945	4.0103646	4.016045771	6.296043865	4.217085872	8.375001859
272	7.094235505	6.0537025	6.573968999	0.82273449	-0.352108471	1.997577452
274	5.853275199	6.991795	8.130314896	5.93842295	5.050411404	5.494417177
285	3.931233873	4.8215393	5.711844708	5.545830322	5.432742342	5.319654363
290	7.450449055	8.2816111	6.619286969	8.917868772	7.76913794	6.620407108
291	3.945140305	5.2712224	4.60818135	8.457196657	5.636441802	7.04681923

292	7.259391142	7.1562852	7.207838162	6.322936265	8.531660807	7.427298536
293	8.12782046	6.4143432	9.841297688	9.482999764	9.322656311	9.402828037
301	8.137867046	8.1635425	8.112191629	8.200565503	8.204591974	8.196539031
302	10.98660727	10.410703	10.69865532	11.37003559	11.34383116	11.39624002
304	5.688622647	4.5914138	6.785831478	5.070633386	1.529833188	3.300233287
307	-0.352108471	-0.352108	-0.352108471	7.49141599	6.671874638	5.852333286
308	11.42953087	8.4032725	9.916401704	8.899837123	9.776837824	10.65383852
309	6.67196754	7.6348588	5.709076289	8.534046201	8.521503837	8.508961472
310	-0.352108471	-0.352108	-0.352108471	6.207862328	7.036396027	7.864929725
311	7.503651391	7.7121014	7.29520141	6.635453109	6.617035395	6.653870824
317	3.680058553	4.3280654	4.976072197	0.228373553	0.808855576	-0.352108471
321	-0.352108471	-0.352108	-0.352108471	6.198777144	6.706465951	7.214154759
322	6.864403563	5.3546856	3.844967735	-0.352108471	-0.352108471	-0.352108471
323	-0.352108471	-0.352108	-0.352108471	5.081130375	6.396868383	7.71260639
324	5.980334028	5.785085	6.175583018	0.440372779	0.440372779	0.440372779
325	-0.352108471	-0.352108	-0.352108471	0.95025487	2.252618212	-0.352108471
327	5.217491139	6.7680071	3.666975149	4.132568577	5.169157826	6.205747074
328	4.645411718	6.2570463	7.868680968	6.124193068	7.281493784	8.4387945
329	6.502263028	3.9725992	9.031926895	-0.352108471	-0.352108471	-0.352108471
331	9.261586072	9.1119908	9.411181337	7.861769608	8.216268226	7.507270991
332	-0.352108471	-0.352108	-0.352108471	5.488932804	6.724891066	7.960849329
334	10.13474056	7.8724594	5.610178194	6.223332356	8.121597162	10.01986197
338	5.428393874	3.3246971	7.532090623	-0.352108471	-0.352108471	-0.352108471
348	5.493783215	5.4582551	5.529311366	0.808855576	0.808855576	0.808855576
351	-0.352108471	-0.352108	-0.352108471	8.459467544	8.646691615	8.272243472
353	-0.352108471	-0.352108	-0.352108471	7.475171153	7.49196897	7.458373336
357	-0.352108471	-0.352108	-0.352108471	0.849500091	-0.352108471	2.051108653
360	-0.352108471	-0.352108	-0.352108471	5.406707798	5.458255064	5.355160532
361	5.939192221	5.5342738	6.344110621	-0.352108471	-0.352108471	-0.352108471
363	6.290509167	7.815472	7.052990603	5.861579664	5.529311366	6.193847962
367	-0.352108471	-0.352108	-0.352108471	5.275824336	8.923588102	7.099706219
372	8.890026116	6.9241703	7.907098232	8.167328804	7.364389464	6.561450124
375	7.61514139	8.9182906	10.22143975	9.473509341	9.39930777	9.325106198
377	6.260977578	6.0908083	5.920639104	10.65856038	7.898410558	5.138260735
378	5.608610738	9.992351	7.800480868	6.458834491	10.01915343	8.238993959
379	-0.352108471	-0.352108	-0.352108471	5.789762726	5.804293869	5.775231583
381	6.692933809	6.3691566	6.045379372	2.20456921	3.055408374	3.906247539
382	6.674128879	5.2100527	8.13820505	7.717952937	5.824707999	9.611197876
387	7.9386766	8.5985778	9.258478956	9.279792422	7.564747449	10.99483739
388	8.687750894	8.633343	8.578935183	12.25976023	5.146817762	8.703288996
389	9.164371527	9.161673	9.167070085	9.530068237	9.544611629	9.515524845
390	13.21877149	6.1498398	9.684305641	10.02570825	12.46515185	7.586264652
391	4.921426854	5.3546856	5.787944444	5.13952956	8.77302793	6.956278745
392	6.982138842	9.9833769	8.482757854	7.811627334	8.08488895	8.358150566
396	9.364505851	9.791867	9.57818642	9.667940649	7.414752691	11.92112861
400	-0.352108471	-0.352108	-0.352108471	8.384281252	5.543702138	6.963991695
402	3.515141236	3.6761388	3.354143641	4.884610637	7.135358184	6.00998441

403	6.090808341	5.2346703	6.946946418	6.497182024	2.612247117	10.38211693
405	3.127737875	3.6858981	2.569577624	8.174087603	6.434644758	4.695201913
408	5.800199788	5.308056	6.292343569	-0.352108471	-0.352108471	-0.352108471
411	5.073617205	4.617923	5.529311366	5.605971047	5.054280952	6.157661143
412	5.106529895	5.3284524	5.217491139	-0.352108471	-0.352108471	-0.352108471
414	3.515141236	3.6761388	3.354143641	6.235921161	6.246629235	6.225213087
420	5.118118228	5.2174911	5.31686405	-0.352108471	-0.352108471	-0.352108471
427	-0.352108471	-0.352108	-0.352108471	5.939192221	4.675625004	7.202759439
432	6.909491214	5.2174911	3.525491064	-0.352108471	-0.352108471	-0.352108471
435	-0.352108471	-0.352108	-0.352108471	7.329165582	5.080146934	6.204656258
438	2.239158726	4.8304259	-0.352108471	-0.352108471	-0.352108471	-0.352108471
439	5.066682906	4.6906481	4.878665478	8.876002641	6.666110637	7.771056639
444	4.890628796	4.4996979	4.108766905	-0.352108471	-0.352108471	-0.352108471
445	7.663530734	3.9368688	5.800199788	0.440372779	0.440372779	0.440372779
463	-0.352108471	-0.352108	-0.352108471	5.233567424	5.254689875	5.212444972
468	9.457065222	3.0570275	6.257046343	8.554431701	5.469789537	11.63907387
471	1.880238567	2.7034702	1.057006959	7.442293468	6.129992934	6.786143201
472	9.646048815	6.0199671	7.833007954	2.898183655	3.919505936	1.876861374
473	-0.352108471	-0.352108	-0.352108471	2.16675574	2.232575625	2.100935855
475	7.359936979	6.1875446	6.77374078	6.533419766	6.285554459	6.037689151
476	0.953592283	1.6620152	2.370438077	2.252618212	2.132840388	2.372396036
477	5.940759291	5.6532998	6.228218771	4.340882501	3.615460478	5.066304524
478	-0.352108471	-0.352108	-0.352108471	1.167362396	2.686833264	-0.352108471
482	6.578185475	5.3713898	7.784981108	5.969272276	6.533156242	5.405388309
483	7.17777694	7.6716722	6.683881727	6.947225037	7.483004983	8.01878493
488	8.186333712	7.1522542	7.669293948	8.122214126	6.711695252	9.532732999
490	-0.352108471	-0.352108	-0.352108471	4.158791148	5.290388732	4.72458994
491	-0.352108471	-0.352108	-0.352108471	0.049668827	3.766938612	1.90830372
493	6.249497805	6.8018138	7.354129742	6.515493975	6.693937107	6.872380239
498	3.127737875	3.136327	3.119148782	9.081034608	9.082863108	9.079206108
501	-0.352108471	-0.352108	-0.352108471	6.039960775	7.013360586	7.986760398
506	6.927852695	9.3478814	8.137867046	8.363135134	8.794263144	9.225391154
509	5.77793971	10.063575	7.92075757	7.730705218	7.49251651	7.254327801
513	-0.352108471	-0.352108	-0.352108471	6.314855501	5.349381241	4.383906981
514	-0.130115716	1.3921092	2.914334214	6.971719777	6.413975447	5.856231116
515	4.190287334	4.6499796	5.109671904	7.843714581	3.080683113	5.462198847
516	-0.352108471	-0.352108	-0.352108471	6.12990004	6.135204448	6.124595632
517	6.197324086	8.2753551	7.236339591	6.594046641	8.408750976	7.501398809
518	6.556689902	4.7784383	8.334941473	6.773169268	9.393501154	8.083335211
521	6.40026323	7.5095361	5.290990324	2.204376426	-0.352108471	4.760861323
522	-0.352108471	-0.352108	-0.352108471	8.496595026	5.386616615	6.94160582
523	6.666809909	6.9959352	7.325060451	4.477947052	6.039058617	2.916835486
526	6.983643214	5.8100936	6.396868383	9.828742258	4.017847844	6.923295051
527	8.179775135	7.7509518	7.322128515	7.608785314	8.474905279	9.341025245
529	6.801813774	6.5092942	7.094333337	7.133055561	7.257115123	7.008995998
533	6.242048279	6.2558778	6.228218771	1.293955958	0.440372779	2.147539136
535	4.095303961	3.136327	5.054280952	0.044132154	0.440372779	-0.352108471

537	7.850078263	6.2767493	7.063413806	5.764101034	7.054600764	8.345100495
545	2.907929169	2.3521085	3.167966808	-0.352108471	-0.352108471	-0.352108471
546	-0.352108471	-0.352108	-0.352108471	4.953000063	3.314032311	1.675064559
550	-0.352108471	-0.352108	-0.352108471	5.704335255	5.715557343	5.693113166
552	7.090140524	6.6903025	6.29046443	1.434979174	-0.260288665	3.130247014
558	5.536335589	8.8499125	7.193124059	8.644778721	8.754362158	8.535195284
567	6.792524508	7.6476165	8.502708572	9.019842152	9.6608816	8.378802703
570	5.017136436	5.3751815	4.659091321	-0.352108471	-0.352108471	-0.352108471
572	5.800199788	1.7608913	9.839508267	9.012929553	6.556689902	4.10045025
573	5.002970041	5.4527237	5.902477441	6.641081844	4.80208491	2.963087977
578	5.089412202	5.1245435	5.054280952	5.55876787	5.588224375	5.529311366
581	6.183964016	9.1800156	3.1879124	7.625397823	6.100474217	6.86293602
587	2.342270555	4.7923037	7.242336783	-0.352108471	-0.352108471	-0.352108471
594	4.579618372	5.4673241	3.691912626	7.060560369	4.816592136	5.938576252
595	7.662449217	6.8549116	8.469986802	8.461492316	9.693261594	9.077376955
598	-0.352108471	-0.352108	-0.352108471	7.353725402	7.474040996	7.233409808
602	-0.352108471	-0.352108	-0.352108471	4.455603148	6.713749539	5.584676343
604	-0.352108471	-0.352108	-0.352108471	5.640498569	5.760537073	5.880575577
607	5.749402114	4.4694506	7.029353582	-0.352108471	-0.352108471	-0.352108471
613	-0.352108471	-0.352108	-0.352108471	2.368970958	2.686833264	2.051108653
617	-0.352108471	-0.352108	-0.352108471	8.019856501	6.251034844	4.482213187
618	6.498998083	3.4566386	9.541357538	7.093676226	6.742862436	6.392048646
622	4.612603102	2.7802203	6.444985911	1.290035436	1.14221151	1.437859363
624	-0.352108471	-0.352108	-0.352108471	8.605277903	6.577483275	4.549688648
627	-0.352108471	-0.352108	-0.352108471	5.462380241	5.526454112	5.494417177
633	1.66985715	-0.352108	3.69182277	7.381849155	7.396922722	7.366775589
642	-0.352108471	-0.352108	-0.352108471	6.5321479	6.523214599	6.896541123
642	-0.352108471	-0.352108	-0.352108471	6.296043865	5.91480293	6.677284801
644	-0.352108471	-0.352108	-0.352108471	3.515778726	5.095423988	6.67506925
649	-0.352108471	-0.352108	-0.352108471	8.252551998	3.887535935	6.070043966
653	6.183964016	7.5240207	4.843907301	0.228373553	0.808855576	-0.352108471
654	-0.352108471	-0.352108	-0.352108471	2.525716947	2.455378162	2.596055731
655	8.278152679	7.1605245	6.042896243	6.498474599	4.243989136	8.752960063
656	4.780251272	3.5151412	2.250031199	3.97076157	5.299716099	2.64180704
657	3.127737875	3.136327	3.119148782	5.663737322	5.665786693	5.661687951
659	3.624456288	3.136327	4.112585606	7.965186454	7.153114613	7.559150534
660	8.225473264	6.502263	4.779052792	-0.352108471	-0.352108471	-0.352108471
663	6.032383908	7.3440039	8.655623821	-0.352108471	-0.352108471	-0.352108471
664	5.631148917	4.8753327	5.253240792	7.430793515	4.148731937	5.789762726
666	6.012092159	7.9936758	9.97525947	7.502040615	6.478723709	8.525357521
667	5.586906204	5.7155573	5.458255064	7.75831886	7.675016565	7.841621156
668	7.717914505	6.8007694	7.259341956	2.024343053	2.051108653	1.997577452
672	7.404619826	5.7252834	9.083956246	5.48586401	5.383503645	5.588224375
675	7.625489951	5.3215488	8.985632148	-0.352108471	-0.352108471	-0.352108471
677	-0.352108471	-0.352108	-0.352108471	6.688193135	7.43837927	7.063286202
678	4.983275232	6.0203811	7.057486951	9.540558217	5.875707677	7.708132947
682	7.042523083	7.530598	6.554448161	6.693541633	13.12881009	9.91117586

686	-0.352108471	-0.352108	-0.352108471	1.610456079	2.813430359	2.211943219
687	6.396071861	5.9002924	5.4045129	-0.352108471	-0.352108471	-0.352108471
688	-0.352108471	-0.352108	-0.352108471	5.529311366	5.0088957	6.049727031
689	6.826791329	7.3241242	7.821457072	6.15922378	8.283427196	10.40763061
694	8.996945065	4.9380774	6.967511245	6.561593381	4.929195545	5.745394463
696	6.020381091	7.2741943	4.766567849	-0.352108471	-0.352108471	-0.352108471
705	-0.352108471	-0.352108	-0.352108471	8.083868211	6.693937107	5.304006003
706	5.951381674	2.5390469	4.245214285	4.210192471	7.972547994	11.73490352
708	-0.352108471	-0.352108	-0.352108471	6.390243821	5.923493182	5.456742543
710	9.175028639	5.5638266	7.369427614	-0.352108471	-0.352108471	-0.352108471
713	2.250607883	7.8255702	5.038089054	-0.352108471	-0.352108471	-0.352108471
714	5.170522111	5.9470136	5.55876787	-0.352108471	-0.352108471	-0.352108471
715	-0.352108471	-0.352108	-0.352108471	5.64354012	5.499659275	5.787420965
717	-0.352108471	-0.352108	-0.352108471	5.620762093	6.535396663	4.706127522
720	-0.352108471	-0.352108	-0.352108471	1.897009512	2.147539136	1.646479888
726	5.656118409	5.2174911	4.778863869	7.64126651	5.923493182	4.205719854
727	6.640019393	6.6538708	6.626167961	0.228373553	0.808855576	-0.352108471
738	3.25230152	7.100875	5.17658824	7.1633918	6.503023606	6.833207703
740	8.903116836	7.6279844	6.352851992	0.808855576	0.808855576	0.808855576
741	4.092568267	8.9821992	6.537383725	-0.352108471	-0.352108471	-0.352108471
746	8.632066898	6.3034612	3.974855591	9.828988495	4.749167616	7.289078056
747	2.588601447	5.5293114	-0.352108471	6.104429468	6.135204448	6.073654487
754	6.888413829	6.3541128	7.422714809	6.784471269	10.10435564	8.444413455
755	2.201036472	-0.352108	4.754181416	5.466802197	5.325716078	5.184629959
757	3.221680638	3.3976438	3.573606975	4.324499914	6.636273252	8.948046589
758	7.344991552	5.8529375	4.360883371	-0.352108471	-0.352108471	-0.352108471
762	2.451290702	4.4805042	0.422077159	-0.352108471	-0.352108471	-0.352108471
765	-0.352108471	-0.352108	-0.352108471	5.003900023	4.711179846	4.857539934
770	6.239860938	5.1144544	7.365267522	6.780090392	4.547384252	5.663737322
771	5.834617878	7.3441675	6.58939268	0.624614178	0.440372779	0.808855576
773	5.817791201	5.7155573	5.920025058	-0.352108471	-0.352108471	-0.352108471
774	-0.352108471	-0.352108	-0.352108471	2.501526031	-0.352108471	5.355160532
777	-0.352108471	-0.352108	-0.352108471	0.919601171	2.191310813	-0.352108471
780	6.965092281	6.5021238	6.03915525	5.20089139	2.915740138	7.486042641
781	-0.352108471	-0.352108	-0.352108471	1.713271551	6.882006614	4.297639083
783	5.242500616	6.0023087	4.482692559	0.289051906	-0.352108471	0.930212283
790	6.463674915	5.8954858	7.031864022	6.861176432	6.912044458	6.886610445
800	9.23591817	9.2038491	9.267987234	10.94163088	11.01856029	10.86470147
801	8.14581367	8.8305437	7.461083662	10.78008366	8.643381996	6.506680336
805	8.320469331	9.4478149	10.57516051	9.906169645	8.441499074	9.17383436
808	8.912294534	7.1464808	5.380666979	7.885273527	7.191457353	7.53836544
811	9.276414114	10.840521	10.05846765	9.237906068	10.93092673	10.0844164
813	8.540899881	8.5656147	8.516185087	8.59245798	8.587057431	8.59785853
814	1.392109249	3.136327	-0.352108471	6.32531018	4.934949219	7.715671142
816	5.368848099	7.635678	6.502263028	-0.352108471	-0.352108471	-0.352108471
817	-0.352108471	-0.352108	-0.352108471	5.85533265	8.625015656	7.240174153
819	5.54524521	5.3954371	5.695053316	0.044132154	0.440372779	-0.352108471

821	7.788129335	9.3259143	8.557021809	10.88904381	8.040790482	5.192537156
823	6.408335249	8.9096683	11.41100139	6.967817429	7.32478698	7.146302205
824	5.377684625	6.8302983	8.282912046	6.380198231	6.666990727	6.523594479
826	7.741124349	8.0405176	8.339910877	8.932767957	9.934662693	7.930873222
827	-0.352108471	-0.352108	-0.352108471	3.245625856	4.677392879	1.813858833
829	5.795957397	9.1049407	7.450449055	8.202893741	8.277079148	8.351264555
830	7.494042651	5.8220038	6.658023207	5.597471128	6.662328952	6.12990004
832	5.485142306	6.9989543	6.242048279	8.695477893	7.687774172	9.703181614
833	6.502263028	7.9031079	5.101418134	7.877278125	5.767139528	6.822208827
835	6.05159641	8.1292719	7.090434151	4.811151293	3.860731867	5.76157072
836	-0.352108471	-0.352108	-0.352108471	6.175041264	5.309738611	7.040343918
838	-0.352108471	-0.352108	-0.352108471	5.923840725	6.120261125	6.022050925
839	5.591305588	5.6118052	5.570805936	5.391782839	3.52198956	4.456886199
841	6.132049638	4.4829661	7.781133204	6.984956811	8.005934009	5.963979613
842	8.961661459	4.5098979	6.735779686	4.37140445	2.798838152	3.585121301
843	-0.352108471	-0.352108	-0.352108471	7.1225757	3.236819248	5.179697474
847	7.084558672	6.7951849	6.939871798	5.734113463	6.32531018	6.916506897
857	10.26908572	8.5838269	9.426456321	10.17702126	9.516914426	10.83712809
858	7.160812723	6.7393234	7.582302	6.94300136	5.803339018	8.082663702
862	5.636758727	6.8161979	7.99563715	0.624614178	0.440372779	0.808855576
871	8.867116017	9.107832	8.626400046	6.99577964	12.85576918	9.925774412
873	-0.352108471	-0.352108	-0.352108471	6.270080905	7.269863612	8.269646319
874	5.354685649	4.4314502	6.277921075	-0.352108471	-0.352108471	-0.352108471
889	5.380093023	2.5995424	3.989817733	-0.352108471	-0.352108471	-0.352108471
890	5.79452377	5.062264	5.428393874	7.108206964	6.068301163	6.588254064
891	-0.352108471	-0.352108	-0.352108471	5.226381766	5.546527222	5.866672678
894	8.561441704	4.4016062	6.481523945	-0.352108471	-0.352108471	-0.352108471
896	-0.352108471	-0.352108	-0.352108471	6.754403141	7.196843491	6.975623316
899	6.860485731	9.2062245	4.514746954	9.131624171	6.183964016	3.236303861
900	8.684940475	7.621847	8.153393761	-0.352108471	-0.352108471	-0.352108471
902	8.06771298	6.2125362	4.357359437	-0.352108471	-0.352108471	-0.352108471
911	-0.352108471	-0.352108	-0.352108471	6.296043865	6.31523113	6.276856601
917	5.781003172	4.9317647	5.356383913	4.018737342	1.878647654	6.158827029
920	5.493783215	5.5293114	5.458255064	-0.352108471	-0.352108471	-0.352108471
928	6.056053999	6.0736545	6.038453511	5.596916991	5.605609608	5.588224375
933	5.089412202	6.6009453	3.577879136	-0.352108471	-0.352108471	-0.352108471
944	-0.352108471	-0.352108	-0.352108471	4.80208491	7.040503188	2.563666632
947	8.289573677	8.5715583	8.430566011	7.024362382	7.971523244	6.07720152
949	-0.352108471	-0.352108	-0.352108471	2.458452582	2.902991023	3.347529463
950	5.22751712	8.4348954	6.83120624	7.134555948	8.515770926	5.75334097
953	9.226601419	9.7165867	10.20657198	9.776795363	6.367865082	8.072330222
955	-0.352108471	-0.352108	-0.352108471	7.135976498	6.435912105	6.785944301
957	5.869068613	7.3456863	6.607377459	-0.352108471	-0.352108471	-0.352108471
960	9.913575227	9.9058398	9.898104288	9.372706092	11.73122264	10.55196436
961	6.398728792	4.8209485	3.24316818	4.002423202	6.566987041	1.437859363
966	7.875021286	7.102821	8.647221598	10.20223816	9.487630864	8.773023567
967	9.526140155	8.5780141	7.62988806	10.29241286	7.835247198	9.063830029

968	6.21555058	6.1523775	6.183964016	9.310177241	5.121036563	7.215606902
972	-0.352108471	-0.352108	-0.352108471	0.219081723	3.228447668	6.237813614
975	7.811707445	11.513219	9.662463025	9.125440131	7.688082324	10.56279794
976	3.424800141	6.6094727	5.017136436	6.204656258	6.195245599	6.214066917
977	8.639139696	8.4045671	8.169994563	8.09424008	9.895157546	8.994698813
978	4.471811676	5.0522677	5.632723695	5.751153254	7.178522138	8.605891021
984	-0.352108471	-0.352108	-0.352108471	2.146123334	3.081875283	1.210371385
987	7.916491175	5.8001998	3.6839084	-0.352108471	-0.352108471	-0.352108471
989	7.305341479	6.2912631	8.319419813	6.558419977	7.576043883	5.54079607
990	7.05303533	6.5345309	7.571539789	5.055622197	5.780944036	6.506265874
991	5.450287933	4.9116502	4.373012483	7.500670614	8.212686149	6.788655079
995	-0.352108471	-0.352108	-0.352108471	6.359270363	6.424327813	6.294212913
996	5.931810751	5.825168	6.038453511	6.771871898	7.386082653	6.157661143
997	3.753242592	6.1383138	8.523384972	8.483674234	5.763296942	7.123485588
998	9.031356711	8.4650146	8.748185667	7.043032659	7.349917404	7.656802148
1000	6.689878453	6.9004869	6.479269959	6.033059519	6.298681693	6.165870606
1005	5.217491139	7.9701489	2.464833418	-0.352108471	-0.352108471	-0.352108471
1010	5.001646961	6.502263	8.002879095	-0.352108471	-0.352108471	-0.352108471
1012	-0.352108471	-0.352108	-0.352108471	6.014622175	6.204656258	6.394690341
1019	5.054381279	4.3858365	4.720108865	7.623987129	7.226858928	7.425423028
1020	-0.352108471	-0.352108	-0.352108471	5.596916991	5.605609608	5.588224375
1021	5.217491139	6.0114989	4.423483399	0.044132154	-0.352108471	0.440372779
1022	6.913488898	6.7984663	7.028511527	-0.352108471	-0.352108471	-0.352108471
1026	7.541301669	4.4931056	10.58949778	7.374429005	11.67704094	9.525734971
1029	-0.352108471	-0.352108	-0.352108471	5.753543754	6.130929297	5.942236525
1031	5.15172345	5.4283939	5.705064299	-0.352108471	-0.352108471	-0.352108471
1033	3.362379091	6.2222282	4.792303669	-0.352108471	-0.352108471	-0.352108471
1041	6.607377459	6.2011696	7.013585304	-0.352108471	-0.352108471	-0.352108471
1044	-0.352108471	-0.352108	-0.352108471	5.169157826	5.966733932	4.371581719
1048	6.20615762	7.3413239	6.77374078	-0.352108471	-0.352108471	-0.352108471
1051	5.718459767	5.5596947	5.877224786	9.151931627	6.83194586	7.991938744
1052	-0.352108471	-0.352108	-0.352108471	6.810957895	4.236470138	9.385445652
1054	-0.352108471	-0.352108	-0.352108471	3.787078433	5.233567424	6.680056414
1059	8.651658241	5.3028582	6.977258204	5.428738754	6.531929302	5.980334028
1062	7.016776417	5.5311094	8.502443459	3.987437271	5.285554459	4.583671646
1063	6.242048279	6.2558778	6.228218771	5.789762726	5.804293869	5.775231583
1069	1.653087098	7.4475285	4.550307802	6.266367194	10.04199253	2.490741857
1072	9.651358833	9.6691777	9.633539928	11.29802876	11.29802876	11.29802876
1073	8.52961696	7.3777551	6.225893307	5.790806265	9.111310342	7.451058303
1075	5.42029107	5.4582551	5.496219058	10.25963782	8.063052958	5.866468096
1082	5.56838165	8.2355769	6.901979267	-0.352108471	-0.352108471	-0.352108471
1087	6.167582836	6.312044	6.023121627	-0.352108471	-0.352108471	-0.352108471
1089	7.700906227	6.4636749	5.226443602	-0.352108471	-0.352108471	-0.352108471
1101	5.813764122	6.3178342	6.821904343	-0.352108471	-0.352108471	-0.352108471
1104	-0.352108471	-0.352108	-0.352108471	3.883023856	4.178899946	4.474776035
1107	-0.352108471	-0.352108	-0.352108471	6.46748826	7.307185689	5.627790831
1108	-0.352108471	-0.352108	-0.352108471	7.668177708	9.401546237	5.934809179

1111	6.289299368	4.5523394	5.420819363	0.228373553	-0.352108471	0.808855576
1116	3.098924625	4.8786655	6.658406331	-0.352108471	-0.352108471	-0.352108471
1118	-0.352108471	-0.352108	-0.352108471	7.161038899	0.929336728	4.045187814
1121	5.49112888	5.0916696	5.291399258	0.289051906	-0.352108471	0.930212283
1122	4.513304033	4.1371548	4.889453296	5.866795484	7.197485464	4.536105503
1123	-0.352108471	-0.352108	-0.352108471	7.885718121	5.794509996	6.840114058
1124	-0.352108471	-0.352108	-0.352108471	7.230161606	3.236973241	5.233567424
1130	8.855216083	6.573969	4.292721916	-0.352108471	-0.352108471	-0.352108471
1137	2.930522656	5.4937832	8.057043774	-0.352108471	-0.352108471	-0.352108471
1138	7.480527585	6.3637627	5.246997726	3.552694803	5.054280952	2.051108653
1140	8.00369157	7.7585064	8.248876705	6.50040249	7.430061825	8.35972116
1147	-0.352108471	-0.352108	-0.352108471	5.233567424	5.254689875	5.212444972
1152	-0.352108471	-0.352108	-0.352108471	5.855547981	6.814574715	4.896521246
1163	-0.352108471	-0.352108	-0.352108471	6.933758644	6.859217306	6.784675967
1164	6.858070517	6.7871341	6.716197628	8.429817558	6.751466056	5.073114555
1173	9.756338542	9.1972611	9.47679983	10.96797735	9.263192723	7.558408099
1174	-0.352108471	-0.352108	-0.352108471	5.412561087	5.421579811	5.403542364
1175	7.548901375	6.2146173	8.883185469	8.061929517	7.248456608	8.875402425
1176	7.639635545	7.2490859	8.030185209	6.560527001	8.709328933	4.411725069
1184	7.249504893	7.352529	7.146480757	6.890254017	6.907463027	6.873045007
1186	7.939978488	7.9748192	7.905137775	7.580379813	7.507270991	7.653488636
1192	7.395172444	7.4859561	7.57673985	9.156626621	9.214066695	9.099186547
1195	8.074125067	6.8018138	5.529502481	0.440372779	0.440372779	0.440372779
1200	-0.352108471	-0.352108	-0.352108471	7.069324776	7.082394844	7.056254708
1209	6.294598926	8.6046455	7.449622211	7.758293406	5.448807611	6.603550508
1211	7.986731438	11.417429	4.5560342	7.9391614	7.999549466	7.878773335
1212	8.291850712	6.8681731	5.444495503	7.195776097	8.403075065	9.610374033
1213	9.243597871	7.4402611	5.636924288	8.584358598	8.351212473	8.118066348
1235	8.704774016	10.08633	7.323217744	6.021653764	7.0424346	8.063215436
1238	4.60818135	4.8304259	4.385936778	0.791292145	0.440372779	1.14221151
1241	4.528924884	4.671913	4.385936778	5.966446363	5.775231583	6.157661143
1244	5.821382858	5.4289387	5.625160771	-0.352108471	-0.352108471	-0.352108471
1258	-0.352108471	-0.352108	-0.352108471	1.347778277	3.157458146	2.252618212
1260	8.697922221	4.7434709	6.720696571	-0.352108471	-0.352108471	-0.352108471
1263	-0.352108471	-0.352108	-0.352108471	7.186511196	6.96115987	7.411862521
1264	6.448936284	9.1633811	7.806158706	7.191902102	5.526141656	8.857662547
1265	7.625917328	8.6327219	8.129319611	11.47857173	7.917494695	9.698033212
1266	-0.352108471	-0.352108	-0.352108471	6.631846251	10.18338295	3.080309554
1267	2.739370013	7.8305787	5.284974356	5.40346407	7.098605618	6.251034844
1277	10.84527839	11.693655	12.54203144	11.44566657	13.04193267	12.24379962
1278	9.860304297	10.009607	9.934955428	10.0105711	10.89311925	11.7756674
1281	6.948029234	8.7744596	10.60088992	9.457277455	9.275289669	9.093301882
1282	9.476044814	7.6827213	11.26936835	9.553777807	11.85439279	7.253162824
1284	10.47703907	10.553027	10.62901523	10.56204105	9.807752056	10.18489655
1287	8.120279852	5.2174911	2.314702426	-0.352108471	-0.352108471	-0.352108471
1288	-0.352108471	-0.352108	-0.352108471	3.921118155	2.211943219	0.502768283
1289	7.980118636	9.6136567	6.346580545	7.896622182	9.253957459	8.575289821

1290	6.590508304	6.6215439	6.559472732	8.480322745	8.489801751	8.470843739
1291	10.41668396	10.223009	10.02933476	8.034656863	10.34644693	9.190551899
1292	9.676504791	9.6477625	9.705247115	8.028981969	8.039791301	8.018172636
1293	9.286219017	7.2523995	8.269309258	9.204135884	8.986005302	9.422266466
1294	5.307588256	6.8045197	6.056053999	3.413036741	5.624064457	7.835092173
1297	4.792303669	5.649381	3.935226313	-0.352108471	-0.352108471	-0.352108471
1303	5.313762023	7.3511713	6.332466669	5.578817747	6.829307464	8.07979718
1307	-0.352108471	-0.352108	-0.352108471	4.673928722	5.637296679	6.600664635
1310	8.134713191	4.8015905	6.468151867	3.258303997	5.241633112	7.224962226
1312	7.197039856	4.4033597	5.800199788	10.7824872	4.023360718	7.40292396
1314	6.759189408	4.5503078	2.341426195	5.563079715	7.68524044	6.624160077
1315	6.303461245	4.4146004	8.192322072	8.185377	5.205994566	6.695685783
1316	-0.352108471	-0.352108	-0.352108471	4.021399483	4.201099247	4.380799012
1317	9.340136108	9.2849746	9.229813057	8.050338643	8.072630865	8.028046422
1327	7.398193617	6.1145004	8.68188685	7.523355179	8.22062535	8.917895522
1329	6.573968999	8.517611	4.63032698	1.35857476	1.884572605	1.621573683
1337	4.434314669	7.4034708	1.465158581	7.81588956	8.907273265	6.724505854
1340	5.384513167	6.2084328	7.032352386	6.823179934	9.443194752	8.133187343
1345	-0.352108471	-0.352108	-0.352108471	6.936608066	6.432268644	7.440947487
1346	-0.352108471	-0.352108	-0.352108471	5.596312349	7.094238567	6.345275458
1347	-0.352108471	-0.352108	-0.352108471	5.859617369	5.873345987	5.845888751
1349	-0.352108471	-0.352108	-0.352108471	2.252618212	2.252618212	2.252618212
1350	9.145134675	5.9571377	7.551136183	6.981201065	6.892803031	6.937002048
1351	2.065292044	1.0538496	3.076734511	8.208715124	7.001625016	7.60517007
1355	6.073062111	5.9744272	6.171697018	-0.352108471	-0.352108471	-0.352108471
1357	4.417353062	5.7155573	3.119148782	-0.352108471	-0.352108471	-0.352108471
1368	8.150584921	6.0473337	7.098959317	7.004567807	7.124816589	6.884319024
1371	6.573968999	6.5884653	6.559472732	-0.352108471	-0.352108471	-0.352108471
1374	5.695541451	11.378911	8.537226068	7.249046889	6.620433142	6.934740015
1376	3.969479587	8.0521799	6.01082974	8.75162142	7.119119679	5.486617937
1378	5.94941124	8.1603775	3.738444995	2.552253116	1.549724107	3.554782125
1382	2.93733314	4.7311114	6.524889604	7.027375773	7.169571732	6.885179814
1384	7.202560832	5.9453772	6.573968999	-0.352108471	-0.352108471	-0.352108471
1385	-0.352108471	-0.352108	-0.352108471	5.198791292	6.77374078	8.348690268
1386	5.852937462	5.1060484	6.599826493	-0.352108471	-0.352108471	-0.352108471
1387	-0.352108471	-0.352108	-0.352108471	7.052848865	5.88700099	4.721153114
1388	7.871775243	5.0894122	2.307049161	7.057424469	4.367102386	5.712263427
1397	-0.352108471	-0.352108	-0.352108471	3.245625856	4.201533236	2.289718476
1398	4.690462568	6.2728949	7.855327149	7.257788984	7.57386965	6.941708318
1399	-0.352108471	-0.352108	-0.352108471	4.665348107	5.605048166	6.544748225
1406	9.107342156	10.544079	7.670605405	11.63434175	7.658347728	3.68235371
1407	-0.352108471	-0.352108	-0.352108471	6.434644758	7.770498513	5.098791003
1410	6.720370627	6.7508628	6.689878453	-0.352108471	-0.352108471	-0.352108471
1415	-0.352108471	-0.352108	-0.352108471	6.735779686	5.92910625	7.542453123
1416	7.582478247	7.0736263	6.564774331	6.298963122	3.002629051	9.595297192
1420	-0.352108471	-0.352108	-0.352108471	0.95025487	2.252618212	-0.352108471
1431	2.221508879	2.4458255	2.670142181	0.495450612	3.245625856	5.995801099

1434	7.33826098	7.0253499	6.712438841	-0.352108471	-0.352108471	-0.352108471
1435	-0.352108471	-0.352108	-0.352108471	7.036396027	8.565938324	5.506853729
1436	7.494467498	6.5373837	5.580299951	0.044132154	-0.352108471	0.440372779
1437	6.897089188	6.1071583	6.502123765	-0.352108471	-0.352108471	-0.352108471
1439	-0.352108471	-0.352108	-0.352108471	6.022050925	6.038453511	6.005648339
1445	5.440482721	6.3691566	7.29783046	2.124237496	2.147539136	2.100935855
1452	4.533588456	7.9232175	6.228402992	-0.352108471	-0.352108471	-0.352108471
1453	5.993679345	4.8631084	5.428393874	6.625595045	4.604759169	8.646430922
1455	7.06641971	7.0919708	7.040868605	7.186150958	7.149537332	7.222764584
1457	6.537383725	4.9935512	8.081216298	-0.352108471	-0.352108471	-0.352108471
1459	5.354685649	4.7467312	5.9626401	-0.352108471	-0.352108471	-0.352108471
1460	5.625160771	5.6532998	5.597021732	-0.352108471	-0.352108471	-0.352108471
1462	1.383520155	-0.352108	3.119148782	5.017136436	8.15969518	1.874577691
1463	-0.352108471	-0.352108	-0.352108471	6.313887715	6.89992346	7.485959206
1464	-0.352108471	-0.352108	-0.352108471	7.852007698	7.640414475	8.063600921
1465	6.76919815	6.7357797	6.702361222	11.64597639	9.561320885	7.47666538
1466	6.700648212	6.4472898	6.573968999	-0.352108471	-0.352108471	-0.352108471
1468	-0.352108471	-0.352108	-0.352108471	5.511864271	4.66078572	6.362942822
1471	8.461910603	8.115481	8.288695803	7.203844697	10.13338184	8.668613267
1472	-0.352108471	-0.352108	-0.352108471	3.949389832	1.14386139	2.546625611
1476	7.309433975	6.8173936	7.063413806	8.790704847	7.763124624	6.735544401
1477	-0.352108471	-0.352108	-0.352108471	0.762376451	-0.352108471	1.876861374
1488	8.764937771	6.2420483	3.719158787	6.752359668	6.22033882	5.688317973
1489	-0.352108471	-0.352108	-0.352108471	6.016006936	8.784732755	3.247281116
1490	9.523670388	9.1421272	8.760583996	9.046626379	9.032143713	9.061109044
1491	5.579737679	3.5128398	4.546288763	1.556591723	1.305230684	1.807952763
1495	7.784633114	6.3324667	4.880300224	10.69379613	8.438433036	6.18306994
1497	5.935025625	3.7087213	4.821873485	1.345967809	1.14221151	1.549724107
1499	4.33467897	7.2657206	5.800199788	7.929880674	7.326356364	6.722832055
1503	7.98886211	4.8048747	6.396868383	5.233567424	4.890145737	5.57698911
1508	11.52352709	10.93352	10.34351316	10.4302022	10.26135335	10.09250451
1509	3.90477055	6.0218323	4.963301433	8.879459618	7.823295134	9.935624102
1513	3.218832558	6.4142581	9.609683714	8.383410412	9.718972752	7.047848073
1514	-0.352108471	-0.352108	-0.352108471	6.35960917	6.396868383	6.434127595
1515	4.239379986	3.9195059	4.559254036	5.596916991	5.605609608	5.588224375
1520	2.465331273	4.3155755	6.165819791	1.07271249	3.680662938	6.288613386
1521	-0.352108471	-0.352108	-0.352108471	2.065292044	4.482692559	-0.352108471
1522	3.038997464	3.991285	3.515141236	3.857433443	7.861801295	5.859617369
1523	5.774370319	7.934825	3.613915593	-0.352108471	-0.352108471	-0.352108471
1528	-0.352108471	-0.352108	-0.352108471	3.742430852	3.36730463	2.992178408
1534	4.559245127	6.536272	8.51329891	4.696144107	6.434047684	5.565095895
1535	7.228191745	5.6886226	4.149053549	6.372987249	6.274700315	6.471274184
1537	6.735779686	6.9729333	6.498626103	8.040262919	6.070317529	10.01020831
1540	-0.352108471	-0.352108	-0.352108471	3.147139248	5.181747782	7.216356316
1544	6.497227402	5.3203266	7.674128237	4.042376545	2.122618472	5.962134619
1556	5.810073753	7.5059727	6.658023207	1.689109503	1.203948072	2.174270934
1557	-0.352108471	-0.352108	-0.352108471	4.456886199	3.256017866	5.657754533

1564	-0.352108471	-0.352108	-0.352108471	1.943550341	2.605481072	2.274515706
1576	5.749402114	5.6913103	5.807493886	0.542875446	1.437859363	-0.352108471
1577	6.573968999	6.5884653	6.559472732	-0.352108471	-0.352108471	-0.352108471
1579	4.687513284	5.0027451	4.372281514	6.063922398	3.519327359	0.974732319
1582	1.383520155	-0.352108	3.119148782	4.306267192	1.610816196	2.958541694
1595	7.04217785	5.9025329	6.472355382	7.645189468	3.545809849	5.595499658
1600	2.391332518	2.7115616	3.031790594	-0.352108471	-0.352108471	-0.352108471
1601	7.726811751	8.2032738	8.679735909	6.414111315	9.416168623	7.915139969
1603	8.58611904	4.418407	6.502263028	5.578297053	9.46592144	7.522109247
1604	4.117428416	3.1277379	2.138047335	0.897715333	-0.352108471	2.147539136
1605	6.735779686	6.3640494	7.107509939	7.031565324	9.285063726	4.778066922
1606	6.433318728	6.4453648	6.421272681	3.585121301	3.615460478	3.554782125
1607	6.427821161	6.576322	6.279320371	7.516066548	5.229907951	6.372987249
1611	4.779005767	5.463922	6.148838283	6.554379124	8.110056793	9.665734462
1612	-0.352108471	-0.352108	-0.352108471	5.704335255	5.030218483	6.378452027
1613	7.972087265	7.4582829	6.944478504	5.793695433	8.563348842	7.178522138
1615	5.235100382	5.3546856	5.474270916	7.421205912	6.276856601	5.132507289
1616	6.761519244	7.1735032	6.967511245	8.246108508	5.439917437	2.633726367
1621	6.104558933	6.8029865	5.406131377	0.044132154	0.440372779	-0.352108471
1627	6.257046343	4.8636031	7.650489632	9.811219085	8.166951604	8.989085344
1630	-0.352108471	-0.352108	-0.352108471	3.342992021	1.660060041	2.501526031
1633	-0.352108471	-0.352108	-0.352108471	7.748025547	4.098960817	5.923493182
1634	6.501268473	7.478342	5.524194952	2.497057926	4.158869237	5.820680548
1635	8.23472817	7.8220941	8.028411128	8.969985822	7.621334376	6.27268293
1636	6.056053999	6.0736545	6.038453511	-0.352108471	-0.352108471	-0.352108471
1639	6.939871798	4.9916691	8.888074492	8.611103295	7.458282885	6.305462474
1641	5.994260948	6.4917622	5.496759717	1.294339716	4.477947052	7.661554387
1642	7.611801192	7.9327429	8.253684552	6.481983938	7.91687825	9.351772563
1647	6.362251997	4.8880695	5.625160771	-0.352108471	-0.352108471	-0.352108471
1649	-0.352108471	-0.352108	-0.352108471	2.43016825	0.980363921	3.879972579
1650	3.852950883	5.8528638	7.852776811	7.660368015	7.422837243	7.185306472
1652	4.792303669	4.8000217	4.784585601	0.689815323	-0.352108471	1.731739118
1653	4.564574005	9.157541	6.861057494	6.931936377	9.195295815	4.668576939
1654	7.849697843	3.1277379	-1.594222092	6.235921161	7.11865072	5.353191602
1655	8.899662105	8.8097666	8.719871078	7.753529229	10.98008996	4.526968496
1656	7.993113414	9.9348067	6.051420176	8.232552636	7.483004983	6.73345733
1659	-0.352108471	-0.352108	-0.352108471	6.385933003	4.493901872	5.439917437
1660	-0.352108471	-0.352108	-0.352108471	6.946330252	7.409897489	6.482763014
1662	6.332466669	6.8958282	5.769105167	-0.352108471	-0.352108471	-0.352108471
1665	-0.352108471	-0.352108	-0.352108471	6.631846251	7.028891053	6.23480145
1674	-0.352108471	-0.352108	-0.352108471	3.721462565	1.117651711	2.419557138
1676	8.510192321	8.5854667	8.660741086	6.32450851	8.843499506	3.805517514
1687	-0.352108471	-0.352108	-0.352108471	8.852394722	5.494417177	2.136439632
1716	-0.352108471	-0.352108	-0.352108471	6.604209278	5.896786008	7.311632548
1717	-0.352108471	-0.352108	-0.352108471	6.949901658	5.198878666	8.70092465
1718	-0.352108471	-0.352108	-0.352108471	7.06548215	7.065023967	7.064565785
1719	5.502018749	4.8024755	5.152247113	5.325716078	5.958603556	4.6928286

1723	6.137515038	5.7155573	6.559472732	5.979239285	6.038453511	5.920025058
1727	8.084585015	8.704899	7.464271031	8.727925041	10.50968422	9.618804633
1728	7.387645813	7.0049838	7.770307784	6.75383721	10.89039143	8.822114318
1729	-0.352108471	-0.352108	-0.352108471	7.094111584	6.981982635	6.869853687
1734	-0.352108471	-0.352108	-0.352108471	7.209478834	7.21486974	7.204087928
1736	3.239461324	7.46991	5.354685649	6.844297079	5.07242169	5.958359385
1738	4.397603875	5.2878552	6.178106535	1.036211897	1.14221151	0.930212283
1742	4.392034833	5.1925725	4.792303669	1.800562285	2.190292434	1.995427359
1744	2.740210233	5.9595841	-0.479163617	-0.352108471	-0.352108471	-0.352108471
1748	-0.352108471	-0.352108	-0.352108471	6.368276372	5.704335255	5.040394138
1755	2.65059567	5.6532998	-0.352108471	-0.352108471	-0.352108471	-0.352108471
1759	5.939192221	3.9100937	7.968290767	-0.352108471	-0.352108471	-0.352108471
1760	5.604099007	6.6741289	7.74415875	9.034746538	7.787391935	6.540037333
1762	5.320563245	4.6942966	5.946829881	5.115331184	5.958359385	6.801387585
1770	-0.352108471	-0.352108	-0.352108471	8.18395236	4.73465025	6.459301305
1773	6.425543059	4.4779804	5.45176171	2.509108481	1.540978788	0.572849094
1774	6.640019393	6.6538708	6.626167961	5.983736712	6.002308672	5.965164752
1777	3.636824788	3.9195059	3.354143641	6.713776402	6.698467242	6.729085562
1778	7.607131056	10.035471	5.178791349	7.949702276	6.468891051	9.430513501
1780	-0.352108471	-0.352108	-0.352108471	5.174371869	-0.009187284	2.582592293
1783	-0.352108471	-0.352108	-0.352108471	6.64789689	4.376964605	8.918829175
1785	7.58670563	5.3139	6.450302837	3.731848435	7.115488819	10.4991292
1786	3.972927419	1.8163872	2.894657322	4.237109033	6.225242708	5.23117587
1787	4.019059497	0.9546997	2.486879591	0.791292145	0.440372779	1.14221151
1789	6.01696972	4.8238115	5.420390631	-0.352108471	-0.352108471	-0.352108471
1791	-0.352108471	-0.352108	-0.352108471	5.738628126	5.731413688	5.745842563
1793	6.478389984	6.502263	6.526136073	5.669790487	5.980334028	6.290877569
1798	-0.352108471	-0.352108	-0.352108471	4.400452081	4.688318557	4.112585606
1802	-0.352108471	-0.352108	-0.352108471	0.897715333	-0.352108471	2.147539136
1804	1.383520155	-0.352108	3.119148782	7.317532598	4.726569252	6.022050925
1808	6.288510867	5.1178178	7.459203977	1.76984594	1.807952763	1.731739118
1809	4.89713088	6.1679668	7.438802737	7.419106394	6.388960531	8.449252258
1810	6.920625702	5.463922	4.007218348	6.650022381	6.192730084	5.735437788
1812	6.213940521	5.4283939	4.642847227	-0.352108471	-0.352108471	-0.352108471
1813	2.807148571	2.4868796	2.166610612	-0.352108471	-0.352108471	-0.352108471
1814	-0.352108471	-0.352108	-0.352108471	2.686833264	1.631891607	3.74177492
1819	-0.352108471	-0.352108	-0.352108471	6.588254064	6.617035395	6.559472732
1820	7.61631181	5.8244294	6.720370627	2.539666369	5.818133522	4.178899946
1821	-0.352108471	-0.352108	-0.352108471	1.903060263	2.803125457	3.70319065
1822	6.390641462	5.3152335	5.852937462	7.999667835	9.195254682	8.597461258
1823	3.162868822	6.5053351	4.834101937	4.206460178	4.469833947	4.338147062
1825	10.174427	9.1628826	8.151338105	6.585491922	6.225162754	5.864833586
1829	9.22003869	7.7920339	6.364029159	7.028432398	8.712381009	5.344483787
1832	6.463674915	6.479333	6.448016836	-0.352108471	-0.352108471	-0.352108471
1836	6.964857347	6.6853616	7.244353071	5.680782182	5.179697474	4.678612766
1837	-0.352108471	-0.352108	-0.352108471	3.064586224	3.98563911	2.143533338
1839	6.991795048	7.6108104	6.372779721	-0.352108471	-0.352108471	-0.352108471

1841	-0.352108471	-0.352108	-0.352108471	6.12990004	6.135204448	6.124595632
1843	6.991795048	7.9553377	6.028252401	6.314219388	9.449713385	7.881966386
1844	3.139575061	2.5232121	1.906849153	0.044132154	0.440372779	-0.352108471
1845	-0.352108471	-0.352108	-0.352108471	7.271984511	2.332185309	4.80208491
1847	1.829261322	5.0171364	8.205011549	-0.352108471	-0.352108471	-0.352108471
1848	6.874885195	3.8319206	9.917849822	-0.352108471	-0.352108471	-0.352108471
1849	-0.352108471	-0.352108	-0.352108471	6.829307464	6.845389604	6.813225323
1850	6.138313782	5.5386983	6.737929236	-0.352108471	-0.352108471	-0.352108471
1854	-0.352108471	-0.352108	-0.352108471	3.144788429	5.508216963	4.326502696
1855	7.252200787	6.4636749	5.675149042	7.245270264	2.340745104	4.793007684
1857	6.13125595	6.6872837	7.243311444	7.482997679	8.530268819	6.435726539
1861	-0.352108471	-0.352108	-0.352108471	5.606077102	6.022050925	6.438024749
1869	-0.352108471	-0.352108	-0.352108471	5.284974356	3.955367621	6.614581091
1870	4.468125456	6.7008873	8.933649045	4.365345369	6.420886033	5.393115701
1871	-0.352108471	-0.352108	-0.352108471	4.872224191	4.843308497	4.901139884
1874	5.272495208	5.5842925	5.428393874	7.259636323	6.312650079	6.786143201
1877	6.97962556	6.7357797	6.491933812	-0.352108471	-0.352108471	-0.352108471
1880	5.244074591	5.9002924	6.55651017	-0.352108471	-0.352108471	-0.352108471
1889	7.799069185	8.6117255	8.205397321	7.459585129	7.927290912	7.69343802
1892	1.383520155	-0.352108	3.119148782	7.412333057	7.426473409	7.398192705
1893	6.212536209	6.2312245	6.193847962	8.252081739	8.269909997	8.234253481
1902	-0.352108471	-0.352108	-0.352108471	8.269807893	4.629915937	6.449861915
1903	4.713047203	3.2148412	6.211253202	9.145497431	5.938179821	7.541838626
1911	-0.352108471	-0.352108	-0.352108471	6.958673191	6.517963851	6.07725451
1912	-0.352108471	-0.352108	-0.352108471	6.799877676	5.369332089	3.938786501
1915	10.71300492	11.833459	11.27323211	12.67936545	12.85655788	12.76796167
1917	-0.352108471	-0.352108	-0.352108471	3.009468748	2.348496817	1.687524887
1933	1.66201518	3.6761388	-0.352108471	7.474897882	7.507270991	7.442524773
1936	6.819677119	6.6538708	6.985483415	8.579128565	8.610681049	8.547576081
1937	8.721951213	9.6226099	10.52326854	10.00821774	9.454144854	10.56229063
1938	10.01889761	11.038047	8.999748397	11.11443299	13.45612985	8.772736119
1947	3.127737875	3.136327	3.119148782	5.957990428	6.345275458	6.732560487
1951	7.551136183	5.216011	9.886261346	4.718925108	2.937235468	3.828080288
1953	10.32261405	7.1837671	4.044920243	-0.352108471	-0.352108471	-0.352108471
1954	7.358525885	6.9543587	7.156442283	6.448016836	6.191251389	6.704782282
1955	6.075624757	5.6682361	6.483013419	3.081409479	1.731739118	4.43107984
1956	4.417353062	5.7155573	3.119148782	-0.352108471	-0.352108471	-0.352108471
1958	6.94286187	8.086273	9.229684161	8.087563681	7.637943112	7.188322542
1960	-0.352108471	-0.352108	-0.352108471	1.995427359	3.048961466	0.941893252
1964	1.783698732	0.5510803	3.016317123	6.602327568	9.052108333	7.82721795
1965	-0.352108471	-0.352108	-0.352108471	5.88700099	4.770743989	7.003257991
1967	-0.352108471	-0.352108	-0.352108471	5.430776207	6.288458531	5.859617369
1968	3.238451656	8.9470353	6.092743499	7.47686742	6.000603181	8.953131659
1969	7.415794329	4.4367298	10.39485886	8.03312524	5.747533079	6.89032916
1974	-0.352108471	-0.352108	-0.352108471	6.762354676	8.879871748	4.644837604
1975	5.210557151	7.7832115	10.35586577	7.716233411	7.411296222	7.563764816
1977	-0.352108471	-0.352108	-0.352108471	6.60629489	7.364389464	8.122484038

1978	3.779783043	3.1277379	2.475692708	8.184171588	6.311517561	7.247844574
1979	4.038879774	2.8988472	5.17891231	8.931389383	6.810729352	7.871059368
1981	7.232769588	7.0871828	6.941596071	6.275940615	9.817325738	8.046633176
1982	8.550581697	6.8821228	10.21904061	9.373381371	9.394474737	9.352288005
1983	-0.352108471	-0.352108	-0.352108471	5.068424575	8.56150726	6.814965918
1984	8.157306673	10.173586	6.141027042	8.871451931	8.249712725	9.493191137
1992	3.047954182	-0.352108	6.448016836	6.69485278	6.813225323	6.576480236
1994	5.90029238	5.9200251	5.880559702	5.147625378	5.315258837	4.979991919
1999	-0.352108471	-0.352108	-0.352108471	2.211943219	1.659465911	2.764420527
2000	6.273832259	8.9728684	3.574796159	6.881092742	6.830443436	6.779794129
2002	5.59604745	5.7494021	5.902756779	6.682822443	8.640940677	4.724704208
2005	7.217370652	5.7873247	6.502347686	0.647185709	1.646479888	-0.352108471
2009	4.534306991	8.5404605	6.537383725	0.647185709	-0.352108471	1.646479888
2010	-0.352108471	-0.352108	-0.352108471	6.022050925	8.197541932	3.846559918
2012	9.900657447	7.8566258	5.812594129	7.916361598	6.541009048	5.165656497
2013	6.588414849	8.8690477	7.728731298	8.229724893	3.600081514	5.914903203
2014	7.224081961	9.2546956	5.193468359	6.761780888	9.219513114	7.990647001
2017	7.449848118	7.508442	7.391254223	7.737676913	7.763252455	7.712101371
2022	9.236645641	9.324648	9.148643248	10.6646886	8.253523763	5.842358922
2023	9.645977583	8.7405815	10.55137369	10.01770257	12.00381431	8.031590818
2029	-0.352108471	-0.352108	-0.352108471	5.089408562	4.80208491	4.514761259
2030	-0.352108471	-0.352108	-0.352108471	4.80208491	4.843308497	4.760861323
2034	7.932742872	7.960348	7.905137775	6.160141861	6.246629235	6.073654487
2035	4.449748927	6.0051143	2.894383523	5.645239247	4.375206621	5.010222934
2036	4.896826685	8.6774415	6.787134073	-0.352108471	-0.352108471	-0.352108471
2037	-0.352108471	-0.352108	-0.352108471	5.693086522	6.631846251	7.570605981
2040	7.496938343	6.5488069	8.445069752	-0.352108471	-0.352108471	-0.352108471
2041	-0.352108471	-0.352108	-0.352108471	4.927679215	6.093580961	3.761777469
2043	-0.352108471	-0.352108	-0.352108471	4.233458039	4.68031436	4.456886199
2048	6.446690805	7.4143359	5.479045719	-0.352108471	-0.352108471	-0.352108471
2050	4.275930666	2.7543518	3.515141236	7.86582773	7.342844162	8.388811297
2052	6.833304259	5.8023642	6.317834233	4.878842175	6.514259464	5.69655082
2054	5.420819363	5.8812424	4.960396365	3.120807694	2.686833264	3.554782125
2055	-0.352108471	-0.352108	-0.352108471	8.465812629	4.927679215	1.389545801
2058	-0.352108471	-0.352108	-0.352108471	5.529311366	2.623713044	8.434909688
2059	-0.352108471	-0.352108	-0.352108471	7.412333057	7.006006406	7.818659708
2060	-0.352108471	-0.352108	-0.352108471	6.165870606	4.952524001	7.379217211
2065	7.695546702	6.9511513	7.323349013	1.825329981	2.641494844	1.009165119
2068	-0.352108471	-0.352108	-0.352108471	8.299332542	6.51496239	4.730592239
2069	5.08289799	3.0818222	4.08236011	6.44500631	4.240633614	5.342819962
2070	-0.352108471	-0.352108	-0.352108471	4.80208491	4.671268472	4.932901348
2071	-0.352108471	-0.352108	-0.352108471	4.178899946	4.551431141	3.80636875
2072	7.210568768	6.9786128	7.442524773	2.585128717	3.119148782	2.051108653
2077	-0.352108471	-0.352108	-0.352108471	7.747692536	5.494417177	3.241141818
2079	3.127737875	3.136327	3.119148782	7.83170519	7.85000045	7.813409929
2091	-0.352108471	-0.352108	-0.352108471	6.345275458	6.701057834	5.989493081
2098	6.598403711	7.185766	7.773128285	5.983736712	4.955631715	7.011841709

2099	-0.352108471	-0.352108	-0.352108471	6.330004048	6.276856601	6.383151496
2101	8.61424024	5.3651483	6.989694288	2.20636753	2.211943219	2.217518907
2107	5.456140284	7.3814837	3.530796835	7.046260879	6.147254752	6.596757815
2108	-0.352108471	-0.352108	-0.352108471	5.663737322	5.207152599	6.120322045
2109	-0.352108471	-0.352108	-0.352108471	4.79472922	4.153789937	5.435668504
2113	5.570057478	4.6641742	5.117115863	6.258034115	6.889903884	6.573968999
2114	7.692181027	6.5373837	5.382586422	-0.352108471	-0.352108471	-0.352108471
2115	5.217491139	4.9287584	5.506223914	5.894225185	5.458255064	5.022284943
2117	-0.352108471	-0.352108	-0.352108471	5.416220218	0.482407625	2.949313922
2120	-0.352108471	-0.352108	-0.352108471	5.234766675	6.038453511	4.43107984
2121	-0.352108471	-0.352108	-0.352108471	7.178903471	6.81196605	6.44502863
2122	6.5002282	3.9037242	9.096732239	7.400897894	5.627005534	3.853113173
2125	6.242048279	8.0947746	4.389321912	-0.352108471	-0.352108471	-0.352108471
2130	-0.352108471	-0.352108	-0.352108471	8.424196	8.280175894	8.136155787
2131	8.759352771	6.8743147	7.816833734	8.500231745	8.511761357	8.488702132
2132	-0.352108471	-0.352108	-0.352108471	6.923295051	6.946414218	6.900175883
2135	-0.352108471	-0.352108	-0.352108471	6.515221104	5.528880746	6.022050925
2137	-0.352108471	-0.352108	-0.352108471	5.375906715	6.165870606	6.955834497
2139	-0.352108471	-0.352108	-0.352108471	2.080138319	4.898975698	7.717813077
2149	7.673425716	7.7658364	7.581015059	7.535088133	7.481351049	7.508219591
2151	-0.352108471	-0.352108	-0.352108471	2.19005738	2.232575625	2.147539136
2152	10.05837813	6.3905161	8.224447097	10.65873184	7.033152281	8.845942058
2153	6.942665805	4.8131929	9.072138752	8.541410162	4.727980486	6.634695324
2159	3.308991012	4.3023377	3.805664353	6.565372851	6.085247509	6.32531018
2180	-0.352108471	-0.352108	-0.352108471	3.306254385	4.456886199	5.607518014
2182	-0.352108471	-0.352108	-0.352108471	5.529311366	7.256880981	3.80174175
2185	-0.352108471	-0.352108	-0.352108471	2.322627657	2.124237496	1.925847335
2186	6.041233575	7.3777551	8.714276692	-0.352108471	-0.352108471	-0.352108471
2188	6.286751578	6.5546628	7.822574067	4.557244691	3.614411483	3.500077898
2189	2.65059567	4.5440465	0.757144822	-0.352108471	-0.352108471	-0.352108471
2191	6.551606154	5.5587679	4.565929586	6.783875524	9.099356912	7.941616218
2192	9.371543471	6.5625652	7.967054352	8.368838247	7.454049592	7.91144392
2193	2.046297133	10.618636	6.332466669	-0.352108471	-0.352108471	-0.352108471
2194	2.379705706	7.8440457	5.111875725	7.684929473	8.376100243	6.993758704
2195	6.763594968	7.3603202	7.957045342	6.418164666	5.140197889	7.696131453
2196	6.30803701	6.502263	6.696489047	-0.352108471	-0.352108471	-0.352108471
2197	6.433318728	5.8377149	7.028922543	6.981888777	7.003831781	6.959945773
2199	6.659204891	6.8637753	6.454634494	7.324498278	4.166290649	5.745394463
2200	6.463674915	6.479333	6.448016836	2.076022254	2.100935855	2.051108653
2201	4.474008088	7.2318668	5.852937462	5.746917231	6.998915179	4.494919283
2203	4.091800068	5.2460267	4.668913391	-0.352108471	-0.352108471	-0.352108471
2204	7.634347441	8.2897006	6.978994273	4.618894849	6.754558526	8.890222202
2206	6.210579573	3.9271055	5.06884253	3.832339467	2.100935855	5.563743079
2207	-0.352108471	-0.352108	-0.352108471	2.169424975	1.625447907	2.713402042
2214	6.696903716	6.6741289	6.651354042	-0.352108471	-0.352108471	-0.352108471
2217	7.458282885	8.5020748	6.414490975	6.804723141	5.271355789	8.338090493
2219	7.017628004	4.8607564	5.939192221	-0.352108471	-0.352108471	-0.352108471

2222	3.683980801	5.1693991	2.198562478	7.841286877	8.173357876	8.007322377
2224	7.016776417	6.0761857	7.957367182	1.146563148	1.783698732	2.420834317
2230	-0.352108471	-0.352108	-0.352108471	1.153691946	2.902991023	4.652290099
2232	-0.352108471	-0.352108	-0.352108471	5.821834654	6.766850887	6.294342771
2235	-0.352108471	-0.352108	-0.352108471	2.724072976	3.677812865	4.631552755
2237	3.726842123	1.8410745	2.783958294	-0.352108471	-0.352108471	-0.352108471
2242	-0.352108471	-0.352108	-0.352108471	5.958359385	7.333941542	4.582777227
2244	5.765810019	4.3067107	7.224909308	0.808855576	0.808855576	0.808855576
2245	-0.352108471	-0.352108	-0.352108471	7.549492193	5.021616724	6.285554459
2247	1.392109249	3.136327	-0.352108471	9.6558732	4.763084469	7.209478834
2250	9.597687225	12.671049	11.13436836	9.730301708	9.158004197	10.30259922
2252	3.672022488	7.4455133	5.55876787	5.567737755	6.476364095	6.022050925
2254	-0.352108471	-0.352108	-0.352108471	5.400974508	5.760537073	6.120099638
2255	-0.352108471	-0.352108	-0.352108471	0.95025487	2.252618212	-0.352108471
2260	4.699855738	3.6761388	5.723572646	7.556478335	7.578336491	7.534620179
2271	6.888413829	7.8452378	5.931589838	5.760537073	9.835666209	1.685407937
2273	6.369156591	5.7524106	6.985902626	4.398556261	11.85187139	8.125213827
2274	6.640019393	6.2886778	6.991360979	8.160328149	8.121597162	8.082866175
2280	4.689234271	8.8198828	6.754558526	7.728772973	6.886610445	6.044447917
2282	7.1250675	7.7328126	6.517322371	8.150840899	7.136426663	6.122012426
2286	3.349849757	9.3150836	6.332466669	9.248218001	7.082757193	4.917296386
2287	7.563139502	6.3968684	5.230597263	2.169424975	2.191310813	2.147539136
2288	7.639654029	3.722998	5.681326035	-0.352108471	-0.352108471	-0.352108471
2289	-0.352108471	-0.352108	-0.352108471	3.354121925	3.585121301	3.816120677
2297	-0.352108471	-0.352108	-0.352108471	6.840114058	5.458724769	8.221503348
2302	-0.352108471	-0.352108	-0.352108471	6.369156591	10.35472451	2.38358867
2305	-0.352108471	-0.352108	-0.352108471	5.96988645	6.938916709	6.454401579
2309	6.104429468	8.4779124	3.730946491	6.866100805	7.427300611	6.304901
2310	5.84844837	6.1122684	6.376088439	1.117721484	1.305230684	0.930212283
2314	7.641521483	7.6935991	7.745676625	9.15598567	7.598869975	6.041754279
2315	6.673731665	6.0968157	6.385273697	2.449166203	2.902991023	3.356815842
2316	11.55446531	10.512475	9.470484251	10.36630957	11.82947108	13.29263259
2317	-0.525004821	5.4275862	2.451290702	5.688622647	4.187591105	7.189654189
2318	9.982663572	8.5575689	7.13247418	9.532966436	7.74575082	8.639358628
2319	9.035948539	6.1348006	3.233652691	6.261742918	5.528542175	6.99494366
2321	-0.352108471	-0.352108	-0.352108471	1.874819493	3.15657568	2.515697587
2323	-0.352108471	-0.352108	-0.352108471	6.935013718	6.165870606	5.396727494
2332	-0.352108471	-0.352108	-0.352108471	6.235921161	6.505633794	5.966208528
2335	8.181629182	8.6650457	7.698212656	9.102693505	9.102693505	9.102693505
2339	-0.352108471	-0.352108	-0.352108471	6.32531018	6.33538923	6.31523113
2350	-0.352108471	-0.352108	-0.352108471	5.584676343	7.410292288	3.759060398
2353	5.85030116	5.5587679	5.26723458	6.345275458	7.618072573	5.072478342
2354	6.609225976	0.4210565	3.515141236	4.920682473	6.846853879	8.773025285
2355	-0.352108471	-0.352108	-0.352108471	9.417033112	6.27294594	7.844989526
2356	3.515141236	4.1520868	2.8781957	7.146302205	6.28095301	8.011651399
2395	8.205860956	8.3595966	8.513332228	7.25414833	7.922309521	7.588228926
2475	4.125607521	6.0532169	5.089412202	3.590870952	2.647426611	3.119148782

2483	7.61679815	6.832171	7.224484585	0.044132154	0.440372779	-0.352108471
2487	10.08538839	2.8419614	6.463674915	-0.352108471	-0.352108471	-0.352108471
2495	11.22548609	13.06983	12.14765807	12.17189408	12.44248541	11.90130275
2512	12.36698484	12.284928	12.2028719	12.21333291	13.78504098	12.99918694
2517	-0.352108471	-0.352108	-0.352108471	5.745394463	4.331476087	7.159312839
2521	10.16942051	9.1222681	8.075115628	9.002076863	8.243372387	7.48466791
2523	8.053495589	8.6475911	7.459400044	5.395888616	5.955808726	5.675848671
2524	7.123881271	7.4351711	7.746460916	3.730614766	7.51811256	5.624363663
2526	2.740477604	4.7885798	6.836681921	3.606283772	6.635383637	0.577183907
2531	7.373857007	7.2622793	7.485434673	5.297807945	5.529311366	5.066304524
2535	5.092988638	6.0583172	7.023645731	5.86687125	8.705778044	7.286324647
2538	4.00769727	4.0872577	4.166818059	2.732213788	1.456674477	2.094444132
2539	6.590838154	4.6643799	8.517296439	5.551927689	7.040160042	6.296043865
2547	6.022396261	7.0634138	8.104431351	8.285404702	7.840701215	8.063052958
2551	-0.352108471	-0.352108	-0.352108471	3.119148782	3.119148782	3.119148782
2553	-0.352108471	-0.352108	-0.352108471	5.095423988	5.124543452	5.066304524
2564	4.768486756	3.633531	5.903442506	-0.352108471	-0.352108471	-0.352108471
2582	6.960683454	5.646239	6.303461245	8.820355491	6.49796827	7.659161881
2589	-0.352108471	-0.352108	-0.352108471	5.169157826	5.065627102	5.272688549
2590	-0.352108471	-0.352108	-0.352108471	7.125013876	7.359723467	6.890304284
2591	6.332466669	3.5505276	9.114405751	-0.352108471	-0.352108471	-0.352108471
2592	6.183964016	6.1999612	6.167966808	-0.352108471	-0.352108471	-0.352108471
2593	-0.352108471	-0.352108	-0.352108471	2.686833264	2.686833264	2.686833264
2596	5.444884047	6.7357797	8.026675325	-0.352108471	-0.352108471	-0.352108471
2597	11.2013696	13.248453	9.154286333	10.20560039	13.70840565	11.95700302
2617	6.151585628	6.555673	5.747498225	6.200625599	7.679737185	9.158848772
2618	-0.352108471	-0.352108	-0.352108471	3.919505936	3.919505936	3.919505936
2619	-0.352108471	-0.352108	-0.352108471	6.285554459	8.635408942	3.935699975
2621	8.686519674	8.7166969	8.656342398	7.556606428	7.584456235	7.528756621
2624	7.650812904	6.562628	8.738997828	11.02495564	3.326127054	7.175541346
2629	-0.352108471	-0.352108	-0.352108471	3.993166125	4.415028907	4.204097516
2632	-0.352108471	-0.352108	-0.352108471	6.923170513	4.580410295	9.26593073
2633	6.17278307	7.0419718	6.607377459	6.463674915	8.52096852	4.406381309
2634	-0.352108471	-0.352108	-0.352108471	7.904564082	5.922413715	6.913488898
2635	6.573968999	5.1119583	8.035979731	-0.352108471	-0.352108471	-0.352108471
2647	-0.352108471	-0.352108	-0.352108471	5.555939844	6.089618607	5.022261081
2648	7.186619416	6.8886555	7.484583343	3.919505936	5.436777163	2.402234708
2649	8.108921488	7.7121014	8.505741605	5.145086344	5.529311366	4.760861323
2650	-0.352108471	-0.352108	-0.352108471	5.260797528	6.056053999	6.85131047
2664	7.876988749	9.2138527	6.540124817	7.101935164	5.856585589	6.479260377
2665	6.258419638	6.5373837	6.816347811	5.839721471	5.60724956	5.723485516
2673	8.138146831	10.200446	6.075848034	6.285577398	7.588326356	8.891075314
2674	-0.352108471	-0.352108	-0.352108471	2.469725738	2.686833264	2.252618212
2675	-0.352108471	-0.352108	-0.352108471	5.016362163	5.017910708	5.017136436
2677	6.146695615	7.1310468	6.638871208	1.761670631	1.646479888	1.876861374
2683	7.9572782	8.166821	8.062049619	9.070767821	6.47267644	3.874585059
2690	5.816094076	6.0565695	6.297044835	-0.352108471	-0.352108471	-0.352108471

2697	6.89992346	6.9268019	6.873045007	8.678484689	8.692723556	8.664245823
2700	-0.352108471	-0.352108	-0.352108471	6.484812654	5.845499835	5.206187016
2702	6.537383725	6.3957135	6.679053947	4.804144212	7.112574557	5.958359385
2706	5.800199788	5.8503502	5.750049386	-0.352108471	-0.352108471	-0.352108471
2720	5.999964988	9.3249334	7.662449217	-0.352108471	-0.352108471	-0.352108471
2730	6.093440296	7.1611846	8.228929001	5.132034894	7.080858494	6.106446694
2734	5.569701552	7.0843391	8.59897666	9.555754306	8.076342711	6.596931117
2735	-0.352108471	-0.352108	-0.352108471	3.919505936	3.919505936	3.919505936
2738	-0.352108471	-0.352108	-0.352108471	5.704335255	4.731640353	6.677030157
2739	5.852937462	6.8492035	4.856671472	11.13010065	4.867725119	7.998912883
2740	4.408656986	7.8344006	6.121528769	-0.352108471	-0.352108471	-0.352108471
2745	9.77971259	9.874147	9.685278138	9.944857578	9.944857578	9.944857578
2746	5.688622647	7.9211495	3.45609575	6.26481039	5.637296679	5.009782968
2749	-0.352108471	-0.352108	-0.352108471	8.026190996	4.476933655	6.251562326
2752	7.606017131	7.9352485	8.264479906	9.150898656	8.077831649	7.004764642
2760	7.92219406	6.6552114	7.288702744	6.146432796	6.1418345	6.151031091
2762	-0.352108471	-0.352108	-0.352108471	7.269863612	6.203704376	8.336022848
2764	-0.352108471	-0.352108	-0.352108471	7.559538207	7.74933204	7.369744374
2767	6.926801914	4.7635308	9.090073008	6.997455924	5.840530024	8.154381824
2768	5.054293344	8.5504874	6.802390362	9.681322766	8.061481385	6.441640003
2770	-0.352108471	-0.352108	-0.352108471	7.145187302	7.532202005	7.338694653
2771	8.707510774	6.5293011	10.8857204	8.799502368	9.294226052	9.04686421
2773	3.966480855	3.5557829	3.761131874	6.785944301	6.757762328	6.814126275
2775	4.01071017	5.4283939	6.846077578	-0.352108471	-0.352108471	-0.352108471
2776	2.811927891	5.6785007	4.245214285	7.428834872	4.650467954	6.039651413
2778	10.29884091	8.9488622	9.623851561	8.671182321	10.21444565	11.75770899
2782	7.481209945	8.1296288	7.805419383	8.839674519	8.372682046	9.306666991
2783	6.303794181	7.0871828	7.870571479	8.878326382	10.03241983	7.724232938
2786	6.635116535	8.4701868	7.552651643	5.747617149	4.544763662	6.950470636
2787	8.062049619	6.6193371	9.504762185	8.539184559	9.546437387	7.531931731
2791	6.440388338	-0.184913	3.127737875	7.983511276	5.235574771	6.609543024
2794	7.424012079	6.9916642	7.207838162	0.235164116	8.122635775	4.178899946
2799	7.336298251	7.4498036	7.39305092	8.222041546	7.112594586	6.003147626
2800	-0.352108471	-0.352108	-0.352108471	7.555631787	4.63696661	6.096299198
2801	8.940128237	6.8355031	7.887815677	3.847881823	7.156915368	5.502398596
2802	7.359058494	8.2892025	6.428914528	6.38341389	7.439230644	8.495047398
2803	6.242048279	4.156177	8.327919596	6.576200407	6.217536358	6.396868383
2804	-0.352108471	-0.352108	-0.352108471	6.243205572	6.348061445	6.295633508
2805	6.020010435	4.2844838	5.152247113	-1.076607949	1.223721097	3.524050143
2806	4.028701554	7.571698	5.800199788	5.133550664	7.203822097	9.27409353
2814	5.939192221	6.5659524	5.312432092	-0.352108471	-0.352108471	-0.352108471
2817	8.04233763	6.5303117	7.286324647	8.459742919	9.041130421	9.622517923
2820	-0.352108471	-0.352108	-0.352108471	5.939192221	4.798732669	7.079651774
2821	5.502720352	7.5720471	6.537383725	8.157650828	8.886414932	7.428886724
2822	6.67196754	5.1668445	8.177090578	5.850738532	6.150437391	6.000587961
2823	6.273832259	6.2858739	6.261790604	-0.352108471	-0.352108471	-0.352108471
2825	5.414783571	5.2365191	5.325651342	5.959506607	4.826052208	7.092961005

2835	7.188487283	4.3453722	5.766929757	0.95025487	2.252618212	-0.352108471
2846	6.674128879	6.6898785	6.658379305	-0.352108471	-0.352108471	-0.352108471
2848	6.239860938	5.5371901	6.942531774	-0.352108471	-0.352108471	-0.352108471
2849	6.881031277	5.6886226	4.496214017	-0.352108471	-0.352108471	-0.352108471
2852	-0.352108471	-0.352108	-0.352108471	3.503248387	6.835067264	5.169157826
2856	3.644963209	5.4283939	7.211824539	-0.352108471	-0.352108471	-0.352108471
2859	2.993849458	5.0522677	7.110685912	-0.352108471	-0.352108471	-0.352108471
2861	-0.352108471	-0.352108	-0.352108471	6.415928975	6.986047344	5.845810605
2869	2.816880127	8.1706863	5.493783215	-0.352108471	-0.352108471	-0.352108471
2870	-0.352108471	-0.352108	-0.352108471	2.169424975	-0.810084366	5.148934315
2872	3.002133479	3.4883371	3.245235305	7.449423849	7.574730164	7.700036479
2873	5.493783215	2.6029821	8.384584344	7.120446052	6.986345374	7.254546729
2874	6.402969886	5.1974297	5.800199788	7.744942324	6.852201546	7.298571935
2876	8.198897972	7.4106255	8.987170458	6.512966919	8.793387128	11.07380734
2879	8.885127417	7.8618358	6.838544105	5.032240293	6.505960865	7.979681437
2882	5.184151491	5.2437595	5.124543452	-0.352108471	-0.352108471	-0.352108471
2885	3.799678963	4.2452143	3.354143641	8.906152791	8.49912695	8.092101108
2890	5.152247113	5.6697357	4.634758505	-0.352108471	-0.352108471	-0.352108471
2893	-0.352108471	-0.352108	-0.352108471	4.559699198	5.320328988	3.799069408
2896	8.660133915	5.6153209	7.137727412	10.90964728	7.956236881	5.002826481
2897	6.03798158	6.0736545	6.002308672	4.522024694	5.124543452	3.919505936
2900	-0.352108471	-0.352108	-0.352108471	2.159015127	4.773032422	3.466023774
2906	7.648141331	6.1286863	6.888413829	-0.352108471	-0.352108471	-0.352108471
2907	6.816905042	6.8453896	6.788420481	8.822644798	8.848465207	8.79682439
2908	-0.352108471	-0.352108	-0.352108471	5.450191304	6.758667632	6.104429468
2912	-0.352108471	-0.352108	-0.352108471	3.919505936	4.61154998	3.227461892
2914	4.722713869	6.0203811	7.318048314	-0.352108471	-0.352108471	-0.352108471
2916	6.488181269	6.435419	6.540943519	3.918072844	3.827651704	3.737230565
2918	-0.352108471	-0.352108	-0.352108471	5.203525788	3.128869024	7.278182552
2920	5.401039968	5.3910182	5.380996361	0.044132154	0.440372779	-0.352108471
2923	-0.352108471	-0.352108	-0.352108471	8.640359129	9.661681892	7.619036366
2926	-0.352108471	-0.352108	-0.352108471	6.51496239	6.532588736	6.497336045
2934	7.037798976	7.065024	7.010573985	6.963991695	6.978612763	6.949370627
2935	8.799373749	3.5037975	6.151585628	6.636273252	4.155841674	9.116704829
2936	3.515141236	3.7437463	3.28653617	5.239507923	6.677210847	5.958359385
2937	5.761334869	7.3866031	6.573968999	9.083823607	6.846853879	4.609884152
2940	-0.352108471	-0.352108	-0.352108471	2.53473567	5.421579811	-0.352108471
2941	-0.352108471	-0.352108	-0.352108471	5.226519665	5.169157826	5.111795986
2947	6.874586128	5.5221145	8.227057708	6.86293602	6.923579896	6.802292144
2950	6.504458524	9.1449872	7.82472285	9.046552185	8.388315214	8.7174337
2954	-0.352108471	-0.352108	-0.352108471	4.277435984	5.169157826	6.060879667
2957	-0.352108471	-0.352108	-0.352108471	5.369332089	5.383503645	5.355160532
2958	2.245600013	1.4200355	3.071164504	-0.352108471	-0.352108471	-0.352108471
2960	7.629150694	8.4071532	8.018151954	5.92702014	5.980385646	6.033751153
2962	-0.352108471	-0.352108	-0.352108471	7.137751962	5.704580295	8.570923628
2963	7.554787271	6.3802352	6.967511245	6.17571908	6.889164886	6.532441983
2967	6.740863465	6.8081556	6.77450951	5.902534088	7.681646284	4.123421891

2968	8.180299067	4.0627585	6.121528769	-0.352108471	-0.352108471	-0.352108471
2975	6.559509134	6.1054242	6.332466669	2.169424975	2.189845889	2.149004061
2976	7.086251434	6.4142581	5.742264837	-0.352108471	-0.352108471	-0.352108471
2977	1.860062114	6.0384535	10.21684491	-0.352108471	-0.352108471	-0.352108471
2979	6.704982178	6.3950667	7.014897631	4.468360629	4.023270159	3.578179689
2982	4.570230597	5.9194595	7.26868849	-0.352108471	-0.352108471	-0.352108471
2987	3.127737875	4.0340787	2.221397017	2.970676224	5.855547981	8.740419737
2992	-0.352108471	-0.352108	-0.352108471	7.990281747	6.325133473	7.15770761
2994	-0.352108471	-0.352108	-0.352108471	3.179776966	4.482692559	1.876861374
2995	-0.352108471	-0.352108	-0.352108471	6.253719412	7.960325437	7.107022425
2997	6.212536209	3.5856415	8.839430905	9.121602218	5.169157826	1.216713433
3000	-0.352108471	-0.352108	-0.352108471	1.167362396	2.686833264	-0.352108471
3005	-0.352108471	-0.352108	-0.352108471	7.643140836	9.323743688	5.962537983
3009	-0.352108471	-0.352108	-0.352108471	6.66034956	6.533166071	6.596757815
3014	5.872206726	6.8107911	6.341498931	6.434828619	6.975186513	7.515544407
3015	7.04152946	4.9992327	6.020381091	7.329808415	7.755210593	8.18061277
3021	6.450302837	6.479333	6.421272681	8.964795166	8.966783408	8.962806924
3026	5.777690558	6.5194696	5.035911528	-0.352108471	-0.352108471	-0.352108471
3028	-0.352108471	-0.352108	-0.352108471	6.081561369	5.665786693	6.497336045
3030	7.0532029	7.5583968	8.063590761	6.713118177	6.099448998	6.406283588
3032	-0.352108471	-0.352108	-0.352108471	6.069050974	6.056053999	6.043057024
3035	7.398193617	5.1562159	9.640171295	7.988337951	11.20456133	4.772114574
3046	-0.352108471	-0.352108	-0.352108471	-0.162668421	4.456886199	9.07644082
3052	-0.352108471	-0.352108	-0.352108471	0.762376451	-0.352108471	1.876861374
3054	3.131295231	3.5151412	3.898987241	6.964397514	6.822208827	6.680020139
3059	-0.352108471	-0.352108	-0.352108471	1.601336827	-0.352108471	3.554782125
3064	4.191276601	8.7513476	6.471312114	5.468559522	6.588254064	7.707948605
3065	3.769410859	2.5886014	1.407792036	5.637296679	7.178359369	4.096233989
3066	6.824063418	6.925707	6.874885195	6.933400364	7.830042649	8.726684934
3068	6.518241051	6.5884653	6.448016836	8.69099188	8.713854436	8.668129324
3069	9.55986917	9.8950497	9.727459417	9.355101689	10.00489536	9.679998524
3070	3.397489732	5.8529375	8.308385191	-0.352108471	-0.352108471	-0.352108471
3071	5.085628549	6.5527505	8.019872406	-0.352108471	-0.352108471	-0.352108471
3074	4.432439699	3.9253602	4.178899946	6.316062741	6.611287089	6.463674915
3081	3.230907932	7.4784634	5.354685649	-0.352108471	-0.352108471	-0.352108471
3090	5.354685649	5.0792369	5.630134363	5.723485516	4.098167687	7.348803344
3091	-0.352108471	-0.352108	-0.352108471	6.56915909	6.588465267	6.549852913
3092	8.263002114	10.233446	6.292558225	7.466249602	8.144006598	6.788492606
3093	5.980334028	6.7848199	5.175848156	2.000013094	1.539678786	1.76984594
3094	5.248304154	4.7370778	5.759530534	6.860600843	7.885729184	5.835472501
3096	5.089412202	5.1245435	5.054280952	-0.352108471	-0.352108471	-0.352108471
3097	5.428393874	5.3044408	5.552346937	5.821410838	3.939369404	7.703452271
3098	5.939192221	6.6138618	5.264522679	7.519935728	7.650335294	7.78073486
3099	-0.352108471	-0.352108	-0.352108471	5.095423988	1.012541458	9.178306518
3105	10.18815237	9.0930446	7.997936849	6.566707377	8.252720256	9.938733134
3106	8.409350362	8.1658357	8.287593055	8.406349062	9.56624669	8.986297876
3107	7.138699673	11.417092	9.2778957	9.198296144	9.076660162	9.319932127

3110	4.836905519	5.1973674	5.017136436	2.024343053	2.051108653	1.997577452
3133	6.733587763	6.9848468	6.859217306	8.469855985	6.867407982	7.668631984
3139	6.565700169	4.6396027	8.491797642	6.01736765	7.991002022	4.043733279
3140	3.618320908	6.2298631	8.841405283	8.030651509	5.687783102	6.859217306
3142	7.148954265	4.7610343	9.536874197	0.939116071	0.440372779	1.437859363
3145	-0.352108471	-0.352108	-0.352108471	6.621237034	5.596916991	4.572596948
3146	7.820604505	7.9270228	7.714186196	8.833787387	8.877971188	8.789603586
3148	-0.352108471	-0.352108	-0.352108471	4.707367729	4.80208491	4.896802091
3149	7.137727412	7.0059725	7.269482318	2.38621749	5.124543452	-0.352108471
3150	-0.352108471	-0.352108	-0.352108471	8.618240121	7.682800044	6.747359966
3151	7.169692427	7.4699623	6.869422526	10.14601793	9.25501272	9.700515327
3155	-0.352108471	-0.352108	-0.352108471	2.039485684	-0.352108471	4.43107984
3159	11.67670098	7.2621473	9.469424158	8.737015381	9.760419768	10.78382416
3162	-0.352108471	-0.352108	-0.352108471	6.715300913	5.903760504	5.092220095
3163	-0.352108471	-0.352108	-0.352108471	6.694172847	9.35003797	4.038307724
3164	7.858118037	6.9747915	8.741444619	8.19498699	5.428920818	6.811953904
3166	6.934539402	7.7334712	7.334005289	-0.352108471	-0.352108471	-0.352108471
3172	6.189072191	7.6640454	6.926558783	-0.352108471	-0.352108471	-0.352108471
3177	-0.352108471	-0.352108	-0.352108471	2.113278123	5.725733748	3.919505936
3178	6.04989125	4.7874689	7.312313562	8.080096592	7.3967979	6.713499208
3181	8.299575662	7.4549104	9.144240941	9.321384939	6.376298356	7.848841648
3182	5.723896253	6.3324667	6.941037084	7.510209198	7.289109828	7.068010458
3183	4.151787417	5.386757	6.621726484	7.46337882	6.312126346	8.614631294
3184	6.167582836	6.3999574	5.935208281	7.19609887	8.000813021	6.39138472
3185	-0.352108471	-0.352108	-0.352108471	6.488107881	6.515031054	6.461184708
3187	-0.352108471	-0.352108	-0.352108471	7.096295931	6.767576823	6.931936377
3189	4.966788347	6.2570463	7.547304338	7.124132745	9.118283565	5.129981925
3190	8.42339261	7.814447	8.118919828	9.433720827	8.121209203	8.777465015
3191	3.245235305	3.136327	3.354143641	8.193053453	8.207404259	8.178702646
3192	6.369156591	7.5066108	5.231702339	7.016084732	8.308388617	5.723780847
3207	10.9103478	7.7509518	4.591555851	6.32531018	7.173685907	5.476934454
3213	-0.352108471	-0.352108	-0.352108471	6.039651413	5.842349393	6.236953434
3219	7.047306935	4.8750268	5.961166865	-0.352108471	-0.352108471	-0.352108471
3235	-0.352108471	-0.352108	-0.352108471	6.926938623	5.355431001	8.498446244
3237	5.939192221	5.9583594	5.920025058	7.082757193	7.100490419	7.065023967
3241	-0.352108471	-0.352108	-0.352108471	7.990538841	6.463674915	4.936810989
3251	-0.352108471	-0.352108	-0.352108471	6.524802878	6.40631083	6.287818783
3257	6.319191314	5.840966	5.362740716	-0.352108471	-0.352108471	-0.352108471
3265	1.911668176	4.8786655	7.845662781	-0.352108471	-0.352108471	-0.352108471
3267	-0.352108471	-0.352108	-0.352108471	2.024343053	2.051108653	1.997577452
3269	6.133336141	5.489617	6.77705526	0.044132154	-0.352108471	0.440372779
3275	6.332466669	6.3439771	6.320956231	6.997455924	7.016299086	6.978612763
3276	6.53740366	8.6768585	7.607131056	9.928760285	8.242258058	6.555755831
3281	7.02332832	5.8749598	8.171696872	6.550537182	5.561570816	6.056053999
3284	7.052990603	7.1550161	6.950965134	-0.352108471	-0.352108471	-0.352108471
3292	2.681724436	5.7155573	-0.352108471	-0.352108471	-0.352108471	-0.352108471
3297	4.14713782	7.4532618	5.800199788	5.016504084	6.94160582	8.866707556

3300	6.862147155	5.7447753	6.303461245	4.780711618	4.539403762	4.66005769
3301	5.524840227	4.5094326	5.017136436	7.284043907	6.64213435	6.000224793
3304	4.447190182	4.351251	4.543129357	0.395051519	1.14221151	-0.352108471
3306	-0.352108471	-0.352108	-0.352108471	5.858942671	7.107022425	8.355102178
3308	8.2833161	5.4387989	6.861057494	8.062353202	8.234604437	7.890101968
3309	8.706352031	5.6716473	11.74105675	9.865498692	9.083899944	8.302301195
3312	11.72664061	9.387224	7.047807296	10.53344541	10.34988851	10.16633162
3313	8.683688676	6.8018138	4.919938871	7.76697743	6.694172847	5.621368264
3315	11.60415388	6.6105304	9.107342156	10.28284962	11.5424811	9.023218138
3316	4.578859472	5.4361909	6.293522372	-0.352108471	-0.352108471	-0.352108471
3320	10.15391215	7.8078346	8.980873376	7.382056394	11.02742677	9.204741582
3326	10.29613331	8.8964382	7.496743117	10.37436414	9.158476009	7.942587883
3329	8.028411128	7.1073931	8.949429178	8.090886677	6.469218275	9.712555079
3337	5.180894808	5.4937832	5.806671621	1.223721097	0.85959438	1.587847814
3338	-0.352108471	-0.352108	-0.352108471	5.613451577	5.105495698	6.121407457
3339	8.873924453	8.9717102	8.92281734	6.243235173	6.038453511	6.448016836
3340	-0.352108471	-0.352108	-0.352108471	8.159795512	8.426283728	8.29303962
3351	-0.352108471	-0.352108	-0.352108471	5.284974356	4.959233174	5.610715538
3358	-0.352108471	-0.352108	-0.352108471	4.896613476	5.017136436	5.137659395
3364	4.522028939	6.3780695	8.234110053	3.590023696	3.250862253	3.420442975
3371	-0.352108471	-0.352108	-0.352108471	6.056053999	4.912659641	7.199448357
3375	5.11101762	7.3333471	9.555676624	6.096590115	5.457998494	4.819406872
3376	4.647646069	5.5913056	6.534965108	-0.352108471	-0.352108471	-0.352108471
3383	7.263919002	5.6366867	6.450302837	7.287375453	8.053131241	6.521619664
3386	-0.352108471	-0.352108	-0.352108471	1.783698732	-0.352108471	3.919505936
3397	8.304467859	4.4892689	6.396868383	7.646004538	8.703955454	6.588053622
3398	-0.352108471	-0.352108	-0.352108471	3.919505936	5.733955955	2.105055916
3399	-0.352108471	-0.352108	-0.352108471	8.318625916	9.196310188	7.440941643
3400	1.957606802	5.8288485	9.700090126	-0.352108471	-0.352108471	-0.352108471
3416	6.462091294	7.0978125	7.733533766	1.689109503	1.731739118	1.646479888
3417	-0.352108471	-0.352108	-0.352108471	4.727148895	5.095423988	5.46369908
3419	5.625160771	6.5311342	4.719187355	6.868554262	3.16571861	5.017136436
3420	-0.352108471	-0.352108	-0.352108471	6.18047806	5.823140203	6.001809131
3421	4.727729798	4.9735278	5.219325861	0.647185709	-0.352108471	1.646479888
3423	4.259723664	3.6761388	4.843308497	5.20089139	5.254689875	5.147092904
3428	-0.352108471	-0.352108	-0.352108471	6.668125644	7.478462511	8.288799378
3431	-0.352108471	-0.352108	-0.352108471	3.947987959	4.016045771	4.084103583
3433	-0.352108471	-0.352108	-0.352108471	7.773518037	5.256406744	6.51496239
3434	6.020656397	5.873346	6.167966808	6.887680082	7.016299086	6.759061079
3437	6.522675414	6.1999612	6.845389604	7.377124155	7.49196897	7.26227934
3454	6.033774876	6.7943232	5.273226589	0.975533543	0.808855576	1.14221151
3455	6.054330535	3.0800634	4.567196948	8.408296827	3.613362653	6.01082974
3459	6.627660242	5.6641722	4.700684121	-0.352108471	-0.352108471	-0.352108471
3460	-0.352108471	-0.352108	-0.352108471	3.235346083	6.539179568	4.887262826
3476	5.980334028	4.8002135	7.160454534	4.403284405	7.315950333	5.859617369
3479	6.826861542	5.8876701	7.766052954	6.761447227	6.368679046	7.154215408
3480	1.383520155	1.9527896	0.814250726	7.246609587	6.549852913	5.853096238

3481	10.38143988	7.2640884	8.822764129	7.242521962	5.834633147	8.650410777
3482	4.878665478	5.7328621	4.024468896	-0.352108471	-0.352108471	-0.352108471
3486	6.093634638	7.4538469	6.77374078	7.696084753	7.17040874	7.433246747
3487	9.769125527	9.7691255	9.769125527	9.974617326	9.979895128	9.969339523
3488	11.37003559	11.393313	11.34675789	11.4703594	11.4703594	11.4703594
3489	4.538693134	6.0203811	7.502069049	9.961621349	6.128501857	8.045061603
3490	5.791964116	7.3053415	8.818718842	7.873692252	11.02837773	9.451034992
3491	7.352453156	6.7568561	7.948050163	8.255214198	10.74341222	9.499313207
3508	2.168105043	4.6883186	-0.352108471	-0.352108471	-0.352108471	-0.352108471
3516	6.965310702	6.9129933	6.860675988	6.038132331	5.50822504	6.568039622
3543	-0.352108471	-0.352108	-0.352108471	6.333081941	6.165870606	5.998659271
3550	6.573968999	6.5884653	6.559472732	7.286324647	7.29520141	7.277447884
3559	-0.352108471	-0.352108	-0.352108471	4.338147062	4.254913518	4.421380607
3563	5.788942239	7.0776952	6.433318728	-0.352108471	-0.352108471	-0.352108471
3566	6.732320039	7.9356905	7.334005289	5.325716078	4.81834049	5.833091666
3569	-0.352108471	-0.352108	-0.352108471	5.412561087	5.421579811	5.403542364
3570	7.149289854	4.2141661	5.681727979	2.139844176	-0.352108471	4.631796822
3572	4.467791151	3.8632301	5.07235221	-0.352108471	-0.352108471	-0.352108471
3576	6.77374078	6.7884205	6.759061079	6.12990004	6.135204448	6.124595632
3579	5.406977617	6.4762162	5.941596889	4.338147062	4.245214285	4.43107984
3581	-0.352108471	-0.352108	-0.352108471	1.359428583	2.686833264	4.014237944
3586	7.987762161	6.6333311	5.278900124	3.557621381	5.383503645	1.731739118
3588	3.731286361	7.2430196	5.487153005	6.126959032	6.454671637	5.799246428
3589	6.864589285	7.7174745	7.291031875	4.165796877	3.119148782	5.212444972
3597	1.300156699	1.5010176	1.70187847	5.980977506	7.541510824	4.420444188
3603	3.995992578	5.104623	4.550307802	3.633572465	4.205439406	3.919505936
3604	8.167814831	7.2973141	6.42681341	5.555702451	5.076733165	6.034671738
3608	8.475239738	8.0685686	8.271904178	4.183731754	9.960936226	7.07233399
3609	-0.352108471	-0.352108	-0.352108471	6.928292805	5.876364328	7.980221282
3611	8.405115873	8.1972472	8.612984573	8.074733539	6.802632252	7.438682896
3614	8.914781845	7.8721753	8.393478561	4.416563954	6.087644454	7.758724955
3615	8.491937437	5.5931087	7.042523083	6.824364836	5.789762726	4.755160616
3619	-0.352108471	-0.352108	-0.352108471	1.167362396	-0.352108471	2.686833264
3620	8.268610322	7.7533828	8.783837825	7.120198948	7.361599114	7.60299928
3622	7.144242261	5.8001998	4.456157314	-0.352108471	-0.352108471	-0.352108471
3628	-0.352108471	-0.352108	-0.352108471	4.80208491	4.697388804	4.906781016
3631	6.222276466	4.2127058	5.217491139	-0.352108471	-0.352108471	-0.352108471
3632	-0.352108471	-0.352108	-0.352108471	6.621166613	6.64213435	6.663102087
3633	5.230583641	4.3152356	4.772909641	5.909921722	3.925489738	4.91770573
3636	6.606274438	7.056304	6.831289207	8.082188235	7.927943305	8.236433165
3638	-0.352108471	-0.352108	-0.352108471	6.49953988	5.307981128	5.903760504
3646	7.913111393	6.3370236	7.1250675	8.250590956	10.78631288	5.714869029
3653	-0.352108471	-0.352108	-0.352108471	3.919505936	2.007020635	5.831991237
3654	7.560028859	7.6208136	7.681598432	4.251654614	6.56915909	8.886663565
3655	8.585414301	5.7861177	7.185765998	6.695525671	5.552208885	6.123867278
3656	3.279603139	5.9572708	4.618436987	6.083327147	7.449808407	4.716845887
3658	6.945152381	5.2216669	3.498181444	2.580385912	1.608502353	2.094444132

3660	-0.352108471	-0.352108	-0.352108471	7.402244318	6.235921161	5.069598004
3661	-0.352108471	-0.352108	-0.352108471	2.856646019	4.313596583	3.585121301
3664	-0.352108471	-0.352108	-0.352108471	1.783698732	-0.352108471	3.919505936
3667	6.01603981	5.3546856	4.693331488	-0.352108471	-0.352108471	-0.352108471
3669	6.566104981	4.7783493	2.990593553	0.228373553	0.808855576	-0.352108471
3672	-0.352108471	-0.352108	-0.352108471	7.102575825	6.479260377	5.855944928
3673	5.101393888	5.8108867	5.456140284	1.346495496	0.440372779	2.252618212
3675	7.08718283	6.8972549	7.277110733	7.969914828	8.331377891	7.608451766
3678	7.496938343	9.1220351	5.871841587	8.837967035	8.387312717	9.288621353
3681	-0.352108471	-0.352108	-0.352108471	1.383520155	3.119148782	-0.352108471
3684	-0.343508985	2.3862175	5.115943966	-0.352108471	-0.352108471	-0.352108471
3685	-0.352108471	-0.352108	-0.352108471	9.568078963	6.642035792	3.715992621
3688	8.180022589	9.4242303	8.80212642	8.028619009	9.297298521	10.56597803
3689	-0.352108471	-0.352108	-0.352108471	4.103893054	3.685573971	4.522212138
3690	-0.352108471	-0.352108	-0.352108471	6.626140968	6.921340591	6.77374078
3692	5.980548488	5.1522471	4.323945738	5.676254103	5.866795484	6.057336865
3693	8.995214539	9.0587261	9.026970344	7.364896091	6.58635054	6.975623316
3696	5.344627021	4.9140225	5.775231583	0.228373553	0.808855576	-0.352108471
3697	-0.352108471	-0.352108	-0.352108471	3.391618073	5.766929757	8.14224144
3700	4.551834616	6.9185331	5.735183859	-0.352108471	-0.352108471	-0.352108471
3703	6.63917186	5.1512515	8.127092204	7.939745657	7.757808357	7.575871057
3704	5.580935913	6.9667286	6.273832259	-0.352108471	-0.352108471	-0.352108471
3705	-0.352108471	-0.352108	-0.352108471	7.821251062	3.728885369	5.775068216
3707	8.407213441	5.2396395	2.072065653	5.876852273	5.821882502	5.849367387
3709	-0.352108471	-0.352108	-0.352108471	0.82273449	1.997577452	-0.352108471
3712	7.068912368	9.7690385	8.418975411	6.883516622	8.547225522	5.219807721
3716	6.513255867	6.502263	6.49127019	7.95008487	6.77374078	5.597396689
3718	6.249310771	7.5247254	8.800140028	6.491089529	5.316276786	4.141464043
3725	8.297586491	6.9166756	7.607131056	3.144232774	5.928559279	5.036396027
3726	5.730322944	5.6469224	5.688622647	9.773336282	4.6963668	7.234851541
3727	4.088858322	6.1573315	8.225804672	6.968740112	6.79004344	6.611346769
3728	4.547838453	7.9362581	6.242048279	7.735600725	5.789762726	3.843924727
3732	6.328948232	8.7335193	11.13809044	7.584066922	7.321623864	7.84650998
3735	6.63996159	7.9596881	5.320235067	8.267005347	7.870064962	7.473124576
3737	-0.352108471	-0.352108	-0.352108471	4.854565569	5.233567424	5.612569278
3739	5.852937462	4.4486745	7.25720046	5.052267685	4.31970398	5.784831391
3743	6.434082627	6.310617	6.187151276	5.584753022	5.575450847	5.580101935
3746	5.852937462	5.6151608	6.090714133	5.854112884	4.001245546	4.927679215
3747	5.493783215	5.5293114	5.458255064	-0.352108471	-0.352108471	-0.352108471
3751	-0.352108471	-0.352108	-0.352108471	6.776333085	5.267768765	6.022050925
3753	4.325367023	0.9842125	2.65478974	-0.352108471	-0.352108471	-0.352108471
3755	-0.352108471	-0.352108	-0.352108471	5.998406448	6.038453511	5.958359385
3756	-0.352108471	-0.352108	-0.352108471	6.082827961	6.235921161	6.38901436
3762	7.182045243	5.667464	6.424754644	6.096442209	5.342163113	6.850721306
3764	-0.352108471	-0.352108	-0.352108471	6.563015794	6.42520231	6.287388827
3767	7.56332667	5.6848521	9.441801235	8.259212257	7.144544239	7.701878248
3768	-0.352108471	-0.352108	-0.352108471	2.737063236	2.972932224	2.85499773

3772	7.084436832	5.6118503	4.139263845	8.203354599	4.66005769	1.116760781
3779	-0.352108471	-0.352108	-0.352108471	2.318472185	4.995200701	7.671929216
3780	4.439664466	5.9002924	7.360920295	-0.352108471	-0.352108471	-0.352108471
3781	6.767634029	6.1488914	7.386376647	0.624614178	0.808855576	0.440372779
3782	5.016452245	6.7334942	5.874973212	3.607797978	4.832674249	6.05755052
3785	5.749402114	5.7752316	5.723572646	-0.352108471	-0.352108471	-0.352108471
3787	-0.352108471	-0.352108	-0.352108471	6.203778275	6.532441983	6.861105692
3790	7.001877779	4.0165856	1.031293462	3.97076157	3.442033616	4.499489523
3799	6.432986623	6.460395	6.446690805	6.165870606	7.405827811	4.925913401
3800	-0.352108471	-0.352108	-0.352108471	3.119148782	3.119148782	3.119148782
3801	2.523212107	-0.352108	5.398532684	-0.352108471	-0.352108471	-0.352108471
3814	5.688622647	3.8858946	7.491350666	-0.352108471	-0.352108471	-0.352108471
3824	9.182196949	9.0010772	8.819957546	5.84130581	8.394109443	10.94691308
3831	6.816905042	6.8453896	6.788420481	-0.352108471	-0.352108471	-0.352108471
3832	4.495193957	5.873109	5.184151491	-0.352108471	-0.352108471	-0.352108471
3835	-0.352108471	-0.352108	-0.352108471	7.036396027	8.563977859	5.508814194
3836	7.826335252	6.271725	4.71711465	6.127905908	4.210409743	5.169157826
3837	3.440713359	5.6251608	7.809608183	8.154949327	8.960221794	7.349676859
3838	5.117115863	6.7355364	3.498695293	7.58484956	8.875763014	8.230306287
3839	7.558396831	7.5821735	7.534620179	6.479260377	6.497336045	6.461184708
3840	6.48210131	6.2873111	6.676891516	6.976556933	7.083361287	7.190165642
3842	-0.352108471	-0.352108	-0.352108471	7.076142219	8.363812961	5.788471477
3843	4.016045771	4.1125856	3.919505936	6.497182024	6.515031054	6.479332994
3845	7.305341479	7.2952014	7.315481548	0.289051906	-0.352108471	-0.352108471
3852	6.888413829	6.33832	7.43850766	-0.352108471	-0.352108471	-0.352108471
3855	-0.352108471	-0.352108	-0.352108471	5.760537073	5.775231583	5.745842563
3856	5.365098992	7.5180702	9.671041483	4.306712954	5.964923401	5.135818178
3858	7.251757684	6.6926949	7.810820492	3.181382067	6.12990004	9.078418014
3872	-0.352108471	-0.352108	-0.352108471	7.61641127	6.974182621	6.331953972
3875	8.85222252	11.257529	10.05487561	10.8062472	11.29508467	11.05066594
3880	6.926801914	5.7288033	8.124800574	8.347659397	7.760249447	8.935069348
3885	4.467400096	7.1287169	5.798058523	-0.352108471	-0.352108471	-0.352108471
3888	-0.352108471	-0.352108	-0.352108471	0.95025487	2.252618212	-0.352108471
3891	5.284974356	5.3152588	5.254689875	-0.352108471	-0.352108471	-0.352108471
3892	-0.352108471	-0.352108	-0.352108471	3.055081529	1.997577452	4.112585606
3895	-0.352108471	-0.352108	-0.352108471	8.636495497	6.165870606	3.695245715
3897	9.425513967	8.4327945	7.440075038	11.35465127	7.850143435	4.345635597
3899	5.842203299	6.2858739	5.398532684	1.80041638	1.549724107	2.051108653
3908	5.872552224	5.7740431	5.675534056	-0.352108471	-0.352108471	-0.352108471
3909	-0.352108471	-0.352108	-0.352108471	4.846372443	5.233567424	5.620762404
3910	-0.352108471	-0.352108	-0.352108471	6.070043966	10.87256036	1.267527571
3911	3.960558219	1.9155521	2.93805515	4.529750072	9.258195391	6.893972732
3912	7.707304336	6.6926473	8.72196135	4.139098863	8.069760072	6.104429468
3913	2.57391305	3.1277379	3.6815627	7.038110141	5.64354012	4.248970098
3915	5.217491139	7.7503844	2.684597874	11.34253253	8.431766751	5.521000967
3916	8.36756994	7.1247807	5.881991373	8.467825184	8.544815697	8.50632044
3918	7.496938343	3.3652828	11.62859393	-0.352108471	-0.352108471	-0.352108471

3920	-0.352108471	-0.352108	-0.352108471	6.963991695	4.6706451	9.25733829
3921	9.531944399	8.6490629	7.766181457	9.768473371	8.784980401	7.80148743
3925	9.397123932	8.4724392	7.547754523	10.39264277	6.841594069	8.617118418
3927	-0.352108471	-0.352108	-0.352108471	10.05262939	8.293891694	11.81136709
3938	-0.352108471	-0.352108	-0.352108471	5.998006297	4.714938642	5.35647247
3939	7.581479685	8.8021264	10.02277316	8.352984769	9.617951574	8.985468172
3945	0.707782115	2.0592582	1.383520155	8.594346013	7.019905662	10.16878636
3949	6.867257122	7.9831209	9.098984622	7.458575985	7.361907759	7.555244212
3954	7.663788002	5.302228	10.02534803	5.736129999	8.209613071	6.972871535
3956	12.27501683	10.465054	11.37003559	11.24997576	12.49803511	11.87400544
3958	7.286324647	6.6546637	7.917985573	6.968247266	8.10209829	9.235949314
3959	-0.352108471	-0.352108	-0.352108471	5.660467942	8.620162393	11.57985684
3977	5.237057889	4.797215	5.017136436	0.727922146	-0.352108471	1.807952763
3984	5.555775398	5.431791	5.493783215	10.61545944	7.228052884	3.840646324
3985	-0.352108471	-0.352108	-0.352108471	7.011395162	6.397419316	7.625371009
3987	4.233062215	5.7732919	7.313521544	7.171914945	7.832164458	6.511665431
3988	-0.352108471	-0.352108	-0.352108471	6.177737104	6.073654487	5.96957187
3992	3.968518528	4.2452143	3.69182277	4.74995208	4.245214285	5.254689875
3995	5.038624768	8.0361427	6.537383725	7.823028303	11.72108795	3.92496866
3996	7.833575671	5.8217259	6.827650764	7.425308672	3.638556	5.531932336
3998	2.822482428	3.1277379	3.432993323	9.09247134	5.604915118	7.348693229
4000	10.13413309	9.9700978	9.806062582	11.05393327	9.940058875	8.826184485
4001	7.456695988	5.691242	6.573968999	7.254608919	8.789493163	5.719724675
4007	4.502620332	6.477905	5.490262688	7.254069128	6.53365233	5.813235532
4008	6.973027794	6.2738323	5.574636724	6.064870333	6.77374078	7.482611227
4010	8.162836383	6.1042316	7.133534001	5.411522783	3.619336938	4.515429861
4015	3.572896292	3.5151412	3.45738618	6.854700456	7.664522813	8.47434517
4016	8.641099725	6.5068324	7.573966062	8.045061603	7.263433807	8.826689399
4017	10.19901888	10.495687	9.902350535	7.536609177	12.42223228	9.979420727
4025	-0.352108471	-0.352108	-0.352108471	1.727886404	2.274515706	2.821145009
4026	11.19040017	12.933061	9.4477389	11.1618143	9.585681629	10.37374796
4034	3.127737875	3.136327	3.119148782	6.235921161	6.246629235	6.225213087
4035	10.06040302	8.4502373	6.840071666	8.746539559	8.536492451	8.326445344
4037	-0.352108471	-0.352108	-0.352108471	5.054442098	5.219901144	5.385360191
4038	-0.352108471	-0.352108	-0.352108471	-0.314714384	1.601336827	3.517388038
4041	5.207939981	6.6041161	8.000292236	-0.352108471	-0.352108471	-0.352108471
4043	10.8460926	8.9595972	7.073101865	7.437584324	7.263191781	7.611976866
4046	-0.352108471	-0.352108	-0.352108471	1.167362396	2.686833264	-0.352108471
4052	-0.352108471	-0.352108	-0.352108471	3.712046455	5.529311366	7.346576277
4053	8.651772321	7.2997821	10.00376259	7.039742186	7.800060623	8.560379059
4054	-0.352108471	-0.352108	-0.352108471	3.059495471	6.164526205	9.26955694
4055	-0.352108471	-0.352108	-0.352108471	7.664251461	7.074360223	7.369305842
4059	6.107538801	6.1414231	6.073654487	-0.352108471	-0.352108471	-0.352108471
4060	6.913488898	6.9268019	6.900175883	6.804723141	6.821025802	6.788420481
4061	8.84323225	9.0709978	9.298763302	8.191745815	9.763364839	8.977555327
4063	6.273832259	6.2858739	6.261790604	-0.352108471	-0.352108471	-0.352108471
4065	6.777273875	6.1573315	5.537389119	1.918895351	2.191310813	1.646479888

4068	-0.352108471	-0.352108	-0.352108471	5.559486597	3.354285802	4.456886199
4069	8.293998691	9.2339241	7.354073309	6.589560041	7.254608919	7.919657797
4070	7.139400565	5.8529375	4.566474358	5.915686299	5.358907059	5.637296679
4071	5.260869153	8.0873886	6.674128879	-0.352108471	-0.352108471	-0.352108471
4074	-0.352108471	-0.352108	-0.352108471	6.704640042	5.412561087	4.120482133
4076	5.217491139	5.2437595	5.191222747	7.406278031	7.426473409	7.386082653
4077	-0.352108471	-0.352108	-0.352108471	9.197240908	7.081969575	8.139605241
4082	6.349474962	8.1407346	9.931994292	8.013807728	7.595907574	8.431707882
4084	6.014212707	6.9903133	6.502263028	-0.352108471	-0.352108471	-0.352108471
4086	6.450302837	6.479333	6.421272681	-0.352108471	-0.352108471	-0.352108471
4087	-0.352108471	-0.352108	-0.352108471	5.789762726	5.962507942	5.61701751
4088	5.152247113	4.6635375	5.640956761	-0.352108471	-0.352108471	-0.352108471
4089	7.053389612	8.6217478	5.48503145	6.22033882	5.877610884	6.563066757
4090	6.378374162	8.3199169	4.43683139	3.468339107	1.02644692	2.247393013
4093	-1.133968888	5.0270747	1.946552907	0.260099797	5.93862089	3.099360343
4097	4.272762536	4.5503078	4.827853067	3.195005998	3.296245714	3.245625856
4109	5.930987544	5.0469866	4.16298569	0.473647518	2.151863433	3.830079347
4111	-0.352108471	-0.352108	-0.352108471	5.355233019	5.886291167	5.620762093
4117	3.661203108	4.6189807	5.576758283	1.036211897	1.14221151	0.930212283
4122	5.417023759	6.2343267	4.599720773	6.429516632	7.943505759	7.186511196
4123	3.615867194	4.1125856	3.119148782	6.43509637	6.355161685	6.515031054
4124	5.452350409	6.5905083	7.728666198	8.073536386	7.019487643	5.965438899
4125	6.08682898	5.252355	6.921302913	7.534087958	4.426580098	5.980334028
4128	6.939871798	4.8338042	9.045939352	-0.352108471	-0.352108471	-0.352108471
4130	6.967852188	5.7984508	6.383151496	6.087644454	7.227060847	4.948228062
4133	6.861057494	6.8767254	6.845389604	6.165870606	6.174080069	6.157661143
4134	3.596683414	7.1126879	5.354685649	6.191098526	6.087644454	5.984190383
4140	6.303461245	6.9301486	5.676773855	9.006625686	6.804723141	4.602820596
4141	6.308663192	6.1754334	6.242048279	7.135985051	5.676636609	6.40631083
4144	-0.352108471	-0.352108	-0.352108471	6.163423754	6.101634422	6.225213087
4147	3.2896142	5.8242888	8.358963369	5.723485516	6.448764026	4.998207005
4148	5.273249497	5.977072	5.625160771	-0.352108471	-0.352108471	-0.352108471
4150	-0.352108471	-0.352108	-0.352108471	6.775725445	7.739056745	5.812394145
4152	-0.352108471	-0.352108	-0.352108471	6.513654132	7.044177561	7.574700991
4154	-0.352108471	-0.352108	-0.352108471	6.91804791	8.54376797	7.73090794
4157	-0.352108471	-0.352108	-0.352108471	6.369156591	3.924634899	8.813678282
4162	6.41724734	6.479333	6.355161685	0.685292531	0.440372779	0.930212283
4163	5.945769727	4.8961342	6.995405288	6.82464971	5.350639199	6.087644454
4166	8.629198696	9.1409705	8.117426847	4.604363342	7.489034095	10.37370485
4170	8.411062485	6.3095778	7.360320155	7.514700892	7.646250764	7.777800636
4171	2.290106823	6.8500114	4.570059097	5.47633612	5.165247287	5.787424953
4172	-0.352108471	-0.352108	-0.352108471	5.529311366	6.247772153	4.810850579
4173	7.495905985	8.0309191	8.565932308	1.744735086	1.939746065	1.549724107
4174	-0.352108471	-0.352108	-0.352108471	6.505960865	8.638828366	4.373093364
4175	-0.352108471	-0.352108	-0.352108471	5.958359385	5.081314597	6.835404172
4176	-0.352108471	-0.352108	-0.352108471	6.6560422	4.838925816	8.473158585
4179	9.044293783	5.7520935	7.398193617	8.487159955	4.163460405	6.32531018

4189	4.669247064	5.5913056	6.513364113	-0.352108471	-0.352108471	-0.352108471
4190	6.674128879	6.9102008	6.438057	8.855195458	6.911899654	4.96860385
4191	6.884519738	5.6886226	4.492725556	7.045200446	8.167416568	9.289632689
4192	-0.352108471	-0.352108	-0.352108471	6.51496239	6.334665756	6.695259025
4193	8.068569844	8.7151305	7.422009174	4.686592411	3.006818612	5.327044813
4194	9.081461739	8.9237395	9.239184012	7.137829112	6.792804583	6.965316847
4199	-0.352108471	-0.352108	-0.352108471	7.3583272	5.23376053	6.296043865
4200	-0.352108471	-0.352108	-0.352108471	6.355161685	6.253639684	6.456683686
4201	4.864823855	6.9398718	9.014919741	7.819895981	4.83072438	6.32531018
4209	6.027539758	5.1577063	5.592623053	9.700302023	3.454664527	6.577483275
4210	8.244000603	9.320134	7.167867183	6.217439724	6.369505651	6.521571578
4211	6.573968999	6.8215739	6.326364144	-0.352108471	-0.352108471	-0.352108471
4213	1.383520155	-0.352108	3.119148782	6.295633508	6.33538923	6.255877787
4215	-0.352108471	-0.352108	-0.352108471	5.761752405	7.409644383	6.585698394
4217	-0.352108471	-0.352108	-0.352108471	3.119148782	3.043555546	3.194742017
4221	-0.352108471	-0.352108	-0.352108471	7.38038141	4.80208491	2.22378841
4224	2.827133462	2.3862175	1.945301519	-0.352108471	-0.352108471	-0.352108471
4232	-0.352108471	-0.352108	-0.352108471	7.025721295	7.040868605	7.010573985
4233	6.898151259	5.8954442	4.892737062	6.56915909	8.392473598	4.745844581
4237	-0.352108471	-0.352108	-0.352108471	2.469725738	2.686833264	2.252618212
4238	-0.352108471	-0.352108	-0.352108471	5.069524118	5.121323858	5.095423988
4240	7.525980954	7.6091098	7.567545379	8.018899753	7.866462943	8.171336563
4245	8.130720735	7.6225236	7.114326424	11.57969288	5.520706995	8.550199937
4248	-0.352108471	-0.352108	-0.352108471	4.481992975	4.559699198	4.637405421
4249	4.587909535	6.3286006	5.458255064	7.454358007	8.483896794	6.424819219
4256	-0.352108471	-0.352108	-0.352108471	6.32531018	8.558646784	4.091973577
4257	7.096275165	5.6886226	4.280970129	6.796814879	6.374924086	7.218705673
4259	1.841742379	4.3155755	6.789408685	4.686642568	6.790856466	8.895070364
4261	7.232246321	6.2740163	6.75313132	3.732542874	1.876861374	5.588224375
4267	6.483998938	5.221876	5.852937462	9.599285526	7.878393458	11.32017759
4277	5.019618295	5.1209983	4.918238335	1.640731613	1.731739118	1.549724107
4281	-0.352108471	-0.352108	-0.352108471	5.017136436	5.054280952	4.979991919
4282	5.624956163	7.5396075	3.710304865	6.356810511	9.974349779	8.165580145
4284	-0.352108471	-0.352108	-0.352108471	5.704335255	6.123165202	5.285505308
4285	6.798388465	6.86419	6.831289207	-0.352108471	-0.352108471	-0.352108471
4287	6.927039912	6.8104362	6.693832542	3.195265415	5.8170712	4.506168307
4288	3.515141236	5.0420722	1.988210268	7.955364032	6.395718659	7.175541346
4289	3.690770627	3.136327	4.245214285	2.902991023	2.686833264	3.119148782
4293	6.969264514	6.8813157	7.057213293	5.640720806	7.528925542	6.584823174
4294	0.343135243	2.3139417	4.284748205	-0.352108471	-0.352108471	-0.352108471
4296	4.814937125	5.3546856	5.894434173	7.423441836	5.795644211	6.609543024
4298	7.042523083	7.0441776	7.040868605	7.1850208	7.204087928	7.165953671
4299	6.714347992	5.4283939	4.142439756	4.365549686	5.972765965	5.169157826
4301	-0.352108471	-0.352108	-0.352108471	4.526646886	4.927679215	5.328711544
4302	5.79087272	6.2558778	5.325867653	3.531321319	5.254689875	1.807952763
4303	6.396868383	8.0950509	4.698685899	-0.352108471	-0.352108471	-0.352108471
4311	1.501017585	-0.352108	3.354143641	7.953816429	10.20133212	5.706300735

4312	5.595965532	10.792868	8.1944168	9.153391828	8.281596292	10.02518736
4313	4.206794112	7.3893229	5.798058523	8.157941569	7.790593864	8.525289274
4314	7.196345585	9.9574592	8.576902394	8.324271493	8.339641505	8.30890148
4320	1.880238567	4.1125856	-0.352108471	7.201489776	7.233835407	7.169144145
4323	8.46749963	7.8942072	9.040792017	8.026705837	9.711407821	6.342003854
4324	-0.352108471	-0.352108	-0.352108471	5.095423988	4.567537243	5.623310733
4326	6.272107604	6.7545585	7.237009448	-0.352108471	-0.352108471	-0.352108471
4329	8.018461777	9.4062994	10.79413709	9.898470394	6.740352179	8.319411287
4337	5.017136436	5.6984289	4.335843999	-0.352108471	-0.352108471	-0.352108471
4338	-0.352108471	-0.352108	-0.352108471	6.826708063	6.853520053	6.840114058
4350	4.332763977	5.9002924	7.467820784	5.529311366	7.770566641	3.28805609
4351	6.90942626	6.6898785	7.128974067	2.902991023	3.119148782	2.686833264
4357	-0.352108471	-0.352108	-0.352108471	4.089348696	6.480600016	5.284974356
4358	-0.352108471	-0.352108	-0.352108471	5.27373346	6.840321172	8.406908884
4361	2.452184546	3.215649	1.684596322	-0.321459837	-0.695874124	-0.478596451
4363	5.247570549	5.1874117	3.640612084	-0.352108471	-0.352108471	-0.352108471
4430	-0.352108471	-0.352108	-0.352108471	7.209478834	6.377345773	8.041611895
4437	8.159410538	6.6000148	5.040619027	1.939639127	1.731739118	2.147539136
4478	7.567891492	5.9519074	9.183875572	8.484997074	8.68061739	8.582807232
4481	3.844844031	3.1277379	2.410631719	5.837940989	4.500374663	5.169157826
4487	6.067640851	5.3568958	4.64615067	5.535358938	6.192065872	4.878652004
4488	6.414258136	4.5467519	8.281764324	-0.352108471	-0.352108471	-0.352108471
4493	5.244785742	7.3621367	6.303461245	4.512470078	8.817432389	6.664951234
4500	5.181868293	5.8001998	6.418531282	0.689815323	-0.352108471	1.731739118
4501	-0.352108471	-0.352108	-0.352108471	6.78903144	5.085607333	3.382183226
4502	9.589108181	9.6063484	9.571868	10.12442447	10.12985608	10.11899287
4507	10.79155834	-0.912833	4.939362434	2.259030153	9.88105778	6.070043966
4520	5.469477566	10.06026	7.764868579	6.040996349	7.188691756	8.336387163
4521	-0.352108471	-0.352108	-0.352108471	3.919505936	3.869275727	3.969736144
4522	6.433318728	5.5725243	7.294113136	7.479274919	9.373891901	5.584657936
4524	6.745249056	6.7820557	6.708442397	6.574970966	-1.373708953	2.600631006
4547	-0.352108471	-0.352108	-0.352108471	2.698725627	3.722510298	4.746294969
4548	5.61148882	5.1933187	4.775148581	0.975533543	0.808855576	1.14221151
4580	6.573968999	8.7027753	4.445162735	5.046451356	6.165870606	7.285289856
4584	6.832572932	5.3282636	8.336882253	5.891036168	6.115255476	5.666816861
4589	-0.352108471	-0.352108	-0.352108471	2.124237496	2.147539136	2.100935855
4594	-0.352108471	-0.352108	-0.352108471	3.686004553	7.719873506	5.702939029
4598	6.332466669	6.3284751	6.336458243	-0.352108471	-0.352108471	-0.352108471
4601	-0.352108471	-0.352108	-0.352108471	2.42544395	3.119148782	1.731739118
4605	7.1250675	4.3940765	9.85605846	4.57059334	5.821410838	7.072228335
4615	3.127737875	3.136327	3.119148782	5.539921566	7.780089551	6.660005559
4616	-0.352108471	-0.352108	-0.352108471	6.913488898	6.926801914	6.900175883
4619	-0.352108471	-0.352108	-0.352108471	5.404838007	5.315258837	5.494417177
4620	9.289237391	3.417918	6.353577689	1.394345699	1.646479888	1.14221151
4624	4.452567255	7.3480175	5.90029238	-0.352108471	-0.352108471	-0.352108471
4627	10.11228834	7.743997	8.92814266	11.25553907	8.19236098	9.723950025
4628	8.739656026	7.1777769	5.615897853	7.407593833	10.38951686	4.425670803

4629	2.515697587	1.4248335	3.606561644	-0.352108471	-0.352108471	-0.352108471
4635	2.8251001	4.5390203	1.111179894	-0.352108471	-0.352108471	-0.352108471
4637	8.684000558	10.401259	12.11851696	11.09330097	10.51247478	9.931648591
4638	5.401204581	6.573969	7.746733418	6.587072453	6.034016405	7.140128501
4641	5.813348161	7.3518709	8.890393566	7.094909768	6.956409727	7.233409808
4643	-0.352108471	-0.352108	-0.352108471	8.712126777	4.385755625	6.548941201
4644	5.818193103	5.6562895	5.494385972	5.564673104	6.540914996	4.588431211
4645	7.296633267	8.235564	9.174494801	-0.352108471	-0.352108471	-0.352108471
4648	-0.352108471	-0.352108	-0.352108471	5.178880455	4.444959	5.91280191
4650	7.042523083	5.6333287	8.451717423	6.931936377	6.43736786	7.426504894
4651	7.801433444	7.4069509	7.012468401	-0.352108471	-0.352108471	-0.352108471
4659	6.589936892	4.123008	5.35647247	4.426911875	8.36682489	6.396868383
4660	7.206366837	6.8625991	6.518831283	3.325487676	3.33713378	3.348779883
4664	-0.352108471	-0.352108	-0.352108471	1.968661759	1.997577452	1.939746065
4666	9.304369252	8.7679235	9.840815009	8.808833741	9.341764806	9.075299274
4669	-0.352108471	-0.352108	-0.352108471	6.56915909	6.588465267	6.549852913
4670	7.101491044	6.9537095	7.24927257	7.959094411	5.158073079	6.558583745
4671	7.134970938	6.271725	5.408478963	0.624614178	0.808855576	0.440372779
4673	8.109483438	7.3820952	7.745789317	9.501084086	8.078573586	6.656063086
4676	6.191905554	7.0881332	6.640019393	6.764566064	7.95460543	7.359585747
4677	-0.352108471	-0.352108	-0.352108471	7.036396027	7.8349852	6.237806853
4681	-0.352108471	-0.352108	-0.352108471	2.439698732	2.151665788	2.29568226
4683	2.396854745	2.5478969	3.215469875	-0.352108471	-0.352108471	-0.352108471
4686	6.385512177	6.9245353	5.846489095	7.070474755	7.433592114	6.707357397
4688	-0.352108471	-0.352108	-0.352108471	2.795182383	-2.338281344	7.92864611
4691	9.736965645	8.4342949	9.085630255	8.907723458	8.650873139	8.394022819
4695	4.538924602	5.4283939	6.317863146	-0.352108471	-0.352108471	-0.352108471
4696	5.55876787	6.190176	4.927359749	7.651534373	3.795436658	5.723485516
4697	2.93044989	4.9045729	6.878695902	6.528762632	6.523594479	6.518426326
4700	-0.352108471	-0.352108	-0.352108471	6.686203675	6.405697902	6.545950789
4702	6.229721613	6.2312245	6.228218771	6.556689902	6.588465267	6.524914536
4704	-0.352108471	-0.352108	-0.352108471	6.2015591	5.938528832	6.070043966
4705	-0.352108471	-0.352108	-0.352108471	6.866733042	6.880482436	6.852983648
4707	-0.352108471	-0.352108	-0.352108471	1.968661759	1.997577452	1.939746065
4708	-0.352108471	-0.352108	-0.352108471	2.124237496	-0.234927404	4.483402396
4709	-0.352108471	-0.352108	-0.352108471	5.075767034	6.724665022	8.373563009
4710	6.972942587	5.6283137	6.300628137	6.039651413	5.822636979	6.256665848
4712	-0.352108471	-0.352108	-0.352108471	4.923545639	4.813836795	4.868691217
4713	-0.352108471	-0.352108	-0.352108471	6.312768514	7.679834019	4.945703008
4714	5.980334028	5.1744201	6.786247948	8.019064489	6.577066306	5.135068122
4715	-0.352108471	-0.352108	-0.352108471	5.980334028	6.002308672	5.958359385
4716	7.395208386	5.3314837	9.458933026	9.609485593	8.065096422	6.520707251
4717	-0.352108471	-0.352108	-0.352108471	5.458255064	5.458255064	5.458255064
4718	6.450513319	5.4203906	4.390267943	6.408567459	5.67247832	6.040522889
4719	-0.352108471	-0.352108	-0.352108471	2.024343053	2.051108653	1.997577452
4720	6.020381091	9.3909205	2.649841648	6.859217306	7.904812393	5.813622218
4722	-0.352108471	-0.352108	-0.352108471	9.729402426	6.926938623	4.12447482

4723	-0.352108471	-0.352108	-0.352108471	7.28629428	6.74407332	6.20185236
4725	4.145039127	6.0337853	5.089412202	6.735779686	5.827487879	7.644071493
4726	5.876276997	5.3069628	6.445591224	8.108423186	5.412561087	2.716698988
4728	-0.352108471	-0.352108	-0.352108471	6.671874638	7.417010808	5.926738469
4729	-0.352108471	-0.352108	-0.352108471	5.55876787	5.588224375	5.529311366
4731	5.842545835	7.0411335	8.239721172	5.811232824	6.818547907	6.314890366
4734	-0.352108471	-0.352108	-0.352108471	4.716325722	2.119531972	3.417928847
4735	9.528676853	8.5203418	7.512006741	7.129338548	9.258328877	8.193833712
4736	8.863871142	8.8514692	8.876273132	9.079598146	9.079990183	9.079206108
4738	4.511589627	5.6886226	6.865655667	9.91227198	5.29515642	7.6037142
4741	7.427431143	9.9143595	4.940502771	3.585121301	5.1376944	2.032548203
4744	5.279913244	6.4153434	5.847628321	-0.352108471	-0.352108471	-0.352108471
4747	7.055583237	7.242522	7.429460688	-0.352108471	-0.352108471	-0.352108471
4751	-0.352108471	-0.352108	-0.352108471	5.458255064	4.860098497	6.056411632
4752	5.250659072	6.2481452	5.749402114	-0.352108471	-0.352108471	-0.352108471
4753	-0.352108471	-0.352108	-0.352108471	6.838058947	1.199415736	4.018737342
4756	6.242048279	6.9147183	5.569378218	-0.352108471	-0.352108471	-0.352108471
4758	-0.352108471	-0.352108	-0.352108471	6.939503072	4.779731665	5.859617369
4762	1.392109249	2.8999886	-0.115770115	2.22936792	2.928781162	1.529954679
4771	7.859001556	7.33283	8.385173089	9.232911027	5.987948285	7.610429656
4776	-0.352108471	-0.352108	-0.352108471	4.018737342	4.482692559	3.554782125
4779	-0.352108471	-0.352108	-0.352108471	11.81114155	3.63178494	7.721463244
4780	4.197141794	7.6812426	5.939192221	6.369156591	5.39355723	7.344755951
4781	-0.352108471	-0.352108	-0.352108471	5.169157826	5.191222747	5.147092904
4783	6.607377459	7.6292212	5.585533755	6.056053999	5.548670833	6.563437165
4784	0.558857181	5.6966186	3.127737875	6.761711516	6.136795257	5.511878999
4790	-0.352108471	-0.352108	-0.352108471	5.704335255	3.734458756	7.674211754
4791	-0.352108471	-0.352108	-0.352108471	6.022050925	6.853351495	5.190750356
4792	7.263578805	6.1469962	6.705287512	-0.352108471	-0.352108471	-0.352108471
4793	8.954450461	3.5296461	6.242048279	2.065292044	4.482692559	-0.352108471
4795	-0.352108471	-0.352108	-0.352108471	4.08236011	6.057350711	2.107369509
4796	8.046493711	8.9293538	7.163633589	7.321702251	5.670030507	8.973373995
4799	10.60697838	6.9230377	8.765008053	4.69815125	6.384751587	5.541451418
4800	5.351365058	5.5054227	5.428393874	6.168413667	5.971674266	6.070043966
4802	2.38621749	3.1700633	1.602371631	6.756268637	5.128763636	3.501258635
4808	5.217491139	3.672385	6.76259727	-0.352108471	-0.352108471	-0.352108471
4811	7.174662452	7.2334203	7.292178125	7.213622785	5.763046254	6.48833452
4814	7.179976733	6.991795	6.803613363	-0.352108471	-0.352108471	-0.352108471
4817	-0.352108471	-0.352108	-0.352108471	6.221635402	5.821410838	5.421186274
4820	-0.352108471	-0.352108	-0.352108471	2.686833264	2.686833264	2.686833264
4824	6.537383725	5.4748067	7.599960781	-0.352108471	-0.352108471	-0.352108471
4830	8.929681391	8.9505275	8.940104426	5.639358717	7.592575489	9.545792262
4831	-0.352108471	-0.352108	-0.352108471	7.444649669	5.890891646	6.667770658
4832	7.515379209	5.5239687	6.519673958	-0.352108471	-0.352108471	-0.352108471
4833	4.622283101	8.0818246	6.352053864	8.221109655	8.552891926	8.884674198
4836	6.99593518	8.8260358	5.165834595	-0.352108471	-0.352108471	-0.352108471
4837	5.767677584	6.8651991	6.316438365	5.86942674	5.773394936	5.821410838

4839	6.224441527	6.1747497	6.199595632	-0.352108471	-0.352108471	-0.352108471
4841	9.498746247	7.3703059	5.241865462	5.345317269	11.31228186	8.328799565
4842	6.705287512	6.6898785	6.720696571	5.366045608	5.665786693	5.066304524
4849	6.640019393	5.5983174	7.681721415	8.613410144	5.159810746	6.886610445
4850	-0.352108471	-0.352108	-0.352108471	7.63716551	5.095423988	2.553682466
4851	5.553158928	6.2248629	6.896566821	7.191596866	6.771155442	6.350714019
4854	6.63917186	7.6502017	5.628142065	-0.352108471	-0.352108471	-0.352108471
4855	3.805664353	2.2586468	5.352681881	2.204376426	0.089391828	4.319361024
4861	2.65478974	3.5584878	1.751091711	-0.352108471	-0.352108471	-0.352108471
4862	6.212536209	4.5937715	7.831300868	4.492606088	6.874484741	9.256363394
4863	9.491856004	6.4333187	3.374781452	0.440372779	0.440372779	0.440372779
4867	-0.352108471	-0.352108	-0.352108471	5.775068216	5.804293869	5.745842563
4868	6.347084208	9.650417	3.043751389	7.675713744	4.484843478	6.080278611
4869	8.465783601	9.7740553	9.119919459	12.84323422	9.447697678	6.052161136
4882	6.001719323	5.6886226	5.375525971	6.175838856	5.789762726	5.403686596
4892	-0.352108471	-0.352108	-0.352108471	2.559157923	3.932093789	3.245625856
4898	-0.352108471	-0.352108	-0.352108471	7.952186442	5.631147761	10.27322512
4900	-0.352108471	-0.352108	-0.352108471	4.156200799	5.511864271	6.867527744
4904	9.214972473	9.2179805	9.211964436	9.853768603	9.878102074	9.829435133
4907	7.08718283	7.0919708	7.082394844	8.166316988	8.17464636	8.157987616
4921	-0.352108471	-0.352108	-0.352108471	4.010144333	3.119148782	4.901139884
4924	3.751483677	6.9398718	10.12825992	7.660075902	7.326356364	6.992636827
4925	-0.352108471	-0.352108	-0.352108471	6.383555469	6.204656258	6.025757047
4926	7.230452779	8.3092275	7.769840125	10.05860736	4.882763655	7.470685508
4927	-0.352108471	-0.352108	-0.352108471	5.085906025	4.80208491	4.518263795
4928	2.016914154	0.334605	3.699223337	9.063961412	3.930402636	6.497182024
4931	-0.352108471	-0.352108	-0.352108471	6.569544076	4.705049281	5.637296679
4942	5.310128272	5.6251608	5.94019327	5.924438939	6.602454754	7.280470568
4946	9.701580204	9.7178823	9.685278138	9.640182848	9.654354648	9.626011048
4947	-0.352108471	-0.352108	-0.352108471	8.318152999	6.655711164	4.993269329
4953	-0.352108471	-0.352108	-0.352108471	5.663737322	5.665786693	5.661687951
4967	4.292235829	5.463922	6.635608221	7.078372564	8.51496253	5.641782597
4973	-0.352108471	-0.352108	-0.352108471	9.523685091	7.117815169	4.711945247
4982	7.267316685	5.9002924	4.533268075	-0.352108471	-0.352108471	-0.352108471
4986	4.052529647	3.8840619	3.715594144	5.584676343	5.072643583	6.096709104
4987	5.089848387	5.4937832	5.897718043	-0.352108471	-0.352108471	-0.352108471
4988	8.500639689	8.5281884	8.473091016	7.584133668	7.204087928	7.964179408
4998	-0.352108471	-0.352108	-0.352108471	4.80208491	4.843308497	4.760861323
4999	-0.352108471	-0.352108	-0.352108471	2.252618212	2.252618212	2.252618212
5001	6.244979906	6.9029581	6.573968999	-0.352108471	-0.352108471	-0.352108471
5007	6.887397702	7.0476248	6.967511245	7.892049344	7.142338625	6.392627907
5018	7.860171405	6.991795	6.12341869	9.15197693	7.560805595	5.969634259
5019	-0.352108471	-0.352108	-0.352108471	4.621398669	6.682822443	8.744246216
5021	4.91793435	5.7494021	6.580869878	8.313256858	7.837782546	7.362308235
5027	4.634500951	3.2452353	1.855969659	1.996031974	2.686833264	1.305230684
5029	7.855015023	8.0173603	7.692669739	6.55537083	6.662724824	6.448016836
5032	-0.352108471	-0.352108	-0.352108471	6.165870606	6.915241236	5.416499976

5033	-0.352108471	-0.352108	-0.352108471	4.137528819	3.997568872	4.277488766
5034	9.545932729	10.535088	8.55677776	9.474567229	11.24645329	7.702681166
5036	4.059996578	5.2188923	6.377788019	8.016557681	8.559960985	8.288259333
5037	8.596083782	7.4338641	6.271644416	8.368124202	9.821741439	6.914506965
5042	7.067110169	5.4805543	6.273832259	-0.352108471	-0.352108471	-0.352108471
5045	3.127737875	3.136327	3.119148782	6.949901658	8.548295331	5.351507985
5048	-0.352108471	-0.352108	-0.352108471	1.971424247	5.781713541	3.876568894
5050	4.588229335	8.7600284	6.674128879	-0.352108471	-0.352108471	-0.352108471
5051	5.283085258	4.2452143	6.320956231	2.124237496	2.100935855	2.147539136
5052	5.950169773	9.2266923	7.588431014	10.88399351	9.099143573	7.314293634
5054	9.918028656	11.87624	7.959817397	11.88521834	8.123181199	10.00419977
5055	-0.352108471	-0.352108	-0.352108471	5.412561087	4.878397677	5.946724498
5058	1.454377468	8.3029535	4.878665478	-0.352108471	-0.352108471	-0.352108471
5062	2.294889372	4.9540232	3.624456288	1.53887571	2.147539136	0.930212283
5066	-0.352108471	-0.352108	-0.352108471	6.724469766	6.759061079	6.689878453
5067	-0.352108471	-0.352108	-0.352108471	0.220993655	3.228447668	6.235901681
5069	6.242048279	8.8016144	3.682482183	-0.352108471	-0.352108471	-0.352108471
5071	5.055866407	7.9461706	10.83647476	7.537461589	7.543756086	7.531167092
5076	-0.352108471	-0.352108	-0.352108471	8.324672814	3.787435184	6.056053999
5079	-0.352108471	-0.352108	-0.352108471	2.12260699	4.368644722	3.245625856
5082	3.755013405	3.1277379	2.500462346	3.658858234	3.712420317	3.685639275
5087	-0.352108471	-0.352108	-0.352108471	4.178899946	4.245214285	4.112585606
5089	-0.352108471	-0.352108	-0.352108471	8.301193095	4.530664855	6.415928975
5090	-0.352108471	-0.352108	-0.352108471	5.684018474	5.691282674	5.676754274
5092	-0.352108471	-0.352108	-0.352108471	5.575983727	5.588224375	5.563743079
5093	5.493783215	5.7281296	5.259436874	7.850864511	7.693029665	7.771947088
5094	8.512176162	9.1380121	7.886340266	8.310966687	8.458390911	8.163542463
5095	2.322223374	7.7678285	5.045025943	0.044132154	0.440372779	-0.352108471
5097	-0.352108471	-0.352108	-0.352108471	2.252618212	2.252618212	2.252618212
5098	-0.352108471	-0.352108	-0.352108471	2.64968008	3.245625856	3.841571632
5099	5.407340099	6.0379816	6.66862306	7.027509989	5.88700099	4.746491991
5100	5.939192221	5.975478	5.902906493	0.044132154	0.440372779	-0.352108471
5101	4.870582734	6.0203811	7.170179449	-0.352108471	-0.352108471	-0.352108471
5108	4.259723664	1.2565661	7.262881203	6.070426394	7.005375408	5.13547738
5110	6.502263028	6.5150311	6.489495002	-0.352108471	-0.352108471	-0.352108471
5111	-0.352108471	-0.352108	-0.352108471	1.383520155	3.119148782	1.352108471
5116	8.321094611	10.253151	6.389037818	0.862346501	3.476503449	2.169424975
5118	6.3205178	8.6059353	4.03510028	7.810573293	7.602066282	8.019080303
5119	6.463674915	6.4045531	6.522796691	7.458282885	8.86878796	6.047777809
5127	5.900507315	5.4517617	5.003016105	5.857894423	5.259641318	5.55876787
5128	3.731346161	5.3920614	7.052776556	6.118252212	4.94037052	5.529311366
5130	8.133208341	4.8713177	6.502263028	0.440372779	0.440372779	0.440372779
5134	4.430450315	4.2007007	4.315575532	2.43016825	-0.352108471	5.212444972
5142	3.955548729	7.5949144	5.775231583	4.649275848	5.369332089	6.089388329
5145	8.65426028	8.0426248	9.265895759	5.477806065	8.073723753	10.66964144
5147	-0.352108471	-0.352108	-0.352108471	5.565967891	5.385936115	5.205904339
5150	6.724232601	6.7378745	6.75151633	7.280454328	6.577066306	5.873678283

5152	4.23970035	5.5176306	4.878665478	-0.352108471	-0.352108471	-0.352108471
5154	3.733364623	4.1125856	3.354143641	6.270222109	6.31523113	6.225213087
5155	-0.352108471	-0.352108	-0.352108471	5.095423988	3.162428145	7.028419831
5156	-0.352108471	-0.352108	-0.352108471	5.412561087	5.421579811	5.403542364
5157	-0.352108471	-0.352108	-0.352108471	2.985292752	2.211943219	1.438593686
5158	7.328889755	7.9810218	7.654955776	3.682181533	4.245214285	3.119148782
5159	2.482465675	7.4115487	4.947007189	9.375316417	3.867019673	6.621168045
5162	-0.352108471	-0.352108	-0.352108471	2.211943219	2.232575625	2.191310813
5164	-0.352108471	-0.352108	-0.352108471	7.394473955	6.165870606	4.937267257
5165	7.921010324	6.2673481	9.574672526	6.794421722	5.62888596	7.959957484
5168	8.158643864	5.3129155	6.735779686	-0.352108471	-0.352108471	-0.352108471
5170	4.064821542	8.178236	6.121528769	-0.352108471	-0.352108471	-0.352108471
5172	6.736972953	8.0347672	7.385870058	5.827895643	5.50095564	6.154835645
5175	4.214559554	4.9718953	5.729231143	6.824475474	5.052267685	3.280059897
5178	4.689057965	5.2849744	5.880890748	-0.352108471	-0.352108471	-0.352108471
5184	3.515141236	3.6761388	3.354143641	7.175541346	7.18512902	7.165953671
5187	-0.352108471	-0.352108	-0.352108471	2.38621749	0.175723873	4.596711107
5188	5.493783215	4.3737166	6.613849861	6.454600772	6.315788466	6.593413078
5195	-0.352108471	-0.352108	-0.352108471	7.193753783	5.88700099	4.580248196
5198	6.434171087	6.1643979	6.299284512	6.373446373	1.544156138	3.958801255
5202	3.452931972	1.6698571	-0.113217673	5.295431597	7.98741104	2.603452154
5204	-0.352108471	-0.352108	-0.352108471	7.807367533	7.759055388	7.78321146
5205	-0.352108471	-0.352108	-0.352108471	3.611610995	2.43572631	1.259841624
5211	-0.352108471	-0.352108	-0.352108471	7.193534033	5.958359385	4.723184736
5213	6.791250076	5.2849744	3.778698636	-0.352108471	-0.352108471	-0.352108471
5214	5.251734074	6.1075388	6.963343528	2.676259628	3.245625856	3.814992084
5216	10.00821774	9.625261	10.39117453	10.93922092	11.66805155	12.39688219
5217	-0.352108471	-0.352108	-0.352108471	7.247844574	9.073780563	5.421908585
5223	6.192721106	6.7884205	5.597021732	9.225619226	9.2266493	9.224589153
5225	-0.352108471	-0.352108	-0.352108471	5.019784859	4.835573571	4.927679215
5226	6.097340265	7.4379278	6.767634029	7.232000306	3.764002741	10.69999787
5230	7.032080741	7.8683632	8.704645642	5.764169867	11.09742037	8.430795118
5238	3.249026237	3.1277379	3.006449514	10.71428473	2.958142906	6.836213819
5239	-0.352108471	-0.352108	-0.352108471	4.160912369	7.05613052	5.608521445
5245	3.127737875	5.1441106	1.111365102	7.701266713	6.824147952	5.94702919
5250	7.178557834	6.0361971	6.607377459	8.468617801	8.625813758	8.783009714
5252	6.371895774	7.9626948	7.167295284	5.983736712	6.002308672	5.965164752
5253	-0.352108471	-0.352108	-0.352108471	6.882935224	5.196367603	6.039651413
5260	-0.352108471	-0.352108	-0.352108471	2.847531327	5.254689875	0.440372779
5261	4.107444797	5.3208399	6.534235073	6.808539879	4.150994655	9.466085104
5265	5.886342689	7.5242323	6.705287512	-0.352108471	-0.352108471	-0.352108471
5269	7.505645306	8.1735045	6.837786161	6.719660678	6.859217306	6.998773933
5270	7.810989158	6.0421284	6.926558783	-0.352108471	-0.352108471	-0.352108471
5272	7.59137116	11.821644	9.706507537	7.872706255	6.684472822	9.060939687
5284	5.640665475	8.2681409	6.954403191	5.629986024	7.253857091	6.441921558
5286	5.770125669	5.164715	5.467420327	0.795123807	2.282627613	1.53887571
5287	7.354597667	7.3165435	7.392651867	5.614696198	5.863245426	5.36614697

5290	-0.352108471	-0.352108	-0.352108471	5.284974356	5.315258837	5.254689875
5293	7.082097045	5.9695814	8.194612669	7.013853304	7.785987102	8.5581209
5294	-0.352108471	-0.352108	-0.352108471	6.165870606	5.39777579	6.933965422
5295	3.733364623	4.1125856	3.354143641	5.80449981	5.893442277	5.715557343
5296	4.872224191	4.8304259	4.914022459	7.055504668	7.082394844	7.028614492
5297	3.527916452	4.6365584	2.419274494	8.831052025	7.135075351	5.439098677
5298	4.781672039	6.5373837	8.29309541	8.471132781	6.039724911	7.255428846
5300	1.383520155	-0.352108	3.119148782	7.605843994	7.690871547	7.520816442
5303	-0.352108471	-0.352108	-0.352108471	3.51558363	1.535850263	2.525716947
5305	6.071865894	6.9400554	5.203676421	-0.352108471	-0.352108471	-0.352108471
5306	5.958359385	7.4482421	4.468476637	1.223721097	0.995848332	1.451593862
5307	7.518238923	6.9819735	7.250106234	7.532560112	4.96994691	6.251253511
5310	-0.352108471	-0.352108	-0.352108471	7.178522138	7.188109812	7.168934463
5311	-0.352108471	-0.352108	-0.352108471	6.463674915	9.292499686	3.634850144
5313	-0.352108471	-0.352108	-0.352108471	0.919601171	2.191310813	-0.352108471
5314	6.151585628	5.1112032	7.19196804	-0.352108471	-0.352108471	-0.352108471
5315	10.332086	10.934418	9.729754207	14.30542405	7.167065071	10.73624456
5316	5.435976872	7.0570382	6.24650753	7.332171714	6.810436227	6.28870074
5318	7.167210339	3.5069961	5.337103237	1.036211897	1.14221151	0.930212283
5326	3.291889437	4.8164356	4.054162528	7.082065508	5.249675704	6.165870606
5327	6.604151277	6.0999564	6.352053864	6.87108183	7.017138883	6.725024778
5328	6.145394489	7.4840181	8.822641756	5.926406398	9.765422725	7.845914561
5329	7.305341479	6.8482987	7.762384298	2.560532227	4.456886199	6.353240172
5331	8.804837621	4.1996884	6.502263028	6.915018431	6.039651413	5.164284396
5332	3.157474865	2.9079292	2.658383472	-0.352108471	-0.352108471	-0.352108471
5333	6.763661796	6.2125362	5.661410622	0.874413692	-0.352108471	2.100935855
5338	5.25880671	8.4349115	2.082701885	-0.352108471	-0.352108471	-0.352108471
5339	2.58030114	4.4826926	6.385083978	9.365704316	7.954843384	8.66027385
5340	6.412258644	6.7508628	6.073654487	-0.352108471	-0.352108471	-0.352108471
5347	-0.352108471	-0.352108	-0.352108471	8.293428652	6.021986568	7.15770761
5351	4.60738245	10.040866	7.3241242	11.10338802	5.477029837	8.290208929
5352	7.587840561	6.0203811	4.452921622	7.915037632	5.512515172	6.713776402
5355	1.623552077	4.6319237	3.127737875	7.398851691	5.98922437	6.694038031
5358	7.17777694	10.260311	4.095242879	7.21467292	7.570408747	6.858937092
5361	4.13007001	7.4703296	5.800199788	4.357394898	7.31285726	10.26831962
5364	-0.352108471	-0.352108	-0.352108471	0.357532875	2.242596918	4.127660961
5370	9.016629471	5.3382703	1.65991105	0.624614178	0.808855576	0.440372779
5371	-0.352108471	-0.352108	-0.352108471	4.728824222	6.833207703	8.937591184
5372	2.170316103	4.8599664	3.515141236	6.341606863	6.309013498	6.32531018
5373	8.624887901	7.7303009	6.835713949	-0.352108471	-0.352108471	-0.352108471
5376	-0.352108471	-0.352108	-0.352108471	7.031973189	2.823385241	4.927679215
5387	5.152247113	4.2848981	6.019596136	-0.352108471	-0.352108471	-0.352108471
5393	-0.352108471	-0.352108	-0.352108471	6.931603315	6.762467494	6.847035404
5394	-0.352108471	-0.352108	-0.352108471	5.821410838	5.825167992	5.817653684
5396	-0.352108471	-0.352108	-0.352108471	5.903760504	5.926961306	5.880559702
5411	4.792303669	4.2336434	5.350963922	7.703335641	8.080426277	7.326245006
5420	-0.352108471	-0.352108	-0.352108471	4.281527167	8.437013559	6.359270363

5425	3.417170858	8.1832287	5.800199788	4.377000921	7.763851867	6.070426394
5426	-0.352108471	-0.352108	-0.352108471	3.943777666	8.77476306	6.359270363
5428	-0.352108471	-0.352108	-0.352108471	3.051990852	2.310744036	2.681367444
5429	8.304267645	7.8941486	8.71438672	6.937347744	8.026049122	9.1147505
5430	-0.352108471	-0.352108	-0.352108471	7.248377169	7.900165925	8.55195468
5432	7.207838162	7.2305473	7.18512902	6.747271561	6.773846551	6.720696571
5433	5.442901191	5.3190968	5.195292329	6.058470282	5.789762726	5.52105517
5434	5.288141214	4.617923	5.958359385	8.059969165	8.080147028	8.039791301
5435	-0.352108471	-0.352108	-0.352108471	4.456886199	4.482692559	4.43107984
5437	-0.352108471	-0.352108	-0.352108471	9.731841961	3.864410444	6.798126203
5438	4.449748927	3.9195059	4.979991919	5.651890859	5.715557343	5.588224375
5439	-0.352108471	-0.352108	-0.352108471	6.016826565	7.109633208	6.563229887
5440	-0.352108471	-0.352108	-0.352108471	6.423333563	6.109400825	6.266367194
5441	9.120851179	4.4827764	6.801813774	8.671586794	10.43870552	6.904468073
5442	-0.352108471	-0.352108	-0.352108471	7.602246173	6.454600772	5.30695537
5450	7.028007245	8.317352	9.606696801	6.351947311	6.448016836	6.255877787
5451	-0.352108471	-0.352108	-0.352108471	6.204656258	6.215464554	6.193847962
5452	5.304984318	10.155618	7.730300925	-0.352108471	-0.352108471	-0.352108471
5455	4.501003242	3.6761388	5.325867653	-0.352108471	-0.352108471	-0.352108471
5457	-0.352108471	-0.352108	-0.352108471	5.821410838	4.658016069	6.984805607
5460	5.938226189	6.3911566	6.844087039	5.291399258	9.387988127	2.194810389
5464	5.174677258	5.0041471	5.089412202	5.838252145	1.331990457	3.585121301
5465	7.795308771	6.9285962	6.061883721	5.765361695	5.456715951	6.07400744
5467	-0.352108471	-0.352108	-0.352108471	5.822181435	6.235921161	6.649660887
5468	-0.352108471	-0.352108	-0.352108471	3.919505936	1.688015621	6.15099625
5469	5.852937462	3.7895619	7.91631301	4.08236011	3.33212945	4.832590771
5476	5.77543481	5.4748867	5.625160771	7.089075912	10.4504022	3.727749622
5478	8.678611829	7.1520816	10.20514208	9.776283733	10.27817108	10.78005843
5479	-0.352108471	-0.352108	-0.352108471	8.967302913	10.30439902	7.630206805
5480	8.014817544	6.1215288	4.228239993	5.660889986	5.458255064	5.255620143
5481	8.541122289	5.8624411	7.201781675	5.001788697	9.230184491	0.773392903
5493	7.550097963	5.9002924	4.250486798	6.12990004	6.135204448	6.124595632
5494	-0.352108471	-0.352108	-0.352108471	2.024343053	3.150546562	0.898139544
5495	6.818997529	7.5191776	8.219357611	4.199252247	7.976036662	6.087644454
5499	7.195397158	6.7871694	6.378941685	6.103340827	2.185039664	10.02164199
5500	6.644566078	6.7036917	6.674128879	6.767454127	7.340170719	7.912887311
5501	6.674128879	7.2614815	6.086776305	6.641653511	6.12990004	5.618146569
5507	-0.352108471	-0.352108	-0.352108471	5.325716078	6.90025422	3.751177936
5510	5.800232101	9.1478499	7.474040996	6.276856601	6.023988658	6.529724543
5511	-0.352108471	-0.352108	-0.352108471	5.597359463	6.296043865	6.994728267
5515	5.635328348	5.7419169	5.688622647	6.939378126	8.976101269	7.957739698
5516	-0.352108471	-0.352108	-0.352108471	4.616936354	8.854623018	6.735779686
5518	7.221345935	6.1892291	6.705287512	8.166316988	7.959624489	8.373009486
5519	4.525021603	5.4729193	4.998970473	0.624614178	0.808855576	0.440372779
5520	-0.352108471	-0.352108	-0.352108471	5.47633612	5.941971976	5.010700265
5524	-0.352108471	-0.352108	-0.352108471	6.829307464	5.45223877	8.206376157
5525	4.018111536	8.4069609	6.212536209	1.601336827	-0.352108471	3.554782125

5527	-0.352108471	-0.352108	-0.352108471	6.800715119	5.523202497	8.07822774
5528	-0.352108471	-0.352108	-0.352108471	7.346697058	7.593347686	7.100046429
5530	-0.352108471	-0.352108	-0.352108471	6.596757815	6.617035395	6.576480236
5531	-0.352108471	-0.352108	-0.352108471	6.7910439	6.619531124	6.705287512
5532	8.1532074	6.9527073	5.752207276	4.796479493	6.913488898	9.030498303
5533	-0.352108471	-0.352108	-0.352108471	7.928212329	8.103616925	7.752807732
5534	4.528054005	5.980334	7.432614051	-0.352108471	-0.352108471	-0.352108471
5536	-0.352108471	-0.352108	-0.352108471	7.254409547	8.297316317	7.775862932
5537	-0.352108471	-0.352108	-0.352108471	7.72540815	8.171122555	8.616836959
5538	-0.352108471	-0.352108	-0.352108471	5.849605198	6.622237124	6.235921161
5546	7.347881921	6.2420483	5.136214637	8.783254855	7.203585592	7.993420223
5547	-0.352108471	-0.352108	-0.352108471	3.422290724	5.923493182	8.42469564
5549	7.115369054	7.0314051	7.199333039	1.58479924	1.731739118	1.437859363
5550	7.377755134	8.0394819	6.716028359	1.563782278	5.910505782	3.73714403
5552	5.898613566	8.1246837	3.672543443	4.36512151	5.412561087	6.460000665
5562	6.715741874	7.878396	7.297068944	0.044132154	-0.352108471	0.440372779
5566	5.798459316	5.873346	5.723572646	7.326356364	7.342696275	7.310016454
5567	-0.352108471	-0.352108	-0.352108471	3.73714403	4.958117366	2.516170694
5571	6.242048279	5.2767044	7.207392127	-0.352108471	-0.352108471	-0.352108471
5573	6.518125564	6.4864005	6.502263028	7.250957754	3.966958852	10.53495666
5575	-0.352108471	-0.352108	-0.352108471	6.450302837	4.648100738	8.252504937
5578	7.143666534	6.9003452	6.657023947	7.012479653	5.838263192	6.425371423
5579	4.044792187	6.2465075	8.448222872	0.044132154	0.440372779	-0.352108471
5580	6.396868383	6.4105853	6.383151496	4.977710418	5.054280952	4.901139884
5584	5.084523138	4.4715058	3.85848839	5.410465611	5.765983138	5.588224375
5585	5.688622647	5.236867	6.140378306	6.806576698	4.80208491	2.797593122
5589	6.310616952	6.1999612	6.421272681	8.301885534	8.320892968	8.2828781
5591	7.072259769	6.7640924	7.380427174	9.209701518	7.887166737	6.564631956
5592	5.217491139	5.2437595	5.191222747	-0.352108471	-0.352108471	-0.352108471
5594	2.879090203	4.1511923	3.515141236	6.577483275	3.980594444	9.174372107
5595	-0.352108471	-0.352108	-0.352108471	4.764823754	9.089053491	6.926938623
5597	-0.352108471	-0.352108	-0.352108471	5.800199788	8.009687299	3.590712276
5601	8.562476809	6.898125	7.730300925	2.252618212	2.252618212	2.252618212
5603	9.08889005	8.3276006	7.566311181	5.590825591	5.427564391	5.754086791
5604	6.317834233	6.3439771	6.291691359	-0.352108471	-0.352108471	-0.352108471
5605	7.143957189	7.515234	6.772680333	9.5924984	7.472973925	5.353449449
5606	7.03750086	8.5990222	7.818261523	8.401371506	6.574218611	10.2285244
5609	6.151585628	6.3748784	5.928292891	3.119148782	3.119148782	3.119148782
5610	5.587178629	5.7855384	5.388818875	1.167362396	-0.352108471	2.686833264
5611	3.547436804	6.5235082	5.035472495	2.124237496	2.100935855	2.147539136
5612	6.242048279	4.0884897	8.39560684	-0.352108471	-0.352108471	-0.352108471
5613	6.093476582	6.7884205	5.398532684	5.723485516	5.731413688	5.715557343
5616	3.960676558	3.6761388	4.245214285	1.968661759	1.997577452	1.939746065
5621	8.326761998	5.6082605	6.967511245	7.970671981	7.348693229	6.726714476
5625	4.107686153	6.056054	8.004421845	-0.352108471	-0.352108471	-0.352108471
5631	-0.352108471	-0.352108	-0.352108471	10.77213557	7.97624566	5.180355749
5635	-0.352108471	-0.352108	-0.352108471	6.070043966	6.101634422	6.038453511

5648	-0.352108471	-0.352108	-0.352108471	7.254608919	8.132937082	6.376280757
5649	4.830141517	5.3546856	5.879229781	-0.352108471	-0.352108471	-0.352108471
5650	1.392109249	3.136327	-0.352108471	5.118656602	6.04366503	4.193648173
5654	5.354685649	3.4663032	7.243068121	7.401312755	5.539204947	6.470258851
5660	8.089245536	6.789528	9.38896304	6.548133264	11.11040755	8.829270406
5669	6.147142694	6.7802071	6.463674915	-0.352108471	-0.352108471	-0.352108471
5672	7.31614654	6.0629293	6.689537938	7.099706219	7.109037535	7.090374904
5673	1.946552907	3.3296045	0.56350129	3.320702002	2.629898059	4.011505944
5676	2.830693931	0.9297832	1.880238567	6.128600107	7.418881452	6.77374078
5678	6.26796092	7.7405013	7.004231132	2.650522147	4.621880905	0.679163388
5681	0.206100132	1.3921092	2.578118366	6.829307464	7.617792651	6.040822276
5682	5.749402114	7.3447591	4.154045119	7.214853916	7.889819055	8.564784194
5683	1.681008233	3.5151412	5.349274238	8.656819156	9.733593924	7.580044388
5684	6.090808341	4.829477	7.352139653	6.179989181	6.782044062	7.384098943
5685	-0.352108471	-0.352108	-0.352108471	5.357748126	9.677593766	7.517670946
5686	1.383520155	-0.352108	3.119148782	10.23653294	3.027159563	6.631846251
5687	-0.352108471	-0.352108	-0.352108471	5.333331364	4.449748927	3.566166491
5688	6.463674915	6.479333	6.448016836	6.926938623	6.946414218	6.907463027
5689	-0.352108471	-0.352108	-0.352108471	6.866118875	6.820702064	6.775285253
5690	6.729573474	6.3989203	6.068267173	7.57353257	8.40002958	9.226526591
5691	-0.352108471	-0.352108	-0.352108471	4.79472922	3.253077834	6.336380607
5692	7.398193617	4.7668679	10.02951931	-0.352108471	-0.352108471	-0.352108471
5693	-0.352108471	-0.352108	-0.352108471	3.357516707	6.087644454	8.817772202
5694	-0.352108471	-0.352108	-0.352108471	6.739878825	5.856478714	7.623278936
5695	7.932742872	7.3826486	8.482837134	5.257632347	8.635028156	6.946330252
5696	4.178899946	2.9191374	5.438662516	4.08236011	4.245214285	3.919505936
5697	2.894657322	0.5657008	5.223613834	-0.352108471	-0.352108471	-0.352108471
5699	6.735779686	7.0156808	6.455878557	5.918018286	5.663737322	5.409456357
5700	5.197387513	6.4978691	5.847628321	6.266367194	5.382807782	7.149926605
5702	-0.352108471	-0.352108	-0.352108471	7.332349758	7.829997332	8.327644905
5704	6.463674915	6.1497094	6.777640385	4.80208491	5.263461501	4.340708319
5705	9.3962061	5.9987621	7.697484075	5.049594135	5.494417177	5.939240219
5706	6.090808341	4.9036944	7.277922313	8.097708198	6.671874638	5.246041078
5707	-0.352108471	-0.352108	-0.352108471	6.183918153	7.643059643	6.913488898
5708	8.699804973	7.2980036	7.998904298	5.964300094	9.170805021	7.567552558
5709	7.296464716	7.3154815	7.277447884	7.271069954	7.29520141	7.246938498
5710	4.933668324	6.6150723	5.774370319	6.266658347	7.117815169	7.968971991
5711	1.392109249	3.136327	-0.352108471	6.266367194	6.276856601	6.255877787
5713	6.044283763	5.8529375	5.66159116	5.250920127	7.220922195	6.235921161
5714	3.515141236	3.6761388	3.354143641	8.520420514	6.838620913	7.679520714
5715	-0.352108471	-0.352108	-0.352108471	7.463736672	9.264017869	5.663455476
5718	8.321094611	9.0310484	7.611140868	3.782624606	9.205635116	6.494129861
5720	7.509846649	6.4737434	6.991795048	6.027163813	9.707390128	7.867276971
5723	9.919831855	8.3510264	6.78222101	5.976946737	4.510937597	5.243942167
5724	8.469475329	5.451894	6.960684686	6.240522534	5.688622647	5.13672276
5725	8.744320385	8.8322984	8.656342398	7.649062677	7.682866779	7.615258575
5728	2.563219874	2.5421313	2.563214568	-0.352108471	-0.352108471	-0.352108471

5730	-0.352108471	-0.352108	-0.352108471	5.760537073	6.840168031	4.680906115
5731	-0.352108471	-0.352108	-0.352108471	4.471069827	4.956774616	4.713922221
5732	2.613734404	5.4283939	8.243053345	-0.352108471	-0.352108471	-0.352108471
5734	5.089412202	6.0490699	4.12975451	-0.352108471	-0.352108471	-0.352108471
5739	-0.352108471	-0.352108	-0.352108471	5.126019982	4.80208491	4.478149838
5740	6.777690144	8.6226362	7.700163162	5.409216477	3.283814455	7.534618499
5742	-0.352108471	-0.352108	-0.352108471	7.272749174	5.575983727	3.879218279
5745	-0.352108471	-0.352108	-0.352108471	6.415928975	6.429470164	6.402387785
5747	-0.352108471	-0.352108	-0.352108471	1.97329804	3.585121301	5.196944563
5756	-0.352108471	-0.352108	-0.352108471	6.774697242	8.851930875	7.813314058
5757	11.05028427	8.5013948	9.775839531	9.13101167	8.556134392	9.705888947
5763	11.04742197	10.409557	9.771692401	10.14646054	8.59821042	11.69471066
5768	8.694577421	6.8354301	4.976282689	7.753639915	8.998169197	8.375904556
5770	-0.352108471	-0.352108	-0.352108471	3.119021332	2.381862494	3.856180171
5780	-0.352108471	-0.352108	-0.352108471	5.663737322	6.690311581	4.637163062
5783	-0.352108471	-0.352108	-0.352108471	6.110765987	8.413620395	7.262193191
5786	8.182202312	4.1857257	6.183964016	-0.352108471	-0.352108471	-0.352108471
5793	-0.352108471	-0.352108	-0.352108471	6.12990004	5.616968343	6.642831737
5795	4.097886718	6.3370956	5.217491139	6.955144685	0.883867186	3.919505936
5797	-0.352108471	-0.352108	-0.352108471	4.884033187	6.637040959	5.760537073
5802	6.48561815	8.5869477	7.536282912	-0.352108471	-0.352108471	-0.352108471
5806	6.242048279	4.9383841	7.545712418	5.916834063	5.169157826	4.421481588
5810	7.079753946	7.3836613	7.687568675	3.617543668	5.955959659	4.29437565
5814	7.691549379	7.3327669	8.050331905	6.899457994	7.109037535	6.689878453
5817	7.764328239	7.507402	8.021254448	7.122286018	7.236989963	7.007582074
5819	6.070200859	6.8231808	6.446690805	6.758753021	7.952329552	5.56517649
5820	4.26871449	4.617923	3.919505936	5.775068216	5.804293869	5.745842563
5824	7.927924624	6.7357797	5.543634748	5.760537073	5.775231583	5.745842563
5826	5.354685649	5.3835036	5.325867653	0.440372779	0.440372779	0.440372779
5828	3.196680244	4.798745	6.400809669	6.144107	5.117340897	4.090574795
5829	9.645743308	7.4834077	5.321072042	9.225836049	5.215846118	7.220841083
5830	-0.352108471	-0.352108	-0.352108471	8.722680124	6.926938623	5.131197122
5831	-0.352108471	-0.352108	-0.352108471	2.274515706	-0.352108471	4.901139884
5832	-0.352108471	-0.352108	-0.352108471	6.913488898	6.926801914	6.900175883
5833	-0.352108471	-0.352108	-0.352108471	1.902694985	6.455104906	4.178899946
5834	6.183964016	5.4749131	6.89301491	9.81208411	7.37970607	4.94732803
5836	-0.352108471	-0.352108	-0.352108471	7.066165076	6.266367194	5.466569311
5837	-0.352108471	-0.352108	-0.352108471	3.879139969	7.395453388	5.637296679
5860	2.492871856	5.8529375	9.213003067	2.826082037	1.362108442	4.290055631
5861	-0.352108471	-0.352108	-0.352108471	6.34460976	9.171006954	7.757808357
5862	7.004231132	4.7646761	9.243786148	-0.352108471	-0.352108471	-0.352108471
5863	2.313941724	4.9799919	-0.352108471	-0.352108471	-0.352108471	-0.352108471
5865	8.974643259	6.3002	7.63742161	7.220185319	6.381272668	5.542360017
5867	3.515141236	3.6761388	3.354143641	1.167362396	-0.352108471	2.686833264
5869	7.616245176	8.7833521	6.449138221	7.596690814	7.311506909	7.026323003
5870	6.778248209	6.4027684	6.590508304	5.756733586	6.087644454	6.418555323
5872	7.286324647	7.2952014	7.277447884	6.225217789	6.135204448	6.31523113

5873	8.945067903	7.7599784	8.352523148	5.38675695	7.948074799	2.825439102
5874	-0.352108471	-0.352108	-0.352108471	2.686833264	2.686833264	2.686833264
5876	-0.352108471	-0.352108	-0.352108471	7.892802136	5.688622647	3.484443158
5877	-0.352108471	-0.352108	-0.352108471	4.016045771	5.109937633	2.922153909
5878	6.79254856	5.0858359	5.939192221	8.707312022	7.230904119	5.754496216
5879	8.18875661	5.8447962	7.016776417	7.222175024	8.725607015	10.22903901
5880	-0.352108471	-0.352108	-0.352108471	5.983736712	6.404640822	5.562832602
5883	5.395440136	5.1110414	5.253240792	-0.352108471	-0.352108471	-0.352108471
5884	2.412645809	6.1320496	9.851453467	-0.352108471	-0.352108471	-0.352108471
5885	-0.352108471	-0.352108	-0.352108471	3.919505936	-0.141682854	7.980694725
5886	-0.352108471	-0.352108	-0.352108471	6.99529589	6.946414218	7.044177561
5887	5.079283667	5.3152588	4.843308497	9.358979356	6.644415048	8.001697202
5888	5.369412555	4.8745632	4.396587441	-0.352108471	-0.352108471	-0.352108471
5894	-0.352108471	-0.352108	-0.352108471	7.741995652	6.693937107	5.645878563
5897	3.711299307	5.4283939	7.145488441	-0.352108471	-0.352108471	-0.352108471
5898	3.515141236	3.6761388	3.354143641	4.583163537	7.003799972	9.424436408
5899	7.714641217	7.7464157	7.682866779	6.822208827	6.841511354	6.802906299
5900	-0.352108471	-0.352108	-0.352108471	7.87032243	6.240686905	7.055504668
5901	8.347852706	6.6134726	10.08223276	10.2000135	6.99162151	8.595817505
5902	6.705287512	8.26244	5.148135068	6.056053999	6.484242908	5.627865089
5903	-0.352108471	-0.352108	-0.352108471	6.570965708	5.345753061	5.958359385
5905	4.197727899	8.5405853	6.369156591	6.710202988	7.772256866	8.834310744
5906	4.417353062	5.7155573	3.119148782	5.78865122	5.731413688	5.845888751
5908	5.520811398	5.8703726	5.695591985	7.451058303	10.3295579	4.572558707
5911	-0.352108471	-0.352108	-0.352108471	6.165870606	6.103069589	6.228671623
5912	4.178899946	4.1125856	4.245214285	6.836213819	6.859202315	6.813225323
5914	1.383520155	-0.352108	3.119148782	5.939192221	5.958359385	5.920025058
5915	1.66201518	1.0528134	2.271216921	2.558363117	2.846251358	2.270474876
5916	-0.352108471	-0.352108	-0.352108471	3.786680653	5.88700099	7.987321327
5917	6.14338204	6.4142581	6.685134231	4.95129188	4.3688235	4.66005769
5919	-0.352108471	-0.352108	-0.352108471	3.010220895	3.919505936	2.100935855
5921	6.502263028	6.9508371	6.053688954	-0.352108471	-0.352108471	-0.352108471
5922	2.989737274	4.9470072	6.904277104	-0.352108471	-0.352108471	-0.352108471
5925	1.569874521	1.2365987	1.21542145	-0.352108471	-0.352108471	-0.352108471
5928	3.559920336	6.7206321	5.140276207	5.145863224	5.669407383	6.192951543
5929	7.216041095	7.1753677	7.134694261	6.113562147	8.30460032	7.209081234
5931	-0.352108471	-0.352108	-0.352108471	9.218546465	3.926800265	6.572673365
5932	3.805664353	2.3524222	5.258906487	6.243037448	6.296043865	6.349050283
5933	7.659808125	6.5493574	8.770258851	4.857983236	6.492651928	4.175317582
5935	7.753479539	8.7047121	6.802246986	8.089784342	6.438826876	7.264305609
5937	6.705287512	6.7206966	6.689878453	5.88700099	5.893442277	5.880559702
5939	9.467246631	8.6421827	10.29231054	8.721480187	6.60898181	7.665230999
5954	7.607131056	7.6448941	7.569368047	8.881957636	11.88274223	5.881173041
5955	-0.352108471	-0.352108	-0.352108471	7.604813664	7.629372342	7.580254986
5962	-0.352108471	-0.352108	-0.352108471	4.41128052	6.165870606	7.920460692
5966	4.184097316	5.1507818	6.117466246	1.0954352	1.58479924	2.074163281
5967	4.501003242	3.6761388	5.325867653	-0.352108471	-0.352108471	-0.352108471

5969	4.368823643	5.3835036	3.354143641	-0.352108471	-0.352108471	-0.352108471
5970	-0.352108471	-0.352108	-0.352108471	7.221207448	5.723485516	4.225763583
5971	-0.352108471	-0.352108	-0.352108471	4.80208491	4.637102219	4.967067602
5976	-0.352108471	-0.352108	-0.352108471	5.01942989	7.881175785	6.450302837
5977	5.940304865	5.8529375	5.765570058	7.577703846	4.161627183	5.869665514
5979	3.234787761	1.7283626	2.481575183	-0.352108471	-0.352108471	-0.352108471
5980	6.314875728	6.3681558	6.421435859	-0.352108471	-0.352108471	-0.352108471
5981	-0.352108471	-0.352108	-0.352108471	5.911756406	6.859217306	7.806678205
5982	7.419011579	9.0916667	5.746356464	7.338535707	6.630725721	6.984630714
5986	-0.352108471	-0.352108	-0.352108471	4.297639083	4.561390363	4.033887802
5987	-0.352108471	-0.352108	-0.352108471	7.530018305	7.309496697	7.419757501
5989	6.932408367	4.6679912	5.800199788	5.596916991	5.605609608	5.588224375
5992	3.011719947	2.4512907	1.890861457	-0.352108471	-0.352108471	-0.352108471
5997	-0.352108471	-0.352108	-0.352108471	4.927679215	8.20335113	1.6520073
5998	-0.352108471	-0.352108	-0.352108471	5.923493182	3.838174394	8.008811971
5999	5.749402114	3.3072346	8.19156966	-0.352108471	-0.352108471	-0.352108471
6001	7.17777694	9.4899576	4.86559632	6.519616277	5.689242659	6.104429468
6002	6.383199651	5.3920614	4.400923066	1.618250677	2.686833264	3.75541585
6007	6.644015295	6.5365333	6.429051302	5.178880455	3.968518095	6.389242815
6009	-0.352108471	-0.352108	-0.352108471	7.036396027	7.044177561	7.028614492
6015	-0.352108471	-0.352108	-0.352108471	6.796400047	7.776249247	7.286324647
6016	3.228537319	3.5996124	3.41407487	5.44897977	5.685576344	5.212383197
6023	-0.352108471	-0.352108	-0.352108471	6.488107881	5.141882827	7.834332935
6029	4.978979723	6.0384535	3.919505936	6.084917755	5.191222747	6.978612763
6046	8.400533141	7.6071311	6.813728971	1.117721484	1.305230684	0.930212283
6047	-0.352108471	-0.352108	-0.352108471	6.12990004	8.990180204	3.269619876
6048	-0.352108471	-0.352108	-0.352108471	8.544945343	4.454114295	6.499529819
6050	7.010716847	6.1956255	7.82580823	8.284444579	6.052042882	10.51684628
6051	6.507929727	6.4636749	6.419420103	0.793818797	-0.352108471	1.939746065
6059	-0.352108471	-0.352108	-0.352108471	6.816905042	6.845389604	6.788420481
6091	-0.352108471	-0.352108	-0.352108471	1.946552907	4.245214285	-0.352108471
6092	2.65059567	2.7500141	2.551177233	-0.352108471	-0.352108471	-0.352108471
6093	3.691501207	4.0160458	4.340590335	5.958359385	2.972934177	8.943784592
6095	5.160932683	6.0908083	7.020683998	-0.352108471	-0.352108471	-0.352108471
6102	4.520973298	3.6349643	5.406982273	7.068740219	5.749166302	8.388314137
6117	3.515141236	3.8489845	3.181297931	4.869149724	5.406707798	5.944265872
6118	-0.352108471	-0.352108	-0.352108471	2.31950733	5.233567424	8.147627517
6122	11.58370674	9.9382481	8.292789555	9.965836102	10.22803011	9.703642094
6124	8.799531962	9.1191351	9.438738218	8.660472311	10.14102893	11.62158555
6125	10.00003003	10.649152	9.350907897	7.299135817	10.34595789	8.822546856
6128	9.863325634	9.1867852	10.53986605	6.586953039	9.577397725	12.56784241
6129	8.964463113	8.7148846	9.214041644	10.69623159	6.377349406	8.5367905
6130	9.679800881	6.9431942	12.41640758	8.876480624	9.910622579	10.94476453
6132	9.372379889	9.7706862	10.16899256	9.735397181	10.5650861	10.15024164
6133	7.042523083	7.0441776	7.040868605	8.776996002	8.779674384	8.77431762
6134	9.518807513	8.8802716	10.15734345	9.100666434	9.823746577	10.54682672
6135	9.582998077	11.04043	8.125565805	9.607901601	8.770959505	9.189430553

6136	9.225777076	8.4473211	7.668865066	9.497516577	10.56355516	8.431477994
6137	10.31260281	10.527327	10.41996502	10.6671273	9.069370303	12.26488429
6138	8.414326396	8.4583909	8.370261881	9.775434224	9.78174292	9.769125527
6139	6.872070602	6.7380116	6.805041124	8.505185291	8.312490055	8.697880528
6141	8.582055395	6.98769	7.784872705	8.635621212	8.678966888	8.65729405
6142	9.412559271	9.9797034	8.845415182	10.60319195	9.879198478	11.32718541
6143	7.31927011	8.5918166	7.955543336	10.82619776	8.179807787	9.503002771
6144	8.39909175	8.4388724	8.359311122	9.069238097	9.099093789	9.039382405
6146	7.858118037	7.0113718	8.704864224	9.693205037	8.902554856	8.111904675
6147	7.856625788	10.105839	5.607412747	9.815889992	10.53626571	9.095514279
6150	6.695550336	6.6204961	6.658023207	4.092726653	3.119148782	5.066304524
6152	8.516595168	8.5400828	8.493107512	9.469109208	9.440152969	9.498065447
6154	7.401120142	7.2780304	7.154940626	11.3085261	9.463758151	7.618990198
6155	10.37638101	7.5519911	8.964186059	8.267948681	9.604109366	10.94027005
6156	7.98291635	6.4624076	7.222661978	8.698082551	9.057717404	9.417352256
6157	7.533336972	5.0992002	9.967473748	11.64690099	6.963077234	9.304989114
6158	7.917256534	10.016216	12.11517522	9.426705004	9.865130755	9.645917879
6159	9.503834851	7.8429568	11.16471291	10.27491064	6.346208053	14.20361324
6160	9.134043485	8.6496871	9.618399839	9.446218349	11.35803936	7.534397342
6161	10.54749483	8.4384159	6.329336959	10.66246074	9.732999246	10.19773
6164	7.820805959	8.1843568	8.547907596	10.82866414	8.55232631	6.275988483
6165	7.819969389	8.3631259	8.906282394	8.908454609	8.11940121	9.697508008
6166	6.135286094	5.4937832	4.852280336	9.009854107	7.405964559	8.207909333
6167	8.700400674	9.1544527	8.246348608	9.694045641	9.606348362	9.78174292
6168	10.47409317	9.7981601	9.122226932	8.870655037	10.72281321	7.018496865
6169	2.628412179	10.6193	6.623855918	7.963843178	8.423814496	8.883785814
6170	8.14876014	8.7588381	8.453799139	11.37095336	9.061513073	6.752072787
6171	10.0568025	10.555879	9.557725821	9.525471912	10.55302715	11.58058239
6173	2.351086241	1.5977964	3.104376103	6.857425346	5.517552795	6.187489071
6175	11.12426785	8.8454367	9.984852293	10.25364993	8.335013437	12.17228643
6176	9.886726279	10.243797	10.60086841	10.37003732	10.67524021	10.06483443
6181	8.040517613	8.0660974	8.014937823	9.182422154	9.146863799	9.21798051
6182	7.062915477	4.7376693	5.90029238	7.821918011	7.331272742	6.840627473
6183	-0.352108471	-0.352108	-0.352108471	7.148009044	7.149537332	7.146480757
6184	4.860692968	5.9960948	5.428393874	5.290194195	9.657759536	7.473976865
6185	-0.352108471	-0.352108	-0.352108471	6.919361747	5.780345578	8.058377915
6187	11.14659317	9.4191061	10.28284962	9.741734302	10.84978508	11.95783586
6188	9.387722965	8.8639222	8.340121533	8.812325416	10.39050274	9.601414078
6189	8.686519674	9.7617848	7.611254501	9.293477065	8.25213417	10.33481996
6191	8.324919686	9.2489934	7.400845997	8.987543758	8.024492115	9.9505954
6193	8.587823739	8.5865437	8.589103829	9.234305479	9.235285343	9.233325615
6194	6.680999206	8.4889268	10.29685444	8.475393846	10.11938028	9.297387062
6196	5.172336182	5.5370351	5.354685649	2.38621749	5.124543452	-0.352108471
6198	3.058761005	2.8031255	2.547489908	-0.352108471	-0.352108471	-0.352108471
6199	-0.352108471	-0.352108	-0.352108471	3.245625856	4.549853921	1.941397791
6201	8.177335428	7.8444622	8.510208661	9.693616298	9.106169981	8.518723663
6202	5.773762627	8.105981	6.939871798	9.732888763	7.749880648	11.71589688

6203	9.038224509	7.9993398	10.07710918	9.912986202	11.60608284	8.21988956
6204	6.464069271	6.6429094	6.821749506	5.945093434	7.377124155	8.809154876
6205	9.164371527	10.361543	7.967200474	9.825041112	10.77911315	8.87096908
6206	7.321960058	8.881218	10.44047599	8.738089432	8.608832687	8.867346176
6207	1.199878824	5.0555969	3.127737875	7.195218384	8.983973515	8.089595949
6208	6.960561228	10.847806	8.90418384	8.853476829	9.519491262	8.187462395
6209	9.28357144	9.2807862	9.286356696	8.618513237	8.619270255	8.617756219
6210	8.077078623	7.636173	7.856625788	9.906218468	8.679356586	7.452494703
6217	4.550307802	5.9216036	3.179012034	7.889583011	9.212323673	10.53506434
6218	9.098135288	8.668575	8.239014714	9.863325634	9.232999971	10.4936513
6222	9.089819636	9.0769458	9.102693505	9.779344077	9.80105521	9.757632943
6223	11.70152004	9.3029401	10.50223007	11.63411304	9.925774412	8.217435784
6224	7.307996819	9.2417756	8.27488623	11.46247863	7.764834804	4.067190983
6227	4.428068644	4.6929891	4.957909594	8.963997453	11.8412019	6.086793006
6228	7.897268052	7.9270228	7.867513291	9.610255777	9.606348362	9.614163193
6229	8.97702576	8.8905959	9.063455621	9.641057557	9.631056556	9.651058557
6230	8.466440998	8.4166969	8.516185087	9.105466455	9.076945767	9.133987144
6232	8.335824565	7.9923409	8.679308186	11.34399961	9.284554373	7.225109134
6233	8.105367245	6.8312892	5.55721117	8.50066116	6.751080379	10.25024194
6234	4.544082676	8.4604434	6.502263028	7.488209285	7.820497371	8.152785456
6235	4.116251281	6.0079044	5.062077834	5.128697496	8.863128384	6.99591294
6237	4.692989119	5.6588605	7.727117712	3.134000228	4.078372564	4.022744899
6238	7.198930368	6.2125362	5.22614205	1.873849414	0.23213893	3.515559898
6240	-0.352108471	-0.352108	-0.352108471	5.800199788	5.825167992	5.775231583
6241	2.826221886	5.9181605	4.372191214	6.076663769	6.164674546	6.252685322
6249	6.883204546	4.9951799	5.939192221	6.165870606	3.666328863	8.665412349
6251	6.726948447	7.9407334	9.154518362	6.180405012	6.355161685	6.005648339
6257	-0.352108471	-0.352108	-0.352108471	4.338279001	4.995200701	5.652122401
6259	-0.352108471	-0.352108	-0.352108471	7.454358007	7.443759962	7.464956052
6261	7.004231132	6.9612972	7.047165038	-0.352108471	-0.352108471	-0.352108471
6262	6.502263028	6.5150311	6.489495002	-0.352108471	-0.352108471	-0.352108471
6272	7.270779117	6.2125362	5.1542933	7.039990107	7.643140836	8.246291565
6275	8.153393761	7.3173605	8.989427022	8.241142258	5.91433043	10.56795409
6277	9.357751616	11.395661	10.37670654	9.237777341	12.38552966	10.8116535
6279	-0.352108471	-0.352108	-0.352108471	5.39648617	6.183039282	5.789762726
6281	6.136577919	4.6929891	3.249400319	5.185388519	5.295431597	5.405474676
6282	8.12917596	9.1133925	10.09760912	7.932796172	11.50037722	9.716586697
6284	-0.352108471	-0.352108	-0.352108471	5.705814628	5.665786693	5.745842563
6299	5.937483625	6.1515856	6.365687632	4.66005769	5.627473494	3.692641885
6301	7.137727412	7.2723922	7.003062599	7.854825187	8.08500447	7.969914828
6302	4.154872907	5.9752582	2.334487648	1.787060316	4.168888164	2.97797424
6303	6.303461245	6.3152311	6.291691359	-0.352108471	-0.352108471	-0.352108471
6304	-0.352108471	-0.352108	-0.352108471	6.958148	5.845499835	4.73285167
6305	-0.352108471	-0.352108	-0.352108471	6.758753021	6.788420481	6.729085562
6307	6.139733886	5.6083948	6.67107294	6.3533338	7.010964757	5.695702844
6309	5.725231643	4.4404417	7.010021611	1.493791735	1.437859363	1.549724107
6311	-0.352108471	-0.352108	-0.352108471	5.299362255	6.270222109	7.241081962

6315	-0.352108471	-0.352108	-0.352108471	1.383520155	3.119148782	-0.352108471
6319	-0.352108471	-0.352108	-0.352108471	6.34823572	7.604813664	8.861391608
6320	5.217491139	5.4143746	5.020607728	7.117815169	12.14497543	2.090654904
6322	7.746459497	4.9882456	6.367352566	4.122478308	3.042501174	5.202455441
6326	5.9536284	5.6886226	5.423616894	1.631676003	3.615460478	-0.352108471
6331	-0.352108471	-0.352108	-0.352108471	5.738172595	4.927679215	4.117185835
6335	2.736529761	5.825168	-0.352108471	-0.352108471	-0.352108471	-0.352108471
6338	-0.352108471	-0.352108	-0.352108471	1.596660082	1.14221151	2.051108653
6339	-0.352108471	-0.352108	-0.352108471	1.601336827	1.519649377	1.683024277
6341	-0.352108471	-0.352108	-0.352108471	5.169157826	5.858453188	4.479862463
6342	-0.352108471	-0.352108	-0.352108471	6.785476674	4.988525306	5.88700099
6345	6.519673958	4.1063654	8.932982481	1.05704313	1.305230684	0.808855576
6347	-0.352108471	-0.352108	-0.352108471	7.80272331	7.822192666	7.783253954
6352	9.102898355	10.004243	10.90558765	8.059481688	8.44604255	8.832603413
6361	-0.352108471	-0.352108	-0.352108471	5.412561087	5.421579811	5.403542364
6367	5.554553474	6.5498529	4.559254036	3.828621282	1.14221151	6.515031054
6382	-0.352108471	-0.352108	-0.352108471	5.859617369	6.204657504	5.514577234
6383	-0.352108471	-0.352108	-0.352108471	5.760537073	4.088780129	7.432294017
6385	8.032096138	6.3520539	4.672011589	5.371477666	10.5666899	7.969083781
6386	8.766492414	3.0340923	5.90029238	7.369305842	7.745664983	6.9929467
6389	-0.352108471	-0.352108	-0.352108471	5.93619057	5.774905391	5.855547981
6390	-0.352108471	-0.352108	-0.352108471	4.850843637	8.389598823	6.62022123
6391	-0.352108471	-0.352108	-0.352108471	2.211943219	2.232575625	2.191310813
6392	-0.352108471	-0.352108	-0.352108471	6.56915909	7.587716612	5.550601567
6396	6.020381091	7.4881062	4.552655939	10.87012437	5.008198433	7.9391614
6397	7.579071048	6.0245565	6.801813774	5.571206288	6.705287512	7.839368736
6398	-0.352108471	-0.352108	-0.352108471	6.396868383	8.405319657	4.388417108
6399	7.957496831	7.6942359	7.825866352	7.831636626	7.098774477	6.365912329
6400	-0.352108471	-0.352108	-0.352108471	6.345063428	5.914736653	6.12990004
6402	5.703568624	5.9002924	6.097016137	-0.352108471	-0.352108471	-0.352108471
6404	-0.352108471	-0.352108	-0.352108471	5.782493488	4.072864942	4.927679215
6406	-0.352108471	-0.352108	-0.352108471	4.472823391	0.29961159	2.38621749
6415	-0.352108471	-0.352108	-0.352108471	7.180112689	6.910846784	7.449378594
6416	-0.352108471	-0.352108	-0.352108471	2.675180916	6.238591483	4.456886199
6418	4.615583513	4.8795733	4.351593721	6.897119319	7.112080183	7.327041047
6419	5.901554956	5.7752316	5.648908209	4.51080266	5.327050432	4.918926546
6421	-0.352108471	-0.352108	-0.352108471	7.826576286	8.858569924	6.794582647
6426	-0.352108471	-0.352108	-0.352108471	4.651904177	5.191222747	4.112585606
6427	6.402440428	7.9670544	9.531668276	6.434644758	7.511571734	5.357717782
6428	7.80939326	10.956345	9.382869276	7.304663518	6.823015534	7.786311501
6429	-0.352108471	-0.352108	-0.352108471	6.893972732	6.907463027	6.880482436
6430	-0.352108471	-0.352108	-0.352108471	7.304663518	7.314125625	7.29520141
6431	-0.352108471	-0.352108	-0.352108471	8.537491542	4.93406783	6.735779686
6432	5.217491139	4.8379732	5.597009043	7.262193191	6.945202805	7.579183577
6433	-0.352108471	-0.352108	-0.352108471	5.745981054	6.725861268	6.235921161
6434	8.494162324	6.5566899	4.619217479	3.950006671	8.223651289	6.08682898
6442	5.688622647	5.7155573	5.661687951	-0.352108471	-0.352108471	-0.352108471

6443	10.15175113	7.405643	4.659534949	3.009194439	4.232958793	3.621076616
6446	9.462682972	3.7183336	6.590508304	2.547787516	5.169157826	7.790528135
6448	-0.352108471	-0.352108	-0.352108471	6.56347761	7.36450578	6.963991695
6449	8.162356099	8.2580066	8.066705567	3.244814433	2.252618212	1.26042199
6451	7.503722725	6.7717321	7.137727412	7.030579874	5.763156891	6.396868383
6452	7.201907578	6.8152071	7.588608089	4.637194169	4.843308497	4.43107984
6453	6.798710977	6.0510463	7.546375659	-0.352108471	-0.352108471	-0.352108471
6455	6.458591234	7.6305046	8.802417916	7.092186231	7.512705237	7.933224243
6457	1.383520155	-0.352108	3.119148782	1.939746065	1.939746065	1.939746065
6464	6.03798158	4.25734	7.818623197	5.923493182	8.340144299	3.506842066
6468	-0.352108471	-0.352108	-0.352108471	5.835528372	5.825167992	5.845888751
6472	4.343430394	6.7052875	9.067144629	6.751466056	3.799273699	9.703658413
6473	4.587295336	3.8056644	3.024033369	4.907571138	5.696126146	4.119016129
6482	6.558105999	5.3202784	5.939192221	4.370221164	1.603572484	7.136869844
6483	4.315575532	4.3859368	4.245214285	6.160141861	6.246629235	6.073654487
6489	7.858127548	6.6353895	5.412651472	6.259121291	3.548254689	4.90368799
6491	2.061010194	5.3069514	3.683980801	7.222030251	6.62022123	6.018412209
6497	7.257665948	7.0529463	6.848226624	7.334005289	8.541127394	6.126883184
6498	6.046423137	6.3599531	6.20318811	6.074457874	5.008183678	5.541320776
6499	-0.352108471	-0.352108	-0.352108471	6.51496239	5.535173571	7.49475121
6500	7.305341479	7.3154815	7.29520141	7.152037191	7.168934463	7.135139919
6506	6.912680142	5.7033035	6.307991822	0.939116071	1.437859363	0.440372779
6509	6.801813774	5.9212503	7.682377213	-0.352108471	-0.352108471	-0.352108471
6510	-0.352108471	-0.352108	-0.352108471	7.545863613	7.552297351	7.539429874
6513	6.939871798	6.9529417	6.926801914	7.232000306	7.241236027	7.222764584
6515	7.205085245	7.2534814	7.15668911	0.044132154	-0.352108471	0.440372779
6517	5.80388026	7.1217199	4.48604062	3.374312894	2.786645675	2.198978456
6520	5.428393874	3.6953928	7.161394966	8.42551598	5.30035606	6.86293602
6521	4.796807482	7.0788472	5.937827329	2.334436445	1.549724107	3.119148782
6522	7.755138946	6.8748852	5.994631444	5.506138839	7.699410534	9.892682229
6523	-0.352108471	-0.352108	-0.352108471	4.43906433	2.357098027	0.275131723
6525	9.041914027	6.0451731	7.54354356	8.681463169	6.968687949	5.255912729
6526	6.938491159	6.8383365	6.888413829	-0.352108471	-0.352108471	-0.352108471
6528	7.452155293	8.3106812	6.593629393	3.145322661	4.318824564	1.971820759
6532	5.831379091	6.8119539	7.792528717	7.802296601	2.867479288	5.334887945
6533	6.396868383	7.1802794	5.613457334	-0.352108471	-0.352108471	-0.352108471
6534	6.056053999	8.4985445	3.613563458	-0.352108471	-0.352108471	-0.352108471
6535	4.315575532	4.3859368	4.245214285	6.396868383	6.410585269	6.383151496
6538	1.66201518	3.949821	-0.625790688	2.807155681	9.160317743	5.983736712
6541	6.344459978	6.4492768	6.396868383	8.381648057	5.391572833	6.886610445
6545	5.42902339	6.6749197	6.051971547	-0.352108471	-0.352108471	-0.352108471
6548	5.824288785	5.873346	5.775231583	5.983736712	6.002308672	5.965164752
6559	5.119945056	3.4758362	6.764053941	0.624614178	0.808855576	0.440372779
6560	-0.352108471	-0.352108	-0.352108471	6.019409619	5.121960481	6.916858758
6561	6.842157198	5.629874	4.417590702	5.514960729	3.816069721	7.213851737
6563	5.514960729	6.5536427	4.476278805	-0.352108471	-0.352108471	-0.352108471
6564	5.763260124	5.9611669	6.159073607	4.653683611	3.75223911	2.850794609

6566	5.894790244	6.0908083	6.286826437	-0.352108471	-0.352108471	-0.352108471
6567	-0.352108471	-0.352108	-0.352108471	10.10771028	4.374249176	7.240979727
6572	-0.352108471	-0.352108	-0.352108471	3.585121301	3.615460478	3.554782125
6573	-0.352108471	-0.352108	-0.352108471	2.469725738	2.686833264	2.252618212
6574	-0.352108471	-0.352108	-0.352108471	6.596757815	7.00289198	6.190623651
6575	2.326353688	6.5422756	4.434314669	5.325866904	6.67294274	5.999404822
6576	6.91261797	4.4646273	5.688622647	5.015048397	3.342751494	4.178899946
6579	4.172115449	7.2670145	5.719564994	5.172734797	6.297069475	7.421404152
6585	-0.352108471	-0.352108	-0.352108471	4.191896335	7.40850324	5.800199788
6591	5.678624265	8.030747	5.354685649	3.178899946	3.413887841	1.94391205
6594	-0.352108471	-0.352108	-0.352108471	-0.352108471	4.461215055	2.099323895
6597	-0.352108471	-0.352108	-0.352108471	6.316009143	6.355161685	6.276856601
6598	7.137727412	6.5599619	7.715492972	-0.352108471	-0.352108471	-0.352108471
6599	-0.352108471	-0.352108	-0.352108471	6.949901658	5.739370866	8.16043245
6601	5.467416477	4.8121945	5.139805497	3.119148782	3.119148782	3.119148782
6605	8.067932147	7.2841737	7.676052938	6.087644454	7.922387229	4.25290168
6609	5.017136436	5.6370472	4.397225703	9.288236408	6.749630214	4.21102402
6611	-0.352108471	-0.352108	-0.352108471	9.773578061	7.033174504	4.292770948
6612	-0.352108471	-0.352108	-0.352108471	9.513796011	7.387215402	5.260634793
6613	1.514670715	2.6159134	2.065292044	8.069981388	10.71050328	5.4294595
6617	4.956047109	5.3484471	5.152247113	5.616719353	6.685265521	7.753811688
6618	4.274165465	8.2734991	6.273832259	-0.352108471	-0.352108471	-0.352108471
6624	8.038621034	10.62286	5.454381622	7.763976467	8.62809411	9.492211753
6625	5.90029238	5.8500532	5.950531523	0.948388217	3.556848206	2.252618212
6626	-0.352108471	-0.352108	-0.352108471	7.240216663	4.839086163	6.039651413
6628	11.99680028	8.2448542	4.492908162	7.8740963	8.534135593	9.194174886
6629	5.843161371	5.4493489	5.64625513	6.335196408	6.070328712	6.600064104
6631	-0.352108471	-0.352108	-0.352108471	6.643431516	9.716029238	8.179730377
6632	5.946632141	5.0409343	5.493783215	7.679141104	5.152027228	6.415584166
6633	6.020381091	6.0384535	6.002308672	8.106008367	8.11143708	8.100579655
6634	6.823749242	3.7260705	9.921427983	9.276916367	8.091018256	6.905120145
6635	3.945254192	5.5066049	2.383903483	6.844671015	5.295416918	6.070043966
6636	7.281663147	6.4675091	6.874586128	6.77374078	6.785704032	6.761777527
6637	1.501017585	-0.352108	3.354143641	3.055017344	3.272876937	3.49073653
6641	6.801813774	8.0440346	5.559592905	-0.352108471	-0.352108471	-0.352108471
6642	6.08682898	6.0384535	6.135204448	6.621168045	6.653870824	6.588465267
6643	4.870777317	4.6146226	4.358467959	6.78795931	5.096513741	5.942236525
6645	6.584823174	6.4105853	6.759061079	3.699000565	1.939746065	5.458255064
6646	6.341534873	6.9808439	7.62015295	5.638163399	6.490302786	7.342442172
6647	5.688622647	7.4091813	3.968064	7.956236881	9.787541769	6.124931994
6648	-0.352108471	-0.352108	-0.352108471	5.947347959	5.723485516	5.499623073
6649	5.354685649	5.3835036	5.325867653	6.368251906	6.31523113	6.421272681
6650	-0.352108471	-0.352108	-0.352108471	7.21075981	10.51385674	3.907662882
6651	-0.352108471	-0.352108	-0.352108471	5.608262756	5.903760504	6.199258253
6659	5.183745457	6.0665761	5.625160771	-0.352108471	-0.352108471	-0.352108471
6664	4.257389521	5.9391922	7.620994922	0.044132154	0.440372779	-0.352108471
6666	-0.352108471	-0.352108	-0.352108471	4.332084414	6.468151867	8.604219321

6667	-0.352108471	-0.352108	-0.352108471	2.755108764	7.981729883	5.368419324
6670	4.717795902	7.1211232	5.919459543	4.726871378	5.26418547	4.995528424
6671	7.09830115	7.2347133	6.961889018	7.500987084	4.746747472	6.123867278
6674	5.800199788	5.2814525	6.318947122	-0.352108471	-0.352108471	-0.352108471
6675	-0.352108471	-0.352108	-0.352108471	5.575446642	6.599842267	6.087644454
6678	9.31376821	11.659664	10.48671632	11.34502642	9.902487785	10.6237571
6683	6.87773092	5.456102	8.299359815	6.185612433	7.217970939	5.153253928
6689	6.208927462	6.3425287	6.476129899	7.199478037	7.75226244	6.646693634
6692	5.681727979	7.0797459	4.283710045	-0.352108471	-0.352108471	-0.352108471
6693	6.387890954	7.7662254	9.144559764	5.789597744	5.054280952	6.524914536
6694	2.495396186	4.7929677	7.090539263	1.779958785	2.934469601	4.088980418
6695	5.824288785	6.7710897	4.877487911	6.165870606	5.346602799	6.985138413
6709	-0.352108471	-0.352108	-0.352108471	7.859529901	5.47278451	10.24627529
6710	6.567543357	4.6827782	5.625160771	-0.352108471	-0.352108471	-0.352108471
6711	-0.352108471	-0.352108	-0.352108471	7.63947808	5.284974356	2.930470632
6712	-0.352108471	-0.352108	-0.352108471	6.276080792	5.698804213	6.853357372
6713	-0.352108471	-0.352108	-0.352108471	5.246198282	7.818685685	6.532441983
6715	9.427849947	4.3489777	6.888413829	4.80208491	1.742933089	7.861236731
6717	5.354685649	5.3835036	5.325867653	6.149337851	6.174080069	6.124595632
6718	3.752553593	6.581932	5.167242809	3.399021821	1.731739118	5.066304524
6720	3.127737875	3.5255613	2.729914416	5.337471912	4.983522488	5.1604972
6721	-0.352108471	-0.352108	-0.352108471	8.910178217	5.511341403	7.21075981
6722	6.640019393	6.6538708	6.626167961	6.47267644	6.497336045	6.448016836
6723	4.861630841	6.6580232	8.454415574	7.990302333	8.568299261	7.412305405
6726	-0.352108471	-0.352108	-0.352108471	6.204656258	6.215464554	6.193847962
6727	4.973433486	7.6439409	10.31444825	9.870228329	8.365982707	6.861737086
6728	-0.352108471	-0.352108	-0.352108471	5.147851643	5.539970986	5.343911315
6729	-0.352108471	-0.352108	-0.352108471	6.250902217	7.140810338	8.030718458
6730	-0.352108471	-0.352108	-0.352108471	7.895050693	7.903541863	7.886559523
6732	2.618057952	1.763278	3.472837931	5.859617369	6.593651383	5.125583354
6733	-0.352108471	-0.352108	-0.352108471	7.405724394	4.313510344	5.859617369
6734	8.319417943	5.7884526	7.05393529	7.877942624	6.383389221	9.372496027
6737	-0.352108471	-0.352108	-0.352108471	7.331272742	7.35252903	7.310016454
6742	-0.352108471	-0.352108	-0.352108471	7.359412085	5.817096042	6.588254064
6744	4.957624072	5.689454	4.225794122	8.809910093	3.398948843	6.104429468
6745	8.869688135	6.8861366	4.902585161	5.213324008	4.342448827	6.084199188
6746	5.537725397	5.8242888	6.110852173	7.738544772	5.480541275	6.609543024
6747	7.509824613	9.1458822	8.327853397	9.073348878	10.73002222	7.416675538
6748	8.740118719	7.7710199	8.255569322	8.558088434	7.620441317	6.6827942
6749	-0.352108471	-0.352108	-0.352108471	1.508345913	4.416402217	2.962374065
6751	-0.352108471	-0.352108	-0.352108471	7.637943112	7.557875189	7.718011034
6752	5.660837638	4.3778353	6.943839959	3.061037829	4.399624403	1.722451256
6753	6.167582836	6.1999612	6.135204448	-0.352108471	-0.352108471	-0.352108471
6760	6.510289667	7.6505815	7.080435576	8.479827407	7.092103815	5.704380222
6764	-0.352108471	-0.352108	-0.352108471	7.584228848	7.706046291	7.64513757
6767	6.477002382	4.5653797	5.521191046	-0.352108471	-0.352108471	-0.352108471
6770	4.648905722	5.3028288	5.956751783	1.835742592	1.939746065	1.731739118

6772	4.977314285	6.2738323	7.570350233	7.209478834	6.81199092	7.606966749
6773	6.703072347	6.3083327	5.913593129	7.316245125	6.63791606	7.994574191
6774	8.390162921	7.4999152	6.609667386	0.206368668	1.242811212	0.72458994
6776	-0.352108471	-0.352108	-0.352108471	5.803338431	7.028519519	6.415928975
6778	5.55876787	7.1052417	4.012294043	5.946951326	8.407112158	7.177031742
6780	5.078585001	6.8745861	8.670587254	0.044132154	-0.352108471	0.440372779
6781	7.730300925	6.981074	8.479527836	6.537951281	7.18048333	6.859217306
6782	4.649979619	7.0425631	2.257396117	10.34088656	2.130955762	6.235921161
6786	7.270880449	4.6897876	5.980334028	2.218009503	4.522024694	6.826039885
6787	2.937735333	5.6934157	4.315575532	5.468948874	-0.214507862	2.627220506
6789	8.549769548	6.4542211	4.358672625	6.869001299	7.149537332	6.588465267
6790	-0.352108471	-0.352108	-0.352108471	2.570858726	5.458255064	8.345651402
6793	6.735779686	6.7317815	6.739777864	7.224139774	8.294990479	6.153289069
6794	-0.352108471	-0.352108	-0.352108471	3.298455116	7.039860535	5.169157826
6804	5.339588368	5.8242888	6.308989201	-0.352108471	-0.352108471	-0.352108471
6809	4.315575532	6.0679574	2.563193654	6.100160853	5.264042908	4.427924963
6810	-0.352108471	-0.352108	-0.352108471	7.013360586	7.028614492	6.998106681
6811	5.16476681	1.0907089	3.127737875	1.249691864	2.211943219	3.174194573
6812	6.537383725	6.712035	6.362732455	4.351814282	8.841701349	6.596757815
6813	-0.352108471	-0.352108	-0.352108471	4.889603357	6.532441983	8.175280609
6815	7.434535565	6.5310032	5.627470797	7.44507053	5.863843461	6.654456996
6822	5.992801103	6.6924259	7.392050751	2.387802959	2.012146151	2.763459768
6827	-0.352108471	-0.352108	-0.352108471	5.789762726	6.282138603	5.297386849
6829	7.249813015	6.5017089	7.997917169	-0.352108471	-0.352108471	-0.352108471
6830	4.850359235	7.1904029	6.020381091	6.235921161	5.626334218	6.845508104
6832	2.111839558	7.9224333	5.017136436	-0.352108471	-0.352108471	-0.352108471
6835	-0.352108471	-0.352108	-0.352108471	4.869552853	6.32428113	5.596916991
6836	5.016503495	5.3862141	5.201358809	7.857072167	7.413298444	8.300845891
6837	-0.352108471	-0.352108	-0.352108471	3.11804817	6.038453511	8.958858852
6838	5.718459767	7.9293044	3.507615128	-0.352108471	-0.352108471	-0.352108471
6839	6.689537938	4.2920309	9.087044965	-0.352108471	-0.352108471	-0.352108471
6843	5.463922025	5.5293114	5.398532684	-0.352108471	-0.352108471	-0.352108471
6844	-0.352108471	-0.352108	-0.352108471	6.588254064	6.617035395	6.559472732
6856	-0.352108471	-0.352108	-0.352108471	4.021644534	5.284974356	6.548304179
6860	-0.352108471	-0.352108	-0.352108471	7.554789051	7.218748868	6.882708686
6867	-0.352108471	-0.352108	-0.352108471	6.13425979	6.957641788	6.545950789
6869	6.792815994	8.5320824	7.662449217	-0.352108471	-0.352108471	-0.352108471
6873	6.357423773	6.0908083	5.824192908	-0.352108471	-0.352108471	-0.352108471
6874	-0.352108471	-0.352108	-0.352108471	6.676981771	5.64354012	4.610098468
6875	-0.352108471	-0.352108	-0.352108471	5.017136436	5.407946464	4.626326407
6876	9.878330788	12.15879	11.01856029	11.44212027	11.82947108	12.2168219
6878	-0.352108471	-0.352108	-0.352108471	9.608918664	7.374725876	5.140533088
6879	-0.352108471	-0.352108	-0.352108471	7.207985984	8.682110645	5.733861322
6880	6.463674915	3.1920633	9.73528654	4.526657632	6.165870606	7.80508358
6881	3.884316644	0.7435668	2.313941724	6.919435259	6.803366569	7.035503949
6882	5.962279012	3.7639149	4.863096971	6.113115027	6.284083537	5.942146517
6885	-0.352108471	-0.352108	-0.352108471	5.661611801	6.59818828	6.12990004

6888	9.847085445	7.3089428	8.578014108	7.683897278	8.077012352	7.290782205
6890	6.16332568	6.6073775	7.051429238	6.233692038	5.733781386	5.983736712
6892	6.780213449	7.681292	5.879134873	6.329244956	5.996241254	5.663237552
6895	-0.352108471	-0.352108	-0.352108471	5.453492919	4.401865511	4.927679215
6897	7.217317616	7.2305473	7.204087928	2.211943219	2.232575625	2.191310813
6898	5.698028929	4.6079329	5.152980918	1.864658285	1.731739118	1.997577452
6902	5.217491139	5.2437595	5.191222747	7.942419631	7.951299987	7.933539276
6904	-0.352108471	-0.352108	-0.352108471	3.964889143	3.628807669	3.292726194
6905	6.967511245	10.331026	3.603996662	-0.352108471	-0.352108471	-0.352108471
6907	-0.352108471	-0.352108	-0.352108471	5.052267685	5.124543452	4.979991919
6909	6.288510867	3.9393654	8.637656348	3.864327241	3.622938259	3.74363275
6917	-0.352108471	-0.352108	-0.352108471	7.347728623	4.927679215	2.507629806
6919	-0.352108471	-0.352108	-0.352108471	2.124237496	2.147539136	2.100935855
6921	6.214089997	5.1764144	7.251765584	5.325716078	5.336173319	5.315258837
6923	6.499772832	6.4354046	6.371036311	6.713235695	8.758017437	7.735626566
6924	-0.352108471	-0.352108	-0.352108471	6.434644758	6.618106005	6.251183512
6926	-0.352108471	-0.352108	-0.352108471	8.513959953	8.527118113	8.500801793
6929	2.201036472	5.2437595	3.754181416	-0.352108471	-0.352108471	-0.352108471
6935	6.121528769	3.3840954	8.858962183	-0.352108471	-0.352108471	-0.352108471
6938	8.263529236	4.6638206	6.463674915	-0.352108471	-0.352108471	-0.352108471
6942	-0.352108471	-0.352108	-0.352108471	7.74246206	5.593839678	6.668150869
6943	-0.352108471	-0.352108	-0.352108471	5.760537073	5.012886332	6.508187813
6945	-0.352108471	-0.352108	-0.352108471	1.631676003	3.615460478	-0.352108471
6949	-0.352108471	-0.352108	-0.352108471	4.829993231	8.642320562	6.736156896
6950	4.213150349	5.6886226	7.164094945	6.86659232	9.277284275	8.071938297
6975	9.614061112	4.588921	7.101491044	-0.352108471	-0.352108471	-0.352108471
6988	6.64166369	5.703893	7.579434344	4.732462344	5.809232281	6.886002219
6992	-0.352108471	-0.352108	-0.352108471	5.903760504	6.621926089	5.185594919
6993	5.192934229	4.8413386	5.017136436	5.07824431	7.572376051	6.32531018
6996	-0.352108471	-0.352108	-0.352108471	6.204656258	6.215464554	6.193847962
6997	5.624064457	5.054281	6.193847962	3.883361274	5.665786693	2.100935855
6998	2.280956994	-0.921828	5.483742177	0.849500091	2.051108653	-0.352108471
7001	4.178899946	5.6927553	2.66504463	8.232913146	7.002290573	9.463535719
7003	-0.352108471	-0.352108	-0.352108471	7.70317734	9.159777686	6.246576995
7004	5.671658532	6.4525073	7.233356065	-0.352108471	-0.352108471	-0.352108471
7005	-0.352108471	-0.352108	-0.352108471	5.296001127	5.718459767	6.140918407
7007	6.219444057	5.9391922	5.658940386	-0.352108471	-0.352108471	-0.352108471
7009	7.825866352	7.8309189	7.82081384	6.665170885	11.88917304	9.277171965
7011	7.308970622	6.6645531	7.953388098	6.599329815	8.087512342	7.343421079
7013	7.307187158	6.6212707	5.935354281	2.020264194	2.232575625	1.807952763
7014	4.262378	6.0421162	5.152247113	4.792646314	4.124295122	5.460997507
7016	-0.352108471	-0.352108	-0.352108471	2.686833264	2.686833264	2.686833264
7020	-0.352108471	-0.352108	-0.352108471	9.989644737	5.261530125	7.625587431
7025	3.127737875	3.136327	3.119148782	2.169424975	2.191310813	2.147539136
7026	4.077393428	5.5072139	4.792303669	7.188191238	7.232000306	7.275809374
7027	1.210463582	1.3921092	1.573754916	5.400338784	6.022050925	6.643763066
7029	3.796320559	4.1176964	4.439072308	6.496486588	8.162566716	4.830406459

7030	6.184820943	7.1134517	5.25619023	7.757756268	6.949744831	7.353750549
7035	-0.352108471	-0.352108	-0.352108471	4.08236011	4.245214285	3.919505936
7037	8.025076756	2.9624897	5.493783215	-0.352108471	-0.352108471	-0.352108471
7038	6.241909143	6.4453648	6.038453511	-0.352108471	-0.352108471	-0.352108471
7040	7.21196432	8.6179819	5.80594671	6.489024611	7.64513757	8.801250529
7041	7.99158635	5.8881572	6.939871798	7.079987565	8.035014128	8.99004069
7042	-0.352108471	-0.352108	-0.352108471	8.003763827	4.827404505	6.415584166
7044	-0.352108471	-0.352108	-0.352108471	3.119148782	3.119148782	3.119148782
7046	-0.352108471	-0.352108	-0.352108471	6.12990004	3.548149212	8.711650868
7048	-0.352108471	-0.352108	-0.352108471	5.760537073	5.775231583	5.745842563
7049	7.373509403	5.9065294	6.640019393	0.044132154	0.440372779	-0.352108471
7050	5.152247113	5.1799508	5.124543452	-0.352108471	-0.352108471	-0.352108471
7052	10.36893741	10.844273	9.893601362	9.020323195	9.387948573	9.204135884
7054	-0.352108471	-0.352108	-0.352108471	1.783698732	3.919505936	-0.352108471
7056	-0.352108471	-0.352108	-0.352108471	7.660585748	4.514703161	6.087644454
7057	9.964021306	12.602158	7.325884788	11.88604951	10.61082144	11.24843547
7058	5.648189858	9.935878	7.792033924	9.892302962	6.971306167	8.431804565
7059	6.433318728	6.117696	6.74894142	-0.352108471	-0.352108471	-0.352108471
7068	1.719878445	3.1277379	4.535597306	2.274515706	0.888730781	3.660300631
7070	10.03637912	8.5003621	9.268370591	9.278239352	10.1743403	8.382138406
7071	-0.352108471	-0.352108	-0.352108471	8.74723766	4.122051856	6.434644758
7073	5.217491139	5.7824372	4.652545128	7.622315458	5.845489766	9.39914115
7076	8.043505415	8.3876167	7.699394096	9.121051672	7.427648029	5.734244386
7077	7.503741249	7.1425377	7.864944834	9.425667153	9.017159113	9.834175193
7078	7.286324647	8.3380415	6.23460779	8.25743723	9.381522245	7.133352214
7083	7.15770761	7.1689345	7.146480757	-0.352108471	-0.352108471	-0.352108471
7084	5.343125955	3.1277379	0.912349796	7.139330909	7.324669702	7.232000306
7086	8.001125914	9.4179537	8.709539825	8.607193972	8.998058326	9.388922679
7088	-0.352108471	-0.352108	-0.352108471	5.745394463	6.549001936	4.94178699
7093	-0.352108471	-0.352108	-0.352108471	0.793818797	-0.352108471	1.939746065
7094	8.690990141	8.3748765	8.53293333	9.22299889	8.932767957	8.642537024
7095	3.895530886	4.671913	3.119148782	6.786143201	8.54738112	5.024905282
7099	-0.352108471	-0.352108	-0.352108471	6.079602095	4.80208491	3.524567725
7106	5.939192221	5.9574518	5.920932683	5.91274284	10.36126965	8.137006247
7109	-0.352108471	-0.352108	-0.352108471	6.396868383	6.083197802	6.710538963
7110	3.319236702	5.3208399	7.322443168	6.336614961	7.155133943	5.518095979
7112	4.260537128	6.189816	8.119094937	5.034906589	6.515031054	3.554782125
7114	11.50148523	10.947952	12.05501867	14.46183784	12.28492837	10.10801891
7122	6.352053864	6.3831515	6.320956231	-0.352108471	-0.352108471	-0.352108471
7123	-0.352108471	-0.352108	-0.352108471	5.284974356	5.315258837	5.254689875
7126	-0.352108471	-0.352108	-0.352108471	5.382055296	6.434644758	7.48723422
7127	7.203164038	5.8242888	4.445413531	-0.352108471	-0.352108471	-0.352108471
7132	4.315575532	4.3859368	4.245214285	7.396868934	7.407655215	7.386082653
7133	4.528924884	4.3210125	4.73683722	7.556440236	6.469790712	5.383141189
7135	6.933588664	4.7602952	9.10688216	6.281677474	6.522035092	6.041319856
7143	2.201036472	-0.352108	4.754181416	-0.352108471	-0.352108471	-0.352108471
7145	6.868366512	5.9002924	4.932218249	6.776636297	7.585451881	8.394267464

7150	5.980334028	6.7238948	5.236773301	2.049826567	2.211943219	2.374059871
7153	8.230657635	6.6400194	5.04938115	6.450302837	6.479332994	6.421272681
7155	3.515141236	3.6761388	3.354143641	-0.352108471	-0.352108471	-0.352108471
7156	6.646597015	5.0110999	5.828848464	6.448016836	6.840089017	6.055944654
7157	4.905208921	4.8304259	4.079991919	7.51840843	7.311706859	7.105005287
7158	-0.352108471	-0.352108	-0.352108471	7.57445379	5.017633941	6.296043865
7159	5.862048362	5.7285791	5.59510977	-0.352108471	-0.352108471	-0.352108471
7162	8.234191881	6.056054	3.877916117	4.155131623	9.598027907	6.876579765
7167	7.665742317	7.6490111	7.682473564	9.910721168	6.75814483	8.334432999
7168	7.042523083	7.6279541	6.45709211	7.426371554	8.885379694	8.155875624
7169	10.03322986	11.061648	10.54743916	10.90800786	9.069536152	12.74647957
7170	-0.352108471	-0.352108	-0.352108471	6.500182882	7.280233065	8.060283248
7171	7.426398055	7.3674502	7.308502298	6.991162987	8.43340695	7.712284969
7172	6.73526938	7.2641212	7.792973081	4.293517576	6.609965635	5.451741606
7175	-0.352108471	-0.352108	-0.352108471	4.764087889	7.738419133	6.251253511
7178	10.20779186	8.738004	7.268216151	8.916176357	10.74883997	12.58150358
7184	8.216597005	10.367775	6.065418822	12.89729099	10.01238748	7.127483981
7186	8.419901028	6.573969	4.728036971	-0.352108471	-0.352108471	-0.352108471
7187	5.493783215	4.9246879	6.062878518	0.440372779	0.440372779	0.440372779
7188	-0.352108471	-0.352108	-0.352108471	3.287523807	4.843308497	1.731739118
7189	3.437881108	5.8177376	8.197594134	7.532001379	5.511864271	3.491727164
7203	6.552587837	6.6073775	6.662167081	7.071686331	7.220841083	7.369995835
7204	5.626442006	5.1758216	6.077062405	7.05069004	6.31523113	5.579772221
7205	7.052946286	8.0727896	6.033102994	8.077831649	8.735855922	7.419807377
7225	-0.352108471	-0.352108	-0.352108471	6.059042783	6.296043865	6.533044948
7227	-0.352108471	-0.352108	-0.352108471	5.663737322	5.665786693	5.661687951
7247	5.908363377	5.980334	6.05230468	-0.352108471	-0.352108471	-0.352108471
7248	-0.352108471	-0.352108	-0.352108471	6.266367194	7.501435361	5.031299027
7249	-0.352108471	-0.352108	-0.352108471	5.419832838	7.649062677	9.878292516
7251	-0.352108471	-0.352108	-0.352108471	4.526097092	5.169157826	5.812218559
7257	6.230072615	6.384557	6.307314817	6.720370627	6.093637723	7.347103531
7259	8.258638281	5.4798047	11.03747187	6.714644217	9.347808123	8.03122617
7260	6.612891896	6.573969	6.535046103	2.049256654	2.100935855	1.997577452
7262	8.181783015	8.948124	8.564953525	8.59701368	8.051529305	8.324271493
7263	-0.352108471	-0.352108	-0.352108471	3.465863469	5.85425191	4.66005769
7264	8.235744582	4.9790103	6.607377459	4.264528015	7.542992993	5.903760504
7265	-0.352108471	-0.352108	-0.352108471	6.573968999	6.728212604	6.419725395
7266	-0.352108471	-0.352108	-0.352108471	6.840114058	9.619491292	4.060736825
7267	8.198892706	7.0911954	5.983498184	8.872444598	6.454226043	7.66333532
7273	6.618332762	7.935824	9.253315231	6.861182604	6.611309999	7.111055209
7275	7.023635522	5.0171267	6.020381091	-0.352108471	-0.352108471	-0.352108471
7277	-0.352108471	-0.352108	-0.352108471	7.412836985	6.548941201	5.685045417
7280	6.449091275	5.71952	7.178662578	5.115552387	6.837975653	8.560398919
7283	-0.352108471	-0.352108	-0.352108471	6.32531018	5.186438854	7.464181507
7284	6.183964016	6.7115627	5.656365381	7.264940393	7.974587308	8.684234223
7286	6.930683186	6.1928549	7.668511469	6.705512608	6.002129064	7.408896153
7289	7.344003864	7.352529	7.335478698	6.810957895	6.841511354	6.780404436

7292	8.936613718	0.9574007	4.947007189	-0.352108471	-0.352108471	-0.352108471
7294	-0.352108471	-0.352108	-0.352108471	5.277395142	5.923493182	6.569591222
7295	7.052459646	6.9265588	6.800657921	7.961080764	8.370710556	8.780340347
7296	7.425791085	6.7871341	6.14847706	9.907283014	9.3664905	9.636886757
7298	4.966092901	6.1215288	7.276964637	6.095329071	8.942912819	7.519120945
7311	7.368619468	7.2800943	7.191569218	8.693981313	8.767224449	8.620738177
7314	8.446342835	7.4955332	7.970938036	8.208067203	9.264330737	8.73619897
7316	8.553684447	5.1773389	11.93002997	9.241418998	8.694420313	9.788417683
7317	6.763107369	6.3180937	5.873079992	9.252167651	9.533149308	9.814130964
7319	-0.352108471	-0.352108	-0.352108471	6.577767658	5.427760889	7.727774428
7320	2.135660902	9.0381515	5.586906204	5.826844382	8.224598207	7.025721295
7321	6.875645566	6.2112364	5.854214789	-0.352108471	-0.352108471	-0.352108471
7322	6.121528769	6.1000409	6.143016668	-0.352108471	-0.352108471	-0.352108471
7323	6.332466669	6.7703105	5.894622816	-0.352108471	-0.352108471	-0.352108471
7325	-0.352108471	-0.352108	-0.352108471	4.989485442	4.330629937	4.66005769
7326	1.270183879	1.3921092	1.514034619	6.479260377	4.979472145	7.979048608
7327	6.294619744	6.003122	6.586117523	8.495226501	7.094346957	5.693467413
7328	9.28884147	8.1415292	6.994217001	7.551136183	7.547185881	7.555086485
7329	-0.352108471	-0.352108	-0.352108471	4.179448717	6.502347686	8.825246654
7332	6.861057494	6.1604088	7.561706215	8.533000693	8.32726194	8.738739445
7334	7.395245316	4.7863714	6.090808341	7.01393143	4.896504602	5.955218016
7335	-0.352108471	-0.352108	-0.352108471	2.594960553	3.626133664	4.657306774
7336	-0.352108471	-0.352108	-0.352108471	7.930567891	4.996781939	6.463674915
7341	-0.352108471	-0.352108	-0.352108471	7.987794345	5.800151118	6.893972732
7343	7.098170024	4.0029292	5.550549618	5.097937396	5.800022145	5.44897977
7345	6.815431805	6.816905	6.81837828	10.3208556	6.498307495	8.409581547
7347	5.939192221	5.4151493	6.463235152	-0.352108471	-0.352108471	-0.352108471
7348	7.249349515	5.8722086	8.626490441	6.841075177	6.115155073	6.478115125
7351	-0.352108471	-0.352108	-0.352108471	2.221964512	2.252618212	2.191310813
7353	-0.352108471	-0.352108	-0.352108471	6.564571166	7.724029065	7.144300116
7358	8.621879677	3.4540835	6.03798158	6.513676419	5.541007941	4.568339463
7360	-0.352108471	-0.352108	-0.352108471	6.800715119	8.837669995	4.763760243
7363	6.339543069	5.9200251	6.759061079	1.158617076	0.440372779	1.876861374
7366	2.370418305	6.9221181	4.64626819	5.519980446	1.796938968	-1.92610251
7368	3.064174529	8.7364102	5.90029238	-0.352108471	-0.352108471	-0.352108471
7375	4.534386	6.1320496	7.729713276	3.80159353	3.422845765	4.180341295
7376	4.728185378	7.6077482	6.167966808	7.247844574	6.544730233	7.950958916
7378	4.381407216	3.9195059	4.843308497	-0.352108471	-0.352108471	-0.352108471
7380	-0.352108471	-0.352108	-0.352108471	3.216713966	2.802945389	2.389176811
7381	5.11786691	7.4889632	6.303415073	5.342108584	9.058528308	7.200318446
7384	7.286736274	8.0381622	7.662449217	9.327273848	8.1697764	7.012278953
7385	5.55876787	5.5882244	5.529311366	5.596916991	5.605609608	5.588224375
7386	7.277956255	6.0703015	6.674128879	6.309768201	6.912001184	7.514234166
7388	6.303461245	3.2548561	9.352066383	5.231758803	1.93776702	3.584762911
7389	-0.352108471	-0.352108	-0.352108471	6.290961884	6.12990004	5.968838196
7390	7.052946286	6.4929079	7.612984637	-0.352108471	-0.352108471	-0.352108471
7391	2.652337612	1.2407682	1.946552907	4.49290523	4.598205038	4.387605422

7392	5.354685649	5.3835036	5.325867653	5.393523004	5.383503645	5.403542364
7398	5.919459543	8.0259638	3.812955333	6.204656258	7.032262505	5.377050011
7399	2.711561556	1.5251106	3.898012544	3.367655385	3.449520541	3.285790229
7402	4.870731848	6.3741369	5.622434355	7.266128842	5.325958889	6.296043865
7403	-0.352108471	-0.352108	-0.352108471	3.141091639	4.977710418	6.814329197
7407	5.858933008	5.7155573	6.002308672	-0.352108471	-0.352108471	-0.352108471
7408	9.723300333	8.6346034	7.545906481	9.382454464	7.559057303	8.470755883
7410	4.142690079	5.1522471	6.161804148	7.184674008	6.159075269	6.671874638
7411	3.429836003	3.3654516	3.397643806	5.188303169	6.32531018	7.462317192
7414	7.782452578	7.8794708	7.830961702	6.973109567	8.426324173	9.879538778
7415	7.239677489	7.5511362	7.862594877	8.452117188	7.802806691	8.12746194
7416	7.930905526	4.8074077	6.369156591	8.989818148	7.910012046	6.830205944
7417	6.979737572	6.9747788	6.977258204	5.198050748	9.758874274	7.478462511
7419	4.094682292	6.3968684	8.699054473	7.711446746	6.061774144	6.886610445
7423	6.640019393	4.9272166	8.352822191	4.369524912	3.763742066	3.15795922
7424	3.127737875	3.136327	3.119148782	6.468151867	6.515031054	6.421272681
7430	7.839605601	8.4384159	9.037226188	4.426152912	5.832719245	4.239285578
7431	8.871947265	11.747888	11.80991768	12.4424511	9.810122031	15.07478016
7434	7.334003235	5.7669298	4.199856278	3.596060105	1.876861374	5.315258837
7442	7.376787373	7.1930265	7.560548258	4.888253807	4.186919329	3.485584851
7444	-0.352108471	-0.352108	-0.352108471	6.577483275	8.38431063	4.770655921
7450	4.889217569	3.7419335	4.315575532	4.256375256	7.711098168	5.983736712
7453	4.809842661	5.9391922	7.068541782	1.968661759	1.997577452	1.939746065
7458	7.415794329	8.8151833	6.016405367	10.56058913	8.768101402	6.975613677
7461	6.112030716	5.7663537	5.939192221	7.014742575	4.362502719	5.688622647
7464	7.08718283	7.0919708	7.082394844	3.472122176	4.843308497	2.100935855
7466	6.685233471	7.9827771	7.334005289	6.529591207	6.590236847	6.650882486
7468	6.78242301	5.6417115	4.501000046	-0.352108471	-0.352108471	-0.352108471
7469	2.481575183	5.3152588	-0.352108471	-0.352108471	-0.352108471	-0.352108471
7474	5.951362351	6.8312892	7.711216064	6.866733042	8.452492173	5.28097391
7477	5.821546955	9.6151286	7.718337775	8.94535663	4.857978377	6.901667503
7486	-0.352108471	-0.352108	-0.352108471	6.214589741	5.362598742	6.021365987
7494	2.539658743	3.715817	3.127737875	4.0073791	6.849654667	9.691930233
7514	7.614452799	8.2505133	6.978392261	7.750727179	4.812640868	6.281684024
7516	9.374892375	5.72738	7.551136183	7.014362816	7.212682058	7.4110013
7518	-0.352108471	-0.352108	-0.352108471	6.204656258	6.777272313	5.632040203
7520	5.779310544	6.4685941	7.157877604	7.192082458	9.077996786	8.135039622
7525	8.304800241	8.1872605	8.422340024	3.169443478	5.086096378	2.002749278
7528	-0.352108471	-0.847104	-0.479940479	1.411143941	4.967105233	8.523066524
7529	5.939192221	6.7638029	5.11458153	7.601051706	7.974870977	7.227232435
7531	5.002635162	6.2125362	7.422437256	8.447789726	4.711260295	12.18431916
7532	-0.352108471	-0.352108	-0.352108471	6.544687493	7.374725876	8.204764258
7533	7.151993326	7.0273758	6.90275822	6.920260803	7.872600424	8.824940044
7534	8.684506271	8.521769	8.359031806	8.832507181	11.29322732	6.371787037
7536	5.688622647	5.7155573	5.661687951	7.700335208	7.692669739	7.708000677
7538	6.888413829	6.2584133	7.518414344	6.365745775	5.233567424	4.101389072
7542	5.718459767	5.7752316	5.661687951	5.983736712	6.002308672	5.965164752

7543	6.23156194	6.1079622	6.355161685	6.560527001	6.532588736	6.588465267
7547	-0.352108471	-0.352108	-0.352108471	5.821410838	5.292050137	6.350771539
7549	7.936330313	10.147478	5.725182209	6.94805115	7.14280972	7.33756829
7555	10.59487497	6.8846031	8.739739036	8.21671462	8.137575638	8.058436655
7556	8.653946329	2.9464532	5.800199788	-0.352108471	-0.352108471	-0.352108471
7561	-0.352108471	-0.352108	-0.352108471	2.169424975	-0.161483164	4.500333114
7562	6.315832277	4.9894341	7.642230459	1.433785054	1.74784442	2.061903786
7581	2.711561556	5.7752316	-0.352108471	0.440372779	0.440372779	0.440372779
7582	5.057341212	6.2145375	5.635939335	1.644875323	2.147539136	1.14221151
7586	6.091633494	7.5343218	8.977010038	9.169987063	6.522158346	3.87432963
7587	7.299617088	4.3708006	5.835208855	1.290035436	1.437859363	1.14221151
7592	-0.352108471	-0.352108	-0.352108471	5.983736712	4.379078243	7.588395181
7594	6.70081996	6.8767254	6.524914536	3.776897663	1.807952763	5.745842563
7596	6.167582836	7.2067514	5.128414275	-0.352108471	-0.352108471	-0.352108471
7620	8.569387437	9.8410775	7.297697327	5.399644331	11.25814164	8.328892986
7621	5.639942626	5.1526923	5.396317463	2.743924656	3.763444409	4.782964162
7626	4.322753636	4.8786655	5.434577321	-0.352108471	-0.352108471	-0.352108471
7629	-0.352108471	-0.352108	-0.352108471	6.663549981	4.297639083	1.931728184
7634	4.696178549	7.0297028	5.862940659	4.282217916	6.965910998	5.624064457
7637	3.067324044	4.8540291	3.960676558	5.423367233	5.125330896	5.274349064
7644	6.918309499	7.941723	5.894896006	7.677203728	3.273687448	5.475445588
7652	6.261564247	6.1679668	6.355161685	2.130680146	3.119148782	1.14221151
7692	-0.352108471	-0.352108	-0.352108471	5.059845876	6.907627548	5.983736712
7695	3.137029745	-0.352108	6.626167961	-0.352108471	-0.352108471	-0.352108471
7706	-0.352108471	-0.352108	-0.352108471	7.240736817	6.497182024	5.753627231
7716	7.661201642	8.3951328	6.927270497	6.769641641	7.835395748	5.703887534
7726	6.151585628	4.6721097	7.631061543	5.595674035	9.75048434	7.673079188
7727	-0.352108471	-0.352108	-0.352108471	8.05826446	6.769641641	5.481018822
7728	6.471312114	5.5389635	7.403660712	2.653660277	4.016045771	5.378431265
7739	-0.352108471	-0.352108	-0.352108471	5.284974356	7.077174255	3.492774457
7741	8.472062733	6.5905083	4.708953875	-0.352108471	-0.352108471	-0.352108471
7745	2.681724436	1.9642013	3.399247549	7.10995036	5.051993053	2.994035746
7750	6.953114539	6.3269242	6.640019393	-0.352108471	-0.352108471	-0.352108471
7753	6.502263028	6.1919279	6.812598199	1.371545024	1.437859363	1.305230684
7754	6.596957353	3.4009836	4.998970473	0.624614178	0.440372779	0.808855576
7756	-0.352108471	-0.352108	-0.352108471	6.836610872	5.458255064	4.079899256
7760	4.156182401	4.2016175	4.178899946	6.359270363	6.548307945	6.170232782
7762	-0.352108471	-0.352108	-0.352108471	7.389650362	4.722457636	6.056053999
7763	6.948800955	7.1846284	6.712973472	9.199084805	5.782227182	7.490655993
7767	3.409093945	5.3205632	7.232032546	-0.352108471	-0.352108471	-0.352108471
7775	-0.352108471	-0.352108	-0.352108471	4.954669547	5.095423988	5.236178429
7782	4.064017356	5.1171159	6.170214371	0.872801732	0.440372779	1.305230684
7784	5.59790927	5.3152588	5.880559702	-0.352108471	-0.352108471	-0.352108471
7791	9.306885836	8.305921	10.30785065	9.985337343	11.93112203	8.039552661
7799	1.783698732	-0.352108	3.919505936	4.97366118	5.529311366	6.084961552
7803	7.485956147	7.8380562	7.133856128	-0.352108471	-0.352108471	-0.352108471
7805	-0.352108471	-0.352108	-0.352108471	4.80208491	5.050021546	4.554148274

7812	-0.352108471	-0.352108	-0.352108471	7.69442279	8.147174035	8.599925279
7813	4.889218131	6.9235485	5.906383311	5.988664101	4.889986617	7.087341585
7818	5.493783215	6.4543531	4.533213306	4.691916222	5.410759683	3.97307276
7827	5.522228925	5.7280926	5.625160771	-0.352108471	-0.352108471	-0.352108471
7837	8.233257672	6.0421972	7.137727412	8.012044455	6.886095759	5.760147063
7840	8.006374658	5.0894122	2.172449746	-0.352108471	-0.352108471	-0.352108471
7846	-0.352108471	-0.352108	-0.352108471	5.986268786	7.713912149	9.441555512
7849	3.364953967	9.8821279	6.623540933	0.440372779	0.440372779	0.440372779
7851	7.048158978	6.6168926	6.185626278	10.6534243	3.652638077	7.153031188
7852	6.488464425	3.0961429	4.792303669	-0.352108471	-0.352108471	-0.352108471
7866	6.788420481	6.2558263	7.321014647	5.898722313	8.975446507	7.43708441
7871	-0.352108471	-0.352108	-0.352108471	4.547696525	6.345275458	8.14285439
7873	5.02423831	7.374953	6.199595632	6.525425359	6.885149665	6.705287512
7874	9.374134794	6.2885109	3.20288694	6.545950789	5.890936224	7.200965353
7879	5.086953883	6.4118503	5.749402114	9.621468609	8.305994833	6.990521057
7884	-0.352108471	-0.352108	-0.352108471	5.661770766	5.785200266	5.723485516
7905	7.251757684	6.4599337	8.043581665	4.819995464	9.404164901	7.112080183
7913	-0.352108471	-0.352108	-0.352108471	5.247084775	7.510362796	9.773640817
7915	6.571079922	6.5532966	6.562188268	2.681798769	2.546625611	2.411452453
7916	7.906194427	6.663797	9.148591903	6.967894694	9.575738644	8.271816669
7917	1.762940593	1.0212779	1.392109249	5.537748484	7.074497347	6.306122915
7918	5.545947536	5.6886226	5.831297758	-0.352108471	-0.352108471	-0.352108471
7922	8.968935914	5.4025961	7.185765998	6.693937107	7.383419948	6.004454266
7936	-0.352108471	-0.352108	-0.352108471	9.017100968	5.837496104	7.427298536
7955	4.811712164	4.4254169	4.618564529	0.228373553	-0.352108471	0.808855576
7965	-0.352108471	-0.352108	-0.352108471	5.712263427	6.324418	5.100108855
7976	8.82249698	7.9996506	7.176804283	5.636571956	4.439548204	5.03806008
7979	-0.352108471	-0.352108	-0.352108471	6.32531018	7.046398208	5.604222153
7982	1.946552907	4.2452143	-0.352108471	2.118278685	2.686833264	1.549724107
7988	4.812830099	5.2849744	5.757118613	-0.352108471	-0.352108471	-0.352108471
7991	9.178857366	3.3052392	6.242048279	7.209478834	5.365330774	9.053626895
8013	5.670355529	6.572702	6.121528769	-0.352108471	-0.352108471	-0.352108471
8022	8.353726476	4.9945313	6.674128879	-0.352108471	-0.352108471	-0.352108471
8027	3.515141236	6.1721123	0.858170134	6.056053999	3.794790437	8.317317561
8029	6.801857078	4.3807541	5.591305588	2.272836381	3.615460478	0.930212283
8030	-0.352108471	-0.352108	-0.352108471	5.809398431	5.859617369	5.909836307
8031	-0.352108471	-0.352108	-0.352108471	3.827292409	5.284974356	6.742656303
8045	-0.352108471	-0.352108	-0.352108471	5.169157826	5.191222747	5.147092904
8050	-0.352108471	-0.352108	-0.352108471	4.806952735	8.837464919	6.822208827
8061	6.955397734	7.6725705	7.313984131	5.533057745	7.15770761	8.782357475
8073	5.089412202	4.7869486	5.391875808	8.031351006	7.329088911	6.626826816
8076	6.466934042	8.3193274	7.393130705	3.156150334	3.500702413	3.845254491
8078	8.871899983	5.499632	7.185765998	7.947813531	8.59861235	9.249411169
8079	5.683083302	5.0894122	4.495741102	9.006475171	8.558877279	8.111279388
8085	7.15770761	4.5389014	9.776513799	5.095423988	1.818892945	8.371955031
8087	7.042523083	7.7422973	6.3427489	5.202626588	5.458255064	5.71388354
8091	5.652271474	8.5448167	2.759726277	1.227667732	1.646479888	0.808855576

8099	-0.352108471	-0.352108	-0.352108471	7.856765125	7.714895119	7.573025113
8110	8.490284424	6.9527073	5.415130253	-0.352108471	-0.352108471	-0.352108471
8125	5.211255078	5.1522471	5.093239148	2.481575183	5.315258837	-0.352108471
8131	7.466207166	6.7844221	8.147992266	5.486076511	5.596916991	5.707757471
8139	5.575838517	5.2809492	5.428393874	-0.352108471	-0.352108471	-0.352108471
8140	8.408533238	7.3777551	6.346977029	7.002712288	9.695935916	8.349324102
8148	5.90029238	6.8892533	4.911331493	7.467246872	6.860007601	8.074486143
8153	5.608777454	6.2671463	6.925515208	6.72865797	6.218762094	7.238553846
8170	5.527638398	4.7559934	6.2992834	-0.352108471	-0.352108471	-0.352108471
8175	3.676426169	3.9349025	3.805664353	6.907463027	9.810731119	4.004194936
8189	6.005735869	8.4977795	7.251757684	8.143484057	4.426487714	11.8604804
8192	6.687972707	8.2448542	9.801735739	2.252618212	2.252618212	2.252618212
8202	-0.352108471	-0.352108	-0.352108471	5.751745665	5.656924845	5.704335255
8204	-0.352108471	-0.352108	-0.352108471	6.633874018	6.469622303	6.55174816
8208	-0.352108471	-0.352108	-0.352108471	6.266367194	6.214565395	6.318168992
8216	5.421815342	6.3034612	7.185107148	-0.352108471	-0.352108471	-0.352108471
8220	5.569205318	5.5913056	5.613405859	-0.352108471	-0.352108471	-0.352108471
8224	8.583527785	9.1878548	9.792181849	8.029024031	6.382429697	4.735835364
8226	-0.352108471	-0.352108	-0.352108471	5.484671023	7.145791238	6.31523113
8228	6.055892079	6.2312245	5.880559702	3.248916248	4.245214285	2.252618212
8233	-0.352108471	-0.352108	-0.352108471	6.696534724	6.56915909	6.441783456
8237	5.345349382	6.1995956	7.053841881	4.069694683	6.660105502	1.479283864
8239	5.697485036	6.2061804	6.714875854	7.728051932	8.100533976	8.47301602
8242	2.601256156	5.7565437	4.178899946	5.615364839	7.97826492	6.796814879
8243	-0.352108471	-0.352108	-0.352108471	1.366179719	7.54759268	4.456886199
8260	3.46119715	6.4636749	9.466152679	7.744466355	7.604813664	7.465160974
8263	1.829978742	2.7365298	3.643080779	5.369332089	5.261950251	5.476713926
8273	-0.352108471	-0.352108	-0.352108471	6.10626679	3.390217934	8.822315646
8288	5.003306707	6.4358233	5.719564994	-0.352108471	-0.352108471	-0.352108471
8289	5.217491139	4.0200646	6.41491772	7.076248058	6.56915909	6.062070121
8291	-0.352108471	-0.352108	-0.352108471	2.47172199	2.077309423	2.274515706
8292	-0.352108471	-0.352108	-0.352108471	5.723485516	5.776232748	5.670738284
8301	3.805664353	4.0053499	3.605978805	7.318035473	8.897018532	8.107527003
8310	2.38621749	-0.352108	5.124543452	2.492032424	5.336173319	-0.352108471
8312	6.984847274	5.4283939	3.871940474	7.570669745	6.091438869	6.831054307
8314	-0.352108471	-0.352108	-0.352108471	4.569595785	5.637296679	6.704997573
8315	-0.352108471	-0.352108	-0.352108471	5.663737322	5.931069616	5.396405027
8318	-0.352108471	-0.352108	-0.352108471	5.939192221	5.958359385	5.920025058
8322	6.053362071	4.5468593	7.559864837	5.086878564	5.345666926	5.604455288
8324	6.613499849	6.349548	6.481523945	-0.352108471	-0.352108471	-0.352108471
8326	-0.352108471	-0.352108	-0.352108471	2.902991023	3.119148782	2.686833264
8332	6.768938711	6.2704092	6.519673958	-0.352108471	-0.352108471	-0.352108471
8336	6.151585628	6.1679668	6.135204448	-0.352108471	-0.352108471	-0.352108471
8394	-0.352108471	-0.352108	-0.352108471	5.834905668	9.602153462	7.718529565
8396	7.564006467	6.6103592	7.08718283	4.365930238	3.919505936	3.473081633
8402	-0.352108471	-0.352108	-0.352108471	0.849500091	-0.352108471	2.051108653
8405	4.967066095	5.6982657	4.235866508	-0.352108471	-0.352108471	-0.352108471

8407	10.0436998	7.6937134	8.868706618	8.496131023	9.35049936	10.2048677
8409	3.702006037	6.2125362	8.723066381	5.815475722	3.942292236	7.688659208
8420	5.270651934	4.3990075	3.527362986	0.289051906	-0.352108471	0.930212283
8425	-0.352108471	-0.352108	-0.352108471	7.323993398	3.736882973	10.91110382
8427	3.829869794	2.425606	3.127737875	4.476918793	5.637296679	6.797674564
8428	-0.352108471	-0.352108	-0.352108471	6.682822443	5.656080167	7.709564718
8436	4.855447759	5.3546856	5.853923539	-0.352108471	-0.352108471	-0.352108471
8438	1.383520155	2.357098	3.119148782	-0.352108471	-0.352108471	-0.352108471
8439	6.890924634	7.0919708	6.689878453	-0.352108471	-0.352108471	-0.352108471
8449	-0.352108471	-0.352108	-0.352108471	2.179939357	1.306251685	3.05362703
8450	6.433318728	6.4453648	6.421272681	6.463674915	6.479332994	6.448016836
8451	5.800199788	5.9243692	5.676030376	4.877471268	9.680009482	7.278740375
8452	7.805419383	5.619404	9.991434722	7.86582773	6.936969129	8.794686331
8454	-0.352108471	-0.352108	-0.352108471	9.03484597	5.603051715	7.318948842
8460	-0.352108471	-0.352108	-0.352108471	8.268076885	5.017136436	1.766195986
8463	-0.352108471	-0.352108	-0.352108471	6.992632695	6.606745784	6.220858873
8471	6.874586128	4.9944242	8.754748025	-0.352108471	-0.352108471	-0.352108471
8477	6.947006362	6.0449945	7.849018203	4.36678506	4.108072315	3.84935957
8479	7.042523083	6.779279	7.30576713	-0.352108471	-0.352108471	-0.352108471
8481	-0.352108471	-0.352108	-0.352108471	3.130821893	3.919505936	4.708189978
8482	-0.352108471	-0.352108	-0.352108471	5.280401645	5.789762726	6.299123807
8483	0.965727592	4.140419	2.553073296	-0.352108471	-0.352108471	-0.352108471
8491	-0.352108471	-0.352108	-0.352108471	2.38621749	2.122666746	2.649768234
8492	2.77514947	3.1345327	2.954841066	-0.352108471	-0.352108471	-0.352108471
8493	4.600268283	5.8369584	7.073648445	0.29941874	3.197122757	1.748270748
8496	6.649748172	7.5749394	8.500130582	5.231429669	5.895191162	6.558952656
8498	5.777785791	6.7422729	6.260029354	6.87306658	6.705113583	7.041019578
8500	2.971767785	5.9002924	8.828816976	-0.352108471	-0.352108471	-0.352108471
8502	-0.352108471	-0.352108	-0.352108471	6.609543024	4.348681051	8.870404996
8516	3.127737875	3.136327	3.119148782	2.903700168	2.252618212	3.554782125
8517	-0.352108471	-0.352108	-0.352108471	1.245740716	0.440372779	2.051108653
8519	7.949286752	7.9102616	7.988311912	2.501526031	-0.352108471	5.355160532
8522	5.928731366	6.395997	6.863262576	3.691912836	6.307268721	8.922624606
8525	6.266052353	6.8818856	6.573968999	6.022050925	4.202080518	7.842021332
8526	7.117674405	5.1544854	3.191296423	3.442948713	5.336173319	1.549724107
8527	5.970796616	5.6109164	5.790856505	-0.352108471	-0.352108471	-0.352108471
8531	6.317834233	3.3638818	9.271786639	6.689327433	4.46264002	5.575983727
8533	-0.352108471	-0.352108	-0.352108471	6.570311461	6.223425304	6.396868383
8534	4.81534536	2.4335672	3.624456288	5.345910491	5.809594275	6.273278059
8535	3.369288587	9.7054789	6.537383725	6.571854611	5.233567424	3.895280236
8537	4.381407216	6.0042448	2.75856961	1.3309757	1.731739118	0.930212283
8539	-0.352108471	-0.352108	-0.352108471	6.596757815	6.617035395	6.576480236
8543	6.020381091	7.5058983	4.534863919	6.513690968	6.019409619	5.525128271
8545	-0.352108471	-0.352108	-0.352108471	5.017136436	5.054280952	4.979991919
8548	5.380082893	6.2420483	7.104013665	-0.352108471	-0.352108471	-0.352108471
8551	7.30087348	5.8021647	6.551519093	6.07919154	5.775231583	6.383151496
8552	3.127737875	3.136327	3.119148782	1.651404068	1.305230684	1.997577452

8553	5.07653385	7.850816	6.463674915	5.51140784	6.976601204	8.441794568
8560	8.450148986	6.8928603	5.335571552	5.740438674	7.70185263	6.721145652
8562	8.244445775	8.2249387	8.23469223	5.718205971	7.911884024	6.815044997
8565	-0.352108471	-0.352108	-0.352108471	8.682742619	7.329061379	8.005901999
8566	6.37650413	8.6508258	7.513664974	6.843135165	4.920273781	8.765996549
8568	-0.352108471	-0.352108	-0.352108471	4.032813514	4.178899946	4.324986377
8569	5.045025943	3.7815658	6.308486129	-0.352108471	-0.352108471	-0.352108471
8570	7.551014561	8.6967105	9.842406356	7.832756024	7.021841047	7.427298536
8572	6.042247545	6.5566899	7.071132258	7.223134165	7.222661978	7.222189791
8573	3.127737875	3.1687987	3.086677097	2.169424975	3.14822909	1.190620859
8574	6.658023207	3.2925403	10.02350609	1.400698343	-0.352108471	3.153505157
8575	-0.352108471	-0.352108	-0.352108471	7.673079188	6.689576901	8.656581474
8576	6.020381091	6.3325422	5.708219959	0.077665561	3.676057187	1.876861374
8577	-0.352108471	-0.352108	-0.352108471	4.736619762	1.75463195	3.245625856
8578	6.051754514	6.6415447	7.231334981	4.379148927	6.566987041	2.191310813
8603	2.711561556	-0.352108	5.775231583	-0.352108471	-0.352108471	-0.352108471
8607	-0.352108471	-0.352108	-0.352108471	3.919505936	3.919505936	3.919505936
8611	6.087530678	5.9532315	6.020381091	-0.352108471	-0.352108471	-0.352108471
8614	8.920617896	8.5161798	8.111741698	7.296097593	8.717991356	10.13988512
8615	2.079909098	8.0246263	5.052267685	6.549114467	6.244622298	6.396868383
8621	-0.352108471	-0.352108	-0.352108471	5.958359385	5.377761049	6.53895772
8622	-0.352108471	-0.352108	-0.352108471	5.017136436	3.348463442	6.68580943
8623	-0.352108471	-0.352108	-0.352108471	6.08682898	7.221919407	4.951738553
8624	-0.352108471	-0.352108	-0.352108471	0.762376451	-0.352108471	1.876861374
8625	5.692087125	6.2685809	5.980334028	2.235698462	2.211943219	2.188187975
8632	5.413510577	4.1218461	2.830181656	0.044132154	-0.352108471	0.440372779
8633	-0.352108471	-0.352108	-0.352108471	4.280868658	6.544253517	5.412561087
8634	5.292949001	4.6531192	5.932778769	6.102961535	7.007811177	7.912660819
8635	3.805664353	3.9195059	3.69182277	5.095423988	5.124543452	5.066304524
8636	-0.352108471	-0.352108	-0.352108471	6.401526885	7.047803158	6.724665022
8639	2.515697587	3.2955425	1.735852699	-0.352108471	-0.352108471	-0.352108471
8642	-0.352108471	-0.352108	-0.352108471	5.939192221	5.443601632	6.434782811
8644	-0.352108471	-0.352108	-0.352108471	2.204376426	-0.352108471	4.760861323
8649	-0.352108471	-0.352108	-0.352108471	3.245625856	1.344198885	5.147052827
8653	4.583433279	4.1125856	5.054280952	4.186919329	3.119148782	5.254689875
8654	-0.352108471	-0.352108	-0.352108471	7.775366346	6.479260377	5.183154407
8655	8.962798351	7.5062708	6.049743181	7.757808357	7.404776674	8.11084004
8658	6.483325563	5.5587679	4.634210177	-0.352108471	-0.352108471	-0.352108471
8661	6.861057494	6.8767254	6.845389604	6.12990004	6.135204448	6.124595632
8662	6.370355426	6.6400194	6.909683359	5.427549346	7.326356364	9.225163383
8663	-0.352108471	-0.352108	-0.352108471	6.27703705	3.391273426	4.834155238
8664	5.872522726	7.7880739	6.830298336	8.433479581	5.939192221	3.444904862
8665	8.576796965	6.7696416	4.962486317	9.028333354	7.277863047	8.1530982
8666	-0.352108471	-0.352108	-0.352108471	6.636273252	9.392370402	3.880176102
8667	6.624807556	6.7871341	6.94946059	8.488193315	7.912102061	9.064284569
8668	7.121141731	7.3154815	6.926801914	6.344555562	6.174080069	6.515031054
8669	8.074241084	5.1074352	6.590838154	4.833742536	7.856808379	6.345275458

8671	-0.352108471	-0.352108	-0.352108471	6.739871389	6.204656258	5.669441127
8672	-0.352108471	-0.352108	-0.352108471	7.46159208	6.577483275	5.69337447
8674	-0.352108471	-0.352108	-0.352108471	-0.800236149	4.560713284	1.880238567
8675	7.009972231	8.9179049	7.963938574	6.344555562	7.985081128	4.704029995
8676	5.3562704	7.7175468	2.994993979	2.072558294	0.815227852	3.329888737
8677	-0.352108471	-0.352108	-0.352108471	4.927679215	3.914731559	5.940626871
8678	7.150486084	6.483324	6.816905042	-0.352108471	-0.352108471	-0.352108471
8682	9.112686581	8.9770872	9.248285985	9.169219801	8.157159713	7.145099625
8683	7.759258846	7.8991344	7.619383264	7.667232662	7.641795584	7.692669739
8687	-0.352108471	-0.352108	-0.352108471	7.098078287	5.494417177	3.890756066
8692	5.927948731	7.0176135	6.472781091	6.641651422	7.315470468	7.989289514
8693	4.16706778	4.9799919	3.354143641	1.916337486	1.731739118	2.100935855
8694	6.44815499	5.4302295	5.939192221	5.712263427	7.486478001	3.938048854
8701	-0.352108471	-0.352108	-0.352108471	6.702366812	9.022567127	4.382166498
8702	6.766005329	6.6445697	6.705287512	5.669631022	4.521216954	5.095423988
8703	-0.352108471	-0.352108	-0.352108471	5.847512348	5.06899778	5.458255064
8704	8.506386372	3.6057216	6.056053999	6.913448327	7.449539283	6.377357371
8718	7.054214915	5.9857307	6.519972795	-0.352108471	-0.352108471	-0.352108471
8720	4.558115529	6.2986722	5.428393874	6.197683273	8.152885412	7.175284342
8721	9.765912892	6.5973455	8.181629182	3.787855241	0.91431724	2.351086241
8722	5.625160771	5.6532998	5.597021732	6.266367194	6.276856601	6.255877787
8724	2.939198617	3.3162771	3.127737875	3.833071076	4.66005769	5.487044303
8727	4.792303669	4.8304259	4.754181416	-0.352108471	-0.352108471	-0.352108471
8728	-0.352108471	-0.352108	-0.352108471	6.51496239	4.448112575	8.581812205
8729	-0.352108471	-0.352108	-0.352108471	5.415665079	6.303569659	5.859617369
8733	-0.352108471	-0.352108	-0.352108471	8.247858438	8.261463396	8.234253481
8735	-0.352108471	-0.352108	-0.352108471	2.912457358	0.848019777	1.880238567
8737	5.698981455	5.876277	6.05357254	6.296043865	7.70388208	4.888205651
8738	-0.352108471	-0.352108	-0.352108471	5.284974356	5.315258837	5.254689875
8740	9.775390841	0.95183	5.363610422	3.477522833	1.807952763	5.147092904
8742	-0.352108471	-0.352108	-0.352108471	2.772468849	1.90830372	1.04413859
8749	5.960830253	5.8001998	5.639569322	-0.352108471	-0.352108471	-0.352108471
8751	6.433318728	6.4453648	6.421272681	7.240174153	7.246938498	7.233409808
8754	2.711561556	4.0019009	1.421222192	9.421598849	4.758553984	7.090076417
8755	5.426639686	7.2382937	6.332466669	-0.352108471	-0.352108471	-0.352108471
8761	3.127737875	3.136327	3.119148782	7.792233337	7.209478834	6.626724331
8763	4.878665478	1.5899128	8.167418132	6.963991695	6.498829008	7.429154382
8764	-0.352108471	-0.352108	-0.352108471	6.003978506	6.002308672	6.005648339
8766	5.927434695	7.7083518	6.817893231	8.529558902	6.564802653	7.547180778
8772	5.217491139	6.4970264	3.937955899	-0.352108471	-0.352108471	-0.352108471
8775	5.261854875	7.4912415	9.720628135	7.76913794	5.777211438	9.761064442
8776	-0.352108471	-0.352108	-0.352108471	5.636331951	6.006489724	5.821410838
8777	-0.352108471	-0.352108	-0.352108471	6.700481766	6.846999794	6.77374078
8780	5.156273414	5.2915926	5.020954247	6.198272066	4.667571989	5.432922028
8786	2.715495103	0.0515452	1.383520155	0.600256943	4.001853132	2.301055037
8793	-0.352108471	-0.352108	-0.352108471	6.390945344	7.395526103	8.400106862
8794	-0.352108471	-0.352108	-0.352108471	5.458255064	5.458255064	5.458255064

8795	5.062989958	5.1158344	5.089412202	3.847846724	8.031147602	5.939497163
8797	2.15990226	4.671913	-0.352108471	1.880238567	-0.352108471	4.112585606
8798	-0.352108471	-0.352108	-0.352108471	5.663737322	4.387216247	6.940258397
8801	-0.352108471	-0.352108	-0.352108471	7.634480443	6.459158017	7.04681923
8802	7.511160936	4.7920103	6.151585628	-0.352108471	-0.352108471	-0.352108471
8803	-0.352108471	-0.352108	-0.352108471	3.744317129	8.332589892	6.038453511
8808	-0.352108471	-0.352108	-0.352108471	6.488333699	5.663737322	4.839140945
8812	-0.352108471	-0.352108	-0.352108471	7.175112333	5.657612942	6.416362638
8814	-0.352108471	-0.352108	-0.352108471	6.496886722	6.532588736	6.461184708
8815	6.57771666	6.8018138	7.025910888	7.15770761	6.74942879	7.565986429
8816	-0.352108471	-0.352108	-0.352108471	6.032678842	6.079429156	6.056053999
8822	-0.352108471	-0.352108	-0.352108471	0.82273449	-0.352108471	1.997577452
8824	4.951513474	6.9569047	5.954209091	9.314173775	5.088170497	7.201172136
8826	2.681724436	5.7155573	-0.352108471	7.837299406	8.255966946	8.046633176
8829	5.785697221	7.2173176	8.64893801	5.708307224	5.55062115	5.865993298
8832	6.673763173	5.1956968	8.151829579	6.981858868	6.585820725	7.37789701
8833	-0.352108471	-0.352108	-0.352108471	7.002061667	7.371089232	6.633034102
8834	2.839240109	3.5151412	4.191042363	5.436344257	7.555463443	9.674582629
8837	-0.352108471	-0.352108	-0.352108471	4.178899946	4.245214285	4.112585606
8843	2.620695344	2.4458255	2.270955716	-0.352108471	-0.352108471	-0.352108471
8844	7.287907295	4.3124923	5.800199788	0.044132154	0.440372779	-0.352108471
8846	-0.352108471	-0.352108	-0.352108471	5.575983727	6.442637652	4.709329801
8847	7.630271838	8.2013461	8.772420315	7.17500483	7.580671038	6.769338621
8848	6.243711799	2.2010365	-1.841638854	-0.352108471	-0.352108471	-0.352108471
8853	-0.352108471	-0.352108	-0.352108471	6.148109327	6.41249299	6.280301159
8861	-0.352108471	-0.352108	-0.352108471	4.788167673	6.89992346	9.011679248
8867	3.326244202	5.9002924	8.474340559	-0.352108471	-0.352108471	-0.352108471
8870	6.6873399	5.8695181	7.505161726	8.937970122	8.753127999	8.568285876
8871	-0.352108471	-0.352108	-0.352108471	5.169157826	7.779483104	2.558832547
8872	-0.352108471	-0.352108	-0.352108471	1.90830372	1.939746065	1.876861374
8874	5.269012491	5.8485232	5.55876787	8.348660622	4.445076143	6.396868383
8877	7.562590964	5.6777465	9.447435442	5.625342717	6.221643648	5.923493182
8878	-0.352108471	-0.352108	-0.352108471	1.933563105	2.245600013	2.557636922
8879	4.583433279	5.3357979	3.831068664	6.177361516	1.079890975	3.628626245
8880	7.145892954	5.6251608	4.104428589	5.663737322	4.931152005	6.396322638
8881	-0.352108471	-0.352108	-0.352108471	6.991222272	7.003831781	6.978612763
8882	-0.352108471	-0.352108	-0.352108471	3.658250427	7.08041375	5.369332089
8883	-0.352108471	-0.352108	-0.352108471	5.750717835	6.296043865	6.841369896
8886	5.829284965	5.9391922	6.049099478	4.890297659	7.28499125	6.087644454
8887	6.831289207	6.8453896	6.817188811	6.636273252	6.662724824	6.609821679
8888	-0.352108471	-0.352108	-0.352108471	2.952408619	8.494562413	5.723485516
8890	-0.352108471	-0.352108	-0.352108471	6.321454008	6.833512543	6.577483275
8891	4.025746598	3.0129081	3.519327359	2.360715852	6.959399527	4.66005769
8892	5.875870905	5.6886226	5.501374389	-0.352108471	-0.352108471	-0.352108471
8894	5.625160771	5.9078932	5.342428385	5.985836085	6.917329546	6.451582815
8896	-0.352108471	-0.352108	-0.352108471	0.763805499	3.425082766	2.094444132
8897	-0.352108471	-0.352108	-0.352108471	6.857664128	5.688622647	4.519581166

8899	-0.352108471	-0.352108	-0.352108471	6.234483536	6.17482898	6.204656258
8904	-0.352108471	-0.352108	-0.352108471	5.869665514	8.662384619	3.076946409
8905	-0.352108471	-0.352108	-0.352108471	2.588601447	2.076900813	3.100302082
8907	4.942456401	5.3546856	5.766914897	5.821410838	8.986480707	2.656340969
8911	5.749402114	5.4182	6.08060427	-0.352108471	-0.352108471	-0.352108471
8914	8.255357175	5.2545667	6.75496194	9.135176459	7.072343191	5.009509923
8915	1.383520155	-0.352108	3.119148782	1.70308327	1.305230684	2.100935855
8916	3.927296353	5.4937832	7.060270077	9.1382795	0.71707893	4.927679215
8924	6.121583321	6.1079622	6.135204448	5.363386034	4.688318557	6.038453511
8925	6.579013184	7.687325	5.470701366	5.681727979	5.504333593	5.859122364
8927	-0.352108471	-0.352108	-0.352108471	5.821410838	8.066625322	3.576196354
8932	-0.352108471	-0.352108	-0.352108471	4.037188095	3.98310057	4.010144333
8933	8.845585048	7.6440897	6.442594304	8.971186951	7.843980686	10.09839322
8934	4.687728637	4.4585636	4.229398638	6.417610763	6.183964016	5.950317268
8939	6.383151496	6.3831515	6.383151496	5.89334693	6.383151496	5.403542364
8943	8.086273015	7.387333	8.785213013	7.703846689	6.886610445	6.069374202
8971	-0.352108471	-0.352108	-0.352108471	8.65465389	6.92012998	7.787391935
8974	7.233420289	8.2467204	6.220120221	6.369156591	6.666996822	6.071316359
8975	4.262826663	7.5402844	5.901555533	4.268782719	3.638085437	3.953434078
8985	8.958800099	8.5952649	9.322335323	6.386203135	9.341977543	7.864090339
8986	4.873965948	6.9266188	5.90029238	8.254344786	7.201489776	6.148634766
8991	-0.352108471	-0.352108	-0.352108471	3.197838286	1.704743119	2.451290702
8992	9.992085632	7.0425231	4.092960534	7.63243203	5.778593187	6.705512608
8993	-0.352108471	-0.352108	-0.352108471	2.451290702	-0.352108471	5.254689875
8994	4.552447108	2.4458255	0.339203952	0.598807818	1.549724107	-0.352108471
8996	6.242048279	5.1353307	7.348765869	-0.352108471	-0.352108471	-0.352108471
8997	5.622946356	5.8979271	5.347965619	7.237481046	6.092421421	6.664951234
9013	-0.352108471	-0.352108	-0.352108471	8.271767364	6.396868383	4.521969401
9015	-0.352108471	-0.352108	-0.352108471	4.641114331	2.032889041	3.337001686
9016	-0.352108471	-0.352108	-0.352108471	4.846380084	5.369332089	5.892284093
9019	3.127737875	1.5359262	4.719549595	7.918642203	7.112539889	8.724744518
9021	-0.352108471	-0.352108	-0.352108471	5.723485516	7.598136142	3.84883489
9022	-0.352108471	-0.352108	-0.352108471	8.199113911	6.014930938	7.107022425
9025	6.002308672	6.0023087	6.002308672	-0.352108471	-0.352108471	-0.352108471
9028	5.978385123	4.8057376	5.392061359	-0.352108471	-0.352108471	-0.352108471
9031	6.539005987	5.9750867	6.257046343	6.285554459	5.198592136	7.372516781
9033	6.765632416	6.2738323	5.782032102	-0.352108471	-0.352108471	-0.352108471
9037	4.689404048	5.5409946	6.392585219	4.809512228	7.347612652	9.885713077
9040	6.831289207	8.6852124	4.977365966	6.53365233	8.039081087	5.028223574
9043	6.888413829	6.9037827	6.873045007	2.572849659	3.337746555	1.807952763
9045	9.130093579	7.3885414	5.64698925	8.947027392	8.719966382	8.492905372
9049	7.087058971	5.9174671	6.502263028	4.08236011	4.123886406	4.040833815
9050	6.243369603	6.4855628	6.72775599	6.368460376	7.32117851	5.415742243
9052	4.720401452	6.6400194	8.559637334	8.186564808	10.12402513	9.155294971
9060	-0.352108471	-0.352108	-0.352108471	7.929846958	8.004134703	7.855559213
9061	-0.352108471	-0.352108	-0.352108471	5.663737322	3.42307981	7.904394834
9063	5.695288594	6.4105853	4.979991919	4.706961556	5.494417177	3.919505936

9064	3.675218758	7.0463464	5.360782592	-0.352108471	-0.352108471	-0.352108471
9069	-0.352108471	-0.352108	-0.352108471	4.178899946	3.10562882	5.252171071
9070	-0.352108471	-0.352108	-0.352108471	6.553582047	6.758502354	6.6560422
9075	4.807954409	6.1075388	7.407123193	-0.352108471	-0.352108471	-0.352108471
9079	6.076474386	6.441194	6.258834195	6.596757815	6.617035395	6.576480236
9091	-0.352108471	-0.352108	-0.352108471	3.126717648	5.923493182	8.720268716
9092	-0.352108471	-0.352108	-0.352108471	6.687283697	8.518982526	4.855584868
9093	-0.352108471	-0.352108	-0.352108471	0.897715333	-0.352108471	2.147539136
9094	3.475977358	7.2333939	5.354685649	5.244410337	5.983736712	6.723063087
9096	8.200122739	5.9391922	3.678261704	5.2310771	4.08236011	2.933643121
9097	4.08236011	5.6367458	2.527974404	5.145345772	7.140810338	9.136274903
9098	5.387827808	5.0471545	5.217491139	-0.352108471	-0.352108471	-0.352108471
9100	5.688622647	4.7300581	6.647187152	5.264899422	4.66005769	4.055215957
9101	7.697484075	9.3755955	6.019372647	2.383424055	5.596916991	8.810409928
9112	5.45216887	5.2163076	5.334238245	-0.352108471	-0.352108471	-0.352108471
9113	-0.352108471	-0.352108	-0.352108471	4.23169962	5.458255064	6.684810508
9114	-0.352108471	-0.352108	-0.352108471	9.04158568	4.267576158	6.654580919
9117	2.817104043	6.0908083	9.364512638	6.728481336	6.880482436	6.576480236
9118	5.343397067	5.5133907	5.428393874	-0.352108471	-0.352108471	-0.352108471
9122	4.769678434	8.8449948	6.807336599	5.711631646	6.029474081	5.393789211
9123	9.977180362	9.2603759	8.543571489	10.82357252	9.163293869	7.503015218
9124	1.725405383	5.3048771	3.515141236	5.695345386	9.033433542	7.364389464
9125	4.842259381	7.8618483	6.352053864	-0.352108471	-0.352108471	-0.352108471
9126	-0.352108471	-0.352108	-0.352108471	1.086108655	2.902991023	4.71987339
9128	7.797672701	5.1371871	6.467429889	0.044132154	-0.352108471	0.440372779
9130	-0.352108471	-0.352108	-0.352108471	8.675595782	4.575594309	6.625595045
9131	-0.352108471	-0.352108	-0.352108471	2.549953871	4.80208491	7.054215949
9133	-0.352108471	-0.352108	-0.352108471	5.635731685	5.637296679	5.638861673
9136	-0.352108471	-0.352108	-0.352108471	5.38675695	5.992023871	4.78149003
9138	-0.352108471	-0.352108	-0.352108471	8.757071629	6.235921161	3.714770693
9139	10.25363947	9.1167897	7.97993986	7.604342909	7.604530028	7.604717148
9140	4.711580131	-0.083697	2.313941724	1.05704313	0.808855576	1.305230684
9141	7.712003927	4.6114766	10.81253123	-0.352108471	-0.352108471	-0.352108471
9144	-0.352108471	-0.352108	-0.352108471	6.814965918	7.610788767	6.019143068
9145	4.560734218	1.089466	2.8251001	6.369156591	6.056063086	6.682250095
9146	8.862102274	7.2266559	5.591209613	7.624442075	6.671114915	8.577769235
9147	5.506476153	6.3719083	5.939192221	-0.352108471	-0.352108471	-0.352108471
9149	6.392379834	6.056054	5.719728164	5.295431597	3.808300023	6.782563171
9153	7.202880948	6.4702818	7.935480142	7.342842975	4.080420317	5.711631646
9158	-0.352108471	-0.352108	-0.352108471	6.649850066	6.689878453	6.609821679
9159	6.369156591	8.6463338	4.091979363	4.80208491	6.436646051	3.167523769
9166	-0.352108471	-0.352108	-0.352108471	4.927679215	3.722982255	6.132376175
9167	-0.352108471	-0.352108	-0.352108471	5.169157826	3.625762757	6.712552894
9168	10.40955719	11.440461	9.378653109	8.963247175	10.71595765	12.46866813
9169	6.920641908	5.0508701	8.790413753	7.902162009	8.021293639	7.961727824
9170	-0.352108471	-0.352108	-0.352108471	5.958359385	5.958359385	5.958359385
9175	7.997043888	6.4151598	7.206101829	6.689783396	6.078960196	7.300606596

9177	5.91063331	7.559113	4.262153572	-0.352108471	-0.352108471	-0.352108471
9180	9.636006898	7.1533661	8.394686482	8.358023042	5.473576744	6.915799893
9181	7.405829366	4.8637719	6.134800615	8.566745243	6.12990004	3.693054838
9184	6.332466669	6.3439771	6.320956231	-0.352108471	-0.352108471	-0.352108471
9188	5.90029238	5.9200251	5.880559702	7.179921364	7.082394844	7.277447884
9189	8.356138191	5.2893636	6.822750891	0.872801732	0.440372779	1.305230684
9194	9.15729976	7.9172789	8.537289355	7.087030751	7.109037535	7.065023967
9196	-0.352108471	-0.352108	-0.352108471	5.998406448	4.823218894	7.173594001
9200	6.615214539	6.0908083	5.566402142	7.21432556	8.396513207	7.805419383
9203	10.0427294	2.6847959	6.363762655	5.726388281	7.273319574	4.179456987
9208	4.383761877	6.4607607	5.422261279	7.573761745	6.359270363	5.144778982
9209	9.169748699	4.5210305	6.845389604	3.10018379	8.093650192	5.596916991
9212	6.242048279	9.5347842	2.949312392	-0.352108471	-0.352108471	-0.352108471
9213	6.871438113	7.3225599	7.096999017	6.23431917	5.339005699	5.786662435
9215	5.409592279	6.3909925	5.90029238	-0.352108471	-0.352108471	-0.352108471
9217	7.281910873	3.4318806	5.356895761	6.345275458	6.661089146	6.02946177
9218	3.023687642	3.1277379	3.231788108	3.139138355	4.066434892	2.211841818
9219	6.383151496	7.937798	4.828504948	6.206854907	7.272704841	8.338554776
9220	-0.352108471	-0.352108	-0.352108471	3.83155557	3.67501408	3.753284825
9221	-0.352108471	-0.352108	-0.352108471	6.900899666	7.869001359	5.932797972
9230	-0.352108471	-0.352108	-0.352108471	8.171089969	6.121648419	7.146369194
9231	6.131855599	6.4938011	5.769910098	6.177455484	6.588254064	6.999052643
9232	-0.352108471	-0.352108	-0.352108471	7.41450965	7.21467292	7.014836189
9236	7.894349489	5.6799187	6.787134073	0.598807818	1.549724107	-0.352108471
9238	-0.352108471	-0.352108	-0.352108471	4.927679215	4.954218546	4.901139884
9240	-0.352108471	-0.352108	-0.352108471	0.919601171	-0.352108471	2.191310813
9246	-0.352108471	-0.352108	-0.352108471	6.866733042	3.710755367	10.02271072
9252	-0.352108471	-0.352108	-0.352108471	5.412561087	5.881427309	4.943694865
9253	6.352053864	7.7558284	4.948279361	6.702212637	6.204656258	5.707099879
9256	-0.352108471	-0.352108	-0.352108471	6.12990004	6.585243937	5.674556144
9258	7.377755134	7.3860827	7.369427614	-0.352108471	-0.352108471	-0.352108471
9260	9.154512565	3.329584	6.242048279	8.892159505	8.053243237	8.472701371
9261	7.843715778	7.3777551	6.911794489	7.554701412	8.971348772	8.263025092
9262	7.488513026	7.2380437	7.363278387	6.139145127	6.74407332	7.349001514
9263	-0.352108471	-0.352108	-0.352108471	5.961762068	5.714657037	6.208867099
9265	-0.352108471	-0.352108	-0.352108471	6.235921161	7.981251269	4.490591053
9266	5.453390045	4.7738799	6.132900169	-0.352108471	-0.352108471	-0.352108471
9270	6.111060532	7.2999875	8.488914385	5.524645228	5.824288785	6.123932342
9274	-0.352108471	-0.352108	-0.352108471	6.97523337	6.116668207	6.545950789
9275	-0.352108471	-0.352108	-0.352108471	6.087644454	6.101634422	6.073654487
9276	-0.352108471	-0.352108	-0.352108471	7.093272886	5.998628691	6.545950789
9283	5.555243693	6.0488902	5.802066932	1.678838435	0.003114953	3.354561918
9290	-0.352108471	-0.352108	-0.352108471	2.221964512	4.876419248	-0.432490224
9294	2.931165817	5.344627	7.758088225	2.281847319	1.876861374	2.686833264
9295	-0.352108471	-0.352108	-0.352108471	5.400334673	6.43341099	4.367258356
9296	9.036711488	8.1054943	7.174277117	7.996847425	7.261477988	6.52610855
9312	-0.352108471	-0.352108	-0.352108471	5.821410838	5.838255388	5.804566288

9317	5.428393874	5.4582551	5.398532684	-0.352108471	-0.352108471	-0.352108471
9318	-0.352108471	-0.352108	-0.352108471	6.184922964	5.008911019	5.596916991
9319	1.424520744	2.2526182	3.080715679	-0.352108471	-0.352108471	-0.352108471
9320	-0.352108471	-0.352108	-0.352108471	6.74407332	9.723136524	3.765010117
9321	-0.352108471	-0.352108	-0.352108471	6.664782857	6.946414218	6.383151496
9322	8.689382124	5.9391922	3.189002319	2.947260382	6.246629235	-0.352108471
9324	-0.352108471	-0.352108	-0.352108471	4.328943044	4.927679215	5.526415385
9325	-0.352108471	-0.352108	-0.352108471	5.745080913	6.204656258	6.664231603
9329	-0.352108471	-0.352108	-0.352108471	5.773635462	5.657479225	5.715557343
9333	3.826365093	5.4539948	7.081624593	-0.352108471	-0.352108471	-0.352108471
9341	-0.352108471	-0.352108	-0.352108471	1.72638491	1.305230684	2.147539136
9342	6.817987879	7.1464808	6.489495002	8.338361295	8.3259471	8.35077549
9343	6.271724951	7.2219907	5.321459249	1.593612684	4.80208491	8.010557136
9344	6.722855594	6.7737408	6.824625966	4.80208491	3.238780563	6.365389257
9349	8.875726438	7.476284	8.176005205	10.27298918	9.314194364	9.793591774
9351	6.771333876	7.101491	7.431648213	5.538465142	7.331075404	9.123685666
9352	6.332466669	7.3377359	5.327197429	4.424292154	5.169157826	5.914023497
9356	6.068484609	5.5587679	5.049051131	-0.352108471	-0.352108471	-0.352108471
9358	-0.352108471	-0.352108	-0.352108471	3.355050029	2.168105043	0.981160057
9361	-0.352108471	-0.352108	-0.352108471	4.199045311	4.843308497	3.554782125
9362	-0.352108471	-0.352108	-0.352108471	4.66005769	4.688318557	4.631796822
9366	7.300045303	3.0044489	5.152247113	-0.352108471	-0.352108471	-0.352108471
9368	6.313045251	7.0975298	6.705287512	9.582346695	6.603550508	3.624754321
9369	7.403589581	9.6185918	8.511090683	6.640981799	9.478595064	3.803368534
9370	5.932497881	6.1414231	5.723572646	0.476561106	-0.352108471	1.305230684
9372	7.599457653	6.991795	6.384132442	-0.352108471	-0.352108471	-0.352108471
9373	6.273832259	8.4330061	4.114658406	-0.352108471	-0.352108471	-0.352108471
9375	-0.352108471	-0.352108	-0.352108471	7.982438	6.808614206	7.395526103
9377	-0.352108471	-0.352108	-0.352108471	5.558051538	5.412561087	5.267070637
9379	7.051951014	5.6759598	8.427942253	6.092834897	6.220238598	6.3476423
9380	8.018017164	7.5830279	7.148038734	4.15029505	6.676169033	9.202043016
9382	-0.352108471	-0.352108	-0.352108471	7.054600764	7.065023967	7.044177561
9383	10.48662507	10.645369	10.32788103	9.357766202	9.222529006	9.493003397
9390	-0.352108471	-0.352108	-0.352108471	1.601336827	-0.352108471	3.554782125
9391	7.74464347	8.5666969	9.388750386	7.898117032	6.216853774	7.057485403
9392	-0.352108471	-0.352108	-0.352108471	6.50840962	2.85499773	-0.798414159
9394	-0.352108471	-0.352108	-0.352108471	6.705287512	8.093451114	5.31712391
9397	6.07919154	5.7752316	6.383151496	6.381623983	6.448016836	6.31523113
9399	-0.352108471	-0.352108	-0.352108471	5.980334028	5.049755821	6.910912236
9401	-0.352108471	-0.352108	-0.352108471	5.46506016	5.311680183	5.388370172
9403	7.308059056	2.3601448	4.834101937	0.440372779	0.440372779	0.440372779
9406	7.630589424	7.6417956	7.619383264	6.796814879	6.813225323	6.780404436
9411	1.392109249	3.136327	-0.352108471	3.919505936	3.919505936	3.919505936
9412	-0.352108471	-0.352108	-0.352108471	6.186654084	5.140820559	5.663737322
9414	-0.352108471	-0.352108	-0.352108471	6.333716952	5.926083128	6.12990004
9415	5.083528108	5.7494021	6.41527612	6.251034844	8.135069204	4.367000484
9416	-0.352108471	-0.352108	-0.352108471	7.380770874	6.963991695	6.547212516

9419	6.657925647	6.3786425	6.099359439	2.982525938	10.02316438	6.502845157
9424	5.764900762	5.2094052	5.487153005	3.984771689	3.337746555	4.631796822
9435	-0.352108471	-0.352108	-0.352108471	7.146302205	7.168934463	7.123669946
9439	5.688622647	3.8506582	7.526587051	-0.352108471	-0.352108471	-0.352108471
9442	6.537383725	6.5498529	6.524914536	-0.352108471	-0.352108471	-0.352108471
9443	-0.352108471	-0.352108	-0.352108471	1.783698732	-0.352108471	3.919505936
9444	6.369156591	7.3159658	5.422347382	5.88700099	5.117928775	6.656073205
9445	8.418474585	7.7071024	6.99573031	8.482991049	8.727022238	8.971053427
9446	7.737443439	8.1222505	7.929846958	3.485942693	8.918347684	6.202145189
9447	-0.352108471	-0.352108	-0.352108471	4.947028615	3.410771276	4.178899946
9448	5.94941124	5.1750751	6.723747374	3.288054249	11.55578322	7.421918736
9453	8.048540869	10.235425	5.861657216	8.056272062	7.200316908	6.344361754
9454	5.189865576	6.8018138	8.413761971	6.505960865	7.208566448	5.803355282
9455	-0.352108471	-0.352108	-0.352108471	7.855055752	2.612079095	5.233567424
9456	-0.352108471	-0.352108	-0.352108471	5.084660056	5.253655595	5.169157826
9464	-0.352108471	-0.352108	-0.352108471	6.705287512	6.720696571	6.689878453
9468	1.544498927	7.2599694	4.402234161	5.657882681	5.03806008	4.418237479
9469	6.642362719	7.9979469	9.353531152	2.19005738	2.232575625	2.147539136
9470	6.711285967	6.9134889	7.11569183	-0.063442489	8.22816271	4.08236011
9472	7.10070861	6.6741289	6.247549147	6.245851737	6.345275458	6.444699178
9474	-0.352108471	-0.352108	-0.352108471	1.946552907	4.245214285	-0.352108471
9475	0.993969088	2.0080661	1.501017585	7.610507833	5.338134843	3.065761853
9480	8.119613748	3.3791905	5.749402114	-0.352108471	-0.352108471	-0.352108471
9491	6.817644959	6.3288135	6.573229206	7.477384778	6.426248865	5.375112952
9493	-0.352108471	-0.352108	-0.352108471	3.755650079	5.596916991	7.438183904
9495	9.004870837	8.6332332	8.261595649	7.410570876	7.42628567	7.394856081
9497	4.389753559	4.6155835	4.841413467	5.444257186	5.022877661	5.233567424
9500	7.54354356	7.5821735	7.504913637	8.000331568	8.090401543	7.910261593
9506	5.820919659	5.3951129	5.608016291	-0.352108471	-0.352108471	-0.352108471
9507	-0.352108471	-0.352108	-0.352108471	5.912731223	6.419009989	6.165870606
9510	6.369156591	6.3831515	6.355161685	8.74194097	8.75496699	8.72891495
9512	-0.352108471	-0.352108	-0.352108471	5.992709867	5.923493182	5.854276497
9513	7.172893309	7.8265955	8.4802976	1.90830372	-0.799919187	4.616526626
9516	-0.352108471	-0.352108	-0.352108471	5.080688208	5.859617369	6.638546529
9517	8.724757641	7.474345	6.223932405	1.455237762	3.101770383	2.278504072
9518	-0.352108471	-0.352108	-0.352108471	10.61797309	8.095105263	5.572237436
9520	6.737716966	4.5126046	5.625160771	5.458255064	5.458255064	5.458255064
9522	6.180907385	6.3819313	5.979883448	6.97270287	7.234869818	6.710535922
9524	7.277765718	5.911264	8.644267448	5.325716078	7.812756484	2.838675672
9526	-0.352108471	-0.352108	-0.352108471	6.186562848	4.607226501	7.765899196
9527	10.25638879	11.071318	9.441459346	8.838285123	9.285047788	9.061666455
9528	5.420115948	7.4174962	9.414876412	7.144314609	8.144186245	6.144442972
9529	1.880238567	1.0973564	2.66312072	2.759099813	10.14062402	6.449861915
9531	7.17777694	7.1881098	7.167444067	7.107022425	7.123669946	7.090374904
9532	-0.352108471	-0.352108	-0.352108471	7.105752362	7.065023967	7.146480757
9533	-0.352108471	-0.352108	-0.352108471	2.686833264	1.983727385	3.389939142
9537	4.789140709	6.3828735	7.976606244	6.981888777	7.003831781	6.959945773

9538	7.08718283	6.9950186	7.179347036	5.688622647	5.715557343	5.661687951
9540	-0.352108471	-0.352108	-0.352108471	4.456886199	4.482692559	4.43107984
9545	5.52809333	8.721468	7.124780656	6.340207437	5.001397432	5.670802435
9550	-0.352108471	-0.352108	-0.352108471	5.412561087	6.09360634	4.731515835
9551	-0.352108471	-0.352108	-0.352108471	8.474371858	10.57934051	6.369403202
9552	-0.352108471	-0.352108	-0.352108471	6.930501922	6.457843773	6.694172847
9553	-0.352108471	-0.352108	-0.352108471	8.020778366	5.87902495	6.949901658
9556	-0.352108471	-0.352108	-0.352108471	4.73755266	4.843308497	4.631796822
9557	-0.352108471	-0.352108	-0.352108471	5.31996161	6.087644454	6.855327299
9559	6.555842248	5.6886226	4.821403046	8.357031501	7.429168474	6.501305448
9562	8.938395791	4.6954143	6.816905042	-0.352108471	-0.352108471	-0.352108471
9563	6.466502204	6.5498529	6.383151496	7.852779831	7.85000045	7.855559213
9564	2.401210306	5.6251608	8.849111237	6.091028778	8.18491552	10.27880226
9567	-0.352108471	-0.352108	-0.352108471	6.573076731	4.620757251	5.596916991
9570	6.705287512	5.2353542	8.175220834	0.808855576	0.808855576	0.808855576
9572	4.178899946	4.2452143	4.112585606	7.318914431	7.327812407	7.310016454
9575	3.77676452	3.1277379	2.47871123	9.430678828	7.15770761	4.884736392
9578	5.089412202	3.9390226	6.239801767	8.569616524	6.65027966	4.730942797
9580	-0.352108471	-0.352108	-0.352108471	5.91224268	5.284974356	4.657706032
9581	-0.352108471	-0.352108	-0.352108471	3.692383899	3.033522536	2.374661173
9583	8.716980513	7.7652098	8.241095163	7.442803779	8.544556018	7.993679898
9584	4.232695765	6.502263	8.771830291	-0.352108471	-0.352108471	-0.352108471
9587	-0.352108471	-0.352108	-0.352108471	6.220814195	6.104429468	5.988044741
9588	7.668336029	6.7761196	7.222227808	8.631044277	8.150207663	7.669371048
9590	3.328967273	4.8455481	4.087257665	4.413952725	3.67886621	5.14903924
9592	7.07688723	6.3808154	7.772959069	6.278776074	8.1244467	9.970117326
9600	5.980334028	6.0023087	5.958359385	3.585121301	3.615460478	3.554782125
9601	7.063413806	7.065024	7.061803645	7.579247893	7.603251574	7.555244212
9604	9.463768812	7.9132346	6.362700427	8.329369232	6.097445169	3.865521105
9605	-0.352108471	-0.352108	-0.352108471	5.325716078	5.336173319	5.315258837
9610	4.414822703	5.3190968	6.223370817	8.069522068	7.054600764	6.03967946
9612	2.793929048	4.8473688	6.90080847	6.57344213	8.721268123	7.647355126
9616	10.93888962	5.2806171	8.109753335	4.977108406	8.777586176	6.877347291
9618	-0.352108471	-0.352108	-0.352108471	4.295034463	4.66005769	5.025080916
9620	6.481523945	6.6782049	6.284842948	-0.352108471	-0.352108471	-0.352108471
9622	3.19376143	2.9208697	2.647978061	-0.352108471	-0.352108471	-0.352108471
9627	3.066661021	2.4868796	1.907098161	0.153943664	1.167362396	2.180781129
9632	5.625044719	6.9226198	6.273832259	8.829338302	8.299358363	7.769378425
9636	6.827262762	6.3324667	5.837670575	10.5366179	4.771902901	7.654260401
9638	6.471250053	5.0516324	7.890867686	5.98032724	7.083520572	8.186713904
9639	-0.352108471	-0.352108	-0.352108471	0.897715333	-0.352108471	2.147539136
9643	7.932742872	7.960348	7.905137775	8.535406575	8.484269499	8.58654365
9644	7.788231263	7.1121207	6.436010227	7.50587715	7.294652308	7.717101992
9645	5.368273037	5.7208707	6.073468341	0.228373553	-0.352108471	0.808855576
9646	-0.352108471	-0.352108	-0.352108471	5.83972872	6.272379278	6.056053999
9655	-0.352108471	-0.352108	-0.352108471	4.80208491	4.275547921	5.328621899
9657	4.110671996	2.7402102	1.369748469	-0.352108471	-0.352108471	-0.352108471

9658	-0.352108471	-0.352108	-0.352108471	7.136603639	7.149537332	7.123669946
9662	5.540813171	3.7939869	7.287639464	3.194909326	4.449748927	5.704588529
9663	-0.352108471	-0.352108	-0.352108471	3.401768868	8.445217496	5.923493182
9665	6.242048279	6.6422305	5.841866108	-0.352108471	-0.352108471	-0.352108471
9666	-0.352108471	-0.352108	-0.352108471	3.919505936	3.919505936	3.919505936
9668	4.984151706	5.054281	4.914022459	6.410192983	6.662724824	6.157661143
9669	4.130459819	5.2769993	6.423538749	-0.352108471	-0.352108471	-0.352108471
9670	-0.352108471	-0.352108	-0.352108471	7.19609887	7.204087928	7.188109812
9672	8.112154609	6.1705984	10.05371084	0.52324203	1.835337653	1.179289842
9673	2.027788313	2.6743842	2.351086241	0.440372779	0.440372779	0.440372779
9675	8.5356281	3.1702468	5.852937462	5.012919573	5.177928403	5.095423988
9679	-0.352108471	-0.352108	-0.352108471	4.178899946	4.245214285	4.112585606
9683	6.712090045	6.1215288	5.530967493	-0.352108471	-0.352108471	-0.352108471
9684	-0.352108471	-0.352108	-0.352108471	4.456886199	3.888434292	5.025338107
9685	6.27065599	6.6895379	7.108419885	6.31523113	6.31523113	6.31523113
9686	7.404181317	7.7309094	7.567545379	-0.352108471	-0.352108471	-0.352108471
9688	10.24853226	2.5452045	6.396868383	5.723485516	7.026054082	4.42091695
9689	5.724727855	6.7357797	7.746831517	8.629646632	5.908600301	7.269123466
9690	-0.352108471	-0.352108	-0.352108471	5.135818178	7.026456989	3.245179367
9692	-0.352108471	-0.352108	-0.352108471	4.911611019	5.775068216	6.638525413
9693	4.724875807	5.3546856	5.984495491	0.044132154	-0.352108471	0.440372779
9694	-0.352108471	-0.352108	-0.352108471	7.181002342	6.235921161	5.29083998
9696	8.394773729	3.3111012	5.852937462	5.504575986	6.518700948	4.490451024
9697	5.866067462	7.879498	6.872782744	8.564870535	6.654943747	7.609907141
9701	-0.352108471	-0.352108	-0.352108471	7.887852394	5.845499835	3.803147276
9703	4.828601104	1.4268746	3.127737875	6.741172219	7.547428012	7.144300116
9706	6.55915606	4.7333542	5.64625513	6.570974966	5.769160278	7.372789653
9709	-0.352108471	-0.352108	-0.352108471	6.822208827	4.034179148	9.610238505
9710	9.355798846	7.2521642	5.148529572	6.534224101	9.033982726	4.034465476
9711	6.182962498	6.2431999	6.303437262	5.812073039	5.435233852	6.188912226
9712	4.615583513	2.9042317	6.326935301	5.799246042	6.719109903	6.259177972
9716	5.55876787	5.2003646	5.917171177	-0.352108471	-0.352108471	-0.352108471
9717	1.66985715	-0.352108	3.69182277	7.08965874	7.135139919	7.044177561
9723	7.756380467	6.5893927	5.422404893	5.159434386	6.59060933	3.728259442
9724	5.3562704	3.9702163	6.742324514	4.438588347	6.57553158	8.712474813
9726	-0.352108471	-0.352108	-0.352108471	6.796389661	9.347491699	4.245287623
9727	-0.352108471	-0.352108	-0.352108471	6.006937552	5.593462023	5.800199788
9728	-0.352108471	-0.352108	-0.352108471	8.661917215	6.345275458	4.0286337
9731	7.293324715	4.6835916	9.903057782	6.370122071	4.240891086	8.499353055
9737	-0.352108471	-0.352108	-0.352108471	6.123107878	5.575983727	5.028859576
9738	1.383520155	-0.352108	3.119148782	3.010650274	3.625414178	2.39588637
9741	3.127737875	2.5927492	3.66272653	8.02535373	7.487057963	8.563649497
9745	-0.352108471	-0.352108	-0.352108471	5.095423988	2.753180577	7.437667399
9746	-0.352108471	-0.352108	-0.352108471	5.845499835	7.080906062	4.610093608
9747	0.733613288	3.4140749	6.094536452	6.613428082	6.981888777	7.350349472
9748	4.533157933	5.2849744	6.036790779	-0.352108471	-0.352108471	-0.352108471
9749	4.878170137	4.5930152	5.163325066	6.059147646	7.55091747	6.805032558

9752	7.385384826	6.7886295	7.982140193	3.16641146	2.863729349	2.561047239
9753	8.33805394	8.015548	8.660559886	7.372342935	6.337969164	6.855156049
9755	6.649492217	4.0598791	5.354685649	-0.352108471	-0.352108471	-0.352108471
9757	5.148763052	6.4158165	3.88170964	-0.352108471	-0.352108471	-0.352108471
9758	4.413850426	7.1348902	5.774370319	5.88702069	5.959965674	5.923493182
9762	7.638570419	7.6577576	7.619383264	5.83433693	5.893442277	5.775231583
9764	5.753083307	6.7046211	7.656158799	2.368970958	2.051108653	2.686833264
9768	4.834101937	4.9140225	4.754181416	4.900032819	3.919505936	5.880559702
9770	5.093645234	4.6929891	4.292333004	-0.092986493	2.530936724	1.218975116
9772	-0.352108471	-0.352108	-0.352108471	4.66005769	3.667273284	5.652842096
9774	-0.352108471	-0.352108	-0.352108471	2.252618212	2.252618212	2.252618212
9775	-0.352108471	-0.352108	-0.352108471	8.445308345	5.859617369	3.273926393
9779	-0.352108471	-0.352108	-0.352108471	6.266367194	6.592530242	5.940204146
9780	4.152721357	4.3859368	3.919505936	7.505464822	7.584456235	7.426473409
9781	4.482692559	4.4826926	4.482692559	5.175003254	4.688318557	5.661687951
9782	3.390089763	3.6401927	3.515141236	9.304340925	3.027400287	6.165870606
9783	3.527916452	2.0382907	5.017542253	4.623653674	5.859617369	7.095581064
9784	4.895789266	7.7691441	6.332466669	-0.352108471	-0.352108471	-0.352108471
9785	7.733937187	5.3101159	2.886294529	5.649070118	5.102227346	6.19591289
9786	-0.352108471	-0.352108	-0.352108471	5.810973776	6.379908636	5.242038916
9787	-0.352108471	-0.352108	-0.352108471	5.775068216	5.804293869	5.745842563
9789	-0.352108471	-0.352108	-0.352108471	5.095423988	2.683050179	7.507797797
9790	6.413064592	6.1999612	6.626167961	6.541009048	6.566987041	6.515031054
9791	4.486893444	3.9195059	5.054280952	-0.352108471	-0.352108471	-0.352108471
9792	-0.352108471	-0.352108	-0.352108471	7.9089289	5.562630472	6.735779686
9793	2.451290702	-0.352108	5.254689875	6.897103579	7.230265357	6.563941801
9794	-0.352108471	-0.352108	-0.352108471	6.975832766	5.355908446	6.165870606
9797	4.898228221	5.0426837	4.753772693	4.967105233	5.231089211	4.703121254
9798	3.515141236	3.1260028	3.904279715	5.169157826	6.107147168	4.231168483
9801	-0.352108471	-0.352108	-0.352108471	6.866733042	7.435655104	6.297810979
9802	9.135297638	7.4571353	8.296216488	6.851354764	8.585704367	7.718529565
9804	6.585334598	7.3981936	8.211052636	6.74407332	9.263824746	4.224321894
9805	-0.352108471	-0.352108	-0.352108471	3.851914096	7.752449596	11.6529851
9807	-0.352108471	-0.352108	-0.352108471	5.807807786	5.859617369	5.911426952
9808	4.764812292	4.8368672	4.908922128	2.227397414	3.153970607	4.080543799
9810	8.938163999	5.1886636	7.063413806	11.83876934	6.345275458	0.851781579
9811	1.548305613	3.1538669	2.351086241	5.125066048	6.128945019	5.627005534
9812	-0.352108471	-0.352108	-0.352108471	6.51496239	8.952871833	4.077052948
9813	6.573968999	6.8095777	6.338360336	5.24541358	7.163898936	6.204656258
9814	6.079428658	7.2688291	6.674128879	-0.352108471	-0.352108471	-0.352108471
9817	8.020596416	6.4636749	4.906753414	-0.352108471	-0.352108471	-0.352108471
9818	4.837838105	8.4420851	6.63996159	7.040039925	5.114287606	8.965792245
9823	-0.352108471	-0.352108	-0.352108471	4.178899946	8.811336071	-0.45353618
9827	-0.352108471	-0.352108	-0.352108471	5.060292738	8.493027093	1.627558384
9828	2.515697587	2.8638858	2.167509344	4.61952944	7.852312882	6.235921161
9829	-0.352108471	-0.352108	-0.352108471	4.612603102	4.245214285	4.979991919
9830	5.593041911	5.122927	5.357984451	1.835742592	1.731739118	1.939746065

9832	5.780178948	6.1025262	6.424873398	3.723847293	2.686833264	4.760861323
9833	5.165045602	5.037556	5.292535209	5.955899749	6.129328352	5.782471146
9834	-0.352108471	-0.352108	-0.352108471	1.595004939	1.968661759	2.342318579
9840	-0.352108471	-0.352108	-0.352108471	0.873566864	4.30363603	2.588601447
9841	4.071613908	9.7647453	6.918179605	0.176450022	2.960269185	5.744088347
9842	6.519673958	6.5498529	6.489495002	4.748044625	5.383503645	4.112585606
9851	6.888413829	7.1190237	6.65780394	3.315115848	4.482692559	2.147539136
9853	6.121528769	6.2500203	5.993037234	8.822321597	6.903642175	4.984962753
9854	-0.352108471	-0.352108	-0.352108471	8.487701923	3.844039289	6.165870606
9857	7.324847766	6.5282698	6.926558783	3.557676152	5.511864271	7.466052391
9858	-0.352108471	-0.352108	-0.352108471	7.4835379	6.74407332	6.00460874
9860	4.195330899	6.7922355	5.493783215	-0.352108471	-0.352108471	-0.352108471
9861	5.493783215	7.8333776	3.154188805	7.218748868	9.849095998	4.588401739
9862	6.846957097	7.7732847	5.920629473	6.196807598	6.038453511	6.355161685
9866	5.998561302	6.485029	6.971496699	1.075361022	5.564673104	10.05398519
9869	-0.352108471	-0.352108	-0.352108471	5.086979029	2.464102471	3.77554075
9870	6.77374078	6.8769143	6.670567271	2.645094132	1.946552907	1.248011682
9875	4.546288763	3.4534819	5.639095647	9.535832513	4.821247242	7.178539877
9877	-0.352108471	-0.352108	-0.352108471	5.596802035	5.095423988	4.594045941
9878	6.692425927	6.2558778	7.128974067	7.36946426	7.123669946	7.615258575
9879	6.672131844	6.5556422	6.61388704	6.129906531	5.623999158	5.876952844
9881	4.508443336	1.9818275	7.03505914	-0.352108471	-0.352108471	-0.352108471
9885	7.485638078	7.1484312	6.811224416	10.18264246	4.579096867	7.380869666
9886	-0.352108471	-0.352108	-0.352108471	8.566461653	3.013063799	5.789762726
9887	-0.352108471	-0.352108	-0.352108471	0.793818797	1.939746065	-0.352108471
9889	-0.352108471	-0.352108	-0.352108471	6.647129021	6.345275458	6.043421895
9890	4.595554449	6.2738323	7.952110069	-0.352108471	-0.352108471	-0.352108471
9891	6.678493225	6.4815239	6.284554665	5.835851878	6.573460638	6.204656258
9895	4.11446209	5.6886226	7.262783204	4.66005769	6.46289976	2.857215619
9898	6.885065247	7.3585026	6.411627881	9.168274926	4.65870287	6.913488898
9899	5.58470995	6.417871	7.251032027	6.013441144	5.605609608	6.421272681
9901	5.70788994	8.3468616	7.027375773	2.686833264	2.686833264	2.686833264
9902	7.679998338	6.8884138	6.09682932	8.43469238	8.794269606	8.614480993
9903	2.425295226	3.1277379	3.830180525	5.201281981	4.456886199	3.712490418
9904	-0.352108471	-0.352108	-0.352108471	6.749542057	4.697428975	5.723485516
9905	-0.352108471	-0.352108	-0.352108471	2.099323895	2.147539136	2.051108653
9906	6.137964501	6.8049593	5.470969743	1.76984594	1.807952763	1.731739118
9907	7.61557436	9.9698569	8.792715612	6.016006936	3.216492509	8.815521363
9908	7.379848571	7.7862073	7.583027949	-0.352108471	-0.352108471	-0.352108471
9909	-0.352108471	-0.352108	-0.352108471	3.836853754	8.207248096	6.022050925
9910	7.241294412	6.5132981	5.785301759	0.532534912	3.014766647	1.77365078
9913	6.261203695	4.8620061	7.660401276	2.715821749	1.876861374	3.554782125
9915	-0.352108471	-0.352108	-0.352108471	5.859617369	5.873345987	5.845888751
9917	9.752203857	7.8536471	5.955090413	7.144469731	5.184582679	6.164526205
9918	3.071062971	-0.069028	1.501017585	6.443488165	6.620834921	6.532161543
9921	5.338939682	7.1577076	8.976475538	6.384545421	8.139840961	7.262193191
9922	6.643961298	4.7989575	2.953953666	-0.352108471	-0.352108471	-0.352108471

9923	4.025396405	6.0023087	7.979220939	-0.352108471	-0.352108471	-0.352108471
9926	6.186677781	6.137515	6.088352295	6.491713684	6.168550609	5.845387534
9927	6.056053999	4.0475121	8.064595901	-0.352108471	-0.352108471	-0.352108471
9928	8.130079352	4.3267266	6.228402992	6.203468718	3.718345889	4.960907304
9929	6.005139519	6.4793592	6.95357888	1.873849414	3.327395831	0.420302997
9931	10.02371757	2.9199094	6.471813476	7.005535728	7.674102345	8.342668962
9933	6.147000016	6.0023087	6.291691359	-0.352108471	-0.352108471	-0.352108471
9939	6.939027266	6.503264	6.721145652	6.943288527	5.321415098	8.565161956
9942	5.829395824	5.4058873	6.252904388	7.264208882	4.082756168	5.673482525
9943	-0.352108471	-0.352108	-0.352108471	4.990395041	5.704335255	6.418275469
9945	6.570599508	7.512373	5.628825995	6.022050925	8.637376742	3.406725108
9948	7.573741943	5.0321403	10.1153436	10.89380691	6.864581573	8.879194243
9953	7.096300341	6.5905083	6.084716267	-0.352108471	-0.352108471	-0.352108471
9955	-0.352108471	-0.352108	-0.352108471	5.494417177	5.07269015	5.916144203
9961	6.624675593	6.6898785	6.559472732	5.363386034	4.688318557	6.038453511
9962	-0.352108471	-0.352108	-0.352108471	1.90830372	0.44132138	3.375286059
9966	7.196698817	6.3508918	8.042505803	11.09000445	6.857611197	2.625217947
9967	-0.352108471	-0.352108	-0.352108471	7.579958746	7.79736455	8.014770353
9968	2.255229707	2.4139212	2.572612597	0.940233577	2.232575625	-0.352108471
9972	-0.352108471	-0.352108	-0.352108471	5.426834386	6.458486305	5.942660345
9973	-0.352108471	-0.352108	-0.352108471	5.060292738	5.054280952	5.066304524
9978	8.400021833	5.2644402	6.832231007	7.070792134	6.294852751	6.682822443
9980	7.533181164	5.5225958	3.512010431	2.579735877	3.506739767	4.433743658
9984	-0.352108471	-0.352108	-0.352108471	4.66005769	3.448587005	5.871528375
9985	6.77374078	6.4865129	7.060968613	-0.352108471	-0.352108471	-0.352108471
9987	-0.352108471	-0.352108	-0.352108471	4.178899946	4.245214285	4.112585606
9989	8.841775715	5.5739006	7.207838162	6.415928975	6.429470164	6.402387785
9990	5.179066351	7.0906116	6.134838983	7.136909616	7.073709405	7.010509195
9991	-0.352108471	-0.352108	-0.352108471	5.034844435	4.285270944	4.66005769
9993	-0.352108471	-0.352108	-0.352108471	6.962622793	6.991300859	6.933944727
9997	5.458255064	6.8370443	4.079465821	2.397492216	-0.523698052	5.318682485
10000	5.625160771	5.6532998	5.597021732	-0.352108471	-0.352108471	-0.352108471
10005	5.217491139	4.9094248	5.52555751	-0.352108471	-0.352108471	-0.352108471
10006	-0.352108471	-0.352108	-0.352108471	6.345275458	5.524551845	7.16599907
10007	8.553294822	6.9486088	7.750951825	8.633222429	6.99756632	5.361910212
10008	4.93827241	8.3216045	6.62993844	1.429982115	2.051108653	0.808855576
10011	-0.352108471	-0.352108	-0.352108471	6.967882254	6.845389604	7.090374904
10013	8.127936319	6.4815239	4.835111571	-0.352108471	-0.352108471	-0.352108471
10014	-0.352108471	-0.352108	-0.352108471	6.577483275	9.18789191	3.967074641
10015	-0.352108471	-0.352108	-0.352108471	6.332214754	7.971859628	7.152037191
10018	5.895444161	5.9583594	5.832528937	-0.352108471	-0.352108471	-0.352108471
10019	3.156118404	5.4750327	4.315575532	7.923945324	8.123876858	8.023911091
10020	8.173230291	10.39646	5.950000211	6.773559605	7.877865971	8.982172337
10021	5.089412202	7.6803769	2.498447515	-0.352108471	-0.352108471	-0.352108471
10023	-0.352108471	-0.352108	-0.352108471	4.80208491	2.214800769	7.389369052
10024	-0.352108471	-0.352108	-0.352108471	6.79328955	6.12990004	5.466510531
10025	5.55876787	3.7776098	7.339925922	-0.352108471	-0.352108471	-0.352108471

10036	5.984058041	7.020468	6.502263028	-0.352108471	-0.352108471	-0.352108471
10040	-0.352108471	-0.352108	-0.352108471	6.296043865	8.566522921	4.02556481
10042	-0.352108471	-0.352108	-0.352108471	3.137777564	2.169424975	1.201072386
10043	3.922803383	7.533337	11.14387056	-0.352108471	-0.352108471	-0.352108471
10048	6.513393601	4.8786655	3.243937355	-0.352108471	-0.352108471	-0.352108471
10049	-0.352108471	-0.352108	-0.352108471	2.430006404	1.305230684	3.554782125
10051	-0.352108471	-0.352108	-0.352108471	7.407593833	7.305589088	7.509598577
10052	8.022485543	5.7390796	6.880782588	6.131560745	5.049536003	7.213585487
10054	2.114525649	0.6525147	1.383520155	5.945179855	6.514592953	7.084006052
10055	2.868619878	5.7743703	8.680120759	9.70237992	6.396868383	3.091356845
10056	-0.352108471	-0.352108	-0.352108471	6.231462073	6.617035395	5.845888751
10057	2.313941724	-0.352108	4.979991919	-0.352108471	-0.352108471	-0.352108471
10058	-0.352108471	-0.352108	-0.352108471	3.984771689	8.827077036	-0.857533659
10059	5.2437856	3.2117008	7.275870366	7.827149815	5.486185202	6.656667509
10060	-0.352108471	-0.352108	-0.352108471	5.851984659	5.958359385	6.06473411
10061	3.383962077	1.7932408	2.588601447	2.245600013	4.843308497	-0.352108471
10066	-0.352108471	-0.352108	-0.352108471	3.873485727	7.726913848	5.800199788
10067	-0.352108471	-0.352108	-0.352108471	7.392065217	9.495289152	5.288841283
10069	8.688360779	3.8961666	6.292263673	5.081587239	6.624675593	8.167763946
10071	7.26030319	0.5284212	3.894362219	7.866196667	6.04935442	4.232512173
10072	-0.352108471	-0.352108	-0.352108471	2.30059104	5.095423988	7.890256936
10073	-0.352108471	-0.352108	-0.352108471	7.036396027	5.043693827	9.029098227
10075	6.235835821	7.1747392	6.705287512	7.948372706	7.882354958	7.81633721
10076	-0.352108471	-0.352108	-0.352108471	5.090955144	6.327498594	7.564042044
10078	-0.352108471	-0.352108	-0.352108471	2.566521557	2.686833264	2.80714497
10079	5.354685649	8.1605816	2.548789675	0.440372779	0.440372779	0.440372779
10081	5.423418052	8.0978691	6.760643551	5.094944745	7.161529275	6.12823701
10082	5.55876787	6.6884024	4.42913331	-0.352108471	-0.352108471	-0.352108471
10084	7.052946286	7.5438013	6.562091319	7.591099557	5.059520804	6.32531018
10090	6.043995933	5.9200251	6.167966808	-0.352108471	-0.352108471	-0.352108471
10092	8.672195035	7.2078382	5.743481288	5.57263748	8.502960473	7.037798976
10093	8.488257143	8.3208704	8.404563757	6.995453563	6.860600843	6.725748122
10094	7.305341479	6.3675477	8.243135268	7.942419631	7.951790231	7.933049032
10095	6.722720212	8.7405151	7.731617636	8.018799431	9.551011601	6.486587261
10096	3.032100559	5.3546856	7.677270739	8.965057682	9.032201693	9.099345705
10097	7.567545379	5.9598492	9.175241591	8.324693977	8.606752498	8.465723238
10098	-0.352108471	-0.352108	-0.352108471	8.422196636	8.73496788	8.109425393
10099	8.655991794	6.8884506	5.120909473	-0.352108471	-0.352108471	-0.352108471
10101	-0.352108471	-0.352108	-0.352108471	4.400452081	4.688318557	4.112585606
10102	-0.352108471	-0.352108	-0.352108471	6.369156591	8.487113729	4.251199453
10105	5.217491139	5.2437595	5.191222747	7.732958287	7.753815204	7.712101371
10106	7.891291013	9.4635895	6.318992537	8.824627107	4.044662409	6.434644758
10107	8.4041377	3.5565304	5.980334028	-0.352108471	-0.352108471	-0.352108471
10109	7.406780685	6.585366	6.996073349	7.940525332	8.720997106	7.160053558
10111	6.241589745	6.7541237	5.98644149	-0.352108471	-0.352108471	-0.352108471
10114	5.012806723	2.7642256	0.515644509	-0.352108471	-0.352108471	-0.352108471
10116	6.831289207	3.5717279	10.09085056	-0.352108471	-0.352108471	-0.352108471

10120	-0.352108471	-0.352108	-0.352108471	5.28443913	6.202841915	4.366036344
10121	6.502263028	6.5150311	6.489495002	5.559971507	5.458255064	5.661687951
10123	-0.352108471	-0.352108	-0.352108471	5.887891897	5.859617369	5.831342841
10124	-0.352108471	-0.352108	-0.352108471	3.845517346	9.148846702	6.497182024
10127	-0.352108471	-0.352108	-0.352108471	2.954768492	8.49220254	5.723485516
10128	5.464467563	5.7854448	5.624956163	7.018738825	6.553547577	6.786143201
10129	7.614894272	5.6651445	6.640019393	-0.352108471	-0.352108471	-0.352108471
10130	8.040517613	5.0420037	11.03903153	7.443032621	8.557990908	6.328074335
10131	7.867075524	4.375982	6.121528769	6.14092838	8.117699187	7.129313784
10133	1.558607889	3.1435646	2.351086241	6.785344739	7.948859342	9.112373945
10134	6.801813774	6.8152071	6.788420481	5.785312543	6.215464554	5.355160532
10135	7.224081961	7.6288222	6.819341757	4.927679215	7.256744609	2.59861382
10139	4.878665478	5.424892	4.332438977	5.200785865	5.954772223	4.446799506
10140	6.304262672	5.0729826	5.688622647	-0.352108471	-0.352108471	-0.352108471
10142	3.519327359	3.9195059	3.119148782	7.112080183	7.123669946	7.100490419
10146	-0.352108471	-0.352108	-0.352108471	7.254534766	7.396922722	7.112146811
10147	8.960446948	7.3341756	8.147311281	5.766605513	5.752100495	5.781110532
10151	6.888413829	7.923074	5.853753645	6.74407332	5.769826746	7.718319895
10152	6.676634183	6.9981067	6.355161685	7.90849764	7.910261593	7.906733687
10153	4.579618372	4.8071978	4.352038969	-0.352108471	-0.352108471	-0.352108471
10155	9.331311477	8.7226292	9.026970344	8.363856385	7.866438362	8.861274409
10156	1.383520155	-0.352108	3.119148782	5.624956163	2.894005165	8.355907161
10157	7.785115583	7.561036	7.673075797	6.143653628	8.109480151	7.12656689
10159	-0.352108471	-0.352108	-0.352108471	6.739237433	9.082855302	7.911046368
10162	3.894362219	3.6761388	4.112585606	1.769397609	2.100935855	1.437859363
10163	-0.352108471	-0.352108	-0.352108471	4.297639083	4.482692559	4.112585606
10164	-0.352108471	-0.352108	-0.352108471	6.020406059	5.903760504	5.787114949
10166	8.064097622	8.5040074	8.943917233	8.234930555	7.507418023	6.77990549
10168	2.28805764	1.3835202	0.478982671	6.999151529	4.447819503	5.723485516
10169	7.865765445	8.1117158	8.357666133	9.97838355	4.145515629	7.061949589
10174	7.144015656	7.7619438	6.526087512	2.365241789	7.678941672	7.337437481
10175	5.800199788	6.9257977	4.674601876	5.339767796	5.663737322	5.987706848
10179	6.607366144	6.8884289	6.326303357	5.260498334	7.556595622	9.85269291
10181	-0.352108471	-0.352108	-0.352108471	5.622951531	3.123053341	4.373002436
10184	5.616637089	6.6691367	7.721636304	-0.352108471	-0.352108471	-0.352108471
10186	-0.352108471	-0.352108	-0.352108471	8.327422832	6.359270363	4.391117895
10188	6.502263028	8.897189	4.107337069	-0.352108471	-0.352108471	-0.352108471
10189	7.625720986	5.7225368	6.674128879	8.997925677	4.481831973	6.739878825
10190	-0.352108471	-0.352108	-0.352108471	6.258904145	6.832997432	6.545950789
10193	8.26146588	8.3248995	8.388333102	9.418447763	7.969136911	6.519826059
10195	3.637317492	4.4790014	4.058159444	-0.352108471	-0.352108471	-0.352108471
10197	6.275670199	9.5069118	7.891291013	4.876036081	11.04492239	7.960479236
10199	5.749402114	5.7752316	5.723572646	-0.352108471	-0.352108471	-0.352108471
10201	5.336797593	7.8458821	6.59133985	8.519718399	4.404136098	6.461927248
10204	6.633391004	4.3217914	5.477591179	8.404582737	6.852471422	7.628527079
10206	7.01689787	2.2123474	4.614622638	3.724386732	5.980653558	8.236920384
10209	8.764190522	9.5528476	7.975533452	8.651507263	10.18202757	9.416767417

10210	-0.352108471	-0.352108	-0.352108471	5.363214028	6.12990004	6.896586052
10211	4.178899946	4.4723897	3.885410195	8.057914127	10.49998128	5.615846978
10213	4.821139212	5.6886226	6.556106082	3.919505936	4.775844621	3.06316725
10216	6.346779205	5.2536204	5.800199788	-0.352108471	-0.352108471	-0.352108471
10217	-0.352108471	-0.352108	-0.352108471	5.637296679	3.321105474	7.953487884
10220	6.801813774	7.6893311	5.914296484	-0.352108471	-0.352108471	-0.352108471
10226	7.160491391	7.4885725	7.816653562	8.657468488	8.022372859	7.38727723
10227	7.586479633	6.4869152	5.387350737	0.044132154	-0.352108471	0.440372779
10228	1.392109249	3.136327	-0.352108471	6.235921161	5.925950508	6.545891814
10229	5.798839501	6.3324667	6.866093836	-0.352108471	-0.352108471	-0.352108471
10233	-0.352108471	-0.352108	-0.352108471	4.877659669	5.128763636	5.379867603
10236	-0.352108471	-0.352108	-0.352108471	1.77976078	0.498717195	3.060804366
10237	-0.352108471	-0.352108	-0.352108471	7.478462511	7.49196897	7.464956052
10238	-0.352108471	-0.352108	-0.352108471	6.463674915	7.831839331	5.095510498
10239	5.699084559	2.7312357	4.215160124	1.845238787	2.952060305	0.73841727
10241	4.613886128	2.9817586	3.797822384	4.114512125	3.120807694	2.127103263
10244	1.383520155	1.1441093	1.622931049	5.533333932	6.33538923	7.137444528
10245	-0.352108471	-0.352108	-0.352108471	2.643871277	7.546976699	5.095423988
10248	5.916797067	9.6328437	7.774820401	-0.352108471	-0.352108471	-0.352108471
10250	6.212536209	6.2312245	6.193847962	7.013360586	7.028614492	6.998106681
10252	6.840745137	6.2125362	5.584327281	-0.352108471	-0.352108471	-0.352108471
10254	5.217491139	5.2437595	5.191222747	5.388370172	5.421579811	5.355160532
10257	5.708446629	4.3675309	7.049362332	-0.352108471	-0.352108471	-0.352108471
10262	-0.352108471	-0.352108	-0.352108471	5.004265437	6.204656258	7.405047079
10263	7.646354586	5.1522471	2.65813964	-0.352108471	-0.352108471	-0.352108471
10267	-0.352108471	-0.352108	-0.352108471	3.76552139	3.902599966	3.628442815
10269	-0.352108471	-0.352108	-0.352108471	5.284974356	5.315258837	5.254689875
10272	3.805664353	3.2570297	4.354298975	10.04490521	5.64354012	1.242175028
10273	10.38320977	7.3432278	4.303245861	1.968661759	4.324587311	-0.387263794
10277	5.42997807	5.1399706	5.284974356	5.815741428	6.204656258	6.593571088
10280	6.585218739	7.909603	9.2339873	9.124781927	7.47270066	5.820619393
10282	-0.352108471	-0.352108	-0.352108471	4.926946533	7.791594193	6.359270363
10283	-0.352108471	-0.352108	-0.352108471	4.995200701	5.170966476	4.819434926
10284	7.334332312	5.7790475	6.556689902	3.916292728	7.374725876	10.83315902
10285	4.490133717	5.2849744	6.079814995	1.493791735	1.549724107	1.437859363
10287	-0.352108471	-0.352108	-0.352108471	4.927679215	4.954218546	4.901139884
10290	-0.352108471	-0.352108	-0.352108471	5.904777429	6.186562848	6.468348268
10291	-0.352108471	-0.352108	-0.352108471	6.406162335	7.381783128	6.893972732
10294	5.55876787	5.5882244	5.529311366	5.789762726	5.804293869	5.775231583
10295	-0.352108471	-0.352108	-0.352108471	8.338289949	6.369156591	4.400023232
10296	-0.352108471	-0.352108	-0.352108471	7.730908829	6.64213435	5.55335987
10298	7.055211028	6.056054	5.05689697	8.097498543	5.080301693	6.588900118
10299	2.970907025	9.1050561	6.03798158	6.431242245	6.479332994	6.383151496
10300	-0.352108471	-0.352108	-0.352108471	8.445718401	5.800199788	3.154681174
10301	4.067170576	4.5639805	4.315575532	1.954444309	2.100935855	1.807952763
10307	5.840966015	5.9583594	5.723572646	-0.352108471	-0.352108471	-0.352108471
10312	6.537383725	6.5498529	6.524914536	-0.352108471	-0.352108471	-0.352108471

10313	6.640019393	6.6538708	6.626167961	6.87035267	6.617035395	7.123669946
10314	-0.352108471	-0.352108	-0.352108471	4.54723853	7.628050379	6.087644454
10318	6.991795048	6.0948019	7.88878824	3.939384717	8.320415364	6.12990004
10320	-0.352108471	-0.352108	-0.352108471	1.82202867	1.646479888	1.997577452
10321	-0.352108471	-0.352108	-0.352108471	2.605817304	-0.352108471	5.563743079
10327	6.913488898	6.9268019	6.900175883	-0.352108471	-0.352108471	-0.352108471
10328	-0.352108471	-0.352108	-0.352108471	7.167444067	7.168934463	7.165953671
10330	-0.352108471	-0.352108	-0.352108471	7.022760502	6.96585859	7.079662414
10335	8.508408768	8.087084	8.929733556	8.200258537	7.181258248	7.690758392
10336	-0.352108471	-0.352108	-0.352108471	2.299826999	3.009752481	2.65478974
10342	-0.352108471	-0.352108	-0.352108471	7.231334026	6.44889409	6.840114058
10344	4.798957482	4.617923	4.979991919	-0.352108471	-0.352108471	-0.352108471
10350	7.155718933	6.0395505	8.271887414	6.13117046	4.137217026	2.143263592
10352	6.753390078	4.4969315	5.625160771	4.942702387	5.124543452	4.760861323
10360	-0.352108471	-0.352108	-0.352108471	5.704335255	3.637195778	7.771474731
10361	-0.352108471	-0.352108	-0.352108471	2.290721782	2.048128167	2.169424975
10362	8.401000743	6.6238559	4.846711094	7.459565715	8.502766296	9.545966877
10365	7.330599807	6.4185724	6.874586128	2.221964512	2.252618212	2.191310813
10367	-0.352108471	-0.352108	-0.352108471	6.022050925	2.554928357	9.489173494
10370	6.952707338	7.1660146	6.739400116	9.520074913	11.37152226	10.44579859
10371	7.519300456	5.555467	6.537383725	6.056053999	5.685753359	6.426354639
10376	8.529817333	6.4660176	10.59361703	9.723338277	7.871676892	11.57499966
10379	5.334199106	6.2145415	5.774370319	6.713776402	9.473291939	3.954260865
10380	5.730355425	5.0601231	5.395239246	6.316009143	2.67573404	9.956284246
10381	6.273832259	8.1065923	4.441072261	5.284974356	7.125487499	3.444461213
10383	8.321012347	8.8776862	8.599349265	10.70816805	8.898919086	7.089670124
10384	5.625160771	5.6532998	5.597021732	3.3031696	3.919505936	2.686833264
10385	-0.352108471	-0.352108	-0.352108471	2.515697587	2.12253331	2.908861864
10388	-0.352108471	-0.352108	-0.352108471	3.90817742	8.830135761	6.369156591
10389	5.672110005	5.9391922	6.206274438	-0.352108471	-0.352108471	-0.352108471
10391	5.784956449	7.2543915	6.519673958	-0.352108471	-0.352108471	-0.352108471
10392	5.578084092	5.7494021	5.920720137	-0.352108471	-0.352108471	-0.352108471
10397	6.952707338	6.9786128	6.926801914	-0.352108471	-0.352108471	-0.352108471
10398	12.34527426	9.2026238	10.77394903	10.81249552	11.11853766	11.4245798
10399	10.12270098	10.385524	9.859878334	9.663736784	9.669177738	9.65829583
10400	-0.352108471	-0.352108	-0.352108471	6.274589383	5.149937472	5.712263427
10401	-0.352108471	-0.352108	-0.352108471	0.95025487	-0.352108471	2.252618212
10402	3.826118735	4.7244087	5.622698566	0.044132154	0.440372779	-0.352108471
10403	4.283753424	7.3166462	5.800199788	-0.352108471	-0.352108471	-0.352108471
10409	5.963493203	6.6235409	7.283588663	7.462761226	6.280962497	8.644559955
10410	9.015732225	7.0557278	10.97573661	8.67383023	9.291255946	8.056404514
10412	7.357801878	5.980334	4.602866179	-0.352108471	-0.352108471	-0.352108471
10413	6.280726552	6.3261959	6.303461245	-0.352108471	-0.352108471	-0.352108471
10417	8.266065935	9.3178897	10.36971354	9.355765616	7.411055208	8.383410412
10419	-0.352108471	-0.352108	-0.352108471	8.68052251	7.025721295	5.37092008
10421	7.556752908	5.5232397	3.48972653	7.488588854	10.14014613	4.837031579
10423	5.284974356	5.4779632	5.091985502	0.026433033	9.051075133	4.538754083

10424	4.434314669	4.4826926	4.385936778	3.818098858	4.482692559	3.153505157
10427	6.801813774	5.5092133	8.094414283	3.274533615	5.88700099	8.499468364
10428	-0.352108471	-0.352108	-0.352108471	5.50759102	6.409127749	5.958359385
10430	-0.352108471	-0.352108	-0.352108471	3.33540524	3.585121301	3.834837363
10432	4.833143003	3.136327	1.439510935	-0.352108471	-0.352108471	-0.352108471
10435	-0.352108471	-0.352108	-0.352108471	6.220691285	5.898665446	6.542717123
10439	-0.352108471	-0.352108	-0.352108471	2.419557138	5.191222747	-0.352108471
10440	8.722282842	6.3160723	7.51917757	5.657236274	6.235921161	6.814606048
10443	-0.352108471	-0.352108	-0.352108471	4.690341824	5.928198256	5.30927004
10444	-0.352108471	-0.352108	-0.352108471	6.260835661	6.073654487	6.448016836
10445	6.886095759	7.1849197	6.587271834	6.345275458	3.581074346	9.109476569
10447	5.354685649	4.9445505	5.764820791	3.681745094	7.376877638	5.529311366
10449	5.354685649	5.3835036	5.325867653	-0.352108471	-0.352108471	-0.352108471
10452	-0.352108471	-0.352108	-0.352108471	5.546954141	7.936269959	6.74161205
10456	8.925224375	5.4463076	7.185765998	5.114516014	5.507852562	4.721179467
10457	4.806600156	5.0171364	5.227672716	8.122647789	5.760563852	6.94160582
10458	-0.352108471	-0.352108	-0.352108471	2.024343053	0.988773321	3.059912784
10459	4.672248391	5.7184598	6.764671143	4.927679215	7.627130039	2.228228391
10460	-0.352108471	-0.352108	-0.352108471	6.220238598	6.246629235	6.193847962
10461	-0.352108471	-0.352108	-0.352108471	0.874413692	-0.352108471	2.100935855
10465	7.140442362	4.7379421	5.939192221	-0.352108471	-0.352108471	-0.352108471
10466	-0.352108471	-0.352108	-0.352108471	6.626175926	5.88700099	5.147826054
10467	-0.352108471	-0.352108	-0.352108471	7.475171153	8.193060637	6.75728167
10468	-0.352108471	-0.352108	-0.352108471	7.762081629	5.291399258	2.820716887
10471	6.634368726	5.879724	6.257046343	8.004900657	6.545950789	5.08700092
10473	-0.352108471	-0.352108	-0.352108471	2.268008971	2.357098027	2.446187082
10474	-0.352108471	-0.352108	-0.352108471	6.523594479	8.238413277	4.80877568
10475	5.326927409	5.2047476	5.449107178	2.873046469	1.337497878	4.40859506
10476	3.127737875	2.8191828	3.436292982	7.082757193	7.687916929	6.477597457
10477	5.794304679	5.5601747	5.677239679	6.65835028	6.123867278	5.589384276
10480	9.139523774	6.3034612	3.467398716	8.508685238	6.997455924	5.48622661
10482	5.625160771	5.6532998	5.597021732	6.804723141	6.821025802	6.788420481
10483	3.519905526	5.4283939	7.336882222	8.361059188	5.004585697	6.682822443
10484	1.392109249	3.136327	-0.352108471	4.708211873	6.566381484	5.637296679
10487	6.162962842	6.3691566	6.575350339	8.490904822	8.878126987	8.103682657
10488	3.127737875	4.5453335	1.710142232	8.293951239	7.349917404	6.405883568
10489	6.276090343	6.5191546	6.033026063	-0.352108471	-0.352108471	-0.352108471
10490	-0.352108471	-0.352108	-0.352108471	6.886610445	7.264090544	6.509130346
10491	7.03729673	9.2389969	8.138146831	6.434788104	7.35694753	8.279106956
10492	3.635213903	5.5959531	4.615583513	7.135075351	7.591999306	6.678151396
10493	7.623136656	8.9114007	8.267268675	7.642665151	9.0676641	8.355164626
10497	5.45176171	5.1799508	5.723572646	0.228373553	0.808855576	-0.352108471
10498	8.023219797	6.7737408	5.524261762	8.563144682	6.251034844	3.938925005
10499	4.916925479	5.3875687	5.152247113	-0.352108471	-0.352108471	-0.352108471
10507	-0.352108471	-0.352108	-0.352108471	6.25897455	6.454261972	6.649549394
10513	2.465782294	1.6698571	0.873932005	5.716588855	6.509641199	6.113115027
10514	-0.352108471	-0.352108	-0.352108471	5.734172375	6.450220033	5.018124717

10516	-0.352108471	-0.352108	-0.352108471	5.909224886	10.63440845	8.271816669
10519	-0.352108471	-0.352108	-0.352108471	6.556689902	7.369126349	5.744253454
10520	-0.352108471	-0.352108	-0.352108471	6.08682898	5.912808598	6.260849361
10521	6.926801914	6.9268019	6.926801914	5.263851904	5.315258837	5.212444972
10523	6.320335654	8.5473925	7.433864099	8.162696129	3.387440303	5.775068216
10524	-0.352108471	-0.352108	-0.352108471	1.778924448	2.252618212	1.305230684
10525	7.496938343	3.868905	11.12497167	6.621168045	5.677165024	7.565171067
10526	-0.352108471	-0.352108	-0.352108471	9.315286188	3.423026993	6.369156591
10527	7.926137273	6.0088852	6.967511245	6.671713129	7.152037191	7.632361252
10528	5.948556941	5.6886226	5.428688354	8.52602621	7.04039671	7.78321146
10529	5.980334028	8.9859357	2.974732375	5.017136436	3.89200288	6.142269992
10531	1.383520155	-0.352108	3.119148782	6.859217306	6.873045007	6.845389604
10534	-0.352108471	-0.352108	-0.352108471	7.749716414	8.825757934	6.673674894
10536	7.25123391	9.0686893	8.159961629	6.909421119	6.277861749	7.540980488
10539	5.701328676	5.8191452	5.583512151	6.506485631	7.808929589	7.15770761
10540	6.394059102	3.7104763	5.052267685	9.310246915	7.776096252	6.241945589
10541	7.551136183	7.5003787	7.601893664	6.306122915	6.33538923	6.276856601
10542	6.332466669	6.9950824	5.669850919	-0.352108471	-0.352108471	-0.352108471
10544	-0.352108471	-0.352108	-0.352108471	5.369332089	5.383503645	5.355160532
10548	6.97953737	6.7425776	6.861057494	-0.352108471	-0.352108471	-0.352108471
10549	7.134723344	6.336836	6.735779686	7.163203647	5.168537565	6.165870606
10550	6.954787303	4.422458	5.688622647	7.973156313	7.165804888	6.358453463
10552	9.081164901	7.4157943	5.750423757	3.169979196	6.150136183	4.66005769
10553	-0.352108471	-0.352108	-0.352108471	6.325740245	5.821410838	5.317081431
10554	3.284245804	1.9518701	2.618057952	-0.352108471	-0.352108471	-0.352108471
10555	1.392109249	3.136327	-0.352108471	6.988612598	7.364389464	7.74016633
10556	4.954063105	4.5796184	4.205173639	6.907068059	4.604607385	5.755837722
10558	5.676689474	6.201695	5.939192221	-0.352108471	-0.352108471	-0.352108471
10564	5.838496068	6.9675112	8.096526422	6.95467348	5.942660345	4.93064721
10565	-0.352108471	-0.352108	-0.352108471	5.595814675	5.017136436	4.438458196
10567	7.621561615	6.258182	6.939871798	7.444308991	6.933209143	7.95540884
10572	-0.352108471	-0.352108	-0.352108471	5.357169056	6.235352281	7.113535507
10573	8.898919277	6.6675036	7.78321146	7.600081918	6.283129722	6.94160582
10574	6.667442303	6.7286295	6.606255082	8.101097862	7.624442075	7.147786288
10575	-0.352108471	-0.352108	-0.352108471	6.751551888	5.960183956	7.542919819
10576	9.129763593	6.5529721	7.841367827	7.9391614	8.278231495	7.600091306
10577	8.351322067	7.4780298	9.224614362	8.481181495	7.609959778	6.738738061
10581	6.170862652	3.734353	8.607372283	8.706814134	8.164107005	8.43546057
10587	3.56024929	4.0510794	3.805664353	6.692058426	7.953646928	5.430469923
10589	8.371322004	9.8948286	6.847815431	8.990620862	8.792533605	8.891577233
10591	-0.352108471	-0.352108	-0.352108471	7.099691722	6.056053999	5.012416276
10592	2.553073296	-0.352108	5.458255064	2.686833264	2.686833264	2.686833264
10594	4.999149256	5.9558997	6.912650242	7.863190169	7.899243108	7.881216638
10595	1.392109249	3.136327	-0.352108471	5.38675695	2.386756102	8.386757799
10598	7.609255075	7.6152586	7.603251574	4.977710418	5.054280952	4.901139884
10602	3.571508387	5.5279483	4.549728355	7.760506878	7.374725876	6.988944873
10605	9.185315806	8.2032738	7.221231853	8.032080022	4.341045674	6.186562848

10606	7.804062284	7.9704012	7.88723175	11.67778911	5.289041695	8.483415404
10607	3.127737875	5.4897166	0.765759116	5.169157826	5.554090283	4.784225368
10608	5.308304737	5.9391922	6.570079706	6.886610445	4.87424533	8.89897556
10609	4.53883581	7.6675845	6.10321016	6.704359571	7.564061146	5.844657995
10611	7.278126217	7.6401479	7.459137062	9.127448217	5.55963739	7.343542803
10612	5.990176999	5.6251608	5.260144544	5.980334028	6.583157653	5.377510404
10613	-0.352108471	-0.352108	-0.352108471	2.902991023	3.119148782	2.686833264
10616	6.735779686	5.1832398	8.28831954	4.065625615	7.261849028	5.663737322
10618	7.413868407	6.5797638	6.996816126	8.307099638	7.936818749	8.121959194
10621	6.369156591	6.3831515	6.355161685	-0.352108471	-0.352108471	-0.352108471
10626	6.927337071	7.2952014	6.559472732	3.356392206	1.646479888	5.066304524
10627	5.316351434	7.2698636	9.22337579	8.96475715	8.616158512	8.790457831
10628	-0.352108471	-0.352108	-0.352108471	2.983199304	3.119148782	3.25509826
10631	3.805664353	3.9195059	3.69182277	7.369305842	7.386082653	7.35252903
10632	-0.352108471	-0.352108	-0.352108471	7.305295945	6.833353607	7.069324776
10634	6.273832259	4.8230783	7.724586246	7.492124558	6.77374078	6.055357002
10636	-0.352108471	-0.352108	-0.352108471	4.73755266	4.843308497	4.631796822
10640	6.104376826	6.1047983	6.10521979	-0.352108471	-0.352108471	-0.352108471
10644	1.392109249	2.929757	-0.145538461	9.028372788	6.468732402	7.748552595
10645	6.379245937	6.8024304	6.590838154	6.225468335	5.406240744	7.044695926
10652	5.364951066	6.3409239	5.852937462	7.136603639	7.475108298	6.79809898
10654	6.121528769	5.2195891	7.023468447	-0.352108471	-0.352108471	-0.352108471
10655	4.337205319	5.247402	4.792303669	-0.352108471	-0.352108471	-0.352108471
10656	-0.352108471	-0.352108	-0.352108471	4.233765303	5.370404518	4.80208491
10657	-0.352108471	-0.352108	-0.352108471	8.046801131	7.535706852	8.557895409
10658	-0.352108471	-0.352108	-0.352108471	6.12990004	9.747924613	2.511875468
10664	-0.352108471	-0.352108	-0.352108471	5.810973776	4.985256248	6.636691304
10670	8.014580832	6.9134889	5.812396964	-0.352108471	-0.352108471	-0.352108471
10671	5.722516228	4.9156773	5.31909676	6.412893955	5.034077076	5.723485516
10672	5.677070927	6.4350371	6.056053999	7.165804888	10.02534264	4.306267139
10681	6.954372958	7.8965146	8.838656231	0.156551639	1.622488375	3.088425111
10682	-0.352108471	-0.352108	-0.352108471	5.775068216	6.368438967	5.181697465
10686	3.948901707	5.0082279	6.067554021	3.103185284	2.30692697	3.899443597
10687	7.816982761	9.0223744	8.419678556	7.65696834	7.563313371	7.469658401
10694	6.861057494	9.381584	4.340531017	8.373719206	7.615410705	7.994564955
10695	-0.352108471	-0.352108	-0.352108471	7.567749599	5.957028947	9.178470251
10712	4.353686578	5.5882244	3.119148782	2.169424975	2.191310813	2.147539136
10714	-0.352108471	-0.352108	-0.352108471	6.425371423	5.229586607	7.621156238
10720	2.728425652	5.3546856	7.980945646	0.476561106	-0.352108471	1.305230684
10723	5.257563032	6.6856354	5.971599196	-0.352108471	-0.352108471	-0.352108471
10724	-0.352108471	-0.352108	-0.352108471	8.449295725	7.224139774	5.998983823
10725	7.995086847	6.3180937	4.641100514	0.440372779	0.440372779	0.440372779
10726	6.001716416	6.6073775	7.213038502	5.412561087	3.670571575	7.1545506
10728	6.026242499	9.02932	7.527781259	10.08474722	7.025721295	3.966695367
10730	3.735110573	4.0310378	4.326965036	4.748497651	7.25945936	6.003978506
10739	7.140329269	5.3773391	6.258834195	-0.352108471	-0.352108471	-0.352108471
10740	5.318797552	6.146078	4.4915171	6.265347842	6.215464554	6.31523113

10741	5.991047043	5.365845	5.678446034	7.312772752	5.589157833	9.036387672
10743	-0.352108471	-0.352108	-0.352108471	2.686833264	2.686833264	2.686833264
10746	7.822138356	4.4209192	6.121528769	-0.352108471	-0.352108471	-0.352108471
10749	8.120300038	8.1635425	8.077057613	8.22430719	8.257485986	8.191128393
10755	7.052946286	6.9121615	7.193731058	7.211532041	7.572743317	7.392137679
10758	7.152376959	6.6835209	6.214664764	3.556644279	1.80072102	2.678682649
10762	7.912896449	7.3238652	6.734833992	0.86953393	0.808855576	0.930212283
10763	6.090808341	6.1079622	6.073654487	7.225513213	7.246938498	7.204087928
10765	3.065321785	4.1788999	5.292478106	4.688079496	3.585121301	2.482163107
10766	-0.352108471	-0.352108	-0.352108471	7.090076417	7.100490419	7.079662414
10767	-0.352108471	-0.352108	-0.352108471	6.609543024	5.066206144	8.152879903
10771	-0.352108471	-0.352108	-0.352108471	3.585121301	3.615460478	3.554782125
10772	10.42540579	6.8312892	3.237172628	5.983736712	8.362626905	3.604846519
10776	6.873791272	7.617511	6.130071529	3.688062254	11.05517075	7.3716165
10780	7.429967756	6.2859506	6.857959174	1.530736894	0.808855576	2.252618212
10785	5.152247113	5.0756167	5.228877567	-0.352108471	-0.352108471	-0.352108471
10787	7.120834324	6.991795	6.862755771	5.043358363	7.883991467	6.463674915
10791	-0.352108471	-0.352108	-0.352108471	6.923295051	6.946414218	6.900175883
10793	7.131107385	5.3340027	6.232555018	5.652071908	3.887516359	7.416627456
10797	1.099634066	1.7836987	2.467763399	4.216655461	4.902742936	4.559699198
10801	5.284974356	5.4829684	5.086980282	10.11385558	8.533000693	6.952145804
10802	-0.352108471	-0.352108	-0.352108471	6.168642212	6.859217306	7.549792399
10807	-0.352108471	-0.352108	-0.352108471	5.663737322	5.665786693	5.661687951
10808	6.84410225	7.0510046	6.947553426	6.056053999	6.205260569	5.906847429
10810	-0.352108471	-0.352108	-0.352108471	4.269304063	7.990496018	6.12990004
10813	-0.352108471	-0.352108	-0.352108471	8.702129534	4.436188645	6.56915909
10818	7.051041553	2.8429728	4.947007189	2.314044813	7.290125007	4.80208491
10824	9.084521048	7.4698766	5.855232142	0.044132154	-0.352108471	0.440372779
10826	4.663000528	2.7560812	6.569919856	4.295277691	6.440038111	8.584798531
10844	-0.352108471	-0.352108	-0.352108471	4.66005769	4.688318557	4.631796822
10845	-0.352108471	-0.352108	-0.352108471	6.186562848	4.847227721	7.525897976
10846	-0.352108471	-0.352108	-0.352108471	4.486432534	4.66005769	4.833682846
10847	6.861057494	5.9020163	7.82009865	-0.352108471	-0.352108471	-0.352108471
10848	2.588601447	5.5293114	-0.352108471	-0.352108471	-0.352108471	-0.352108471
10849	7.05185842	7.672332	7.362095214	5.752394131	6.004748528	6.257102924
10855	5.326927409	5.5293114	5.124543452	1.305230684	1.305230684	1.305230684
10856	-0.352108471	-0.352108	-0.352108471	10.9961967	4.420057488	7.708127092
10857	-0.352108471	-0.352108	-0.352108471	2.686117285	8.760853746	5.723485516
10858	2.803125457	-0.352108	5.958359385	2.099323895	2.147539136	2.051108653
10863	-0.352108471	-0.352108	-0.352108471	4.75330713	5.017136436	5.280965741
10865	7.792033924	7.6064982	7.977569637	9.183909136	6.713776402	4.243643668
10867	6.450950486	6.4912436	6.531536689	6.186562848	7.520384783	4.852740914
10868	-0.352108471	-0.352108	-0.352108471	1.783698732	-0.352108471	3.919505936
10869	4.386224296	4.0408587	4.731589924	3.51352536	6.758753021	10.00398068
10875	4.104525714	2.15095	3.127737875	3.722142557	1.347343115	2.534742836
10881	-0.352108471	-0.352108	-0.352108471	6.218320354	6.235921161	6.253521967
10885	-0.352108471	-0.352108	-0.352108471	4.297639083	5.673208817	2.922069348

10886	6.30347915	3.9801886	5.141833898	0.791292145	0.440372779	1.14221151
10888	7.681594358	6.2474636	4.813332809	5.692910329	5.844279396	5.995648463
10890	5.800199788	5.825168	5.775231583	-0.352108471	-0.352108471	-0.352108471
10893	-0.352108471	-0.352108	-0.352108471	4.835137762	4.613679539	4.724408651
10897	7.436342955	4.3155755	1.194808109	9.327401888	7.123643365	4.919884842
10899	5.69427937	6.6073775	7.520475548	9.582417934	6.272010444	7.927214189
10902	6.24569579	5.6326887	5.939192221	6.905535737	9.296248482	4.514822992
10904	3.580159247	5.5204564	4.550307802	7.883781477	7.629862983	8.13769997
10905	4.657831069	5.3764418	5.017136436	0.044132154	-0.352108471	0.440372779
10907	6.056053999	6.0736545	6.038453511	5.637296679	5.653299811	5.621293547
10908	6.657217604	7.1205872	6.193847962	-0.352108471	-0.352108471	-0.352108471
10910	-0.352108471	-0.352108	-0.352108471	7.400204331	7.423933442	7.376475219
10912	2.938787496	4.9470072	6.955226882	-0.352108471	-0.352108471	-0.352108471
10914	-0.352108471	-0.352108	-0.352108471	7.462761226	9.504921948	5.420600504
10916	-0.352108471	-0.352108	-0.352108471	8.091794594	9.167136066	7.016453122
10919	5.284974356	5.4213407	5.148607968	-0.352108471	-0.352108471	-0.352108471
10920	-0.352108471	-0.352108	-0.352108471	5.505017309	7.185533606	6.345275458
10921	7.593149813	6.7469441	8.439355505	8.558377898	6.751466056	4.944554215
10922	7.186619416	6.3999555	7.973283314	6.539318808	7.272998452	5.805639164
10923	6.18142457	6.033653	6.107538801	6.946330252	7.165707925	6.726952578
10926	5.523239719	4.6090888	6.437390683	-0.352108471	-0.352108471	-0.352108471
10928	-0.352108471	-0.352108	-0.352108471	5.903760504	8.491044216	3.316476792
10929	5.360763101	6.3055197	7.250276371	6.412230992	7.799418975	7.105824984
10933	5.374265435	6.0029799	5.688622647	8.260049597	7.552716094	8.9673831
10938	3.997713568	9.0770539	6.537383725	5.452431129	6.577483275	7.702535421
10939	4.046609087	7.6592658	5.852937462	1.873505285	3.919505936	5.965506587
10944	12.71762375	9.2463753	5.775126873	7.344731636	4.932969915	9.756493356
10945	6.059664267	7.3777551	8.695846	7.190571761	6.162641978	8.218501544
10946	-0.352108471	-0.352108	-0.352108471	6.682822443	6.698467242	6.667177643
10948	5.90029238	4.2543109	7.546273833	-0.352108471	-0.352108471	-0.352108471
10949	8.100408188	7.7274531	7.91393063	9.810882518	8.610864578	7.410846638
10950	5.89144105	6.4681519	7.044862685	5.772932242	6.667544955	6.220238598
10951	-0.352108471	-0.352108	-0.352108471	5.263851904	5.20849783	5.319205979
10952	-0.352108471	-0.352108	-0.352108471	6.944790218	7.643140836	8.341491454
10953	5.55876787	5.3063339	5.811201816	5.095423988	5.598414606	4.592433369
10954	-0.352108471	-0.352108	-0.352108471	4.237154657	3.119148782	5.355160532
10955	-0.352108471	-0.352108	-0.352108471	6.949901658	6.659392646	7.240410671
10956	7.342454581	6.5849872	8.099921982	5.931347273	8.819004217	7.375175745
10959	7.088053344	8.5564836	7.822268476	5.758739304	7.349917404	8.941095503
10960	4.910343046	5.9391922	6.968041397	8.215823114	5.895186222	7.055504668
10961	5.493783215	5.5293114	5.458255064	6.32531018	6.33538923	6.31523113
10963	7.504512579	8.5235906	8.014051606	7.000149166	7.009957676	7.019766185
10969	6.600014783	6.479333	6.720696571	7.326356364	7.342696275	7.310016454
10970	8.533806571	9.8410545	7.226558684	8.540082824	11.07483001	6.005335637
10971	6.575958791	5.0244408	5.800199788	8.816988496	8.754379576	8.879597416
10972	6.933588664	8.1348895	5.732287782	1.999631788	0.62969974	3.369563836
10975	6.519673958	6.5498529	6.489495002	7.787391935	7.811531416	7.763252455

10978	-0.352108471	-0.352108	-0.352108471	2.553073296	5.458255064	-0.352108471
10979	5.297171664	3.3339794	4.315575532	8.777194858	7.752449596	6.727704335
10980	-0.352108471	-0.352108	-0.352108471	7.152205469	7.604813664	8.057421859
10983	6.512012895	7.1489543	7.785895635	7.189846589	7.708132947	8.226419305
10984	13.56335825	15.361893	14.46262557	15.87876005	13.77892441	11.67908878
10985	5.152247113	8.2494846	2.055009582	6.707763664	5.983736712	5.259709759
10987	7.063413806	6.4887553	7.638072327	5.219123492	4.53752218	5.900724803
10988	5.493378529	4.6539182	6.332838881	10.43236039	2.840186115	6.636273252
10989	5.458403243	5.5291632	5.493783215	7.125111061	7.003799972	6.882488884
10992	5.596127468	7.1976093	6.396868383	8.029243459	9.202581337	6.855905582
11007	7.215278602	7.2517577	7.288236766	7.428892269	9.53404429	5.323740248
11010	7.259341956	7.5833616	6.935322328	7.69222184	5.723485516	3.754749191
11011	8.056536983	6.3703877	9.742686245	5.886342249	7.201489776	8.516637303
11014	7.416660475	7.4324764	7.424568434	7.387436362	6.760110864	7.073773613
11015	6.573968999	6.5990833	6.548854669	3.666436685	6.296043865	8.925651046
11017	-0.352108471	-0.352108	-0.352108471	8.089684608	7.271069954	6.4524553
11018	-0.352108471	-0.352108	-0.352108471	5.369332089	4.749761002	5.988903175
11019	5.939192221	6.8465105	5.031873987	7.975507869	5.19078168	2.406055492
11020	-0.352108471	-0.352108	-0.352108471	2.975135281	2.398531246	2.686833264
11027	4.615484867	5.2785295	4.947007189	-0.352108471	-0.352108471	-0.352108471
11030	-0.352108471	-0.352108	-0.352108471	6.034128238	6.928292805	7.822457372
11031	-0.352108471	-0.352108	-0.352108471	7.161815324	4.950292674	6.056053999
11034	9.769996878	6.9490395	8.359518179	10.7822818	8.69594998	6.609618165
11037	1.383520155	-0.352108	3.119148782	0.897715333	2.147539136	-0.352108471
11039	3.765118102	6.5407415	5.152929786	4.34040441	5.917122861	5.128763636
11040	5.50401475	7.073007	6.288510867	-0.352108471	-0.352108471	-0.352108471
11041	-0.352108471	-0.352108	-0.352108471	4.555577106	5.704335255	6.853093404
11043	-0.352108471	-0.352108	-0.352108471	5.455463676	5.388370172	5.321276667
11047	7.269153621	5.851102	8.687205203	6.357032901	8.171734534	9.986436167
11052	7.852413297	5.8101651	6.831289207	6.545950789	6.302547261	6.789354316
11054	8.869117574	6.9217481	4.974378711	5.142321764	5.383503645	4.901139884
11057	7.45286166	7.7234618	7.588161717	6.154712819	6.477305467	6.316009143
11061	4.947007189	4.655467	5.238547367	-0.352108471	-0.352108471	-0.352108471
11062	-0.352108471	-0.352108	-0.352108471	2.124237496	1.38954684	2.858928152
11065	-0.352108471	-0.352108	-0.352108471	2.811717702	8.104792426	5.458255064
11067	3.3648504	8.4357344	5.90029238	-0.352108471	-0.352108471	-0.352108471
11070	-0.352108471	-0.352108	-0.352108471	5.349381241	5.774058161	4.92470432
11072	-0.352108471	-0.352108	-0.352108471	4.914839551	7.516304119	10.11776869
11073	-0.352108471	-0.352108	-0.352108471	4.785420335	5.458255064	4.112585606
11075	-0.352108471	-0.352108	-0.352108471	4.783315286	5.637296679	6.491278072
11078	8.106647193	9.1681739	7.045120488	8.065239467	8.754472096	7.376006837
11079	-0.352108471	-0.352108	-0.352108471	7.605055129	7.827989767	7.382120491
11080	7.042523083	5.6436576	8.441388547	-0.352108471	-0.352108471	-0.352108471
11083	7.261520038	7.3440039	7.426487691	4.456886199	4.253005516	4.660766883
11086	5.354685649	7.157077	3.55229426	2.230275765	8.108039886	5.169157826
11091	6.607377459	6.6215439	6.593211042	5.017136436	5.054280952	4.979991919
11092	6.17064887	1.4077347	10.93356303	3.501376714	5.076027132	4.288701923

11094	6.090808341	7.4379003	4.743716385	-0.352108471	-0.352108471	-0.352108471
11096	-0.352108471	-0.352108	-0.352108471	4.378620115	4.66005769	4.941495264
11098	5.824288785	6.3630186	5.285558971	5.767743489	8.187620839	10.60749819
11100	7.251757684	6.0284557	8.475059698	7.677857161	8.389939172	8.033898167
11101	-0.352108471	-0.352108	-0.352108471	4.189671027	4.456886199	4.724101372
11102	3.855349974	6.1832159	5.019282955	1.12335747	0.808855576	1.437859363
11105	5.842203299	6.3112981	5.373108463	5.15430558	5.099234515	5.04416345
11108	-0.352108471	-0.352108	-0.352108471	5.407346691	7.064495631	6.235921161
11112	7.398193617	9.0806149	5.715772357	5.426431427	4.911884224	5.169157826
11116	8.620319187	5.469805	7.045062092	5.888251948	7.486200408	6.687226178
11117	8.841911097	8.5103335	8.178756	8.726834229	7.660833196	8.193833712
11118	7.710831709	4.6928925	6.20186208	1.916337486	1.731739118	2.100935855
11119	7.233420289	7.5059666	6.960873996	0.044132154	0.440372779	-0.352108471
11124	1.946552907	-0.352108	4.245214285	6.735779686	7.94416573	5.527393642
11128	-0.352108471	-0.352108	-0.352108471	6.717841276	4.697609333	8.738073218
11129	3.127737875	3.136327	3.119148782	2.211943219	2.232575625	2.191310813
11130	-0.352108471	-0.352108	-0.352108471	6.296043865	6.31523113	6.276856601
11133	3.797822384	5.6105004	1.985144396	2.929877893	3.012289909	3.094701926
11135	6.052917931	8.6859373	7.369427614	8.012846519	7.444308991	6.875771464
11136	-0.352108471	-0.352108	-0.352108471	0.822554576	1.631676003	2.440797431
11137	6.735779686	5.6237703	7.847789097	3.864023042	3.615460478	4.112585606
11140	8.1471131	6.3828735	4.618633854	5.156707362	7.747185018	10.33766267
11142	8.779772799	5.2498882	7.014830488	6.369156591	8.093274212	4.645038969
11148	5.073700926	7.6479784	6.360839663	6.998121009	9.629520821	4.366721198
11149	8.607405075	7.5030088	6.398612591	5.478987605	6.845389604	4.112585606
11152	7.650812904	7.8275848	7.474040996	6.349497303	6.174080069	6.524914536
11156	5.152247113	4.3650797	5.93941455	-0.352108471	-0.352108471	-0.352108471
11157	5.089412202	5.1245435	5.054280952	-0.352108471	-0.352108471	-0.352108471
11159	3.693630547	5.1227357	4.408183111	0.228373553	-0.352108471	0.808855576
11163	5.793573715	1.2367088	3.515141236	0.849500091	-0.352108471	2.051108653
11164	-0.352108471	-0.352108	-0.352108471	6.724469766	6.759061079	6.689878453
11167	10.79247225	9.6111024	8.42973264	7.588834184	12.72384535	10.15633977
11168	-0.352108471	-0.352108	-0.352108471	10.32735114	2.323269217	6.32531018
11169	5.852937462	5.8097505	5.896124426	6.415928975	3.228559088	9.603298861
11171	0.531669581	2.2391587	3.94664787	-0.352108471	-0.352108471	-0.352108471
11174	6.720370627	6.7508628	6.689878453	5.234766675	6.038453511	4.43107984
11176	-0.352108471	-0.352108	-0.352108471	6.6969269	8.734416533	7.715671716
11178	5.970245565	3.8449111	8.095580024	0.044132154	-0.352108471	0.440372779
11179	3.127737875	3.6552986	2.600177152	7.120691864	8.026083043	6.215300685
11180	6.242048279	5.3849084	7.099188177	0.762376451	-0.352108471	1.876861374
11181	4.904572896	6.3290035	3.480142317	-0.352108471	-0.352108471	-0.352108471
11183	6.137818553	5.9742894	6.056053999	5.284974356	5.315258837	5.254689875
11185	7.511491784	7.8525094	7.682000613	8.216186052	6.138007963	7.177097008
11186	-0.352108471	-0.352108	-0.352108471	5.930418595	6.296043865	6.661669136
11188	-0.352108471	-0.352108	-0.352108471	5.323929678	8.748862375	7.036396027
11189	-0.352108471	-0.352108	-0.352108471	3.680762464	8.363339386	6.022050925
11190	6.336590478	8.5049166	4.168264361	-0.352108471	-0.352108471	-0.352108471

11191	6.303461245	6.9069074	5.70001505	-0.352108471	-0.352108471	-0.352108471
11193	-0.352108471	-0.352108	-0.352108471	6.12990004	6.893309616	5.366490464
11194	7.231594464	5.4937832	3.755971966	5.789762726	6.120169646	5.459355806
11196	6.553359934	6.9908132	7.428266385	3.252081944	4.163121594	2.341042294
11198	4.016045771	4.981965	3.050126516	6.898982206	6.406486527	5.913990848
11200	1.536298775	2.3147854	2.369587412	-0.352108471	-0.352108471	-0.352108471
11212	-0.352108471	-0.352108	-0.352108471	5.158940678	5.492491478	5.325716078
11213	5.275550329	4.8956643	5.085607333	3.745997314	4.769034835	2.722959794
11214	-0.352108471	-0.352108	-0.352108471	5.094129627	6.180463731	5.637296679
11215	3.127737875	2.4920726	3.763403121	5.796604305	4.541711346	5.169157826
11218	-0.352108471	-0.352108	-0.352108471	5.704335255	5.715557343	5.693113166
11222	-0.352108471	-0.352108	-0.352108471	8.916076408	4.533253635	6.724665022
11224	9.595884353	8.0488847	11.14288406	10.18593386	11.04529834	9.326569381
11226	7.148431247	6.9178841	7.37897843	5.276825162	8.007666442	6.642245802
11227	-0.352108471	-0.352108	-0.352108471	6.750973351	6.497182024	6.243390698
11228	5.043054533	8.4660625	6.754558526	-0.352108471	-0.352108471	-0.352108471
11230	-0.352108471	-0.352108	-0.352108471	8.151859699	8.292450792	8.222155246
11234	-0.352108471	-0.352108	-0.352108471	2.204376426	-0.352108471	4.760861323
11237	-0.352108471	-0.352108	-0.352108471	2.567556948	4.602685654	3.585121301
11243	-0.352108471	-0.352108	-0.352108471	6.679398756	8.721837549	4.636959963
11244	-0.352108471	-0.352108	-0.352108471	4.09022467	6.686515673	5.388370172
11245	3.127737875	3.2744025	2.981073213	5.340345662	6.51496239	7.689579119
11252	-0.352108471	-0.352108	-0.352108471	6.098057327	6.150515881	6.045598773
11253	-0.352108471	-0.352108	-0.352108471	6.293823158	4.658849082	5.47633612
11257	-0.352108471	-0.352108	-0.352108471	5.457998494	5.421579811	5.494417177
11258	6.497621264	6.3691566	6.240691917	5.759925606	5.804293869	5.715557343
11259	9.75740385	6.3968684	3.036332915	7.261737295	9.076451528	8.169094411
11260	8.706557066	4.6417007	6.674128879	-0.352108471	-0.352108471	-0.352108471
11261	7.548006705	5.8282467	9.267766661	7.393006279	7.496586827	7.600167375
11262	8.431877632	6.6073775	4.782877286	-0.352108471	-0.352108471	-0.352108471
11264	8.953660204	8.8575184	9.04980201	7.7927523	7.716178874	7.869325725
11267	-0.352108471	-0.352108	-0.352108471	7.458282885	4.508868586	10.40769718
11273	7.771588187	4.8933452	6.332466669	5.813397253	7.05168679	6.432542021
11276	8.76655368	6.8161979	4.865842198	5.516329242	6.912428792	8.308528341
11277	3.02698104	5.8242888	8.621596529	6.100981295	6.893382753	6.497182024
11280	-0.352108471	-0.352108	-0.352108471	3.720557712	2.921957572	2.123357432
11282	7.547345435	7.1250675	6.702789566	7.911746015	6.875966205	8.947525825
11283	5.291399258	5.4582551	5.124543452	-0.352108471	-0.352108471	-0.352108471
11284	-0.352108471	-0.352108	-0.352108471	6.087644454	6.101634422	6.073654487
11285	6.151585628	4.6029687	7.70020258	0.61505922	2.133542422	1.374300821
11311	5.939192221	7.6401003	4.238284093	-0.352108471	-0.352108471	-0.352108471
11313	4.047998928	4.8786655	5.709332029	7.032433877	5.723485516	4.414537155
11315	5.951591022	5.6886226	5.425654272	8.236263963	8.832919693	7.639608232
11316	5.432975716	8.8916995	7.162337587	6.252567117	10.11190906	8.182238087
11320	9.502310428	7.6279944	8.56515243	6.944720985	10.06502239	3.824419582
11321	-0.352108471	-0.352108	-0.352108471	5.457665298	6.596757815	7.735850333
11322	-0.352108471	-0.352108	-0.352108471	2.891547989	6.135204448	-0.352108471

11328	4.06596187	4.5241994	4.295080645	5.316008865	8.33381536	6.824912113
11329	-0.352108471	-0.352108	-0.352108471	8.270942143	4.758982638	6.51496239
11330	6.636323465	7.8297725	9.023221613	7.419575248	4.275131129	10.56401937
11331	-0.352108471	-0.352108	-0.352108471	5.637296679	6.103094337	5.17149902
11332	3.72461963	4.0641048	3.894362219	7.113011701	5.47907603	6.296043865
11333	7.916001466	7.2266559	6.537310422	8.285380395	8.531441672	8.777502949
11334	-0.352108471	-0.352108	-0.352108471	7.468827193	5.98050285	6.724665022
11335	6.090808341	6.1079622	6.073654487	3.119148782	3.119148782	3.119148782
11337	6.68088213	6.9786128	6.383151496	7.8557263	7.827584812	7.883867788
11338	6.818986369	6.8576112	6.896236025	8.132440628	6.118807471	10.14607378
11340	-0.352108471	-0.352108	-0.352108471	6.312019947	7.036396027	7.760772106
11343	4.429949807	3.136327	5.723572646	7.395526103	6.367936567	8.423115639
11344	6.767634029	6.6898785	6.845389604	7.100428707	7.327812407	6.873045007
22794	3.400971508	5.054281	6.707590396	2.685883497	2.751563581	2.620203413
22796	-0.352108471	-0.352108	-0.352108471	6.12990004	6.135204448	6.124595632
22800	-0.352108471	-0.352108	-0.352108471	5.095423988	4.533492242	5.657355734
22801	7.05393529	5.9629858	8.144884797	7.262668404	8.076280549	8.889892695
22803	-0.352108471	-0.352108	-0.352108471	5.575983727	5.588224375	5.563743079
22806	8.774572407	8.0637228	9.485422006	8.810644235	7.079106891	7.944875563
22807	-0.352108471	-0.352108	-0.352108471	3.119148782	5.659140661	0.579156902
22808	-0.352108471	-0.352108	-0.352108471	5.461162703	4.66005769	3.858952676
22809	2.513339833	0.4886953	1.501017585	6.745152057	7.232708855	6.25759526
22818	-0.352108471	-0.352108	-0.352108471	5.069628453	7.112080183	9.154531912
22820	6.650702363	6.3538237	6.502263028	10.05931917	7.082269275	4.105219384
22821	-0.352108471	-0.352108	-0.352108471	6.262494648	6.428056267	6.345275458
22822	8.828377374	7.3217512	5.815124983	5.504438215	3.228350737	7.780525694
22826	2.063269944	7.1678971	4.615583513	7.921737327	6.534540863	7.228139095
22827	5.90068335	4.9841517	4.067620061	6.74407332	8.271827838	5.216318802
22828	-0.352108471	-0.352108	-0.352108471	6.433214732	5.958359385	5.483504037
22834	6.06950748	5.3512458	5.710376656	1.988898614	2.100935855	1.876861374
22836	5.398878921	7.0635004	6.231189673	8.280622695	8.120985773	7.961348851
22837	6.603683995	4.7735613	5.688622647	-0.352108471	-0.352108471	-0.352108471
22838	5.555777437	5.6532998	5.458255064	-0.352108471	-0.352108471	-0.352108471
22839	6.415928975	6.1928009	6.639057057	8.413413636	2.901574126	5.657493881
22841	5.980334028	6.7313264	5.229341628	-0.352108471	-0.352108471	-0.352108471
22844	7.179763278	9.2242692	8.202016251	-0.352108471	-0.352108471	-0.352108471
22845	-0.352108471	-0.352108	-0.352108471	6.747271561	6.773846551	6.720696571
22846	-0.352108471	-0.352108	-0.352108471	8.404182358	4.361564596	6.382873477
22848	7.951873779	9.2014373	10.45100074	9.801249887	5.584100902	7.692675395
22850	-0.352108471	-0.352108	-0.352108471	2.686833264	2.686833264	2.686833264
22853	-0.352108471	-0.352108	-0.352108471	1.968661759	1.965500585	1.971822932
22858	5.857260806	5.5954997	5.333738511	2.26457781	2.523191309	2.393884559
22861	-0.352108471	-0.352108	-0.352108471	2.18764845	1.383520155	0.579391861
22862	-0.352108471	-0.352108	-0.352108471	7.600205102	4.119029636	5.859617369
22863	6.658023207	5.7516583	7.564388073	4.420541739	4.197167339	4.643916139
22864	5.658752616	4.4996979	3.340643085	4.977732308	6.616664157	8.255596007
22866	-0.352108471	-0.352108	-0.352108471	6.382873477	8.529349449	4.236397505

22868	6.972071229	5.5398727	6.255971957	4.424398591	2.212230095	3.318314343
22870	8.29082699	6.8469571	5.403087204	6.51496239	8.246536643	4.783388138
22872	3.187894448	5.1522471	7.116599779	10.13933326	6.567704622	8.353518943
22873	1.539693917	3.2452353	4.950776693	2.334436445	3.379562464	1.289310425
22874	-0.352108471	-0.352108	-0.352108471	3.682181533	3.563511762	3.800851305
22876	2.681724436	1.0692497	4.294199159	-0.352108471	-0.352108471	-0.352108471
22877	5.908221573	5.5882244	6.228218771	6.442231866	6.246629235	6.637834496
22883	1.695655193	2.4349289	2.065292044	7.478001079	10.29955492	8.888778001
22884	-0.352108471	-0.352108	-0.352108471	6.344326921	5.458255064	4.572183207
22885	5.493783215	5.5293114	5.458255064	6.713776402	6.729085562	6.698467242
22887	10.41246047	7.3781729	4.343885236	6.668865983	5.449706766	7.888025199
22888	6.570764467	6.6691367	6.767508926	6.440256725	6.373952196	6.307647666
22889	-0.352108471	-0.352108	-0.352108471	5.20089139	5.254689875	5.147092904
22890	5.354685649	5.3835036	5.325867653	6.396868383	6.410585269	6.383151496
22894	5.499603697	3.1740837	4.336843676	5.820435134	6.309958134	6.065196634
22897	5.354685649	6.0955569	4.613814437	-0.352108471	-0.352108471	-0.352108471
22900	5.626802631	5.4423851	5.81122012	1.76984594	1.731739118	1.807952763
22904	2.553073296	0.2069451	4.899201485	5.29818808	6.056053999	6.813919918
22906	5.637563121	6.7052875	7.773011902	4.272763834	8.119995301	6.196379568
22907	-0.352108471	-0.352108	-0.352108471	3.906425881	3.727916258	3.549406635
22911	6.801813774	6.8648849	6.738742641	-0.352108471	-0.352108471	-0.352108471
22913	7.622518643	5.8179612	9.427076069	7.827924462	7.846725922	7.865527382
22915	4.901506784	5.2437595	4.559254036	6.31523113	6.31523113	6.31523113
22919	6.962610801	7.9989043	9.035197794	8.267255306	7.223970505	9.310540107
22924	6.490304795	1.5874548	4.038879774	6.266367194	5.334256671	7.198477716
22925	-0.352108471	-0.352108	-0.352108471	4.741335279	5.516191992	5.128763636
22926	6.305519736	6.2558778	6.355161685	5.747617149	5.421579811	6.073654487
22930	-0.352108471	-0.352108	-0.352108471	6.377287067	6.032025449	6.204656258
22931	-0.352108471	-0.352108	-0.352108471	2.274515706	-0.352108471	4.901139884
22933	3.127737875	3.136327	3.119148782	5.760537073	5.775231583	5.745842563
22936	5.061176021	7.8054614	6.433318728	6.532441983	6.549852913	6.515031054
22937	-0.352108471	-0.352108	-0.352108471	5.128763636	7.00964221	3.247885062
22938	7.259341956	7.2774479	7.241236027	6.022050925	6.038453511	6.005648339
22943	7.787414058	8.1860488	7.986731438	10.50543536	7.587817397	9.046626379
22944	4.658482176	5.6736962	6.688910282	3.715877345	4.8336904	4.274783872
22948	8.663537	8.6927236	8.634350444	8.481446383	8.489801751	8.473091016
22949	3.415477276	5.4500085	7.484539702	-0.352108471	-0.352108471	-0.352108471
22955	-0.352108471	-0.352108	-0.352108471	8.423147771	3.023823261	5.723485516
22974	2.65059567	1.8977565	3.403434816	8.695100112	7.273767717	5.852435321
22978	4.617382386	5.3152588	3.919505936	7.918642203	7.927022814	7.910261593
22980	8.057301306	6.8610575	5.664813682	6.952707338	5.251385446	8.65402923
22981	5.852937462	5.9030349	5.802839999	-0.352108471	-0.352108471	-0.352108471
22982	6.901979267	6.9037827	6.900175883	-0.352108471	-0.352108471	-0.352108471
22983	6.920328612	5.1917794	6.056053999	0.044132154	-0.352108471	0.440372779
22984	-0.352108471	-0.352108	-0.352108471	6.74407332	5.258887764	8.229258876
22985	3.127737875	2.7683414	3.487134364	6.693937107	8.074926929	5.312947285
22987	4.871233248	4.617923	5.124543452	4.00104605	5.315258837	2.686833264

22990	-0.352108471	-0.352108	-0.352108471	6.022050925	6.038453511	6.005648339
22992	-0.352108471	-0.352108	-0.352108471	5.923493182	6.436231581	5.410754783
22997	4.784443809	5.0755196	5.366595308	-0.352108471	-0.352108471	-0.352108471
23002	3.202588505	1.7605619	2.481575183	-0.352108471	-0.352108471	-0.352108471
23005	8.40798568	3.2784882	5.843236918	0.875857225	2.276479193	3.67710116
23008	-0.352108471	-0.352108	-0.352108471	6.422125394	5.325716078	4.229306762
23011	7.620813645	6.264091	8.977536258	7.306531223	7.21486974	7.398192705
23013	6.396868383	8.6656367	4.128100051	7.738656439	6.235921161	4.733185883
23014	-0.352108471	-0.352108	-0.352108471	4.80208491	7.154500842	2.449668978
23017	6.444344941	8.3302157	7.387280312	-0.352108471	-0.352108471	-0.352108471
23020	5.748554906	3.1277379	0.506920845	6.956278745	6.83357915	7.078978339
23022	5.661877241	4.2321371	4.947007189	7.575129529	9.397748895	5.752510164
23024	-0.352108471	-0.352108	-0.352108471	4.66005769	4.688318557	4.631796822
23028	6.621375333	7.3143303	8.007285247	6.396868383	4.325965404	8.467771361
23030	3.510791208	7.2358917	10.96099212	6.261817617	6.479200813	6.044434421
23031	5.454270861	4.7989575	4.143644102	6.721060945	6.803648407	6.762354676
23032	10.53707414	5.5994466	8.068260349	6.420072833	7.981021082	4.859124585
23034	-0.352108471	-0.352108	-0.352108471	5.851704574	6.151913688	6.001809131
23035	-0.352108471	-0.352108	-0.352108471	2.515697587	5.383503645	-0.352108471
23036	5.769132707	8.2644201	7.016776417	4.297639083	4.482692559	4.112585606
23037	6.515350569	7.1472278	6.831289207	-0.352108471	-0.352108471	-0.352108471
23038	6.144738623	7.2078382	8.2709377	4.927679215	6.266163582	3.589194848
23039	6.129154436	7.2191033	6.674128879	7.962914419	4.965345804	1.967777189
23041	6.441863207	8.1717493	4.711977071	-0.352108471	-0.352108471	-0.352108471
23042	4.684878868	3.9090457	5.460712065	1.935130509	2.274515706	2.613900903
23046	2.201036472	-0.352108	4.754181416	-0.352108471	-0.352108471	-0.352108471
23048	6.114636752	5.0894122	4.064187652	3.725166081	2.420433935	5.029898226
23049	8.033846106	7.511077	8.556615201	7.284298749	6.926801914	7.641795584
23051	8.098323384	6.2634999	7.180911636	5.934798323	4.482946372	7.386650274
23053	-0.352108471	-0.352108	-0.352108471	5.375686461	6.202145189	7.028603916
23054	4.990673431	5.3257161	5.660758725	-0.352108471	-0.352108471	-0.352108471
23059	7.362898191	7.1903972	7.535399176	6.452415203	7.291996879	5.612833527
23061	7.4755167	4.2303582	5.852937462	6.995370627	5.655249734	6.32531018
23062	8.077501205	7.5271777	6.976854187	3.723847293	2.686833264	4.760861323
23064	5.156223819	4.8780491	5.017136436	-0.352108471	-0.352108471	-0.352108471
23065	4.341685584	6.6400194	8.938353202	-0.352108471	-0.352108471	-0.352108471
23067	-0.352108471	-0.352108	-0.352108471	5.945877642	5.637296679	5.328715715
23070	-0.352108471	-0.352108	-0.352108471	4.927679215	5.162677265	4.692681165
23071	-0.352108471	-0.352108	-0.352108471	6.682822443	6.698467242	6.667177643
23075	5.884825072	9.4534818	2.316168355	4.575701584	8.40294373	6.489322657
23076	6.735779686	6.7508628	6.720696571	-0.352108471	-0.352108471	-0.352108471
23080	7.044142848	7.1881098	6.900175883	0.849500091	-0.352108471	2.051108653
23081	6.449754253	6.4168832	6.433318728	5.457401074	4.576871797	5.017136436
23085	4.590734517	3.3976438	2.204553096	4.493074164	6.915596346	5.704335255
23086	9.36812472	9.3688497	9.369574686	8.149457474	7.996298794	8.302616153
23087	-0.352108471	-0.352108	-0.352108471	4.80208491	3.385507109	6.218662712
23091	4.178899946	3.7191088	4.638691089	6.022050925	4.612133582	7.431968268

23092	5.816819807	9.7696384	7.793229106	6.730179436	6.844515667	6.958851898
23093	-0.352108471	-0.352108	-0.352108471	4.927679215	4.954218546	4.901139884
23094	-0.352108471	-0.352108	-0.352108471	1.842407068	1.807952763	1.876861374
23095	7.676556837	6.0206564	4.364755958	6.768161686	8.267506524	7.517834105
23096	-0.352108471	-0.352108	-0.352108471	2.076022254	2.100935855	2.051108653
23099	6.699669623	7.0441776	6.355161685	6.191192686	5.653299811	6.729085562
23102	-0.352108471	-0.352108	-0.352108471	8.138734114	6.481523945	4.824313775
23105	6.537383725	5.4646719	7.610095507	-0.352108471	-0.352108471	-0.352108471
23107	5.346983862	5.3551833	5.363382639	4.312928258	4.930625625	4.621776941
23112	4.439235342	5.597852	6.756468726	8.328883636	6.461840164	4.594796692
23113	6.637046067	7.7648689	5.509223186	-0.352108471	-0.352108471	-0.352108471
23119	6.1067292	5.7049355	5.303141868	-0.352108471	-0.352108471	-0.352108471
23120	6.15154247	6.9134889	7.675435326	-0.352108471	-0.352108471	-0.352108471
23125	-0.352108471	-0.352108	-0.352108471	9.735552686	3.209800195	6.47267644
23126	6.178645924	5.4217537	5.800199788	2.301055037	3.89409388	0.708016195
23129	4.867589503	4.7140092	4.560428825	0.379002596	6.243388056	3.311195326
23131	2.659511827	3.5959639	3.127737875	8.082343149	7.209478834	6.336614519
23132	0.709954536	3.3238738	2.016914154	8.492224117	2.862255241	5.677239679
23135	-0.352108471	-0.352108	-0.352108471	6.022050925	4.149359872	7.894741978
23137	4.137290146	5.2492247	6.361159261	6.758889758	6.71093492	6.662980082
23138	5.614838129	4.7841331	6.445543199	-0.352108471	-0.352108471	-0.352108471
23139	5.677560781	8.4968049	7.08718283	2.176699173	2.021948617	2.099323895
23140	6.521624659	4.7286969	5.625160771	6.149337851	6.686595003	5.612080698
23144	5.55876787	7.339779	3.777756753	6.369156591	7.993983431	4.74432975
23145	5.284974356	4.8994396	5.670509125	4.328694932	7.536005664	5.932350298
23148	11.18910833	1.9588297	6.573968999	5.812004781	8.391327579	7.10166618
23150	-0.352108471	-0.352108	-0.352108471	4.079365824	2.694427604	1.309489384
23152	8.386872366	7.1377274	5.888582458	6.053934119	5.139899864	5.596916991
23155	6.878890016	6.4712501	6.06361009	3.172580615	3.444533984	2.900627247
23158	5.903499469	5.4937832	5.084066961	5.903760504	8.957594003	2.849927005
23160	1.383520155	-0.352108	3.119148782	6.128525768	7.054600764	7.980675761
23161	-0.352108471	-0.352108	-0.352108471	6.113115027	7.003139405	5.223090649
23162	5.376287016	4.3810439	4.878665478	1.223721097	1.305230684	1.14221151
23163	3.83572752	4.5503078	5.264888084	6.311056085	5.821410838	5.33176559
23164	7.378762478	7.9301228	7.654442651	8.07411195	8.355515917	8.636919885
23166	-0.352108471	-0.352108	-0.352108471	1.631676003	3.615460478	-0.352108471
23167	-0.352108471	-0.352108	-0.352108471	5.88700099	3.227651827	8.546350153
23168	5.745394463	5.7155573	5.775231583	4.580195866	5.605609608	3.554782125
23170	6.87100449	6.521105	7.220903987	-0.352108471	-0.352108471	-0.352108471
23171	-0.352108471	-0.352108	-0.352108471	3.235355345	2.211943219	1.188531093
23173	7.346824737	4.5315597	5.939192221	-0.352108471	-0.352108471	-0.352108471
23175	1.383520155	-0.352108	3.119148782	6.32531018	6.33538923	6.31523113
23176	-0.352108471	-0.352108	-0.352108471	4.943286	6.813211995	5.878248998
23177	8.300766402	8.4746689	8.648571346	5.233567424	3.897234107	6.56990074
23178	-0.352108471	-0.352108	-0.352108471	2.002763867	1.997577452	1.992391037
23180	9.013050438	7.3139841	5.614917825	-0.352108471	-0.352108471	-0.352108471
23181	1.383520155	-0.352108	3.119148782	3.245625856	3.337746555	3.153505157

23184	-0.352108471	-0.352108	-0.352108471	4.456886199	4.482692559	4.43107984
23185	4.944495873	6.4327494	5.688622647	4.967642314	7.149216888	9.330791462
23186	-0.352108471	-0.352108	-0.352108471	6.165870606	5.76850177	6.563239442
23187	-0.352108471	-0.352108	-0.352108471	4.667242547	6.926938623	9.186634699
23190	-0.352108471	-0.352108	-0.352108471	6.296043865	4.939040992	7.653046739
23191	-0.352108471	-0.352108	-0.352108471	6.878850027	6.729085562	7.028614492
23193	5.846368804	7.8162096	6.831289207	7.463736672	5.220915492	9.706557853
23195	5.740399084	6.7052875	7.67017594	3.818060928	5.383503645	2.252618212
23196	-0.352108471	-0.352108	-0.352108471	7.329088911	5.959950203	8.698227619
23197	-0.352108471	-0.352108	-0.352108471	3.210996379	4.178899946	5.146803513
23199	-0.352108471	-0.352108	-0.352108471	7.044177561	7.802689174	6.285665948
23203	-0.352108471	-0.352108	-0.352108471	6.609543024	7.964843359	5.254242688
23204	2.977960136	3.8056644	4.63336857	9.522895775	3.84274911	6.682822443
23207	7.305341479	7.3178812	7.292801747	6.826859584	7.528959936	8.231060288
23208	1.66985715	-0.352108	3.69182277	8.994503386	4.395178474	6.69484093
23210	-0.352108471	-0.352108	-0.352108471	7.186511196	7.204087928	7.168934463
23211	6.332466669	7.661227	5.003706387	6.12990004	6.964019301	5.29578078
23213	8.190131516	7.0940219	9.286241145	7.885401903	8.509613549	9.133825194
23214	3.286524408	5.5587679	7.831011332	6.927461799	7.856668635	7.392065217
23215	4.247086177	6.3324667	8.41784716	-0.352108471	-0.352108471	-0.352108471
23216	6.85672293	5.3863346	6.121528769	4.237354153	5.859617369	7.481880585
23220	-0.352108471	-0.352108	-0.352108471	1.946552907	0.268308258	3.624797556
23223	2.740210233	-0.352108	5.832528937	-0.352108471	-0.352108471	-0.352108471
23224	5.90029238	6.272065	5.528519803	-0.352108471	-0.352108471	-0.352108471
23225	5.753125775	6.7645426	6.258834195	-0.352108471	-0.352108471	-0.352108471
23232	3.188365197	8.9932515	6.090808341	-0.352108471	-0.352108471	-0.352108471
23233	-0.352108471	-0.352108	-0.352108471	5.043435488	7.288305724	6.165870606
23234	7.633287415	5.5146506	6.573968999	4.400642798	7.120431348	5.760537073
23239	5.506125303	5.8124324	5.659278871	-0.352108471	-0.352108471	-0.352108471
23240	7.202880948	7.1358739	7.269888009	9.1428279	6.288499729	3.434171559
23241	7.841606393	6.9795601	6.117513834	8.175956815	7.50587715	6.835797485
23242	-0.352108471	-0.352108	-0.352108471	4.857539934	4.49146636	5.223613509
23244	5.260515072	6.6235409	7.986566794	-0.352108471	-0.352108471	-0.352108471
23245	11.63330029	11.222625	12.04397532	9.950532901	10.71396365	11.4773944
23246	6.573968999	7.4862118	5.6617262	1.90830372	1.939746065	1.876861374
23248	4.220535928	5.8476283	7.474720713	1.703584312	0.743857882	1.223721097
23251	5.852937462	5.1208337	6.58504124	-0.352108471	-0.352108471	-0.352108471
23254	-0.352108471	-0.352108	-0.352108471	2.065292044	3.449619376	0.680964713
23255	-0.352108471	-0.352108	-0.352108471	7.498611778	4.391025634	5.944818706
23256	5.428393874	4.7624113	6.09437648	8.156749636	7.357359337	6.557969039
23258	5.97539282	6.6315297	6.303461245	-0.352108471	-0.352108471	-0.352108471
23263	-0.352108471	-0.352108	-0.352108471	6.266367194	8.502791965	4.029942422
23264	-0.352108471	-0.352108	-0.352108471	7.949797171	9.282264384	6.617329957
23265	-0.352108471	-0.352108	-0.352108471	5.760537073	8.307561902	3.213512244
23268	-0.352108471	-0.352108	-0.352108471	5.983736712	4.952037565	7.015435859
23270	-0.352108471	-0.352108	-0.352108471	6.450302837	5.927150083	6.973455592
23271	-0.352108471	-0.352108	-0.352108471	1.946552907	4.245214285	-0.352108471

23272	7.368929106	7.0737094	6.778489705	7.069096541	5.715557343	4.362018146
23275	-0.352108471	-0.352108	-0.352108471	5.936972408	5.017136436	4.097300463
23276	-0.352108471	-0.352108	-0.352108471	5.958359385	5.958359385	5.958359385
23277	6.846957097	8.5599964	5.13391777	4.161895069	5.529311366	6.896727662
23283	-0.352108471	-0.352108	-0.352108471	3.492690609	4.865109282	4.178899946
23284	-0.352108471	-0.352108	-0.352108471	6.128312408	4.904478682	3.680644956
23288	7.375407987	7.904095	8.432781997	-0.352108471	-0.352108471	-0.352108471
23291	-0.352108471	-0.352108	-0.352108471	5.686745699	4.651569952	5.169157826
23293	7.757296035	9.8233685	5.691223527	5.410222762	7.654233331	6.532228046
23295	2.66769987	7.7721024	5.219901144	4.72458994	5.58495468	3.8642252
23299	-0.352108471	-0.352108	-0.352108471	4.892537618	5.859617369	6.82669712
23300	-0.352108471	-0.352108	-0.352108471	5.914934646	5.073899708	5.494417177
23301	-0.352108471	-0.352108	-0.352108471	5.369332089	5.100443381	5.638220796
23303	-0.352108471	-0.352108	-0.352108471	5.095423988	5.124543452	5.066304524
23304	5.749402114	2.6565569	8.84224728	2.947695678	10.33657302	6.64213435
23305	7.987390912	8.6668076	7.307974228	6.392464334	5.962319293	5.532174252
23306	2.65059567	5.6532998	-0.352108471	-0.352108471	-0.352108471	-0.352108471
23307	6.502263028	4.7891524	8.215373684	-0.352108471	-0.352108471	-0.352108471
23309	-0.352108471	-0.352108	-0.352108471	3.447274322	3.585121301	3.722968281
23310	5.861579664	5.5293114	6.193847962	-0.352108471	-0.352108471	-0.352108471
23313	6.189714237	5.5259162	4.862118258	4.9405116	6.811810265	8.683108931
23315	6.167966808	6.5187174	5.817216208	7.949451176	6.104429468	4.25940776
23317	8.12788013	7.4432023	8.812557987	-0.352108471	-0.352108471	-0.352108471
23321	6.404970197	5.9984064	5.591842699	-0.352108471	-0.352108471	-0.352108471
23324	-0.352108471	-0.352108	-0.352108471	7.753529229	9.324769412	6.182289045
23326	6.927337071	4.5195292	9.335144928	7.824184932	7.948836079	7.886510506
23327	3.534670788	5.2655441	6.996417412	7.572971681	3.359882049	5.466426865
23328	6.886324359	7.1464808	6.626167961	3.470468384	4.688318557	2.252618212
23331	8.346425764	5.2218548	6.784140258	1.493791735	1.437859363	1.549724107
23332	2.8251001	-0.352108	6.002308672	-0.352108471	-0.352108471	-0.352108471
23333	-0.352108471	-0.352108	-0.352108471	6.280785144	7.7016594	6.991222272
23334	7.042510888	7.9376183	7.490064594	1.967011497	4.20202554	6.437039583
23338	-0.352108471	-0.352108	-0.352108471	6.204656258	5.770235089	6.639077427
23339	-0.352108471	-0.352108	-0.352108471	3.485299609	8.6899893	6.087644454
23344	7.398193617	5.2796653	9.516721893	0.882835079	1.689109503	2.495383927
23348	7.764477324	5.3102901	6.537383725	3.20802376	8.83607809	6.022050925
23349	-0.352108471	-0.352108	-0.352108471	6.220238598	4.582161095	7.858316102
23350	-0.352108471	-0.352108	-0.352108471	6.136859819	6.497182024	6.857504229
23351	6.609550563	6.056054	5.502557435	-0.352108471	-0.352108471	-0.352108471
23352	2.588601447	-0.352108	5.529311366	-0.352108471	-0.352108471	-0.352108471
23360	-0.352108471	-0.352108	-0.352108471	4.096638726	5.596916991	7.097195256
23363	-0.352108471	-0.352108	-0.352108471	7.358324194	7.885711656	7.622017925
23365	7.101491044	6.430493	7.772489067	6.886610445	6.900175883	6.873045007
23367	-0.352108471	-0.352108	-0.352108471	7.591455381	10.81107722	4.371833546
23369	5.089412202	5.1245435	5.054280952	6.434644758	6.448016836	6.421272681
23370	4.680024204	7.5615971	6.120810648	-0.352108471	-0.352108471	-0.352108471
23371	-0.352108471	-0.352108	-0.352108471	4.498245804	7.641842129	6.070043966

23373	-0.352108471	-0.352108	-0.352108471	0.881271924	1.848794271	2.816316617
23379	5.939192221	5.9583594	5.920025058	-0.352108471	-0.352108471	-0.352108471
23380	-0.352108471	-0.352108	-0.352108471	5.704335255	5.715557343	5.693113166
23381	5.694288095	5.463922	5.233555955	-0.352108471	-0.352108471	-0.352108471
23383	5.919602486	6.3691566	6.818710695	3.119148782	1.001288989	5.237008574
23384	-0.352108471	-0.352108	-0.352108471	6.456300849	5.803499231	6.12990004
23385	-0.352108471	-0.352108	-0.352108471	7.237322918	7.661684316	6.81296152
23386	5.776028412	6.6895379	7.603047463	-0.352108471	-0.352108471	-0.352108471
23392	6.706547766	6.056054	5.405560232	3.325116882	6.530241548	4.927679215
23394	6.071632948	6.075676	6.073654487	0.044132154	0.440372779	-0.352108471
23395	6.502263028	6.5150311	6.489495002	1.493791735	1.549724107	1.437859363
23396	-0.352108471	-0.352108	-0.352108471	5.960229497	3.154259885	4.557244691
23399	4.966655053	5.8529375	6.739219871	8.199266326	9.55849577	6.840036882
23400	-0.352108471	-0.352108	-0.352108471	6.835443563	6.747271561	6.659099559
23403	7.063413806	5.1342152	8.992612367	-0.352108471	-0.352108471	-0.352108471
23404	6.561809811	5.1425483	3.723286822	3.867457035	5.926961306	1.807952763
23405	-0.352108471	-0.352108	-0.352108471	6.913488898	6.816764603	7.010213194
23406	8.213306202	5.8414453	7.027375773	9.249434818	7.756104605	10.74276503
23410	3.667916057	4.0823601	4.496804164	3.645602413	1.524655022	2.585128717
23411	5.428393874	5.4582551	5.398532684	-0.352108471	-0.352108471	-0.352108471
23412	-0.352108471	-0.352108	-0.352108471	1.737679284	7.866490536	4.80208491
23413	5.688622647	5.7155573	5.661687951	5.663737322	5.665786693	5.661687951
23414	6.433318728	5.2387634	7.627874068	0.044132154	0.440372779	-0.352108471
23420	-0.352108471	-0.352108	-0.352108471	2.139844176	4.257742579	0.021945772
23426	-0.352108471	-0.352108	-0.352108471	7.535571763	4.724228317	6.12990004
23431	7.558979541	7.2513033	6.943627137	5.420703748	6.731862502	8.043021255
23432	5.765954776	2.6556616	8.876247922	3.772616852	6.186673578	4.979645215
23433	6.180304537	3.2056737	4.692989119	8.807209491	5.701860041	7.254534766
23434	5.391018164	5.3835036	5.398532684	0.793818797	-0.352108471	1.939746065
23435	7.137727412	7.1464808	7.128974067	-0.352108471	-0.352108471	-0.352108471
23439	7.681952154	6.0729503	6.87745121	5.726388281	6.415773968	5.037002593
23443	6.754322418	6.6608845	6.707603469	5.951862052	6.38294239	5.520781713
23446	2.961764703	8.3965748	5.679169776	5.937584898	6.120364579	5.754805217
23450	7.323359936	8.6788647	8.001112307	7.305876064	5.974196817	8.637555311
23452	2.685732087	-0.352108	5.723572646	-0.352108471	-0.352108471	-0.352108471
23457	8.079811343	6.6741289	5.268446414	4.375164248	0.676269646	2.525716947
23461	3.519327359	3.9195059	3.119148782	1.688802714	1.939746065	1.437859363
23463	7.416889498	10.319741	4.514038468	6.345275458	4.213456757	8.477094158
23464	-0.352108471	-0.352108	-0.352108471	5.578154865	5.233567424	4.888979982
23468	9.804049994	6.5048286	8.154439274	10.02262407	8.523925919	7.025227772
23469	-0.352108471	-0.352108	-0.352108471	6.48390551	6.32531018	6.166714851
23471	3.127737875	8.0241507	-1.768674914	5.923493182	2.28624554	9.560740824
23474	-0.352108471	-0.352108	-0.352108471	5.99704135	7.300476197	8.603911043
23475	6.234938986	9.4952417	2.974636318	1.454895716	2.100935855	0.808855576
23476	3.127737875	2.908022	3.347453733	5.429934077	6.676169033	7.922403988
23478	5.471966574	6.1215288	6.771090964	6.804723141	6.058864621	7.550581661
23479	-0.352108471	-0.352108	-0.352108471	5.155162759	8.808614795	6.981888777

23480	6.449513142	7.8259417	7.137727412	3.475855724	9.361152706	6.418504215
23481	6.257046343	7.1122979	5.401794829	6.682822443	8.181804947	5.183839938
23483	7.451958417	3.9702313	5.711094862	0.228373553	0.808855576	-0.352108471
23491	8.925671182	9.4156787	9.170674948	8.092333076	9.213783115	6.970883038
23492	-0.352108471	-0.352108	-0.352108471	7.532004806	6.12990004	4.727795275
23495	8.521120071	6.3968684	4.272616694	-0.352108471	-0.352108471	-0.352108471
23499	-0.352108471	-0.352108	-0.352108471	8.202448598	5.169157826	2.135867053
23503	5.960709576	6.8678067	6.414258136	-0.352108471	-0.352108471	-0.352108471
23505	6.396868383	8.4438433	4.349893457	7.081809584	7.275234691	7.178522138
23507	5.394392653	4.5275136	6.261271753	-1.118602831	1.427477396	3.973557622
23509	-0.352108471	-0.352108	-0.352108471	5.998406448	7.18160554	4.815207355
23511	5.836219855	6.5344823	7.232744718	-0.352108471	-0.352108471	-0.352108471
23513	-0.352108471	-0.352108	-0.352108471	6.53389913	7.835921017	5.231877244
23516	-0.352108471	-0.352108	-0.352108471	5.604153369	6.57113554	6.087644454
23518	8.263231782	6.0923221	7.17777694	5.578889148	6.165870606	6.752852064
23521	8.877460729	7.4243668	10.33055465	6.8277183	11.80598196	9.316850133
23522	5.852937462	5.873346	5.832528937	-0.352108471	-0.352108471	-0.352108471
23523	6.317834233	6.3439771	6.291691359	-0.352108471	-0.352108471	-0.352108471
23524	4.681125638	6.7557939	5.718459767	7.985216799	9.316830007	6.653603591
23526	8.047456911	9.0493743	7.045539486	3.632640725	2.123223749	5.142057702
23527	2.86736051	3.2452353	3.6231101	5.921336385	5.942660345	5.963984306
23528	5.089412202	4.4848553	5.693969148	-0.352108471	-0.352108471	-0.352108471
23530	-0.352108471	-0.352108	-0.352108471	5.233567424	5.254689875	5.212444972
23534	-0.352108471	-0.352108	-0.352108471	7.082757193	9.127040761	5.038473626
23536	9.258776798	6.0204943	7.639635545	2.405817835	5.371275165	8.336732495
23541	-0.352108471	-0.352108	-0.352108471	6.056053999	2.90283259	9.209275408
23543	9.134211828	6.8109783	7.972595042	6.89373565	7.586612656	7.240174153
23544	6.095889766	6.1995956	6.303301498	4.696153595	4.245214285	5.147092904
23545	7.676370056	8.0996359	8.522901683	7.526430661	7.320969016	7.115507372
23549	-0.352108471	-0.352108	-0.352108471	4.297639083	4.482692559	4.112585606
23550	-0.352108471	-0.352108	-0.352108471	6.476985109	3.990149738	5.233567424
23553	-0.352108471	-0.352108	-0.352108471	1.37037826	3.971134403	2.670756332
23556	8.510775538	1.8575274	5.184151491	-0.352108471	-0.352108471	-0.352108471
23557	6.339373704	7.1697433	6.754558526	0.874413692	2.100935855	-0.352108471
23560	-0.352108471	-0.352108	-0.352108471	6.636273252	4.139387601	9.133158902
23563	5.119945056	4.2207629	6.019127243	3.006078473	5.670215652	4.338147062
23566	-0.352108471	-0.352108	-0.352108471	3.171900247	2.234223122	2.703061684
23567	5.059679236	4.3280654	3.596451515	1.218975116	1.997577452	0.440372779
23568	7.027375773	8.5376449	5.517106672	5.923493182	7.370494729	4.476491636
23576	7.923242441	4.2583742	6.090808341	5.776693931	11.20946373	8.493078831
23580	-0.352108471	-0.352108	-0.352108471	5.341718312	5.574791816	5.458255064
23581	-0.352108471	-0.352108	-0.352108471	2.966558054	6.888800375	4.927679215
23582	5.157883099	5.7632236	4.552542617	3.719973086	7.400204331	11.08043558
23585	6.861057494	8.4738527	5.248262329	7.436538706	5.760537073	4.08453544
23587	-0.352108471	-0.352108	-0.352108471	5.872307495	6.285525708	5.459089281
23588	-0.352108471	-0.352108	-0.352108471	3.683772944	6.350499927	5.017136436
23589	7.007466298	6.8028046	6.598142994	9.340615408	6.705287512	4.069959615

23591	6.106386272	5.6532998	6.559472732	-0.352108471	-0.352108471	-0.352108471
23593	6.402280332	6.8454315	6.623855918	-0.352108471	-0.352108471	-0.352108471
23594	7.647264576	7.8693257	7.425203426	5.442235949	5.529311366	5.355160532
23596	-0.352108471	-0.352108	-0.352108471	8.105365606	2.633298571	5.369332089
23603	7.070880555	9.5065111	8.288695803	6.141437972	9.950708937	8.046073455
23607	6.801813774	6.1490676	7.454559933	-0.352108471	-0.352108471	-0.352108471
23608	2.351086241	-0.352108	5.054280952	6.472548238	6.791245422	6.153851055
23609	6.713761053	4.6634842	5.688622647	0.440372779	0.440372779	0.440372779
23612	7.568346539	7.5334415	7.603251574	5.430898714	5.458255064	5.403542364
23616	5.354685649	5.3835036	5.325867653	5.775068216	5.804293869	5.745842563
23621	-0.352108471	-0.352108	-0.352108471	4.178899946	4.245214285	4.112585606
23625	-0.352108471	-0.352108	-0.352108471	5.084064216	2.086178387	3.585121301
23627	6.427753893	1.8214372	11.03407061	2.313289664	1.939746065	2.686833264
23629	5.980334028	6.4134257	5.547242364	-0.352108471	-0.352108471	-0.352108471
23633	6.832222404	5.1097254	8.554719431	8.762526086	7.389152202	6.015778319
23636	-0.352108471	-0.352108	-0.352108471	7.159235898	5.171608076	9.146863719
23637	4.945127095	3.0869644	4.016045771	6.417922692	4.320741485	5.369332089
23640	-0.352108471	-0.352108	-0.352108471	4.037734573	4.456886199	4.876037826
23641	6.419382098	6.7203706	7.021359156	7.578384002	6.612481321	7.095432661
23644	-0.352108471	-0.352108	-0.352108471	5.812438496	6.034547868	5.923493182
23645	4.692989119	5.9928175	3.393160694	8.674443538	7.533336972	6.392230407
23646	6.08421453	7.166531	8.248847466	6.61517553	6.505369988	6.724981072
23647	5.317115259	5.1539303	4.990745292	1.427477396	1.305230684	1.549724107
23648	4.457860241	6.3989275	5.428393874	-0.352108471	-0.352108471	-0.352108471
23649	7.743546215	7.826412	7.660680431	6.136283814	6.497336045	5.775231583
23654	7.116455896	5.5876518	6.352053864	7.534433503	7.025721295	6.517009086
23659	5.206978924	5.1522471	5.097515302	4.786565833	6.038556342	5.412561087
23673	-0.352108471	-0.352108	-0.352108471	3.919505936	3.919505936	3.919505936
23677	6.433318728	7.5991588	5.26747866	6.81196605	8.180153401	5.4437787
23678	3.527916452	4.1417014	2.914131516	1.129398324	1.873849414	2.618300505
23704	6.501325856	7.2334683	7.965610799	1.429982115	2.051108653	0.808855576
23705	-0.352108471	-0.352108	-0.352108471	7.120853204	6.532441983	5.944030762
23710	-0.352108471	-0.352108	-0.352108471	4.468401233	4.016115382	4.920687083
23729	4.396788056	5.8984627	5.147625378	3.172412632	4.430456666	5.688500701
23731	5.181747782	5.3835036	4.979991919	0.044132154	-0.352108471	0.440372779
23741	7.462568767	8.7906046	8.126586667	7.245105036	7.81077061	8.376436184
23742	2.591048207	5.7667517	4.178899946	4.933081039	5.335957066	5.738833093
23743	8.277391195	6.4590486	4.640706029	6.105042043	6.847297891	5.362786195
23746	5.573740293	7.2360738	8.898407384	5.9257796	9.362656508	2.488902692
23753	-0.352108471	-0.352108	-0.352108471	7.320831282	5.67944993	4.038068577
23760	3.039085443	4.6226868	6.206288146	6.022050925	5.437108968	6.606992882
23764	7.101764321	7.2616987	7.421632983	4.391914009	7.447005078	5.919459543
23765	7.446721128	6.990778	6.534834801	0.791292145	0.440372779	1.14221151
23768	8.093108738	6.991795	5.890481358	-0.352108471	-0.352108471	-0.352108471
23770	7.761737527	8.5581857	8.159961629	9.37493894	8.027801809	8.701370375
23774	6.172497934	5.8692224	5.565946913	-0.352108471	-0.352108471	-0.352108471
23780	6.508233805	5.7752316	7.241236027	7.002061667	7.044177561	6.959945773

23786	-0.352108471	-0.352108	-0.352108471	3.355836818	2.686833264	2.017829709
23787	6.831289207	6.8453896	6.817188811	7.629719548	7.660627648	7.598811448
23788	-0.352108471	-0.352108	-0.352108471	3.919543357	6.705287512	9.491031667
24137	5.39299759	5.6886226	5.984247704	7.281950102	5.586223009	6.434086555
24138	5.555495184	6.1184215	5.836958364	4.456886199	4.840457771	4.073314628
24139	5.591305588	6.1456332	5.036977942	5.197951829	5.668425445	6.138899061
24144	6.059743139	6.67857	6.369156591	4.105278382	5.498891438	4.80208491
24147	8.354762507	7.1121207	5.869478983	-0.352108471	-0.352108471	-0.352108471
24149	6.515586665	5.8523414	6.183964016	-0.352108471	-0.352108471	-0.352108471
25758	7.137727412	8.0961514	6.179303447	4.808489274	6.022050925	7.235612576
25769	7.491883962	3.5667388	5.529311366	1.542169626	1.646479888	1.437859363
25771	-0.352108471	-0.352108	-0.352108471	6.497182024	6.515031054	6.479332994
25777	6.431242245	4.9613992	7.901085311	5.559035342	4.968668467	5.263851904
25778	6.904464102	8.3825326	9.860601197	7.793995347	6.528630518	5.263265689
25788	5.052267685	6.5778282	3.526707186	1.270297347	2.445220679	0.095374015
25789	-0.352108471	-0.352108	-0.352108471	5.20126514	6.938822793	6.070043966
25792	-0.352108471	-0.352108	-0.352108471	7.592712528	6.004304985	9.181120071
25793	5.939192221	5.9583594	5.920025058	6.165870606	6.174080069	6.157661143
25796	7.148954265	8.9209069	5.377001652	2.211943219	2.232575625	2.191310813
25797	6.396868383	6.4105853	6.383151496	-0.352108471	-0.352108471	-0.352108471
25798	5.895233955	6.7737408	7.652247604	1.76984594	1.807952763	1.731739118
25803	8.739692952	2.5106286	5.625160771	-0.352108471	-0.352108471	-0.352108471
25804	9.020520975	7.0131595	11.02788243	7.913286824	6.710517437	9.116056211
25813	-0.352108471	-0.352108	-0.352108471	7.329088911	7.342696275	7.315481548
25814	7.501341961	6.6741289	5.846915796	7.234851541	8.097755087	6.371947996
25820	5.042557899	4.8304259	5.254689875	-0.352108471	-0.352108471	-0.352108471
25824	6.242048279	6.2558778	6.228218771	7.751300689	7.675016565	7.827584812
25825	7.503256399	6.7823741	8.22413865	2.193505993	10.08152408	6.137515038
25828	6.8086681	7.0305038	7.252339507	5.777198307	8.472954681	7.125076494
25829	8.35539256	7.5180418	6.680690993	7.013360586	8.786278928	5.240442244
25831	-0.352108471	-0.352108	-0.352108471	7.435116686	6.168525672	8.701707699
25832	-0.352108471	-0.352108	-0.352108471	5.006450696	5.88700099	6.767551284
25833	6.735779686	6.9777913	6.493768102	-0.352108471	-0.352108471	-0.352108471
25836	7.15770761	7.7593147	6.556100517	-0.352108471	-0.352108471	-0.352108471
25839	-0.352108471	-0.352108	-0.352108471	5.596916991	6.24931616	4.944517823
25840	4.824032789	4.7079127	4.765972752	5.171890839	4.912488812	5.042189825
25844	7.989609428	5.4377196	6.713664529	7.26625021	7.431136247	7.348693229
25849	-0.352108471	-0.352108	-0.352108471	5.280760807	6.396868383	7.512975958
25850	5.019282955	6.1912665	3.847299435	0.228373553	0.808855576	-0.352108471
25851	8.69435141	7.6966204	6.69888932	6.376166133	5.902534088	5.428902042
25852	-0.352108471	-0.352108	-0.352108471	7.251624328	5.157688188	6.204656258
25853	-0.352108471	-0.352108	-0.352108471	6.222672199	7.248887173	6.735779686
25855	2.226167861	4.9116502	7.597132555	6.847584365	5.924443035	5.001301705
25859	4.793544035	3.5642559	4.178899946	1.344088765	2.460345581	3.576602397
25862	7.134726894	9.024607	8.079666968	7.928821329	6.335196408	4.741571486
25865	4.515242679	5.9245596	5.219901144	4.178899946	4.245214285	4.112585606
25870	3.636824788	1.5378098	5.735839732	3.789390581	6.263986052	5.026688316

25873	8.239261726	8.3045466	8.271904178	8.677025842	8.209022413	9.145029272
25874	4.188589018	3.8435025	4.016045771	8.491743211	7.54696179	6.602180368
25880	-0.352108471	-0.352108	-0.352108471	4.679286418	4.234485981	4.456886199
25885	6.369156591	6.3831515	6.355161685	0.95025487	2.252618212	-0.352108471
25886	2.280956994	-0.352108	4.914022459	2.024343053	2.051108653	1.997577452
25888	5.173604895	6.0767166	5.625160771	-0.352108471	-0.352108471	-0.352108471
25891	-0.352108471	-0.352108	-0.352108471	9.057897116	6.415928975	3.773960833
25893	8.21367466	8.9912187	7.436130605	5.676459262	6.201109411	5.938784336
25894	5.428393874	5.4582551	5.398532684	-0.352108471	-0.352108471	-0.352108471
25895	4.501003242	3.6761388	5.325867653	5.923573886	5.653299811	6.193847962
25896	4.925859754	4.4385056	3.951151354	3.676310814	1.622488375	-0.431334064
25900	-0.352108471	-0.352108	-0.352108471	6.145829416	5.494417177	4.843004937
25902	5.493783215	5.5293114	5.458255064	0.044132154	0.440372779	-0.352108471
25903	-0.352108471	-0.352108	-0.352108471	6.235921161	7.263144806	5.208697515
25906	-0.352108471	-0.352108	-0.352108471	5.637296679	5.653299811	5.621293547
25907	4.855716014	5.6886226	6.52152928	10.54566481	6.923170513	3.300676219
25909	7.510033523	3.5198879	5.514960729	5.980334028	4.939932211	7.020735846
25912	8.359763066	5.3341511	6.846957097	8.400734706	6.671874638	4.943014571
25913	-0.352108471	-0.352108	-0.352108471	8.975872463	5.063102822	7.019487643
25914	-0.352108471	-0.352108	-0.352108471	4.477281902	6.09266681	5.284974356
25915	-0.352108471	-0.352108	-0.352108471	5.688622647	5.715557343	5.661687951
25917	5.078146788	4.8304259	5.325867653	5.637576964	5.529311366	5.745842563
25920	-0.352108471	-0.352108	-0.352108471	5.919459543	6.278508208	5.560410879
25921	6.433318728	9.2419754	3.624662043	7.311199987	6.72156463	6.131929274
25923	5.023812365	5.4994153	4.54820943	4.489274171	7.86441047	6.176842321
25926	-0.352108471	-0.352108	-0.352108471	1.775848523	1.025548163	1.400698343
25927	2.280956994	-0.352108	4.914022459	-0.352108471	-0.352108471	-0.352108471
25929	-0.352108471	-0.352108	-0.352108471	0.793818797	-0.352108471	1.939746065
25930	7.136294354	6.6652281	7.607360639	6.345275458	6.389019324	6.301531591
25932	-0.352108471	-0.352108	-0.352108471	9.445616569	7.232744539	8.339180554
25934	-0.352108471	-0.352108	-0.352108471	6.265475706	5.169157826	4.072839945
25936	6.424082183	11.182436	8.803259273	9.944604641	7.377142908	4.809681175
25937	4.422424947	6.3195146	8.216604336	6.632545947	5.027876734	3.423207522
25938	9.010718858	7.8257767	6.640834571	6.237446502	7.268440449	6.752943475
25939	-0.352108471	-0.352108	-0.352108471	5.153651595	5.805628974	6.457606352
25940	-0.352108471	-0.352108	-0.352108471	5.169157826	5.191222747	5.147092904
25941	-0.352108471	-0.352108	-0.352108471	5.33204173	6.310779945	5.821410838
25942	4.792303669	4.1298287	5.454778632	2.351086241	5.054280952	-0.352108471
25943	-0.352108471	-0.352108	-0.352108471	5.939192221	4.081849691	7.796534752
25948	-0.352108471	-0.352108	-0.352108471	7.115661583	6.359270363	5.602879144
25959	7.697484075	9.1502511	6.244717039	6.810436227	6.720696571	6.900175883
25960	5.017136436	4.5054147	5.528858208	7.517670946	7.292630803	7.742711089
25963	2.523212107	-0.352108	5.398532684	-0.352108471	-0.352108471	-0.352108471
25970	-0.352108471	-0.352108	-0.352108471	5.437466772	7.240174153	9.042881535
25973	6.088359503	7.3786306	4.798088381	6.188322428	6.047709107	6.328935748
25977	-0.352108471	-0.352108	-0.352108471	6.12990004	6.135204448	6.124595632
25979	7.137727412	8.2531302	6.022324617	-0.352108471	-0.352108471	-0.352108471

25983	2.764225616	-0.352108	5.880559702	-0.352108471	-0.352108471	-0.352108471
25987	-0.352108471	-0.352108	-0.352108471	3.585121301	3.615460478	3.554782125
25994	5.829112626	5.6118503	5.394588051	7.869489252	5.060292738	2.251096224
25996	5.834507644	4.3443168	5.089412202	6.434644758	8.477424734	4.391864782
25998	3.80092067	4.4826926	3.119148782	3.820525905	4.954218546	2.686833264
25999	6.801813774	6.8152071	6.788420481	7.364389464	7.386082653	7.342696275
26000	6.151585628	6.1679668	6.135204448	5.017136436	5.054280952	4.979991919
26001	-0.352108471	-0.352108	-0.352108471	6.840114058	6.859202315	6.821025802
26002	-0.352108471	-0.352108	-0.352108471	6.296043865	7.166259349	5.425828382
26003	4.615583513	6.4299117	2.801255293	7.427681558	6.462808162	5.497934765
26005	-0.352108471	-0.352108	-0.352108471	8.084487417	6.056053999	4.027620581
26007	1.392109249	1.5104972	1.273721318	6.914866099	4.756190645	5.835528372
26010	7.527781259	6.9571344	8.098428135	6.29311442	7.059223646	6.676169033
26012	5.90029238	7.5915796	4.209005204	2.211943219	-1.875832852	6.29971929
26015	5.493783215	4.035437	6.952129465	-0.352108471	-0.352108471	-0.352108471
26017	-0.352108471	-0.352108	-0.352108471	6.537383725	7.310428491	5.764338958
26019	4.792303669	6.0135727	3.571034613	5.359116665	6.196807598	7.034498531
26020	8.769684609	8.0756067	7.381528794	7.889819055	6.071471362	9.708166747
26022	7.204336147	7.203004	7.203670057	-0.352108471	-0.352108471	-0.352108471
26024	4.656559241	3.5873447	5.725773777	1.394345699	1.14221151	1.646479888
26027	6.704621053	6.7508628	6.658379305	4.456886199	4.482692559	4.43107984
26030	2.666473317	2.1599023	1.653331202	4.623293667	5.789762726	6.956231785
26032	6.828623008	6.183964	5.539305023	-0.352108471	-0.352108471	-0.352108471
26035	5.625160771	5.6532998	5.597021732	-0.352108471	-0.352108471	-0.352108471
26036	3.635275312	5.743428	7.851580631	6.254539546	5.621763229	6.887315863
26037	5.354685649	5.6410916	5.068279745	-0.352108471	-0.352108471	-0.352108471
26038	4.688098262	5.8818505	5.284974356	3.919505936	3.919505936	3.919505936
26039	6.090808341	6.8981999	5.283416735	-0.352108471	-0.352108471	-0.352108471
26047	4.682964349	4.9799919	4.385936778	1.290035436	1.14221151	1.437859363
26050	4.731111372	4.8304259	4.631796822	-0.352108471	-0.352108471	-0.352108471
26053	2.38621749	5.1245435	-0.352108471	-0.352108471	-0.352108471	-0.352108471
26056	3.43439816	5.7494021	8.064406068	4.248375265	6.033399587	7.818423909
26058	5.169850962	5.9344202	6.698989371	3.808570573	7.638400459	5.723485516
26059	-0.352108471	-0.352108	-0.352108471	0.682499127	1.563603021	2.444706916
26060	5.747047015	5.4147563	5.08246552	5.866748145	5.31909676	4.771445375
26063	-0.352108471	-0.352108	-0.352108471	2.162584513	2.168105043	2.173625573
26064	-0.352108471	-0.352108	-0.352108471	6.960789244	8.530865821	10.1009424
26065	-0.352108471	-0.352108	-0.352108471	3.585121301	3.615460478	3.554782125
26071	6.345267449	6.0203811	5.695494734	8.574165946	5.789762726	3.005359505
26073	-0.352108471	-0.352108	-0.352108471	5.269179996	7.516030975	9.762881953
26080	7.368669107	7.2517577	7.134846261	4.219481858	7.936945973	6.078213916
26082	5.298277456	5.6504741	6.002670694	2.099323895	0.771269572	3.427378218
26086	-0.352108471	-0.352108	-0.352108471	5.494417177	5.494417177	5.494417177
26088	-0.352108471	-0.352108	-0.352108471	3.928167375	4.482667582	3.373667168
26090	-0.352108471	-0.352108	-0.352108471	-0.17577123	1.77365078	3.72307279
26091	-0.352108471	-0.352108	-0.352108471	6.4832423	8.644488633	7.563865467
26092	6.766662835	7.0780531	6.455272547	3.46613827	1.437859363	5.494417177

26093	5.591305588	5.6532998	5.529311366	0.440372779	0.440372779	0.440372779
26097	-0.352108471	-0.352108	-0.352108471	6.165870606	6.174080069	6.157661143
26098	-0.352108471	-0.352108	-0.352108471	7.527328194	6.596757815	5.666187437
26099	7.100899168	7.4790179	7.857136534	6.541009048	3.63594445	9.446073645
26100	-0.352108471	-0.352108	-0.352108471	7.402120487	7.673820483	7.945520479
26108	5.719551143	8.3233271	3.115775196	5.923493182	4.219887391	7.627098973
26115	5.066428095	1.1890477	3.127737875	6.143169347	4.59549483	5.369332089
26118	6.092743499	6.5884653	5.597021732	2.147539136	2.147539136	2.147539136
26121	6.151585628	6.0425019	6.260669336	-0.352108471	-0.352108471	-0.352108471
26128	-0.352108471	-0.352108	-0.352108471	4.80208491	3.866478079	5.737691741
26130	4.665648492	6.0452628	5.355455665	1.713292741	0.301246641	3.12533884
26133	8.926425637	6.7545585	4.582691414	2.705533665	9.765170898	6.235352281
26135	7.016776417	7.4368762	6.596676625	6.871682855	7.081024566	6.976353711
26136	4.772739719	5.1398055	5.506871276	6.177582421	7.306022007	6.741802214
26137	6.241598969	6.3152311	6.167966808	6.372987249	6.410585269	6.33538923
26147	6.658666986	5.1419178	5.90029238	-0.352108471	-0.352108471	-0.352108471
26155	-0.352108471	-0.352108	-0.352108471	8.594325443	6.988122429	5.381919415
26156	9.754301692	3.8559789	6.805140275	7.101332219	7.822957374	6.379707063
26164	7.651123604	7.4068294	7.16253525	6.970121018	7.130171318	7.290221619
26167	-0.352108471	-0.352108	-0.352108471	5.745394463	5.775231583	5.715557343
26168	5.557045014	6.1817288	4.932361242	0.289051906	-0.352108471	0.930212283
26173	-0.352108471	-0.352108	-0.352108471	5.472287825	5.369470884	5.420879354
26188	6.816905042	6.8453896	6.788420481	-0.352108471	-0.352108471	-0.352108471
26190	7.170668878	9.403067	4.938270742	5.837931103	6.355161685	6.872392267
26191	6.732456343	6.6898785	6.647300562	-0.352108471	-0.352108471	-0.352108471
26205	6.463674915	7.171651	5.755698857	-0.352108471	-0.352108471	-0.352108471
26211	-0.352108471	-0.352108	-0.352108471	0.897715333	2.147539136	-0.352108471
26224	5.018523587	4.8786655	4.73880737	-0.352108471	-0.352108471	-0.352108471
26226	-0.352108471	-0.352108	-0.352108471	6.144999634	5.845499835	5.546000037
26227	-0.352108471	-0.352108	-0.352108471	3.733074867	7.675595643	5.704335255
26229	-0.352108471	-0.352108	-0.352108471	9.777151184	6.415584166	3.054017147
26234	-0.352108471	-0.352108	-0.352108471	4.991494611	6.925224158	5.958359385
26235	7.772607719	7.0634138	6.354219893	5.017136436	5.731806615	4.302466256
26238	6.7845177	4.9799919	3.175466138	1.290035436	1.437859363	1.14221151
26253	2.239158726	1.6290946	2.849222896	0.598807818	-0.352108471	1.549724107
26254	-0.352108471	-0.352108	-0.352108471	3.652517449	2.096182139	0.539846828
26258	3.645361578	7.2071663	10.7689711	6.615527989	9.431840942	3.799215036
26259	1.66201518	3.6761388	-0.352108471	1.998530673	6.578873173	4.288701923
26262	-0.352108471	-0.352108	-0.352108471	7.112080183	7.125715465	7.0984449
26263	5.117115863	7.6761805	2.558051256	-0.352108471	-0.352108471	-0.352108471
26267	6.432600891	5.8001998	5.167798684	-0.352108471	-0.352108471	-0.352108471
26268	-0.352108471	-0.352108	-0.352108471	5.708484191	0.529813372	3.119148782
26270	-0.352108471	-0.352108	-0.352108471	5.166304952	3.470468384	1.774631816
26278	6.57228183	3.7257587	5.149020242	-0.352108471	-0.352108471	-0.352108471
26280	2.430446926	3.3526491	2.891547989	1.968661759	5.065821771	-1.128498253
26284	7.042523083	7.9121688	6.172877316	4.259416141	7.852691857	6.056053999
26286	-0.352108471	-0.352108	-0.352108471	5.529311366	4.196819226	6.861803506

26289	-0.352108471	-0.352108	-0.352108471	5.01963484	7.19609887	9.372562901
26301	-0.352108471	-0.352108	-0.352108471	-0.420989195	4.314095009	1.946552907
26353	7.932742872	8.8958046	6.969681191	0.228373553	-0.352108471	0.808855576
26469	6.964525206	5.0322877	5.998406448	6.204656258	4.100987177	8.308325339
26470	-0.352108471	-0.352108	-0.352108471	5.728825219	-0.355158691	2.686833264
26472	6.579253565	7.7836429	8.988032165	9.002007431	8.343176751	8.672592091
26503	7.623306157	4.7642022	6.193754168	2.348496817	1.14221151	3.554782125
26507	6.352053864	6.3831515	6.320956231	0.289051906	-0.352108471	0.930212283
26509	9.14142521	7.4969383	5.852451476	8.143606005	8.378549486	8.613492966
26512	-0.352108471	-0.352108	-0.352108471	4.178899946	4.912487319	3.445312572
26515	5.821144499	4.2689074	5.045025943	5.220888745	1.578759116	3.39982393
26517	5.329443517	3.8056644	2.281885189	6.573706302	7.914406915	9.255107528
26520	-0.352108471	-0.352108	-0.352108471	4.58758557	7.458282885	10.3289802
26525	3.538981516	10.340762	6.939871798	-0.352108471	-0.352108471	-0.352108471
26528	-0.352108471	-0.352108	-0.352108471	6.635453109	6.617035395	6.653870824
26531	7.418556588	7.6404892	7.862421858	2.930976295	1.450757256	4.411195334
26578	-0.352108471	-0.352108	-0.352108471	6.03972704	6.345275458	6.650823875
26585	9.095275794	10.150242	11.20520749	10.30212759	9.771865098	10.03699634
26610	6.291695869	6.7508628	5.832528937	5.744669453	6.174080069	5.315258837
26716	3.194919234	5.7184598	8.242000299	-0.352108471	-0.352108471	-0.352108471
26750	-0.352108471	-0.352108	-0.352108471	3.79520848	5.524906899	4.66005769
26953	7.8875673	6.1601688	7.023868057	-0.352108471	-0.352108471	-0.352108471
26958	5.158989505	4.2269887	4.692989119	5.903760504	7.999764209	3.8077568
26960	-0.352108471	-0.352108	-0.352108471	5.280838845	4.49290523	3.704971615
26985	8.50465951	7.5822179	6.659776387	5.031399336	7.350690663	9.66998199
26986	9.036577582	8.3841322	8.710354877	6.716933979	9.813371762	12.90980955
26993	5.676856174	5.3152588	6.038453511	-0.352108471	-0.352108471	-0.352108471
26994	6.537383725	6.5498529	6.524914536	5.869665514	5.893442277	5.845888751
26996	-0.352108471	-0.352108	-0.352108471	2.076022254	2.100935855	2.051108653
26999	2.803125457	-0.352108	5.958359385	-0.352108471	-0.352108471	-0.352108471
27000	-0.352108471	-0.352108	-0.352108471	6.66853284	5.980334028	5.292135216
27013	-0.352108471	-0.352108	-0.352108471	6.689369697	6.51496239	6.340555083
27018	5.373803093	6.4267817	5.90029238	8.069180721	7.289078056	6.50897539
27020	5.567303509	6.0908083	6.614313172	6.25843907	6.893972732	7.529506393
27030	7.233420289	9.5108706	4.955969934	-0.352108471	-0.352108471	-0.352108471
27031	2.05053464	5.1811997	3.615867194	4.162109162	4.296218215	4.430327269
27032	8.913597772	5.1620002	7.037798976	6.94160582	5.420304984	8.462906656
27042	4.692989119	4.7545518	4.631426448	6.17075544	4.442957573	7.898553308
27043	-0.352108471	-0.352108	-0.352108471	6.903642175	7.034710302	6.772574047
27044	5.052267685	5.1245435	4.979991919	6.573515752	6.751466056	6.92941636
27063	2.153542172	1.9465529	1.739563642	7.631180945	5.704335255	3.777489565
27068	6.30667169	4.8989757	3.491279706	5.854662198	7.633484442	6.74407332
27072	0.646228405	6.9494164	3.797822384	5.915852686	4.249204357	7.582501014
27085	3.688837602	1.7342855	2.711561556	-0.352108471	-0.352108471	-0.352108471
27086	4.16706778	3.5975821	4.736553482	0.872801732	1.305230684	0.440372779
27089	5.482113919	4.9197655	4.357417075	0.044132154	0.440372779	-0.352108471
27090	-0.352108471	-0.352108	-0.352108471	9.632727286	6.713776402	3.794825517

27095	6.939871798	8.6200365	5.259707065	-0.352108471	-0.352108471	-0.352108471
27097	-0.352108471	-0.352108	-0.352108471	-0.72678813	5.150674567	2.211943219
27101	5.749402114	5.4035453	6.095258973	7.053070406	6.170264227	7.935876586
27102	6.273832259	5.6157769	6.931887572	8.14643898	6.022050925	3.89766287
27115	6.203747741	5.7752316	5.346715425	0.228373553	0.808855576	-0.352108471
27122	8.994698813	8.5858873	9.40351033	8.126202188	8.322306903	8.224254545
27125	-0.352108471	-0.352108	-0.352108471	5.789762726	5.804293869	5.775231583
27131	1.383520155	-0.352108	3.119148782	2.880228422	7.944893753	5.412561087
27145	7.378771276	7.1689345	7.588608089	3.314032311	4.688318557	1.939746065
27148	0.281260669	3.8493234	2.065292044	5.575983727	3.531453418	7.620514035
27151	4.741928851	7.3701791	6.056053999	-0.352108471	-0.352108471	-0.352108471
27153	2.596968388	1.3921092	0.18725011	6.534375618	5.704335255	4.874294891
27160	6.137515038	5.2714665	7.003563595	5.314908033	6.20719443	5.761051232
27161	-0.352108471	-0.352108	-0.352108471	0.940233577	2.232575625	-0.352108471
27163	2.907929169	1.6332569	4.182601415	-0.352108471	-0.352108471	-0.352108471
27165	-0.352108471	-0.352108	-0.352108471	5.88700099	6.864218611	4.909783368
27166	6.592882062	6.7206966	6.848511079	5.969862298	11.08864583	8.529254064
27173	-0.352108471	-0.352108	-0.352108471	3.03902233	5.511864271	7.984706212
27183	-0.352108471	-0.352108	-0.352108471	2.124237496	2.147539136	2.100935855
27197	6.103271777	7.3933658	8.683459865	5.326085421	6.906962769	8.487840118
27198	7.628446264	7.7405367	7.852627184	2.458478139	2.960269185	3.462060231
27201	5.490262688	5.3835036	5.597021732	1.223721097	1.305230684	1.14221151
27202	6.640648628	5.5587679	4.476887112	-0.352108471	-0.352108471	-0.352108471
27229	7.077695328	7.7976944	7.437694861	1.76984594	1.807952763	1.731739118
27230	-0.352108471	-0.352108	-0.352108471	6.762354676	7.080913136	6.443796216
27231	-0.352108471	-0.352108	-0.352108471	4.569899033	4.477947052	4.385995071
27233	4.03844829	5.1062182	6.17398819	5.045930461	4.688318557	5.403542364
27236	2.588601447	3.7202533	1.456949631	-0.352108471	-0.352108471	-0.352108471
27238	5.493783215	4.0729942	6.914572265	-0.352108471	-0.352108471	-0.352108471
27241	2.783958294	-0.352108	5.920025058	-0.352108471	-0.352108471	-0.352108471
27243	6.77374078	6.7884205	6.759061079	6.204656258	6.215464554	6.193847962
27245	5.493783215	5.5293114	5.458255064	5.723485516	5.731413688	5.715557343
27246	-0.352108471	-0.352108	-0.352108471	7.490962177	5.405071495	6.448016836
27247	-0.352108471	-0.352108	-0.352108471	2.588801725	7.126278144	4.857539934
27249	-0.352108471	-0.352108	-0.352108471	2.076156865	1.861166652	1.968661759
27252	-0.352108471	-0.352108	-0.352108471	4.66005769	2.647697119	6.67241826
27254	-0.352108471	-0.352108	-0.352108471	5.35767489	5.688622647	6.019570405
27257	6.537828779	6.1515856	5.765342478	5.728340377	7.599372572	6.663856474
27258	-0.352108471	-0.352108	-0.352108471	6.636648361	5.973500237	5.310352113
27287	4.140892218	4.9042312	5.667570149	7.861581775	4.665641434	6.263611604
27292	-0.352108471	-0.352108	-0.352108471	6.056053999	6.073654487	6.038453511
27297	6.132049638	6.0023087	6.261790604	6.013323705	6.497336045	5.529311366
27303	3.755482464	5.6574939	7.559505297	-0.352108471	-0.352108471	-0.352108471
27304	6.427305038	6.9120012	7.396697329	7.124174257	7.486619122	6.761729393
27309	-0.352108471	-0.352108	-0.352108471	8.075580324	6.32531018	4.575040036
27315	3.046628152	4.3296817	1.763574581	7.288837175	6.266367194	5.243897212
27327	6.183964016	5.6139114	6.754016618	8.870202908	2.554323947	5.712263427

27332	4.834101937	4.9140225	4.754181416	-0.352108471	-0.352108471	-0.352108471
27333	7.014830488	6.8158326	7.213828363	7.405008048	7.661556013	7.148460083
27334	5.908529786	7.3595674	8.810605066	7.405907392	3.264792644	5.335350018
27335	-0.352108471	-0.352108	-0.352108471	8.603159288	7.025721295	5.448283302
27336	-0.352108471	-0.352108	-0.352108471	5.17624662	4.456886199	3.737525779
27338	6.123464522	6.573969	7.024473477	8.283357691	11.84096615	4.725749232
27339	6.916902905	6.3259785	5.735054062	8.122270746	8.096623898	8.070977051
27340	4.72290018	6.5274214	5.625160771	-0.352108471	-0.352108471	-0.352108471
27341	8.441583316	5.4677681	6.954675705	4.935567507	2.420058224	3.677812865
27343	-0.352108471	-0.352108	-0.352108471	6.180884509	6.641226906	7.101569303
27345	6.709860607	5.4581656	4.206470524	4.601932212	6.081561369	7.561190526
27346	-0.352108471	-0.352108	-0.352108471	7.649421913	4.435200535	6.042311224
27347	-0.352108471	-0.352108	-0.352108471	5.017136436	5.659255615	4.375017256
27348	-0.352108471	-0.352108	-0.352108471	6.12990004	8.158514235	4.101285845
27350	-0.352108471	-0.352108	-0.352108471	5.032557161	9.327101948	7.179829555
27352	-0.352108471	-0.352108	-0.352108471	7.77741297	5.426310767	6.601861868
27433	5.800199788	6.6856637	4.914735866	-0.352108471	-0.352108471	-0.352108471
27434	2.728078487	4.5853023	3.656690413	-0.352108471	-0.352108471	-0.352108471
27436	1.383520155	0.3166727	2.450367626	6.355161685	3.937328752	8.772994618
27440	-0.352108471	-0.352108	-0.352108471	4.553130743	6.721462614	5.637296679
27443	8.386263177	4.8853777	6.635820429	6.488107881	4.154692756	8.821523006
27445	7.461044856	5.466305	6.463674915	-0.352108471	-0.352108471	-0.352108471
28232	6.985681406	6.8610575	6.736433582	8.386851221	6.621168045	4.85548487
28511	5.711094862	7.4440762	3.978113556	1.769397609	1.437859363	2.100935855
28512	-0.352108471	-0.352108	-0.352108471	6.12990004	5.991950789	6.267849292
28513	2.33062269	9.2697769	5.800199788	-0.352108471	-0.352108471	-0.352108471
28952	-0.352108471	-0.352108	-0.352108471	3.089363405	2.301055037	1.51274667
28955	6.020381091	6.0384535	6.002308672	6.804783999	6.729085562	6.880482436
28956	-0.352108471	-0.352108	-0.352108471	7.936959839	6.804723141	5.672486444
28958	5.804500999	4.0895134	4.947007189	1.036211897	1.14221151	0.930212283
28962	1.949648284	1.390066	1.66985715	5.483227165	6.065730918	4.900723412
28964	7.741402636	4.95698	6.349191313	6.956278745	8.93000611	4.98255138
28971	6.433318728	5.706274	7.160363456	-0.352108471	-0.352108471	-0.352108471
28972	6.237359685	4.4008338	5.31909676	8.717686121	3.530048435	6.123867278
28973	6.396868383	5.7609201	7.03281671	-0.352108471	-0.352108471	-0.352108471
28974	6.37860232	6.7251418	6.55187204	10.67494309	7.311506909	3.94807073
28976	-0.352108471	-0.352108	-0.352108471	4.927679215	4.954218546	4.901139884
28977	1.895892502	2.9815613	4.067230157	-0.352108471	-0.352108471	-0.352108471
28982	5.339283507	7.1507213	3.527845723	2.393884559	2.100935855	2.686833264
28984	-0.352108471	-0.352108	-0.352108471	6.945665103	6.204656258	5.463647413
28985	-0.352108471	-0.352108	-0.352108471	6.825079184	5.983174787	7.66698358
28987	-0.352108471	-0.352108	-0.352108471	4.209585124	4.497788032	4.353686578
28988	6.292446905	5.2562937	5.774370319	6.463674915	6.106300602	6.821049228
28991	5.386363878	6.3968684	7.407372887	5.864389623	5.575983727	5.28757783
28992	1.383520155	-0.352108	3.119148782	5.830669655	5.81215202	5.821410838
29015	-0.352108471	-0.352108	-0.352108471	5.958359385	3.303235231	8.613483538
29028	1.605426148	3.1277379	4.650049603	7.063180684	4.579640992	5.821410838

29035	-0.352108471	-0.352108	-0.352108471	2.902991023	3.119148782	2.686833264
29058	-0.352108471	-0.352108	-0.352108471	5.457623201	7.609959778	9.762296355
29062	1.313555951	4.0160458	6.718535591	4.34283885	5.907753157	7.472667463
29063	6.67661128	5.1446553	5.91063331	5.334050769	5.961166865	6.588282961
29066	-0.352108471	-0.352108	-0.352108471	5.789762726	2.529204905	9.050320547
29070	7.947233071	8.1005797	7.793886488	6.557896833	6.33538923	6.780404436
29071	-0.352108471	-0.352108	-0.352108471	6.137282446	4.921340285	5.529311366
29072	-0.352108471	-0.352108	-0.352108471	4.80208491	3.777692081	5.82647774
29074	5.601327606	5.2299739	5.415650777	6.45373869	6.4770882	6.430389181
29075	-0.109652291	2.3862175	4.882087271	4.057445307	2.097551045	6.017339569
29078	5.391018164	4.2504633	6.531573067	4.52596196	6.306122915	8.086283871
29081	2.803125457	4.5829492	1.023301716	-0.352108471	-0.352108471	-0.352108471
29082	-0.352108471	-0.352108	-0.352108471	0.762376451	-0.352108471	1.876861374
29085	-0.352108471	-0.352108	-0.352108471	6.70825981	6.595493818	6.821025802
29086	8.376699525	4.4483834	6.412541451	8.670516551	7.450257203	6.229997854
29089	6.674128879	8.0685603	5.279697499	4.80208491	5.737295227	3.866874593
29090	-0.352108471	-0.352108	-0.352108471	6.959144107	6.08682898	5.214513852
29093	-0.352108471	-0.352108	-0.352108471	1.937219413	1.997577452	1.876861374
29099	4.522388273	4.6499796	4.777570964	6.362189164	7.561371235	6.9617802
29100	5.019282955	3.9175304	6.121035531	5.328767528	7.135075351	8.941383175
29101	7.764328239	6.4218119	9.106844563	9.151802906	7.763821673	6.37584044
29102	1.66201518	3.6761388	-0.352108471	5.284974356	5.315258837	5.254689875
29103	6.754558526	6.7884205	6.720696571	7.680867551	7.629372342	7.73236276
29105	-0.352108471	-0.352108	-0.352108471	8.854101178	6.259177972	3.664254767
29109	1.946552907	2.8538579	1.039247916	6.905698177	7.839688273	8.77367837
29117	6.73711611	6.9134889	7.089861686	4.001756877	4.927679215	5.853601553
29121	5.688235857	7.2324736	8.776711429	6.830603111	9.265033524	4.396172698
29123	3.627066922	2.8946573	2.162247722	6.289738157	6.686930883	6.48833452
29127	-0.352108471	-0.352108	-0.352108471	9.178499758	5.26977979	7.224139774
29128	-0.352108471	-0.352108	-0.352108471	5.903760504	5.926961306	5.880559702
29763	5.55876787	5.5882244	5.529311366	-0.352108471	-0.352108471	-0.352108471
29766	6.446862061	6.1676355	5.888409016	6.960593515	6.597901644	6.235209772
29767	5.836958364	5.7155573	5.958359385	1.82202867	1.646479888	1.997577452
29774	6.396868383	3.4554413	9.338295475	0.044132154	0.440372779	-0.352108471
29781	-0.352108471	-0.352108	-0.352108471	7.700643532	5.637296679	3.573949826
29789	3.515141236	3.6761388	3.354143641	3.80889006	6.523594479	9.238298897
29796	5.656289537	4.2749393	7.037639786	8.539973503	6.611098778	4.682224053
29797	5.366764601	6.0203811	6.673997582	0.685292531	0.440372779	0.930212283
29799	5.254651642	3.8755155	6.633787817	5.980977506	6.526979592	5.434975419
29803	-0.352108471	-0.352108	-0.352108471	5.511864271	4.864368089	6.159360454
29842	-0.352108471	-0.352108	-0.352108471	2.069671431	5.775068216	9.480465001
29843	-0.352108471	-0.352108	-0.352108471	3.856760004	7.917241976	5.88700099
29844	-0.352108471	-0.352108	-0.352108471	7.004815064	4.998803199	6.001809131
29855	-0.352108471	-0.352108	-0.352108471	6.056844696	5.58597698	5.821410838
29880	-0.352108471	-0.352108	-0.352108471	5.88700099	5.893442277	5.880559702
29881	-0.352108471	-0.352108	-0.352108471	6.417337328	4.456886199	2.496435071
29886	6.98667423	6.3034612	5.620248259	7.583649138	6.038266652	6.810957895

29888	5.657419033	8.7317979	7.194608474	8.652188099	6.963991695	5.275795291
29889	-0.352108471	-0.352108	-0.352108471	5.821410838	1.461677386	10.18114429
29890	4.469217117	5.5587679	6.648318624	6.343016691	7.094349975	7.845683258
29894	5.979826013	5.1841515	4.38847697	-0.352108471	-0.352108471	-0.352108471
29896	-0.352108471	-0.352108	-0.352108471	6.092569559	5.567460487	5.042351414
29901	-0.352108471	-0.352108	-0.352108471	6.455934733	6.33538923	6.576480236
29903	-0.352108471	-0.352108	-0.352108471	5.613451577	5.946831771	5.280071383
29904	6.502554792	5.803801	7.201308571	2.546625611	1.939746065	3.153505157
29911	-0.352108471	-0.352108	-0.352108471	5.494417177	5.494417177	5.494417177
29919	-0.352108471	-0.352108	-0.352108471	4.188928666	6.022050925	7.855173184
29920	7.301234227	4.5771502	5.939192221	-0.352108471	-0.352108471	-0.352108471
29923	6.20709195	7.4357282	6.821410068	-1.517590719	4.467754991	1.475082136
29924	6.833507336	7.3424546	7.851401826	6.804723141	7.294257377	6.315188906
29926	0.683997153	2.1002213	1.392109249	7.487653701	9.971551837	5.003755565
29927	7.366953672	7.389392	7.378172854	6.819832971	7.470685508	8.121538044
29934	-0.352108471	-0.352108	-0.352108471	6.345275458	3.946190247	8.744360668
29946	-0.352108471	-0.352108	-0.352108471	7.326250104	6.087644454	4.849038805
29951	5.640469507	7.6755769	6.658023207	-0.352108471	-0.352108471	-0.352108471
29952	6.208649533	7.0390623	6.623855918	-0.352108471	-0.352108471	-0.352108471
29956	9.36603002	4.044545	6.705287512	5.842848767	6.434644758	7.02644075
29957	5.939192221	5.857316	6.02106846	6.51496239	9.652757569	3.377167212
29959	-0.352108471	-0.352108	-0.352108471	7.699813202	6.12990004	4.559986878
29964	7.144008157	4.9328989	6.038453511	-0.352108471	-0.352108471	-0.352108471
29965	8.786386489	6.7357797	4.685172883	6.415928975	6.429470164	6.402387785
29966	4.325659144	3.5193274	2.712995574	4.178899946	4.744513663	3.613286229
29969	4.152721357	4.0758489	4.229593859	7.776147191	4.977438041	6.376792616
29978	-0.352108471	-0.352108	-0.352108471	4.863235064	6.751466056	8.639697048
29979	-0.352108471	-0.352108	-0.352108471	4.581212806	6.243909369	5.412561087
29982	6.51219561	6.8668803	6.689537938	-0.352108471	-0.352108471	-0.352108471
29984	-0.352108471	-0.352108	-0.352108471	7.455642915	4.318359065	5.88700099
29985	-0.352108471	-0.352108	-0.352108471	5.095423988	5.89269242	4.298155555
29997	-0.352108471	-0.352108	-0.352108471	6.593151207	8.410675965	10.22820072
29998	4.482692559	4.4826926	4.482692559	5.64354012	5.665786693	5.621293547
30000	7.998904298	8.6756935	7.322115106	7.402857594	5.233567424	3.064277253
30001	6.831550127	4.6854967	5.758523395	1.157998715	2.819798514	1.988898614
30008	9.938964031	8.2032738	6.467583629	7.855823578	7.271932595	8.439714562
30010	9.023404481	1.0543387	5.038871611	-0.352108471	-0.352108471	-0.352108471
30011	-0.352108471	-0.352108	-0.352108471	6.735779686	6.750862801	6.720696571
30062	4.346498009	5.9611669	7.575835722	-0.352108471	-0.352108471	-0.352108471
30811	3.682181533	3.5941051	3.770257999	4.195595504	5.124519876	4.66005769
30814	-0.352108471	-0.352108	-0.352108471	5.017136436	7.271027803	2.763245068
30815	-0.352108471	-0.352108	-0.352108471	9.489000106	5.878686198	7.683843152
30816	2.103572783	3.173969	1.03317661	8.077827351	5.439500958	2.801174564
30827	2.711561556	5.7752316	-0.352108471	4.66005769	4.688318557	4.631796822
30833	3.968518528	4.2452143	3.69182277	3.245625856	3.337746555	3.153505157
30835	6.405394225	8.036012	4.774776465	1.848794271	2.051108653	1.646479888
30836	6.295406877	7.1761525	6.735779686	8.108816557	6.32531018	4.541803804

30837	5.701328676	5.873346	5.529311366	6.669136697	6.549852913	6.788420481
30844	-0.352108471	-0.352108	-0.352108471	6.266367194	6.276856601	6.255877787
30845	-0.352108471	-0.352108	-0.352108471	5.011509295	5.529311366	6.047113437
30846	7.419575248	6.9601104	7.879040127	6.895988469	9.051419013	11.20684956
30968	-0.352108471	-0.352108	-0.352108471	8.991808588	5.821410838	2.651013088
50485	-0.352108471	-0.352108	-0.352108471	4.927679215	6.578636598	3.276721832
50486	5.625160771	6.8313888	4.418932781	-0.352108471	-0.352108471	-0.352108471
50488	7.577765451	5.0871679	6.332466669	-0.352108471	-0.352108471	-0.352108471
50506	-0.352108471	-0.352108	-0.352108471	3.760567851	4.557244691	5.353921531
50509	7.466207166	8.6024546	6.329959688	6.44316181	6.6560422	6.868922591
50614	-0.352108471	-0.352108	-0.352108471	1.194816876	3.119148782	5.043480687
50626	7.092979298	4.4058249	5.749402114	-0.352108471	-0.352108471	-0.352108471
50649	5.65953574	5.5587679	5.458000001	-0.352108471	-0.352108471	-0.352108471
50652	6.156815946	6.2904837	6.42415147	3.135445554	4.509501421	5.883557288
50700	-0.352108471	-0.352108	-0.352108471	6.034789061	1.804222811	3.919505936
50717	6.403273394	4.9739719	5.688622647	5.839415865	7.119104888	6.479260377
50807	2.419557138	1.8542919	2.984822416	4.748488389	7.980296749	11.21210511
50808	8.315000385	6.711724	9.918276797	6.649742828	9.61437929	8.132061059
50809	6.341498931	6.4544986	6.228499221	8.676591527	7.069984516	5.463377505
50813	6.920432842	6.9398718	6.959310754	8.135558837	7.855473441	7.575388044
50814	6.012642407	6.5942801	6.303461245	6.287201116	4.665471125	5.47633612
50831	-0.352108471	-0.352108	-0.352108471	2.181832679	2.245600013	2.309367347
50848	6.790732909	6.5710574	7.010408437	4.20408136	6.410585269	1.997577452
50853	0.688532109	2.4815752	4.274618257	-0.352108471	-0.352108471	-0.352108471
50854	5.493783215	5.5293114	5.458255064	6.359270363	6.383151496	6.33538923
50861	5.774370319	6.331224	5.21751664	-0.352108471	-0.352108471	-0.352108471
50862	5.617307162	7.1098214	6.363564301	1.697414861	1.14221151	2.252618212
50865	4.876360176	6.6400194	8.403678609	0.808855576	0.808855576	0.808855576
50937	4.837050227	4.0160458	3.195041315	4.311442591	8.190990035	6.251216313
50940	4.5088668	4.4633304	4.554403201	4.681770414	3.94221199	3.202653565
50944	6.035244926	6.3028839	6.570522784	-0.352108471	-0.352108471	-0.352108471
51002	6.320360815	7.9158694	4.724852206	7.851395897	7.51420419	7.682800044
51008	-0.352108471	-0.352108	-0.352108471	2.434335261	3.585121301	4.735907341
51009	6.096621231	6.8119539	7.527286577	2.92016921	2.686833264	3.153505157
51010	6.463674915	6.479333	6.448016836	-0.352108471	-0.352108471	-0.352108471
51011	3.064630013	7.4319783	5.248304154	2.580284794	3.182789328	2.881537061
51012	-0.352108471	-0.352108	-0.352108471	6.235921161	7.995067821	4.4767745
51014	5.852937462	4.5669565	7.138918437	-0.352108471	-0.352108471	-0.352108471
51015	-0.352108471	-0.352108	-0.352108471	4.086396019	4.456886199	4.82737638
51016	-0.352108471	-0.352108	-0.352108471	6.056053999	7.647051903	4.465056095
51018	6.913488898	6.1934843	7.633493452	-0.352108471	-0.352108471	-0.352108471
51019	-0.352108471	-0.352108	-0.352108471	6.767480029	6.709581906	6.651683784
51020	-0.352108471	-0.352108	-0.352108471	4.637866428	6.290187386	7.942508344
51021	4.3514372	4.4868934	4.622349688	4.56273683	4.121846117	3.680955403
51023	-0.352108471	-0.352108	-0.352108471	5.575983727	6.237615023	4.91435243
51024	1.934017962	4.1788999	6.423781929	3.952076395	5.677239679	7.402402963
51025	-0.352108471	-0.352108	-0.352108471	0.95025487	-0.352108471	2.252618212

51026	6.151585628	7.6177473	4.685423938	-0.352108471	-0.352108471	-0.352108471
51031	6.835211701	4.6635925	5.749402114	-0.352108471	-0.352108471	-0.352108471
51035	4.583433279	1.0513276	8.115538988	8.846908253	7.738888398	6.630868543
51043	6.849096524	7.0978125	7.346528536	-0.352108471	-0.352108471	-0.352108471
51056	-0.352108471	-0.352108	-0.352108471	7.374012451	6.993562721	6.61311299
51059	-0.352108471	-0.352108	-0.352108471	6.235921161	7.095719752	5.37612257
51060	-0.352108471	-0.352108	-0.352108471	5.169157826	5.191222747	5.147092904
51061	6.831289207	7.3368253	6.32575307	7.206478843	7.667689977	7.43708441
51063	5.017136436	7.8536087	2.180664165	0.228373553	0.808855576	-0.352108471
51065	6.396868383	7.3700848	5.423651973	7.843360631	7.518229813	7.680795222
51067	2.783958294	5.9200251	-0.352108471	0.044132154	0.440372779	-0.352108471
51069	-0.352108471	-0.352108	-0.352108471	5.442973377	2.894423748	0.345874119
51070	4.324986295	7.052259	5.688622647	4.860061001	5.958359385	7.056657768
51071	6.000247872	7.6656518	4.334843917	-0.352108471	-0.352108471	-0.352108471
51072	3.392731564	3.1277379	2.862744186	4.244939442	5.017136436	5.78933343
51073	-0.352108471	-0.352108	-0.352108471	6.296043865	6.31523113	6.276856601
51074	5.428393874	7.5663338	3.290453914	-0.352108471	-0.352108471	-0.352108471
51076	5.980334028	8.6149786	3.345689426	-0.352108471	-0.352108471	-0.352108471
51077	6.073315734	6.6142706	7.155225366	7.280806664	6.736151269	7.825462059
51078	-0.352108471	-0.352108	-0.352108471	5.158472126	7.245818252	6.202145189
51079	7.685345331	3.505654	5.595499658	8.564944236	7.886327916	7.207711596
51081	-0.352108471	-0.352108	-0.352108471	4.994366381	5.980334028	6.966301676
51084	-0.352108471	-0.352108	-0.352108471	2.887746055	2.686833264	2.485920472
51088	7.58629982	5.9879683	6.787134073	-0.352108471	-0.352108471	-0.352108471
51092	-0.352108471	-0.352108	-0.352108471	6.056053999	4.802864297	7.309243701
51094	7.024132346	6.6741289	6.324125411	2.204376426	2.359882426	2.048870426
51095	-0.352108471	-0.352108	-0.352108471	0.884207706	8.578476772	4.731342239
51096	-0.352108471	-0.352108	-0.352108471	2.141970815	2.010073694	2.076022254
51097	-0.352108471	-0.352108	-0.352108471	4.413952725	5.007087038	3.820818412
51100	4.475153606	8.1728245	6.323989029	7.897512186	5.273884602	6.585698394
51101	-0.352108471	-0.352108	-0.352108471	6.755400952	5.88700099	5.018601028
51102	-0.352108471	-0.352108	-0.352108471	4.80208491	4.442182752	5.161987068
51103	-0.352108471	-0.352108	-0.352108471	5.406454269	6.284545401	5.845499835
51107	6.640019393	8.1161194	5.163919398	-0.352108471	-0.352108471	-0.352108471
51108	7.194608474	7.2040879	7.18512902	7.429168474	7.443759962	7.414576987
51110	8.522309178	6.7233999	4.924490607	-0.352108471	-0.352108471	-0.352108471
51115	4.624859933	4.617923	4.631796822	4.035615969	6.073654487	1.997577452
51116	5.758523395	6.1478061	5.369240708	5.128763636	5.191222747	5.066304524
51117	-0.352108471	-0.352108	-0.352108471	5.581973955	5.958359385	6.334744814
51118	9.135668534	7.4207556	8.278212087	4.832674249	3.173186306	6.492162193
51119	3.637822277	3.5151412	3.392460195	5.088251181	6.063716272	5.575983727
51121	-0.352108471	-0.352108	-0.352108471	7.504356555	6.682822443	5.861288331
51123	8.880300252	6.2420483	3.603796306	4.890362278	6.087644454	7.284926631
51124	-0.352108471	-0.352108	-0.352108471	6.067944725	4.670719452	5.369332089
51126	-0.352108471	-0.352108	-0.352108471	6.927548141	6.32531018	5.72307222
51128	10.09875553	9.2601109	8.421466364	7.490655993	5.925540989	9.055770997
51131	4.983878974	3.8508272	4.417353062	8.206868105	6.943908403	5.680948702

51132	8.942557916	7.9757892	7.009020494	8.50643431	3.946179484	6.226306897
51133	9.089264964	6.0275287	7.558396831	5.789762726	5.924051489	5.655473963
51135	7.933246481	9.2698371	6.596655885	4.523055044	6.194255311	2.851854777
51138	8.095767591	4.8315822	6.463674915	6.32597821	5.704335255	5.082692299
51142	7.160144567	7.2691536	7.378162675	9.916675497	7.15770761	4.398739723
51143	6.837680934	5.3531522	8.322209653	4.76286975	7.88775061	6.32531018
51148	2.375336938	1.8802386	1.385140197	9.784059907	8.18491552	6.585771133
51149	-0.352108471	-0.352108	-0.352108471	6.000222335	5.775231583	6.225213087
51150	5.775231583	5.7752316	5.775231583	5.980334028	6.002308672	5.958359385
51154	-0.352108471	-0.352108	-0.352108471	8.278098816	4.376584435	6.327341626
51155	5.428393874	7.5324736	3.324314191	6.439080015	7.387897781	6.913488898
51157	7.51917757	6.0182774	9.020077695	8.189559484	7.783514883	7.986537183
51160	5.671162933	8.0736673	6.872415104	4.295558047	4.927679215	5.559800383
51163	6.492539081	2.6021473	4.547343177	0.808855576	0.808855576	0.808855576
51164	-0.352108471	-0.352108	-0.352108471	4.178899946	4.245214285	4.112585606
51166	6.369156591	6.3831515	6.355161685	-0.352108471	-0.352108471	-0.352108471
51168	7.762619187	4.3855143	6.074066758	-0.352108471	-0.352108471	-0.352108471
51172	2.313941724	4.9799919	-0.352108471	-0.352108471	-0.352108471	-0.352108471
51174	7.658222527	9.02866	6.287785086	5.687593236	5.440342203	5.56396772
51177	-0.352108471	-0.352108	-0.352108471	8.797874287	6.625595045	4.453315803
51181	-0.352108471	-0.352108	-0.352108471	5.663737322	5.403166952	5.924307691
51182	6.444762452	5.5056377	4.566512853	5.362907068	5.784960503	6.207013938
51185	-0.352108471	-0.352108	-0.352108471	5.017136436	3.520516721	6.513756151
51186	-0.198099909	8.2301915	4.016045771	5.760537073	4.998688579	6.522385566
51188	-0.352108471	-0.352108	-0.352108471	6.414848889	7.025892365	6.720370627
51196	6.417642846	6.9820481	7.546453331	3.015521513	6.383151496	-0.352108471
51198	-0.352108471	-0.352108	-0.352108471	5.657665837	5.08099834	5.369332089
51199	8.552475495	5.8001998	3.04792408	-0.352108471	-0.352108471	-0.352108471
51200	4.640023572	3.9130675	5.366979609	5.953079284	6.335196408	6.717313532
51202	6.438837895	5.3617469	5.90029238	5.284974356	5.656157877	4.913790835
51203	-0.352108471	-0.352108	-0.352108471	3.030666647	5.789762726	8.548858805
51204	-0.352108471	-0.352108	-0.352108471	2.75880335	2.049256654	1.339709957
51206	2.089114519	5.2752485	3.682181533	2.18071281	6.414739587	4.297726199
51218	-0.352108471	-0.352108	-0.352108471	5.515939194	6.528162656	6.022050925
51222	4.669448073	6.7052875	8.74112695	-0.352108471	-0.352108471	-0.352108471
51226	-0.352108471	-0.352108	-0.352108471	6.004055887	6.895440991	7.786826095
51228	7.944557904	8.2932379	8.641917884	11.11719353	7.863969603	4.610745681
51230	6.465504752	5.5533016	6.009403184	5.038712954	6.230935805	5.63482438
51232	7.923357374	9.5526506	8.738004005	4.571342734	8.032716566	11.4940904
51234	-0.352108471	-0.352108	-0.352108471	8.103562579	4.008545419	6.056053999
51236	6.674128879	6.7163061	6.63195166	5.117338506	6.497336045	7.877333584
51241	-0.352108471	-0.352108	-0.352108471	1.492819042	3.337746555	-0.352108471
51246	-0.352108471	-0.352108	-0.352108471	10.69472539	5.554186394	8.124455894
51250	6.472179624	5.4884884	5.980334028	5.789762726	6.172815652	5.4067098
51253	5.800199788	5.825168	5.775231583	5.821410838	5.825167992	5.817653684
51255	6.815766583	4.0970484	5.456407505	-0.352108471	-0.352108471	-0.352108471
51256	-0.352108471	-0.352108	-0.352108471	4.178899946	4.245214285	4.112585606

51257	-0.352108471	-0.352108	-0.352108471	7.67825419	5.958359385	4.238464579
51258	6.049371728	8.5506032	7.299987458	7.973504604	8.39909175	8.824678896
51260	6.632910846	5.6886226	4.744334448	-0.352108471	-0.352108471	-0.352108471
51264	4.563315445	7.6165612	6.089938313	6.154267535	6.53628338	6.345275458
51268	5.708307224	7.5908015	3.825812906	5.38675695	6.610539552	4.162974349
51271	5.688622647	5.7155573	5.661687951	7.357359337	7.3720224	7.342696275
51272	3.152579374	5.6772397	8.201899984	6.633361894	7.515917946	8.398473998
51278	6.090808341	7.280263	4.901353723	0.624614178	0.808855576	0.440372779
51279	5.520712498	5.0422071	5.999217859	4.586443407	9.039677357	6.813060382
51280	4.615583513	4.5318733	4.699293772	7.174377708	6.349064641	5.523751573
51281	6.212536209	5.8643707	6.560701765	-0.352108471	-0.352108471	-0.352108471
51282	3.464585346	5.6239374	4.544261391	9.026053403	6.025999089	7.526026246
51283	5.625160771	5.6532998	5.597021732	5.903760504	5.926961306	5.880559702
51284	7.687113844	7.4131685	7.550141174	6.260835661	6.098297717	6.423373606
51285	6.766242362	4.7325619	5.749402114	-0.352108471	-0.352108471	-0.352108471
51287	3.515141236	3.2804088	3.749873645	5.630272544	7.348693229	9.067113913
51289	6.804144622	5.9341686	6.369156591	-0.352108471	-0.352108471	-0.352108471
51290	-0.352108471	-0.352108	-0.352108471	4.610601178	5.821410838	7.032220498
51292	6.264668196	6.7357797	7.206891176	7.469464088	5.821410838	4.173357588
51293	-0.352108471	-0.352108	-0.352108471	8.674916953	6.611364719	7.643140836
51294	-0.352108471	-0.352108	-0.352108471	5.529311366	5.417906901	5.64071583
51295	-0.352108471	-0.352108	-0.352108471	5.663737322	5.665786693	5.661687951
51299	-0.352108471	-0.352108	-0.352108471	3.119148782	3.322933285	2.915364278
51306	6.439963118	7.3777551	8.315547149	-0.352108471	-0.352108471	-0.352108471
51311	7.044627223	6.9389629	6.991795048	-0.352108471	-0.352108471	-0.352108471
51312	-0.352108471	-0.352108	-0.352108471	4.716755351	7.243912706	5.980334028
51315	-0.352108471	-0.352108	-0.352108471	5.095423988	5.124543452	5.066304524
51316	6.906139814	6.2588342	5.611528576	7.763592	5.784960503	3.806329006
51317	-0.352108471	-0.352108	-0.352108471	6.755008001	7.994443751	7.374725876
51320	-0.352108471	-0.352108	-0.352108471	10.2205435	6.497182024	2.77382055
51321	6.910067869	7.2227716	7.06641971	-0.352108471	-0.352108471	-0.352108471
51322	4.780916268	5.5873867	5.184151491	6.056053999	4.342506257	7.769601741
51324	-0.352108471	-0.352108	-0.352108471	5.067718723	8.04566108	6.556689902
51329	3.752410772	6.1416036	4.947007189	-0.352108471	-0.352108471	-0.352108471
51330	8.271904178	8.3775056	8.166302741	8.719522258	9.701745762	7.737298753
51332	6.294373215	4.806726	5.550549618	-0.352108471	-0.352108471	-0.352108471
51333	5.093708821	5.4283939	5.763078928	-0.352108471	-0.352108471	-0.352108471
51335	-0.352108471	-0.352108	-0.352108471	6.913448327	6.946414218	6.880482436
51341	-0.352108471	-0.352108	-0.352108471	5.729130667	5.668425445	5.607720223
51343	5.651764351	5.3835036	5.920025058	-0.352108471	-0.352108471	-0.352108471
51351	6.055135433	5.4131985	6.697072349	6.248032091	4.983985071	3.719938051
51352	-0.352108471	-0.352108	-0.352108471	6.763708171	6.39125838	6.577483275
51362	6.363762655	5.355158	7.372367313	6.032141459	6.793008544	5.271274375
51366	-0.352108471	-0.352108	-0.352108471	2.099323895	2.147539136	2.051108653
51368	-0.352108471	-0.352108	-0.352108471	4.444556497	7.472162272	5.958359385
51371	-0.352108471	-0.352108	-0.352108471	7.038566468	5.605777824	8.471355112
51372	2.682175909	5.9489752	4.315575532	7.636511252	6.566821108	7.10166618

51374	6.310712697	5.6251608	4.939608845	6.26758739	7.575129529	8.882671669
51377	1.90830372	1.9397461	1.876861374	-0.352108471	-0.352108471	-0.352108471
51379	6.192528489	4.6938295	7.69122747	6.029379157	8.159314756	7.094346957
51380	6.216409359	5.4958944	6.936924363	1.825329981	1.549724107	2.100935855
51382	1.783698732	-0.666961	4.234358003	4.206547363	3.119148782	2.0317502
51386	7.396926017	8.6555085	6.138343513	8.726299413	8.089528252	7.45275709
51388	4.464976357	3.1463523	3.805664353	6.08682898	8.890444016	3.283213944
51389	1.93748006	3.2986358	2.618057952	8.287323812	5.446142271	6.866733042
51390	5.852937462	7.0991064	4.606768555	-0.352108471	-0.352108471	-0.352108471
51393	2.741060894	0.0431576	1.392109249	2.547414545	4.622828057	3.585121301
51398	5.749402114	5.7752316	5.723572646	6.737448313	6.653870824	6.821025802
51399	5.415461631	5.8131109	6.210760241	5.088940933	5.574791066	6.060641199
51400	3.334031409	5.2849744	7.235917304	8.537212273	8.907361041	9.277509808
51406	4.016045771	4.6684689	3.363622691	7.286324647	8.013063162	6.559586132
51409	7.137727412	7.1464808	7.128974067	-0.352108471	-0.352108471	-0.352108471
51421	-0.352108471	-0.352108	-0.352108471	7.331272742	7.35252903	7.310016454
51422	-0.352108471	-0.352108	-0.352108471	6.963991695	3.977248458	9.950734931
51426	-0.352108471	-0.352108	-0.352108471	7.29572006	5.470026894	6.382873477
51428	-0.352108471	-0.352108	-0.352108471	5.169157826	8.366069546	1.972246105
51429	-0.352108471	-0.352108	-0.352108471	6.104429468	3.973347116	8.235511819
51430	4.89956404	5.062887	5.226209947	-0.352108471	-0.352108471	-0.352108471
51433	-0.352108471	-0.352108	-0.352108471	5.489764936	7.012304751	6.251034844
51434	-0.352108471	-0.352108	-0.352108471	3.119148782	3.119148782	3.119148782
51435	-0.352108471	-0.352108	-0.352108471	5.290303242	6.022050925	6.753798609
51440	-0.352108471	-0.352108	-0.352108471	4.456886199	4.482692559	4.43107984
51441	-0.352108471	-0.352108	-0.352108471	7.511984635	5.47633612	3.440687606
51444	6.124947903	6.502263	6.879578154	-0.352108471	-0.352108471	-0.352108471
51449	4.979991919	4.2205765	5.739407335	5.577939858	7.024738766	8.471537674
51455	-0.352108471	-0.352108	-0.352108471	5.923493182	5.926961306	5.920025058
51458	-0.352108471	-0.352108	-0.352108471	1.560761548	2.191310813	0.930212283
51465	4.431473802	6.3968684	8.362262963	-0.352108471	-0.352108471	-0.352108471
51473	3.458535395	4.7552401	6.051944835	0.440372779	0.440372779	0.440372779
51474	7.08718283	7.0988366	7.075529067	7.196717237	7.429168474	7.661619712
51478	5.670784109	5.3858055	5.528294812	-0.352108471	-0.352108471	-0.352108471
51479	-0.352108471	-0.352108	-0.352108471	8.097572983	5.16611952	6.631846251
51493	2.84317252	6.0384535	-0.352108471	5.789762726	2.577576166	9.001949286
51495	4.319641631	6.0203811	7.721120552	5.939192221	3.861464771	8.016919672
51497	7.489189205	5.9002924	4.311395555	8.63438663	6.693937107	4.753487584
51499	-0.352108471	-0.352108	-0.352108471	7.204588172	6.903642175	6.602696177
51501	-0.352108471	-0.352108	-0.352108471	5.338185733	5.775231583	4.901139884
51504	8.335225499	8.8394758	7.830975233	6.883638803	8.975801101	7.929719952
51506	7.496451613	6.9675112	6.438570877	6.630621079	7.174857204	6.086384954
51507	8.044082269	8.1175462	8.191010166	7.384775166	6.911873916	7.857676415
51510	-0.352108471	-0.352108	-0.352108471	6.859217306	4.47248144	9.245953171
51512	-0.352108471	-0.352108	-0.352108471	4.477947052	4.843308497	4.112585606
51514	-0.352108471	-0.352108	-0.352108471	5.441168403	5.704335255	5.967502107
51522	-0.352108471	-0.352108	-0.352108471	5.227920761	6.396868383	7.565816004

51523	-0.352108471	-0.352108	-0.352108471	8.482967602	5.454747476	6.968857539
51526	-0.352108471	-0.352108	-0.352108471	5.263851904	2.739473082	7.788230727
51527	-0.352108471	-0.352108	-0.352108471	4.927679215	4.954218546	4.901139884
51528	-0.352108471	-0.352108	-0.352108471	5.880139475	6.123478787	6.001809131
51529	4.46201997	5.8242888	7.1865576	6.854064725	6.299485234	5.744905742
51534	6.183964016	5.4207502	6.947177815	7.900808028	7.209478834	6.51814964
51535	-0.352108471	-0.352108	-0.352108471	2.405260623	4.559699198	6.714137774
51538	-0.352108471	-0.352108	-0.352108471	4.220283075	4.137516816	4.178899946
51547	4.724115311	6.2570463	7.789977374	-0.352108471	-0.352108471	-0.352108471
51552	6.681051616	5.9391922	5.197332827	7.177494894	5.473125466	6.32531018
51559	6.299246806	7.4168895	8.53453219	7.57577737	6.637066482	5.698355594
51564	5.89921589	7.7633625	6.831289207	6.310616139	7.408390712	6.859503426
51566	6.124708376	6.183964	6.243219655	5.845499835	5.873345987	5.817653684
51567	9.749922359	8.0204997	6.291077106	4.870983156	6.29836953	5.584676343
51569	6.167582836	6.1999612	6.135204448	6.631846251	6.653870824	6.609821679
51573	8.305972041	3.9370855	6.121528769	4.625564325	5.325716078	6.025867831
51582	7.629370509	7.9496204	8.269870368	-0.352108471	-0.352108471	-0.352108471
51585	-0.352108471	-0.352108	-0.352108471	5.712263427	5.731413688	5.693113166
51586	6.055584097	7.518684	6.787134073	7.222915496	9.466702048	8.344808772
51588	4.340479165	7.0367661	5.688622647	7.233079593	6.631846251	6.030612909
51593	-0.352108471	-0.352108	-0.352108471	5.800199788	2.970807742	8.629591833
51594	2.553073296	2.5977509	2.508395726	6.007585277	5.319889366	5.663737322
51596	7.094863006	5.5120595	6.303461245	7.971540778	8.950754778	8.461147778
51599	-0.352108471	-0.352108	-0.352108471	5.704335255	7.633216076	3.775454434
51601	6.121528769	6.1414231	6.101634422	0.044132154	0.440372779	-0.352108471
51602	7.212932128	7.0167764	6.820620706	8.791943322	6.750169956	7.771056639
51605	-0.352108471	-0.352108	-0.352108471	5.859617369	5.421329723	6.297905015
51608	-0.352108471	-0.352108	-0.352108471	5.388370172	5.421579811	5.355160532
51614	4.434314669	4.4826926	4.385936778	6.77374078	6.788420481	6.759061079
51619	6.103083059	4.6061791	5.35463107	1.437859363	1.437859363	1.437859363
51626	-0.352108471	-0.352108	-0.352108471	3.767483207	3.615460478	3.919505936
51629	6.714613543	6.2899125	6.502263028	7.129313784	6.963975544	7.294652023
51635	-0.352108471	-0.352108	-0.352108471	5.860389288	6.631846251	7.403303215
51637	6.2064191	8.831936	7.51917757	7.565391169	6.724665022	5.883938874
51639	-0.352108471	-0.352108	-0.352108471	5.284974356	5.315258837	5.254689875
51643	2.989802693	7.7989826	5.394392653	3.792182502	6.714299082	5.253240792
51645	-0.352108471	-0.352108	-0.352108471	5.631432108	7.506886071	6.56915909
51646	3.003655834	6.3823224	4.692989119	8.113000316	7.224139774	6.335279232
51647	4.176758563	4.923857	4.550307802	6.880832995	6.876579765	6.872326536
51651	6.705287512	7.5902131	5.820361896	-0.352108471	-0.352108471	-0.352108471
51657	-0.352108471	-0.352108	-0.352108471	4.171955224	7.25257163	5.712263427
51659	5.437440043	5.7043353	5.971230467	-0.352108471	-0.352108471	-0.352108471
51660	5.089412202	3.9992132	6.179611183	-0.352108471	-0.352108471	-0.352108471
51661	1.783698732	3.9195059	-0.352108471	5.859617369	4.058585122	7.660649616
51663	7.789324397	6.4136577	7.101491044	0.808855576	0.808855576	0.808855576
51665	1.383520155	-0.352108	3.119148782	5.886425029	5.926961306	5.845888751
51666	2.486879591	-0.352108	5.325867653	-0.352108471	-0.352108471	-0.352108471

51667	-0.352108471	-0.352108	-0.352108471	5.88700099	7.338600665	4.435401315
51669	-0.352108471	-0.352108	-0.352108471	6.886610445	6.068977539	7.704243351
51678	4.449748927	4.9799919	3.919505936	6.115657815	6.073654487	6.157661143
51684	2.479453761	8.5081127	5.493783215	4.27890488	8.052836332	6.165870606
51693	6.801813774	6.2660669	7.337560692	5.538722293	6.149337851	6.759953409
51699	-0.352108471	-0.352108	-0.352108471	6.022050925	6.544096941	5.500004909
51702	5.641106521	3.6711761	7.611036934	-0.352108471	-0.352108471	-0.352108471
51714	-0.352108471	-0.352108	-0.352108471	9.535112267	6.296043865	3.056975463
51715	-0.352108471	-0.352108	-0.352108471	2.169424975	2.191310813	2.147539136
51720	-0.352108471	-0.352108	-0.352108471	2.039485684	-0.352108471	4.43107984
51725	5.185586761	5.3050787	5.066094857	1.644875323	1.14221151	2.147539136
51726	7.016776417	7.0229788	7.010573985	5.575983727	5.588224375	5.563743079
51727	6.056053999	6.0736545	6.038453511	1.427477396	1.549724107	1.305230684
51728	-0.352108471	-0.352108	-0.352108471	3.119148782	3.119148782	3.119148782
51729	6.640019393	6.6538708	6.626167961	-0.352108471	-0.352108471	-0.352108471
51747	1.66985715	-0.352108	3.69182277	9.992481445	3.335774932	6.664128189
51752	5.649551807	6.7052875	7.761023217	-0.352108471	-0.352108471	-0.352108471
51755	4.513304033	4.1125856	4.914022459	4.49290523	3.919505936	5.066304524
51759	5.123761479	7.1135257	3.133997254	7.851250602	6.668865983	5.486481363
51763	2.16755883	2.6715554	2.419557138	6.187786833	5.987502076	6.087644454
51768	10.33983763	6.6580618	8.498949707	7.319460441	8.73961392	8.029537181
51773	-0.352108471	-0.352108	-0.352108471	6.434644758	5.486363508	7.382926008
51776	5.51959128	5.3371965	5.428393874	1.90830372	1.939746065	1.876861374
51780	5.591305588	2.7774933	8.40511789	0.044132154	-0.352108471	0.440372779
51804	8.324270279	8.1370816	8.511458909	6.859246472	6.873503784	6.844989161
51805	-0.352108471	-0.352108	-0.352108471	1.999631788	2.191310813	1.807952763
51808	7.639063299	5.8022074	9.475919233	11.5068171	4.17269669	7.839756893
51809	-0.352108471	-0.352108	-0.352108471	3.585121301	3.918291565	3.251951037
51816	6.873791272	6.8289675	6.918615007	4.362857055	6.709020222	9.055183389
53339	-0.352108471	-0.352108	-0.352108471	1.162206581	1.666761161	2.171315742
53340	-0.352108471	-0.352108	-0.352108471	5.704335255	5.715557343	5.693113166
53342	-0.352108471	-0.352108	-0.352108471	4.953627254	6.12990004	7.306172826
53344	-0.352108471	-0.352108	-0.352108471	2.019412013	1.873693801	1.946552907
53349	-0.352108471	-0.352108	-0.352108471	5.458255064	5.16916214	5.747347988
53353	-0.352108471	-0.352108	-0.352108471	4.207628449	4.80208491	5.396541371
53354	6.847646477	6.0388597	5.2300729	7.994564784	6.392769641	4.790974498
53358	-0.352108471	-0.352108	-0.352108471	4.374271398	5.923493182	7.472714967
53615	5.939192221	5.9583594	5.920025058	7.695010698	7.617484963	7.772536433
53635	5.609202143	6.9286216	4.289782714	7.348035589	5.871050458	6.609543024
53820	4.979991919	5.7202413	4.239742497	1.218975116	0.440372779	1.997577452
53827	8.468173444	7.7920339	7.115894405	8.435596219	4.999603189	11.87158925
53834	5.439805394	5.3972911	5.354776768	6.479260377	5.567182839	7.391337914
53838	7.468260011	7.4939952	7.442524773	6.77374078	6.788420481	6.759061079
53841	-0.352108471	-0.352108	-0.352108471	3.385478649	7.265953507	5.325716078
53844	6.317834233	6.3439771	6.291691359	-0.352108471	-0.352108471	-0.352108471
53917	-0.352108471	-0.352108	-0.352108471	2.879347611	6.434644758	9.989941906
53938	-0.352108471	-0.352108	-0.352108471	5.972179095	5.835528372	5.698877648

53942	-0.352108471	-0.352108	-0.352108471	4.925871155	6.56915909	8.212447024
53944	5.168965007	5.9856879	6.802410864	5.914903203	6.527456998	5.302349409
53981	6.352932756	7.4744473	5.231418187	7.933674328	7.952342287	7.91500637
54014	3.215228584	1.6126137	2.413921152	-0.352108471	-0.352108471	-0.352108471
54020	5.354685649	5.2271959	5.48217543	-0.352108471	-0.352108471	-0.352108471
54053	-0.352108471	-0.352108	-0.352108471	7.677697856	3.200808737	5.439253296
54058	6.502263028	5.496584	7.507942042	-0.352108471	-0.352108471	-0.352108471
54059	-0.352108471	-0.352108	-0.352108471	5.017136436	5.146407029	4.887865842
54067	1.392109249	1.3760133	1.408205209	1.167362396	2.686833264	-0.352108471
54107	5.863834306	3.4496011	4.656717726	6.383151496	4.746856317	8.019446675
54108	2.12618815	4.0160458	5.905903392	6.525683261	5.800199788	5.074716314
54149	-0.352108471	-0.352108	-0.352108471	3.282392851	5.295431597	7.308470343
54165	2.860773008	2.0210917	3.700454289	-0.352108471	-0.352108471	-0.352108471
54187	-0.352108471	-0.352108	-0.352108471	5.939192221	5.705243906	6.173140537
54205	6.738859034	7.7131438	8.687428534	6.561123102	6.488990562	6.633255642
54206	4.426865312	4.7323714	4.579618372	0.689815323	-0.352108471	1.731739118
54212	5.749402114	5.7752316	5.723572646	-0.352108471	-0.352108471	-0.352108471
54361	-0.352108471	-0.352108	-0.352108471	5.095423988	5.124543452	5.066304524
54407	-0.352108471	-0.352108	-0.352108471	7.604813664	8.619042109	6.59058522
54431	-0.352108471	-0.352108	-0.352108471	7.328554585	7.304001074	7.353108096
54434	7.651189583	7.7313381	7.57104105	7.599970365	7.507270991	7.692669739
54442	5.876783138	7.0505667	6.463674915	7.442587199	4.628131829	6.035359514
54443	6.212536209	5.8465643	6.578508075	-0.499482893	4.316090332	1.90830372
54453	6.913488898	6.9268019	6.900175883	6.556689902	6.588465267	6.524914536
54455	4.089894112	7.5105055	5.800199788	-0.352108471	-0.352108471	-0.352108471
54458	1.501017585	-0.352108	3.354143641	7.780488171	7.797723886	7.763252455
54460	7.104901534	7.7168051	6.492998006	3.341452738	5.546459669	1.136445807
54461	-0.352108471	-0.352108	-0.352108471	8.910662411	2.580126515	5.745394463
54462	5.101071723	5.5246135	5.948155185	7.386463326	6.68034224	5.974221154
54464	1.383520155	-0.352108	3.119148782	3.000039162	3.218334491	2.781743832
54471	6.658244114	6.0480939	5.4379437	0.685292531	0.440372779	0.930212283
54472	-0.352108471	-0.352108	-0.352108471	6.804723141	7.215455623	6.393990659
54476	5.573387224	4.9058738	4.238360373	6.684260862	5.876341455	6.280301159
54491	7.016776417	4.5437254	9.489827447	3.447055498	7.020079349	5.233567424
54495	5.839733082	6.4505296	6.145131319	4.977710418	4.485507002	5.469913833
54498	-0.352108471	-0.352108	-0.352108471	0.593305467	2.208091219	1.400698343
54499	6.474992002	5.4856761	5.980334028	5.627079346	9.973041899	7.800060623
54502	5.242161786	5.7246682	6.207174638	4.752288804	7.985488211	6.368888508
54505	5.519508656	5.7585234	5.997538135	5.651086567	3.953083253	4.80208491
54507	-0.352108471	-0.352108	-0.352108471	6.72865797	6.549852913	6.907463027
54509	5.681727979	5.5882244	5.775231583	3.41553996	4.954218546	1.876861374
54510	-0.352108471	-0.352108	-0.352108471	6.397175529	4.74991884	8.044432218
54517	6.396868383	6.4105853	6.383151496	-0.352108471	-0.352108471	-0.352108471
54518	-0.352108471	-0.352108	-0.352108471	5.980334028	5.17497708	6.785690977
54522	4.498824301	6.6884613	5.593642802	3.733586426	2.462906036	3.098246231
54529	-0.352108471	-0.352108	-0.352108471	3.48963174	8.982210582	6.235921161
54531	1.328643428	1.3921092	1.45557507	4.681934516	7.649806696	6.165870606

54534	4.695524885	5.8744238	5.284974356	-0.352108471	-0.352108471	-0.352108471
54535	-0.352108471	-0.352108	-0.352108471	4.456886199	5.089866335	3.823906064
54539	7.533336972	6.3402385	8.726435421	5.169157826	6.398706384	3.939609267
54540	-0.352108471	-0.352108	-0.352108471	6.022050925	5.789830958	6.254270892
54543	5.749402114	5.7752316	5.723572646	7.535634638	7.555244212	7.516025064
54545	2.946665475	6.2730965	9.599527545	1.493791735	1.437859363	1.549724107
54552	5.559971507	6.6638531	4.456089877	5.663737322	5.710679745	5.616794898
54553	5.980334028	6.0023087	5.958359385	-0.352108471	-0.352108471	-0.352108471
54554	4.259723664	3.6761388	4.843308497	1.678838435	1.549724107	1.807952763
54555	7.924897654	6.0876445	4.250391255	4.466776131	1.771521433	3.119148782
54581	4.036988101	3.0016666	3.519327359	6.164526205	7.098778816	5.230273595
54583	-0.352108471	-0.352108	-0.352108471	3.228447668	4.201291885	2.255603452
54587	6.477478606	9.0072731	7.742375865	8.489516008	9.032811303	8.761163655
54596	8.399602453	8.2516288	8.547576081	7.610066037	7.578336491	7.641795584
54602	-0.352108471	-0.352108	-0.352108471	6.806422367	4.668813097	8.944031638
54619	-0.352108471	-0.352108	-0.352108471	6.087644454	4.202289312	7.972999597
54622	6.24650753	7.932453	4.560562031	-0.352108471	-0.352108471	-0.352108471
54623	-0.352108471	-0.352108	-0.352108471	6.79867074	8.53521005	5.06213143
54626	6.073062111	5.8445235	6.301600732	4.591429197	4.04957035	3.507711504
54627	4.246107069	5.3546856	6.463264229	-0.352108471	-0.352108471	-0.352108471
54629	5.647557376	6.8564204	6.251988912	6.874544191	6.209684281	6.542114236
54662	5.800199788	4.2225901	7.377809459	8.481477505	7.19609887	5.910720235
54663	-0.352108471	-0.352108	-0.352108471	5.823032532	5.859617369	5.896202206
54674	-0.352108471	-0.352108	-0.352108471	5.663737322	6.827700861	4.499773783
54675	-0.352108471	-0.352108	-0.352108471	7.675322316	5.368419324	3.061516331
54676	2.771630448	3.2452353	3.718840162	3.466485722	4.178899946	4.891314169
54680	-0.352108471	-0.352108	-0.352108471	6.12522053	5.614110498	5.869665514
54700	7.286324647	7.2952014	7.277447884	-0.352108471	-0.352108471	-0.352108471
54708	5.800214613	7.3477234	6.573968999	7.1273692	3.861465154	5.494417177
54715	-0.352108471	-0.352108	-0.352108471	5.16766094	5.402287772	5.284974356
54716	3.972745827	4.6049907	5.23723563	5.670931515	4.612241674	6.729621355
54729	4.366177319	6.068805	5.217491139	-0.352108471	-0.352108471	-0.352108471
54732	5.529311366	5.5293114	5.529311366	7.492234695	7.414576987	7.569892404
54737	5.84855283	5.9531359	6.057719069	1.937219413	2.042663021	1.831775805
54739	7.750076311	11.579072	3.921080731	5.944844995	6.370559697	5.519130292
54741	5.665360006	5.8942521	6.123144253	4.96203345	10.07589668	7.518965064
54749	-0.352108471	-0.352108	-0.352108471	5.88700099	5.893442277	5.880559702
54751	9.180742539	9.2133332	9.245923919	8.015966417	7.148091733	8.883841101
54752	-0.352108471	-0.352108	-0.352108471	6.25450531	5.050655725	3.84680614
54753	4.099150854	6.4755755	5.287363196	-0.352108471	-0.352108471	-0.352108471
54758	0.17320027	5.0629156	2.618057952	-0.352108471	-0.352108471	-0.352108471
54764	-0.352108471	-0.352108	-0.352108471	0.793818797	-0.352108471	1.939746065
54765	5.493783215	5.8282527	5.159313761	5.017136436	4.484799241	5.54947363
54768	3.6661225	2.8431725	2.02022254	-0.352108471	-0.352108471	-0.352108471
54776	6.831289207	6.8453896	6.817188811	6.104429468	6.135204448	6.073654487
54784	5.959506607	6.0384535	5.880559702	0.044132154	0.440372779	-0.352108471
54788	5.978718296	6.1075388	6.236359307	6.305009645	7.840024054	7.07251685

54793	6.414258136	4.47463	8.353886312	5.344765918	6.915034162	6.12990004
54795	5.175308152	5.3517823	5.528256413	6.616314976	6.266367194	5.916419412
54798	-0.352108471	-0.352108	-0.352108471	4.852072611	1.639179101	3.245625856
54802	-0.352108471	-0.352108	-0.352108471	7.024005522	7.054600764	7.085196007
54805	6.396868383	4.8531383	7.940598432	-0.352108471	-0.352108471	-0.352108471
54810	6.861057494	6.4891004	7.233014576	-0.352108471	-0.352108471	-0.352108471
54811	4.478932971	4.3878341	4.433383511	2.600631006	-0.277213399	5.478475412
54812	-0.352108471	-0.352108	-0.352108471	5.186168792	1.984073811	3.585121301
54813	6.129093229	5.859383	6.398803434	4.259012879	8.98142958	6.62022123
54815	-0.352108471	-0.352108	-0.352108471	4.574700105	9.729374277	7.152037191
54816	5.90029238	3.03183	8.768754781	-0.352108471	-0.352108471	-0.352108471
54821	-0.352108471	-0.352108	-0.352108471	0.940233577	2.232575625	-0.352108471
54822	7.350009179	8.5607412	9.771473312	9.608477625	5.227293693	7.417885659
54829	4.551931141	0.3397199	2.44582553	-0.352108471	-0.352108471	-0.352108471
54831	-0.352108471	-0.352108	-0.352108471	6.833207703	5.298417208	8.367998198
54834	-0.352108471	-0.352108	-0.352108471	1.631676003	3.615460478	-0.352108471
54840	6.502263028	6.1735584	6.830967621	-0.352108471	-0.352108471	-0.352108471
54841	7.536751785	8.6361134	6.437390133	6.664211791	6.393291482	6.528751636
54842	6.737551942	5.7465446	6.242048279	-0.352108471	-0.352108471	-0.352108471
54843	7.127169143	6.502263	5.877356913	6.415928975	6.581904074	6.249953876
54847	3.419976621	1.2079068	2.313941724	-0.352108471	-0.352108471	-0.352108471
54849	8.875558499	4.2378213	6.556689902	5.918509458	4.651439255	5.284974356
54851	8.428245087	5.8529375	3.277629836	-0.352108471	-0.352108471	-0.352108471
54856	1.646363336	2.4458255	3.245287724	7.315505558	5.052422474	6.183964016
54858	-0.352108471	-0.352108	-0.352108471	2.38621749	5.124543452	-0.352108471
54861	6.607377459	6.6215439	6.593211042	-0.352108471	-0.352108471	-0.352108471
54863	6.702890375	4.7908469	8.614933804	1.971716184	4.036683664	6.101651144
54865	6.658023207	5.4545566	7.861489807	3.953053961	6.38526169	5.169157826
54867	5.35376166	1.6765208	3.515141236	8.001358392	2.737305785	5.369332089
54868	3.805664353	1.0788104	6.532518306	5.637296679	2.963455425	8.311137932
54870	2.351086241	2.2047626	2.497409858	7.27562412	6.149337851	5.023051581
54875	-0.352108471	-0.352108	-0.352108471	0.82273449	-0.352108471	1.997577452
54880	2.313941724	0.768052	3.859831404	-0.352108471	-0.352108471	-0.352108471
54884	-0.352108471	-0.352108	-0.352108471	5.377212601	4.813635374	5.095423988
54885	5.68288063	5.5081187	5.595499658	8.577879408	3.501423418	6.039651413
54888	-0.352108471	-0.352108	-0.352108471	6.217177009	6.588465267	5.845888751
54890	-0.352108471	-0.352108	-0.352108471	2.121850199	3.630525242	5.139200285
54892	-0.352108471	-0.352108	-0.352108471	7.333599114	6.32531018	5.317021247
54896	4.663000528	4.4826926	4.843308497	1.549724107	1.549724107	1.549724107
54897	-0.352108471	-0.352108	-0.352108471	6.447138931	5.23117587	4.01521281
54899	4.475811998	7.9492604	6.212536209	4.807156471	5.789762726	6.772368981
54900	5.038089054	5.8811041	4.195074022	3.908180645	2.252618212	5.563743079
54901	5.023812365	4.3859368	5.661687951	5.440881004	4.843308497	6.038453511
54902	7.409130764	6.4706128	6.939871798	-0.352108471	-0.352108471	-0.352108471
54904	4.3676597	7.6731025	6.020381091	6.587502004	5.233567424	3.879632843
54906	8.069596053	8.0888602	8.050331905	7.036396027	7.044177561	7.028614492
54908	-0.352108471	-0.352108	-0.352108471	6.948900943	5.163207055	6.056053999

54913	-0.352108471	-0.352108	-0.352108471	4.53664933	1.701648233	3.119148782
54915	4.550307802	6.1219461	2.978669528	0.95025487	-0.352108471	2.252618212
54916	-0.352108471	-0.352108	-0.352108471	3.329263459	6.861584516	5.095423988
54918	9.682169607	6.6400194	3.597869178	4.168950146	7.875151704	6.022050925
54919	2.810374321	6.6523102	4.731342239	2.014151132	3.637093921	2.825622527
54920	-0.352108471	-0.352108	-0.352108471	5.529311366	5.292977085	5.765645647
54921	-0.352108471	-0.352108	-0.352108471	6.415928975	9.622979133	3.208878817
54922	-0.352108471	-0.352108	-0.352108471	4.178899946	4.245214285	4.112585606
54923	3.636824788	3.9195059	3.354143641	6.359270363	6.383151496	6.33538923
54925	2.854049416	3.2452353	3.636421193	3.038680847	6.429470164	-0.352108471
54926	-0.352108471	-0.352108	-0.352108471	3.807425866	2.743882657	4.870969075
54928	5.760328836	8.5781316	7.169230199	4.486893444	3.553932763	5.419854124
54930	1.779634147	8.5248601	5.152247113	-0.352108471	-0.352108471	-0.352108471
54933	8.052703623	5.1837761	6.618239874	4.363415057	5.345666926	6.327918794
54934	-0.352108471	-0.352108	-0.352108471	1.400698343	-0.352108471	3.153505157
54936	5.775231583	4.7515777	6.798885497	5.862190408	6.022050925	6.181911442
54939	7.679586811	9.0748086	6.284365007	6.385876888	7.577900666	6.981888777
54940	-0.352108471	-0.352108	-0.352108471	5.693578671	6.802225342	7.910872012
54942	5.463922025	4.3542761	6.573567905	-0.352108471	-0.352108471	-0.352108471
54944	8.289832316	7.5854585	6.881084692	6.962911095	5.128297619	8.797524571
54946	-0.352108471	-0.352108	-0.352108471	6.663856474	6.689878453	6.637834496
54947	3.470136814	5.9414478	8.412758832	2.043642601	1.269708024	2.817577178
54948	-0.352108471	-0.352108	-0.352108471	6.810787265	4.341180188	5.575983727
54949	-0.352108471	-0.352108	-0.352108471	2.527034931	4.245214285	0.808855576
54958	-0.352108471	-0.352108	-0.352108471	3.245625856	4.470626423	2.020625289
54961	5.747301951	3.3521548	4.549728355	6.223903139	3.362112229	4.793007684
54964	6.229986339	5.2849744	4.339962373	-0.352108471	-0.352108471	-0.352108471
54965	7.051819572	6.5150311	7.588608089	6.740979681	6.532588736	6.949370627
54968	-0.352108471	-0.352108	-0.352108471	4.927679215	2.784573532	7.070784897
54969	7.042523083	8.5912568	5.493789338	-0.352108471	-0.352108471	-0.352108471
54972	6.801813774	5.5938471	8.009780493	-0.352108471	-0.352108471	-0.352108471
54973	5.354685649	5.1283305	5.581040843	1.878185472	5.017136436	8.156087399
54976	5.354685649	5.3835036	5.325867653	6.903642175	6.926801914	6.880482436
54977	-0.352108471	-0.352108	-0.352108471	5.154546509	5.997420944	5.575983727
54978	6.334102349	6.0605084	6.197305359	5.620234771	7.853481844	10.08672892
54979	-0.352108471	-0.352108	-0.352108471	0.874413692	-0.352108471	2.100935855
54980	5.931810751	5.825168	6.038453511	-0.352108471	-0.352108471	-0.352108471
54982	3.127737875	3.136327	3.119148782	5.958359385	5.958359385	5.958359385
54985	-0.352108471	-0.352108	-0.352108471	3.536505546	3.919505936	3.153505157
54986	-0.352108471	-0.352108	-0.352108471	3.753284825	2.191310813	5.315258837
54987	-0.352108471	-0.352108	-0.352108471	0.82273449	-0.352108471	1.997577452
54989	5.743427971	5.825168	5.661687951	0.624614178	0.808855576	0.440372779
54994	7.6453538	6.4396924	7.042523083	-0.352108471	-0.352108471	-0.352108471
54996	5.506320168	5.054281	5.958359385	-0.352108471	-0.352108471	-0.352108471
54998	6.553666035	6.2570463	5.960426651	9.336167023	8.669171663	8.002176304
55002	-0.352108471	-0.352108	-0.352108471	7.967719037	6.675314719	5.3829104
55004	4.178899946	4.8615173	3.496282571	7.361848237	5.776469942	6.56915909

55007	7.072293719	6.0316538	8.112933652	6.602657749	5.287360142	7.917955356
55009	5.768807923	6.379717	6.990626032	1.598101998	-0.952181543	4.148385539
55010	5.18789546	7.3597691	6.273832259	5.956840309	6.165870606	6.374900903
55011	-0.352108471	-0.352108	-0.352108471	6.34641237	6.204656258	6.062900146
55012	6.104798308	8.7782849	3.431311755	-0.352108471	-0.352108471	-0.352108471
55013	-0.352108471	-0.352108	-0.352108471	2.451290702	5.254689875	-0.352108471
55014	6.735779686	7.572746	5.898813403	5.412561087	4.520449918	6.304672257
55017	4.897279614	6.571532	8.245784341	5.800199788	9.661200602	1.939198973
55018	1.115419563	1.5010176	1.886615606	5.240354047	4.691916222	4.143478396
55023	7.675582535	5.3991849	6.537383725	4.921245645	5.101786835	5.282328025
55027	1.392109249	2.6841069	0.100111599	1.432957346	1.825329981	2.217702616
55030	-0.352108471	-0.352108	-0.352108471	1.968661759	1.997577452	1.939746065
55031	-0.352108471	-0.352108	-0.352108471	6.735779686	9.61109233	3.860467042
55032	-0.352108471	-0.352108	-0.352108471	5.788761324	5.821410838	5.854060352
55033	6.051283555	8.0021844	7.026733991	6.654609994	6.629085838	6.680134151
55035	5.622434355	3.182219	8.062649718	6.204656258	7.501860847	4.907451669
55037	-0.352108471	-0.352108	-0.352108471	7.311506909	7.187667549	7.435346269
55041	9.218299488	7.5665544	5.914809282	4.927973588	6.050658323	7.173343058
55048	-0.352108471	-0.352108	-0.352108471	6.918733623	5.950555894	6.434644758
55049	6.688515381	5.451953	7.925077713	5.723485516	5.731413688	5.715557343
55052	-0.352108471	-0.352108	-0.352108471	6.964225117	7.159614859	7.061919988
55054	-0.352108471	-0.352108	-0.352108471	5.164205092	5.095423988	5.026642884
55055	5.664172181	6.0736545	5.254689875	5.760537073	5.775231583	5.745842563
55056	-0.352108471	-0.352108	-0.352108471	3.120807694	2.686833264	3.554782125
55062	-0.352108471	-0.352108	-0.352108471	8.14878294	6.514592953	4.880402967
55064	6.142514077	6.6400194	7.137524708	-0.352108471	-0.352108471	-0.352108471
55065	-0.352108471	-0.352108	-0.352108471	6.98391079	6.208432776	5.432954762
55069	-0.352108471	-0.352108	-0.352108471	6.332483041	6.834405266	6.583444154
55071	6.414258136	6.4453648	6.383151496	6.631357585	6.674249904	6.588465267
55073	4.968876799	4.5968917	5.340861945	-0.352108471	-0.352108471	-0.352108471
55074	-0.352108471	-0.352108	-0.352108471	5.760918599	4.927679215	4.094439831
55075	7.041132241	5.4477908	6.24446152	9.375770134	7.449953079	8.412861606
55081	-0.352108471	-0.352108	-0.352108471	7.241986056	6.149337851	5.056689645
55083	-0.352108471	-0.352108	-0.352108471	6.48960905	6.671874638	6.854140227
55093	-0.352108471	-0.352108	-0.352108471	4.456886199	5.254237572	3.659534827
55095	-0.352108471	-0.352108	-0.352108471	5.994586978	8.46722126	7.230904119
55101	-0.352108471	-0.352108	-0.352108471	5.077715323	6.033399587	6.989083851
55105	7.532706586	6.4862653	5.439823932	-0.352108471	-0.352108471	-0.352108471
55107	-0.352108471	-0.352108	-0.352108471	2.301055037	1.421157603	3.180952471
55108	5.034059334	5.7494021	6.464744895	5.365324136	6.396868383	7.428412629
55111	-0.352108471	-0.352108	-0.352108471	3.583632251	2.039485684	0.495339118
55114	-0.352108471	-0.352108	-0.352108471	7.106011286	6.379921394	7.832101178
55116	2.685821551	2.6547897	2.623757929	4.79472922	6.219993395	3.369465046
55119	6.073654487	6.0736545	6.073654487	6.3629082	6.410585269	6.31523113
55122	6.906333693	7.0919708	6.720696571	1.76984594	1.807952763	1.731739118
55124	6.209189517	5.825168	6.593211042	2.4171862	2.686833264	2.147539136
55125	7.906269112	8.7359201	8.321094611	-0.352108471	-0.352108471	-0.352108471

55128	5.886580909	8.0205592	3.752602645	1.591046029	1.876861374	1.305230684
55133	5.924636176	7.4544397	6.689537938	-0.352108471	-0.352108471	-0.352108471
55138	2.932305749	2.5530733	2.173840844	-0.352108471	-0.352108471	-0.352108471
55139	-0.352108471	-0.352108	-0.352108471	5.859617369	5.348078894	6.371155844
55142	7.218147054	6.8086736	7.627620525	5.201358809	5.54408868	4.858628938
55144	5.364396521	7.8972681	10.43013958	7.551136183	9.263834232	5.838438134
55149	7.706634876	7.7938865	7.619383264	6.872480799	7.135139919	6.609821679
55152	-0.352108471	-0.352108	-0.352108471	4.843807885	6.072702243	5.458255064
55154	5.710376656	5.5882244	5.832528937	0.940233577	2.232575625	-0.352108471
55157	-0.352108471	-0.352108	-0.352108471	6.474604683	6.113115027	5.751625371
55160	6.229863096	5.2024139	7.257312332	4.663254863	4.927679215	5.192103567
55161	5.941762076	5.7155573	6.167966808	6.41724734	6.479332994	6.355161685
55163	7.146051896	7.6774193	7.411735596	7.027375773	7.94872399	6.106027556
55165	-0.352108471	-0.352108	-0.352108471	6.127547123	8.108083215	7.117815169
55171	5.868750891	9.6248615	7.746806187	6.627434945	4.051426259	9.203443632
55173	7.191783448	6.3676161	5.543448678	4.508312208	5.65835131	5.083331759
55174	-0.352108471	-0.352108	-0.352108471	4.756084874	7.776649513	6.266367194
55175	7.83762433	7.5648175	7.292010696	3.728048194	2.100935855	5.355160532
55177	6.607377459	8.7800045	4.434750465	5.169157826	5.191222747	5.147092904
55179	3.708250298	7.2058417	5.457046014	0.624614178	0.440372779	0.808855576
55182	-0.352108471	-0.352108	-0.352108471	6.568081043	6.396868383	6.225655722
55184	5.758523395	6.2515562	5.265490541	3.748374607	5.916973891	4.832674249
55186	3.980884861	4.8786655	5.776446095	6.930390828	6.16151075	6.545950789
55187	6.551519093	5.0883424	8.014695776	-0.352108471	-0.352108471	-0.352108471
55188	2.681724436	3.0218939	2.34155499	-0.352108471	-0.352108471	-0.352108471
55191	-0.352108471	-0.352108	-0.352108471	7.208624718	5.017136436	2.825648154
55193	-0.352108471	-0.352108	-0.352108471	5.377275034	7.704743061	6.541009048
55194	7.207838162	8.0519438	6.363732508	5.953806071	8.233856234	10.5139064
55195	5.567994192	5.7359892	5.903984111	6.672907029	5.511952341	7.833861718
55198	1.392109249	3.136327	-0.352108471	0.292732044	3.919505936	7.546279828
55200	-0.352108471	-0.352108	-0.352108471	2.884085473	3.572809864	3.228447668
55201	5.303441833	6.056054	6.808666165	-0.352108471	-0.352108471	-0.352108471
55205	-0.352108471	-0.352108	-0.352108471	6.682822443	7.62312604	5.742518845
55206	7.093830636	7.6972857	6.490375619	3.413859648	6.157655056	8.901450464
55208	8.235365546	8.9027813	7.56794981	6.523594479	8.624216706	4.422972251
55209	6.289711002	5.8001998	5.310688573	-0.352108471	-0.352108471	-0.352108471
55210	-0.352108471	-0.352108	-0.352108471	4.303912246	3.776859569	4.830964923
55214	6.388107363	6.1889144	6.288510867	1.036211897	1.14221151	0.930212283
55215	-0.352108471	-0.352108	-0.352108471	5.597728237	6.215464554	4.979991919
55216	4.430045089	7.6820629	6.056053999	-0.352108471	-0.352108471	-0.352108471
55226	-0.352108471	-0.352108	-0.352108471	5.821410838	4.868646042	6.774175633
55228	2.481575183	4.1227315	0.840418915	3.701877892	0.351893671	7.051862114
55234	8.709851525	7.1256647	5.541477781	7.123669946	9.195829566	5.051510326
55238	6.521408278	6.0411302	7.001686328	4.463476681	5.021739545	5.580002409
55239	6.770915757	8.099141	7.435028399	6.467735705	7.228147803	6.847941754
55240	-0.352108471	-0.352108	-0.352108471	4.30019621	7.998479491	6.149337851
55247	-0.352108471	-0.352108	-0.352108471	6.296043865	4.474671971	8.11741576

55250	-0.352108471	-0.352108	-0.352108471	6.539110051	6.617035395	6.461184708
55252	-0.352108471	-0.352108	-0.352108471	7.495550985	8.416922778	7.956236881
55256	8.091674987	6.6400194	5.188363799	7.4668722	10.88185977	4.051884627
55257	3.895359011	5.3546856	6.814012287	5.729430242	6.170030093	6.610629944
55262	8.627323979	1.9426247	5.284974356	5.152996505	7.439091226	6.296043865
55266	6.133248067	4.69484	3.256431892	5.636342902	6.470939859	6.053641381
55269	-0.352108471	-0.352108	-0.352108471	0.421012343	9.422986951	4.921999647
55272	-0.352108471	-0.352108	-0.352108471	5.637296679	4.345997259	6.928596098
55276	-0.352108471	-0.352108	-0.352108471	5.017136436	3.957881955	6.076390916
55278	3.245235305	3.428504	3.061966623	2.133979419	3.604915675	0.663043163
55280	4.551347431	4.7746536	4.663000528	0.727922146	1.807952763	-0.352108471
55285	5.188023226	7.712057	6.450040136	8.035534783	6.747964147	5.460393511
55288	6.958027251	6.2521643	6.605095787	0.044132154	0.440372779	-0.352108471
55290	6.056053999	3.8711218	8.240986195	-0.352108471	-0.352108471	-0.352108471
55293	7.109007851	6.4333187	5.757629605	-0.352108471	-0.352108471	-0.352108471
55294	6.705287512	7.5343854	5.876189592	5.723485516	4.76461212	6.682358911
55297	4.878665478	4.876467	4.880863991	-0.352108471	-0.352108471	-0.352108471
55298	5.287363196	6.3712083	4.203518064	5.113559352	6.91845452	6.016006936
55299	-0.352108471	-0.352108	-0.352108471	5.20089139	3.40855663	6.99322615
55300	1.068424843	1.3835202	1.698615467	5.980334028	6.416147401	5.544520655
55301	5.878648753	6.3241383	6.769627837	6.000983357	6.288401646	6.575819935
55308	6.735779686	7.4694812	6.002078175	2.795182383	3.337746555	2.252618212
55311	-0.352108471	-0.352108	-0.352108471	5.717169102	6.270222109	6.823275116
55312	-0.352108471	-0.352108	-0.352108471	2.497489344	3.740808219	3.119148782
55313	5.06884253	5.3835036	4.754181416	7.189945282	7.233409808	7.146480757
55317	2.980965692	4.0160458	5.051125849	7.415637154	7.010596604	7.820677703
55323	-0.352108471	-0.352108	-0.352108471	5.575983727	6.552901374	4.599066079
55324	-0.352108471	-0.352108	-0.352108471	6.567242302	6.013323705	5.459405109
55325	-0.352108471	-0.352108	-0.352108471	7.620894845	3.36730463	-0.886285586
55327	4.725685207	3.6761388	5.775231583	1.58479924	1.437859363	1.731739118
55331	8.390305846	7.1054593	9.675152417	10.01762311	4.940842697	7.479232901
55332	6.901979267	6.8795537	6.924404788	8.122214126	9.395606489	6.848821762
55333	5.581892295	4.3193847	6.84439993	7.518618502	6.523809895	5.529001288
55334	-0.352108471	-0.352108	-0.352108471	11.01717006	7.311506909	3.605843761
55337	5.638440722	6.3583722	5.998406448	2.393884559	3.970916082	0.816853037
55338	11.13656857	9.3120279	10.22429824	8.687824621	8.068254759	8.37803969
55341	6.513388045	5.4166411	4.319894132	5.418927122	8.50188854	2.335965704
55343	7.1250675	9.7127339	4.537401137	0.869437987	2.686833264	4.50422854
55352	6.107538801	4.0931198	8.121957827	2.252618212	2.157658228	2.347578196
55353	-0.352108471	-0.352108	-0.352108471	6.803911573	7.435116686	8.066321798
55355	5.827205236	6.2558778	5.398532684	-0.352108471	-0.352108471	-0.352108471
55357	5.352963836	6.1458404	5.749402114	5.168862837	2.360239897	7.977485777
55361	6.674128879	6.6898785	6.658379305	6.997455924	7.016299086	6.978612763
55362	5.428393874	6.7688278	4.08795991	7.036520286	9.203001812	4.870038761
55364	-0.352108471	-0.352108	-0.352108471	5.643917324	6.056053999	6.468190674
55366	-0.352108471	-0.352108	-0.352108471	5.458255064	7.59796796	3.318542168
55367	-0.352108471	-0.352108	-0.352108471	5.745394463	5.775231583	5.715557343

55379	6.717944937	7.9311975	9.144450044	8.35946807	8.927116711	7.791819429
55384	6.878058762	4.4343147	1.990570576	4.588844105	4.456886199	4.324928294
55421	6.119207367	3.9122807	8.32613407	5.27122146	6.667687048	3.874755873
55422	-0.352108471	-0.352108	-0.352108471	5.441867342	3.878248037	4.66005769
55425	6.705287512	3.7447705	9.665804523	0.647185709	-0.352108471	1.646479888
55437	-0.352108471	-0.352108	-0.352108471	7.336289898	6.463674915	5.591059932
55449	4.121813477	9.4494478	6.785630654	6.59549746	6.796870789	6.998244118
55450	5.319117131	5.6087269	5.463922025	8.987376304	7.090076417	5.19277653
55454	-0.352108471	-0.352108	-0.352108471	7.304622832	5.869665514	4.434708197
55471	7.359883697	7.6817556	8.003627415	6.40286535	5.911035771	6.894694928
55501	7.030793546	6.1679668	5.305140071	1.05704313	0.808855576	1.305230684
55504	-0.352108471	-0.352108	-0.352108471	4.178899946	5.603977489	2.753822402
55505	-0.352108471	-0.352108	-0.352108471	5.293500402	8.315945881	6.804723141
55508	6.972176722	7.3915683	6.552785117	2.88676039	4.843308497	0.930212283
55511	-0.352108471	-0.352108	-0.352108471	2.38621749	1.49495207	3.27748291
55512	-0.352108471	-0.352108	-0.352108471	6.82244073	6.969141537	6.675739923
55526	7.886660128	9.6575902	6.115730079	5.262366871	6.265638387	7.268909902
55527	4.148643332	9.7863792	6.967511245	5.923493182	5.926961306	5.920025058
55530	8.539209891	7.4632677	6.387325477	5.343003906	8.770748776	7.056876341
55531	8.150383151	7.9748192	8.3259471	6.834255134	7.109037535	6.559472732
55544	7.680387293	6.9134889	6.146590504	2.492032424	5.336173319	-0.352108471
55558	8.962566876	7.2682187	5.573870562	5.471523648	5.179908508	5.325716078
55567	5.203503428	6.2953008	5.749402114	-0.352108471	-0.352108471	-0.352108471
55568	7.469118306	7.3777551	7.286391961	0.939116071	0.440372779	1.437859363
55571	-0.352108471	-0.352108	-0.352108471	7.234851541	8.657399332	5.81230375
55573	8.563313237	8.8654423	8.26118421	7.508938593	7.653578306	7.798218019
55578	5.463922025	7.3941633	3.533680733	5.883760549	6.10626679	6.328773031
55582	-0.352108471	-0.352108	-0.352108471	1.968661759	1.997577452	1.939746065
55585	7.237556464	3.8799793	5.55876787	7.816131679	5.655427693	6.735779686
55588	7.192541266	6.7431167	6.293692114	2.85499773	3.955942293	1.754053167
55591	2.649419007	7.7855633	5.217491139	-0.352108471	-0.352108471	-0.352108471
55593	-0.352108471	-0.352108	-0.352108471	3.812727913	7.364389464	10.91605101
55596	5.255522095	4.9140225	5.597021732	5.83433693	5.893442277	5.775231583
55599	2.681724436	3.4987012	1.864747714	5.637296679	6.700628631	4.573964727
55602	-0.352108471	-0.352108	-0.352108471	6.235921161	6.246629235	6.225213087
55604	6.991795048	6.9981067	6.985483415	0.044132154	0.440372779	-0.352108471
55607	-0.352108471	-0.352108	-0.352108471	5.596916991	5.67757571	5.516258273
55610	-0.352108471	-0.352108	-0.352108471	2.076022254	2.100935855	2.051108653
55611	5.571436751	5.6886226	5.805808543	11.41520493	3.396021543	7.405613237
55614	4.855015771	5.5799665	5.217491139	2.43016825	-0.352108471	5.212444972
55615	1.321522306	4.9339534	3.127737875	4.249557331	6.137866886	8.026176441
55620	3.968518528	4.2452143	3.69182277	2.301055037	4.954218546	-0.352108471
55621	6.77374078	8.7156105	4.831871014	3.12397275	3.677812865	4.231652981
55623	4.955814037	5.0171364	5.078458834	-0.352108471	-0.352108471	-0.352108471
55624	-0.352108471	-0.352108	-0.352108471	6.225162754	5.11898394	7.331341568
55625	-0.352108471	-0.352108	-0.352108471	5.605179132	5.845499835	6.085820539
55629	7.680370506	6.6561251	5.631879622	4.776750732	3.228447668	1.680144605

55632	4.687425547	8.35252	6.519972795	-0.352108471	-0.352108471	-0.352108471
55634	4.297639083	4.4826926	4.112585606	3.351148927	5.893442277	0.808855576
55635	5.082403109	6.0894758	7.096548526	2.825622527	3.919505936	1.731739118
55638	-0.352108471	-0.352108	-0.352108471	7.968598773	4.769714408	6.369156591
55643	7.012904137	6.502263	5.99162192	5.681727979	4.408596279	6.954859678
55646	-0.352108471	-0.352108	-0.352108471	6.235921161	3.335363006	9.136479316
55651	-0.352108471	-0.352108	-0.352108471	8.618324117	6.709482007	4.800639897
55652	4.128789217	6.6123092	5.370549232	4.811735937	6.847035404	8.882334871
55654	4.876017436	4.3484155	5.403619412	9.557819938	7.454735544	5.35165115
55657	6.273832259	7.3073048	5.240359743	-0.352108471	-0.352108471	-0.352108471
55658	2.950649284	5.1522471	7.353844942	-0.352108471	-0.352108471	-0.352108471
55660	4.400341142	3.6761388	5.124543452	-0.352108471	-0.352108471	-0.352108471
55661	-0.352108471	-0.352108	-0.352108471	7.031454429	7.167295284	7.30313614
55663	-0.352108471	-0.352108	-0.352108471	6.709482007	5.621619332	7.797344682
55664	-0.352108471	-0.352108	-0.352108471	5.591254988	2.247756883	3.919505936
55666	7.816833734	8.0995029	7.534164577	4.440967932	0.945112849	2.693040391
55667	4.414740353	3.7525428	3.090345207	6.782442412	6.951023671	6.866733042
55668	8.755587093	7.9936758	7.231764536	3.87668691	4.103893054	4.331099198
55669	-0.352108471	-0.352108	-0.352108471	5.323028331	3.919505936	2.515983541
55670	7.368246125	7.4315272	7.494808305	6.50377304	5.925043132	6.214408086
55676	6.952955212	6.9980602	7.04316527	6.682896946	5.711376822	4.739856697
55677	7.400498955	5.2849744	3.169449758	6.42520231	5.755827116	7.094577504
55679	7.163235418	7.244076	7.082394844	7.271069954	7.246938498	7.29520141
55689	5.826051513	5.4582551	6.193847962	0.044132154	0.440372779	-0.352108471
55690	4.153967827	4.0348854	4.273050256	7.155889879	6.039525779	8.272253979
55691	6.75358421	8.1239472	5.383221178	6.65068432	6.848539219	6.74961177
55695	-0.352108471	-0.352108	-0.352108471	4.268504401	3.544890796	4.992118006
55697	-0.352108471	-0.352108	-0.352108471	8.381185301	3.139888845	5.760537073
55698	6.538980134	5.4596566	7.618303658	-0.352108471	-0.352108471	-0.352108471
55700	5.422294084	9.0627498	7.242521962	7.787391935	8.621962756	6.952821115
55701	-0.352108471	-0.352108	-0.352108471	5.82786553	5.230757201	5.529311366
55702	4.455411975	4.9841517	5.512891436	0.598807818	-0.352108471	1.549724107
55703	2.451290702	-0.352108	5.254689875	4.178899946	4.164070437	4.193729454
55704	5.354685649	5.3835036	5.325867653	5.723485516	5.731413688	5.715557343
55705	8.49918517	9.1027838	9.706382423	7.113460584	8.840156792	7.976808688
55706	-0.352108471	-0.352108	-0.352108471	4.376579178	5.814268798	5.095423988
55707	-0.352108471	-0.352108	-0.352108471	7.155303995	4.904689811	6.029996903
55715	6.573968999	7.6684273	5.47951066	-0.352108471	-0.352108471	-0.352108471
55720	2.721116247	5.1693921	3.945254192	1.043426334	1.646479888	0.440372779
55721	4.886275954	5.8529145	5.369595226	0.86953393	0.808855576	0.930212283
55727	1.501017585	-0.352108	3.354143641	6.12990004	7.87809651	4.38170357
55728	5.491200093	4.7541814	6.228218771	5.810560167	5.775231583	5.845888751
55729	7.173321394	6.6416301	6.90747574	6.716509042	6.844298182	6.972087322
55731	-0.352108471	-0.352108	-0.352108471	5.958359385	5.958359385	5.958359385
55732	-0.352108471	-0.352108	-0.352108471	7.071901948	4.972199902	6.022050925
55735	-0.352108471	-0.352108	-0.352108471	4.611928496	8.257361021	6.434644758
55737	-0.352108471	-0.352108	-0.352108471	5.849473764	7.376789186	4.322158341

55738	8.094717949	7.6553246	7.875021286	5.030533283	7.963830766	6.497182024
55739	-0.352108471	-0.352108	-0.352108471	5.637628902	6.296043865	6.954458829
55740	5.664172181	4.9770767	6.351267642	8.322490079	5.311761046	6.817125562
55742	4.498150788	5.9253809	7.352611004	9.949029977	8.789483361	7.629936745
55744	-0.352108471	-0.352108	-0.352108471	5.958359385	5.958359385	5.958359385
55748	-0.352108471	-0.352108	-0.352108471	5.300919109	6.341902566	5.821410838
55749	7.053389612	7.701977	6.404802267	6.23199295	6.628098252	7.024203553
55750	-0.352108471	-0.352108	-0.352108471	5.704335255	3.39211717	8.016553339
55752	-0.352108471	-0.352108	-0.352108471	5.937461314	5.169157826	4.400854337
55753	1.563719053	2.6224566	3.681194208	-0.352108471	-0.352108471	-0.352108471
55754	-0.352108471	-0.352108	-0.352108471	7.086413157	7.093739676	7.090076417
55759	4.847192458	6.2125362	7.57787996	-0.352108471	-0.352108471	-0.352108471
55761	-0.352108471	-0.352108	-0.352108471	8.524217559	5.79119766	7.15770761
55762	7.336518169	5.4953398	6.415928975	1.179289842	1.549724107	0.808855576
55763	-0.352108471	-0.352108	-0.352108471	4.178899946	4.935055406	3.422744485
55765	5.579367614	6.0457801	5.112955086	1.761670631	1.646479888	1.876861374
55766	8.383027684	7.5511362	6.719244682	5.517196731	8.235962799	6.876579765
55768	-0.352108471	-0.352108	-0.352108471	4.318823674	5.688622647	7.05842162
55769	5.044800317	5.8830437	5.463922025	-0.352108471	-0.352108471	-0.352108471
55771	7.091092121	8.1899319	9.288771683	7.68864657	9.538902278	5.838390861
55773	-0.352108471	-0.352108	-0.352108471	6.430737678	6.204656258	5.978574838
55775	-0.352108471	-0.352108	-0.352108471	6.056053999	8.686809931	3.425298067
55776	7.3241242	4.9172564	9.730992033	-0.352108471	-0.352108471	-0.352108471
55777	5.800199788	4.973486	6.626913588	-0.352108471	-0.352108471	-0.352108471
55778	3.868535913	4.617923	3.119148782	5.539774305	5.458255064	5.621293547
55780	5.307093674	5.0651838	5.186138762	7.218748868	7.471015121	6.966482616
55783	-0.352108471	-0.352108	-0.352108471	7.440009978	5.06205971	6.251034844
55784	4.372191214	4.1125856	4.631796822	3.606517346	5.336173319	1.876861374
55785	4.682964349	2.6101215	6.7558072	4.158869237	1.217949484	7.099788991
55790	5.545126722	5.6886226	5.832118572	-0.352108471	-0.352108471	-0.352108471
55798	9.361723234	6.0941625	7.727942861	10.24434892	4.075283853	7.159816388
55800	4.278704721	4.2310973	4.183489901	1.227667732	0.808855576	1.646479888
55802	5.724668212	6.4722588	4.977077659	5.370934752	6.062716354	6.754497955
55809	5.926665747	5.4283939	4.930122001	-0.352108471	-0.352108471	-0.352108471
55810	6.824706934	5.9391922	5.053677509	-0.352108471	-0.352108471	-0.352108471
55811	6.868675947	5.2849744	3.701272766	-0.352108471	-0.352108471	-0.352108471
55812	6.242048279	6.2558778	6.228218771	-0.352108471	-0.352108471	-0.352108471
55814	4.692989119	4.7541814	4.631796822	3.585121301	3.615460478	3.554782125
55815	-0.352108471	-0.352108	-0.352108471	5.458255064	5.812477721	5.104032407
55823	-0.352108471	-0.352108	-0.352108471	5.095423988	5.124543452	5.066304524
55824	7.50744655	6.5851844	5.662922201	4.776474833	6.481523945	8.186573057
55827	9.449288932	5.8459441	7.64761654	-0.352108471	-0.352108471	-0.352108471
55830	3.081461292	2.2069086	3.956014006	7.967036148	7.027375773	6.087715399
55831	7.192947048	7.6283292	7.410638113	7.904703535	7.14555803	6.386412524
55832	6.672585169	6.901683	6.787134073	3.787770961	6.001809131	8.215847301
55833	-0.352108471	-0.352108	-0.352108471	5.382237167	8.112305955	6.747271561
55844	5.5042802	6.9120012	8.319722167	6.941881205	6.23567117	5.529461136

55845	7.51917757	6.8146941	8.223661046	6.668335837	6.022050925	5.375766013
55850	1.697502965	2.1956028	1.946552907	4.456886199	3.71637007	5.197402329
55851	7.484018122	7.4939952	7.474040996	6.642245802	6.63062078	6.653870824
55852	-0.352108471	-0.352108	-0.352108471	3.130142041	4.076694717	3.603418379
55854	-0.352108471	-0.352108	-0.352108471	6.415928975	7.824856751	5.007001198
55856	7.505122518	3.2727311	5.388926799	8.79434509	6.270222109	3.746099127
55858	-0.352108471	-0.352108	-0.352108471	7.602877881	7.535634638	7.468391396
55859	-0.352108471	-0.352108	-0.352108471	7.349440613	7.807078794	8.264716976
55860	4.151574402	4.2452143	4.338854169	0.372554099	3.324709145	1.848631622
55862	-0.352108471	-0.352108	-0.352108471	5.789762726	5.804293869	5.775231583
55863	-0.352108471	-0.352108	-0.352108471	2.846613221	7.273972255	5.060292738
55884	4.789383821	5.2849744	5.780564891	9.630906271	6.655369412	8.143137841
55890	-0.352108471	-0.352108	-0.352108471	5.775231583	5.775231583	5.775231583
55892	-0.352108471	-0.352108	-0.352108471	4.334386097	4.435075177	4.535764257
55898	5.879796907	7.0475529	6.463674915	6.872926213	6.760883872	6.816905042
55902	-0.352108471	-0.352108	-0.352108471	6.596757815	7.216190289	5.977325342
55904	6.212536209	7.840436	4.584636391	3.245625856	3.337746555	3.153505157
55905	5.284974356	5.8506084	4.71934028	1.876861374	1.876861374	1.876861374
55907	-0.352108471	-0.352108	-0.352108471	5.789762726	5.214004937	6.365520515
55914	-0.352108471	-0.352108	-0.352108471	10.63840597	6.735779686	2.833153406
55917	-0.352108471	-0.352108	-0.352108471	8.579859391	2.245262784	5.412561087
55920	-0.352108471	-0.352108	-0.352108471	4.183043699	4.847568765	5.512093832
55924	5.235875513	6.2458263	7.255777069	1.086055948	1.731739118	0.440372779
55930	5.039857145	5.7927795	4.286934793	0.228373553	-0.352108471	0.808855576
55954	-0.352108471	-0.352108	-0.352108471	5.529311366	4.527030553	6.531592179
55959	-0.352108471	-0.352108	-0.352108471	4.66005769	4.688318557	4.631796822
55966	5.428393874	5.072397	5.784390724	-0.352108471	-0.352108471	-0.352108471
55967	6.054885801	7.7423759	9.429865929	6.12990004	6.066856893	6.192943187
55968	6.735779686	5.0301626	8.44139674	9.255520967	7.78321146	6.310901954
55969	-0.352108471	-0.352108	-0.352108471	7.094349975	8.635524188	5.553175761
55970	8.081466428	8.1005797	8.062353202	7.937881566	8.062353202	7.813409929
55971	5.961166865	6.7059849	5.216348867	-0.352108471	-0.352108471	-0.352108471
55973	3.092313489	5.3546856	7.617057809	6.27821251	3.756060362	5.017136436
55974	7.658728352	7.5787856	7.738671055	1.05704313	1.305230684	0.808855576
56005	7.707102448	8.7361969	6.678007979	9.577564942	8.662645212	7.747725482
56006	1.383520155	-0.352108	3.119148782	2.351086241	5.054280952	-0.352108471
56034	-0.352108471	-0.352108	-0.352108471	5.263851904	3.902909708	6.6247941
56052	8.670809626	7.3214877	5.972165833	4.817531639	5.260683106	4.374380173
56063	-0.352108471	-0.352108	-0.352108471	3.119148782	3.390817621	2.847479942
56098	6.450302837	6.479333	6.421272681	-0.352108471	-0.352108471	-0.352108471
56102	3.011970594	5.3546856	7.697400704	-0.352108471	-0.352108471	-0.352108471
56106	6.120810648	9.1291045	3.112516782	-0.352108471	-0.352108471	-0.352108471
56111	-0.352108471	-0.352108	-0.352108471	4.725730333	6.682940177	5.704335255
56112	-0.352108471	-0.352108	-0.352108471	4.456886199	5.813835915	3.099936484
56123	6.502263028	5.4667175	7.53780856	-0.352108471	-0.352108471	-0.352108471
56125	4.962326451	6.9808719	5.971599196	0.440372779	0.440372779	0.440372779
56127	8.119361839	5.6519467	6.885654293	6.707475369	5.999070915	5.290666462

56129	6.427239861	5.5334282	5.980334028	-0.352108471	-0.352108471	-0.352108471
56130	7.889542092	6.3324667	4.775391246	-0.352108471	-0.352108471	-0.352108471
56131	5.334952249	5.8278042	6.320656234	-0.352108471	-0.352108471	-0.352108471
56135	5.354685649	5.3835036	5.325867653	-0.352108471	-0.352108471	-0.352108471
56157	5.595499658	5.5293114	5.661687951	-0.352108471	-0.352108471	-0.352108471
56160	6.134800615	6.1679668	6.101634422	-0.352108471	-0.352108471	-0.352108471
56169	4.725969713	5.9985776	5.36227364	0.874413692	-0.352108471	2.100935855
56171	5.57473968	5.5427961	5.55876787	-0.352108471	-0.352108471	-0.352108471
56172	4.843038887	4.3943954	5.291682362	-0.352108471	-0.352108471	-0.352108471
56180	5.532118463	4.691633	5.111875725	7.911318496	7.516025064	7.120731633
56242	4.713922221	4.6627188	4.765125641	1.238701993	1.778924448	2.319146903
56244	5.90029238	6.2114335	5.589151238	-0.352108471	-0.352108471	-0.352108471
56252	3.127737875	3.136327	3.119148782	5.592773207	4.063564683	7.121981732
56253	6.309773462	4.2695759	5.289674704	4.125386496	2.469725738	0.814064979
56255	5.939192221	7.8713344	4.007050077	8.483814337	4.610209508	6.547011923
56257	-0.352108471	-0.352108	-0.352108471	5.874032613	5.534637897	5.704335255
56260	3.789504048	9.2515078	6.520505908	3.122004006	5.627005534	8.132007061
56262	7.035554271	7.4338641	7.832173927	6.709581906	6.720696571	6.698467242
56270	4.701847563	7.6660805	6.183964016	6.689387364	6.920180633	6.804783999
56271	-0.352108471	-0.352108	-0.352108471	5.295431597	2.77959653	7.811266665
56288	7.651923169	5.6251608	3.598398373	-0.352108471	-0.352108471	-0.352108471
56301	2.833283455	1.9391515	2.38621749	0.044132154	-0.352108471	0.440372779
56474	4.291171802	6.078368	5.184769894	-0.352108471	-0.352108471	-0.352108471
56478	-0.352108471	-0.352108	-0.352108471	5.54059285	6.022050925	6.503509
56548	-0.352108471	-0.352108	-0.352108471	5.596916991	5.309048244	5.884785738
56603	5.680046016	5.6251608	5.570275527	-0.352108471	-0.352108471	-0.352108471
56616	-0.352108471	-0.352108	-0.352108471	4.863989554	5.474326098	5.169157826
56648	6.449648682	6.0651966	5.680744586	4.917166473	6.625247983	8.333329493
56651	4.449748927	4.9799919	3.919505936	1.744735086	1.939746065	1.549724107
56652	-0.352108471	-0.352108	-0.352108471	5.095423988	5.124543452	5.066304524
56654	-0.352108471	-0.352108	-0.352108471	7.649062677	7.749669138	7.548456216
56655	-0.352108471	-0.352108	-0.352108471	5.812549441	9.045787508	7.429168474
56660	-0.352108471	-0.352108	-0.352108471	5.723485516	7.493129927	3.953841104
56672	8.67580726	7.29644	7.986123615	7.692191855	6.865288895	7.278740375
56675	7.936989338	8.2060919	7.667886768	3.644427226	9.532381494	6.58840436
56681	3.438778715	8.361806	5.90029238	9.802007181	5.744687039	7.77334711
56731	6.927183976	7.1578622	7.042523083	7.785195405	5.212674344	6.498934874
56776	-0.352108471	-0.352108	-0.352108471	6.359270363	6.107311259	6.611229467
56829	6.542351758	7.207296	5.877407475	-0.867599802	2.049256654	4.966113109
56848	-0.352108471	-0.352108	-0.352108471	3.228447668	3.337746555	3.119148782
56850	5.904400435	6.183964	6.463527597	-0.352108471	-0.352108471	-0.352108471
56852	8.342713403	5.9391922	3.53567104	-0.352108471	-0.352108471	-0.352108471
56882	4.587398901	5.3920614	6.196723817	0.044132154	-0.352108471	0.440372779
56886	8.866030478	7.7566326	6.647234767	6.150470235	5.784035832	5.417601429
56888	7.949620438	7.5748406	8.324400237	6.532441983	7.208182247	5.85670172
56889	-0.352108471	-0.352108	-0.352108471	5.729212323	6.671874638	7.614536953
56890	-0.352108471	-0.352108	-0.352108471	3.245625856	-1.670650613	8.161902325

56891	-0.352108471	-0.352108	-0.352108471	4.053861851	4.954218546	3.153505157
56893	-0.352108471	-0.352108	-0.352108471	6.12990004	8.810137628	3.449662453
56894	6.888413829	7.1985306	6.578297075	7.078372564	6.992640887	7.16410424
56896	4.495239923	5.4895739	6.483907898	2.498005078	1.348914576	3.647095579
56897	-0.352108471	-0.352108	-0.352108471	2.252618212	2.252618212	2.252618212
56900	5.493783215	5.5293114	5.458255064	-0.352108471	-0.352108471	-0.352108471
56902	3.734798172	5.1134061	6.49201397	5.982003862	6.702640675	5.261367049
56903	-0.352108471	-0.352108	-0.352108471	6.204656258	5.471446658	6.937865858
56905	4.26798078	3.3433479	3.805664353	3.55629917	2.301055037	1.045810904
56907	-0.352108471	-0.352108	-0.352108471	7.034741548	6.192514566	7.876968531
56910	6.347551663	6.573969	6.800386336	0.975533543	1.14221151	0.808855576
56911	1.425625587	3.6057696	2.515697587	0.624614178	0.808855576	0.440372779
56915	-0.352108471	-0.352108	-0.352108471	4.596219431	5.973729281	5.284974356
56917	6.414680048	7.0260612	6.720370627	3.516548235	3.379219387	3.24189054
56918	5.268974051	4.617923	5.920025058	1.12335747	1.437859363	0.808855576
56919	-0.352108471	-0.352108	-0.352108471	6.929246588	6.810957895	6.692669202
56926	-0.352108471	-0.352108	-0.352108471	6.840114058	7.833237781	5.846990336
56927	5.089412202	4.2429377	5.935886714	6.054588057	6.537499674	6.296043865
56929	-0.352108471	-0.352108	-0.352108471	5.716336945	5.458255064	5.200173183
56931	6.568734508	4.7358084	5.652271474	-0.352108471	-0.352108471	-0.352108471
56937	-0.352108471	-0.352108	-0.352108471	1.454941542	1.383520155	1.312098768
56938	7.635214613	8.6109224	8.123068514	4.479715328	7.830397474	6.155056401
56940	5.310889134	5.871722	5.591305588	-0.352108471	-0.352108471	-0.352108471
56942	6.77374078	6.7884205	6.759061079	6.345275458	6.355161685	6.33538923
56943	-0.352108471	-0.352108	-0.352108471	5.149133629	7.663488032	6.40631083
56947	-0.352108471	-0.352108	-0.352108471	6.79004344	7.667147196	5.912939685
56949	5.152247113	5.3888412	4.915652991	3.524795961	6.666052015	5.095423988
56951	-0.352108471	-0.352108	-0.352108471	7.192715245	8.153443131	7.673079188
56965	-0.352108471	-0.352108	-0.352108471	9.738071513	7.112080183	4.486088852
56970	-0.352108471	-0.352108	-0.352108471	0.378404991	2.076022254	3.773639517
56975	-0.352108471	-0.352108	-0.352108471	8.1791634	4.491229415	6.335196408
56984	-0.352108471	-0.352108	-0.352108471	5.892395808	6.488107881	7.083819954
56985	7.219551575	4.9620651	6.090808341	-0.352108471	-0.352108471	-0.352108471
56993	-0.352108471	-0.352108	-0.352108471	8.161280744	6.895919043	5.630557342
56995	4.194323704	8.8648142	6.529568951	8.184266684	2.852889775	5.51857823
56996	6.121528769	6.638851	5.604206505	9.105353393	5.923493182	2.741632971
56999	6.661698657	3.5171257	5.089412202	-0.352108471	-0.352108471	-0.352108471
57000	5.262846526	7.0993419	8.935837271	5.849205167	5.964599853	5.73381048
57002	6.159249072	6.3691566	6.57906411	-0.352108471	-0.352108471	-0.352108471
57010	6.636606745	7.7339872	7.185296981	6.362981115	4.970085925	7.755876305
57016	2.016914154	1.3393858	2.694442482	1.963985013	1.876861374	2.051108653
57019	5.284974356	4.6984335	5.871515189	-0.352108471	-0.352108471	-0.352108471
57020	4.016045771	2.1377449	5.894346687	4.178899946	4.50328921	3.854510681
57035	-0.352108471	-0.352108	-0.352108471	10.78499251	2.115613166	6.450302837
57037	-0.352108471	-0.352108	-0.352108471	5.590609441	6.32531018	7.06001092
57045	7.998944175	7.5029595	7.750951825	4.80208491	4.843308497	4.760861323
57060	-0.352108471	-0.352108	-0.352108471	3.6498283	5.44897977	7.248131241

57062	7.751434144	6.3753935	7.063413806	5.760537073	4.941360883	6.579713263
57085	-0.352108471	-0.352108	-0.352108471	5.583895223	6.056053999	6.528212775
57092	-0.352108471	-0.352108	-0.352108471	6.609543024	6.63062078	6.588465267
57095	-0.352108471	-0.352108	-0.352108471	6.296043865	3.846057035	8.746030696
57097	6.788976801	6.6215439	6.956409727	0.598807818	1.549724107	-0.352108471
57103	5.020587291	10.251788	7.63618745	5.985264703	6.344036917	5.626492488
57104	8.69926147	6.9129933	5.12672522	7.262894302	7.727171758	7.49503303
57106	-0.352108471	-0.352108	-0.352108471	4.989921879	3.605356286	4.297639083
57107	7.1144378	7.9945597	6.234315922	8.990114606	4.961525982	6.975820294
57115	4.288635999	6.1463463	5.217491139	-0.352108471	-0.352108471	-0.352108471
57116	-0.352108471	-0.352108	-0.352108471	2.73496259	2.564345668	2.393728747
57118	9.387374614	9.2732507	9.159126777	7.775347331	8.514546855	9.253746379
57124	10.53233641	8.2529058	9.392621122	9.571938236	9.909145	10.24635177
57125	4.684878868	4.2452143	5.124543452	1.804300246	1.876861374	1.731739118
57128	-0.352108471	-0.352108	-0.352108471	5.791067154	5.931810751	6.072554349
57129	-0.352108471	-0.352108	-0.352108471	8.692481662	3.77936066	6.235921161
57130	4.750902969	5.413918	6.076932934	7.041674609	6.868895554	6.696116498
57132	7.153084345	7.9574774	7.555280876	9.042582091	5.008712344	7.025647217
57134	3.195693796	6.1702349	4.682964349	5.536964019	-0.478069171	2.529447424
57136	-0.352108471	-0.352108	-0.352108471	7.160134469	4.759802167	9.560466772
57140	3.150687521	3.1047882	3.127737875	8.571428111	5.850091509	7.21075981
57142	7.049307119	7.5261472	8.002987274	7.661917043	8.056397728	8.450878413
57146	5.874321292	3.7994131	4.83686721	0.440372779	0.440372779	0.440372779
57148	-0.352108471	-0.352108	-0.352108471	5.760537073	6.39629716	5.124776985
57149	-0.352108471	-0.352108	-0.352108471	5.807462027	6.039524337	5.923493182
57153	5.688622647	6.5809064	4.796338895	8.362467561	8.919206195	8.640836878
57157	-0.352108471	-0.352108	-0.352108471	5.195273529	8.105285791	6.65027966
57158	-0.352108471	-0.352108	-0.352108471	6.837787178	7.350906735	7.094346957
57162	-0.352108471	-0.352108	-0.352108471	7.657842008	3.330992345	5.494417177
57169	6.830732744	6.7884205	6.873045007	6.226457505	5.893442277	6.559472732
57175	5.852937462	8.4123056	3.293569273	9.955761578	4.980650259	7.468205918
57176	-0.352108471	-0.352108	-0.352108471	5.490522765	8.323539808	2.657505722
57178	4.596401634	3.898201	5.294602254	7.177031742	7.333182908	7.020880576
57179	4.228795565	5.3537904	6.478785198	5.540133168	6.787320335	8.034507502
57181	-0.352108471	-0.352108	-0.352108471	5.923493182	6.748776523	5.098209841
57182	-0.352108471	-0.352108	-0.352108471	5.412561087	5.178905519	5.646216656
57184	2.844361502	7.7255872	5.284974356	-0.352108471	-0.352108471	-0.352108471
57186	4.792303669	4.8304259	4.754181416	-0.352108471	-0.352108471	-0.352108471
57188	-0.352108471	-0.352108	-0.352108471	4.172489362	7.674497003	5.923493182
57190	7.203901872	6.7884205	7.619383264	7.445326447	7.407655215	7.482997679
57191	-0.352108471	-0.352108	-0.352108471	5.575983727	5.588224375	5.563743079
57194	4.007024518	7.0634138	10.11980309	-0.352108471	-0.352108471	-0.352108471
57198	5.775231583	7.1708612	4.379601939	6.148282339	7.800049293	6.974165816
57205	-0.352108471	-0.352108	-0.352108471	3.910289569	6.680573626	5.295431597
57212	7.629736298	1.8786265	4.754181416	6.072777462	6.369156591	6.665535719
57213	5.973512349	1.6378164	3.805664353	1.569894481	1.14221151	1.997577452
57214	6.720370627	5.6995671	7.741174168	7.143425013	9.985344146	4.30150588

57215	6.151585628	6.471882	5.831289264	-0.352108471	-0.352108471	-0.352108471
57217	6.414258136	4.4541887	8.374327612	-0.352108471	-0.352108471	-0.352108471
57221	5.156254332	5.0163053	4.876356332	1.245740716	0.440372779	2.051108653
57222	7.676052938	8.7086427	6.643463199	7.849779623	7.944725202	7.754834044
57223	3.255652739	5.2347758	4.245214285	7.962328903	7.063710467	6.165092031
57226	4.77115143	6.199679	7.628206623	0.624614178	0.440372779	0.808855576
57228	-0.352108471	-0.352108	-0.352108471	6.908576198	6.031941504	6.470258851
57231	6.705287512	8.6366275	4.773947538	-0.352108471	-0.352108471	-0.352108471
57282	6.659357577	5.8100237	4.960689758	-0.352108471	-0.352108471	-0.352108471
57291	-0.352108471	-0.352108	-0.352108471	5.55876787	5.588224375	5.529311366
57332	6.926558783	7.2740737	6.579043871	-0.352108471	-0.352108471	-0.352108471
57333	7.3241242	8.1805061	6.467742289	10.7441747	8.20469834	5.665221975
57335	6.262287306	4.9223212	7.602253373	1.382911631	4.404446118	2.893678875
57336	5.355578954	5.2849744	5.214369758	-0.352108471	-0.352108471	-0.352108471
57379	6.133259832	6.0384535	5.94364719	2.094444132	1.061335674	3.127552591
57396	-0.352108471	-0.352108	-0.352108471	4.857389821	4.617382386	4.377374952
57403	7.135878474	5.1818534	6.158865923	3.279366281	4.119353161	3.699359721
57404	7.725216026	8.73717	6.713262076	6.552438858	5.867779085	5.183119313
57407	-0.352108471	-0.352108	-0.352108471	4.943117462	4.482692559	5.403542364
57410	5.625160771	3.3533372	7.896984319	6.245077194	6.731591845	6.48833452
57412	6.139657808	7.3820603	4.897255357	1.622488375	1.305230684	1.939746065
57414	-0.352108471	-0.352108	-0.352108471	3.119148782	5.779327167	0.458970397
57418	-0.352108471	-0.352108	-0.352108471	6.415928975	6.429470164	6.402387785
57419	-0.352108471	-0.352108	-0.352108471	6.619715506	5.372018247	5.995866876
57446	6.477960335	5.7956554	6.136807855	4.054306507	4.791487661	5.528668814
57448	-0.352108471	-0.352108	-0.352108471	4.705495341	7.693862712	6.199679026
57449	4.460216147	7.933593	6.196904592	-0.352108471	-0.352108471	-0.352108471
57452	-0.352108471	-0.352108	-0.352108471	5.983736712	8.331429035	3.636044389
57453	-0.352108471	-0.352108	-0.352108471	5.095423988	0.664917151	9.525930825
57455	2.685732087	1.7810365	3.590427691	1.493791735	0.041890574	2.945692896
57458	6.537383725	6.5498529	6.524914536	-0.352108471	-0.352108471	-0.352108471
57460	-0.352108471	-0.352108	-0.352108471	5.244637527	2.629274231	3.936955879
57461	7.134542387	5.6592789	4.184015356	0.395051519	1.14221151	-0.352108471
57462	9.949345649	6.9586256	8.453985612	5.580270995	10.73871764	8.159494318
57465	7.460743135	7.4538415	7.457292337	6.000797316	6.886095759	7.771394202
57466	-0.352108471	-0.352108	-0.352108471	7.66030187	5.842630242	6.751466056
57467	2.736529761	5.825168	-0.352108471	-0.352108471	-0.352108471	-0.352108471
57469	6.590508304	6.6215439	6.559472732	-0.352108471	-0.352108471	-0.352108471
57470	-0.352108471	-0.352108	-0.352108471	3.228447668	3.337746555	3.119148782
57472	8.784927056	3.0156577	5.90029238	-0.352108471	-0.352108471	-0.352108471
57473	6.623540933	5.8259521	7.421129737	3.59721797	1.939746065	5.254689875
57474	11.05034992	7.9257518	9.488050876	8.832773341	7.149285957	7.991029649
57475	7.8651478	7.1785025	6.491857255	5.264042908	4.335695815	6.19239
57476	-0.352108471	-0.352108	-0.352108471	3.53404206	2.648346333	4.419737788
57478	7.484018122	6.0163629	8.951673352	-0.352108471	-0.352108471	-0.352108471
57479	-0.352108471	-0.352108	-0.352108471	5.635342591	6.893972732	8.152602872
57480	7.305107368	5.882695	8.72751969	1.12335747	0.808855576	1.437859363

57484	5.688622647	6.5370661	4.840179172	-0.352108471	-0.352108471	-0.352108471
57486	6.554662822	5.5701821	7.539143545	3.424613542	6.056569455	8.688525369
57488	5.201388631	10.992924	8.097156385	7.350816905	6.494630027	5.638443149
57489	-0.352108471	-0.352108	-0.352108471	5.088336947	3.119148782	1.149960616
57491	-0.352108471	-0.352108	-0.352108471	6.712406125	7.168934463	6.255877787
57492	3.312874463	4.3155755	5.318276601	6.074265544	4.637194169	3.200122793
57493	7.313984131	8.9694604	5.658507909	6.758264767	5.095423988	3.432583208
57497	-0.352108471	-0.352108	-0.352108471	6.450302837	7.074778174	5.825827501
57506	5.481187704	9.9669721	7.724079888	7.043436743	7.166024253	6.920849233
57507	6.769503656	4.0146191	5.392061359	1.968661759	1.997577452	1.939746065
57514	7.262874357	7.2305473	7.29520141	6.149337851	6.174080069	6.124595632
57515	6.75496194	5.2200029	8.289920969	8.144699842	7.596913147	7.049126453
57520	5.38675695	5.3152588	5.458255064	-0.352108471	-0.352108471	-0.352108471
57522	5.678165805	4.0643007	4.871233248	8.147100384	1.810859062	4.978979723
57523	4.83686721	4.501577	5.172157411	-0.352108471	-0.352108471	-0.352108471
57530	5.089412202	5.1245435	5.054280952	-0.352108471	-0.352108471	-0.352108471
57532	1.383520155	1.3456902	1.421350154	5.883240199	8.808883933	7.346062066
57535	7.805651168	8.4889447	9.172238201	7.57369926	6.156805352	6.865252306
57536	-0.352108471	-0.352108	-0.352108471	4.125531621	4.993866775	4.559699198
57538	7.255495739	6.8312892	6.407082676	-0.352108471	-0.352108471	-0.352108471
57542	7.634810206	6.767634	5.900457851	0.939116071	0.440372779	1.437859363
57545	3.320927332	5.3920006	7.463073909	-0.352108471	-0.352108471	-0.352108471
57546	8.293737092	8.305847	8.299792057	10.21722217	5.221321141	7.719271656
57551	5.398838658	6.2738323	7.14882586	6.296043865	6.225411528	6.366676203
57552	6.682350247	5.2783178	5.980334028	2.847531327	0.440372779	5.254689875
57553	8.699553643	6.1215288	3.543503895	2.55987745	4.975088963	3.767483207
57555	7.351870863	7.352529	7.351212697	6.001809131	6.038453511	5.965164752
57559	8.072934799	6.1014309	7.08718283	-0.352108471	-0.352108471	-0.352108471
57560	6.913488898	5.0649925	8.761985317	-0.352108471	-0.352108471	-0.352108471
57561	-0.352108471	-0.352108	-0.352108471	5.458255064	5.314371021	5.602139107
57562	6.537146105	6.3152311	6.759061079	6.796814879	6.813225323	6.780404436
57563	4.898255012	5.6148381	6.331421246	1.964002179	3.119148782	0.808855576
57567	4.978158736	6.2125362	7.446913681	-0.352108471	-0.352108471	-0.352108471
57571	5.354685649	5.3835036	5.325867653	-0.352108471	-0.352108471	-0.352108471
57573	10.1689822	10.20259	10.13537444	8.645875762	5.934364674	11.35738685
57574	6.55187204	6.4453648	6.658379305	5.723485516	5.731413688	5.715557343
57582	6.262394126	5.6224344	4.982474583	0.289051906	-0.352108471	0.930212283
57583	5.191969759	6.1969436	5.694456662	0.808855576	0.808855576	0.808855576
57584	10.23560443	4.6321238	7.433864099	-0.352108471	-0.352108471	-0.352108471
57585	4.714702999	5.4937832	6.272863431	4.065454755	7.78153161	5.923493182
57586	2.140287079	5.3264422	3.733364623	2.776179788	2.881761131	2.670598445
57587	7.592570356	7.7938865	7.391254223	7.550822982	7.603251574	7.49839439
57591	3.80405037	3.8072783	3.805664353	6.675159846	6.905677231	6.444642462
57594	3.280486615	10.223041	6.75176365	5.047004585	3.70308637	2.359168155
57595	-0.352108471	-0.352108	-0.352108471	6.15007306	5.775231583	6.524914536
57596	5.354685649	5.3835036	5.325867653	-0.352108471	-0.352108471	-0.352108471
57599	6.470581262	3.8677344	5.169157826	-0.352108471	-0.352108471	-0.352108471

57602	-0.352108471	-0.352108	-0.352108471	5.675863648	6.499425261	6.087644454
57604	11.14795557	7.2688675	3.389779407	5.479099849	7.624975512	6.55203768
57606	5.652254201	6.1923204	6.732386524	4.456886199	4.482692559	4.43107984
57608	6.977258204	7.220849	6.733667396	-0.352108471	-0.352108471	-0.352108471
57609	6.334127991	5.5587679	4.783407749	-0.352108471	-0.352108471	-0.352108471
57611	7.174096023	6.0203811	4.86666616	1.935165301	0.151687366	1.043426334
57613	6.269206667	3.991667	8.546746304	6.598490492	4.997626553	5.798058523
57615	7.480440003	8.0484881	8.616536154	6.687409375	5.405185664	7.969633085
57617	6.774139572	6.3968684	6.019597193	7.289078056	9.390152264	5.188003847
57619	7.04456855	4.2290497	5.636809111	-0.352108471	-0.352108471	-0.352108471
57620	7.546524525	4.8214035	6.183964016	-0.352108471	-0.352108471	-0.352108471
57621	7.218932222	3.2160501	5.217491139	-0.352108471	-0.352108471	-0.352108471
57623	2.621772913	2.2060694	2.413921152	5.958359385	7.451412154	4.465306615
57624	-0.352108471	-0.352108	-0.352108471	5.958359385	5.958359385	5.958359385
57630	5.292183578	6.9134889	8.534794219	-0.352108471	-0.352108471	-0.352108471
57633	5.428393874	3.9261994	6.930588353	-0.352108471	-0.352108471	-0.352108471
57634	6.234419216	6.5021238	6.769828314	6.12990004	5.547614426	6.712185655
57636	6.183753922	8.2977819	7.240767906	6.840114058	7.529013824	6.151214292
57643	3.486368175	2.4139212	1.341474129	5.335209685	7.565046041	3.105373328
57646	-0.352108471	-0.352108	-0.352108471	6.366776401	6.392114231	6.341438571
57647	-0.352108471	-0.352108	-0.352108471	7.852268327	4.461252742	6.156760535
57649	5.152247113	5.1799508	5.124543452	-0.352108471	-0.352108471	-0.352108471
57658	7.034930127	5.9629396	6.498934874	5.232499485	6.344802955	5.78865122
57659	6.095486511	4.6929891	3.290491727	8.696440088	4.343505502	6.519972795
57661	-0.352108471	-0.352108	-0.352108471	8.059424863	3.520100589	5.789762726
57665	2.239158726	0.4653926	4.01292488	-0.352108471	-0.352108471	-0.352108471
57666	7.062672095	7.1689345	6.956409727	-0.352108471	-0.352108471	-0.352108471
57669	6.42925664	3.6050162	5.017136436	-0.352108471	-0.352108471	-0.352108471
57674	6.720598625	5.4528725	7.98832475	6.999651261	6.849024193	6.698397125
57679	-0.352108471	-0.352108	-0.352108471	6.713776402	9.484284184	3.94326862
57680	-0.352108471	-0.352108	-0.352108471	7.349994721	6.472017122	5.594039522
57683	6.564883227	6.4665022	6.368121182	1.12335747	0.808855576	1.437859363
57687	5.870080727	6.8682325	6.369156591	-0.352108471	-0.352108471	-0.352108471
57688	5.639271032	6.1075388	6.57580657	7.725173814	6.474238624	7.099706219
57693	-0.352108471	-0.352108	-0.352108471	5.923493182	3.483613141	8.363373224
57696	7.435794968	5.1710352	6.303415073	-0.352108471	-0.352108471	-0.352108471
57701	6.183964016	6.1999612	6.167966808	5.88700099	5.893442277	5.880559702
57703	5.852937462	5.873346	5.832528937	-0.352108471	-0.352108471	-0.352108471
57704	5.780555051	6.7671095	6.273832259	-0.352108471	-0.352108471	-0.352108471
57707	5.645917886	6.6142706	7.582623214	5.299668551	5.767479853	6.235291155
57709	-0.352108471	-0.352108	-0.352108471	0.940233577	2.232575625	-0.352108471
57710	6.641264752	7.244076	6.038453511	5.774127426	5.926961306	5.621293547
57711	3.636824788	3.3494067	3.924242887	1.403216514	1.727071877	1.079361152
57713	4.716952933	8.2920796	6.504516254	8.367462895	3.961113643	6.164288269
57720	8.954808956	6.3404241	7.64761654	0.624614178	0.808855576	0.440372779
57722	5.314533813	4.9899604	5.152247113	-0.352108471	-0.352108471	-0.352108471
57724	-0.352108471	-0.352108	-0.352108471	6.912349504	4.555995245	5.734172375

57731	-0.352108471	-0.352108	-0.352108471	5.600955507	5.412561087	5.224166668
57761	5.217491139	5.6588972	4.776085034	4.121550341	3.501080718	4.742019963
57787	-0.352108471	-0.352108	-0.352108471	6.908633468	7.318948842	7.729264217
57798	7.644075882	8.6774638	8.160769863	7.039551591	7.739290074	6.339813108
57801	9.148553976	6.3034612	3.458368513	-0.352108471	-0.352108471	-0.352108471
57804	5.440913558	7.9747332	10.50855278	7.784173954	5.639964005	6.712068979
57805	-0.352108471	-0.352108	-0.352108471	6.936660808	7.217419545	6.655902072
57819	5.911656798	6.5905083	7.26935981	-0.352108471	-0.352108471	-0.352108471
57824	2.313941724	3.1433048	1.484578692	-0.352108471	-0.352108471	-0.352108471
57826	2.436716207	4.0537544	3.245235305	6.180373585	8.76687395	3.593873219
57835	5.837198182	6.8224388	4.851957573	3.87211091	6.114937456	1.629284364
57862	3.117396088	4.2010992	5.284802407	6.08233239	6.498042382	6.290187386
57864	4.998688086	7.5489764	6.273832259	-0.352108471	-0.352108471	-0.352108471
58155	-0.352108471	-0.352108	-0.352108471	3.585121301	7.265262643	-0.095020041
58157	-0.352108471	-0.352108	-0.352108471	3.384482817	3.615460478	3.153505157
58160	-0.352108471	-0.352108	-0.352108471	2.065292044	4.482692559	-0.352108471
58189	6.573968999	9.4943801	3.6535579	5.368985197	5.095423988	4.821862779
58190	3.515141236	5.9565521	1.07373037	5.723485516	3.702945908	7.744025123
58472	-0.352108471	-0.352108	-0.352108471	6.250282655	6.081458557	6.165870606
58475	2.896293591	6.7443331	4.820313327	1.825329981	2.100935855	1.549724107
58476	-0.352108471	-0.352108	-0.352108471	7.375497935	8.578655479	7.977076707
58477	-0.352108471	-0.352108	-0.352108471	5.657493881	7.776478226	3.538509535
58485	-0.352108471	-0.352108	-0.352108471	7.364389464	7.386082653	7.342696275
58490	6.020381091	5.2555426	6.785219545	-0.352108471	-0.352108471	-0.352108471
58493	6.32682969	8.3367008	7.331765268	5.247026494	3.700559908	2.154093323
58494	6.81568939	6.6788995	6.747294441	2.546625611	1.939746065	3.153505157
58497	5.7425783	4.8270489	5.284813592	-0.352108471	-0.352108471	-0.352108471
58499	6.166257062	6.9471227	6.556689902	-0.352108471	-0.352108471	-0.352108471
58500	6.285494677	6.9148456	6.600170153	3.793686399	5.591305588	7.388924778
58504	6.147197569	5.5587679	4.970338171	-0.352108471	-0.352108471	-0.352108471
58505	6.234628092	4.8786655	3.522702864	0.920492535	3.245625856	5.570759177
58506	8.407301102	4.3584459	6.382873477	7.702721441	8.377575324	9.052429207
58508	-0.352108471	-0.352108	-0.352108471	1.806061184	5.575983727	9.345906269
58516	6.40271776	7.9426509	9.482584113	2.06022916	1.848794271	1.637359382
58517	5.800199788	8.4195016	3.180897966	4.182387884	6.345315925	5.263851904
58526	-0.352108471	-0.352108	-0.352108471	10.58638679	4.520706318	7.553546556
58527	1.66201518	0.1499869	3.174043454	3.174536588	2.655229797	2.135923007
58528	-0.352108471	-0.352108	-0.352108471	6.047498583	0.884777956	3.46613827
58533	10.49052215	7.3981936	4.305865088	6.235921161	6.351526092	6.12031623
58985	-0.352108471	-0.352108	-0.352108471	1.783698732	-1.314499333	4.881896797
58986	6.815350011	6.502263	6.189176046	7.707102448	7.731338116	7.682866779
59274	7.548473852	5.1898393	6.369156591	7.581174592	7.289078056	6.996981519
59277	2.772692854	5.2174911	7.662289424	-0.352108471	-0.352108471	-0.352108471
59283	6.32247105	6.3831515	6.261790604	6.32450851	6.246629235	6.402387785
59285	4.587224297	4.1637749	3.740325419	-0.352108471	-0.352108471	-0.352108471
59286	6.801813774	6.7710365	6.832590998	-0.352108471	-0.352108471	-0.352108471
59338	4.911650208	7.006006	2.817294404	2.78581638	4.885343373	3.835579877

59339	5.595865775	5.8522299	6.1085941	6.697621258	8.469023497	4.92621902
59340	4.656717726	5.7256773	3.587758183	2.640854735	3.27996028	3.919065824
59349	-0.352108471	-0.352108	-0.352108471	7.20924711	6.990165328	7.099706219
60312	3.977894487	4.5378201	5.09774572	5.339363025	9.044441178	7.191902102
60313	6.537383725	6.8266121	6.248155349	5.174103199	5.760537073	6.346970947
60314	-0.352108471	-0.352108	-0.352108471	6.396868383	6.410585269	6.383151496
60412	-0.352108471	-0.352108	-0.352108471	5.789762726	5.804293869	5.775231583
60436	9.533345056	6.8022253	4.071105627	0.044132154	-0.352108471	0.440372779
60481	-0.352108471	-0.352108	-0.352108471	7.577433573	3.161230604	5.369332089
60487	-0.352108471	-0.352108	-0.352108471	6.577483275	7.86280741	5.292159141
60490	-0.352108471	-0.352108	-0.352108471	5.775068216	4.765707108	6.784429323
60491	7.639511791	6.1515856	4.663659466	-0.352108471	-0.352108471	-0.352108471
60492	8.603707241	8.8371526	8.370261881	5.973500237	5.873345987	6.073654487
60493	1.392109249	1.5880026	1.196215903	6.045783324	5.845499835	5.645216346
60526	-0.352108471	-0.352108	-0.352108471	6.752184247	5.760537073	4.768889898
60528	5.745277164	6.5373837	7.329490285	-0.352108471	-0.352108471	-0.352108471
60558	6.114367042	5.4937832	4.873199387	4.178899946	7.074324057	1.283475834
60559	6.065707629	6.8767254	5.254689875	7.785987102	7.827584812	7.744389392
60560	6.090808341	6.1079622	6.073654487	-0.352108471	-0.352108471	-0.352108471
60592	6.242048279	4.5278264	7.956270112	-0.352108471	-0.352108471	-0.352108471
60626	7.466207166	7.474041	7.458373336	6.735779686	6.750862801	6.720696571
60672	-0.352108471	-0.352108	-0.352108471	8.325134299	5.233567424	2.142000548
60673	5.652870912	9.5334287	7.593149813	7.212946788	6.450302837	5.687658887
60678	-0.352108471	-0.352108	-0.352108471	4.161183828	8.310658494	6.235921161
60681	7.089794111	7.7423759	8.394957618	6.241870568	10.45440747	8.348139018
60685	3.140892121	3.1277379	3.114583629	9.959656043	5.695375042	7.827515543
63875	4.165056742	6.6073775	9.049698176	6.275406712	8.463204971	7.369305842
63876	-0.352108471	-0.352108	-0.352108471	0.830820342	3.355865518	5.880910695
63891	-0.352108471	-0.352108	-0.352108471	6.022393352	6.668157563	6.345275458
63895	6.090808341	9.1352748	3.04634185	-0.352108471	-0.352108471	-0.352108471
63897	-0.352108471	-0.352108	-0.352108471	4.307390425	6.431273752	5.369332089
63898	6.433318728	6.4453648	6.421272681	-0.352108471	-0.352108471	-0.352108471
63901	6.996816126	3.7304858	10.26314646	3.496718474	-0.686712309	7.680149257
63905	5.294336754	5.1522471	5.010157472	5.614929861	7.347612652	9.080295444
63908	-0.352108471	-0.352108	-0.352108471	2.204376426	-0.352108471	4.760861323
63916	-0.352108471	-0.352108	-0.352108471	7.357359337	11.69849342	3.016225257
63917	7.482027287	6.1955411	4.909054948	6.824147952	6.845389604	6.802906299
63923	5.688622647	4.6402083	6.737037001	-0.352108471	-0.352108471	-0.352108471
63925	-0.352108471	-0.352108	-0.352108471	5.824288785	5.608716946	6.039860624
63928	4.646989744	7.0614187	9.475847744	2.845098058	3.5616058	4.278113542
63929	6.887774551	8.8769145	10.86605448	10.14841176	8.132941777	6.117471795
63932	8.707241083	7.4451024	8.076171744	7.282430292	7.856913531	7.569671911
63933	7.422618389	8.1578931	7.790255725	6.585211616	5.271561728	7.898861504
63935	-0.352108471	-0.352108	-0.352108471	4.456886199	6.016705236	2.897067163
63939	6.939918434	7.2682981	6.611538781	8.383495281	7.395881893	6.408268504
63940	-0.352108471	-0.352108	-0.352108471	7.435582094	5.55876787	3.681953646
63943	1.860013666	3.3848996	2.62245663	-0.352108471	-0.352108471	-0.352108471

63950	-0.352108471	-0.352108	-0.352108471	1.167362396	2.686833264	-0.352108471
63967	6.719923116	6.4923644	6.606143757	4.49290523	4.620365567	4.365444893
64062	8.370154701	6.183964	3.99777333	-0.352108471	-0.352108471	-0.352108471
64077	-0.352108471	-0.352108	-0.352108471	6.8287835	4.66005769	2.491331879
64080	-0.352108471	-0.352108	-0.352108471	2.204376426	-0.352108471	4.760861323
64083	6.779229539	6.0874079	6.433318728	4.557244691	4.980903231	4.13358615
64084	8.034490159	4.4796025	6.257046343	-0.352108471	-0.352108471	-0.352108471
64089	-0.352108471	-0.352108	-0.352108471	6.415584166	10.20914208	2.622026249
64097	0.254673727	5.2183858	2.736529761	-0.352108471	-0.352108471	-0.352108471
64098	6.446033496	6.2911804	6.368606935	1.992178665	1.731739118	2.252618212
64106	8.163571973	6.6400194	5.116466812	-0.352108471	-0.352108471	-0.352108471
64108	-0.352108471	-0.352108	-0.352108471	3.490394555	6.32531018	9.160225805
64109	-0.352108471	-0.352108	-0.352108471	2.672987808	7.182370621	4.927679215
64114	8.279007428	7.0871828	5.895358231	7.110592173	7.379014829	6.842169517
64115	5.354685649	5.3835036	5.325867653	7.484728707	7.516025064	7.453432349
64121	-0.352108471	-0.352108	-0.352108471	2.748644951	3.489652612	3.119148782
64123	3.177906891	7.1265873	5.152247113	-0.352108471	-0.352108471	-0.352108471
64131	5.749402114	5.7752316	5.723572646	-0.352108471	-0.352108471	-0.352108471
64145	6.712704044	7.0903874	6.901545707	6.351797047	6.545570313	6.44868368
64149	9.061334584	8.1328188	7.204302972	5.978341452	9.593141867	7.78574166
64151	-0.352108471	-0.352108	-0.352108471	7.871562408	3.649511737	5.760537073
64167	5.093120047	4.1125856	6.073654487	5.738663982	6.497336045	4.979991919
64175	4.45328559	4.9470072	5.440728789	3.090683483	2.169424975	1.248166466
64207	9.551450267	6.6114826	8.081466428	0.044132154	0.440372779	-0.352108471
64208	-0.352108471	-0.352108	-0.352108471	4.951503876	5.515630971	5.233567424
64210	-0.352108471	-0.352108	-0.352108471	2.024343053	2.051108653	1.997577452
64215	6.317834233	5.6413476	6.994320828	6.756010887	6.407361112	6.058711337
64219	-0.352108471	-0.352108	-0.352108471	5.88700099	5.262629658	6.511372322
64220	5.750073398	8.7882338	7.269153621	-0.352108471	-0.352108471	-0.352108471
64222	-0.352108471	-0.352108	-0.352108471	8.145718949	6.55174816	4.957777371
64223	5.681727979	5.5882244	5.775231583	5.219901144	5.124543452	5.315258837
64224	5.22867225	5.7398526	6.251032887	0.044132154	-0.352108471	0.440372779
64225	5.018877004	6.7161533	8.413429695	3.409696216	8.014830639	5.712263427
64240	-0.352108471	-0.352108	-0.352108471	7.558491418	6.415928975	5.273366531
64241	2.891547989	-0.352108	6.135204448	0.440372779	0.440372779	0.440372779
64285	-0.352108471	-0.352108	-0.352108471	1.964002179	0.808855576	3.119148782
64319	8.126098466	6.7054902	7.415794329	8.3861716	7.821164041	7.256156482
64320	-0.352108471	-0.352108	-0.352108471	4.250799937	8.707720816	6.479260377
64324	-0.352108471	-0.352108	-0.352108471	4.574260186	6.186562848	7.798865511
64326	-0.352108471	-0.352108	-0.352108471	2.477574272	3.038779864	3.599985456
64332	5.114366655	6.3022478	5.708307224	-0.352108471	-0.352108471	-0.352108471
64333	2.91562497	2.874763	2.833900981	-0.352108471	-0.352108471	-0.352108471
64343	7.550691998	7.6019333	7.653174666	-0.352108471	-0.352108471	-0.352108471
64344	6.84643295	5.4887327	6.167582836	0.86953393	0.808855576	0.930212283
64359	8.96094041	6.7922743	7.876607375	3.203502594	7.893737128	12.58397166
64374	2.752301789	3.503174	3.127737875	8.854669181	8.171122555	7.487575928
64376	-0.352108471	-0.352108	-0.352108471	1.492819042	1.455244114	1.53039397

64393	8.01968687	7.3896415	8.64973221	7.883534115	7.909550197	7.857518032
64395	3.527916452	3.136327	3.919505936	1.80041638	1.549724107	2.051108653
64397	8.202363764	6.7024312	5.202498572	6.373195497	4.413101083	8.333289911
64398	-0.352108471	-0.352108	-0.352108471	1.146579317	4.418336914	2.782458115
64403	3.8441738	4.9470072	6.049840578	-0.352108471	-0.352108471	-0.352108471
64409	-0.352108471	-0.352108	-0.352108471	6.606648584	7.344598048	6.975623316
64412	-0.352108471	-0.352108	-0.352108471	6.266367194	6.276856601	6.255877787
64418	7.680437959	6.8630334	6.045628857	5.689860511	6.30721667	5.99853859
64421	5.130802973	4.5462888	3.961774553	6.772537347	7.094871452	6.9337044
64422	6.37517147	6.6741289	6.973086287	6.424941448	6.167146283	6.296043865
64423	-0.352108471	-0.352108	-0.352108471	6.545950789	6.614414028	6.477487549
64425	-0.352108471	-0.352108	-0.352108471	4.121846117	3.772970267	4.470721967
64427	6.607377459	3.8233977	9.391357177	-0.352108471	-0.352108471	-0.352108471
64429	4.055460084	5.9788128	5.017136436	8.691769498	7.304663518	5.917557537
64430	-0.352108471	-0.352108	-0.352108471	6.556689902	6.588465267	6.524914536
64432	-0.430338958	2.2456	4.921538984	-0.352108471	-0.352108471	-0.352108471
64434	6.083202488	8.4963522	7.289777346	6.830258893	6.401088366	7.25942942
64579	-0.352108471	-0.352108	-0.352108471	6.153217526	5.494417177	4.835616827
64581	4.869400283	7.6840754	6.276737847	1.657360368	1.876861374	1.437859363
64595	4.5088668	5.8497669	3.167966703	6.442904904	6.146874307	5.85084371
64598	-0.352108471	-0.352108	-0.352108471	4.693340309	5.596916991	6.500493674
64599	6.723786325	4.7138212	5.718803747	0.044132154	0.440372779	-0.352108471
64600	5.284974356	4.6977248	5.872223893	-0.352108471	-0.352108471	-0.352108471
64645	6.346998353	5.1245435	3.90208855	6.790575708	5.907806919	6.349191313
64648	-0.352108471	-0.352108	-0.352108471	4.178899946	4.245214285	4.112585606
64651	1.392109249	3.136327	-0.352108471	3.790723471	6.676411376	5.233567424
64682	-0.352108471	-0.352108	-0.352108471	3.642300096	4.245563566	4.848827036
64689	-0.352108471	-0.352108	-0.352108471	7.478462511	7.49196897	7.464956052
64708	6.414258136	4.4743412	8.354175031	5.768037081	5.057085094	5.412561087
64710	5.278710445	3.3524406	4.315575532	7.89873015	7.127759146	6.356788142
64718	5.77329188	5.5882244	5.958359385	1.842407068	1.876861374	1.807952763
64743	7.159460696	7.1121207	7.064780794	-0.352108471	-0.352108471	-0.352108471
64744	3.814304253	5.4584815	7.102658732	-0.352108471	-0.352108471	-0.352108471
64746	-0.352108471	-0.352108	-0.352108471	7.273593076	5.325716078	3.37783908
64748	7.774792467	7.0167764	6.258760367	0.440372779	0.440372779	0.440372779
64750	2.761190761	10.347051	6.554121048	5.666775296	4.98465686	5.325716078
64755	-0.352108471	-0.352108	-0.352108471	6.88036327	6.463674915	6.04698656
64756	5.566834031	7.0400885	6.303461245	5.88700099	7.24829239	4.52570959
64757	-0.352108471	-0.352108	-0.352108471	0.919601171	-0.352108471	2.191310813
64759	-0.352108471	-0.352108	-0.352108471	7.411911832	6.220238598	5.028565365
64760	-0.352108471	-0.352108	-0.352108471	5.723485516	5.731413688	5.715557343
64763	-0.352108471	-0.352108	-0.352108471	7.102622694	5.824727136	6.463674915
64766	7.146480757	6.0304897	8.262471772	1.90117116	1.549724107	2.252618212
64769	6.250777728	5.689482	5.128186251	0.536727595	2.958541694	5.380355793
64773	-0.352108471	-0.352108	-0.352108471	6.873298864	5.123514031	5.998406448
64777	-0.352108471	-0.352108	-0.352108471	8.672755728	-0.314955837	4.178899946
64778	5.939192221	6.0730906	5.805293798	-0.352108471	-0.352108471	-0.352108471

64779	-0.352108471	-0.352108	-0.352108471	4.822946739	5.767916455	5.295431597
64781	6.972090567	7.0231519	6.92102926	7.094095983	7.064440118	7.034784252
64782	-0.352108471	-0.352108	-0.352108471	6.406486527	6.410585269	6.402387785
64783	-0.352108471	-0.352108	-0.352108471	1.783698732	3.711361887	-0.143964422
64784	2.465395097	5.2174911	7.969587181	-0.352108471	-0.352108471	-0.352108471
64786	5.537882078	8.5567546	7.047318347	7.683080299	6.713119361	5.743158423
64792	4.590848545	7.4137688	6.002308672	3.788235057	3.169499624	2.55076419
64793	5.005505193	7.0043403	9.003175473	-0.352108471	-0.352108471	-0.352108471
64798	2.794084087	2.3510862	1.908088394	-0.352108471	-0.352108471	-0.352108471
64799	-0.352108471	-0.352108	-0.352108471	2.947260382	4.887790674	1.00673009
64801	-0.352108471	-0.352108	-0.352108471	5.613451577	6.19403316	5.032869994
64802	6.857594311	8.2623707	9.667147028	7.11375507	6.61633976	7.611170381
64834	4.641167391	9.6742478	7.15770761	8.442141746	6.836213819	5.230285892
64837	-0.352108471	-0.352108	-0.352108471	4.057995846	0.736988586	2.397492216
64840	5.950174623	7.7989976	6.874586128	-0.352108471	-0.352108471	-0.352108471
64841	5.003627844	5.5089082	5.256268008	3.010220895	2.100935855	3.919505936
64848	-0.352108471	-0.352108	-0.352108471	6.949901658	6.878181404	7.021621912
64852	2.928049433	9.1127127	6.020381091	-0.352108471	-0.352108471	-0.352108471
64853	7.726306258	7.839781	7.953255701	7.003799972	5.034974955	8.97262499
64854	4.729831277	7.0707535	5.90029238	1.345967809	1.14221151	1.549724107
64855	10.04867405	8.137867	6.227060046	10.16997508	8.418446513	6.666917941
64856	-0.352108471	-0.352108	-0.352108471	6.692644478	6.32531018	5.957975882
64857	8.804505221	6.9900309	7.897268052	5.367886754	4.566323711	4.967105233
64860	-0.352108471	-0.352108	-0.352108471	6.469652106	1.888147785	4.178899946
64864	6.674128879	6.1746037	7.173654103	4.80208491	5.012286671	4.591883149
64866	6.503697469	5.7545534	5.00540933	0.289051906	-0.352108471	0.930212283
64881	5.961166865	6.1082995	5.814034224	-0.352108471	-0.352108471	-0.352108471
64897	7.339129632	4.1000004	5.719564994	-0.352108471	-0.352108471	-0.352108471
64900	5.417023759	6.9950766	3.838970952	0.484598105	2.795182383	5.105766662
64919	7.539035592	7.2078382	6.876640731	5.20089139	5.254689875	5.147092904
64922	7.033116437	5.6886226	4.344128857	2.588069749	3.20829756	2.898183655
64925	6.537383725	6.7158643	6.358903141	0.044132154	0.440372779	-0.352108471
64926	4.778807391	7.1818607	5.980334028	-0.352108471	-0.352108471	-0.352108471
64927	8.668305562	6.8119539	4.955602246	6.860193211	6.211642887	7.508743534
64928	7.561151448	6.7871341	6.013116698	7.495082972	5.958359385	4.421635797
64940	6.433318728	6.4453648	6.421272681	-0.352108471	-0.352108471	-0.352108471
64943	-0.352108471	-0.352108	-0.352108471	4.915509878	5.493783215	6.072056552
64946	2.139844176	0.2514863	4.028202024	9.094706106	7.335962107	5.577218107
64951	4.755240115	5.2146317	4.295848569	-0.352108471	-0.352108471	-0.352108471
64960	6.239980599	5.8721274	6.056053999	1.036211897	1.14221151	0.930212283
64963	6.787134073	7.3538527	6.220415432	-0.352108471	-0.352108471	-0.352108471
64969	-0.352108471	-0.352108	-0.352108471	7.970563352	4.927679215	1.884795078
64975	-0.352108471	-0.352108	-0.352108471	6.796814879	7.489832597	6.103797162
64976	-0.352108471	-0.352108	-0.352108471	7.798509736	4.429012023	6.113760879
64978	-0.352108471	-0.352108	-0.352108471	5.785505277	5.825167992	5.745842563
64981	-0.352108471	-0.352108	-0.352108471	6.47267644	6.829513733	6.115839148
64983	-0.352108471	-0.352108	-0.352108471	5.704335255	5.715557343	5.693113166

65003	-0.352108471	-0.352108	-0.352108471	6.656376543	6.165870606	5.675364669
65008	-0.352108471	-0.352108	-0.352108471	6.886610445	6.900175883	6.873045007
65009	-0.352108471	-0.352108	-0.352108471	5.821410838	7.007140474	4.635681202
65010	3.622310641	2.0992354	2.860773008	0.044132154	-0.352108471	0.440372779
65059	2.553073296	-0.352108	5.458255064	6.40196491	6.215464554	6.588465267
65062	-0.352108471	-0.352108	-0.352108471	5.107416872	6.666585107	5.88700099
65065	5.961166865	9.0280216	2.894312103	-0.352108471	-0.352108471	-0.352108471
65078	3.964368457	5.7494021	7.534435771	-0.352108471	-0.352108471	-0.352108471
65080	4.616151079	7.0978125	9.579473981	0.808855576	0.808855576	0.808855576
65082	6.488053522	5.4309597	5.959506607	5.51540809	7.938549709	3.092266471
65083	-0.352108471	-0.352108	-0.352108471	4.920065843	7.040602213	5.980334028
65084	5.90029238	7.8436656	3.956919138	6.165870606	4.860612601	7.471128611
65095	-0.352108471	-0.352108	-0.352108471	3.585121301	3.615460478	3.554782125
65108	7.997293817	5.3509639	6.674128879	3.050245359	3.188052205	3.119148782
65110	7.398848825	6.8860958	6.373342693	4.964006879	7.367734333	6.165870606
65117	-0.352108471	-0.352108	-0.352108471	8.220781697	7.063286202	5.905790708
65123	-0.352108471	-0.352108	-0.352108471	3.02968186	2.076022254	1.122362649
65124	6.415584166	6.2748379	6.556330467	8.788876079	4.174171811	6.481523945
65125	-0.352108471	-0.352108	-0.352108471	2.252618212	2.252618212	2.252618212
65244	8.22223116	7.5277813	6.833331358	7.121742116	8.581877728	5.661606504
65250	6.861057494	6.8767254	6.845389604	5.723485516	5.731413688	5.715557343
65258	2.553073296	3.555049	1.55109759	-0.352108471	-0.352108471	-0.352108471
65260	6.909009051	9.1173919	4.700626177	9.091042088	5.049796103	7.070419096
65263	5.62065828	4.3393256	4.979991919	0.639342438	2.052593179	1.345967809
65265	7.825383622	7.783254	7.867513291	6.361035528	6.841511354	5.880559702
65266	5.852937462	6.9601805	4.745694415	-0.352108471	-0.352108471	-0.352108471
65267	-0.352108471	-0.352108	-0.352108471	6.56348723	3.838990613	5.201238922
65268	-0.352108471	-0.352108	-0.352108471	-0.503747661	5.205920142	2.351086241
65979	8.470122167	9.7546676	7.185576688	6.627732289	8.625050135	10.62236798
65981	-0.352108471	-0.352108	-0.352108471	6.47523743	5.264093599	5.869665514
65983	0.320585424	1.5010176	2.681449745	2.076022254	0.739174258	3.41287025
65985	-0.352108471	-0.352108	-0.352108471	5.396341388	7.397395377	6.396868383
65988	5.977600527	6.1515856	6.32557073	4.973314835	5.509951389	5.241633112
65990	-0.352108471	-0.352108	-0.352108471	2.951536416	1.366941702	4.536131131
65992	-0.352108471	-0.352108	-0.352108471	6.095536005	5.623698733	5.859617369
65993	-0.352108471	-0.352108	-0.352108471	6.952349711	7.294621465	7.123485588
65996	5.566433708	8.137505	6.85196936	6.837896976	8.204674434	7.521285705
65998	-0.352108471	-0.352108	-0.352108471	5.800199788	7.486765233	4.113634342
66005	-0.352108471	-0.352108	-0.352108471	6.713776402	6.729085562	6.698467242
66008	5.054754712	4.8658504	5.243659007	-0.352108471	-0.352108471	-0.352108471
66036	7.638475353	7.4782435	7.798707247	6.292710438	6.116150736	5.939591035
78988	5.883690331	5.9530917	6.022493092	7.390676288	8.519854519	6.261498057
78992	-0.352108471	-0.352108	-0.352108471	6.614003991	7.054600764	7.495197537
78994	7.652552373	5.8565647	6.754558526	-0.352108471	-0.352108471	-0.352108471
78998	5.656289537	5.7155573	5.597021732	1.218975116	0.440372779	1.997577452
78999	4.947007189	6.4806504	3.413363971	7.206823982	6.949901658	6.692979334
79000	3.41407487	3.136327	3.69182277	6.31523113	6.31523113	6.31523113

79001	6.946351317	7.1577076	7.369063902	7.887147635	9.036381991	6.737913278
79002	8.399665392	8.4498164	8.349514391	7.191902102	7.21486974	7.168934463
79004	-0.352108471	-0.352108	-0.352108471	5.663737322	5.665786693	5.661687951
79005	6.256109845	6.4105853	6.101634422	6.014753478	5.804293869	6.225213087
79006	7.012887374	6.4636749	5.914462456	5.895237137	6.77374078	7.652244423
79009	-0.352108471	-0.352108	-0.352108471	8.797896667	5.760537073	2.723177479
79016	7.365949534	6.6350594	8.096839644	9.193027696	7.080117661	4.967207626
79022	4.640845938	6.3178342	7.994822528	-0.352108471	-0.352108471	-0.352108471
79026	9.143898825	7.019034	8.081466428	8.215261918	8.923130714	7.507393122
79029	2.553073296	0.6439336	4.462212988	-0.352108471	-0.352108471	-0.352108471
79031	-0.352108471	-0.352108	-0.352108471	2.784130118	4.857539934	6.930949751
79035	4.731111372	2.7226036	6.739619152	6.609358453	6.868836921	7.128315389
79036	4.863226045	2.40894	7.3175121	7.123485588	6.344483658	7.902487518
79038	-0.352108471	-0.352108	-0.352108471	5.958359385	5.958359385	5.958359385
79041	4.962148436	6.0932891	7.22442972	7.166552573	5.523336263	3.880119952
79042	8.108157606	5.072859	6.590508304	6.981888777	6.288881886	7.674895668
79047	-0.352108471	-0.352108	-0.352108471	7.319522177	6.359270363	5.399018549
79050	-0.352108471	-0.352108	-0.352108471	7.50587715	7.528756621	7.482997679
79054	3.654774043	6.2372177	8.819661403	5.458255064	7.150025221	3.766484907
79056	5.163777147	5.7282969	6.292816736	1.184035823	1.437859363	0.930212283
79058	-0.352108471	-0.352108	-0.352108471	2.686833264	3.288537255	2.085129272
79064	6.816905042	5.337374	8.296436083	5.821410838	6.485414236	5.15740744
79065	6.758702766	6.8449248	6.801813774	0.044132154	0.440372779	-0.352108471
79066	5.645392	4.9088628	6.381921183	2.92016921	2.686833264	3.153505157
79068	9.452464317	5.7078873	7.580175832	6.448016836	5.479280235	7.416753437
79071	7.042523083	7.0441776	7.040868605	0.395051519	1.14221151	-0.352108471
79073	7.567545379	6.9375473	8.197543502	6.630819448	6.560204177	6.489588906
79075	-0.352108471	-0.352108	-0.352108471	5.630463094	4.927679215	4.224895336
79077	6.519673958	6.5498529	6.489495002	7.063286202	7.082394844	7.044177561
79078	-0.352108471	-0.352108	-0.352108471	5.944722576	5.97880156	5.961762068
79080	4.924617113	4.3065499	4.615583513	7.186511196	7.129941003	7.243081389
79083	8.067058037	7.3471469	7.707102448	3.895746505	5.775068216	7.654389926
79084	-0.352108471	-0.352108	-0.352108471	6.434644758	6.448016836	6.421272681
79086	4.578605156	2.4139212	0.249237147	3.010220895	1.494171942	4.526269849
79088	4.731342239	5.0591577	4.403526732	1.556591723	1.66308105	1.450102397
79089	6.134800615	6.1679668	6.101634422	6.56915909	6.588465267	6.549852913
79094	3.54921092	5.0819401	4.315575532	2.124237496	2.147539136	2.100935855
79095	-0.352108471	-0.352108	-0.352108471	6.532441983	5.84169629	7.223187677
79101	-0.352108471	-0.352108	-0.352108471	6.070043966	6.101634422	6.038453511
79102	-0.352108471	-0.352108	-0.352108471	7.517670946	7.483479721	7.551862171
79109	4.160573095	6.2744092	5.217491139	5.712263427	3.082204507	8.342322347
79142	7.875021286	9.1869421	6.563100437	-0.352108471	-0.352108471	-0.352108471
79143	1.383520155	-0.352108	3.119148782	6.912551088	8.514346153	10.11614122
79144	7.673298345	6.9779046	8.368692133	9.27518472	8.202186715	7.12918871
79153	5.54638729	7.7006946	6.623540933	-0.352108471	-0.352108471	-0.352108471
79155	6.199595632	6.2312245	6.167966808	-0.352108471	-0.352108471	-0.352108471
79157	4.805894371	4.9799919	4.631796822	2.096182139	1.939746065	2.252618212

79158	6.494850053	7.2488467	8.0028434	7.69235088	7.305415326	6.918479773
79159	8.90093852	3.2468215	6.073880033	8.139974393	6.382981583	7.261477988
79171	6.183964016	8.4512467	3.91668132	6.296043865	6.636146932	5.955940799
79174	-0.352108471	-0.352108	-0.352108471	4.977710418	5.726671899	4.228748937
79175	5.980334028	4.1482081	7.812459965	-0.352108471	-0.352108471	-0.352108471
79176	4.304520476	5.0894122	5.874303927	4.456886199	4.055673818	4.858098581
79180	5.896777473	6.6400194	7.383261312	8.03804272	6.769641641	5.501240562
79183	6.512147343	5.8724934	6.192320362	6.067050902	6.790856466	7.514662029
79188	6.020381091	5.6303835	6.41037867	7.877482668	5.848389372	6.86293602
79192	7.723635071	5.280891	6.502263028	-0.352108471	-0.352108471	-0.352108471
79228	-0.352108471	-0.352108	-0.352108471	5.859261334	5.199361398	5.529311366
79230	7.40101804	3.8324686	5.616743323	2.498005078	1.876861374	3.119148782
79269	8.445297939	6.3931751	10.49742082	7.337230787	4.955462599	9.718998975
79365	-0.352108471	-0.352108	-0.352108471	6.296043865	4.27316396	8.318923771
79411	5.085607333	4.9799919	5.191222747	1.58479924	1.731739118	1.437859363
79442	6.547187707	4.3186563	5.432922028	5.852863847	7.153718042	4.552009652
79443	5.688622647	5.7155573	5.661687951	6.51496239	6.532588736	6.497336045
79447	-0.352108471	-0.352108	-0.352108471	5.760537073	5.775231583	5.745842563
79469	-0.352108471	-0.352108	-0.352108471	5.620762093	5.653299811	5.588224375
79567	9.096103812	6.0335312	7.564817513	5.953793665	7.495536378	6.724665022
79571	6.834956138	6.519674	6.204391777	-0.352108471	-0.352108471	-0.352108471
79572	5.971599196	5.8922408	6.050957599	-0.352108471	-0.352108471	-0.352108471
79573	1.383520155	-0.352108	3.119148782	5.284974356	5.315258837	5.254689875
79575	-0.352108471	-0.352108	-0.352108471	3.349712172	8.22981328	5.789762726
79581	6.242048279	5.0935678	7.390528797	8.888628446	5.575983727	2.263339007
79583	5.806407293	5.9583594	6.110311476	-0.352108471	-0.352108471	-0.352108471
79585	5.392061359	5.5539547	5.230167971	-0.352108471	-0.352108471	-0.352108471
79586	7.009263213	5.2174911	3.425719065	2.494267741	8.95270329	5.723485516
79587	-0.352108471	-0.352108	-0.352108471	6.056053999	7.790180286	4.321927712
79594	5.852937462	7.2197114	4.486163553	8.871018692	4.494626193	6.682822443
79595	6.110429784	5.690155	5.90029238	-0.352108471	-0.352108471	-0.352108471
79598	6.808890702	6.5237729	7.094008547	6.548106829	4.510515903	5.529311366
79600	5.227550975	5.6065246	5.985498285	1.631676003	3.615460478	-0.352108471
79602	-0.352108471	-0.352108	-0.352108471	6.463674915	6.479332994	6.448016836
79603	-0.352108471	-0.352108	-0.352108471	5.637296679	5.653299811	5.621293547
79608	8.989814916	6.4333187	3.876822539	3.522070642	6.430149827	0.613991457
79611	6.75496194	8.9222679	4.587655941	-0.352108471	-0.352108471	-0.352108471
79612	4.201099247	3.9195059	4.482692559	0.897715333	-0.352108471	2.147539136
79616	-0.352108471	-0.352108	-0.352108471	5.095423988	6.000882793	4.189965183
79622	1.501017585	-0.352108	3.354143641	4.027914765	6.266367194	8.504819622
79627	5.90029238	6.6652206	5.135364208	0.228373553	0.808855576	-0.352108471
79628	-0.352108471	-0.352108	-0.352108471	6.270222109	5.662217806	6.878226411
79631	-0.352108471	-0.352108	-0.352108471	5.637296679	5.653299811	5.621293547
79633	4.756742582	7.4248741	6.090808341	-0.352108471	-0.352108471	-0.352108471
79639	7.21054788	4.5164064	5.863477149	5.374787781	3.799408031	4.587097906
79641	-0.352108471	-0.352108	-0.352108471	6.28531312	3.459015953	9.111610286
79642	-0.352108471	-0.352108	-0.352108471	5.88700099	6.704755643	5.069246337

79644	6.370529939	5.3247267	5.847628321	3.50198806	1.678838435	-0.144311189
79647	3.289189542	8.0830638	5.686126657	6.45948883	7.425203426	8.390918022
79648	6.523931874	5.0522677	3.580603497	2.2153075	2.435312858	2.32531018
79649	4.167062861	2.6610869	3.41407487	5.855161109	9.538355061	2.171967156
79650	7.407823587	8.0681001	7.737961847	6.949901658	5.529073837	8.37072948
79651	1.501017585	-0.352108	3.354143641	2.039485684	-0.352108471	4.43107984
79652	6.77374078	6.7884205	6.759061079	-0.352108471	-0.352108471	-0.352108471
79654	-0.352108471	-0.352108	-0.352108471	7.152037191	7.168934463	7.135139919
79657	-0.352108471	-0.352108	-0.352108471	8.883919843	6.74407332	4.604226797
79658	-0.352108471	-0.352108	-0.352108471	5.7728658	4.80208491	3.831304021
79660	9.148118084	8.16864	7.189161965	3.855732012	9.259908425	6.557820219
79665	-0.352108471	-0.352108	-0.352108471	2.678682649	5.617887196	-0.260521897
79667	4.846956763	4.2931614	4.570059097	-0.352108471	-0.352108471	-0.352108471
79671	5.90029238	5.5501947	6.250390023	-0.352108471	-0.352108471	-0.352108471
79672	-0.352108471	-0.352108	-0.352108471	5.064224012	6.165870606	7.2675172
79676	-0.352108471	-0.352108	-0.352108471	6.766453815	6.773846551	6.759061079
79680	6.486805217	6.7775615	6.196048945	4.103893054	5.054280952	3.153505157
79684	5.625160771	5.6532998	5.597021732	-0.352108471	-0.352108471	-0.352108471
79689	2.351086241	1.0924157	3.609756788	1.043426334	0.440372779	1.646479888
79690	7.648357335	6.7408596	7.194608474	3.261261387	4.826956994	6.392652602
79691	8.252876448	6.6473961	7.45013629	3.894950463	7.394657313	5.644803888
79697	-0.352108471	-0.352108	-0.352108471	3.736322538	6.454525438	5.095423988
79699	7.874365488	6.7348555	7.30461047	6.627658487	7.684816315	7.156237401
79705	-0.352108471	-0.352108	-0.352108471	5.704335255	3.59914697	7.809523539
79707	9.231618302	8.5176617	9.945574885	7.591099561	9.180400365	8.385749963
79709	7.998904298	8.0797577	7.918050919	8.112109697	7.408368635	6.704627574
79710	1.383520155	-0.352108	3.119148782	5.325716078	5.336173319	5.315258837
79711	7.449622211	7.474041	7.425203426	7.178522138	7.188109812	7.168934463
79712	-0.352108471	-0.352108	-0.352108471	3.245625856	5.034370055	1.456881657
79714	2.351086241	2.3714773	2.330695134	-0.352108471	-0.352108471	-0.352108471
79717	3.474380252	4.3155755	5.156770811	7.713631986	4.9183863	6.316009143
79718	3.66960684	8.3711553	6.020381091	6.20188331	6.882573705	7.5632641
79723	6.615503319	4.4029421	5.509222715	1.223721097	1.305230684	1.14221151
79724	-0.352108471	-0.352108	-0.352108471	5.824288785	6.175746248	5.472831321
79727	6.595129197	5.975292	5.355454849	3.606446812	3.751349834	3.896252856
79728	6.182833209	5.4489798	4.715126332	-0.352108471	-0.352108471	-0.352108471
79741	6.82126504	5.9391922	5.057119403	-0.352108471	-0.352108471	-0.352108471
79742	6.525105542	7.7254983	5.324712747	3.874845854	6.545755645	9.216665437
79747	1.363577016	9.9514107	5.657493881	-0.352108471	-0.352108471	-0.352108471
79751	7.867153417	6.056054	4.244954581	8.242599605	5.823360095	10.66183912
79753	6.159099383	6.3831707	6.607242115	7.196070268	6.825760021	7.010915144
79755	-0.352108471	-0.352108	-0.352108471	6.271905109	7.095432661	7.918960214
79759	5.457139548	7.5059083	6.481523945	-0.352108471	-0.352108471	-0.352108471
79760	-0.352108471	-0.352108	-0.352108471	5.708801509	5.263851904	4.8189023
79763	5.550549618	4.5599556	6.541143656	9.700267411	4.558360156	7.129313784
79770	8.118478735	6.1852842	7.15188147	6.22018789	7.304856817	8.389525744
79776	-0.352108471	-0.352108	-0.352108471	8.511051832	3.367332611	5.939192221

79778	5.284974356	4.9587197	5.61122902	6.133512735	6.926938623	7.72036451
79782	4.691943082	5.2149592	4.953451118	-0.352108471	-0.352108471	-0.352108471
79784	6.688932906	4.801856	5.745394463	-0.352108471	-0.352108471	-0.352108471
79786	6.427990035	5.1841515	3.940312948	1.117721484	1.305230684	0.930212283
79792	9.175122757	5.980334	2.7855453	-0.352108471	-0.352108471	-0.352108471
79794	5.865107274	5.0242786	5.444692932	5.54524521	3.887455064	7.203035356
79796	6.497951189	7.0413321	6.769641641	3.622017489	4.832674249	6.043331009
79798	-0.352108471	-0.352108	-0.352108471	5.369332089	5.383503645	5.355160532
79800	6.030685393	7.1256856	6.578185475	4.097820191	6.157357479	8.216894767
79801	7.09781253	6.0717755	8.123849527	-0.352108471	-0.352108471	-0.352108471
79803	-0.352108471	-0.352108	-0.352108471	4.331576758	4.456886199	4.582195641
79809	5.463922025	5.0114031	5.916440999	2.259334908	4.249323288	3.254329098
79810	4.878665478	4.9140225	4.843308497	4.817531639	3.919505936	5.715557343
79812	3.993415015	3.4733142	3.733364623	6.254738707	5.840361047	5.425983388
79813	5.321063837	3.7486371	4.534850492	-0.352108471	-0.352108471	-0.352108471
79814	8.060414377	7.9748192	8.146009552	6.07919154	5.775231583	6.383151496
79817	2.351086241	5.054281	-0.352108471	4.178899946	4.245214285	4.112585606
79827	6.151585628	3.8856123	8.417558994	4.416483494	5.510999071	6.605514649
79829	3.666474075	5.7232059	4.694839979	4.271568891	3.470468384	2.669367877
79834	4.059425208	9.1967713	6.628098252	5.036590978	6.519972795	8.003354613
79837	-0.352108471	-0.352108	-0.352108471	7.269778778	3.882188675	5.575983727
79838	7.014830488	7.0441776	6.985483415	4.976042035	5.191222747	4.760861323
79840	5.392000621	5.5293114	5.254689875	0.044132154	-0.352108471	0.440372779
79845	4.231097311	5.2729017	3.189292972	0.624614178	0.440372779	0.808855576
79847	3.919505936	5.5054799	2.333531941	5.393523004	3.303298811	7.483747198
79850	6.056053999	6.6090268	5.503081194	8.848072569	1.409454702	5.128763636
79856	6.845389604	7.3666387	6.324140492	6.584605723	7.342869719	5.826341728
79862	6.749007119	7.2597847	7.770562357	4.675013723	5.706418	5.190715862
79866	5.150781781	4.3249473	5.976616275	-0.352108471	-0.352108471	-0.352108471
79867	4.981477599	4.5700591	4.158640595	0.228373553	0.808855576	-0.352108471
79868	-0.352108471	-0.352108	-0.352108471	4.04487627	6.12990004	8.21492381
79872	-0.352108471	-0.352108	-0.352108471	6.86293602	6.513185958	7.212686082
79874	3.978133825	5.6251608	7.272187717	-0.352108471	-0.352108471	-0.352108471
79875	-0.352108471	-0.352108	-0.352108471	6.187912724	6.110762977	6.149337851
79876	4.721636369	6.3958994	5.55876787	6.165870606	6.822797624	5.508943588
79877	-0.352108471	-0.352108	-0.352108471	8.00650318	7.15770761	6.30891204
79879	6.397175529	6.0736545	6.720696571	4.208083493	2.100935855	6.31523113
79882	6.882935672	6.2650023	6.573968999	6.677030402	5.042204336	5.859617369
79883	2.351086241	3.1415013	1.56067119	-0.352108471	-0.352108471	-0.352108471
79884	6.537383725	5.5664223	7.508345163	0.808855576	0.808855576	0.808855576
79885	-0.352108471	-0.352108	-0.352108471	6.804608153	5.07377629	5.939192221
79888	5.852937462	5.873346	5.832528937	-0.352108471	-0.352108471	-0.352108471
79890	-0.352108471	-0.352108	-0.352108471	4.624397093	5.704335255	6.784273416
79892	3.318185829	2.6505957	1.98300551	7.329088911	6.776532209	7.881645613
79893	-0.352108471	-0.352108	-0.352108471	3.833412591	2.686833264	4.979991919
79895	-0.352108471	-0.352108	-0.352108471	5.923493182	6.150741123	5.696245241
79898	-0.352108471	-0.352108	-0.352108471	5.44039385	6.699694083	6.070043966

79901	8.129211415	6.2738323	4.418453103	5.500077446	5.458255064	5.416432682
79902	-0.352108471	-0.352108	-0.352108471	5.511864271	3.999923266	7.023805276
79903	-0.352108471	-0.352108	-0.352108471	7.019487643	6.502079754	7.536895532
79905	5.268429393	5.3413269	5.414224389	6.269744616	5.92800466	5.586264704
79917	-0.352108471	-0.352108	-0.352108471	4.536671833	2.225499476	3.381085654
79921	3.805664353	5.018169	2.59315972	5.872307495	7.46119765	4.283417339
79923	5.974545641	8.5550666	7.264806117	6.896276597	6.034905799	7.757647395
79924	8.069039817	6.7699938	5.470947834	5.525602066	5.323975643	5.424788855
79929	6.288510867	7.9246294	4.652392332	-0.352108471	-0.352108471	-0.352108471
79931	4.478306873	8.5964606	6.537383725	-0.352108471	-0.352108471	-0.352108471
79932	6.857268256	6.8980976	6.938926904	8.275668882	5.808082345	7.041875613
79933	6.995223439	7.3267668	7.658310125	7.278477476	7.218779379	7.338175573
79939	6.440349559	6.8325729	7.224796305	7.308855623	7.376708576	7.241002671
79940	2.732117059	5.7142657	8.696414335	0.975533543	0.808855576	1.14221151
79943	6.941311974	6.6718746	6.402437303	2.024343053	2.051108653	1.997577452
79944	11.46433596	12.341692	11.90301375	10.24233349	9.25870823	11.22595875
79954	5.956452551	6.056054	6.155655447	6.122040588	6.022050925	5.922061262
79962	5.742452228	5.2483042	4.75415608	4.201099247	3.400188256	5.002010239
79966	7.2574548	8.1107521	6.404157471	6.541302808	6.77736617	7.013429532
79968	5.659195707	3.8526025	7.465788864	6.13283526	5.169252326	7.096418193
79971	-0.352108471	-0.352108	-0.352108471	6.369156591	4.483354272	8.254958909
79974	-0.352108471	-0.352108	-0.352108471	1.831525138	2.585128717	3.338732297
79977	7.17777694	6.2543398	8.101214072	-0.352108471	-0.352108471	-0.352108471
79979	7.13178117	6.9946748	6.857568352	4.371868981	6.923287786	5.647578383
79987	-0.352108471	-0.352108	-0.352108471	2.397492216	-0.352108471	5.147092904
79989	7.496068808	6.8354301	6.174791302	6.673694267	6.492891233	6.312088198
79990	-0.352108471	-0.352108	-0.352108471	6.186562848	5.709591052	6.663534645
79993	6.049511629	5.2216669	4.393822196	4.373002436	6.066266721	2.679738151
79998	-0.352108471	-0.352108	-0.352108471	4.13623093	11.64596747	7.8910992
80005	-0.352108471	-0.352108	-0.352108471	6.466140449	6.204656258	5.943172067
80008	6.437194531	5.0826197	3.728044812	0.939116071	1.437859363	0.440372779
80011	6.573968999	9.43788	3.710057951	-0.352108471	-0.352108471	-0.352108471
80012	8.525375615	10.552734	6.498017297	7.338548995	7.827114021	6.84998397
80013	-0.352108471	-0.352108	-0.352108471	4.66005769	4.544219328	4.775896051
80014	3.513288917	7.8639564	5.688622647	4.28305004	5.095423988	5.907797936
80019	6.748789459	6.1215288	5.494268079	5.317943336	7.09136918	6.204656258
80022	-0.352108471	-0.352108	-0.352108471	2.146123334	2.191310813	2.100935855
80023	6.768395908	5.8529375	4.937479015	6.642214975	6.23567117	5.829127366
80028	8.334697496	7.5720419	9.097353082	7.191434148	8.325203573	7.75831886
80032	5.452723741	4.574195	6.33125245	3.538105901	-0.581711188	1.478197356
80035	6.305519736	6.2558778	6.355161685	5.237204548	5.494417177	4.979991919
80039	5.035222837	5.2218394	4.848606265	2.460720591	4.790473589	0.130967593
80055	-0.352108471	-0.352108	-0.352108471	0.897715333	-0.352108471	2.147539136
80069	6.140087083	4.955018	7.325156207	1.371545024	1.437859363	1.305230684
80071	-0.352108471	-0.352108	-0.352108471	4.179667512	5.529311366	6.87895522
80095	-0.352108471	-0.352108	-0.352108471	2.139844176	-0.352108471	4.631796822
80097	-0.352108471	-0.352108	-0.352108471	8.982924358	7.605055129	6.2271859

80099	-0.352108471	-0.352108	-0.352108471	4.067191766	2.481575183	0.8959586
80111	-0.352108471	-0.352108	-0.352108471	5.809683002	5.821410838	5.833138673
80115	3.246640567	3.1563093	3.336971836	-0.352108471	-0.352108471	-0.352108471
80131	6.387314214	3.5067002	4.947007189	0.323567339	6.173804703	3.248686021
80139	7.458282885	7.474041	7.442524773	7.792722561	7.822192666	7.763252455
80142	-0.352108471	-0.352108	-0.352108471	5.120327044	6.64789689	8.175466735
80143	8.283240037	5.5207185	6.901979267	2.960272696	2.169424975	1.378577254
80144	5.428393874	5.3441924	5.512595355	-0.352108471	-0.352108471	-0.352108471
80145	7.137727412	6.0373568	8.238098054	-0.352108471	-0.352108471	-0.352108471
80146	6.396868383	6.3057999	6.48793683	-0.352108471	-0.352108471	-0.352108471
80148	6.450302837	4.0443467	8.856258988	-0.352108471	-0.352108471	-0.352108471
80149	5.55876787	3.5973355	7.520200199	-0.352108471	-0.352108471	-0.352108471
80150	-0.352108471	-0.352108	-0.352108471	4.50955308	4.604936301	4.557244691
80152	-0.352108471	-0.352108	-0.352108471	6.020381091	5.903450087	6.137312096
80161	6.083216808	5.9649007	6.201532907	-0.352108471	-0.352108471	-0.352108471
80162	-0.352108471	-0.352108	-0.352108471	7.794659744	5.821410838	3.848161932
80168	5.341183412	5.2849744	5.2287653	-0.352108471	-0.352108471	-0.352108471
80169	1.817698148	1.3835202	0.949342163	5.982003862	4.822573544	7.141434179
80173	7.582976966	5.3546856	3.126394332	5.750895627	4.459483409	5.105189518
80176	-0.352108471	-0.352108	-0.352108471	7.232000306	7.241236027	7.222764584
80196	4.954109947	5.7494021	6.544694281	-0.352108471	-0.352108471	-0.352108471
80198	3.153505157	3.3377466	3.245625856	-0.352108471	-0.352108471	-0.352108471
80199	-0.352108471	-0.352108	-0.352108471	5.101230542	5.001788697	4.902346851
80204	5.344627021	3.6023395	7.086914527	2.70315649	2.357098027	2.011039563
80207	5.225205553	6.2330281	4.217383012	-0.352108471	-0.352108471	-0.352108471
80210	7.331818668	8.0932309	7.712524769	7.273823363	7.300708227	7.246938498
80212	5.980334028	6.0023087	5.958359385	6.087644454	6.101634422	6.073654487
80213	-0.352108471	-0.352108	-0.352108471	3.501347858	6.689500118	5.095423988
80216	1.501017585	-0.352108	3.354143641	3.919505936	3.919505936	3.919505936
80217	4.329518547	5.7644547	5.046986617	-0.352108471	-0.352108471	-0.352108471
80218	5.017136436	6.3141882	3.720084686	6.056053999	4.376227265	7.735880733
80219	5.451868246	6.9076477	8.363427245	6.008180529	7.084947944	8.161715359
80222	6.217176725	6.5524721	6.887767429	3.968083022	8.737567406	6.352825214
80223	6.642574621	8.3147025	7.478638566	4.282405722	4.826488843	5.370571964
80224	9.006623652	5.9666155	7.486619561	5.450612119	6.561463791	7.672315462
80227	5.939192221	6.1237857	5.754598785	-0.352108471	-0.352108471	-0.352108471
80228	9.475093445	9.4702768	9.479910088	8.630178311	8.531441672	8.72891495
80232	6.991085456	6.083682	6.537383725	1.839745951	4.456886199	7.074026448
80233	-0.352108471	-0.352108	-0.352108471	1.551002809	3.384482817	5.217962826
80256	-0.352108471	-0.352108	-0.352108471	6.264160615	5.455074122	5.859617369
80258	-0.352108471	-0.352108	-0.352108471	4.234381997	5.315258837	3.153505157
80263	5.758523395	4.5321109	6.984935849	2.973271304	3.519327359	4.065383413
80264	6.954346295	7.3154815	6.593211042	3.682181533	4.245214285	3.119148782
80270	6.414258136	5.7945951	7.033921129	0.598807818	1.549724107	-0.352108471
80273	3.054232215	4.5503078	6.046383388	5.125131444	6.784412458	8.443693473
80274	-0.352108471	-0.352108	-0.352108471	3.389667979	2.147539136	4.631796822
80279	-0.352108471	-0.352108	-0.352108471	6.266367194	9.854579127	2.67815526

80301	-0.352108471	-0.352108	-0.352108471	5.694437461	6.872782744	8.051128027
80305	6.432653401	4.6155835	2.798513626	5.095423988	6.816761615	3.374086361
80306	-0.352108471	-0.352108	-0.352108471	5.026688316	4.148619868	5.904756765
80307	6.458623164	5.7838824	5.109141713	7.152873735	0.072605572	3.612739653
80311	7.07131791	5.5356046	6.303461245	-0.352108471	-0.352108471	-0.352108471
80312	7.560829242	5.4093158	6.485072534	-0.352108471	-0.352108471	-0.352108471
80317	9.820234852	9.8153882	9.825081469	8.366100887	8.413151789	8.319049985
80321	5.343591945	6.502263	7.660934112	-0.352108471	-0.352108471	-0.352108471
80324	-0.352108471	-0.352108	-0.352108471	6.79867074	6.689878453	6.907463027
80325	-0.352108471	-0.352108	-0.352108471	6.729377134	5.983736712	5.23809629
80331	5.463922025	7.6193778	3.308466291	8.475881183	7.234851541	5.9938219
80333	-0.352108471	-0.352108	-0.352108471	7.002741396	6.204656258	5.40657112
80335	6.349191313	6.2424418	6.455940863	9.230048536	5.557121845	7.39358519
80339	4.019632967	6.6433219	5.331477429	2.678098132	3.99590524	3.337001686
80344	1.383520155	-0.352108	3.119148782	1.374300821	1.939746065	0.808855576
80346	-0.352108471	-0.352108	-0.352108471	5.325716078	5.336173319	5.315258837
80351	-0.352108471	-0.352108	-0.352108471	6.554659539	6.165870606	5.777081673
80381	7.402710261	7.4288784	7.415794329	7.855473441	10.42750473	5.283442148
80700	-0.352108471	-0.352108	-0.352108471	7.242521962	5.039883593	9.445160332
80704	5.079283667	5.3152588	4.843308497	1.530736894	0.808855576	2.252618212
80709	6.072281416	5.1234227	5.597852034	4.121930465	-0.952331984	1.58479924
80712	4.014810134	6.573969	9.133127865	-0.352108471	-0.352108471	-0.352108471
80724	-0.352108471	-0.352108	-0.352108471	6.029083441	5.550442011	5.789762726
80727	6.272113412	7.4218008	6.846957097	8.11139689	8.824423311	7.39837047
80731	8.611833514	3.0940414	5.852937462	-0.352108471	-0.352108471	-0.352108471
80736	6.413550728	5.3564725	4.299394212	1.227667732	0.808855576	1.646479888
80741	4.197102645	5.6167433	7.036384002	-0.352108471	-0.352108471	-0.352108471
80742	5.8261513	6.3691566	6.912161881	-0.352108471	-0.352108471	-0.352108471
80757	6.396868383	7.2213223	5.572414434	-0.352108471	-0.352108471	-0.352108471
80758	-0.352108471	-0.352108	-0.352108471	5.017136436	5.054280952	4.979991919
80759	4.91726567	7.7476677	6.332466669	2.039485684	-0.352108471	4.43107984
80760	7.825105859	7.2592139	8.390997824	6.029076362	4.651904177	3.274731991
80761	7.48354716	5.3564725	3.22939778	1.789517331	1.588701675	1.689109503
80762	7.925631762	6.7357797	5.54592761	2.65949763	2.211943219	1.764388808
80772	7.289777346	7.244076	7.335478698	4.08236011	4.245214285	3.919505936
80776	-0.352108471	-0.352108	-0.352108471	4.724955416	6.149337851	7.573720285
80777	7.601933332	7.5785512	7.625315436	6.022050925	6.526286131	5.517815719
80778	6.173033727	10.319567	8.246300486	7.248883109	7.381678228	7.315280669
80781	1.392109249	3.136327	-0.352108471	5.637296679	5.653299811	5.621293547
80789	9.671328132	3.2220535	6.446690805	-0.352108471	-0.352108471	-0.352108471
80790	-0.352108471	-0.352108	-0.352108471	5.871599621	3.042172777	4.456886199
80821	7.531970087	5.5207125	3.50945491	4.950315419	5.005105417	4.977710418
80829	7.217317616	7.2305473	7.204087928	-0.352108471	-0.352108471	-0.352108471
80830	3.669750275	1.8514872	2.760618758	6.386519472	7.576425126	5.196613819
80832	9.544510253	9.2432265	8.941942751	5.50311456	11.52900119	8.516057875
80851	5.900703788	3.8056644	1.710624918	6.613428537	2.387605953	10.83925112
80852	-0.352108471	-0.352108	-0.352108471	2.802945389	2.051108653	3.554782125

80856	3.763205746	5.8984915	8.033777209	3.919505936	2.790821184	5.048190687
80862	-0.352108471	-0.352108	-0.352108471	5.707365561	6.764476761	6.235921161
81029	6.893765651	7.0898244	6.991795048	0.14959489	2.064739793	3.979884696
81031	-0.352108471	-0.352108	-0.352108471	5.060879346	10.22122578	7.641052565
81033	2.065292044	-0.352108	4.482692559	1.804300246	1.731739118	1.876861374
81034	6.791156986	6.9092955	6.850226219	6.225937921	5.228208078	7.223667764
81037	3.156147497	3.5151412	3.874134975	5.094707026	6.682822443	8.270937859
81050	-0.352108471	-0.352108	-0.352108471	5.723485516	5.731413688	5.715557343
81285	4.331087074	4.3347012	4.338315403	0.685292531	0.440372779	0.930212283
81343	6.121528769	6.1414231	6.101634422	-0.352108471	-0.352108471	-0.352108471
81490	4.910921778	8.7228883	6.816905042	-0.352108471	-0.352108471	-0.352108471
81493	5.559971507	4.0364682	7.083474795	4.920275323	5.816563325	5.368419324
81502	5.935502203	6.056054	6.176605795	7.267174057	7.932090689	7.599632373
81532	-0.352108471	-0.352108	-0.352108471	5.412561087	4.711054194	6.114067981
81537	7.820641569	5.0459959	6.433318728	-0.352108471	-0.352108471	-0.352108471
81539	8.240762624	8.2416329	8.239892398	7.617156715	1.968135913	4.792646314
81542	6.63987902	5.4481128	6.043995933	7.465370061	5.082850362	9.84788976
81543	6.753841062	6.4251279	6.096414808	3.560919742	-1.446833482	1.05704313
81544	-0.352108471	-0.352108	-0.352108471	6.050502124	4.812540974	7.288463274
81550	8.096039735	5.4514418	6.77374078	-0.352108471	-0.352108471	-0.352108471
81552	5.939192221	7.2736407	4.604743704	7.640972487	7.130842071	6.620711656
81555	7.93299766	1.6398081	4.786402873	2.473055456	4.532271743	3.5026636
81556	7.480178217	6.7052875	5.930396807	-0.352108471	-0.352108471	-0.352108471
81563	2.168802966	5.0171364	7.865469905	-0.352108471	-0.352108471	-0.352108471
81565	5.349194799	5.5587679	5.768340942	8.045831352	3.720936301	5.883383827
81566	6.705287512	6.7206966	6.689878453	1.873849414	1.939746065	1.807952763
81569	4.048231905	1.2529594	2.65059567	-0.352108471	-0.352108471	-0.352108471
81570	8.169438337	4.6971991	6.433318728	-0.352108471	-0.352108471	-0.352108471
81571	5.17910263	6.3691566	7.559210551	-0.352108471	-0.352108471	-0.352108471
81575	7.524733284	7.0128211	8.036645486	6.454261972	6.887913509	6.020610435
81576	5.546246248	3.839732	4.692989119	4.983644395	5.845499835	6.707355275
81577	-0.352108471	-0.352108	-0.352108471	3.585121301	4.316315393	2.85392721
81603	5.000596186	5.6574939	6.314391576	5.977010385	6.6560422	7.335074016
81605	5.688622647	3.6926662	7.684579142	3.11131689	5.958359385	8.805401879
81606	6.332466669	6.2248677	6.44006562	7.37525479	8.754053886	10.13285298
81608	6.095923221	6.6400194	7.184115564	7.478462511	9.471973166	5.484951856
81609	8.41687693	6.0017252	3.586573424	6.528042901	3.952594664	9.103491137
81611	7.66556257	6.1937542	4.721945766	5.980334028	7.460936982	4.499731074
81614	5.441019591	6.9269084	6.183964016	3.322276022	8.789831976	6.056053999
81618	4.852471579	6.1350949	5.493783215	6.369156591	4.649101157	8.089212024
81619	4.98284752	5.4775912	5.972334837	5.95428886	6.041492036	5.867085684
81620	7.141316727	7.7751504	7.458233568	6.131354478	5.724668212	5.317981946
81624	6.428655029	6.7737408	7.11882653	-0.352108471	-0.352108471	-0.352108471
81628	4.960006502	6.573969	8.187931497	5.578083691	5.369332089	5.160580486
81631	-0.352108471	-0.352108	-0.352108471	8.078573586	11.11468453	5.042462646
81669	-0.352108471	-0.352108	-0.352108471	5.551463511	5.627005534	5.702547557
81671	3.805664353	3.5661822	4.045146534	7.263979321	7.013360586	6.762741852

81688	4.169764452	5.6886226	7.207480842	0.727922146	1.807952763	-0.352108471
81689	5.981703702	6.1045589	6.227414164	-0.352108471	-0.352108471	-0.352108471
81786	-0.352108471	-0.352108	-0.352108471	7.093911607	5.47719731	6.285554459
81788	8.304448046	8.8493567	8.576902394	7.402947837	7.399343256	7.395738674
81790	8.439221476	4.8021397	6.620680601	6.170030093	8.853589904	3.486470282
81792	6.369156591	7.6149171	5.12339607	7.133757538	5.458330193	6.296043865
81793	4.303637301	7.5778813	5.940759291	5.975693713	7.299166056	6.637429884
81796	5.552256259	5.9391922	6.326128184	-0.352108471	-0.352108471	-0.352108471
81831	-0.352108471	-0.352108	-0.352108471	5.679126224	6.596757815	7.514389407
81833	-0.352108471	-0.352108	-0.352108471	6.345275458	6.355161685	6.33538923
81839	-0.352108471	-0.352108	-0.352108471	6.609543024	6.63062078	6.588465267
81844	4.469588097	6.5047179	5.487153005	3.301549522	7.165585325	5.233567424
81846	6.609632567	2.9273409	4.768486756	0.685292531	0.440372779	0.930212283
81854	5.327034875	2.9166574	4.121846117	3.40194973	1.394637337	5.409262124
81855	1.596610088	3.6368248	5.677039489	7.330399645	5.698786261	6.514592953
81857	4.878665478	4.4509656	5.306365389	7.695698749	5.369185218	6.532441983
81858	-0.352108471	-0.352108	-0.352108471	6.569711703	5.876952844	5.184193986
81873	6.205676296	8.4050067	7.305341479	7.460593074	6.225677256	6.843135165
81875	-0.352108471	-0.352108	-0.352108471	5.713389324	6.621168045	7.528946767
81876	5.284974356	6.3863441	4.183604637	6.407504925	7.965839464	9.524174003
81887	4.817531639	3.1479137	6.487149554	6.01226783	5.88700099	5.761734149
81888	3.533617112	5.1841515	6.834685871	5.830338531	7.842089107	6.836213819
81890	5.089412202	4.284452	5.894372392	-0.352108471	-0.352108471	-0.352108471
81892	4.025174714	3.136327	4.914022459	6.204656258	6.215464554	6.193847962
81926	-0.352108471	-0.352108	-0.352108471	7.261502113	7.129581018	7.195541565
81929	4.85156964	5.9575452	7.063520775	-0.352108471	-0.352108471	-0.352108471
81930	2.705934611	6.6895379	10.67314126	3.59425584	3.575986763	3.585121301
81931	-0.352108471	-0.352108	-0.352108471	7.126019756	4.178899946	1.231780135
83259	10.78863045	8.6646778	9.726654142	7.335317928	8.910309521	8.122813725
83394	3.527916452	3.136327	3.919505936	2.051108653	2.051108653	2.051108653
83439	5.775846281	5.6522715	5.528696668	1.683074947	5.095423988	8.507773029
83440	5.824288785	5.9190303	5.7295473	-0.352108471	-0.352108471	-0.352108471
83442	10.13296842	9.526807	9.82988773	10.63675203	9.507510392	10.07213121
83445	9.691724195	8.1575603	6.623396417	7.444268716	6.12576271	6.785015713
83451	4.53675364	7.7273456	6.132049638	5.574991057	6.75763359	7.940276124
83452	9.0329577	5.2649508	7.148954265	6.66302406	6.564854867	6.761193252
83460	-0.352108471	-0.352108	-0.352108471	3.515457114	3.44626434	3.377071566
83461	5.085883833	6.1494001	5.617641948	6.963850182	6.255235865	6.609543024
83464	7.208169218	6.8768769	7.042523083	-0.352108471	-0.352108471	-0.352108471
83468	-0.352108471	-0.352108	-0.352108471	7.724438171	4.57423753	6.149337851
83541	-0.352108471	-0.352108	-0.352108471	3.245625856	3.337746555	3.153505157
83544	9.602240643	6.1588	7.880520335	7.656950668	5.614332304	9.699569031
83547	3.497339483	5.3910182	7.284696846	-0.352108471	-0.352108471	-0.352108471
83549	7.114527862	5.0670888	6.090808341	-0.352108471	-0.352108471	-0.352108471
83590	6.734743009	6.3968684	6.058993756	6.251749538	7.269863612	8.287977686
83595	6.703944702	5.5062399	4.30853501	4.029859974	1.835742592	-0.358374791
83596	3.092217044	8.7906513	5.941434187	-0.352108471	-0.352108471	-0.352108471

83604	8.110337885	5.7166399	6.913488898	3.886210183	8.081263241	5.983736712
83605	3.127737875	2.2629017	3.992574055	2.641611363	3.585121301	4.52863124
83606	5.980334028	4.6291393	7.331528728	1.345967809	1.14221151	1.549724107
83636	4.682964349	7.1926748	2.17325392	6.763616776	5.064465535	8.462768017
83637	6.212536209	5.7113495	6.713722927	6.56915909	6.33202207	6.806296109
83638	3.599161659	1.7104178	2.65478974	6.541009048	5.132500634	7.949517461
83641	-0.352108471	-0.352108	-0.352108471	5.034557703	3.711447169	4.373002436
83642	-0.352108471	-0.352108	-0.352108471	6.840114058	6.041234306	7.638993811
83648	5.463922025	5.5293114	5.398532684	-0.352108471	-0.352108471	-0.352108471
83658	5.284491263	6.2642494	5.774370319	8.851320138	6.949901658	5.048483179
83660	-0.352108471	-0.352108	-0.352108471	6.061426254	5.325716078	4.590005902
83667	-0.352108471	-0.352108	-0.352108471	3.245625856	3.075216659	3.416035053
83692	2.456043316	7.6525186	5.054280952	5.354343885	5.745394463	6.136445041
83694	2.65059567	3.2783197	2.022871607	7.591869696	5.112024927	6.351947311
83695	-0.352108471	-0.352108	-0.352108471	1.79770586	5.254651642	8.711597425
83696	-0.352108471	-0.352108	-0.352108471	3.194371043	6.409798778	4.80208491
83698	6.71245091	5.5778117	6.145131319	0.791292145	0.440372779	1.14221151
83700	2.55641115	6.3968684	10.23732561	5.980334028	6.551763288	5.408904769
83714	4.544261391	4.2452143	4.843308497	1.530736894	2.252618212	0.808855576
83715	-0.352108471	-0.352108	-0.352108471	1.957012451	3.245625856	4.534239261
83716	4.913028459	4.0160458	3.119063082	5.852863847	3.550773176	8.154954519
83719	7.309713553	6.8469571	6.384200641	-0.352108471	-0.352108471	-0.352108471
83733	6.172221787	5.7494021	5.326582442	-0.352108471	-0.352108471	-0.352108471
83742	7.204945658	7.6225236	8.0401015	3.886134936	9.169678294	6.527906615
83743	6.038513836	5.1289621	4.219410306	0.476561106	-0.352108471	1.305230684
83746	6.705287512	6.9657259	6.444849091	-0.352108471	-0.352108471	-0.352108471
83752	1.383520155	-0.352108	3.119148782	8.906793545	4.686836214	6.796814879
83759	-0.352108471	-0.352108	-0.352108471	8.849389674	5.723485516	2.597581357
83786	4.788579763	5.0396142	4.537545298	7.680837115	6.324316173	9.037358056
83787	6.888450633	7.1095361	6.667365196	5.471692453	4.93312003	6.010264877
83851	6.755500212	5.1562993	5.955899749	-0.352108471	-0.352108471	-0.352108471
83852	-0.352108471	-0.352108	-0.352108471	5.789762726	5.804293869	5.775231583
83853	4.59024713	5.5979093	6.605571409	1.929530708	2.051108653	1.807952763
83857	6.405937537	5.6407828	6.023360165	4.970473812	5.529886425	4.411061198
83860	-0.352108471	-0.352108	-0.352108471	5.263851904	6.356508181	4.171195628
83867	5.774370319	5.825168	5.723572646	-0.352108471	-0.352108471	-0.352108471
83871	2.065292044	-0.352108	4.482692559	6.558608437	6.999484347	7.440360257
83873	-0.352108471	-0.352108	-0.352108471	2.221964512	2.252618212	2.191310813
83874	-0.352108471	-0.352108	-0.352108471	6.696345531	4.042318646	5.369332089
83877	4.018429536	4.7729096	5.527389745	0.598807818	-0.352108471	1.549724107
83878	6.739745787	3.6952365	5.217491139	-0.352108471	-0.352108471	-0.352108471
83881	3.326996042	5.6173207	4.472158387	0.727922146	1.807952763	-0.352108471
83882	-0.352108471	-0.352108	-0.352108471	5.300418005	4.89042997	5.095423988
83890	2.711561556	3.5963386	1.826784469	-0.352108471	-0.352108471	-0.352108471
83891	6.461093679	3.9738886	5.217491139	-0.352108471	-0.352108471	-0.352108471
83895	5.90029238	7.8826221	3.917962638	5.213645062	8.825330223	7.019487643
83931	5.980334028	6.0023087	5.958359385	6.866733042	6.880482436	6.852983648

83933	-0.352108471	-0.352108	-0.352108471	5.949545308	7.770557489	4.128533127
83937	-0.352108471	-0.352108	-0.352108471	5.745394463	5.775231583	5.715557343
83939	-0.352108471	-0.352108	-0.352108471	5.575983727	0.638891285	10.51307617
83982	5.085607333	6.9110537	3.260160947	5.529311366	5.529311366	5.529311366
83985	4.222001954	5.4937832	6.765564475	6.161839974	2.433438191	4.297639083
83990	8.579287539	10.059679	11.54007048	7.61604139	8.877367657	10.13869392
84033	5.289905506	6.5373837	7.784861943	3.572063041	5.336173319	1.807952763
84034	7.960102136	8.0006237	7.91958059	6.380541917	6.056053999	5.731566081
84056	4.434961537	7.2324736	10.02998575	7.723555937	6.071484426	6.897520181
84058	-0.352108471	-0.352108	-0.352108471	6.204656258	6.700552881	5.708759635
84060	5.138735922	5.0522677	4.965799449	5.622844465	6.702534557	6.162689511
84061	7.594893599	8.7799869	8.187440272	3.606517346	4.491365131	2.721669562
84064	5.216665947	6.9984117	6.107538801	-0.352108471	-0.352108471	-0.352108471
84065	5.581135637	6.6073775	7.633619281	5.488813385	4.80208491	4.115356435
84066	-0.352108471	-0.352108	-0.352108471	1.688802714	1.939746065	1.437859363
84067	-0.352108471	-0.352108	-0.352108471	5.811468301	4.716235508	5.263851904
84069	4.882035507	5.5529468	5.217491139	-0.352108471	-0.352108471	-0.352108471
84075	6.020381091	6.7794102	5.26135198	-0.352108471	-0.352108471	-0.352108471
84079	6.913488898	7.4964795	6.330498273	-0.352108471	-0.352108471	-0.352108471
84080	-0.352108471	-0.352108	-0.352108471	6.115657815	6.073654487	6.157661143
84081	-0.352108471	-0.352108	-0.352108471	6.61077438	6.31657545	6.463674915
84085	4.928333285	5.0399701	4.984151706	5.596301458	6.177700521	5.88700099
84102	5.530278441	5.9413637	5.735821067	4.904478682	4.245214285	5.563743079
84105	3.336346647	5.0894122	6.842477757	3.749732623	6.342128298	5.045930461
84109	5.49470711	7.310019	6.402363066	0.044132154	0.440372779	-0.352108471
84124	5.256268008	5.4582551	5.054280952	4.583433279	5.054280952	4.112585606
84128	6.033946416	5.4283939	4.822841332	2.80652814	1.776802743	3.836253538
84133	4.930470149	8.6739805	6.802225342	5.850983312	5.941434187	6.031885062
84141	-0.352108471	-0.352108	-0.352108471	6.165870606	3.40664166	8.925099552
84142	-0.352108471	-0.352108	-0.352108471	4.926329825	4.484531333	5.368128318
84144	5.187223787	5.7049355	6.222647281	1.437859363	1.437859363	1.437859363
84148	5.477780901	6.2420483	7.006315657	-0.352108471	-0.352108471	-0.352108471
84153	-0.352108471	-0.352108	-0.352108471	6.77374078	6.788420481	6.759061079
84159	3.127737875	3.136327	3.119148782	7.238918097	7.135139919	7.342696275
84162	5.828848464	5.825168	5.832528937	6.802382176	7.016299086	6.588465267
84163	2.423475267	3.5151412	4.606807204	0.897715333	-0.352108471	2.147539136
84164	-0.352108471	-0.352108	-0.352108471	3.959314364	8.339361337	6.149337851
84166	6.396868383	6.4105853	6.383151496	4.66005769	4.688318557	4.631796822
84168	2.65059567	4.8561664	0.445024936	-0.352108471	-0.352108471	-0.352108471
84171	-0.352108471	-0.352108	-0.352108471	6.566033225	7.607490017	5.524576432
84179	-0.352108471	-0.352108	-0.352108471	7.800182135	4.774498542	1.748814948
84193	-0.352108471	-0.352108	-0.352108471	5.760537073	5.290807752	6.230266394
84196	5.975693575	5.5231107	5.749402114	6.377541456	4.611292898	5.494417177
84197	-0.352108471	-0.352108	-0.352108471	4.456886199	4.004094343	4.909678056
84216	9.035849875	6.7357797	4.435709497	2.301055037	4.954218546	-0.352108471
84224	-0.352108471	-0.352108	-0.352108471	4.595011698	3.736344232	2.877676766
84231	10.23880216	6.7545585	3.270314896	7.523606591	7.555244212	7.49196897

84232	6.954675705	4.0058671	9.903484351	6.739974181	6.287974154	7.191974209
84243	-0.352108471	-0.352108	-0.352108471	7.286324647	7.619331937	6.953317357
84245	7.309459055	6.693678	7.001568536	7.278740375	6.30917905	8.2483017
84246	-0.352108471	-0.352108	-0.352108471	2.686833264	3.712925324	1.660741203
84247	-0.352108471	-0.352108	-0.352108471	6.056053999	6.073654487	6.038453511
84255	2.351086241	2.5193947	2.182777795	-0.352108471	-0.352108471	-0.352108471
84259	5.625160771	4.0277507	7.222570869	0.044132154	-0.352108471	0.440372779
84262	6.433318728	6.4453648	6.421272681	5.637296679	5.653299811	5.621293547
84263	-0.352108471	-0.352108	-0.352108471	6.056053999	4.835243577	7.27686442
84264	-0.352108471	-0.352108	-0.352108471	3.245625856	2.167076618	4.324175094
84266	-0.352108471	-0.352108	-0.352108471	5.821410838	6.47308539	5.169736286
84267	7.586996411	1.5722403	4.579618372	6.469055374	5.920555088	7.01755566
84268	2.211071469	7.8789804	5.045025943	-0.352108471	-0.352108471	-0.352108471
84269	2.407155079	2.5232121	2.639269134	1.90830372	1.939746065	1.876861374
84271	4.501929911	3.8257343	5.178125502	6.736734301	8.13743452	7.43708441
84274	-0.352108471	-0.352108	-0.352108471	6.204656258	6.215464554	6.193847962
84276	5.346492148	7.9679554	6.657223782	5.827895643	4.397545314	7.258245971
84277	5.267512683	5.8278956	6.388278602	5.010261535	3.959090419	6.061432651
84282	-0.352108471	-0.352108	-0.352108471	3.417899765	6.748763753	5.083331759
84285	1.328794835	1.6698571	2.010919464	3.589288335	3.81023428	3.368342391
84286	7.466207166	10.507513	4.4249015	2.168105043	4.688318557	-0.352108471
84287	-0.352108471	-0.352108	-0.352108471	5.496431992	6.434644758	7.372857524
84293	7.352453156	7.9639646	6.740941743	3.801316843	2.073301066	0.345285289
84294	6.692705039	4.4052829	8.980127196	9.47737886	6.01555717	7.746468015
84296	4.889291061	5.8113861	5.350338576	1.525874689	3.980802614	2.753338652
84298	1.383520155	-0.352108	3.119148782	6.824070034	5.605887822	6.214978928
84299	5.657493881	4.8900866	6.424901157	5.584676343	6.01100587	5.158346816
84300	5.980334028	8.5047567	3.455911335	6.250828564	4.216306283	5.233567424
84304	6.977258204	5.7271151	8.227401282	-0.352108471	-0.352108471	-0.352108471
84310	6.766594494	7.1121207	7.457646996	6.35674501	4.970729633	5.663737322
84312	-0.352108471	-0.352108	-0.352108471	6.505960865	5.246204134	7.765717596
84316	3.127737875	1.3772614	4.878214363	6.386836766	5.021833744	5.704335255
84317	-0.352108471	-0.352108	-0.352108471	10.04224865	-0.007975782	5.017136436
84320	-0.352108471	-0.352108	-0.352108471	7.005019726	6.77374078	6.542461834
84324	5.90029238	5.9519035	5.848681221	-0.352108471	-0.352108471	-0.352108471
84326	-0.352108471	-0.352108	-0.352108471	5.946488185	6.74406273	6.345275458
84327	-0.352108471	-0.352108	-0.352108471	5.923493182	5.926961306	5.920025058
84328	5.701758731	6.2048047	5.198712737	5.32911612	3.840699343	6.817532897
84330	-0.352108471	-0.352108	-0.352108471	4.80208491	4.843308497	4.760861323
84331	6.481523945	6.3927328	6.570315073	-0.352108471	-0.352108471	-0.352108471
84332	5.456407505	5.3835036	5.529311366	-0.352108471	-0.352108471	-0.352108471
84334	3.519697412	5.2527512	4.386224296	5.265354555	4.209750764	4.73755266
84335	5.979176954	8.168924	10.35867098	6.963991695	6.224682193	7.703301197
84336	5.718612439	5.6251608	5.531709103	-0.352108471	-0.352108471	-0.352108471
84337	7.442524773	7.4425248	7.442524773	7.528959936	7.55064888	7.507270991
84340	6.40229251	5.2006733	5.801482926	8.959547949	4.58793361	6.77374078
84343	7.857598295	4.8807149	6.369156591	-0.352108471	-0.352108471	-0.352108471

84364	5.90029238	6.8032541	4.99733063	6.44739251	7.077316842	6.762354676
84365	6.212536209	6.1916703	6.233402072	-0.352108471	-0.352108471	-0.352108471
84376	8.502900368	8.4166969	8.589103829	7.607035011	7.615258575	7.598811448
84418	6.288510867	6.3152311	6.261790604	-0.352108471	-0.352108471	-0.352108471
84436	5.8993644	5.4394504	5.669407383	1.727216326	2.506545095	0.947887556
84440	6.419814424	4.9346649	5.677239679	4.927679215	6.145324408	3.710034022
84444	6.207810414	5.7743703	5.340930224	5.575983727	3.966551194	7.185416259
84445	3.797822384	3.6761388	3.919505936	7.905601839	7.935107432	7.876096247
84446	-0.352108471	-0.352108	-0.352108471	5.621162875	4.780619904	5.20089139
84447	-0.352108471	-0.352108	-0.352108471	2.469725738	2.686833264	2.252618212
84449	5.0319334	4.7105331	4.871233248	2.94739082	3.594997091	4.242603362
84450	-0.352108471	-0.352108	-0.352108471	0.849500091	2.051108653	-0.352108471
84451	-0.352108471	-0.352108	-0.352108471	4.499096633	6.740136862	5.619616747
84464	3.87704012	7.2156943	5.546367231	1.227667732	1.646479888	0.808855576
84466	1.608183739	3.5690192	2.588601447	-0.352108471	-0.352108471	-0.352108471
84498	2.800331696	5.6251608	8.449989847	6.32531018	4.554082567	8.096537794
84501	3.921862223	4.5373358	5.152809293	1.043426334	0.440372779	1.646479888
84503	7.4871992	7.6430046	7.331393785	6.132384991	6.417266267	6.274825629
84504	5.117115863	5.1799508	5.054280952	-0.352108471	-0.352108471	-0.352108471
84516	5.253175981	8.9292149	7.091195445	10.18367458	4.832419964	7.508047272
84520	-0.352108471	-0.352108	-0.352108471	5.789762726	5.85807738	5.721448072
84522	-0.352108471	-0.352108	-0.352108471	4.66005769	1.120964058	8.199151321
84530	5.159434386	4.2452143	6.073654487	5.584676343	5.605609608	5.563743079
84532	5.657493881	6.1596782	5.155309599	-0.352108471	-0.352108471	-0.352108471
84542	5.920533403	6.6647303	7.408927118	3.685639275	5.494417177	1.876861374
84548	4.377360553	8.4511557	6.414258136	-0.352108471	-0.352108471	-0.352108471
84552	6.550959389	9.3230193	7.936989338	2.310899623	3.677812865	5.044726108
84557	6.429770168	6.7181678	6.573968999	9.293231508	3.799598355	6.546414932
84561	5.852491963	7.9876068	3.717377078	5.889175989	7.091429582	6.490302786
84570	-0.352108471	-0.352108	-0.352108471	6.693937107	7.657204881	5.730669333
84572	6.212536209	6.2312245	6.193847962	7.04681923	7.065023967	7.028614492
84612	6.486915185	6.8399467	6.133883699	5.786662435	5.659615047	5.913709822
84617	7.150225809	7.8972681	8.644310295	10.18469383	8.577244196	9.380969011
84618	2.413921152	5.1799508	-0.352108471	-0.352108471	-0.352108471	-0.352108471
84619	2.553073296	-0.352108	5.458255064	-0.352108471	-0.352108471	-0.352108471
84620	5.275595562	6.6012704	5.938433002	3.087716148	0.907438756	1.997577452
84622	3.498170515	8.7460628	6.122116649	0.440372779	0.440372779	0.440372779
84627	8.641706723	4.2856431	6.463674915	1.72593607	3.585121301	5.444306532
84629	5.749402114	5.1828024	6.31600185	6.449861915	5.678587805	7.221136025
84634	-0.352108471	-0.352108	-0.352108471	2.38621749	5.124543452	-0.352108471
84640	6.008766053	4.5503078	3.091849551	5.958359385	6.079118266	5.837600503
84641	6.073207852	4.1991649	7.94725076	5.789762726	6.239358644	5.340166808
84645	6.152796703	4.417152	5.284974356	-0.352108471	-0.352108471	-0.352108471
84650	-0.352108471	-0.352108	-0.352108471	6.872566731	4.6775697	5.775068216
84656	8.280933203	6.3831515	4.485369789	8.88055728	7.572831864	6.265106447
84661	-0.352108471	-0.352108	-0.352108471	1.959896095	5.078758622	3.519327359
84665	6.121528769	7.564269	4.678788501	2.732252495	3.919505936	5.106759376

84671	4.836101998	4.617923	5.054280952	1.518484901	1.731739118	1.305230684
84674	6.969212549	6.1166741	7.821750959	6.914644543	5.066468176	8.762820909
84678	-0.352108471	-0.352108	-0.352108471	5.83433693	2.658540408	9.010133452
84681	3.445523539	2.7115616	1.977599573	-0.352108471	-0.352108471	-0.352108471
84687	4.107001209	5.1522471	6.197493017	6.000652426	5.095423988	4.19019555
84693	-0.352108471	-0.352108	-0.352108471	6.434644758	6.400751804	6.468537712
84705	5.797886585	5.9078411	5.852863847	6.136795257	7.068811929	5.204778586
84709	-0.352108471	-0.352108	-0.352108471	6.663856474	6.689878453	6.637834496
84717	-0.352108471	-0.352108	-0.352108471	6.862268088	5.950299088	6.406283588
84719	4.155596192	6.8142431	5.484919652	2.168105043	2.225020927	2.111189159
84722	6.625218558	5.8529375	5.080656365	0.044132154	0.440372779	-0.352108471
84726	8.028411128	7.4061827	8.650639552	6.078995398	6.235921161	6.392846924
84734	-0.352108471	-0.352108	-0.352108471	5.529311366	5.529311366	5.529311366
84747	7.595762813	5.3546856	3.113608485	5.409208735	5.579625618	5.494417177
84752	-0.352108471	-0.352108	-0.352108471	7.097456946	6.096058685	6.596757815
84759	-0.352108471	-0.352108	-0.352108471	4.279364163	5.095423988	5.911483813
84765	5.089412202	5.1245435	5.054280952	-0.352108471	-0.352108471	-0.352108471
84766	5.688622647	5.7155573	5.661687951	0.897715333	-0.352108471	2.147539136
84767	5.284974356	5.3152588	5.254689875	2.760618758	5.873345987	-0.352108471
84769	-0.352108471	-0.352108	-0.352108471	8.487081553	5.077006571	6.782044062
84771	7.728287468	6.2336451	6.980966299	7.261244111	1.415050014	4.338147062
84777	8.848406619	7.9088982	8.378652411	6.796814879	8.554830984	5.038798775
84787	-0.352108471	-0.352108	-0.352108471	4.80208491	5.364539421	4.239630399
84790	8.83022732	8.077371	8.453799139	9.572867736	9.267430767	8.961993798
84793	-0.352108471	-0.352108	-0.352108471	4.544323499	7.988410888	6.266367194
84798	5.855905479	7.2185983	6.537251893	-0.352108471	-0.352108471	-0.352108471
84803	6.567831173	7.6323898	8.696948446	6.015503565	4.602173492	7.428833637
84807	7.008755637	5.4828969	6.245826291	5.184847066	-0.804732305	2.19005738
84809	-0.352108471	-0.352108	-0.352108471	4.21934628	2.065292044	-0.088762191
84817	-0.352108471	-0.352108	-0.352108471	4.841747932	6.56915909	8.296570247
84818	-0.352108471	-0.352108	-0.352108471	6.396901492	6.369156591	6.341411689
84833	3.427182756	6.2125362	8.997889661	1.223721097	1.305230684	1.14221151
84836	-0.352108471	-0.352108	-0.352108471	7.753432823	5.663737322	3.57404182
84838	-0.352108471	-0.352108	-0.352108471	2.686833264	2.686833264	2.686833264
84849	1.783698732	3.9195059	-0.352108471	3.330812986	2.857244297	3.804381674
84851	5.068573035	4.0160458	2.963518507	1.79269925	-0.341531925	3.926930425
84852	0.064641487	11.052894	5.55876787	-0.352108471	-0.352108471	-0.352108471
84856	5.251244996	4.5553643	3.859483687	6.333721317	5.693160971	6.013441144
84864	-0.352108471	-0.352108	-0.352108471	6.396868383	3.876720712	8.917016053
84865	8.264193649	9.9813489	9.12277128	6.827448783	8.071076431	7.449262607
84866	-0.352108471	-0.352108	-0.352108471	5.886425029	7.649557788	4.12329227
84868	8.082609643	6.9667435	5.8508773	3.214328646	2.875005529	3.553651763
84872	1.392109249	3.136327	-0.352108471	4.477947052	4.843308497	4.112585606
84875	-0.352108471	-0.352108	-0.352108471	7.204683996	6.066900737	4.929117477
84876	4.434314669	4.3467833	4.521846024	6.840114058	7.988534477	5.691693639
84878	-0.352108471	-0.352108	-0.352108471	7.928318258	5.668425445	3.408532632
84881	-0.352108471	-0.352108	-0.352108471	-1.603209609	2.124237496	5.851684601

84883	2.065292044	-0.352108	4.482692559	4.825459812	5.731413688	3.919505936
84885	-0.352108471	-0.352108	-0.352108471	7.716899633	6.864244415	6.011589196
84889	-0.352108471	-0.352108	-0.352108471	5.794915175	5.789762726	5.784610276
84893	-0.352108471	-0.352108	-0.352108471	4.979855164	4.80208491	4.624314656
84896	-0.352108471	-0.352108	-0.352108471	5.523784224	6.651504685	6.087644454
84897	1.917440458	4.8341019	7.750763417	5.881152222	4.242739248	7.519565196
84898	4.680663176	3.9685185	3.256373879	7.100847388	6.410056867	6.755452127
84901	5.189616664	7.4464787	2.932754661	5.862922053	3.983878847	7.741965259
84904	6.376628371	6.394396	6.385512177	8.779362233	4.153642176	6.466502204
84909	7.60927147	6.8585836	8.359959313	3.659315043	5.863200788	1.455429298
84912	7.898998598	5.987128	4.075257444	5.109168614	7.721093571	6.415131092
84913	-0.352108471	-0.352108	-0.352108471	6.356430144	7.05414488	6.705287512
84914	6.210802504	6.0384535	6.383151496	5.293014658	5.825167992	4.760861323
84916	2.405615662	2.7005309	2.553073296	6.32345662	4.593053508	5.458255064
84918	6.425665899	4.5091748	5.467420327	8.312100266	7.021301473	5.73050268
84923	-0.352108471	-0.352108	-0.352108471	6.766960947	6.28894386	7.244978034
84924	5.136690127	4.9934779	5.065084027	3.861329726	6.32951825	5.095423988
84926	6.625961662	6.5887933	6.607377459	7.468215014	6.247115888	8.689314139
84928	7.76921396	7.357799	7.563506482	6.306115104	6.935635899	5.676594309
84929	5.939192221	7.1358046	4.742579887	-0.352108471	-0.352108471	-0.352108471
84932	6.173142889	6.2379707	6.108315099	2.742642846	0.710777182	4.77450851
84934	-0.352108471	-0.352108	-0.352108471	6.962788071	5.035153547	5.998970809
84936	7.283024581	5.3546311	3.42623756	-0.352108471	-0.352108471	-0.352108471
84937	-0.352108471	-0.352108	-0.352108471	7.893658173	6.266367194	4.639076214
84938	-0.352108471	-0.352108	-0.352108471	6.22028448	6.0395156	6.12990004
84939	-0.352108471	-0.352108	-0.352108471	3.784849312	2.239730506	3.012289909
84941	3.683980801	2.8783494	4.48961218	2.212158027	2.389615384	2.03470067
84945	5.721859655	6.5211979	6.121528769	-0.352108471	-0.352108471	-0.352108471
84950	-0.352108471	-0.352108	-0.352108471	6.147915031	5.893442277	6.402387785
84951	5.939192221	6.6110324	5.267352033	0.791292145	1.14221151	0.440372779
84952	7.214932646	5.629874	4.044815254	2.498005078	3.119148782	1.876861374
84955	-0.352108471	-0.352108	-0.352108471	6.384445387	5.295953578	5.840199482
84959	-0.352108471	-0.352108	-0.352108471	2.452355666	4.717886936	3.585121301
84961	9.897981083	9.5510341	9.204087168	9.937376152	6.218264246	8.077820199
84962	1.383520155	-0.352108	3.119148782	7.521308925	7.55064888	7.49196897
84967	6.580916434	6.573969	6.567021565	3.884993804	7.834240934	5.859617369
84968	-0.352108471	-0.352108	-0.352108471	4.559699198	6.369984184	2.749414213
84973	6.6890844	7.2173176	7.745550831	7.054707329	6.431979261	5.809251193
84975	5.467785857	5.5587679	5.649749884	1.90830372	1.939746065	1.876861374
84981	7.630589424	8.1981511	7.063027705	5.169157826	6.821277412	3.517038239
84984	5.314182891	4.8686712	5.759694547	5.769936357	6.276815009	6.783693662
84991	5.155398661	6.7545585	8.353718391	3.665466294	3.669796527	3.674126761
84993	1.383520155	-0.352108	3.119148782	6.596757815	7.586527141	5.60698849
84996	5.958359385	5.9583594	5.958359385	2.469725738	2.252618212	2.686833264
85002	5.468477282	6.2738323	7.079187236	-0.352108471	-0.352108471	-0.352108471
85012	2.065292044	-0.352108	4.482692559	7.39796665	7.592535142	7.787103634
85014	-0.352108471	-0.352108	-0.352108471	6.111624209	2.945013769	9.27823465

85016	5.272198236	3.3589528	4.315575532	3.365217942	2.607687719	4.122748165
85021	8.099635869	7.7641727	8.435099048	4.198376291	6.51496239	8.83154849
85026	5.625160771	4.8962877	6.354033885	0.440372779	0.440372779	0.440372779
85027	6.765298135	7.0115295	6.888413829	-0.352108471	-0.352108471	-0.352108471
85300	9.493130187	8.9153355	8.33754074	7.645974262	8.127485912	7.164462612
85301	6.832572932	7.4650603	6.200085577	0.228373553	0.808855576	-0.352108471
85302	-0.352108471	-0.352108	-0.352108471	3.453806753	0.970079685	2.211943219
85315	2.38621749	5.1245435	-0.352108471	0.289051906	-0.352108471	0.930212283
85320	-0.352108471	-0.352108	-0.352108471	2.168105043	4.688318557	-0.352108471
85359	4.778799825	3.136327	6.421272681	6.621168045	6.653870824	6.588465267
85360	6.273832259	6.2858739	6.261790604	7.8752966	7.886559523	7.864033677
85364	-0.352108471	-0.352108	-0.352108471	4.579410823	6.897845428	5.738628126
85365	5.402265996	6.4636749	7.525083834	-0.352108471	-0.352108471	-0.352108471
85366	6.676242611	9.1722276	7.924235121	1.854057389	9.996704403	5.925380896
85371	7.343076225	6.6904766	7.016776417	5.643490857	5.27122146	4.898952063
85376	8.161766964	3.201689	5.681727979	3.428141285	4.782566466	2.073716103
85377	6.787134073	5.0416624	8.53260573	-0.352108471	-0.352108471	-0.352108471
85378	4.825897128	5.6090851	5.217491139	4.648370104	-0.517786016	2.065292044
85379	1.383520155	-0.352108	3.119148782	1.167362396	-0.352108471	2.686833264
85397	-0.352108471	-0.352108	-0.352108471	4.456886199	5.342972175	3.570800224
85406	1.999010143	1.6620152	1.325020218	1.717718408	1.179374859	2.256061956
85407	6.261203695	7.1052531	5.41715427	6.288357381	7.012139391	5.564575371
85415	3.120551286	4.2090916	2.032011016	4.456886199	3.752331459	5.16144094
85437	-0.352108471	-0.352108	-0.352108471	6.678528048	9.509173508	3.847882587
85440	5.493783215	5.3450549	5.642511495	6.585987027	7.612525434	5.559448621
85441	6.28161222	5.2500678	7.31315665	3.853086798	3.510198867	3.167310937
85442	5.718459767	7.871617	3.565302584	-0.352108471	-0.352108471	-0.352108471
85443	-0.352108471	-0.352108	-0.352108471	5.872806176	6.567671021	6.220238598
85444	-0.352108471	-0.352108	-0.352108471	2.571154353	3.68200639	1.460302315
85450	6.572304858	7.461248	7.016776417	-0.352108471	-0.352108471	-0.352108471
85456	6.257046343	7.1297248	5.384367915	-0.352108471	-0.352108471	-0.352108471
85457	6.20889986	6.4517249	6.694549978	7.620346447	5.377523302	6.498934874
85459	6.089299524	5.8001998	5.511100051	-0.352108471	-0.352108471	-0.352108471
85460	-0.352108471	-0.352108	-0.352108471	2.902991023	2.686833264	3.119148782
85461	5.052267685	4.8285765	5.275958864	-0.352108471	-0.352108471	-0.352108471
85463	6.27004087	5.5913056	4.912570307	-0.352108471	-0.352108471	-0.352108471
85465	4.697886322	5.2532408	5.808595262	5.821410838	3.292015634	8.350806041
85476	-0.352108471	-0.352108	-0.352108471	6.204656258	2.874668701	9.534643815
85477	1.383520155	-0.352108	3.119148782	1.90830372	1.939746065	1.876861374
85478	-0.352108471	-0.352108	-0.352108471	5.704335255	2.888348999	8.520321511
85509	-0.352108471	-0.352108	-0.352108471	5.859617369	5.671499241	6.047735497
85865	6.356780189	7.1058108	7.85484144	8.481056576	4.967882955	6.724469766
87178	10.66965894	7.3348011	9.002230017	8.40188526	7.347690401	6.293495543
88455	7.185765998	7.2040879	7.167444067	6.77374078	6.788420481	6.759061079
88745	-0.352108471	-0.352108	-0.352108471	6.792568998	6.121096218	5.449623439
89122	8.252623863	6.2725067	4.292389578	8.029451742	6.391703033	4.753954324
89777	4.178899946	4.1125856	4.245214285	6.266367194	6.276856601	6.255877787

89782	7.633665131	1.8027719	4.718218516	2.697772388	4.66005769	6.622342991
89790	7.02788799	6.9268019	7.128974067	3.055408374	3.919505936	2.191310813
89795	-0.352108471	-0.352108	-0.352108471	6.822208827	4.590523374	9.053894279
89796	5.656532109	6.4786045	6.067568295	7.288053042	5.882959551	8.693146532
89846	6.474775011	5.7103767	4.9459783	-0.352108471	-0.352108471	-0.352108471
89857	4.893310105	3.737841	4.315575532	5.457998494	8.810281472	2.105715516
89858	6.69160764	5.0522677	3.412927731	-0.352108471	-0.352108471	-0.352108471
89866	-0.352108471	-0.352108	-0.352108471	6.32531018	6.33538923	6.31523113
89872	-0.352108471	-0.352108	-0.352108471	1.41945414	2.049256654	2.679059168
89883	3.613387334	4.5373358	5.461284182	-0.352108471	-0.352108471	-0.352108471
89890	7.692324422	8.3191935	7.065455358	6.377487473	6.178989656	6.278238565
89891	7.441788381	6.7667812	8.116795577	5.869665514	6.129228791	5.610102238
89910	-0.352108471	-0.352108	-0.352108471	6.165870606	7.342381689	4.989359523
89932	5.553615219	7.8347761	6.694195663	6.092908426	6.538483369	5.647333483
89941	8.101656291	7.9601021	7.818547982	5.723310862	6.908528552	8.093746242
89970	5.833726066	5.980334	6.12694199	-0.352108471	-0.352108471	-0.352108471
90007	7.584087926	5.2285043	9.939671503	5.10873923	8.078948179	6.593843704
90011	6.895865703	7.6906661	6.101065333	6.488107881	8.571202555	4.405013207
90025	1.11708714	1.7836987	2.450310324	6.40631083	5.376604349	7.436017311
90060	1.25047138	3.9685185	6.686565676	3.61631576	6.567007149	9.517698537
90102	-0.352108471	-0.352108	-0.352108471	6.992253866	6.829307464	6.666361061
90135	8.802912536	6.4923205	7.64761654	-0.352108471	-0.352108471	-0.352108471
90141	8.230464395	7.6536667	7.076869035	1.864658285	1.731739118	1.997577452
90203	4.033545615	6.6075809	5.320563245	-0.352108471	-0.352108471	-0.352108471
90204	2.065292044	-0.352108	4.482692559	4.580727159	7.889977403	6.235352281
90231	5.354957525	5.1522471	4.949536702	6.112587778	7.517115756	4.708059801
90233	6.461098925	3.2226986	4.841898744	1.29104505	5.607672285	3.449358668
90246	-0.352108471	-0.352108	-0.352108471	4.08236011	1.271461797	6.893258424
90249	-0.352108471	-0.352108	-0.352108471	5.430898714	5.458255064	5.403542364
90313	3.515141236	3.8300956	3.200186845	7.334242202	7.40032982	7.466417438
90317	-0.352108471	-0.352108	-0.352108471	5.295215654	5.017136436	4.739057218
90321	5.372783739	4.671913	6.073654487	2.278504072	1.437859363	3.119148782
90324	4.178899946	4.5809407	3.776859191	5.324738906	8.151770667	2.497707144
90333	6.846957097	4.7216163	8.972297904	1.475082136	1.807952763	1.14221151
90338	5.780731917	7.2363344	6.508533137	6.993768266	5.20089139	3.408014513
90355	5.291901397	6.4139735	5.852937462	5.929817327	5.736862343	6.122772311
90379	6.623540933	9.5099351	3.73714675	5.584676343	4.47453227	6.694820416
90390	6.378273034	6.626253	6.502263028	-0.352108471	-0.352108471	-0.352108471
90407	6.333717463	5.9384179	6.729016984	2.541584564	3.22141505	2.881499807
90410	5.659278871	5.9376626	5.380895166	-0.352108471	-0.352108471	-0.352108471
90411	7.192639764	10.261353	13.33006694	9.476682029	8.254104243	8.865393136
90416	2.186071746	4.1125856	6.039099466	7.761136634	6.022050925	4.282965216
90417	5.55876787	5.5882244	5.529311366	-0.352108471	-0.352108471	-0.352108471
90441	4.153967827	3.6761388	4.631796822	7.172442894	7.300708227	7.044177561
90480	7.224081961	8.0130371	6.435126823	5.13265557	6.5583441	5.845499835
90485	6.746204904	6.1811449	6.463674915	2.947798352	5.636809111	8.32581987
90499	3.435007638	4.0251747	4.61534179	4.743879823	3.818098858	2.892317893

90507	6.396868383	9.0588658	3.734870943	4.930505787	5.174029583	5.052267685
90523	5.017136436	6.274379	3.759893835	-0.352108471	-0.352108471	-0.352108471
90525	-0.352108471	-0.352108	-0.352108471	5.494417177	3.90268819	7.086146163
90550	5.796951735	6.996785	6.396868383	-0.352108471	-0.352108471	-0.352108471
90624	8.979045907	9.6703846	8.287707201	9.13784247	7.758758575	6.379674679
90627	-0.352108471	-0.352108	-0.352108471	5.748575889	6.691901308	6.220238598
90632	5.463922025	5.5293114	5.398532684	-0.352108471	-0.352108471	-0.352108471
90637	5.284974356	5.9833132	4.586635527	-0.352108471	-0.352108471	-0.352108471
90649	6.46696622	5.719565	4.972163768	3.627756523	4.683557056	2.571955991
90668	2.741135556	2.8779269	3.014718167	-0.352108471	-0.352108471	-0.352108471
90701	-0.352108471	-0.352108	-0.352108471	6.204656258	5.275547027	7.133765489
90780	5.828848464	5.825168	5.832528937	-0.352108471	-0.352108471	-0.352108471
90784	4.225476119	3.5632483	3.894362219	1.87051746	2.191310813	1.549724107
90799	8.128107665	6.2398609	4.351614212	-0.352108471	-0.352108471	-0.352108471
90806	5.895195597	6.3988044	6.147000016	4.274943274	9.771120937	7.023032106
90809	1.188791836	4.1125856	7.036379376	6.497182024	6.211082415	6.783281634
90826	3.452766383	6.3045646	4.878665478	-0.352108471	-0.352108471	-0.352108471
90827	-0.352108471	-0.352108	-0.352108471	6.011060592	5.233567424	4.456074255
90850	2.980157593	5.4937832	8.007408837	5.88700099	6.036990268	5.737011711
90853	7.800060623	7.8275848	7.772536433	7.649062677	7.682866779	7.615258575
90861	8.477997976	5.2404366	6.859217306	-0.352108471	-0.352108471	-0.352108471
90864	6.590508304	5.4137442	7.767272436	-0.352108471	-0.352108471	-0.352108471
90871	-0.352108471	-0.352108	-0.352108471	5.406707798	5.371019024	5.442396573
90933	-0.352108471	-0.352108	-0.352108471	1.492819042	3.337746555	-0.352108471
90957	7.016776417	3.3851838	10.648369	7.737544924	6.739878825	5.742212726
90993	6.610895082	6.7871341	6.963373064	4.058714557	7.021818365	9.984922174
91010	5.199889546	6.4333187	7.666747909	3.585121301	5.152763901	2.017478701
91012	6.851278433	8.0325481	5.670008745	0.538184292	3.299606409	1.918895351
91039	7.97794913	8.7396636	9.501378166	7.186741904	8.282993987	6.09048982
91056	6.803586453	7.3154815	6.291691359	5.343911315	5.926961306	4.760861323
91107	-0.352108471	-0.352108	-0.352108471	5.575983727	5.588224375	5.563743079
91120	6.619197037	6.479333	6.759061079	1.937219413	1.997577452	1.876861374
91156	-0.352108471	-0.352108	-0.352108471	6.196133489	7.653201227	4.739065751
91179	4.178899946	4.7699746	3.587825308	5.917668693	6.965316847	8.012965002
91252	-0.352108471	-0.352108	-0.352108471	4.325505146	7.682451865	6.003978506
91289	6.342244821	9.3109461	7.826595454	6.545950789	8.401990643	4.689910934
91300	6.77374078	5.1922051	8.35527642	-0.352108471	-0.352108471	-0.352108471
91304	7.483407675	10.5149	4.451915026	7.165804888	6.02456832	8.307041457
91316	-0.352108471	-0.352108	-0.352108471	6.32531018	6.33538923	6.31523113
91369	5.71551863	6.466297	6.090907831	5.017136436	5.037481686	4.996791185
91373	5.016491534	5.3673546	5.718217754	5.930371471	6.904123208	6.41724734
91408	1.383520155	-0.352108	3.119148782	6.63770735	7.818531425	5.456883274
91442	4.6888805	6.1729579	3.204803148	-0.352108471	-0.352108471	-0.352108471
91445	-0.352108471	-0.352108	-0.352108471	5.991735221	5.815785788	5.903760504
91450	7.12416845	6.573969	6.023769549	-0.352108471	-0.352108471	-0.352108471
91452	6.760866103	6.2117907	5.66271525	1.689109503	1.646479888	1.731739118
91523	1.383520155	0.6261753	2.140864999	5.20596667	6.754701387	5.980334028

91544	-0.352108471	-0.352108	-0.352108471	4.456886199	4.482692559	4.43107984
91548	5.173810713	5.9562314	6.738652149	4.750963477	7.999596828	6.375280153
91574	9.719548766	8.1628634	6.606178131	7.74186346	4.71383197	6.227847715
91582	4.886952939	7.3607807	9.834608413	6.64213435	6.662724824	6.621543876
91612	6.007101566	4.4557793	2.904456987	4.294153815	6.5059697	5.400061757
91624	7.388668438	7.5719245	7.205412329	-0.352108471	-0.352108471	-0.352108471
91647	-0.352108471	-0.352108	-0.352108471	4.520286589	5.369332089	6.218377588
91653	-0.352108471	-0.352108	-0.352108471	6.690172	5.08382998	5.88700099
91661	2.711561556	-0.352108	5.775231583	0.044132154	-0.352108471	0.440372779
91663	8.461775661	7.6512983	8.056536983	9.030316954	9.150258145	8.910375764
91683	5.308889672	6.6517784	5.980334028	-0.352108471	-0.352108471	-0.352108471
91695	5.371621734	5.980334	6.589046323	4.20031331	6.369635403	5.284974356
91748	5.391018164	6.3199067	4.462129642	6.849541372	7.403503448	7.12652241
91749	8.471837241	6.9679349	9.975739554	6.736691293	6.173080407	6.45488585
91754	10.02239479	7.4969383	4.971481897	1.223721097	1.151458371	1.295983823
91775	7.274390182	2.4290792	4.851734675	4.313590712	4.904478682	5.495366652
91801	-0.352108471	-0.352108	-0.352108471	4.845290992	5.118656602	5.392022211
91807	8.127417516	8.838326	7.416509	6.613210803	5.341903266	7.88451834
91862	4.682964349	4.9799919	4.385936778	1.530736894	2.252618212	0.808855576
91869	-0.352108471	-0.352108	-0.352108471	5.537668162	5.745394463	5.953120764
91875	-0.352108471	-0.352108	-0.352108471	3.136326969	3.119148782	3.153505157
91893	5.186138762	5.4582551	4.914022459	2.169424975	2.147539136	2.191310813
91942	8.707715234	5.6077	7.15770761	5.87051543	6.44086039	7.011205349
91948	8.300571608	9.010354	7.590789181	9.12907743	7.5316884	5.93429937
91949	-0.352108471	-0.352108	-0.352108471	5.845499835	5.275594597	6.415405073
91977	3.527916452	3.136327	3.919505936	5.780944036	6.246629235	5.315258837
91978	-0.352108471	-0.352108	-0.352108471	5.369332089	5.668264776	5.070399401
92002	-0.352108471	-0.352108	-0.352108471	6.165870606	2.831255972	9.50048524
92014	8.202136007	7.6744054	8.729866607	5.639711591	6.536514975	7.433318358
92017	-0.352108471	-0.352108	-0.352108471	5.233567424	8.165540744	2.301594103
92070	5.933346321	6.183964	6.43458171	1.475082136	-0.196735912	3.146900185
92092	6.519673958	5.4398195	7.599528386	3.8208192	4.559699198	5.298579197
92126	9.27421614	7.3790032	5.483790181	6.715221125	8.277602281	9.839983437
92140	6.709007107	6.2595096	5.810012155	7.069785735	6.77205408	6.920919907
92154	6.440466489	6.3520539	6.263641238	7.999579836	7.690758392	7.381936948
92170	-0.352108471	-0.352108	-0.352108471	5.886425029	6.284876226	5.487973831
92181	-0.352108471	-0.352108	-0.352108471	6.514344938	4.178777881	8.849911996
92285	10.17983947	6.4686213	8.324230385	7.434456273	8.642858517	8.038657395
92291	6.991795048	8.3395212	5.644068901	-0.352108471	-0.352108471	-0.352108471
92305	-0.352108471	-0.352108	-0.352108471	5.233567424	5.254689875	5.212444972
92335	-0.352108471	-0.352108	-0.352108471	5.919436356	5.723485516	5.527534675
92345	4.016045771	4.2589309	3.773160614	3.241701208	6.276856601	9.312011993
92400	-0.352108471	-0.352108	-0.352108471	3.585121301	2.690317848	4.479924754
92421	4.439206069	5.1245435	5.809880834	3.768326697	1.939527756	0.110728816
92521	-0.352108471	-0.352108	-0.352108471	7.076901479	7.088778992	7.065023967
92579	0.296841853	1.3835202	2.470198458	7.507600459	7.070555652	7.289078056
92609	10.80763819	8.2221038	5.636569487	7.391802441	7.227421543	7.063040645

92610	-0.352108471	-0.352108	-0.352108471	5.141739075	6.296043865	7.450348656
92659	0.486617086	1.6620152	2.837413275	6.682963902	5.64354012	4.604116337
92689	5.97774402	6.5502319	7.122719701	7.362089337	8.552040358	6.172138317
92691	5.110388728	5.3989618	5.687534962	0.762376451	-0.352108471	1.876861374
92703	-0.352108471	-0.352108	-0.352108471	6.001809131	5.930794706	6.072823557
92714	5.775231583	4.5960915	6.954371674	-0.352108471	-0.352108471	-0.352108471
92715	-0.352108471	-0.352108	-0.352108471	4.08236011	3.307512663	4.857207558
92737	-0.352108471	-0.352108	-0.352108471	6.036056902	5.338134843	4.640212784
92797	-0.352108471	-0.352108	-0.352108471	6.916603554	6.614053211	7.219153897
92799	-0.352108471	-0.352108	-0.352108471	2.082455004	2.342793532	2.60313206
92806	5.740630976	6.0377833	5.889207138	1.05704313	1.305230684	0.808855576
92822	3.797822384	5.5406981	2.054946695	5.591305588	7.325226398	3.857384779
92856	-0.352108471	-0.352108	-0.352108471	3.384482817	5.624431044	1.144534591
92912	6.643175906	5.5587679	4.474359834	-0.352108471	-0.352108471	-0.352108471
93010	4.311279682	3.1277379	1.944196069	3.315115848	4.25511609	2.375115605
93100	6.020381091	7.6245748	4.416187361	5.766785892	7.75792346	6.762354676
93129	7.646733741	5.6886226	3.730511553	4.975647011	6.070832428	5.523239719
93164	7.031788726	7.6409192	8.250049657	3.364509603	6.158865923	8.953222244
93183	6.348298929	7.1458688	7.943438692	1.434979174	1.939746065	0.930212283
93323	5.664172181	6.0736545	5.254689875	7.076901479	7.088778992	7.065023967
93377	-0.352108471	-0.352108	-0.352108471	6.579427792	3.375993044	4.977710418
93380	-0.352108471	-0.352108	-0.352108471	2.211943219	3.078892472	1.344993966
93436	-0.352108471	-0.352108	-0.352108471	4.500730297	4.66005769	4.819385082
93487	8.779482436	7.4075938	6.035705229	8.093256519	6.420771494	9.765741543
93556	5.551643179	7.253083	6.402363066	6.837727763	5.860408356	6.34906806
93611	4.697621232	6.3213699	5.509495557	2.689753515	8.128679439	5.409216477
93621	7.5721129	6.9546757	6.33723851	8.825012889	7.767667959	6.71032303
93624	7.735408894	7.6152586	7.855559213	5.10819936	5.315258837	4.901139884
93627	-0.352108471	-0.352108	-0.352108471	3.446982306	4.954218546	1.939746065
93649	6.573968999	6.5884653	6.559472732	6.739878825	6.759061079	6.720696571
93663	-0.352108471	-0.352108	-0.352108471	3.313060576	4.851659645	4.08236011
93664	2.62245663	-0.352108	5.597021732	-0.352108471	-0.352108471	-0.352108471
93974	5.82803966	6.7777281	6.302883855	8.154471537	7.596099384	7.037727232
93986	1.392109249	1.612067	1.172151524	3.885702903	0.163658162	2.024680532
94015	6.829912859	5.9638239	6.396868383	-0.352108471	-0.352108471	-0.352108471
94025	5.946049998	7.0319237	8.117797456	-0.352108471	-0.352108471	-0.352108471
94027	7.099953796	5.2359798	6.167966808	-0.352108471	-0.352108471	-0.352108471
94056	5.987162856	6.003228	6.019293182	6.71873029	6.776545416	6.747637853
94059	5.661692129	7.1320446	6.396868383	-0.352108471	-0.352108471	-0.352108471
94081	5.256268008	4.596919	5.915617032	7.889303643	6.900899666	5.912495689
94101	5.217491139	4.3654974	6.069484887	-0.352108471	-0.352108471	-0.352108471
94104	-0.352108471	-0.352108	-0.352108471	6.087644454	6.101634422	6.073654487
94134	-0.352108471	-0.352108	-0.352108471	1.995427359	2.051108653	1.939746065
94137	5.493783215	3.0404874	7.947078997	-0.352108471	-0.352108471	-0.352108471
94160	5.179868644	4.7110274	5.648709911	-0.352108471	-0.352108471	-0.352108471
94234	6.239539073	5.2849744	4.330409639	-0.352108471	-0.352108471	-0.352108471
94239	-0.352108471	-0.352108	-0.352108471	7.843946541	6.775428903	8.912464178

94241	-0.352108471	-0.352108	-0.352108471	4.152826211	6.56915909	8.985491968
95681	6.735779686	6.3194103	7.152149096	-0.352108471	-0.352108471	-0.352108471
96459	3.465763751	5.5302552	7.594746726	6.488549187	5.912903045	6.200726116
96626	4.016045771	1.971735	6.060356584	1.270654317	0.440372779	2.100935855
103910	9.617685135	9.8077655	9.427604818	9.312977242	8.209606328	8.761291785
112401	-0.352108471	-0.352108	-0.352108471	5.688622647	4.362095569	7.015149725
112464	7.185765998	4.7531756	9.618356393	6.876579765	7.078465349	6.674694182
112483	-0.352108471	-0.352108	-0.352108471	7.586354189	6.99529589	6.404237591
112487	9.249420068	8.0667995	6.88417892	5.420879354	2.673938688	8.167820021
112495	5.919459543	5.9583594	5.880559702	5.233567424	5.254689875	5.212444972
112597	7.707647354	9.3545475	8.531097423	7.288722029	6.99593518	6.703148332
112611	-0.352108471	-0.352108	-0.352108471	4.178899946	2.025565681	6.332234211
112755	4.259723664	3.6761388	4.843308497	0.289051906	-0.352108471	0.930212283
112770	3.805664353	3.9195059	3.69182277	6.648899212	6.63062078	6.667177643
112812	6.607377459	6.7393641	6.475390811	5.529311366	5.217852579	5.840770153
112817	8.33084037	4.9824946	6.656667509	6.131175268	5.840338979	6.422011557
112849	-0.352108471	-0.352108	-0.352108471	0.727678478	3.919505936	7.111333393
112869	-0.352108471	-0.352108	-0.352108471	5.886425029	4.296838028	7.47601203
112936	-0.352108471	-0.352108	-0.352108471	8.170666918	6.705287512	5.239908106
112937	4.112585606	3.5540616	4.671109593	5.432742342	2.095853319	8.769631366
112939	5.876276997	6.507143	5.245410946	7.144947014	8.081466428	9.017985843
112950	-0.352108471	-0.352108	-0.352108471	5.494417177	5.494417177	5.494417177
113000	-0.352108471	-0.352108	-0.352108471	7.716865863	6.676264726	7.196565294
113026	6.444650146	6.3324667	6.220283192	5.494664348	8.694035602	7.094349975
113130	-0.352108471	-0.352108	-0.352108471	5.372211021	3.304083104	4.338147062
113146	5.719564994	5.9510862	5.488043761	0.762376451	-0.352108471	1.876861374
113157	7.882737453	3.7812592	5.831998311	6.296534544	5.229100122	5.762817333
113178	6.624675593	3.5821848	9.667166343	7.511116711	8.930133989	8.22062535
113179	-0.352108471	-0.352108	-0.352108471	5.048691416	7.063416581	6.056053999
113189	-0.352108471	-0.352108	-0.352108471	6.51496239	6.532588736	6.497336045
113201	4.094704148	4.4385056	4.782306959	4.303912246	5.225490971	3.382333521
113246	6.690302477	6.6930999	6.687505103	6.445143931	4.923713223	5.684428577
113251	-0.352108471	-0.352108	-0.352108471	4.927679215	4.954218546	4.901139884
113277	7.890827346	5.9573941	9.824260545	6.691957149	6.595493818	6.788420481
113278	-0.352108471	-0.352108	-0.352108471	6.149337851	6.309687355	5.988988346
113402	6.932009707	5.6886226	4.445235587	4.343111875	4.927679215	5.512246555
113444	-0.352108471	-0.352108	-0.352108471	6.60719621	6.79004344	6.97289067
113510	4.918926546	4.4826926	5.355160532	-0.352108471	-0.352108471	-0.352108471
113612	-0.352108471	-0.352108	-0.352108471	5.859617369	5.370299232	6.348935506
113691	7.184411154	5.1570997	6.17075544	5.375956071	6.313902726	5.844929398
113829	1.383520155	-0.352108	3.119148782	8.23304433	2.505619847	5.369332089
113878	5.456586205	5.7494021	6.042218023	-0.352108471	-0.352108471	-0.352108471
114026	5.240570946	2.1409703	3.690770627	4.944973278	2.007275259	3.476124269
114041	1.66201518	3.6761388	-0.352108471	3.818098858	4.482692559	3.153505157
114049	6.590838154	6.6952683	6.486407992	-0.352108471	-0.352108471	-0.352108471
114224	6.186998191	6.3713983	6.002598127	8.850902243	3.66408391	6.257493076
114299	8.82200586	6.1722592	3.522512573	8.769536334	6.463674915	4.157813496

114327	4.434314669	4.0914425	4.777186851	5.789762726	5.302571806	6.276953646
114609	6.335756628	4.1075772	5.221666912	3.629619582	3.76507088	3.900522179
114614	-0.352108471	-0.352108	-0.352108471	1.968661759	0.713037454	3.224286064
114625	6.332466669	6.89282	5.77211331	-0.352108471	-0.352108471	-0.352108471
114769	6.729557555	4.520764	5.625160771	-0.352108471	-0.352108471	-0.352108471
114781	6.161399353	3.1385599	4.649979619	1.086055948	1.731739118	0.440372779
114783	-0.352108471	-0.352108	-0.352108471	3.268770296	7.38266186	5.325716078
114785	4.181357699	5.4923767	4.83686721	6.025473287	4.869616713	7.18132986
114795	4.630899821	6.2144081	7.797916351	0.624614178	0.808855576	0.440372779
114803	5.088910439	8.4853577	6.787134073	-0.352108471	-0.352108471	-0.352108471
114804	7.146175474	7.2305473	7.061803645	5.760537073	5.775231583	5.745842563
114814	7.576136979	7.0545431	7.315340054	6.637716396	7.013354726	6.262078066
114819	7.497721025	8.2487146	6.746727467	6.126614759	7.759074157	6.942844458
114823	-0.352108471	-0.352108	-0.352108471	6.982834856	3.459527511	10.5061422
114825	7.07688723	5.564538	8.589236427	-0.352108471	-0.352108471	-0.352108471
114826	9.626192922	9.3702723	9.882113544	7.06010079	8.205103199	7.632601994
114836	3.020747404	4.7138991	6.407050724	-0.352108471	-0.352108471	-0.352108471
114879	5.821447986	5.6886226	5.555797308	4.29934907	6.751466056	9.203583042
114880	2.995934318	6.3439771	-0.352108471	3.119148782	3.119148782	3.119148782
114883	7.637794788	5.4937832	3.349771642	5.052267685	5.964683346	4.139852025
114897	5.774370319	3.5850742	7.963666421	3.970413419	8.681073916	6.325743667
114904	7.255201004	8.0881657	7.671683361	7.774346644	7.798259589	7.750433698
114907	6.195541118	6.2558778	6.135204448	-0.352108471	-0.352108471	-0.352108471
114908	7.600442205	8.0167969	7.808619573	6.705026474	6.449940077	6.577483275
114926	-0.352108471	-0.352108	-0.352108471	1.783698732	-0.352108471	3.919505936
114960	5.268658791	6.3519473	7.435235832	5.471958612	6.164674546	6.857390479
114984	6.674128879	6.7008502	6.647407551	6.165870606	5.76911462	6.562626592
114987	6.762900357	6.6644287	6.713664529	-0.352108471	-0.352108471	-0.352108471
114990	8.423332855	8.7242509	9.025168973	6.529110117	8.06432575	9.599541384
114991	8.714268537	8.9376253	8.490911731	7.618437404	4.927679215	2.236921026
115024	4.275651379	3.9195059	4.631796822	6.570974966	5.886988646	7.254961286
115098	-0.352108471	-0.352108	-0.352108471	8.0829707	7.054600764	6.026230829
115110	-0.352108471	-0.352108	-0.352108471	1.968661759	1.997577452	1.939746065
115196	8.559383458	8.748505	8.370261881	4.533402076	4.954218546	4.112585606
115265	-0.352108471	-0.352108	-0.352108471	5.095423988	5.124543452	5.066304524
115273	7.804017298	6.2663731	4.728728856	4.621417873	4.137528819	3.653639765
115290	6.55168719	5.4173924	5.984539772	7.937861295	6.572889217	5.207917138
115294	5.287831912	4.0981463	4.692989119	2.451290702	3.507061063	1.395520341
115352	-0.352108471	-0.352108	-0.352108471	4.505562533	7.754237547	6.12990004
115361	5.523369442	10.180203	7.851786454	8.817904547	4.246570244	6.532237396
115362	6.407695919	6.6215439	6.193847962	1.961524965	1.731739118	2.191310813
115399	4.58858854	5.895268	3.281909073	2.575541304	1.997577452	3.153505157
115416	-0.352108471	-0.352108	-0.352108471	4.66005769	4.268954283	5.051161096
115572	-0.352108471	-0.352108	-0.352108471	2.536362819	2.837303708	2.686833264
115704	6.927825301	7.0247388	7.121652232	5.983736712	6.002308672	5.965164752
115708	5.174316983	4.1788999	3.183482908	6.082070874	5.869665514	5.657260155
115727	6.463674915	6.7587503	6.168599573	-0.352108471	-0.352108471	-0.352108471

115752	7.389968954	7.844834	7.617401495	6.101908953	5.658147203	6.545670703
115761	2.179993378	4.7480446	7.316095872	1.493791735	2.438117353	0.549466117
115817	2.46950858	8.2398627	5.354685649	6.924275141	6.087644454	5.251013768
115825	4.947007189	4.9799919	4.914022459	-0.352108471	-0.352108471	-0.352108471
115908	9.219732269	6.8119539	4.40417554	4.200268952	4.927679215	5.655089478
115939	6.982793148	6.9037827	7.061803645	6.472605455	6.33538923	6.609821679
115992	-0.352108471	-0.352108	-0.352108471	1.079006084	1.90830372	2.737601355
116028	-0.352108471	-0.352108	-0.352108471	6.138118665	5.88700099	5.635883314
116039	0.882771258	6.7285574	3.805664353	2.419557138	5.191222747	-0.352108471
116068	6.020381091	6.0384535	6.002308672	6.382873477	6.410585269	6.355161685
116092	-0.352108471	-0.352108	-0.352108471	6.584450939	6.515031054	6.653870824
116113	6.729383037	5.8735024	7.585263678	6.003711401	7.550539586	6.777125493
116138	4.789355786	5.5282948	6.267233838	-0.352108471	-0.352108471	-0.352108471
116150	7.738876885	7.7464157	7.731338116	4.927679215	4.954218546	4.901139884
116151	7.181337851	5.7282969	4.275256032	0.616568244	4.637333234	8.658098224
116224	-0.352108471	-0.352108	-0.352108471	6.194240113	5.958359385	5.722478656
116225	6.000828803	5.2532408	4.505652781	3.978191052	5.637296679	7.296402306
116228	1.783698732	-0.352108	3.919505936	4.534956964	7.679280208	6.107118586
116236	6.846751508	5.3621074	6.104429468	3.319372359	4.286421842	3.8028971
116254	6.888413829	6.9598529	6.816974741	0.044132154	0.440372779	-0.352108471
116349	6.183964016	6.0089248	6.359003202	-0.352108471	-0.352108471	-0.352108471
116372	-0.352108471	-0.352108	-0.352108471	3.440349165	1.933317362	2.686833264
116448	5.833597119	5.6652071	5.749402114	-0.352108471	-0.352108471	-0.352108471
116461	-0.352108471	-0.352108	-0.352108471	5.779855963	4.558459688	5.169157826
116496	6.444919838	8.0880012	4.801838443	5.435979445	6.317926244	5.876952844
116535	-0.352108471	-0.352108	-0.352108471	3.554793223	5.494417177	7.43404113
116540	-0.352108471	-0.352108	-0.352108471	7.434031101	5.338202135	3.242373168
116541	4.471025746	6.6741289	8.877232011	6.017271115	8.438834653	7.228052884
116828	6.861057494	6.8767254	6.845389604	-0.352108471	-0.352108471	-0.352108471
116840	-0.352108471	-0.352108	-0.352108471	5.412561087	4.483586226	6.341535949
116844	7.429291064	7.5142953	7.3442868	6.962345725	6.202627216	5.442908708
116966	5.252571404	6.8661071	3.639035742	6.3775071	-0.982915775	2.697295663
116969	5.425512467	7.1221521	6.273832259	-0.352108471	-0.352108471	-0.352108471
116985	-0.352108471	-0.352108	-0.352108471	6.769641641	6.788420481	6.750862801
116988	7.379382859	4.1194214	5.749402114	7.543842494	8.087936625	7.81588956
117144	3.62216954	2.5886014	1.555033355	-0.352108471	-0.352108471	-0.352108471
117145	10.16062596	6.7689979	3.377369755	7.040807296	5.919349629	8.162264963
117177	6.570295729	4.1434958	5.356895761	6.816633855	4.902600883	5.859617369
117178	-0.352108471	-0.352108	-0.352108471	9.168109774	6.022050925	2.875992076
117195	5.245137737	5.5873575	5.929577197	5.516569456	5.748307778	5.632438617
117245	4.660293379	4.5885885	4.516883701	5.939192221	7.513583201	4.364801242
117246	-0.352108471	-0.352108	-0.352108471	3.114831108	5.821410838	8.527990568
117531	3.019155055	3.5193274	4.019499663	0.95875246	2.649848031	1.804300246
117581	-0.352108471	-0.352108	-0.352108471	5.663737322	5.665786693	5.661687951
118424	8.222532838	6.0956474	7.15909013	7.439787929	5.518732824	6.479260377
118429	-0.352108471	-0.352108	-0.352108471	6.087644454	8.090023534	4.085265375
118442	-0.352108471	-0.352108	-0.352108471	4.80208491	4.843308497	4.760861323

118460	8.399900192	7.4764785	6.553056862	2.97797424	3.649308576	2.306639904
118461	-0.352108471	-0.352108	-0.352108471	1.997577452	1.997577452	1.997577452
118472	-0.352108471	-0.352108	-0.352108471	3.580823089	4.80208491	6.023346731
118490	5.424058049	5.1245435	5.723572646	2.368970958	2.051108653	2.686833264
118611	-0.352108471	-0.352108	-0.352108471	6.285554459	6.31523113	6.255877787
118812	4.376528333	5.3889489	4.882738629	1.847863424	4.993022525	3.420442975
118924	6.438353249	7.0441776	5.832528937	6.443439905	6.813225323	6.073654487
118980	5.725558391	5.1529298	4.580301181	-2.883197702	2.030285487	6.943768677
118987	6.868723584	7.0238681	7.17901253	7.601341029	5.967751069	6.784546049
119392	4.223002093	4.5307426	3.915261593	5.226639187	4.213349141	3.200059094
119467	-0.352108471	-0.352108	-0.352108471	4.80208491	6.426981033	3.177188788
119504	9.033875044	7.2703879	10.79736214	6.360718632	9.456552772	7.908635702
119679	5.217491139	5.2437595	5.191222747	-0.352108471	-0.352108471	-0.352108471
119692	5.527756134	5.719565	5.911373854	-0.352108471	-0.352108471	-0.352108471
119749	4.248846646	5.1006122	5.95237771	-0.352108471	-0.352108471	-0.352108471
119764	-0.352108471	-0.352108	-0.352108471	5.325716078	5.336173319	5.315258837
120114	5.157610748	7.125009	3.190212489	4.297726199	3.615460478	4.979991919
120224	5.226167363	4.5971331	4.911650208	2.024343053	2.051108653	1.997577452
120425	-0.352108471	-0.352108	-0.352108471	5.564327261	5.045930461	4.52753366
120526	4.678491524	4.9841518	4.831321667	3.203655936	8.889907897	6.046781917
120787	-0.352108471	-0.352108	-0.352108471	5.000355532	5.118656602	5.236957671
120892	6.891019964	4.5776666	9.204373279	7.247844574	6.288551791	8.207137358
121006	7.585015974	6.1215288	4.658041564	-0.352108471	-0.352108471	-0.352108471
121256	-0.352108471	-0.352108	-0.352108471	5.663737322	3.454608303	7.87286634
121260	-0.352108471	-0.352108	-0.352108471	5.233567424	6.041107527	4.42602732
121274	-0.352108471	-0.352108	-0.352108471	4.606385815	5.529311366	6.452236916
121441	10.10188767	3.67494	6.888413829	1.114208477	1.880238567	2.646268658
121456	4.583433279	5.054281	4.112585606	0.440372779	0.440372779	0.440372779
121457	4.75203451	6.1348006	7.517566721	5.831882629	7.098774477	8.365666325
121512	6.799519626	6.5884653	7.010573985	3.753284825	2.191310813	5.315258837
121551	7.758936954	4.9888483	2.218759731	4.456886199	3.993367271	4.920405128
121599	1.783698732	1.0981297	2.469267744	0.874413692	2.100935855	-0.352108471
121601	3.134717702	6.6215439	-0.352108471	5.436203094	4.754644882	5.095423988
121665	6.917345136	6.0592292	6.488287179	6.38229808	7.612704881	8.843111683
122416	-0.352108471	-0.352108	-0.352108471	6.392584054	6.396868383	6.401152711
122704	5.826911535	6.9393943	8.051877111	1.448328272	1.598101998	1.747875723
122769	7.503777584	2.9312047	5.217491139	6.631846251	3.935625393	9.32806711
122773	-0.352108471	-0.352108	-0.352108471	3.607604587	4.911650208	6.21569583
122786	7.586845637	6.183964	4.781082394	6.616491993	6.266367194	5.916242394
122953	6.332466669	6.4048935	6.260039847	-0.352108471	-0.352108471	-0.352108471
123041	7.163447031	8.7374215	10.31139595	3.80284622	10.02217215	6.912509186
123096	6.212536209	8.4620038	3.963068578	-0.352108471	-0.352108471	-0.352108471
123207	6.281347898	6.1414231	6.421272681	3.761755053	1.807952763	5.715557343
123228	5.625160771	4.1907334	7.059588108	-0.352108471	-0.352108471	-0.352108471
123263	9.671746895	8.8211847	7.970622429	6.662402318	7.334312635	6.998357476
123283	4.382026917	7.4963575	5.939192221	-0.352108471	-0.352108471	-0.352108471
123606	-0.352108471	-0.352108	-0.352108471	7.28018736	7.003799972	6.727412585

123720	6.555519439	6.7952996	6.315739249	7.329601031	7.702446957	6.956755105
123722	8.056217068	7.6520774	7.854147238	6.316353013	5.939497163	5.562641312
123745	6.327522603	8.6061228	7.46682272	1.158617076	1.876861374	0.440372779
123811	4.821539291	3.8306378	5.812440747	0.939116071	0.440372779	1.437859363
123879	3.00530001	7.8514877	5.428393874	0.044132154	-0.352108471	0.440372779
123920	-0.352108471	-0.352108	-0.352108471	6.6560422	6.674249904	6.637834496
124044	4.901927854	6.1348006	7.367673376	0.044132154	0.440372779	-0.352108471
124149	5.800199788	5.825168	5.775231583	-0.352108471	-0.352108471	-0.352108471
124152	4.942532363	6.0450341	5.493783215	5.807345806	4.450744817	3.094143827
124221	2.205662717	8.2293196	5.217491139	-0.352108471	-0.352108471	-0.352108471
124245	-0.352108471	-0.352108	-0.352108471	6.080278611	5.825167992	6.33538923
124359	5.980215154	5.3269274	4.673639664	-0.352108471	-0.352108471	-0.352108471
124402	5.028294363	5.8529375	6.67758056	7.768999313	5.541320776	3.313642238
124446	6.720370627	5.9547106	7.486030625	-0.352108471	-0.352108471	-0.352108471
124491	10.60070969	6.7485569	2.896404121	6.295505162	5.417314844	5.856410003
124583	-0.352108471	-0.352108	-0.352108471	9.378282549	4.040681465	6.709482007
124739	6.303461245	5.1625733	7.444349219	-0.352108471	-0.352108471	-0.352108471
124773	-0.352108471	-0.352108	-0.352108471	5.86831314	3.375240742	4.621776941
124801	2.794181776	2.280957	1.767732212	4.105630685	9.136705405	6.621168045
124808	-0.352108471	-0.352108	-0.352108471	5.924907944	7.317428146	6.621168045
124842	-0.352108471	-0.352108	-0.352108471	5.55542532	6.532441983	7.509458647
124857	-0.352108471	-0.352108	-0.352108471	7.696187302	5.983736712	4.271286122
124923	2.670466985	4.4868934	6.303319902	3.77278635	3.953434078	4.134081806
124935	8.163200626	10.705685	5.620716389	5.325565994	10.64716998	7.986367989
124961	5.152247113	5.1799508	5.124543452	-0.352108471	-0.352108471	-0.352108471
124975	7.738685842	5.9874902	4.236294566	7.610738001	5.769760533	6.690249267
124995	6.677917073	5.5916842	6.134800615	2.335166442	2.536084801	2.737003159
125050	6.120953226	6.278238	6.199595632	-0.352108471	-0.352108471	-0.352108471
125061	7.962002853	7.3176651	6.673327363	5.607838856	5.798039202	5.702939029
125144	-0.352108471	-0.352108	-0.352108471	7.394190584	10.98355153	3.804829637
125150	7.141642983	5.7429144	4.344185765	1.864658285	-0.017926954	3.747243524
125476	-0.352108471	-0.352108	-0.352108471	5.821410838	6.153574972	5.489246703
125919	8.335688791	7.5180702	6.700451683	7.217174762	6.680596033	6.144017305
125931	-0.352108471	-0.352108	-0.352108471	5.528678444	3.899165999	4.713922221
125950	4.666721742	6.190066	5.428393874	5.292713606	6.751388245	6.022050925
125958	5.983728392	6.6231941	6.303461245	-0.352108471	-0.352108471	-0.352108471
125965	7.192332117	5.599099	4.005865821	4.5986471	5.599602802	5.099124951
125981	6.415311207	5.1903345	7.640287881	1.245740716	0.440372779	2.051108653
126003	2.905418398	4.1248641	3.515141236	8.87755094	5.578623452	7.228087196
126017	-0.352108471	-0.352108	-0.352108471	2.501526031	-0.352108471	5.355160532
126069	4.476787422	5.1914165	4.834101937	1.394345699	-0.361039605	3.149731003
126070	6.708309831	5.6067887	4.505267587	3.591481705	3.79021001	3.988938314
126074	5.954590303	5.5882244	6.320956231	3.87211091	2.051108653	5.693113166
126129	-0.352108471	-0.352108	-0.352108471	3.490670316	5.494417177	7.498164037
126133	5.790856505	5.8744121	5.707300866	3.864985883	4.72458994	5.584193997
126147	6.912042523	5.3043679	8.519717113	2.166656576	1.646479888	2.686833264
126204	-0.352108471	-0.352108	-0.352108471	8.419881835	5.55876787	2.697653905

126205	5.64625513	5.5591194	5.733390872	1.895859924	1.141109877	1.518484901
126208	-0.352108471	-0.352108	-0.352108471	5.738003302	5.284974356	4.83194541
126259	3.564438277	4.3359073	5.107376412	0.440372779	0.440372779	0.440372779
126298	-0.352108471	-0.352108	-0.352108471	1.783698732	1.928763824	1.638633641
126299	-0.352108471	-0.352108	-0.352108471	3.717018359	6.217192107	4.967105233
126308	6.670900682	5.476238	4.281575288	7.090285804	7.521974665	7.953663525
126321	5.31909676	2.610908	8.027285488	7.811863603	6.190074859	7.000969231
126328	7.791755961	7.8693257	7.714186196	6.769641641	6.788420481	6.750862801
126364	3.445762001	6.2806901	4.863226045	1.394345699	1.14221151	1.646479888
126374	6.121528769	3.9213219	8.321735645	-0.352108471	-0.352108471	-0.352108471
126393	9.141419653	6.6846231	7.913021367	7.725479856	9.68416352	8.704821688
126433	6.570570825	4.7680391	8.373102544	6.774861833	6.775911759	6.775386796
126549	6.783301918	6.8767254	6.689878453	4.899465632	3.337746555	6.461184708
126661	5.242983992	5.9831939	5.613088937	1.036211897	1.14221151	0.930212283
126669	10.62416941	4.8364324	7.730300925	0.395051519	1.14221151	-0.352108471
126731	6.695975478	4.0133958	5.354685649	5.923493182	3.694651085	8.152335279
126792	4.550307802	5.4196962	3.680919435	3.588210414	6.878924433	5.233567424
126868	4.286259633	6.7013068	5.493783215	-0.352108471	-0.352108471	-0.352108471
126917	6.382873477	6.3791753	6.386571668	-0.352108471	-0.352108471	-0.352108471
127002	-0.352108471	-0.352108	-0.352108471	6.056053999	6.073654487	6.038453511
127253	-0.352108471	-0.352108	-0.352108471	4.701167306	6.001809131	7.302450957
127254	-0.352108471	-0.352108	-0.352108471	4.358033276	5.760537073	7.16304087
127262	-0.352108471	-0.352108	-0.352108471	7.003799972	7.016299086	6.991300859
127281	5.455547246	5.1826463	5.31909676	2.881913056	2.179939357	1.477965658
127294	6.247578166	5.7072189	5.97739855	1.085571587	5.249226858	3.167399223
127428	4.549728355	5.1799508	3.919505936	3.241094247	2.051108653	4.43107984
127435	5.183015302	4.8512576	5.017136436	2.289360373	2.274515706	2.25967104
127495	2.764225616	3.7080885	1.820362695	-0.352108471	-0.352108471	-0.352108471
127544	6.854454372	9.0781625	7.966308413	6.12990004	7.325589341	4.93421074
127687	3.076450934	3.9622038	3.519327359	2.076022254	2.100935855	2.051108653
127703	6.469314697	7.6046979	5.333931507	5.961166865	7.003730975	4.918602756
127731	-0.352108471	-0.352108	-0.352108471	1.577677026	2.633343959	3.689010892
127829	7.134347894	6.0203811	4.906414289	8.527189358	6.297476756	7.412333057
127933	6.674128879	9.2089668	4.139290942	5.535358938	4.549849375	6.520868501
128077	9.181761489	9.1862683	9.177254692	6.728640909	6.497336045	6.959945773
128102	5.868265759	5.3152588	6.421272681	6.504868974	6.588465267	6.421272681
128178	4.942353437	4.8304259	5.054280952	3.80092067	4.482692559	3.119148782
128240	5.249862356	5.4595089	5.354685649	4.764422158	6.296043865	7.827665573
128272	-0.352108471	-0.352108	-0.352108471	4.559699198	6.307285814	2.812112583
128371	-0.352108471	-0.352108	-0.352108471	3.261546676	3.919505936	4.577465195
128387	9.078698995	7.294256	10.86314203	6.881186765	8.231814137	9.582441509
128553	3.164602038	6.7781556	4.97137884	5.079283667	7.216263253	2.942304082
128637	4.636275217	8.784884	6.710579627	5.422285101	5.529311366	5.315258837
128674	4.298298976	5.9222488	5.1102739	0.476561106	-0.352108471	1.305230684
128861	-0.352108471	-0.352108	-0.352108471	5.307880044	5.852863847	6.39784765
128866	8.383860249	7.4912415	6.59862276	10.74185558	7.721463244	4.701070904
128869	-0.352108471	-0.352108	-0.352108471	1.059179699	5.179117864	3.119148782

128872	5.436925628	5.9583273	6.479729071	5.667033469	5.517905788	5.816161149
128876	6.245901837	7.0074534	5.484350273	-0.352108471	-0.352108471	-0.352108471
128954	-0.352108471	-0.352108	-0.352108471	1.492819042	3.337746555	-0.352108471
128977	-0.352108471	-0.352108	-0.352108471	6.463674915	6.916156778	6.011193051
128989	5.974072495	5.4694396	4.96480678	6.994280132	8.823759625	5.164800639
129049	8.034340119	6.1096989	9.958981353	8.325954428	6.449118953	7.387536691
129138	-0.352108471	-0.352108	-0.352108471	5.096091601	6.312578908	5.704335255
129401	6.346727397	5.4937832	4.640839033	-0.352108471	-0.352108471	-0.352108471
129446	5.428393874	5.4582551	5.398532684	-0.352108471	-0.352108471	-0.352108471
129531	6.073207852	7.2978942	4.848521532	0.975533543	1.14221151	0.808855576
129563	5.301899295	5.7494021	6.196904934	-0.352108471	-0.352108471	-0.352108471
129684	-0.352108471	-0.352108	-0.352108471	3.919505936	3.919505936	3.919505936
129685	8.447321071	8.4654461	8.429196032	6.180197528	7.295056154	8.409914779
129787	-0.352108471	-0.352108	-0.352108471	6.262794607	6.668713086	6.465753847
129804	-0.352108471	-0.352108	-0.352108471	5.791816861	3.585121301	1.378425741
129831	5.247322627	5.3920614	5.53680009	-0.352108471	-0.352108471	-0.352108471
129852	-0.352108471	-0.352108	-0.352108471	5.329241098	6.810846835	6.070043966
129868	2.41236255	2.4154798	2.413921152	-0.352108471	-0.352108471	-0.352108471
129880	10.68066338	11.797486	9.563841193	8.066020999	9.302845624	10.53967025
130026	9.052242903	9.0028661	8.953489292	7.768534579	8.02211051	7.514958648
130074	-0.352108471	-0.352108	-0.352108471	6.406486527	6.6742236	6.138749454
130367	6.022844903	8.3873256	7.205085245	0.975533543	0.808855576	1.14221151
130535	-0.352108471	-0.352108	-0.352108471	5.718459767	3.905932344	7.530987189
130617	5.592623053	5.0306439	6.15460216	7.974042457	6.903642175	5.833241892
130749	5.354685649	3.3264552	7.382916079	-0.352108471	-0.352108471	-0.352108471
130813	4.446433087	4.7923037	5.138174251	-0.352108471	-0.352108471	-0.352108471
130940	7.376378972	5.2885544	6.332466669	-0.352108471	-0.352108471	-0.352108471
130951	2.774371847	2.6547897	2.535207633	-0.352108471	-0.352108471	-0.352108471
131076	-0.352108471	-0.352108	-0.352108471	7.363054506	6.022050925	4.681047344
131408	-0.352108471	-0.352108	-0.352108471	6.12990004	6.135204448	6.124595632
131474	-0.352108471	-0.352108	-0.352108471	2.138198525	8.052649451	5.095423988
131566	-0.352108471	-0.352108	-0.352108471	8.487311391	7.690758392	6.894205393
131583	-0.352108471	-0.352108	-0.352108471	6.643323087	6.876426316	7.109529545
131601	5.528242089	8.0192395	6.77374078	-0.352108471	-0.352108471	-0.352108471
131616	4.84493126	8.0217062	6.433318728	-0.352108471	-0.352108471	-0.352108471
131669	-0.352108471	-0.352108	-0.352108471	5.821411338	4.872224191	3.923037043
131909	4.948794725	3.1433352	6.754254219	1.540978788	1.939746065	1.14221151
131965	-0.352108471	-0.352108	-0.352108471	6.664356519	4.487610934	5.575983727
132014	6.380626467	7.2531836	6.816905042	1.90830372	1.939746065	1.876861374
132320	-0.352108471	-0.352108	-0.352108471	6.316732863	5.095423988	3.874115113
132332	-0.352108471	-0.352108	-0.352108471	6.766572321	2.147200078	4.456886199
132660	3.234152935	5.062887	6.891621053	3.83226546	4.64805297	5.46384048
133060	6.058027712	4.5801658	5.31909676	-0.352108471	-0.352108471	-0.352108471
133686	6.168090483	6.1079622	6.228218771	3.314541055	5.191222747	1.437859363
134147	8.017253217	5.7437985	10.29070797	8.054446927	5.893263659	6.973855293
134218	5.766929757	4.6198666	6.913992945	4.666773335	1.605880603	3.136326969
134265	4.683876778	5.3910182	6.098159551	7.36272357	5.651048706	6.506886138

134266	-0.352108471	-0.352108	-0.352108471	4.501651414	4.412120985	4.456886199
134353	7.656966275	6.5318751	8.78205747	3.550878841	4.954218546	2.147539136
134510	6.7330713	3.4457531	5.089412202	-0.352108471	-0.352108471	-0.352108471
134526	0.211262559	5.1602016	2.685732087	-0.352108471	-0.352108471	-0.352108471
134549	8.713120087	11.533853	5.892387454	7.581912294	6.413774262	8.750050325
134553	7.289605497	7.88829	8.486974535	9.834760824	6.664128189	3.493495553
135112	1.783698732	-0.352108	3.919505936	4.80208491	4.843308497	4.760861323
135114	5.915852686	6.5705196	5.261185764	6.106386272	5.653299811	6.559472732
135154	-0.352108471	-0.352108	-0.352108471	7.149285497	5.182455715	6.165870606
135228	6.208889547	5.4273255	5.818107534	1.804300246	1.731739118	1.876861374
135293	4.659953204	5.3565025	5.008227864	1.154753083	1.475855286	1.796957489
136647	-0.352108471	-0.352108	-0.352108471	5.369332089	2.046130426	8.692533751
137362	-0.352108471	-0.352108	-0.352108471	2.451290702	5.254689875	-0.352108471
137392	-0.352108471	-0.352108	-0.352108471	6.296043865	6.769499241	5.82258849
137492	-0.352108471	-0.352108	-0.352108471	5.681727979	5.775231583	5.588224375
137964	1.25802751	9.983755	5.620891261	6.443033625	7.355470506	5.530596744
138241	6.008863898	6.1690597	5.848668051	0.044132154	-0.352108471	0.440372779
138428	-0.352108471	-0.352108	-0.352108471	5.555799529	7.651301488	6.603550508
138474	4.663000528	5.9981911	3.327809991	4.197748308	5.993099668	5.095423988
138639	-0.352108471	-0.352108	-0.352108471	6.204656258	5.470769864	6.938542652
138649	-0.352108471	-0.352108	-0.352108471	5.404055244	5.971144873	5.687600059
138716	-0.352108471	-0.352108	-0.352108471	5.494417177	6.140582551	4.848251802
138724	5.800199788	5.825168	5.775231583	-0.352108471	-0.352108471	-0.352108471
138948	4.637201132	2.8431725	1.049143908	-0.352108471	-0.352108471	-0.352108471
139081	-0.352108471	-0.352108	-0.352108471	0.088697018	4.190991333	2.139844176
139221	7.450540243	6.816905	6.183269842	-0.352108471	-0.352108471	-0.352108471
139422	6.071865894	1.488281	10.65545079	0.968206476	2.58964242	1.778924448
139538	4.591035201	5.1522471	5.713459025	-0.352108471	-0.352108471	-0.352108471
139562	6.903083627	7.8346426	7.368863092	6.646279159	6.224523211	7.068035106
139716	1.3830066	2.2456	3.108193427	-0.352108471	-0.352108471	-0.352108471
140453	6.695281831	5.7888147	6.242048279	3.605011212	1.997577452	5.212444972
140459	10.06737176	4.6703544	7.368863092	6.32531018	7.42133441	5.22928595
140460	2.249866357	6.618763	4.434314669	6.191249109	4.442775673	5.317012391
140461	7.451946507	7.4644235	7.476900573	-0.170547908	4.044986734	1.937219413
140465	-0.352108471	-0.352108	-0.352108471	4.941127012	5.526007836	5.233567424
140467	5.624683414	5.7494021	5.874120815	6.446390821	5.933596618	5.420802415
140469	0.985314745	6.7547657	3.870040209	4.80208491	4.734528909	4.869640911
140564	5.889602147	6.0160069	6.142411725	5.479873401	4.089954539	6.869792262
140576	2.129081329	6.9715343	4.550307802	7.9014325	5.411225172	10.39163983
140597	4.222735929	5.9560885	5.089412202	-0.352108471	-0.352108471	-0.352108471
140606	5.998406448	6.9702207	5.026592214	8.267464527	7.854376094	7.441287662
140609	-0.352108471	-0.352108	-0.352108471	5.077701691	6.641533047	5.859617369
140685	-0.352108471	-0.352108	-0.352108471	6.459304363	6.497336045	6.421272681
140688	7.081937271	5.0301707	6.056053999	-0.352108471	-0.352108471	-0.352108471
140690	4.576692604	6.6592049	8.741717178	3.772762915	3.630416192	3.915109637
140700	4.947007189	4.9799919	4.914022459	-0.352108471	-0.352108471	-0.352108471
140710	-0.352108471	-0.352108	-0.352108471	3.782132804	2.124237496	0.466342188

140711	-0.306239829	1.8802386	4.066716964	5.683164102	-0.031919049	2.825622527
140739	6.090808341	4.2703392	7.91127753	-0.352108471	-0.352108471	-0.352108471
140775	7.743278113	7.9408287	7.545727567	6.608518847	6.987922423	6.229115271
140801	-0.352108471	-0.352108	-0.352108471	5.222956311	5.191222747	5.254689875
140803	6.726177317	6.1183043	7.334050331	5.074415874	3.16927636	4.121846117
140809	5.284974356	4.6832997	5.886648965	-0.352108471	-0.352108471	-0.352108471
140823	-0.352108471	-0.352108	-0.352108471	6.479260377	6.497336045	6.461184708
140883	5.334561398	5.6884362	6.042311058	0.440372779	0.440372779	0.440372779
140885	6.502263028	5.7295947	7.27493133	9.11829842	7.087030751	5.055763083
140890	6.400592293	6.7737408	7.146889267	-0.352108471	-0.352108471	-0.352108471
140901	-0.352108471	-0.352108	-0.352108471	7.494097175	6.266367194	5.038637212
140947	-0.352108471	-0.352108	-0.352108471	5.663737322	5.665786693	5.661687951
142678	6.415928975	7.1261077	5.705750269	-0.352108471	-0.352108471	-0.352108471
142679	4.755240115	3.7841621	5.726318086	4.178899946	4.245214285	4.112585606
142913	5.955089042	6.0276148	6.100140465	1.76984594	1.731739118	1.807952763
143241	5.824288785	5.873346	5.775231583	1.697414861	1.14221151	2.252618212
143244	3.194903159	6.8851459	5.040024535	2.387802959	2.597822563	2.177783356
143384	5.748354287	4.7951934	6.701515223	7.599776093	5.73299091	3.866205727
143471	-0.352108471	-0.352108	-0.352108471	7.780424202	1.975916071	4.878170137
143502	-0.352108471	-0.352108	-0.352108471	2.039000386	7.981522684	5.010261535
143872	5.840951908	7.40613	6.623540933	-0.352108471	-0.352108471	-0.352108471
143888	7.233640376	5.6710216	8.796259121	6.926801914	9.543416538	4.310187289
144097	-0.352108471	-0.352108	-0.352108471	6.038453511	3.912822889	8.164084133
144108	3.127737875	3.136327	3.119148782	4.08236011	4.245214285	3.919505936
144195	6.550874147	6.5688561	6.586838152	2.281094784	3.563765528	4.846436272
144233	6.065196634	7.0432293	5.087164007	6.382429697	6.969571564	5.79528783
144245	5.519415741	5.3152588	5.723572646	2.710182349	0.440372779	4.979991919
144348	7.233090752	5.3444745	6.288782637	6.415928975	8.334763435	4.497094514
144363	-0.352108471	-0.352108	-0.352108471	7.255355713	6.369156591	5.482957468
144404	7.857408401	5.8110875	9.903729252	6.881056107	7.354219327	7.117637717
144717	9.711076608	4.8615727	7.286324647	-0.352108471	-0.352108471	-0.352108471
144776	2.51010775	5.012156	3.761131874	0.849500091	-0.352108471	2.051108653
144817	3.154631683	6.8796412	5.017136436	2.929626001	5.501488496	0.357763505
145200	5.678446034	4.2557691	7.101122942	-0.352108471	-0.352108471	-0.352108471
145216	4.579618372	4.9140225	4.245214285	0.808855576	0.808855576	0.808855576
145389	-0.352108471	-0.352108	-0.352108471	5.091385644	6.235921161	7.380456677
145438	7.065444921	6.9268019	7.204087928	1.954444309	1.807952763	2.100935855
145482	4.58858854	3.2578236	5.91935344	4.059833757	7.490629409	5.775231583
145483	1.501017585	-0.352108	3.354143641	1.864658285	1.997577452	1.731739118
145501	6.067920556	4.6414507	5.354685649	5.940426725	5.468243785	5.704335255
145567	-0.352108471	-0.352108	-0.352108471	3.585121301	3.615460478	3.554782125
145624	5.152247113	3.6399979	6.664496376	-0.352108471	-0.352108471	-0.352108471
145820	8.318263603	6.9808439	5.643424221	3.526802152	3.331827732	3.136853312
145864	2.280956994	-0.352108	4.914022459	-0.352108471	-0.352108471	-0.352108471
146050	5.94941124	4.8247903	7.074032224	9.589949567	6.374837172	3.159724777
146059	4.538083686	6.033597	7.529110263	0.044132154	0.440372779	-0.352108471
146177	3.134671562	0.7584343	1.946552907	3.203220169	2.564345668	1.925471168

146198	-0.352108471	-0.352108	-0.352108471	1.968661759	1.997577452	1.939746065
146336	-0.352108471	-0.352108	-0.352108471	6.186562848	6.84173007	5.531395627
146429	-0.352108471	-0.352108	-0.352108471	5.287882532	2.139844176	-1.008194181
146513	6.311614365	6.6415447	6.971475131	0.395051519	-0.352108471	1.14221151
146540	6.851596565	6.8335853	6.842590929	5.747319767	4.79273235	6.701907184
146547	4.947007189	5.0454191	4.848595312	-0.352108471	-0.352108471	-0.352108471
146664	5.50073427	4.6780901	5.089412202	4.80208491	5.99616903	3.60800079
146723	4.905873799	6.798825	3.012922619	5.825702225	6.927423116	4.723981333
146771	5.595499658	6.90247	4.288529348	1.12335747	0.808855576	1.437859363
146850	-0.352108471	-0.352108	-0.352108471	6.056053999	6.038453511	6.073654487
146857	4.800484379	6.0209415	7.24139857	3.055408374	3.919505936	2.191310813
146909	4.677837508	6.9720906	9.266343627	8.068950007	4.484680012	6.276815009
146923	7.773581937	7.3867697	7.580175832	6.465742382	6.764980292	7.064218202
147007	6.607377459	6.6215439	6.593211042	0.228373553	0.808855576	-0.352108471
147081	7.396680528	6.5596	8.23376105	3.599727888	3.655785158	3.627756523
147166	4.072308319	5.3191029	6.56589752	2.383338121	1.305230684	0.227123247
147172	6.184820943	7.5463696	4.823272238	2.219830878	3.587895627	4.955960376
147179	3.979545943	3.8009207	3.622295397	3.316275833	0.461843501	1.889059667
147339	6.831289207	8.2580747	5.404503681	6.270222109	4.905740415	7.634703803
147372	6.648260902	6.479333	6.817188811	6.283292661	6.720696571	5.845888751
147381	-0.352108471	-0.352108	-0.352108471	5.658932548	6.600867533	6.12990004
147407	-0.352108471	-0.352108	-0.352108471	1.964912095	2.187132414	2.076022254
147409	6.640019393	6.6538708	6.626167961	-0.352108471	-0.352108471	-0.352108471
147429	5.074091507	5.6724599	5.373275698	5.203525788	2.740352399	7.666699177
147650	5.411322995	6.2945519	5.852937462	-0.352108471	-0.352108471	-0.352108471
147664	7.900064474	10.478524	5.321604731	7.547958879	6.204236666	4.860514453
147670	7.101746133	8.6650577	7.883401897	6.567717306	7.183612904	5.951821708
147694	9.421995861	6.981366	8.201680952	6.651348031	5.281331678	8.021364385
147699	5.320563245	5.3152588	5.325867653	-0.352108471	-0.352108471	-0.352108471
147700	-0.352108471	-0.352108	-0.352108471	2.065292044	4.482692559	-0.352108471
147710	5.708627399	7.3307205	6.519673958	-0.352108471	-0.352108471	-0.352108471
147727	7.4871992	5.3083583	9.666040122	1.804300246	1.731739118	1.876861374
147804	3.127737875	3.2887997	2.966676046	7.847425112	7.840015574	7.843720343
147912	-0.352108471	-0.352108	-0.352108471	2.578370801	9.300013642	5.939192221
147945	2.803125457	5.9583594	-0.352108471	-0.352108471	-0.352108471	-0.352108471
147947	7.142706794	3.5760851	10.70932853	2.942215415	3.270217129	2.614213701
147991	0.844169974	2.6547897	4.465409505	-0.352108471	-0.352108471	-0.352108471
148014	-0.352108471	-0.352108	-0.352108471	5.083930458	4.559699198	4.035467939
148022	6.134800615	4.9987049	7.270896306	-0.352108471	-0.352108471	-0.352108471
148189	6.2088898	4.9042312	3.599572568	1.689236102	1.668440768	1.678838435
148203	8.617557198	7.084019	5.550480743	8.243469871	5.214701252	6.729085562
148206	6.321182753	7.1484892	7.975795696	8.113774824	6.733189133	7.423481978
148213	6.956000881	8.3624412	5.549560577	6.00432218	6.479332994	5.529311366
148223	-0.352108471	-0.352108	-0.352108471	4.570594918	3.787204974	4.178899946
148266	6.028048365	6.4636749	6.899301464	-0.352108471	-0.352108471	-0.352108471
148281	5.749402114	5.3810435	6.117760751	-0.352108471	-0.352108471	-0.352108471
148304	-0.352108471	-0.352108	-0.352108471	1.798779064	1.968661759	2.138544454

148327	-0.352108471	-0.352108	-0.352108471	5.017136436	6.206979421	3.82729345
148345	5.017136436	7.5844188	2.449854073	-0.352108471	-0.352108471	-0.352108471
148413	-0.352108471	-0.352108	-0.352108471	5.404988946	2.501526031	-0.401936885
148423	-0.352108471	-0.352108	-0.352108471	7.676488118	6.671874638	5.667261159
148534	4.537820104	7.7700895	1.305550693	5.938433002	5.669105462	6.207760543
148696	5.749402114	4.8085145	6.690289712	-0.352108471	-0.352108471	-0.352108471
148709	5.601694565	4.4826926	6.720696571	0.975533543	0.808855576	1.14221151
148808	7.404415956	2.5555679	4.979991919	0.791292145	0.440372779	1.14221151
148870	5.360267056	7.1358985	3.584635578	-0.352108471	-0.352108471	-0.352108471
148932	5.800199788	5.825168	5.775231583	-0.352108471	-0.352108471	-0.352108471
148979	-0.352108471	-0.352108	-0.352108471	4.456886199	4.482692559	4.43107984
149013	-0.352108471	-0.352108	-0.352108471	5.169157826	5.770339486	4.567976165
149018	6.02529475	5.2250268	5.625160771	-0.352108471	-0.352108471	-0.352108471
149041	1.383520155	-0.352108	3.119148782	1.995427359	2.051108653	1.939746065
149345	-0.352108471	-0.352108	-0.352108471	5.325716078	3.144733859	7.506698297
149428	4.694839979	4.8304259	4.559254036	6.745152057	6.880482436	6.609821679
149461	2.58480451	4.4081831	6.231561712	1.374300821	0.808855576	1.939746065
149466	6.994540798	5.8217989	4.649056906	1.429982115	2.051108653	0.808855576
149483	3.51606145	7.3407263	5.428393874	-0.352108471	-0.352108471	-0.352108471
149499	4.847368759	3.9195059	5.775231583	-0.352108471	-0.352108471	-0.352108471
149603	-0.352108471	-0.352108	-0.352108471	4.404434355	8.464855162	6.434644758
149620	5.284974356	5.4023386	5.167610084	0.228373553	0.808855576	-0.352108471
149992	6.974267616	7.1980192	7.421770869	2.064739793	2.252618212	1.876861374
150084	-0.352108471	-0.352108	-0.352108471	5.958359385	4.884439554	7.032279215
150159	-0.352108471	-0.352108	-0.352108471	2.38621749	5.124543452	-0.352108471
150223	-0.352108471	-0.352108	-0.352108471	5.165235283	2.575878933	3.870557108
150244	4.886860718	4.7306158	4.574370823	4.780236561	3.963449484	5.597023638
150274	6.619197037	4.6913033	8.547090744	0.95025487	-0.352108471	2.252618212
150291	-0.352108471	-0.352108	-0.352108471	5.255958484	5.393523004	5.531087525
150353	-0.352108471	-0.352108	-0.352108471	5.541320776	5.588224375	5.494417177
150368	7.052977603	6.2952802	6.674128879	-0.352108471	-0.352108471	-0.352108471
150381	6.056053999	7.5810502	4.531057775	-0.352108471	-0.352108471	-0.352108471
150465	7.886413181	4.3566444	6.121528769	5.284974356	6.317532563	4.25241615
150468	-0.352108471	-0.352108	-0.352108471	6.799187547	6.204656258	5.610124969
150572	-0.352108471	-0.352108	-0.352108471	6.591990649	5.688622647	4.785254645
150622	4.624859933	5.2312478	4.018472052	1.568700478	3.524550744	2.546625611
150677	9.036665081	3.0392981	6.03798158	-0.352108471	-0.352108471	-0.352108471
150696	4.406199352	4.3158285	4.496570221	3.635664202	5.284336357	6.933008513
150759	7.288294839	7.1689345	7.407655215	4.188896228	2.100935855	6.276856601
150864	6.454033016	7.5997155	5.308350525	3.676368992	3.542556211	3.810181774
150921	6.055676819	6.4333187	6.810960637	-0.352108471	-0.352108471	-0.352108471
150935	2.103572783	-0.229174	4.436319997	2.553073296	3.041952253	2.06419434
150962	5.493783215	3.4066297	7.580936746	-0.352108471	-0.352108471	-0.352108471
151009	6.556689902	7.8278522	5.285527604	3.166717128	5.631148531	0.702285726
151056	-0.352108471	-0.352108	-0.352108471	6.64789689	6.674249904	6.621543876
151121	5.284974356	5.3152588	5.254689875	-0.352108471	-0.352108471	-0.352108471
151174	-0.352108471	-0.352108	-0.352108471	4.66005769	4.688318557	4.631796822

151194	8.118093872	7.3233064	6.52851883	7.02826092	6.986144846	7.007202883
151234	5.478536194	5.680265	5.276807358	-0.352108471	-0.352108471	-0.352108471
151246	6.016188175	6.5314763	6.273832259	2.579251289	3.497013509	4.414775729
151258	4.702869762	5.2647065	5.826543237	-0.352108471	-0.352108471	-0.352108471
151306	2.777872975	6.8067344	4.792303669	-0.352108471	-0.352108471	-0.352108471
151354	8.526500833	5.8001998	3.073898742	5.958359385	5.311201928	6.605516841
151475	7.451903952	9.4148928	5.488915059	1.483450039	4.24400866	2.863729349
151477	4.680131247	3.1277379	1.575344504	1.90117116	1.549724107	2.252618212
151636	9.168791693	8.06044	6.952088238	7.312834771	9.003450504	5.622219039
151887	-0.352108471	-0.352108	-0.352108471	4.668754626	7.604813664	10.5408727
151963	-0.352108471	-0.352108	-0.352108471	5.770362668	5.825167992	5.715557343
152002	-0.352108471	-0.352108	-0.352108471	4.178899946	3.11672189	5.241078001
152006	-0.352108471	-0.352108	-0.352108471	3.119148782	3.119148782	3.119148782
152007	-0.352108471	-0.352108	-0.352108471	2.252618212	3.878445392	0.626791031
152098	2.201036472	4.7541814	-0.352108471	1.383520155	3.119148782	-0.352108471
152137	-0.352108471	-0.352108	-0.352108471	3.612309474	5.820804688	4.716557081
152217	-0.352108471	-0.352108	-0.352108471	6.866733042	7.039536769	6.693929315
152404	5.518872571	6.5373837	7.555894878	-0.352108471	-0.352108471	-0.352108471
152485	4.878665478	3.9914912	5.765839755	9.563901774	2.283084591	5.923493182
152503	-0.352108471	-0.352108	-0.352108471	5.090388781	5.55876787	6.027146959
152519	6.519972795	6.4919329	6.54801273	4.573177085	4.245214285	4.901139884
152579	-0.352108471	-0.352108	-0.352108471	8.995722529	6.396868383	3.798014236
152641	6.537383725	7.5683905	5.506376915	-0.352108471	-0.352108471	-0.352108471
152789	4.879008692	6.4335704	5.656289537	-0.352108471	-0.352108471	-0.352108471
152815	4.290620593	8.2903468	6.290483708	1.086055948	0.440372779	1.731739118
152831	8.116665574	7.7702435	7.423821417	6.356491455	6.588093743	6.819696032
152926	3.820316761	1.7836987	-0.252919296	1.78645205	1.169942663	1.478197356
153090	5.550348507	5.5671872	5.55876787	-0.352108471	-0.352108471	-0.352108471
153201	7.681185484	7.1666034	6.652021359	5.180588559	7.816964612	6.498776586
153222	5.939192221	6.0983361	5.780048381	6.49815925	6.204656258	5.911153266
153339	-0.352108471	-0.352108	-0.352108471	7.501937604	4.120009949	5.810973776
153478	5.767649218	7.7448453	6.756247253	5.554237038	7.901415066	6.727826052
153527	4.434314669	4.4826926	4.385936778	4.977710418	5.054280952	4.901139884
153562	5.579960479	7.09719	6.338575234	0.624614178	0.440372779	0.808855576
153642	6.877873345	6.8584729	6.868173108	0.542875446	-0.352108471	1.437859363
153684	6.623540933	6.6538708	6.593211042	-0.352108471	-0.352108471	-0.352108471
153830	-0.352108471	-0.352108	-0.352108471	6.301401509	5.052267685	3.803133862
153918	-0.352108471	-0.352108	-0.352108471	3.818577921	2.451290702	1.084003484
154043	3.127737875	3.136327	3.119148782	6.330004048	6.383151496	6.276856601
154141	7.567747	7.1689345	7.966559536	6.810436227	6.720696571	6.900175883
154150	4.731111372	4.8304259	4.631796822	-0.352108471	-0.352108471	-0.352108471
154215	-0.352108471	-0.352108	-0.352108471	4.6888805	3.919505936	5.458255064
154664	3.682181533	4.4314288	2.932934247	2.943999871	3.228124277	3.086062074
154761	6.735779686	4.5730519	8.898507441	0.044132154	0.440372779	-0.352108471
154791	6.236695219	5.3783539	7.095036499	7.106495446	5.607376822	4.108258198
154796	6.954346295	7.1053136	6.803378955	4.913453562	7.832520937	6.372987249
154807	5.555777437	4.6490074	6.462547521	6.699412027	4.013532912	5.35647247

154881	-0.352108471	-0.352108	-0.352108471	3.988552347	3.725166081	3.461779814
155006	6.201853743	5.0218469	5.611850338	6.257154943	5.142321764	4.027488586
155054	-0.352108471	-0.352108	-0.352108471	3.699359721	4.245214285	3.153505157
155066	-0.352108471	-0.352108	-0.352108471	1.879468461	5.95954341	3.919505936
155185	7.893365184	7.5180418	7.142718369	0.044132154	-0.352108471	0.440372779
155400	-0.352108471	-0.352108	-0.352108471	3.090828498	6.229286881	4.66005769
155435	-0.352108471	-0.352108	-0.352108471	6.192795461	5.169157826	4.14552019
157247	6.325912701	11.362795	8.844353864	7.768793458	7.795411926	7.74217499
157273	7.258571938	8.519519	7.889045476	2.097698605	4.523055044	6.948411483
157285	6.582680225	6.0718659	5.561051563	-0.352108471	-0.352108471	-0.352108471
157381	4.904231184	5.5240469	4.284415506	0.791292145	0.440372779	1.14221151
157506	7.237411851	7.5532206	6.921603065	6.555262436	7.369100357	5.741424516
157657	5.079912626	5.0843562	5.088799708	1.873707872	1.646479888	2.100935855
157693	6.674128879	4.3880633	8.960194414	-0.352108471	-0.352108471	-0.352108471
157769	5.719564994	5.7155573	5.723572646	4.456886199	4.482692559	4.43107984
157777	6.556908606	6.9786128	6.135204448	1.227667732	0.808855576	1.646479888
157869	9.498378266	1.773305	5.635841639	-0.352108471	-0.352108471	-0.352108471
157922	6.288510867	4.0206929	8.556328861	6.581341534	6.637744513	6.609543024
157983	4.819798327	8.184897	6.502347686	4.974100742	7.141919785	6.058010264
158046	3.080305375	8.0676534	5.573979364	-0.449453392	3.546225348	7.541904089
158055	-0.352108471	-0.352108	-0.352108471	5.233567424	4.01862044	6.448514407
158056	-0.352108471	-0.352108	-0.352108471	0.95025487	2.252618212	-0.352108471
158067	5.657577612	3.2679932	8.047161978	2.587713284	4.245214285	0.930212283
158135	-0.352108471	-0.352108	-0.352108471	3.859689372	2.19005738	0.520425389
158158	7.341995514	6.5634192	6.952707338	-0.352108471	-0.352108471	-0.352108471
158234	6.978516816	6.0966267	6.537571752	2.76826138	8.107722042	5.437991711
158314	-0.352108471	-0.352108	-0.352108471	4.907213164	5.080554094	5.253895024
158326	2.162429289	8.9551065	5.55876787	-0.352108471	-0.352108471	-0.352108471
158358	8.57691103	2.5406247	5.55876787	6.318987861	7.274641898	6.796814879
158399	6.692425927	7.6038948	5.780957025	6.769641641	7.721399865	5.817883416
158401	-0.352108471	-0.352108	-0.352108471	3.919505936	3.528926197	4.310085674
158405	6.212536209	8.0296933	4.395379098	1.999631788	1.931214528	2.068049048
158435	6.257523222	5.4610651	7.053981388	2.881537061	4.954218546	0.808855576
158696	6.110613973	6.3737167	5.847511245	9.015971147	4.548116978	6.782044062
158724	-0.352108471	-0.352108	-0.352108471	5.021244133	7.810613816	6.415928975
158866	4.812291254	6.7335249	5.772908066	2.419080028	1.916337486	1.413594945
158931	-0.352108471	-0.352108	-0.352108471	6.039651413	7.837119555	4.242183271
159013	4.730615771	6.5856878	2.87554372	3.585651366	2.191310813	4.979991919
159090	-0.352108471	-0.352108	-0.352108471	8.091794594	10.17697388	6.00661531
159195	6.762783932	7.4239334	6.101634422	5.67944993	5.665786693	5.693113166
160287	7.678111331	5.3546856	3.031259967	-0.352108471	-0.352108471	-0.352108471
160428	6.867473012	6.5884653	7.146480757	1.995427359	2.051108653	1.939746065
160518	6.150905078	6.1079622	6.193847962	5.338134843	5.421579811	5.254689875
160760	-0.352108471	-0.352108	-0.352108471	2.069354904	2.933697157	2.501526031
160857	7.412792659	7.1881098	7.637475505	7.170749369	6.773846551	7.567652187
160897	-0.352108471	-0.352108	-0.352108471	5.412561087	5.421579811	5.403542364
161142	-0.352108471	-0.352108	-0.352108471	4.520091556	6.251034844	7.981978131

161145	5.805829598	7.3184208	4.293238376	-0.352108471	-0.352108471	-0.352108471
161291	-0.352108471	-0.352108	-0.352108471	0.947967741	2.124237496	3.300507251
161357	7.988338592	4.495758	6.242048279	-0.352108471	-0.352108471	-0.352108471
161424	4.523354777	6.3691566	8.214958404	1.707893654	2.630956295	2.169424975
161635	5.734392038	4.0180573	7.450726788	-0.352108471	-0.352108471	-0.352108471
161742	8.906589873	5.7207288	7.31365934	5.598405697	4.41206724	6.784744154
161779	-0.352108471	-0.352108	-0.352108471	3.916845328	6.421470323	5.169157826
161823	-0.352108471	-0.352108	-0.352108471	2.024343053	2.051108653	1.997577452
161829	7.145882028	6.614467	7.677297084	8.199529303	6.602203591	5.004877879
161882	6.038453511	4.3955272	7.681379817	-0.352108471	-0.352108471	-0.352108471
162073	7.051611387	8.0255579	6.077664882	5.877005427	5.00695276	6.747058095
162239	6.690051919	5.6251608	4.560269624	4.626464242	5.958359385	7.290254527
162282	5.108579594	6.6399616	8.171343586	-0.352108471	-0.352108471	-0.352108471
162427	5.287855205	4.9140225	5.661687951	2.281847319	1.876861374	2.686833264
162963	5.727797845	5.9002924	6.072786916	-0.352108471	-0.352108471	-0.352108471
162967	8.534281946	2.8226101	5.678446034	2.118278685	1.057471689	3.179085681
162968	-0.352108471	-0.352108	-0.352108471	4.80208491	6.745348393	2.858821427
162972	6.875766365	6.8194234	6.847594901	5.363386034	8.151029767	2.575742301
162989	6.493400374	5.7752316	5.057062792	-0.352108471	-0.352108471	-0.352108471
162998	5.386853672	7.5709185	9.754983296	6.307069424	4.884089374	7.730049475
163033	6.984591889	7.7034158	7.344003864	-0.352108471	-0.352108471	-0.352108471
163049	4.914022459	4.9086178	4.919427076	8.262569965	4.40533195	6.333950958
163051	6.87819102	3.3402747	5.109232857	2.493184469	5.586906204	8.680627938
163059	-0.352108471	-0.352108	-0.352108471	2.544098373	2.439966475	2.492032424
163071	7.619806991	6.6885154	5.757223771	5.289214314	6.4387435	7.588272686
163087	7.509248289	6.6182187	5.727189027	2.221587275	2.179939357	2.13829144
163126	-0.352108471	-0.352108	-0.352108471	3.008569927	3.482681784	3.245625856
163175	-0.352108471	-0.352108	-0.352108471	2.958488302	0.934617512	1.946552907
163227	4.639213866	5.681728	6.724242091	1.835742592	1.731739118	1.939746065
163351	3.915406297	8.3729661	6.144186222	6.368888508	6.33538923	6.402387785
163479	-0.352108471	-0.352108	-0.352108471	1.968661759	1.997577452	1.939746065
163590	9.667052281	9.5187163	9.815388235	8.610885543	8.817423118	8.404347968
163732	5.413052115	6.4636749	7.514297714	-0.352108471	-0.352108471	-0.352108471
163859	6.353573297	4.3137621	8.393384512	6.548894265	6.979571411	6.764232838
163933	4.316673712	5.8529375	7.389201212	-0.352108471	-0.352108471	-0.352108471
164091	4.438054711	9.3194704	6.878762534	6.589897504	6.038453511	5.487009518
164656	5.575517823	6.4495955	4.701440103	-0.352108471	-0.352108471	-0.352108471
165215	3.140571014	5.8476283	8.554685627	6.931936377	6.188466951	7.675405804
165324	5.856153166	4.5909914	5.223572287	-0.352108471	-0.352108471	-0.352108471
165631	4.792303669	3.6298793	5.954728031	-0.352108471	-0.352108471	-0.352108471
165918	1.866037237	6.167134	4.01658562	-0.352108471	-0.352108471	-0.352108471
166348	7.861969562	7.845843	7.829716523	6.939394323	8.232170041	5.646618606
166614	6.831289207	8.0675907	5.594987685	-0.352108471	-0.352108471	-0.352108471
166785	6.183964016	6.1999612	6.167966808	-0.352108471	-0.352108471	-0.352108471
166863	-0.352108471	-0.352108	-0.352108471	4.783927893	4.80208491	4.820241928
166979	1.816709515	3.663711	2.740210233	-0.352108471	-0.352108471	-0.352108471
167465	3.739479841	6.3655266	5.052503221	8.140519457	6.847035404	5.553551352

167681	6.199595632	4.532658	7.866533263	-0.352108471	-0.352108471	-0.352108471
168374	2.515697587	5.3024132	-0.27101804	-0.352108471	-0.352108471	-0.352108471
168448	9.129930389	4.0517459	6.590838154	5.17630952	4.67635013	4.926329825
168451	8.08779652	3.3668546	5.727325549	3.703134593	1.587843812	5.818425374
168455	-0.352108471	-0.352108	-0.352108471	5.835528372	3.272373272	8.398683471
168507	-0.352108471	-0.352108	-0.352108471	2.076022254	2.100935855	2.051108653
168850	-0.352108471	-0.352108	-0.352108471	6.754140297	5.357967701	6.056053999
169355	6.112592537	4.4573562	5.284974356	-0.352108471	-0.352108471	-0.352108471
169436	6.899497233	5.9326023	7.866392188	-0.352108471	-0.352108471	-0.352108471
169522	-0.352108471	-0.352108	-0.352108471	5.369332089	2.938996888	7.799667289
169611	5.090461483	7.4989785	6.294719976	1.640731613	1.731739118	1.549724107
169714	-0.352108471	-0.352108	-0.352108471	2.19005738	2.232575625	2.147539136
169792	-0.352108471	-0.352108	-0.352108471	5.190180163	6.396868383	7.603556602
170062	-0.352108471	-0.352108	-0.352108471	5.017136436	4.095043541	5.93922933
170384	-0.352108471	-0.352108	-0.352108471	7.689309429	6.104429468	4.519549506
170394	5.304221134	6.5741633	5.939192221	4.508316383	5.095423988	5.682531593
170463	7.360780676	8.7198802	6.001681123	7.364389464	6.25544224	8.473336688
170575	5.140821053	6.4858568	5.813338943	3.104255198	4.692649193	6.281043187
170591	4.401354803	6.9629664	5.682160582	-0.352108471	-0.352108471	-0.352108471
170622	-0.352108471	-0.352108	-0.352108471	7.075027314	6.847035404	6.619043495
170685	-0.352108471	-0.352108	-0.352108471	6.139508822	5.393523004	4.647537187
170689	2.62245663	0.7439189	4.500994336	0.95025487	2.252618212	-0.352108471
170691	7.193586113	7.0919708	7.29520141	1.437859363	1.437859363	1.437859363
170954	6.335199306	5.3546856	4.374171992	5.967566423	5.233567424	4.499568424
170958	1.783698732	3.9195059	-0.352108471	2.553073296	5.458255064	-0.352108471
170959	4.983925974	6.1121944	3.855657502	1.05704313	0.808855576	1.305230684
170960	4.125736893	2.9239264	1.722115859	5.102762726	6.068683866	5.585723296
171017	-0.352108471	-0.352108	-0.352108471	5.412561087	4.377562188	6.447559987
171023	5.90029238	3.290601	8.509983786	7.242526124	5.55876787	3.875009616
171024	-0.352108471	-0.352108	-0.352108471	2.024343053	2.051108653	1.997577452
171220	3.918308276	5.1227518	6.327195423	1.653454619	1.290035436	0.926616254
171484	-0.352108471	-0.352108	-0.352108471	2.481575183	-0.352108471	5.315258837
171546	7.115577709	6.4818442	6.798710977	1.995427359	2.051108653	1.939746065
171586	-0.352108471	-0.352108	-0.352108471	4.225500254	5.017136436	5.808772617
192111	8.192691989	8.3501449	8.035239038	5.594834376	5.731413688	5.458255064
192134	8.09705323	7.4321811	7.764617143	3.012289909	3.337746555	2.686833264
192286	6.463674915	6.479333	6.448016836	6.786143201	6.813225323	6.759061079
192668	5.688622647	5.041865	6.335380327	4.178899946	2.192875082	6.16492481
192669	-0.352108471	-0.352108	-0.352108471	2.252618212	3.96848257	0.536753853
195827	-0.352108471	-0.352108	-0.352108471	3.553766553	4.353101604	3.953434078
196047	10.05330911	7.9252296	8.989269339	7.135075351	8.587597945	5.682552757
196074	-0.352108471	-0.352108	-0.352108471	6.039651413	6.073654487	6.005648339
196264	6.221869239	5.1370351	7.306703386	6.150814957	3.967265593	8.334364321
196294	6.607377459	7.2456794	5.969075484	0.044132154	0.440372779	-0.352108471
196383	6.224862874	6.2436502	6.206075575	-0.352108471	-0.352108471	-0.352108471
196403	5.066569165	4.9042312	4.741893203	-0.352108471	-0.352108471	-0.352108471
196415	-0.352108471	-0.352108	-0.352108471	4.557244691	5.079549824	4.034939558

196441	-0.352108471	-0.352108	-0.352108471	3.964067715	4.66005769	5.356047664
196463	1.832656838	4.7311114	7.629565907	5.373155485	4.231215835	6.515095135
196483	-0.352108471	-0.352108	-0.352108471	1.631676003	3.615460478	-0.352108471
196527	-0.352108471	-0.352108	-0.352108471	1.954444309	1.807952763	2.100935855
196740	7.119744898	7.4217343	7.723723711	-0.167816955	1.689109503	3.546035961
196883	6.068499534	4.976069	5.522284255	-0.352108471	-0.352108471	-0.352108471
196968	6.032792961	5.0894122	4.146031442	1.179289842	0.808855576	1.549724107
197021	2.15990226	4.671913	-0.352108471	4.840642371	5.959771619	7.078900868
197131	5.023982941	6.3532624	5.688622647	-0.352108471	-0.352108471	-0.352108471
197135	-0.352108471	-0.352108	-0.352108471	3.241094247	2.051108653	4.43107984
197196	5.269218467	7.1805073	6.224862874	3.988831276	4.900032819	5.811234362
197358	9.492532054	6.9442237	4.395915248	7.045895639	6.991300859	7.100490419
197370	7.382956475	5.7341724	4.085388274	-0.352108471	-0.352108471	-0.352108471
199223	5.00982419	6.1215288	7.233233348	-0.352108471	-0.352108471	-0.352108471
199699	8.604899524	7.3999659	6.195032352	7.705342059	5.761754088	6.733548073
199704	-0.352108471	-0.352108	-0.352108471	6.019409619	6.073654487	5.965164752
199745	-0.352108471	-0.352108	-0.352108471	3.245625856	3.337746555	3.153505157
199746	4.615387423	5.8824943	7.149601117	3.500810671	5.892148037	8.283485403
199777	5.664088163	5.3387163	5.989460026	1.825329981	2.265129821	1.385530142
199800	4.273206125	6.8443296	5.55876787	-0.352108471	-0.352108471	-0.352108471
199870	3.127737875	0.4549993	5.800476446	6.164724575	6.175335611	6.170030093
199899	3.600481751	0.6653328	2.132907287	3.235175761	2.520455855	2.877815808
199990	-0.352108471	-0.352108	-0.352108471	6.410170138	3.847357133	5.128763636
200010	6.637888669	6.9003701	6.375407279	-0.352108471	-0.352108471	-0.352108471
200030	-0.352108471	-0.352108	-0.352108471	6.234949724	3.585121301	0.935292878
200058	-0.352108471	-0.352108	-0.352108471	6.415928975	7.336154076	5.495703874
200081	-0.352108471	-0.352108	-0.352108471	7.030577409	7.188109812	6.873045007
200150	-0.352108471	-0.352108	-0.352108471	0.998561388	2.168105043	3.337648697
200162	5.876952844	6.8183689	4.93553678	5.983736712	5.52941672	6.438056703
200185	5.673266141	7.5414888	6.607377459	4.145042328	5.789762726	7.434483124
200197	-0.352108471	-0.352108	-0.352108471	5.409444926	7.26094789	6.335196408
200315	6.794186972	8.7005759	7.747381455	8.368204667	7.228052884	6.0879011
200316	3.41407487	4.8822675	1.94588227	5.395886994	3.615867194	1.835847393
200539	6.640019393	6.6538708	6.626167961	-0.352108471	-0.352108471	-0.352108471
200558	6.384848847	6.2800845	6.332466669	-0.352108471	-0.352108471	-0.352108471
200576	5.639975304	6.9695558	4.310394757	6.166112602	4.237555116	5.201833859
200734	-0.352108471	-0.352108	-0.352108471	7.551136183	7.801394858	7.300877508
200765	6.524020149	7.1912022	6.857611197	7.394837442	6.806019973	7.100428707
200879	6.969681275	7.9927758	5.946586714	4.275651379	7.506073575	1.045229183
200916	7.504511108	7.5506489	7.458373336	7.344860083	7.361907759	7.327812407
200933	9.509257301	7.0733449	8.291301101	7.855359013	7.128044171	6.400729329
201161	4.368112127	5.7494021	7.130692101	-0.352108471	-0.352108471	-0.352108471
201163	7.8724194	6.7895548	5.706690245	3.883156074	5.971467073	1.794845074
201164	8.1436829	5.7545997	6.949141282	1.345967809	1.14221151	1.549724107
201181	8.246814975	7.136534	9.357095981	8.254856327	6.62360496	7.439230644
201191	7.299569177	5.0622456	6.180907385	-0.352108471	-0.352108471	-0.352108471
201254	3.085573541	5.6251608	8.164748002	0.044132154	0.440372779	-0.352108471

201255	-0.352108471	-0.352108	-0.352108471	5.458255064	4.206331409	6.710178719
201292	7.428247301	7.6152586	7.241236027	2.903700168	2.252618212	3.554782125
201475	6.90161932	8.3052319	5.498006746	3.05504663	0.8680033	1.961524965
201477	-0.352108471	-0.352108	-0.352108471	5.131838854	7.166836847	6.149337851
201595	6.47470686	6.8227154	7.170723976	6.267548248	6.719517257	6.493532752
201627	-0.352108471	-0.352108	-0.352108471	2.937210282	6.382905097	4.66005769
201633	-0.352108471	-0.352108	-0.352108471	5.417224669	5.281537812	5.349381241
201853	13.43556854	13.778924	14.12228029	12.56854535	13.23753795	13.90653055
201895	7.715071783	7.474041	7.95610257	7.692157917	7.528756621	7.855559213
201931	-0.352108471	-0.352108	-0.352108471	4.703081131	7.505777804	6.104429468
201965	-0.352108471	-0.352108	-0.352108471	5.095423988	5.124543452	5.066304524
202052	6.132119415	6.9789464	7.825773465	8.345199752	1.674104462	5.009652107
202299	-0.352108471	-0.352108	-0.352108471	6.679845279	1.929217798	4.304531538
202374	6.672937028	5.658707	4.64447699	-0.352108471	-0.352108471	-0.352108471
202781	4.515752443	7.0529906	9.590228763	0.624614178	0.440372779	0.808855576
203068	10.0301857	10.262929	9.797442603	10.38372362	11.69779243	9.069654805
203100	5.28323533	6.6924259	8.101616525	1.585227283	2.529447424	3.473667564
203102	-0.352108471	-0.352108	-0.352108471	5.582192779	4.813830548	6.35055501
203197	7.903179714	5.3776118	2.852043788	3.584762911	2.986345121	4.183180702
203245	6.477919921	8.0975434	9.717166944	5.941194113	8.006828735	6.974011424
203260	-0.352108471	-0.352108	-0.352108471	5.311586026	6.104159744	6.896733462
203286	5.729410118	5.4283939	5.127377631	-0.352108471	-0.352108471	-0.352108471
203522	5.017136436	5.054281	4.979991919	-0.352108471	-0.352108471	-0.352108471
203547	6.67090677	8.3535832	7.512244991	6.801905484	5.924443035	5.046980586
204010	7.591293105	6.1515856	4.711878152	5.192287165	6.022050925	6.851814685
204851	-0.352108471	-0.352108	-0.352108471	2.99677462	4.688318557	1.305230684
205327	-0.352108471	-0.352108	-0.352108471	6.862051769	5.785505277	4.708958786
205428	-0.352108471	-0.352108	-0.352108471	6.227060744	5.415760932	5.821410838
205564	3.416753068	7.7075662	5.562159617	8.280354971	7.240174153	6.199993335
205717	6.272317051	7.4769937	5.067640382	8.990037256	6.964395188	4.938753119
207063	-0.352108471	-0.352108	-0.352108471	4.424689719	6.087644454	7.75059919
219287	5.38675695	5.3152588	5.458255064	1.475082136	1.14221151	1.807952763
219333	5.428393874	5.4582551	5.398532684	-0.352108471	-0.352108471	-0.352108471
219347	4.963301433	4.671913	5.254689875	5.962540258	5.315258837	6.609821679
219402	6.75496194	5.9467163	7.563207537	-0.352108471	-0.352108471	-0.352108471
219447	5.999674881	6.2154027	6.107538801	-0.352108471	-0.352108471	-0.352108471
219482	6.816905042	6.3469852	7.286824927	-0.352108471	-0.352108471	-0.352108471
219527	-0.352108471	-0.352108	-0.352108471	4.716626033	6.266367194	7.816108355
219541	-0.352108471	-0.352108	-0.352108471	2.313941724	-0.352108471	4.979991919
219654	-0.352108471	-0.352108	-0.352108471	5.401218419	9.236610442	7.318914431
219699	5.288995261	5.1522471	5.015498966	4.701546485	6.492287497	5.596916991
219790	-0.063977979	2.3139417	4.691861427	2.242596918	2.232575625	2.252618212
219844	6.490914351	5.035798	7.94603069	2.808873637	2.334436445	1.859999252
219855	9.567757604	10.057749	9.077766391	5.943092199	8.979859061	7.46147563
219874	6.573968999	7.4045974	5.743340581	-0.352108471	-0.352108471	-0.352108471
219875	-0.352108471	-0.352108	-0.352108471	5.903760504	6.088693899	5.718827109
219902	7.052488825	6.206447	5.360405116	7.264035061	6.068415012	4.872794963

219931	-0.352108471	-0.352108	-0.352108471	4.400452081	4.688318557	4.112585606
219938	5.493783215	5.4205665	5.566999896	-0.352108471	-0.352108471	-0.352108471
219972	-0.352108471	-0.352108	-0.352108471	5.078578358	3.36730463	1.656030901
219988	6.463674915	5.7974013	7.129948491	7.468165213	6.251034844	5.033904474
220002	-0.352108471	-0.352108	-0.352108471	5.713395024	5.733576008	5.723485516
220070	4.129193875	4.9841517	5.839109536	2.073301066	1.89926805	2.247334082
220074	8.002836109	9.0525622	6.95311002	6.407045466	6.66228969	6.917533914
220082	7.233420289	6.9150602	7.551780416	-0.352108471	-0.352108471	-0.352108471
220108	7.575267767	8.5943083	6.556227274	0.624614178	0.808855576	0.440372779
220134	8.796764647	8.8524604	8.741068878	7.149231072	7.245996144	7.052466
220323	6.332466669	4.5790376	8.085895716	8.757508619	7.067751423	7.912630021
220359	8.240890183	4.6597155	6.450302837	1.158344319	3.346892105	2.252618212
220429	2.711561556	2.4779183	2.94520481	-0.352108471	-0.352108471	-0.352108471
220441	5.711925407	4.8708731	5.291399258	1.591415247	2.252618212	0.930212283
220594	-0.352108471	-0.352108	-0.352108471	2.461532669	4.482692559	0.440372779
220729	7.795945694	4.718147	6.257046343	4.714381525	3.156541914	3.935461719
220906	6.886923832	5.9628388	6.424881323	6.594186439	5.736549925	7.451822953
220930	2.280956994	4.9880158	-0.426101797	-0.352108471	-0.352108471	-0.352108471
220988	-0.352108471	-0.352108	-0.352108471	6.906890338	6.420822611	6.663856474
221002	6.502263028	6.65465	6.349876069	-0.352108471	-0.352108471	-0.352108471
221035	5.999482013	5.6142717	5.806876842	2.025457316	6.249600322	4.137528819
221060	6.87759802	4.5443591	5.710978578	0.975533543	1.14221151	0.808855576
221074	6.152439471	6.2697825	6.035096416	-0.352108471	-0.352108471	-0.352108471
221091	5.990519134	6.2873746	6.138946856	6.041417066	5.47633612	4.911255175
221092	5.380817234	5.8001998	6.219582341	6.288041837	5.920817098	6.104429468
221143	-0.352108471	-0.352108	-0.352108471	3.701877892	2.191310813	5.212444972
221150	-0.352108471	-0.352108	-0.352108471	2.895806021	5.052267685	7.20872935
221154	6.002308672	6.0023087	6.002308672	5.958359385	5.958359385	5.958359385
221178	3.127737875	3.7195458	2.53592998	4.178899946	3.19879068	5.159009211
221188	-0.352108471	-0.352108	-0.352108471	3.767483207	3.615460478	3.919505936
221223	3.758937782	2.7606188	1.762299734	0.476561106	1.305230684	-0.352108471
221301	-0.352108471	-0.352108	-0.352108471	4.456886199	7.702447163	1.211325236
221322	5.492945331	8.0641207	6.778533013	3.31024476	2.753318655	3.867170866
221336	-0.352108471	-0.352108	-0.352108471	4.927030449	4.438505554	3.949980658
221393	-0.352108471	-0.352108	-0.352108471	4.594993411	3.585121301	2.575249191
221395	7.570869798	5.8648729	6.717871361	-0.352108471	-0.352108471	-0.352108471
221400	5.55876787	3.0290065	8.088529221	-0.352108471	-0.352108471	-0.352108471
221442	4.64626819	4.8884122	4.404124174	2.247393013	1.807952763	2.686833264
221477	6.624424137	3.8105581	5.217491139	-0.352108471	-0.352108471	-0.352108471
221491	-0.352108471	-0.352108	-0.352108471	5.801098295	6.038453511	5.563743079
221656	-0.352108471	-0.352108	-0.352108471	5.095423988	5.124543452	5.066304524
221692	3.682181533	3.9834604	3.380902678	5.09343014	6.59756953	5.845499835
221710	6.748384093	6.7990975	6.77374078	-0.352108471	-0.352108471	-0.352108471
221718	-0.352108471	-0.352108	-0.352108471	6.084074277	6.32531018	6.566546084
221749	7.39370248	6.1215288	4.849355057	0.446802307	6.723440295	3.585121301
221785	-0.352108471	-0.352108	-0.352108471	2.187383195	2.317853228	2.252618212
221830	3.002603941	7.0178419	5.010222934	-0.352108471	-0.352108471	-0.352108471

221833	-0.352108471	-0.352108	-0.352108471	6.826080339	4.893154399	5.859617369
221883	6.151585628	5.1530189	7.150152377	-0.352108471	-0.352108471	-0.352108471
221895	8.397996571	7.7150774	8.056536983	-0.352108471	-0.352108471	-0.352108471
221908	-0.352108471	-0.352108	-0.352108471	5.669144456	4.80208491	3.935025364
221914	-0.352108471	-0.352108	-0.352108471	4.591994762	5.637296679	6.682598595
221927	-0.352108471	-0.352108	-0.352108471	2.902991023	4.409326454	1.396655591
221935	4.947007189	2.7692418	7.124772545	-0.352108471	-0.352108471	-0.352108471
221937	10.22061804	10.803214	9.638022037	8.501347401	8.518425304	8.484269499
221938	2.351086241	1.2012726	3.500899872	1.270297347	0.808855576	1.731739118
221960	-0.352108471	-0.352108	-0.352108471	3.52433951	2.147539136	4.901139884
221981	-0.352108471	-0.352108	-0.352108471	5.704335255	9.272440781	2.136229728
222068	9.94127085	4.3903389	7.165804888	0.476561106	-0.352108471	1.305230684
222235	6.429170264	6.1999612	6.658379305	5.080554094	5.529311366	4.631796822
222236	6.366945375	7.0274221	5.70646867	0.289051906	-0.352108471	0.930212283
222256	6.451724919	6.797363	6.106086851	6.96170561	5.761472688	6.361589149
222484	5.594197874	6.9867748	8.379351626	6.457429872	6.796458395	6.11840135
222553	8.18781607	7.298998	6.410179957	6.135740193	6.652432567	5.61904782
222584	5.828848464	3.8509041	7.806792844	-0.352108471	-0.352108471	-0.352108471
222611	4.372191214	5.6090592	3.13532318	6.9664421	3.067830771	5.017136436
222658	5.38379986	6.7956077	8.207415587	5.989949753	7.495134335	9.000318916
222663	6.767552344	6.8767254	6.658379305	5.546527222	5.529311366	5.563743079
222865	4.413452322	5.9011161	7.388779848	7.136107988	3.876532349	5.506320168
222962	-0.352108471	-0.352108	-0.352108471	6.346162167	3.412762187	9.279562148
223082	-0.352108471	-0.352108	-0.352108471	0.95025487	-0.352108471	2.252618212
246184	-0.352108471	-0.352108	-0.352108471	3.787842279	5.52367653	4.655759405
252839	7.364365087	6.0763762	6.720370627	7.549264307	6.558419977	5.567575647
252969	4.596539004	4.5610633	4.578801146	5.623457858	4.410815013	5.017136436
252983	6.542911663	5.4177564	5.980334028	-0.352108471	-0.352108471	-0.352108471
252995	-0.352108471	-0.352108	-0.352108471	3.555305497	6.267994919	4.911650208
253039	4.045982599	3.6244563	3.202929976	1.253937875	4.798393796	3.026165836
253044	5.375376867	5.2628167	5.31909676	-0.352108471	-0.352108471	-0.352108471
253143	-0.352108471	-0.352108	-0.352108471	6.071430822	4.178899946	2.286369069
253260	6.520082846	8.3964829	7.458282885	3.95204103	5.233567424	6.515093817
253461	8.725426084	6.8018138	4.878201463	-0.352108471	-0.352108471	-0.352108471
253558	-0.352108471	-0.352108	-0.352108471	6.296043865	6.31523113	6.276856601
253635	6.450002038	6.3236499	6.197297765	5.677785997	4.177572433	4.927679215
253639	5.924940787	6.8945567	6.409748741	5.537525048	5.36239135	5.449958199
253714	10.4671732	8.4279133	6.38865339	8.658718272	5.194726176	6.926722224
253827	7.569449151	8.843507	8.206478063	5.388820598	7.876691905	10.36456321
253832	7.451565443	8.6594299	6.243700959	3.061101714	1.14221151	4.979991919
253868	6.607016258	5.8319983	5.056980365	0.762376451	-0.352108471	1.876861374
253982	5.262620663	5.307328	5.284974356	6.008119643	5.562890912	5.785505277
254013	4.187593997	6.4866125	5.337103237	6.549443322	4.747541319	5.648492321
254048	7.537200822	7.9437232	8.350245518	5.586906204	6.02075664	5.153055767
254065	3.683980801	3.4306791	3.937282489	8.30986052	6.463674915	4.61748931
254099	-0.352108471	-0.352108	-0.352108471	5.886425029	5.926961306	5.845888751
254102	8.221827593	5.1264302	6.674128879	2.211943219	2.232575625	2.191310813

254225	6.735779686	6.7508628	6.720696571	-0.352108471	-0.352108471	-0.352108471
254240	-0.352108471	-0.352108	-0.352108471	3.244499551	5.272100811	4.258300181
254295	7.684693308	4.62796	6.156326632	5.217268495	9.537239158	0.897297832
254359	-0.352108471	-0.352108	-0.352108471	6.496886722	6.532588736	6.461184708
254863	5.788905932	5.8810131	5.973120225	4.776442757	6.022050925	7.267659093
254958	-0.352108471	-0.352108	-0.352108471	5.539848076	5.95094085	5.745394463
255022	1.383520155	1.3559597	1.411080616	3.962303322	2.342205358	0.722107394
255082	5.21905516	5.4937832	5.768511269	-0.352108471	-0.352108471	-0.352108471
255189	7.067158728	6.3968684	5.726578037	-0.352108471	-0.352108471	-0.352108471
255252	7.914878548	7.2289277	6.542976772	7.156753887	6.920101788	6.683449689
255324	5.052622603	6.2407803	3.86446487	6.316009143	6.046029896	6.58598839
255403	2.920655209	5.3190968	7.717538311	1.371545024	1.305230684	1.437859363
255654	-0.352108471	-0.352108	-0.352108471	2.865393679	2.508272848	2.686833264
255783	4.720108865	4.3859368	5.054280952	-0.352108471	-0.352108471	-0.352108471
255919	6.783043088	7.5296218	6.036464421	-0.120586129	2.121209733	4.363005595
255928	4.122631124	5.4619762	4.792303669	-0.352108471	-0.352108471	-0.352108471
256006	-0.352108471	-0.352108	-0.352108471	6.633983813	6.197874136	6.415928975
256051	6.905888978	3.6769095	5.291399258	5.298609063	6.329998693	7.361388324
256076	-0.352108471	-0.352108	-0.352108471	7.09771715	7.101695289	7.099706219
256130	-0.352108471	-0.352108	-0.352108471	4.52611146	4.80208491	5.078058361
256281	2.402866965	7.0598175	4.731342239	-0.352108471	-0.352108471	-0.352108471
256302	4.323161992	6.5685405	8.813918972	4.193585611	3.605011212	3.016436813
256355	4.397178123	1.1240594	2.760618758	0.044132154	0.440372779	-0.352108471
256356	5.412785261	2.7365298	0.06027426	3.86802867	3.203670606	2.539312541
256380	8.002453643	5.7645796	6.883516622	5.696774722	8.719275357	7.208025039
256472	8.482280937	6.9398718	5.39746266	-0.352108471	-0.352108471	-0.352108471
256933	-0.352108471	-0.352108	-0.352108471	5.23117587	4.163384448	6.298967293
256987	1.392109249	0.6613649	2.122853612	2.387357251	5.095423988	7.803490725
257000	6.624675593	10.185091	3.064260581	5.277219847	5.156478558	5.397961136
257062	2.618057952	5.5882244	-0.352108471	-0.352108471	-0.352108471	-0.352108471
257106	2.351086241	-0.352108	5.054280952	-0.352108471	-0.352108471	-0.352108471
257144	2.920614754	3.8477905	4.774966251	7.36312201	6.630621079	5.898120148
257364	8.922247489	6.9675112	5.012775001	6.769421128	5.095423988	3.421426848
257396	-0.352108471	-0.352108	-0.352108471	2.888404339	7.51337844	5.20089139
257415	-0.352108471	-0.352108	-0.352108471	5.647436589	5.100686928	4.553937267
257629	5.895373921	5.8284011	5.962346694	6.615527989	6.738393601	6.492662376
259296	4.923749454	6.2738323	7.623915064	-0.352108471	-0.352108471	-0.352108471
260341	3.797822384	3.6761388	3.919505936	3.646079301	5.191222747	2.100935855
260425	2.588601447	-0.352108	5.529311366	-0.352108471	-0.352108471	-0.352108471
261726	4.640191479	8.1642327	6.402212088	0.289051906	-0.352108471	0.930212283
261734	1.383520155	-0.352108	3.119148782	7.036396027	3.457977877	10.61481418
266655	8.071720344	8.7560584	7.387382251	6.831268578	6.733784849	6.928752307
266971	5.741790366	8.4824511	7.112120745	0.440372779	0.440372779	0.440372779
267020	5.334238245	5.3828326	5.285643864	4.212536839	0.674933824	2.443735331
280636	-0.352108471	-0.352108	-0.352108471	2.61692804	6.86293602	11.108944
280659	-0.352108471	-0.352108	-0.352108471	6.395756437	5.45070971	7.340803165
282618	-0.352108471	-0.352108	-0.352108471	1.99196379	3.660200284	2.826082037

282809	-0.352108471	-0.352108	-0.352108471	2.519016059	2.139844176	1.760672292
282890	6.485362982	5.9417621	5.39816117	-0.352108471	-0.352108471	-0.352108471
282969	5.34274782	6.1655016	5.754124716	4.628113008	7.781199508	6.204656258
282980	4.918802255	7.7931481	6.355975153	3.3031696	5.836316644	0.770022555
282991	0.679446692	4.0130283	7.346609939	5.477176123	5.493783215	5.510390307
283011	-0.352108471	-0.352108	-0.352108471	6.671874638	6.689878453	6.653870824
283028	-0.352108471	-0.352108	-0.352108471	2.687313607	5.596916991	8.506520375
283045	5.021296917	6.2016069	7.381916802	1.14221151	1.14221151	1.14221151
283078	5.110258875	7.4966636	6.303461245	-0.352108471	-0.352108471	-0.352108471
283080	4.095303961	3.136327	5.054280952	0.044132154	0.440372779	-0.352108471
283089	7.508871913	7.6881405	7.598506213	6.362981115	6.88868102	5.837281209
283130	4.758034591	7.5438387	6.150936629	6.351793093	6.520834686	6.43631389
283131	11.03821027	8.6471095	9.842659868	6.68348585	8.977568587	7.830527219
283143	-0.352108471	-0.352108	-0.352108471	5.664076022	2.693723869	4.178899946
283149	6.573968999	6.5919201	6.556017863	8.425153342	8.646173588	8.535663465
283174	6.396868383	6.4105853	6.383151496	-0.352108471	-0.352108471	-0.352108471
283194	-0.352108471	-0.352108	-0.352108471	3.1200183	7.938604431	5.529311366
283209	9.484803367	6.932457	8.208630159	6.887728041	7.041867106	6.733588975
283237	-0.352108471	-0.352108	-0.352108471	4.572821938	6.497182024	8.42154211
283267	8.000791442	8.655064	9.309336471	6.151697586	7.108984518	8.066271449
283299	5.181626131	4.4826926	5.880559702	0.476561106	-0.352108471	1.305230684
283335	3.402171849	5.7610936	4.581632733	0.289051906	-0.352108471	0.930212283
283337	-0.352108471	-0.352108	-0.352108471	2.686833264	2.686833264	2.686833264
283349	6.352053864	4.6858026	8.018305156	7.052089784	6.003978506	4.955867227
283373	6.242048279	2.6996043	9.784492276	7.784700846	7.18130912	7.483004983
283375	1.392109249	1.6687394	1.115479055	6.085586463	4.018737342	1.951888221
283377	4.713157169	6.1276241	5.420390631	0.440372779	0.440372779	0.440372779
283404	1.820317844	2.7115616	3.602805268	-0.352108471	-0.352108471	-0.352108471
283422	5.416755409	5.5587679	5.700780331	-0.352108471	-0.352108471	-0.352108471
283431	5.691655254	6.8385531	7.985451044	7.104346779	6.946414218	7.26227934
283432	6.286940651	8.8064836	7.54671213	8.323144418	4.274781825	6.298963122
283450	-0.352108471	-0.352108	-0.352108471	6.370545086	6.96585859	5.775231583
283459	4.528924884	4.671913	4.385936778	6.32531018	6.31523113	6.33538923
283460	7.173278044	8.590362	10.007446	6.411432414	6.960337814	5.862527014
283464	7.017668263	6.9529417	7.082394844	6.278615783	6.174080069	6.383151496
283480	-0.352108471	-0.352108	-0.352108471	8.441625886	4.030216436	6.235921161
283487	7.436272311	7.6193767	7.802481028	8.72888555	7.777997401	6.827109253
283489	-0.352108471	-0.352108	-0.352108471	6.829307464	3.82544391	9.833171017
283491	6.398375221	6.8767254	5.920025058	4.927679215	4.954218546	4.901139884
283507	-0.352108471	-0.352108	-0.352108471	5.054752278	6.121696471	5.588224375
283514	7.398193617	7.4922194	7.304167827	-0.352108471	-0.352108471	-0.352108471
283521	3.820053939	1.2263703	2.523212107	-0.352108471	-0.352108471	-0.352108471
283551	5.018251426	4.2245128	5.811990065	0.440372779	0.440372779	0.440372779
283575	3.986033342	4.617923	3.354143641	1.873849414	1.807952763	1.939746065
283585	6.098078317	5.3223397	6.873816911	3.415895326	2.588220532	3.002057929
283592	5.986976026	4.6417519	7.332200159	1.843175982	2.206185083	2.024680532
283596	-0.352108471	-0.352108	-0.352108471	4.655317146	9.230758645	6.943037895

283624	-0.352108471	-0.352108	-0.352108471	4.832259402	6.830732744	8.829206086
283635	4.380659212	6.2730965	8.165533808	3.514991766	1.565410274	5.464573259
283663	7.360749928	3.7870378	5.573893887	2.380725313	5.344143265	8.307561217
283683	5.546367231	6.0384535	5.054280952	2.529447424	1.939746065	3.119148782
283693	5.625373322	6.2395908	6.853808233	7.569043296	5.925900831	4.282758366
283731	4.817047211	6.365564	5.591305588	3.041395306	4.251326213	5.46125712
283767	5.93871762	5.0488488	5.493783215	3.856218142	3.182601909	2.508985676
283788	-0.352108471	-0.352108	-0.352108471	7.675724814	5.497706334	6.586715574
283796	5.591305588	5.6501613	5.532449852	-0.352108471	-0.352108471	-0.352108471
283856	6.199679026	6.479333	5.920025058	4.499717253	3.337746555	5.661687951
283871	6.468151867	5.8840588	7.052244952	7.387749391	9.674767244	5.100731538
283875	3.630792518	5.4989447	7.36709692	6.721923052	7.014978257	6.428867846
283876	6.038383809	6.2474636	6.456543359	1.223721097	1.305230684	1.14221151
283888	4.151165895	5.0846754	6.018184812	2.722314828	5.475425549	4.098870188
283899	6.521613016	5.6934646	6.107538801	2.686833264	1.608488685	3.765177842
283902	3.884443122	6.6855056	5.284974356	0.044132154	0.440372779	-0.352108471
283922	-0.352108471	-0.352108	-0.352108471	3.038680847	4.948453082	1.128908612
283932	6.177986381	7.0653443	7.952702234	3.293591639	5.941173134	4.617382386
283953	7.368063388	5.9068425	4.445621706	-0.352108471	-0.352108471	-0.352108471
283970	7.261991367	6.9978561	7.526126679	7.095970797	8.170701273	6.02124032
283981	-0.352108471	-0.352108	-0.352108471	3.119148782	3.119148782	3.119148782
283985	4.31885757	4.4315687	4.544279759	2.862380891	2.024680532	1.186980174
283989	5.758523395	3.5522076	7.964839195	-0.352108471	-0.352108471	-0.352108471
283991	7.036907464	5.2061501	6.121528769	2.211943219	0.704149026	3.719737412
284021	6.840836283	5.6462551	4.451673977	4.46509823	4.555364341	4.645630453
284023	9.312623719	8.0402708	6.767917969	10.18278973	7.443142368	4.703495002
284034	5.604405525	9.0949925	7.349699016	7.322437573	7.492610575	7.152264571
284047	7.890470135	9.4261678	10.96186547	5.383562122	10.98670747	8.185134795
284058	-0.352108471	-0.352108	-0.352108471	5.255332057	5.369332089	5.48333212
284069	2.016914154	-0.352108	4.385936778	4.438505554	4.245214285	4.631796822
284080	6.414258136	8.1938494	4.63466686	-0.352108471	-0.352108471	-0.352108471
284086	5.455819204	6.5988068	7.741794438	3.076801296	4.199045311	5.321289325
284098	5.052267685	8.8208009	1.283734475	2.049256654	1.526552342	2.571960965
284106	-0.352108471	-0.352108	-0.352108471	9.133614457	6.280301159	3.42698786
284110	6.013626829	6.0526947	6.033160789	3.182601909	2.252618212	4.112585606
284116	4.273770249	6.3545955	5.314182891	1.14221151	-0.19493469	2.479357709
284119	6.688191699	13.191764	9.939978022	10.38657738	7.710084556	13.0630702
284161	8.104344652	7.8991344	8.309554875	7.310072079	7.342696275	7.277447884
284184	7.385292886	4.9178784	6.151585628	1.689109503	1.731739118	1.646479888
284207	-0.352108471	-0.352108	-0.352108471	1.760746145	3.345400448	2.553073296
284217	5.652271474	5.5293114	5.775231583	4.108072315	5.529311366	2.686833264
284232	14.16164786	12.442451	10.72325433	13.03550605	10.4030105	11.71925827
284257	-0.352108471	-0.352108	-0.352108471	2.451290702	5.254689875	-0.352108471
284276	3.624456288	2.7887771	4.460135447	2.18181885	0.808855576	3.554782125
284297	5.854618147	6.6294784	6.242048279	7.063286202	7.999012266	6.127560139
284323	7.20576003	9.1976926	5.213827432	8.710752167	4.792351609	6.751551888
284344	5.661488853	6.7295934	6.195541118	8.686523674	7.171235926	5.655948177

284352	-0.352108471	-0.352108	-0.352108471	2.252618212	4.117436641	0.387799782
284353	0.022453161	8.1621164	4.092284781	-0.352108471	-0.352108471	-0.352108471
284361	3.846832868	5.7377745	4.792303669	-0.352108471	-0.352108471	-0.352108471
284379	5.386773706	4.9154635	5.858083893	3.39824945	3.33706132	3.367655385
284385	4.529128697	7.7139288	6.121528769	-0.352108471	-0.352108471	-0.352108471
284390	-0.352108471	-0.352108	-0.352108471	2.38621749	4.459315285	0.313119695
284395	4.840598726	6.0908083	7.341017955	-0.352108471	-0.352108471	-0.352108471
284403	5.726143017	6.3146192	6.020381091	-0.352108471	-0.352108471	-0.352108471
284406	8.394732309	8.4891742	8.583616018	6.016006936	6.989965632	5.04204824
284412	6.18966628	6.3956138	5.983718797	7.295277878	7.026651754	7.563904002
284418	-0.352108471	-0.352108	-0.352108471	5.095423988	5.124543452	5.066304524
284428	-0.352108471	-0.352108	-0.352108471	2.451290702	5.254689875	-0.352108471
284434	7.441875269	8.5263285	6.357422	2.73734835	3.012289909	3.287231469
284440	8.246225822	6.6719406	9.820511058	6.363762655	5.957816387	6.769708924
284454	6.721780511	5.3950788	6.058429676	5.890439961	5.047778452	6.733101471
284551	7.165804888	7.4934092	6.838200581	7.501358274	7.548624299	7.595890325
284576	5.998406448	11.212097	0.784715434	-0.771952874	3.96815687	1.598101998
284577	3.54853814	7.1980133	5.373275698	6.728388561	10.83142141	2.625355717
284581	7.077671653	6.7014235	6.889547564	5.984984884	7.455763618	4.514206149
284600	5.575517823	6.2865194	4.864516278	2.376386664	2.393884559	2.411382455
284612	7.240552188	2.145426	4.692989119	2.075707141	2.473324272	2.274515706
284613	3.376572818	6.7744663	5.075519558	3.771392125	4.742680396	5.713968666
284615	6.640019393	6.9041683	6.375870496	-0.352108471	-0.352108471	-0.352108471
284618	3.064219239	2.6573268	2.860773008	-0.352108471	-0.352108471	-0.352108471
284649	4.579618372	4.9140225	4.245214285	-0.352108471	-0.352108471	-0.352108471
284677	-0.352108471	-0.352108	-0.352108471	5.23117587	6.241350462	4.221001278
284695	-0.352108471	-0.352108	-0.352108471	4.472882012	3.564592672	4.018737342
284739	5.284974356	5.2627535	5.307195168	-0.352108471	-0.352108471	-0.352108471
284805	5.339482499	3.0183174	4.178899946	2.463550772	3.119148782	1.807952763
284865	7.886153128	5.4756111	6.68088213	1.023607914	2.334436445	3.645264975
284889	-0.352108471	-0.352108	-0.352108471	6.80054651	5.730016661	4.659486811
284898	3.515141236	3.6761388	3.354143641	5.369332089	5.383503645	5.355160532
284904	7.623360775	8.3343564	6.912365178	6.102629885	5.823341069	6.3819187
284912	4.821539291	5.2020263	4.441052278	2.342793532	4.245214285	0.440372779
284942	3.181437345	4.5195869	5.857736495	5.938576252	3.664596399	8.212556106
284948	1.383520155	-0.352108	3.119148782	3.132770386	5.825167992	0.440372779
284950	5.741379086	5.1417464	4.542113696	6.681290203	7.395842732	7.038566468
284992	6.06875656	3.539709	8.597804163	-0.352108471	-0.352108471	-0.352108471
285025	-0.352108471	-0.352108	-0.352108471	3.7090491	3.631776812	3.554504525
285033	8.088789171	3.1615324	5.625160771	-0.352108471	-0.352108471	-0.352108471
285051	-0.352108471	-0.352108	-0.352108471	6.473375392	3.864940259	5.169157826
285103	5.284974356	4.3603524	6.209596345	3.266312788	2.655229797	2.044146806
285148	7.582855329	7.5378446	7.49283394	7.154670274	5.987934388	8.321406159
285175	5.248302639	6.9382755	6.093289078	-0.352108471	-0.352108471	-0.352108471
285195	6.151585628	8.4046078	3.898563503	-0.352108471	-0.352108471	-0.352108471
285203	5.061063419	6.3488076	5.704935534	0.82273449	-0.352108471	1.997577452
285267	-0.352108471	-0.352108	-0.352108471	5.412561087	5.421579811	5.403542364

285282	9.259499519	5.0946149	7.177057188	3.067083405	5.695288594	8.323493783
285313	6.212536209	8.0140475	4.411024871	-0.352108471	-0.352108471	-0.352108471
285315	6.822470677	5.8484172	6.33544393	5.544294567	5.964812231	5.754553399
285335	-0.352108471	-0.352108	-0.352108471	2.211943219	2.232575625	2.191310813
285349	6.072888354	6.1688661	6.12087721	4.777745925	5.663737322	6.549728718
285352	2.760618758	3.0225605	2.498676991	0.044132154	0.440372779	-0.352108471
285368	5.006130849	4.9774847	4.948838525	3.469178822	3.226161794	2.983144766
285375	4.052071482	4.9315679	5.811064255	1.688802714	1.437859363	1.939746065
285382	6.053201629	5.0936262	5.573413938	5.100841931	6.724087732	8.347333533
285386	-0.352108471	-0.352108	-0.352108471	4.927679215	4.917815466	4.937542964
285440	8.938668253	9.5625529	8.314783573	10.04994894	5.810128151	7.930038544
285456	4.297291017	2.7502277	5.844354326	6.001809131	4.294711678	7.708906585
285463	7.105747243	7.0241903	6.942633299	3.53749254	7.931291536	5.734392038
285484	6.323345273	5.5535381	4.783731017	-0.352108471	-0.352108471	-0.352108471
285513	7.232638921	6.3324667	5.432294416	-0.352108471	-0.352108471	-0.352108471
285521	5.810645685	4.8572962	3.903946657	4.264737406	2.954087547	5.575387265
285527	5.614711362	8.3688787	6.991795048	-0.352108471	-0.352108471	-0.352108471
285550	-0.352108471	-0.352108	-0.352108471	6.588254064	6.458297792	6.718210335
285605	7.426373543	7.1946087	6.962843781	6.295750117	6.959074387	5.632425847
285613	-0.352108471	-0.352108	-0.352108471	5.281319318	3.919505936	2.557692553
285636	6.640019393	6.6538708	6.626167961	-0.352108471	-0.352108471	-0.352108471
285638	6.504078908	7.2416967	7.979314469	-0.151652638	3.522070642	7.195793922
285672	6.843992567	6.9981067	6.689878453	1.05704313	0.808855576	1.305230684
285679	4.522024694	5.3159043	3.728145125	-0.352108471	-0.352108471	-0.352108471
285696	6.768942691	6.8028795	6.73500589	4.124812677	4.825632758	4.475222718
285704	5.048581062	8.7783967	6.913488898	6.658269112	7.450932417	7.054600764
285708	4.297291017	4.0509069	4.543675118	0.816314132	1.596660082	2.377006031
285753	5.428393874	7.0815531	3.775234698	4.22480309	6.076760471	5.150781781
285755	7.416850758	7.1059853	7.727716222	9.850577387	6.222321452	2.594065518
285758	2.481575183	3.5732463	1.389904029	0.701713319	1.167362396	1.633011474
285762	7.409706394	5.3166459	3.223585336	2.076022254	2.051108653	2.100935855
285800	4.230311308	6.4078822	5.31909676	-0.352108471	-0.352108471	-0.352108471
285847	8.978172924	6.502263	4.026353132	0.228373553	0.808855576	-0.352108471
285855	7.990348243	6.856185	9.124511468	6.584092692	7.69050115	8.796909607
285955	4.814358965	5.3835036	4.245214285	-0.352108471	-0.352108471	-0.352108471
285962	-0.352108471	-0.352108	-0.352108471	2.803874401	5.274309145	7.74474389
285965	7.163884331	8.0761926	7.620038487	4.927108276	5.392000621	5.856892965
285972	5.152247113	4.3506532	5.953841021	4.036683664	5.584371059	2.488996268
285987	6.618454158	5.8529375	5.087420765	1.043426334	1.646479888	0.440372779
286016	1.392109249	3.136327	-0.352108471	3.884413197	3.337746555	4.43107984
286042	-0.352108471	-0.352108	-0.352108471	1.473175732	2.072558294	2.671940856
286053	5.870109216	4.9866785	5.428393874	-0.352108471	-0.352108471	-0.352108471
286075	6.273832259	6.2858739	6.261790604	-0.352108471	-0.352108471	-0.352108471
286076	6.137515038	7.2704948	5.004535298	2.641840638	4.843308497	0.440372779
286101	5.285149151	6.2738323	7.262515367	4.158869237	3.337746555	4.979991919
286102	1.995832172	4.7994554	3.397643806	5.265298274	4.921497466	5.609099083
286122	1.006400681	2.8431725	4.679944359	-0.352108471	-0.352108471	-0.352108471

286144	6.997181405	6.6741289	6.351076353	-0.352108471	-0.352108471	-0.352108471
286148	7.108015359	5.5656007	8.650429976	3.765339163	7.143894276	10.52244939
286151	-0.352108471	-0.352108	-0.352108471	6.906299979	5.983736712	5.061173445
286183	-0.352108471	-0.352108	-0.352108471	0.51532816	3.552844027	2.034086093
286184	1.383520155	-0.352108	3.119148782	5.783882438	7.919651084	3.648113793
286186	3.692804111	5.4785362	7.264268277	5.285023717	5.393523004	5.502022292
286190	-0.352108471	-0.352108	-0.352108471	7.384563756	2.8168101	5.100686928
286205	9.363576856	9.6801122	9.521844524	7.015517182	9.071777868	8.043647525
286207	-0.352108471	-0.352108	-0.352108471	0.940233577	2.232575625	-0.352108471
286223	6.020381091	4.9392174	7.101544767	-0.352108471	-0.352108471	-0.352108471
286297	4.205053102	5.980334	7.755614954	-0.352108471	-0.352108471	-0.352108471
286336	-0.352108471	-0.352108	-0.352108471	5.887251126	6.189655896	6.038453511
286410	-0.352108471	-0.352108	-0.352108471	9.451973369	3.019868953	6.235921161
286437	10.80389404	10.600949	10.70242157	9.335445501	11.03245383	7.638437167
286467	5.172148081	4.3859368	5.958359385	2.368970958	2.686833264	2.051108653
286495	-0.352108471	-0.352108	-0.352108471	5.157865587	1.873234985	3.515550286
286827	6.599502875	5.5726159	7.626389892	5.637954374	6.66130097	4.614607778
317649	-0.352108471	-0.352108	-0.352108471	6.113850767	6.40631083	6.698770893
317662	-0.352108471	-0.352108	-0.352108471	2.124237496	2.147539136	2.100935855
317671	5.935349667	8.3960687	7.165709172	-0.352108471	-0.352108471	-0.352108471
317701	3.901586848	5.6588863	4.780236561	-0.352108471	-0.352108471	-0.352108471
317751	5.876276997	5.9200251	5.832528937	0.440372779	0.440372779	0.440372779
317781	7.922197336	7.3271516	6.732105789	5.871319513	5.771457123	5.971181903
319085	7.044122659	4.155091	5.59960682	0.791292145	0.440372779	1.14221151
326342	5.013952558	5.4904894	5.252220963	4.622246972	3.300477529	3.961362251
326625	2.844322884	7.4407737	5.142548317	1.610206098	2.169424975	2.728643851
327657	6.640019393	7.9739914	5.30604736	-0.352108471	-0.352108471	-0.352108471
333929	6.273832259	6.2858739	6.261790604	-0.352108471	-0.352108471	-0.352108471
337968	6.614624415	6.4247235	6.519673958	-0.352108471	-0.352108471	-0.352108471
338436	6.56437229	6.6211418	6.677911352	7.204147879	4.817190262	6.01066907
338442	2.419557138	1.8395576	2.99955665	-0.352108471	-0.352108471	-0.352108471
338557	4.462502061	5.344627	6.226751981	2.039485684	-0.352108471	4.43107984
338645	5.923573886	7.8061933	4.040954492	1.58479924	1.731739118	1.437859363
338653	-0.352108471	-0.352108	-0.352108471	5.233567424	4.481802969	5.985331878
338667	7.042523083	4.5763832	9.508662972	4.66005769	4.688318557	4.631796822
338699	7.730043418	3.2469034	5.488473394	0.228373553	-0.352108471	0.808855576
338739	4.485694886	4.9421496	4.713922221	0.727922146	-0.352108471	1.807952763
338761	6.121528769	6.1414231	6.101634422	-0.352108471	-0.352108471	-0.352108471
338773	5.249224703	5.2437595	5.254689875	2.469725738	2.686833264	2.252618212
338817	3.033771374	5.5927938	8.151816233	1.182147892	3.91110333	2.546625611
338949	6.640019393	7.0751355	6.204903312	-0.352108471	-0.352108471	-0.352108471
339005	6.144186222	4.4682884	7.820084091	5.550848618	5.53494234	5.542895479
339044	-0.352108471	-0.352108	-0.352108471	2.715466875	2.920869745	3.126272616
339059	6.588448077	6.0184744	6.303461245	0.440372779	0.440372779	0.440372779
339105	2.451290702	-0.352108	5.254689875	-0.352108471	-0.352108471	-0.352108471
339123	9.726501499	3.3868783	6.556689902	0.005072915	1.946552907	3.888032899
339145	7.178685391	3.1258088	5.152247113	3.381085654	4.954218546	1.807952763

339166	3.870214259	4.5873902	5.304566221	1.740870922	3.513926194	2.627398558
339175	6.655167767	7.0302053	6.280130204	5.130985206	7.722351631	6.426668418
339184	8.968434982	7.3774273	5.786419624	-0.352108471	-0.352108471	-0.352108471
339201	4.200971051	4.976206	4.58858854	6.51496239	3.618549613	9.411375168
339231	4.276366062	5.0235932	4.649979619	4.604264605	7.274729721	5.939497163
339240	3.27681682	2.3733834	2.8251001	-0.352108471	-0.352108471	-0.352108471
339263	5.126581771	5.4937832	5.860984659	5.829474075	5.7686356	5.890312551
339324	4.477687161	6.1383138	7.798940403	3.391527281	5.353437222	1.42961734
339398	5.577840935	6.2420483	6.906255623	-0.352108471	-0.352108471	-0.352108471
339416	7.611726253	7.0659821	6.520237918	1.245740716	2.051108653	0.440372779
339448	4.585494077	5.8774671	7.169440156	6.874487191	7.457478212	6.29149617
339451	6.967511245	6.8055026	7.129519899	-0.352108471	-0.352108471	-0.352108471
339483	-0.352108471	-0.352108	-0.352108471	5.668425445	7.431304437	3.905546453
339505	1.383520155	-0.352108	3.119148782	5.254689875	5.681192388	4.828187363
339529	-0.352108471	-0.352108	-0.352108471	1.543191936	2.049256654	2.555321371
339541	-0.352108471	-0.352108	-0.352108471	2.451290702	2.438991888	2.463589516
339674	2.553073296	5.4582551	-0.352108471	-0.352108471	-0.352108471	-0.352108471
339742	2.38621749	1.1025242	3.669910809	0.044132154	-0.352108471	0.440372779
339761	3.127737875	3.136327	3.119148782	1.434979174	1.939746065	0.930212283
339803	3.880357111	6.4148936	5.147625378	2.169424975	2.147539136	2.191310813
339942	8.711026868	5.226349	6.968687949	1.727216326	4.498327471	-1.04389482
339978	-0.352108471	-0.352108	-0.352108471	3.95685111	8.150431651	6.053641381
340017	4.049772207	3.8700402	3.690308212	1.454895716	2.100935855	0.808855576
340037	7.49297876	5.3384256	6.415702196	8.228727163	5.361327173	6.795027168
340061	-0.352108471	-0.352108	-0.352108471	7.99704284	3.582482612	5.789762726
340073	-0.352108471	-0.352108	-0.352108471	4.121011163	0.384225261	2.252618212
340075	6.697817748	6.2420483	5.78627881	4.178899946	4.245214285	4.112585606
340152	7.041446928	7.3790314	7.716615823	8.94690497	6.823749242	4.700593514
340273	6.818462795	6.4622499	6.106036957	5.775068216	4.705420536	6.844715896
340351	-0.099919723	2.5886014	5.277122618	-0.352108471	-0.352108471	-0.352108471
340371	-0.352108471	-0.352108	-0.352108471	6.735779686	6.750862801	6.720696571
340393	-0.352108471	-0.352108	-0.352108471	3.010220895	3.919505936	2.100935855
340481	-0.352108471	-0.352108	-0.352108471	7.925731627	5.745394463	3.565057299
340515	8.905428127	6.1455725	3.385716844	6.449221091	7.025789768	5.872652414
340533	-0.352108471	-0.352108	-0.352108471	5.958359385	5.958359385	5.958359385
340544	5.793507601	5.980334	6.167160455	-0.352108471	-0.352108471	-0.352108471
340554	5.238877031	4.7541814	5.723572646	4.931330546	3.337746555	6.524914536
340990	2.313941724	-0.352108	4.979991919	-0.352108471	-0.352108471	-0.352108471
341116	6.73041029	7.464791	5.996029598	5.636809111	8.474414172	2.799204049
341350	4.780758256	6.9251167	5.852937462	-0.352108471	-0.352108471	-0.352108471
341567	5.50246231	5.4283939	5.354325438	-0.352108471	-0.352108471	-0.352108471
341640	7.355359934	6.9008997	6.446439397	0.289051906	-0.352108471	0.930212283
341676	7.315389875	7.4989721	7.407180969	4.78551164	5.29703662	5.808561601
341880	2.588601447	-0.352108	5.529311366	-0.352108471	-0.352108471	-0.352108471
341883	-0.352108471	-0.352108	-0.352108471	4.406177525	3.432834346	3.919505936
342346	-0.352108471	-0.352108	-0.352108471	3.052703383	1.286146567	2.169424975
342510	5.595499658	6.1558333	5.035166055	4.435075177	3.615460478	5.254689875

342666	3.665625461	5.6251608	7.584696082	-0.352108471	-0.352108471	-0.352108471
342667	6.488500116	8.6699101	4.307090083	5.906610812	6.035359514	6.164108217
342892	8.207676013	8.3316688	8.083683208	6.171506005	7.50680485	8.842103695
342909	5.90029238	3.1465831	8.654001689	-0.352108471	-0.352108471	-0.352108471
342931	-0.352108471	-0.352108	-0.352108471	5.881400907	5.958359385	6.035317862
342945	8.913326013	8.7985774	9.028074588	6.980373291	6.698467242	7.26227934
343035	1.501017585	2.0673753	0.934659871	2.894004318	4.507115498	3.700559908
343071	6.151585628	5.3495815	6.953589722	-0.352108471	-0.352108471	-0.352108471
343099	-0.352108471	-0.352108	-0.352108471	4.80208491	5.732619672	3.871550149
343702	-0.352108471	-0.352108	-0.352108471	3.919505936	3.919505936	3.919505936
343990	5.718459767	5.767563	5.66935658	-0.352108471	-0.352108471	-0.352108471
344148	-0.352108471	-0.352108	-0.352108471	6.266367194	6.276856601	6.255877787
344167	5.284974356	5.5386607	5.03128799	-0.352108471	-0.352108471	-0.352108471
344558	-0.352108471	-0.352108	-0.352108471	4.166562338	7.412963114	5.789762726
345275	8.370773408	6.911093	7.640933204	6.339527978	5.305335104	7.373720851
345557	6.382873477	6.3885363	6.377210621	1.245740716	2.051108653	0.440372779
345778	8.470882879	7.0721259	5.673368997	4.570078915	6.270222109	7.970365302
345895	5.749402114	6.1636281	5.335176159	-0.352108471	-0.352108471	-0.352108471
346288	6.915292282	6.9037827	6.926801914	3.476124269	2.051108653	4.901139884
346389	9.64447658	8.1868367	6.729196837	7.255035575	9.879455564	4.630615586
346653	7.649685617	7.3519683	7.054251025	6.669574409	6.711909172	6.690741791
347487	8.332297558	5.7743703	3.21644308	-0.352108471	-0.352108471	-0.352108471
347688	2.451290702	2.4671772	2.435404203	-0.352108471	-0.352108471	-0.352108471
347689	-0.352108471	-0.352108	-0.352108471	6.415928975	6.178370298	6.653487652
347734	7.121700748	7.3241242	7.526547652	7.18608445	6.923117079	7.054600764
347735	2.15990226	4.671913	-0.352108471	2.881537061	4.954218546	0.808855576
347736	8.702558087	1.3190906	5.010824335	0.044132154	0.440372779	-0.352108471
347746	4.485691727	5.8654274	7.245162993	6.462887111	5.38675695	4.31062679
347862	8.933537848	7.4644335	10.40264219	8.453031314	7.737339536	9.168723093
347902	-0.352108471	-0.352108	-0.352108471	5.859617369	4.824058724	6.895176014
348013	5.089412202	2.3809248	7.797899595	-0.352108471	-0.352108471	-0.352108471
348094	4.431503209	4.9393624	5.447221659	0.044132154	0.440372779	-0.352108471
348110	6.757119757	6.4105853	7.103654244	4.432495672	3.119148782	5.745842563
348180	-0.352108471	-0.352108	-0.352108471	4.061959707	4.780236561	5.498513415
348235	6.093289078	5.9583594	6.228218771	1.796938968	0.440372779	3.153505157
348262	-0.352108471	-0.352108	-0.352108471	8.037412925	6.382873477	4.728334029
348654	-0.358485819	2.2010365	4.760558764	5.284974356	4.061574733	6.508373979
348761	-0.352108471	-0.352108	-0.352108471	2.703061684	1.127962105	4.278161263
349075	8.407090464	7.3900289	7.898559703	7.053015042	7.04373608	7.034457117
349114	3.994291929	4.5497284	5.105164781	6.178600791	7.626150042	9.073699293
349152	8.526484284	10.338137	9.432310685	8.182869201	9.796328561	6.56940984
349196	5.591305588	6.6876112	4.495000015	8.858862914	7.21467292	5.570482926
349408	5.817359515	4.9140225	6.720696571	0.95025487	2.252618212	-0.352108471
349667	-0.352108471	-0.352108	-0.352108471	1.054964352	9.968764191	5.511864271
353088	5.70586317	5.2437595	6.167966808	3.572335178	1.997577452	5.147092904
353091	-0.352108471	-0.352108	-0.352108471	4.465359657	2.108978996	6.821740319
353116	-0.352108471	-0.352108	-0.352108471	6.204656258	6.215464554	6.193847962

353132	6.967968685	3.8888191	5.428393874	-0.352108471	-0.352108471	-0.352108471
353135	-0.352108471	-0.352108	-0.352108471	4.791602495	5.393523004	5.995443514
353274	4.320213968	4.4826926	4.64517115	2.841536824	2.354923042	3.328150606
353324	-0.352108471	-0.352108	-0.352108471	0.897715333	-0.352108471	2.147539136
359710	4.792303669	4.8304259	4.754181416	-0.352108471	-0.352108471	-0.352108471
359822	5.394799792	7.6445481	6.519673958	-0.352108471	-0.352108471	-0.352108471
359845	7.799978222	7.0724845	7.436231365	7.888903002	7.939085645	7.838720358
360132	3.245235305	1.0177121	5.472758546	0.919601171	2.191310813	-0.352108471
373156	8.165812357	4.5725008	6.369156591	5.169157826	5.191222747	5.147092904
374291	7.885404347	6.7425639	7.313984131	6.829307464	5.363218811	8.295396116
374354	1.392109249	3.136327	-0.352108471	6.40631083	6.429470164	6.383151496
374378	8.680523657	6.6400194	4.599515128	0.440372779	0.440372779	0.440372779
374383	-0.352108471	-0.352108	-0.352108471	4.615990409	6.104429468	7.592868526
374393	6.672770329	7.9264733	5.41906734	1.493791735	1.437859363	1.549724107
374395	-0.352108471	-0.352108	-0.352108471	4.64557448	7.128427499	5.88700099
374491	9.562332258	6.2297216	2.897110969	-0.352108471	-0.352108471	-0.352108471
374654	-0.352108471	-0.352108	-0.352108471	2.19005738	2.232575625	2.147539136
374655	-0.352108471	-0.352108	-0.352108471	4.927679215	4.910392912	4.944965518
374666	8.75705839	6.7464901	4.735921722	5.390627963	6.769929259	6.080278611
374786	-0.352108471	-0.352108	-0.352108471	0.897715333	2.147539136	-0.352108471
374872	7.899526545	6.8372257	8.961827422	6.695682134	6.010880884	7.380483383
374875	7.673892538	2.7610897	5.217491139	-0.352108471	-0.352108471	-0.352108471
374882	-0.352108471	-0.352108	-0.352108471	3.136326969	3.451602177	2.821051762
374918	-0.352108471	-0.352108	-0.352108471	2.313941724	-0.352108471	4.979991919
374946	4.522024694	4.7482189	4.295830461	5.218487673	2.86382517	4.041156422
374969	-0.352108471	-0.352108	-0.352108471	6.396868383	5.174358637	7.619378128
374987	7.041242604	9.1457521	8.093497364	7.503651391	7.40365293	7.603649851
375033	6.10840822	6.8961178	6.502263028	5.954381829	7.140810338	8.327238847
375056	-0.352108471	-0.352108	-0.352108471	1.931562613	2.469725738	3.007888862
375133	5.439724472	5.825168	5.054280952	-0.352108471	-0.352108471	-0.352108471
375260	-0.352108471	-0.352108	-0.352108471	1.315841796	2.191310813	0.440372779
375316	2.565254368	2.6857321	2.806209807	-0.352108471	-0.352108471	-0.352108471
375341	2.39854798	4.3359198	6.273291662	6.43311159	2.980811522	4.706961556
375346	-0.352108471	-0.352108	-0.352108471	5.388940365	7.538409465	6.463674915
375449	3.636824788	3.9195059	3.354143641	3.73714403	3.919505936	3.554782125
375607	2.564903039	6.7737408	10.98257852	2.252618212	5.288688167	-0.783451744
375719	6.735779686	5.6002971	7.871262272	3.511085416	7.405424712	5.458255064
375743	-0.352108471	-0.352108	-0.352108471	5.760537073	6.140363555	5.380710591
375757	3.09617961	5.3835036	0.808855576	-0.352108471	-0.352108471	-0.352108471
375775	2.760618758	3.2496536	2.271583904	-0.352108471	-0.352108471	-0.352108471
375790	7.356612843	7.4168994	7.477186034	2.679906795	4.490335807	3.585121301
375940	5.489583732	5.2401543	5.739013132	3.14058393	4.907473824	1.373694036
376267	5.217491139	3.6138225	6.821159738	-0.352108471	-0.352108471	-0.352108471
376497	5.386086779	5.9757535	5.680920151	5.442167957	5.989336366	4.894999547
376940	-0.352108471	-0.352108	-0.352108471	3.278324455	2.920396231	3.099360343
377007	6.999048955	8.5482214	5.449876507	1.05704313	1.305230684	0.808855576
377677	6.341498931	6.1774288	6.505569015	5.798409829	5.830939431	5.863469032

378708	-0.352108471	-0.352108	-0.352108471	5.573088363	5.873882669	5.723485516
378884	-0.352108471	-0.352108	-0.352108471	4.412766833	3.585121301	2.757475769
378938	9.231166605	9.8495588	8.612774406	6.450640055	6.91500483	6.682822443
379013	5.334238245	6.0672699	4.601206613	3.060467316	5.369285173	7.67810303
379025	7.946946324	3.8536384	5.90029238	0.808855576	0.808855576	0.808855576
386597	6.754017386	5.825168	7.682866779	5.840199482	6.276856601	5.403542364
386680	5.625160771	5.6532998	5.597021732	-0.352108471	-0.352108471	-0.352108471
387032	4.731342239	7.6771131	1.785571376	5.37974618	6.034696055	5.707221118
387066	-0.352108471	-0.352108	-0.352108471	6.524513748	8.180960262	4.868067234
387129	5.749402114	6.1835825	5.315221752	-0.352108471	-0.352108471	-0.352108471
387254	8.165341174	8.4909014	7.83978098	6.689783396	7.044177561	6.33538923
387521	6.37092775	6.9451187	6.658023207	6.463674915	7.366860182	5.560489648
387522	4.749794444	7.2863246	9.82285485	8.556463087	8.522554666	8.539508877
387590	1.392109249	3.136327	-0.352108471	6.530787144	5.156185365	5.843486254
387628	-0.352108471	-0.352108	-0.352108471	5.12763925	4.401599365	5.853679134
387640	5.866795484	5.7752316	5.958359385	6.193241632	5.665786693	6.720696571
387644	5.013999274	6.1348006	7.255601956	-0.352108471	-0.352108471	-0.352108471
387680	-0.352108471	-0.352108	-0.352108471	6.022050925	4.800821405	7.243280445
387700	5.924443035	3.8926146	7.95627143	3.167132307	2.677192416	2.922162362
387715	6.991795048	6.9981067	6.985483415	-0.352108471	-0.352108471	-0.352108471
387723	4.215160124	5.2666288	3.163691446	3.305582051	2.024343053	0.743104054
387751	4.588632431	5.9079759	5.248304154	0.044132154	-0.352108471	0.440372779
387763	8.001538821	7.2774479	6.553356947	-0.352108471	-0.352108471	-0.352108471
387775	-0.352108471	-0.352108	-0.352108471	2.38621749	5.124543452	-0.352108471
387882	7.757984028	8.40528	7.110688051	8.620205222	8.834935583	9.049665945
387890	6.048656227	4.255838	5.152247113	-0.352108471	-0.352108471	-0.352108471
387893	7.472932879	7.4969383	7.520943807	-0.352108471	-0.352108471	-0.352108471
387921	6.433318728	6.4453648	6.421272681	-0.352108471	-0.352108471	-0.352108471
387978	4.965407552	6.0898692	5.527638398	3.59721797	3.298692349	3.895743592
388022	6.573968999	7.0550105	6.092927453	6.791733532	6.022050925	5.252368318
388121	4.544261391	4.1770731	4.911449657	6.32455531	7.795331321	4.853779299
388135	9.215787766	6.8576915	4.499595184	0.872801732	0.440372779	1.305230684
388152	-0.352108471	-0.352108	-0.352108471	4.422006664	4.691916222	4.961825779
388165	6.27334292	7.2976226	8.321902227	6.554330238	5.138271466	3.722212694
388228	9.417135542	7.3757444	5.334353338	6.747834851	4.394649049	9.101020653
388324	-0.352108471	-0.352108	-0.352108471	2.910985799	2.316018402	3.505953196
388325	4.26871449	4.617923	3.919505936	5.665568776	5.054280952	6.276856601
388419	-0.352108471	-0.352108	-0.352108471	3.309106943	4.178899946	5.048692948
388507	6.629948642	5.8445443	7.415353029	8.685420125	5.430149742	2.174879358
388524	-0.352108471	-0.352108	-0.352108471	5.902763525	5.930745839	5.916754682
388550	7.319411632	9.8820533	8.600732457	6.622651635	6.742736775	6.862821915
388561	6.260458727	6.3228896	6.198027805	1.622906063	1.807952763	1.437859363
388566	5.931810751	4.2476963	7.615925233	0.228373553	-0.352108471	0.808855576
388567	6.184820943	4.7351884	7.634453473	2.555275869	7.285397695	4.920336782
388574	7.807259768	6.6766342	5.546008598	1.796938968	3.058439447	0.535438489
388610	3.198776722	3.056699	3.127737875	8.025088431	8.143182825	7.906994037
388630	5.428393874	7.9666038	2.890183904	-0.352108471	-0.352108471	-0.352108471

388685	4.363953422	5.5304556	3.197451278	1.845238787	2.252618212	1.437859363
388697	4.875457945	5.5595243	5.217491139	5.359431903	6.843135165	8.326838427
388698	4.679717253	5.7740431	6.868369027	2.216380688	2.166656576	2.116932463
388714	6.382873477	6.4105853	6.355161685	-0.352108471	-0.352108471	-0.352108471
388753	5.499998698	6.8018138	8.10362885	-0.352108471	-0.352108471	-0.352108471
388759	10.71585968	8.4119555	6.10805132	5.782387296	8.512487973	7.147437635
388789	6.656125064	6.6538708	6.658379305	0.044132154	0.440372779	-0.352108471
388910	-0.352108471	-0.352108	-0.352108471	3.067782853	4.178899946	5.290017039
388960	5.55876787	5.3064074	5.811128349	-0.352108471	-0.352108471	-0.352108471
388962	-0.352108471	-0.352108	-0.352108471	5.923493182	5.926961306	5.920025058
389015	10.66586667	9.9918964	9.317926137	8.747524246	8.974741184	9.201958122
389072	6.698034342	5.3258163	8.070252354	2.266276845	1.885767663	2.076022254
389114	7.811489345	7.6453965	7.479303716	8.244065921	6.353429101	4.462792281
389119	7.137806146	3.4321426	5.284974356	-0.352108471	-0.352108471	-0.352108471
389136	-0.352108471	-0.352108	-0.352108471	3.253610347	7.626224528	5.439917437
389152	4.753865155	6.7541357	2.753594626	1.394345699	1.14221151	1.646479888
389197	7.57364657	5.1646666	6.369156591	-0.352108471	-0.352108471	-0.352108471
389206	-0.352108471	-0.352108	-0.352108471	2.519010526	5.325716078	8.13242163
389400	2.413921152	5.1799508	-0.352108471	0.624614178	0.440372779	0.808855576
389421	7.112120745	7.5624965	6.661744953	-0.352108471	-0.352108471	-0.352108471
389432	6.975263409	6.5711063	6.166949167	5.621110071	6.269885154	4.972334989
389493	3.368266788	2.065292	0.7623173	2.258253426	1.791107639	2.024680532
389524	5.124543452	6.9431196	3.305967298	-0.352108471	-0.352108471	-0.352108471
389538	7.059808236	5.6251608	4.190513307	-0.352108471	-0.352108471	-0.352108471
389541	-0.352108471	-0.352108	-0.352108471	8.873628679	5.906733668	2.939838657
389610	2.448539722	1.7836987	1.118857742	3.222716608	1.157398153	2.19005738
389630	-0.352108471	-0.352108	-0.352108471	4.279634776	5.493783215	6.707931654
389641	6.396586133	6.1999612	6.593211042	6.236695219	6.515031054	5.958359385
389643	5.479009326	7.1421903	8.805371273	6.676379606	7.77424412	5.578515091
389649	8.168574184	7.3237146	6.478854952	1.75605734	5.915852686	10.07564803
389668	9.779340729	9.4665024	9.153664131	8.415109911	7.066723123	9.763496699
389715	2.84317252	4.1587613	1.527583775	-0.352108471	-0.352108471	-0.352108471
389741	-0.352108471	-0.352108	-0.352108471	5.541826516	4.64902146	5.095423988
389763	6.573968999	7.584828	5.563110022	-0.352108471	-0.352108471	-0.352108471
389791	7.33996113	9.8328566	4.847065677	7.371735579	5.886743097	4.401750616
389792	8.316249499	8.4650242	8.167474822	4.833992389	5.723485516	6.612978643
389813	-0.352108471	-0.352108	-0.352108471	5.679357672	4.803908551	5.241633112
389834	4.103052932	8.408891	6.255971957	3.366593439	5.412561087	7.458528736
389941	-0.352108471	-0.352108	-0.352108471	5.575983727	7.574097512	3.577869941
390066	-0.352108471	-0.352108	-0.352108471	6.818185735	5.284974356	3.751762977
390079	6.373808137	6.9744496	6.674128879	-0.352108471	-0.352108471	-0.352108471
390142	7.772618403	4.2681438	6.020381091	-0.352108471	-0.352108471	-0.352108471
390157	-0.352108471	-0.352108	-0.352108471	9.270304069	2.372517607	5.821410838
390226	3.662601802	2.3139417	0.965281646	-0.352108471	-0.352108471	-0.352108471
390231	9.008734332	3.3591937	6.183964016	-0.352108471	-0.352108471	-0.352108471
390275	5.635826733	5.220961	5.428393874	-0.352108471	-0.352108471	-0.352108471
390539	-0.352108471	-0.352108	-0.352108471	0.897715333	2.147539136	-0.352108471

390756	5.625160771	5.7206083	5.529713276	-0.352108471	-0.352108471	-0.352108471
390874	-0.352108471	-0.352108	-0.352108471	-0.312499197	1.995427359	4.303353916
390963	8.605093366	7.5773131	6.549532778	6.509185427	6.391863632	6.626507221
390980	9.457492045	8.5534723	10.36151175	8.391506486	8.007595937	8.199551212
391627	-0.352108471	-0.352108	-0.352108471	2.38621749	5.124543452	-0.352108471
392196	-0.352108471	-0.352108	-0.352108471	5.392083658	4.463274772	4.927679215
392862	-0.352108471	-0.352108	-0.352108471	2.38621749	5.124543452	-0.352108471
399473	5.962072235	9.0309213	7.496496758	4.158869237	5.709659516	2.608078959
399664	-0.352108471	-0.352108	-0.352108471	5.712263427	4.304918356	7.119608499
399665	-0.352108471	-0.352108	-0.352108471	3.606517346	5.336173319	1.876861374
399746	5.428393874	5.3775666	5.479221111	-0.352108471	-0.352108471	-0.352108471
399761	7.906244879	8.0182569	8.130269011	2.820330831	5.780693203	8.741055575
399814	-0.352108471	-0.352108	-0.352108471	2.295316925	4.877311541	7.459306156
399823	-0.352108471	-0.352108	-0.352108471	9.656491579	6.022050925	2.387610271
399844	4.201099247	4.4826926	3.919505936	2.424859109	3.919505936	0.930212283
399881	-0.352108471	-0.352108	-0.352108471	2.239979033	3.337746555	1.14221151
399886	1.480940338	1.2861	1.383520155	2.217487623	2.118278685	2.019069748
399900	-0.352108471	-0.352108	-0.352108471	5.564420131	5.005528582	5.284974356
399909	-0.352108471	-0.352108	-0.352108471	7.072591202	5.308348614	3.544106026
399923	7.891723217	8.1005797	7.682866779	6.900179493	6.720696571	7.079662414
399947	-0.352108471	-0.352108	-0.352108471	3.680127125	3.392883967	3.536505546
399948	6.142386792	4.5685245	5.355455665	-0.352108471	-0.352108471	-0.352108471
399959	4.238176943	5.4275895	6.617002076	6.12990004	7.41376259	4.84603749
399968	-0.352108471	-0.352108	-0.352108471	1.70308327	1.305230684	2.100935855
399979	-0.352108471	-0.352108	-0.352108471	7.371677012	7.555244212	7.188109812
400002	9.88683101	7.3398659	8.613348453	7.994024596	7.103411138	6.21279768
400121	5.600886829	10.190542	7.895714382	6.695905193	6.662724824	6.729085562
400212	9.016534868	8.8378292	9.195240516	7.31878593	7.718422267	8.118058603
400322	-0.352108471	-0.352108	-0.352108471	2.969210976	2.451290702	1.933370429
400456	2.289786469	0.494432	1.392109249	1.190059422	1.939746065	0.440372779
400506	-0.352108471	-0.352108	-0.352108471	4.800358203	6.842463472	5.821410838
400508	5.316006598	5.4275895	5.539172421	2.676064501	0.605398724	1.640731613
400511	6.569257994	6.7355253	6.402990729	1.515574069	2.100935855	0.930212283
400548	1.169379371	1.5010176	1.832655798	4.834410909	5.12763925	5.420867591
400612	5.645995832	6.1507978	6.655599813	6.910262313	5.154689539	3.399116766
400618	5.869947055	4.513304	3.15666101	1.290035436	1.437859363	1.14221151
400644	7.428545491	7.1481148	6.867684036	6.260418718	6.114415754	5.968412789
400655	5.128962071	3.7642422	6.493681965	5.168494212	6.753916436	3.583071987
400657	4.867086667	4.9799919	4.754181416	5.460434222	5.605609608	5.315258837
400661	-0.352108471	-0.352108	-0.352108471	2.546513039	3.144652207	3.742791374
400682	6.173779995	6.5150311	5.832528937	5.852120102	5.665786693	6.038453511
400706	5.633353739	5.7752316	5.917109427	4.02845604	3.494843976	4.562068104
400745	-0.352108471	-0.352108	-0.352108471	6.679894429	4.841179717	5.760537073
400748	5.854321977	6.5707504	6.212536209	-0.352108471	-0.352108471	-0.352108471
400765	-0.352108471	-0.352108	-0.352108471	1.400698343	-0.352108471	3.153505157
400798	5.240497058	4.4585636	3.676630217	5.637895348	5.665251835	5.692608323
400804	5.317370559	5.3733719	5.261369182	8.212531249	3.391602615	5.802066932

400818	4.578801146	2.8888006	6.268801658	-0.352108471	-0.352108471	-0.352108471
400870	5.625160771	5.6532998	5.597021732	-0.352108471	-0.352108471	-0.352108471
400892	10.77262473	5.0114009	7.892012834	2.146123334	2.100935855	2.191310813
400931	8.150175441	8.0660974	8.234253481	4.231032423	2.686833264	5.775231583
400941	8.890478571	6.9675112	5.044543919	1.76984594	1.807952763	1.731739118
400950	8.261905243	7.2592056	9.264604849	9.217452168	7.556075917	5.894699666
400955	4.803618005	2.8367941	6.770441956	0.044132154	-0.352108471	0.440372779
400958	8.009356603	6.5108061	9.507907069	5.485123609	6.382521369	4.587725849
400961	-0.352108471	-0.352108	-0.352108471	4.178899946	6.049178256	2.308621635
401010	-0.352108471	-0.352108	-0.352108471	4.653681558	6.867392588	5.760537073
401014	5.017136436	5.054281	4.979991919	-0.352108471	-0.352108471	-0.352108471
401024	5.800199788	4.4099411	7.190458438	-0.352108471	-0.352108471	-0.352108471
401039	3.894362219	3.6761388	4.112585606	4.248750405	5.233567424	6.218384442
401052	7.244863824	7.1815515	7.118239191	5.579072611	6.747774898	6.163423754
401081	-0.352108471	-0.352108	-0.352108471	5.169157826	7.148437129	3.189878523
401082	4.922996633	5.8432369	6.763477202	8.063390227	6.095756386	7.079573307
401089	5.015650258	4.6339339	5.397366655	6.594402602	5.537609977	4.480817351
401093	-0.352108471	-0.352108	-0.352108471	5.903760504	7.015128477	4.792392531
401105	6.000247872	6.6020516	5.398444155	1.518484901	1.731739118	1.305230684
401152	-0.395259066	1.3835202	3.162299376	4.175292888	5.366700028	2.983885747
401176	-0.352108471	-0.352108	-0.352108471	6.003978506	6.002308672	6.005648339
401232	5.824288785	5.873346	5.775231583	4.967105233	4.954218546	4.979991919
401233	7.521934454	7.1829284	7.860940545	1.917379553	2.242596918	2.567814284
401242	9.008306653	7.2202022	5.432097798	3.805152612	5.359400601	6.913648589
401247	6.475702667	5.1238684	5.79978551	0.831589977	2.631888258	1.731739118
401250	-0.352108471	-0.352108	-0.352108471	5.369332089	5.55529679	5.183367387
401260	6.829815208	5.9194595	5.009103878	2.102425001	3.245625856	4.388826711
401289	5.800199788	8.5657553	3.034644242	-0.352108471	-0.352108471	-0.352108471
401317	-0.352108471	-0.352108	-0.352108471	4.381734268	5.128763636	5.875793003
401320	6.415928975	6.4105853	6.421272681	3.086062074	3.919505936	2.252618212
401321	3.053533646	7.6920338	5.372783739	4.171144437	4.971504216	5.771863995
401324	5.82391272	5.646455	5.735183859	1.158617076	0.440372779	1.876861374
401335	4.890080051	6.1919092	5.540994634	1.835742592	1.939746065	1.731739118
401357	5.602389337	7.86917	6.735779686	4.818774833	6.001809131	7.18484343
401397	-0.352108471	-0.352108	-0.352108471	3.022824329	4.103893054	5.18496178
401492	4.942352457	4.6711825	4.806767494	2.096182139	2.252618212	1.939746065
401505	-0.352108471	-0.352108	-0.352108471	5.041497906	7.430344416	6.235921161
401508	5.438743023	5.0208482	5.229795633	1.540978788	1.14221151	1.939746065
401548	6.674128879	4.9710533	8.377204506	-0.352108471	-0.352108471	-0.352108471
401554	5.564591206	6.2858739	4.843308497	0.685292531	0.440372779	0.930212283
401557	-0.352108471	-0.352108	-0.352108471	7.281527317	6.799585347	7.040556332
401563	4.872224191	5.3447363	4.399712117	-0.352108471	-0.352108471	-0.352108471
401588	1.880238567	4.1125856	-0.352108471	1.964002179	3.119148782	0.808855576
401612	6.18045734	5.5954997	5.010541977	2.513628242	1.873849414	1.234070586
401884	4.731342239	4.9799919	4.482692559	-0.352108471	-0.352108471	-0.352108471
401944	2.016914154	4.5203272	-0.486498867	2.534742836	2.828965351	2.240520322
402381	-0.352108471	-0.352108	-0.352108471	2.686833264	2.686833264	2.686833264

402415	4.504224559	9.099403	6.801813774	1.075127527	2.211943219	3.348758911
402483	5.91576024	6.5251953	5.306325132	6.287192184	7.029403463	6.658297824
402665	-0.352108471	-0.352108	-0.352108471	6.745648015	5.257970247	6.001809131
403150	7.94321586	7.7257533	7.834484571	6.498934874	6.325024587	6.672845162
403312	7.946261914	6.2345125	4.522763055	5.263851904	5.315258837	5.212444972
403315	2.79997354	4.8345008	6.869028154	6.000222335	8.000684573	3.999760097
403340	-0.352108471	-0.352108	-0.352108471	6.392315925	7.547757663	5.236874187
404093	-0.352108471	-0.352108	-0.352108471	5.916099984	5.530871047	5.723485516
404216	5.253240792	5.209544	5.296937619	-0.352108471	-0.352108471	-0.352108471
404217	3.527916452	3.136327	3.919505936	5.637296679	5.653299811	5.621293547
404636	-0.352108471	-0.352108	-0.352108471	1.90830372	1.939746065	1.876861374
404663	8.210570338	6.4503028	4.690035337	0.044132154	-0.352108471	0.440372779
404672	9.266421739	6.3219796	7.794200678	9.756800082	5.602674288	7.679737185
404770	6.310474709	5.6298125	6.991136904	4.595573415	5.575983727	6.556394038
407835	6.285816951	7.4941959	6.890006441	5.502551577	6.68988391	4.315219244
414149	8.720252597	10.147192	7.293313201	7.803717181	7.731338116	7.876096247
414152	5.55876787	5.5882244	5.529311366	-0.352108471	-0.352108471	-0.352108471
414194	6.746243716	5.4434049	4.140566173	-0.352108471	-0.352108471	-0.352108471
414243	8.79574336	2.9101316	5.852937462	-0.352108471	-0.352108471	-0.352108471
414245	9.095909055	3.8917756	6.493842351	8.095024939	6.206574219	4.318123499
414328	2.313941724	-0.352108	4.979991919	-0.352108471	-0.352108471	-0.352108471
414332	7.164576595	4.9470072	2.729437784	0.689815323	-0.352108471	1.731739118
414777	5.75176029	4.8517347	3.951709059	4.450744817	3.337746555	5.563743079
414919	-0.352108471	-0.352108	-0.352108471	6.75496194	4.722164596	8.787759284
415116	-0.352108471	-0.352108	-0.352108471	6.913488898	3.233288032	10.59368976
439921	6.265637698	8.559616	7.412626846	7.64761654	3.887551224	11.40768186
439931	-0.352108471	-0.352108	-0.352108471	0.851977332	2.167095551	1.509536442
439990	5.55876787	4.2630676	6.854468115	5.958359385	5.455011096	6.461707673
440028	-0.352108471	-0.352108	-0.352108471	2.012200255	2.614704452	1.409696058
440041	-0.352108471	-0.352108	-0.352108471	2.732772606	-0.352108471	5.817653684
440044	5.238877031	7.6546039	2.823150182	0.395051519	1.14221151	-0.352108471
440072	2.697410991	6.4427072	4.570059097	0.598807818	1.549724107	-0.352108471
440101	1.880238567	-0.352108	4.112585606	4.812363898	5.525951753	5.169157826
440104	-0.352108471	-0.352108	-0.352108471	1.031973476	4.774008569	2.902991023
440119	5.379561447	4.5671969	3.754832449	1.416910214	1.290035436	1.163160659
440145	-0.352108471	-0.352108	-0.352108471	5.739083926	6.00432218	6.269560434
440173	7.717613268	8.9785204	8.34806685	7.250260926	7.458340638	7.042181214
440193	6.32763073	4.9116502	3.495669687	0.044132154	-0.352108471	0.440372779
440224	-0.352108471	-0.352108	-0.352108471	7.249045245	6.975623316	6.702201386
440253	5.359791817	6.5678645	7.77593713	7.47489242	7.342696275	7.607088565
440275	4.29742836	4.0160458	3.734663182	7.660007451	7.669038175	7.664522813
440279	-0.352108471	-0.352108	-0.352108471	5.903760504	4.41560128	7.391919729
440299	5.019282955	4.9140225	5.124543452	3.477522833	1.807952763	5.147092904
440300	10.71086806	9.073785	7.436701886	7.371347861	9.269694946	8.320521403
440346	6.195655283	6.4376768	6.679698396	6.687357735	4.774212626	5.730785181
440348	-0.352108471	-0.352108	-0.352108471	8.072203794	6.159662462	4.247121129
440390	1.66985715	-0.352108	3.69182277	1.995427359	2.051108653	1.939746065

440400	4.615583513	4.671913	4.559254036	7.42546171	7.35252903	7.49839439
440416	7.198251027	5.9158527	4.633454344	1.880238567	-0.352108471	4.112585606
440435	-0.352108471	-0.352108	-0.352108471	6.128867033	6.235921161	6.342975288
440446	7.269123066	6.889091	7.649155172	7.089332346	7.571326142	8.053319937
440456	-0.352108471	-0.352108	-0.352108471	6.117607993	6.496434318	5.738781667
440465	5.474014689	6.4527052	4.495324183	5.246137506	7.517055054	2.975219957
440482	-0.352108471	-0.352108	-0.352108471	7.057326175	5.774531775	6.415928975
440498	-0.352108471	-0.352108	-0.352108471	3.867053136	5.663737322	7.460421508
440503	5.980334028	7.7496405	4.211027585	-0.352108471	-0.352108471	-0.352108471
440508	4.978718278	5.4199399	5.861161521	5.035252831	4.20826195	3.381271069
440515	4.892666879	5.9391922	6.985717564	6.6049373	6.675432496	6.534442104
440533	2.736529761	2.3742856	3.098773939	1.509536442	-0.265997875	3.285070759
440556	6.008490517	4.5611367	5.284813592	-0.352108471	-0.352108471	-0.352108471
440563	-0.352108471	-0.352108	-0.352108471	6.130718295	5.243942167	4.35716604
440574	6.465214847	5.2689741	4.072733255	5.974328953	6.022050925	6.069772897
440594	8.460343176	8.943954	7.976732366	9.024637121	6.0713306	7.54798386
440603	6.451340363	6.7620481	7.072755785	7.253162567	6.736797698	6.994980132
440712	5.249224703	4.3097843	6.188665068	-0.352108471	-0.352108471	-0.352108471
440822	6.090907831	5.9200251	6.261790604	3.367253076	5.804293869	0.930212283
440862	-0.352108471	-0.352108	-0.352108471	5.663737322	5.665786693	5.661687951
440894	4.288787513	0.2731265	2.280956994	-0.352108471	-0.352108471	-0.352108471
440896	5.835482903	7.4759394	6.655711164	3.248686021	1.14221151	5.355160532
440900	-0.352108471	-0.352108	-0.352108471	0.95025487	2.252618212	-0.352108471
440905	7.049402551	1.6162358	4.332819167	-0.352108471	-0.352108471	-0.352108471
440926	4.780455428	4.4649182	4.622686795	-0.352108471	-0.352108471	-0.352108471
440934	-0.352108471	-0.352108	-0.352108471	2.076022254	2.100935855	2.051108653
440952	-0.352108471	-0.352108	-0.352108471	3.866119728	3.919505936	3.972892143
440970	7.079845576	5.5738939	4.067942198	2.95889416	2.978133346	2.968513753
441072	8.299524492	8.3501449	8.248904043	6.130436808	6.515031054	5.745842563
441081	6.407087129	4.8473688	3.28765039	4.54270355	4.559699198	4.576694846
441087	6.275785213	7.2865519	5.26501854	6.097037437	5.597636691	6.596438183
441089	-0.352108471	-0.352108	-0.352108471	5.183235465	5.605609608	4.760861323
441151	5.92941891	4.5770627	5.253240792	-0.352108471	-0.352108471	-0.352108471
441178	4.792303669	6.4883157	3.096291623	-0.352108471	-0.352108471	-0.352108471
441208	-0.352108471	-0.352108	-0.352108471	6.29936863	5.760537073	5.221705516
441234	5.980334028	4.7321481	7.228519914	1.954444309	2.100935855	1.807952763
441242	6.853636264	5.3075412	8.399731302	-0.352108471	-0.352108471	-0.352108471
441250	-0.352108471	-0.352108	-0.352108471	5.821410838	5.825167992	5.817653684
441272	-0.352108471	-0.352108	-0.352108471	2.747264095	0.932208597	4.562319593
441317	3.322915003	2.7402102	2.157505462	-0.352108471	-0.352108471	-0.352108471
441327	2.960303223	3.8349844	3.397643806	7.560644097	2.543342009	5.051993053
441376	8.055751488	3.1064917	5.581121573	1.14221151	1.14221151	1.14221151
441425	-0.352108471	-0.352108	-0.352108471	7.646869435	5.859617369	4.072365303
441457	-0.352108471	-0.352108	-0.352108471	4.138026527	8.43308239	6.285554459
441478	-0.352108471	-0.352108	-0.352108471	6.396868383	7.499096019	5.294640746
441518	-0.352108471	-0.352108	-0.352108471	6.3785581	6.957743639	6.668150869
441531	5.724668212	5.5293114	5.920025058	-0.352108471	-0.352108471	-0.352108471

441549	-0.352108471	-0.352108	-0.352108471	0.897715333	2.147539136	-0.352108471
441666	1.946552907	1.2795148	2.613591055	5.225663259	8.446764378	6.836213819
441728	5.745394463	5.7155573	5.775231583	-0.352108471	-0.352108471	-0.352108471
441733	8.854223243	8.5765228	8.715372997	10.39091511	5.888594706	8.139754909
441869	4.401382469	7.6351311	6.018256787	-0.352108471	-0.352108471	-0.352108471
441925	-0.352108471	-0.352108	-0.352108471	5.284974356	5.315258837	5.254689875
441956	2.566510924	7.0180964	4.792303669	1.223721097	1.14221151	1.305230684
442075	7.737175083	5.3554557	2.973736247	5.696259122	5.463827095	5.928691148
442132	4.692989119	4.7541814	4.631796822	5.27319164	5.191222747	5.355160532
442245	-0.352108471	-0.352108	-0.352108471	2.313941724	-0.352108471	4.979991919
442421	6.967173867	6.2604587	5.553743587	5.543764327	7.027226205	6.285495266
442578	1.392109249	3.136327	-0.352108471	2.710182349	0.440372779	4.979991919
442582	4.758495381	4.6735474	4.843443409	0.872801732	1.305230684	0.440372779
445372	2.73125229	2.5156976	2.300142884	-0.352108471	-0.352108471	-0.352108471
492311	-0.352108471	-0.352108	-0.352108471	3.245625856	2.742114909	3.749136803
493812	7.590434087	9.2564113	5.924456828	6.650923964	7.381083215	8.111242466
493829	5.947877441	4.4654144	2.982951266	0.624614178	0.440372779	0.808855576
493856	-0.352108471	-0.352108	-0.352108471	6.479260377	6.497336045	6.461184708
493869	7.599139544	7.763401	7.434878062	6.698753019	6.821025802	6.576480236
493901	-0.352108471	-0.352108	-0.352108471	6.311104816	5.821410838	5.33171686
494115	-0.352108471	-0.352108	-0.352108471	-0.477659345	2.146123334	4.769906013
494551	-0.352108471	-0.352108	-0.352108471	7.669671459	6.076418555	6.873045007
497048	2.419557138	1.7486918	3.090422524	-0.352108471	-0.352108471	-0.352108471
497259	2.783958294	2.7854929	2.7824237	5.980334028	6.481737176	5.47893088
497661	-0.352108471	-0.352108	-0.352108471	5.430898714	6.984596062	3.877201366
503519	-0.352108471	-0.352108	-0.352108471	6.204656258	6.215464554	6.193847962
503569	-0.352108471	-0.352108	-0.352108471	5.512443055	2.845356836	4.178899946
503582	8.222591862	6.9129332	7.567762534	6.468204264	5.503230866	5.985717565
503693	7.443574605	8.6633136	6.223835606	5.959771619	3.181248298	8.73829494
503835	6.705287512	6.7206966	6.689878453	6.753165957	6.515031054	6.991300859
541468	5.958359385	5.9583594	5.958359385	0.044132154	0.440372779	-0.352108471
541471	0.964314959	4.3368764	2.65059567	-0.352108471	-0.352108471	-0.352108471
548644	5.185586761	0.8561052	9.515068282	0.044132154	-0.352108471	0.440372779
550112	4.189015174	3.8056644	3.422313532	4.435867475	2.264739913	3.350303694
552889	-0.352108471	-0.352108	-0.352108471	7.156991566	6.541009048	5.925026529
553103	5.354685649	6.1144923	4.594878997	-0.352108471	-0.352108471	-0.352108471
554203	7.408889715	5.885114	8.9326654	3.992127367	9.438720113	6.71542374
554236	-0.352108471	-0.352108	-0.352108471	4.187631723	4.726140676	4.456886199
574406	5.737776816	4.9355089	6.540044767	4.675488217	2.330562372	3.503025295
574414	-0.352108471	-0.352108	-0.352108471	-1.26097585	4.217370563	1.478197356
595135	8.065643396	7.5606176	7.055591778	3.746501554	9.465601696	6.606051625
606495	-0.352108471	-0.352108	-0.352108471	2.169424975	2.191310813	2.147539136
619189	-0.352108471	-0.352108	-0.352108471	1.33427356	2.233123905	1.783698732
619190	7.02496035	7.7173979	8.409835477	8.625399705	6.621760009	7.623579857
619208	1.383520155	-0.352108	3.119148782	1.400698343	-0.352108471	3.153505157
619434	-0.352108471	-0.352108	-0.352108471	4.178899946	3.217705361	5.14009453
641364	7.518191577	6.199679	4.881166475	3.064586224	2.948100703	3.181071745

641455	4.977484687	4.9347306	5.020238824	0.762376451	-0.352108471	1.876861374
641467	4.909068675	3.8188382	4.363953422	1.824963332	2.376908379	2.100935855
641515	4.783083047	9.3999115	7.091497261	0.440372779	0.440372779	0.440372779
641638	3.850179204	5.9002924	7.950405556	-0.352108471	-0.352108471	-0.352108471
641737	5.762940099	6.2575232	6.752106345	0.189461371	3.890964789	7.592468206
641977	6.050658323	8.7601081	3.341208537	4.731342239	5.661419446	3.801265032
642236	5.965476118	5.2083363	5.586906204	3.033522536	3.027193952	3.03985112
642273	-0.352108471	-0.352108	-0.352108471	5.758337747	3.561777633	4.66005769
642280	-0.352108471	-0.352108	-0.352108471	3.373910559	5.605609608	1.14221151
642366	-0.352108471	-0.352108	-0.352108471	5.716728623	2.43016825	-0.856392123
642426	-0.352108471	-0.352108	-0.352108471	4.617382386	1.958013259	7.276751513
642441	-0.352108471	-0.352108	-0.352108471	1.438512382	2.245600013	3.052687645
642587	-0.352108471	-0.352108	-0.352108471	2.064739793	0.339265573	3.790214012
642633	5.409152593	8.2692003	6.839176453	7.07796731	6.641725768	6.205484227
642648	-0.352108471	-0.352108	-0.352108471	6.411416545	5.412561087	4.41370563
642757	5.146252583	4.9013721	5.023812365	1.043426334	0.440372779	1.646479888
642826	7.591987731	7.2999875	7.007987186	1.124162771	0.440372779	1.807952763
642852	6.257046343	6.8013513	5.712741385	-0.352108471	-0.352108471	-0.352108471
642934	8.223525854	6.3495694	4.475612938	1.889059667	0.608284395	3.16983494
642943	6.369253574	5.0079917	5.688622647	4.842884858	3.567505139	6.118264577
642968	-0.352108471	-0.352108	-0.352108471	5.663737322	6.000546358	5.326928286
643008	4.086371885	3.9195059	3.752639986	3.235271764	6.65015065	-0.179607123
643155	-0.352108471	-0.352108	-0.352108471	6.77374078	4.822028375	8.725453184
643224	5.832003073	6.27194	6.051971547	-0.352108471	-0.352108471	-0.352108471
643314	7.32386522	7.352529	7.29520141	5.514960729	5.775231583	5.254689875
643355	7.17777694	7.472869	6.882684902	-0.352108471	-0.352108471	-0.352108471
643382	-0.352108471	-0.352108	-0.352108471	4.178899946	5.938081493	2.419718398
643387	8.398709786	7.1960989	5.993487954	9.960173286	0.286677576	5.123425431
643401	4.940612622	4.4826926	5.398532684	1.179289842	0.808855576	1.549724107
643406	11.95044425	11.880564	11.91550423	10.68598581	12.50763286	8.864338755
643432	7.584050702	8.4156218	9.247192946	6.5504786	9.225232224	7.887855412
643623	6.722637499	6.9300962	7.137554879	0.047280654	3.708097346	7.368914038
643677	6.394354872	6.2125362	6.030717546	-0.352108471	-0.352108471	-0.352108471
643714	-0.352108471	-0.352108	-0.352108471	6.279247891	6.052493321	6.165870606
643763	8.223995653	8.3885462	8.553096752	6.747005247	6.841838197	6.794421722
643770	5.577957687	5.2286715	5.927243842	2.879532747	1.646479888	4.112585606
643827	-0.352108471	-0.352108	-0.352108471	1.880238567	-0.352108471	4.112585606
643836	-0.352108471	-0.352108	-0.352108471	4.860625611	6.463674915	8.066724219
643837	6.573968999	5.8635039	7.284434123	-0.352108471	-0.352108471	-0.352108471
643904	8.563200932	7.3679106	6.172620288	5.333243483	7.225269909	3.441217057
643962	3.662109543	3.8056644	3.949219163	7.447036241	5.816656262	6.631846251
643988	-0.352108471	-0.352108	-0.352108471	1.873849414	2.294368503	1.453330325
644050	5.852937462	6.7349538	4.970921153	-0.352108471	-0.352108471	-0.352108471
644128	8.320541927	8.1459218	8.495162014	5.714752974	7.209435678	8.704118382
644150	1.553627006	1.7836987	2.013770458	1.783698732	3.919505936	-0.352108471
644215	-0.352108471	-0.352108	-0.352108471	1.970890817	2.801544164	2.38621749
644285	4.098488742	5.980334	7.862179314	0.872801732	0.440372779	1.305230684

644316	5.200714372	5.973098	5.586906204	-0.352108471	-0.352108471	-0.352108471
644538	6.303461245	6.3152311	6.291691359	-0.352108471	-0.352108471	-0.352108471
644660	6.317340387	5.4539948	4.590649299	6.253169245	6.720244285	5.786094205
644669	1.383520155	-0.352108	3.119148782	0.762376451	-0.352108471	1.876861374
644762	-0.352108471	-0.352108	-0.352108471	3.998406827	3.860015046	4.136798608
644903	-0.352108471	-0.352108	-0.352108471	3.245625856	2.998874658	3.492377054
644919	3.3436831	7.5495947	5.446638914	5.36792541	3.743939131	4.555932271
644936	4.676859199	4.0130283	3.349197432	1.968661759	1.997577452	1.939746065
644943	-0.352108471	-0.352108	-0.352108471	3.919505936	3.919505936	3.919505936
644961	8.003293525	7.548899	7.776096252	8.050629261	7.563153223	7.075677186
644962	-0.352108471	-0.352108	-0.352108471	2.432271576	2.024343053	1.616414529
645027	-0.352108471	-0.352108	-0.352108471	2.211943219	1.525243319	2.898643119
645158	2.681724436	5.7155573	-0.352108471	-0.352108471	-0.352108471	-0.352108471
645177	-0.352108471	-0.352108	-0.352108471	0.6663574	2.501526031	4.336694662
645212	-0.352108471	-0.352108	-0.352108471	5.389163245	3.524609154	4.456886199
645367	6.666097446	8.7243324	7.69521493	5.169157826	8.899649703	1.438665948
645432	1.783698732	-0.352108	3.919505936	1.270654317	0.440372779	2.100935855
645644	6.004275218	5.873346	6.135204448	6.649850066	6.689878453	6.609821679
645949	-0.352108471	-0.352108	-0.352108471	5.531832167	6.369156591	7.206481014
645961	2.65059567	1.7607368	3.540454561	-0.352108471	-0.352108471	-0.352108471
646024	6.939871798	5.8780598	8.001683769	-0.352108471	-0.352108471	-0.352108471
646029	-0.352108471	-0.352108	-0.352108471	1.280952158	2.419557138	3.558162118
646074	5.754124716	5.5882244	5.920025058	6.822643333	6.479332994	7.165953671
646113	3.364818568	5.7237042	4.544261391	1.12335747	0.808855576	1.437859363
646127	4.085401266	5.8887701	7.692138989	1.804300246	1.731739118	1.876861374
646214	12.24379962	12.2438	12.24379962	10.9215946	10.68888548	11.15430373
646300	3.794565881	5.8531269	7.91168792	5.911588358	5.639650211	5.367712064
646383	-0.352108471	-0.352108	-0.352108471	6.832688908	4.66005769	2.487426471
646405	5.76907542	6.6633713	7.557667098	3.07793304	8.610332099	5.844132569
646450	6.376119016	6.2451149	6.310616952	7.29372613	6.965995246	7.621457015
646471	7.486480364	7.7121014	7.260859358	6.022419724	6.383151496	5.661687951
646643	5.235408508	5.5587679	5.882127232	-0.352108471	-0.352108471	-0.352108471
646652	5.33908768	6.0381576	5.688622647	-0.352108471	-0.352108471	-0.352108471
646719	4.992965796	5.873346	4.112585606	3.405984988	2.051108653	4.760861323
646762	12.39358467	7.633207	2.872829372	1.086055948	0.440372779	1.731739118
646982	5.51774067	4.8545369	5.186138762	0.727922146	-0.352108471	1.807952763
647024	4.590116187	4.9774847	5.364853187	1.986845002	1.968646897	1.97774595
647060	5.550549618	5.4251658	5.675933456	-0.352108471	-0.352108471	-0.352108471
647087	-0.352108471	-0.352108	-0.352108471	6.767351589	7.177031742	7.586711894
647135	7.862511654	7.0699027	7.466207166	6.000307897	6.681116072	5.319499721
647166	6.964266675	6.0483867	6.506326703	0.689815323	1.731739118	-0.352108471
647215	2.212057023	2.4158264	2.313941724	-0.352108471	-0.352108471	-0.352108471
647979	6.777429272	5.0894122	3.401395132	1.500083195	0.808855576	2.191310813
648691	6.926801914	6.9512116	6.902392266	3.884413197	3.337746555	4.43107984
648791	5.31909676	4.4103073	6.227886211	-0.352108471	-0.352108471	-0.352108471
648987	7.412226129	7.2037262	6.995226235	5.925380896	6.135204448	5.715557343
649159	3.477856611	4.4126518	3.945254192	-0.352108471	-0.352108471	-0.352108471

649305	-0.352108471	-0.352108	-0.352108471	6.314890366	6.179632169	6.450148562
649395	5.273919496	6.979598	6.12675876	7.013764918	5.018248953	6.016006936
650623	3.41407487	3.5426396	3.285510135	5.760537073	9.282132741	2.238941405
651746	5.635939335	6.0375467	5.234331935	-0.352108471	-0.352108471	-0.352108471
652968	-0.352108471	-0.352108	-0.352108471	5.958359385	7.409377372	4.507341397
653121	9.22127001	7.1286201	11.31391991	8.2620304	8.39453474	8.12952606
653190	6.183964016	6.1999612	6.167966808	-0.352108471	-0.352108471	-0.352108471
653238	8.603799159	7.8453125	9.362285792	6.260835661	8.992467768	3.529203555
653269	-0.352108471	-0.352108	-0.352108471	1.307197977	2.139844176	2.972490374
653316	6.300726405	6.6371108	6.973495121	2.202894548	1.916337486	1.629780425
653319	5.805829598	5.313741	6.297918221	6.265347842	5.437723644	7.092972041
653427	5.523239719	5.5882244	5.458255064	6.470258851	6.479332994	6.461184708
653483	6.201178186	6.0367194	6.365637004	1.05704313	1.305230684	0.808855576
653509	-0.352108471	-0.352108	-0.352108471	2.609208936	3.52433951	4.439470085
653567	8.120300038	7.9560775	8.28452261	7.559509372	6.997105418	8.121913326
653641	6.840874497	5.6249562	4.409037828	0.395051519	1.14221151	-0.352108471
653784	1.600140312	3.3630101	2.481575183	7.8702319	5.268086279	6.56915909
654364	-0.352108471	-0.352108	-0.352108471	2.076022254	2.100935855	2.051108653
654434	7.368582523	7.168058	7.569107051	7.078623791	6.674249904	7.482997679
654463	-0.352108471	-0.352108	-0.352108471	6.183964016	8.197762641	4.17016539
654466	5.287558971	7.7872085	6.537383725	0.044132154	-0.352108471	0.440372779
654483	6.958557799	6.321481	6.640019393	3.098246231	2.677051624	3.519440837
654816	-0.352108471	-0.352108	-0.352108471	2.38621749	1.90220876	2.87022622
654841	1.880238567	2.4173215	1.343155683	1.167362396	2.686833264	-0.352108471
664618	8.620055978	6.713186	4.806315972	8.366063136	5.469796697	2.573530259
677763	7.621307356	3.9464575	5.783882438	-0.352108471	-0.352108471	-0.352108471
678655	1.113517241	3.9926294	2.553073296	-0.352108471	-0.352108471	-0.352108471
692094	-0.352108471	-0.352108	-0.352108471	3.73714403	3.919505936	3.554782125
727699	6.59435665	4.9841517	3.373946761	-0.352108471	-0.352108471	-0.352108471
727758	8.301630122	9.4556269	10.60962369	9.10261772	7.895923822	8.499270771
727764	6.352053864	6.3831515	6.320956231	5.284336357	5.421579811	5.147092904
727832	7.821892757	6.1797049	4.537516985	4.253536939	3.519327359	2.785117779
727857	2.481575183	5.3152588	-0.352108471	-0.352108471	-0.352108471	-0.352108471
727896	8.629037055	7.5043071	8.066672076	6.063071306	6.667874621	5.458267991
727897	5.292263812	4.6497476	4.007231426	-0.352108471	-0.352108471	-0.352108471
727910	9.665624708	8.7889793	10.54227015	4.453124139	11.73600339	8.094563763
727956	-0.352108471	-0.352108	-0.352108471	2.588601447	3.416494304	1.760708591
727957	4.80036144	5.5722584	5.186309932	0.044132154	-0.352108471	0.440372779
727982	6.413598714	5.2425006	4.071402517	2.024343053	2.051108653	1.997577452
728099	4.608240284	4.7313422	4.854444195	5.483681163	8.041028188	6.762354676
728113	-0.352108471	-0.352108	-0.352108471	6.220238598	6.842694841	5.597782356
728175	7.827096397	5.3208416	6.573968999	-0.352108471	-0.352108471	-0.352108471
728194	5.014378707	5.0198942	5.017136436	-0.352108471	-0.352108471	-0.352108471
728215	-0.352108471	-0.352108	-0.352108471	5.412561087	6.455024394	4.370097781
728239	-0.352108471	-0.352108	-0.352108471	5.095423988	6.281223384	3.909624592
728276	5.829558501	5.980334	6.131109556	-0.352108471	-0.352108471	-0.352108471
728294	7.991120916	7.0194876	6.04785437	7.903233567	5.303341441	6.603287504

728342	6.037384919	9.6500558	7.843720343	1.036211897	1.14221151	0.930212283
728378	5.12275185	7.819057	2.426446723	2.168105043	4.688318557	-0.352108471
728392	-0.352108471	-0.352108	-0.352108471	1.601336827	-0.352108471	3.554782125
728430	6.333168826	3.5099261	9.156411591	1.500083195	0.808855576	2.191310813
728448	6.982507911	7.3002834	6.664732455	6.512324019	6.569647067	6.540985543
728489	3.127737875	4.7239442	1.531531543	6.235921161	6.275631672	6.19621065
728495	5.784726556	8.2134179	6.999072227	7.514458236	8.711589433	6.31732704
728498	6.199649067	4.3699781	5.284813592	-0.352108471	-0.352108471	-0.352108471
728558	7.916336852	8.5518265	7.280847223	6.108515041	6.411432414	6.714349787
728606	4.263981799	5.2252056	6.186429306	4.381407216	4.356756032	4.406058401
728609	-0.352108471	-0.352108	-0.352108471	6.858057892	5.420879354	3.983700816
728613	4.867086667	2.3933863	7.340787043	1.345967809	1.549724107	1.14221151
728621	7.821159777	6.7329762	7.277067986	5.493783215	5.529311366	5.458255064
728658	6.399093246	7.9086489	7.153871086	5.447926497	7.697805394	9.947684291
728661	5.685832011	4.2674298	2.849027643	0.044132154	0.440372779	-0.352108471
728730	6.155367366	5.626442	5.097516645	2.176777034	2.100935855	2.252618212
728739	3.694803897	1.6766603	2.685732087	-0.352108471	-0.352108471	-0.352108471
728747	-0.352108471	-0.352108	-0.352108471	2.451290702	5.254689875	-0.352108471
728752	5.148678732	6.7431483	8.337617916	7.018254481	6.077045201	7.959463762
728755	2.62245663	2.6703485	2.574564776	0.624614178	0.440372779	0.808855576
728758	3.397643806	2.5975271	4.197760525	0.617107247	3.188422968	1.902765107
728773	8.537285854	6.4307504	7.484018122	7.805112732	5.915575618	6.860344175
728780	5.866795484	6.6189382	5.114652817	-0.352108471	-0.352108471	-0.352108471
728841	4.345552728	5.8769528	7.408352961	-0.352108471	-0.352108471	-0.352108471
728855	-0.352108471	-0.352108	-0.352108471	4.98230645	3.428426646	1.874546843
728882	3.693385802	3.3368967	3.515141236	4.780236561	7.871864475	1.688608647
728927	3.9184181	3.5356078	4.301228371	3.340803748	6.378330955	9.415858162
728929	-0.352108471	-0.352108	-0.352108471	3.185088003	3.180115815	3.182601909
728963	6.07928876	5.5081954	4.937101979	2.681367444	1.807952763	3.554782125
729020	-0.352108471	-0.352108	-0.352108471	5.637296679	4.915312754	6.359280604
729041	-0.352108471	-0.352108	-0.352108471	-0.691758944	5.100511796	2.204376426
729047	2.62245663	-0.352108	5.597021732	-0.352108471	-0.352108471	-0.352108471
729059	-0.352108471	-0.352108	-0.352108471	5.529311366	9.305857378	1.752765354
729082	5.276999284	4.8304259	5.723572646	6.66228969	6.595493818	6.729085562
729083	1.383520155	-0.352108	3.119148782	7.516141656	5.189616664	2.863091671
729156	7.063413806	7.8807081	6.246119475	-0.352108471	-0.352108471	-0.352108471
729218	5.604811639	4.5870979	3.569384172	3.19408489	3.254329098	3.314573306
729224	-0.352108471	-0.352108	-0.352108471	6.087644454	8.896283568	3.279005341
729230	-0.352108471	-0.352108	-0.352108471	4.471969047	5.923493182	7.375017318
729234	7.173216815	5.9002924	4.627367946	-0.352108471	-0.352108471	-0.352108471
729288	5.147625378	5.0309475	5.264303258	1.90117116	0.125768811	3.676573508
729348	12.25629413	12.628608	12.4424511	11.56558005	11.59854904	11.53261106
729375	-0.352108471	-0.352108	-0.352108471	4.502294297	4.651904177	4.801514057
729438	7.135705742	8.3950861	7.765395899	5.252762275	8.656334653	6.954548464
729440	2.280956994	4.9140225	-0.352108471	-0.352108471	-0.352108471	-0.352108471
729522	4.152855543	4.9470072	5.741158835	-0.352108471	-0.352108471	-0.352108471
729582	5.837315695	5.6614885	5.749402114	4.941761661	5.628187051	5.284974356

729603	7.305585019	8.9227724	10.53995979	7.35827986	5.723484561	8.993075158
729614	2.608409114	4.4343147	6.260220223	1.77365078	1.997577452	1.549724107
729627	5.546367231	6.0384535	5.054280952	1.748270748	2.191310813	1.305230684
729648	-0.352108471	-0.352108	-0.352108471	6.204521859	5.08184628	7.327197437
729652	5.924443035	5.4766133	6.372272754	1.622906063	1.807952763	1.437859363
729668	4.736897423	4.3359073	3.934917266	0.044132154	-0.352108471	0.440372779
729683	4.904231184	8.0885771	1.719885262	6.689738252	5.673206489	4.656674726
729723	6.834544042	7.8295873	8.824630522	6.328565253	5.527444067	5.92800466
729732	8.196253796	6.4606394	7.328446606	7.631560883	8.237096988	7.026024778
729815	2.90532861	2.5232121	2.141095603	-0.352108471	-0.352108471	-0.352108471
729830	5.828334333	5.8529375	5.87754059	6.226065905	5.393523004	4.560980104
729920	7.16679269	6.9981067	7.335478698	3.657927132	5.124543452	2.191310813
729950	-0.352108471	-0.352108	-0.352108471	3.039219967	5.052267685	7.065315404
729968	5.995073095	5.006002	4.01693083	0.228373553	-0.352108471	0.808855576
729975	7.785138791	7.9017577	7.668519914	6.782044062	8.442593877	5.121494247
729987	4.933009816	5.5621596	6.191309417	1.383520155	-0.412176871	3.179217182
729991	-0.352108471	-0.352108	-0.352108471	6.306122915	6.988397092	5.623848739
729993	4.281398903	3.8774325	4.079415695	1.835742592	1.731739118	1.939746065
730051	8.347114316	5.7878371	10.90639153	6.308332738	5.245307588	7.371357888
730101	-0.352108471	-0.352108	-0.352108471	1.179995407	2.686833264	4.19367112
730102	6.831289207	6.1259322	7.536646239	0.044132154	0.440372779	-0.352108471
730152	-0.352108471	-0.352108	-0.352108471	5.624186603	5.896887543	5.760537073
730227	4.819198953	4.9110773	4.865138141	1.371545024	1.067877447	1.6752126
730249	5.852937462	5.873346	5.832528937	-0.352108471	-0.352108471	-0.352108471
730338	4.947007189	5.0447512	4.849263165	3.420977679	2.986363532	3.203670606
731424	6.104798308	6.1079622	6.101634422	5.445736258	5.825167992	5.066304524
731631	2.760618758	5.873346	-0.352108471	2.141842139	2.232575625	2.051108653
731789	2.681724436	5.7155573	-0.352108471	-0.352108471	-0.352108471	-0.352108471
752014	6.020381091	6.0384535	6.002308672	-0.352108471	-0.352108471	-0.352108471
767846	7.217317616	7.2305473	7.204087928	-0.352108471	-0.352108471	-0.352108471
768096	7.378986177	7.0205456	6.662104972	8.828542622	6.601861868	4.375181115
768097	5.152247113	5.1799508	5.124543452	-0.352108471	-0.352108471	-0.352108471
780776	1.66985715	-0.352108	3.69182277	1.478197356	0.808855576	2.147539136
780851	4.51958692	5.7397228	3.299451026	-0.352108471	-0.352108471	-0.352108471
790952	9.822866008	10.797959	11.77305288	10.87546361	7.605173409	9.240318508
790955	3.913265813	4.8586077	4.385936778	1.154163373	9.054745048	5.104454211
100009667	4.862123794	6.3881977	5.625160771	-0.352108471	-0.352108471	-0.352108471
100009676	2.313941724	2.4116557	2.216227741	5.776014922	6.996106807	6.386060865
100049587	5.460736426	3.5386593	4.49969785	0.044132154	-0.352108471	0.440372779
100093630	-0.352108471	-0.352108	-0.352108471	6.442417784	4.885056859	5.663737322
100101267	-0.352108471	-0.352108	-0.352108471	9.99566555	6.434644758	2.873623966
100101467	-0.352108471	-0.352108	-0.352108471	7.162930734	6.345275458	5.527620181
100113386	6.76370968	10.481546	8.622627603	6.231464489	7.139414226	6.685439357
100113407	6.551924034	4.5468043	8.557043757	4.524559503	6.318354747	5.421457125
100126784	-0.352108471	-0.352108	-0.352108471	3.257984007	6.597374422	4.927679215
100127888	7.193793348	4.5120816	5.852937462	-0.352108471	-0.352108471	-0.352108471
100127955	6.042866175	5.0522677	4.061669196	-0.352108471	-0.352108471	-0.352108471

100127967	7.010746409	5.3849191	3.759091886	1.76984594	2.022386357	1.517305524
100128006	7.734690198	7.7746004	7.694779984	3.523498977	4.109806718	2.937191237
100128025	4.470233423	3.8926749	4.181454156	0.689815323	1.731739118	-0.352108471
100128071	-0.352108471	-0.352108	-0.352108471	4.559699198	7.402047393	1.717351003
100128126	6.114621473	9.7121152	2.517127752	5.562684146	5.69655082	5.830417494
100128127	-0.352108471	-0.352108	-0.352108471	3.523498977	4.702326924	2.34467103
100128233	6.915905977	6.0042752	5.092644458	3.622795883	5.054280952	2.191310813
100128252	2.84317252	6.0384535	-0.352108471	-0.352108471	-0.352108471	-0.352108471
100128285	7.480076024	6.991795	6.503514071	-0.352108471	-0.352108471	-0.352108471
100128288	6.814666936	8.0506494	5.578684505	5.114526679	6.720022075	8.32551747
100128292	5.184151491	2.5588929	7.809410083	1.190059422	1.939746065	0.440372779
100128338	5.880618662	6.7193663	7.558114024	5.075545493	3.96138536	2.847225226
100128340	6.861057494	7.4239083	6.298206641	-0.352108471	-0.352108471	-0.352108471
100128398	7.126229097	7.069396	7.09781253	7.015842684	6.697558522	6.856700603
100128494	6.593987376	6.6737661	6.633876737	8.580655142	4.058374142	6.319514642
100128531	6.224862874	6.9423464	5.507379381	5.875147482	5.702486887	6.047808077
100128640	2.976771008	5.6290742	4.302922615	1.769397609	2.186754596	1.352040622
100128682	3.29075662	4.5572447	5.823732761	3.725242432	3.669380942	3.613519451
100128731	10.31959442	6.2849209	8.302257681	8.332318834	7.695296622	8.013807728
100128788	4.587097906	3.1543068	6.019888963	2.825622527	4.13460375	1.516641303
100128881	-0.352108471	-0.352108	-0.352108471	4.020564006	3.119148782	2.217733557
100128946	-0.352108471	-0.352108	-0.352108471	5.845486367	5.107185874	5.47633612
100128966	5.90029238	6.7755365	5.025048275	-0.352108471	-0.352108471	-0.352108471
100129027	-0.352108471	-0.352108	-0.352108471	2.19005738	2.232575625	2.147539136
100129138	5.483227165	5.0272459	5.939208422	1.77365078	1.997577452	1.549724107
100129195	3.55296241	3.4856923	3.519327359	5.441760481	4.907571138	4.373381794
100129223	-0.352108471	-0.352108	-0.352108471	5.397423705	5.596916991	5.796410278
100129269	7.324111644	8.9656576	8.144884631	5.320380917	7.30293721	6.311659063
100129361	-0.352108471	-0.352108	-0.352108471	6.532441983	9.065224186	3.999659781
100129387	4.152721357	4.7816768	3.523765884	0.215589614	2.101644539	1.158617076
100129396	-0.352108471	-0.352108	-0.352108471	5.48038507	5.605609608	5.355160532
100129434	13.71126191	12.287112	12.99918694	12.09160162	12.05978117	12.12342206
100129461	3.897916785	5.3211461	6.744375431	-0.352108471	-0.352108471	-0.352108471
100129482	3.434063996	5.5868821	7.739700271	3.518475388	6.056053999	8.59363261
100129503	4.058159444	3.136327	4.979991919	1.245740716	0.440372779	2.051108653
100129520	6.939871798	6.9529417	6.926801914	-0.352108471	-0.352108471	-0.352108471
100129583	2.481575183	2.4530973	2.510053061	-0.352108471	-0.352108471	-0.352108471
100129620	5.841308752	5.5587679	5.276226989	0.044132154	-0.352108471	0.440372779
100129662	6.991795048	6.9981067	6.985483415	6.345275458	6.355161685	6.33538923
100129697	-0.352108471	-0.352108	-0.352108471	0.919601171	-0.352108471	2.191310813
100129716	7.844935289	8.1767271	8.010831185	4.560250079	7.120543035	9.680835992
100129935	5.459607292	7.398238	6.428922631	0.791292145	1.14221151	0.440372779
100130075	-0.352108471	-0.352108	-0.352108471	5.587357467	5.734165027	5.440549907
100130093	-0.352108471	-0.352108	-0.352108471	3.384482817	4.624785411	2.144180224
100130207	-0.352108471	-0.352108	-0.352108471	3.585121301	5.13628526	2.033957342
100130231	4.871233248	4.617923	5.124543452	-0.352108471	-0.352108471	-0.352108471
100130238	-0.352108471	-0.352108	-0.352108471	2.738224417	1.600625532	2.169424975

100130285	6.263611604	5.1235654	7.40365782	1.731739118	1.731739118	1.731739118
100130451	10.05731557	9.5447948	9.80105521	7.386283023	8.96894828	5.803617767
100130520	7.460800975	3.902655	5.681727979	0.874413692	2.100935855	-0.352108471
100130557	7.146175474	6.1995978	8.092753187	3.892888144	6.03613661	8.179385076
100130581	8.750782632	7.7714348	6.792086899	3.251947075	4.283924671	5.315902268
100130698	4.393370554	0.712776	2.553073296	-0.352108471	-0.352108471	-0.352108471
100130776	-0.352108471	-0.352108	-0.352108471	6.554628999	6.590717732	6.572673365
100130872	5.190074303	5.054281	5.325867653	1.640731613	1.549724107	1.731739118
100130890	7.107828715	6.3623856	7.853271846	6.132257081	5.32075504	4.509253
100130899	5.644336636	8.320514	2.968159301	1.327000025	1.72638491	2.125769796
100130954	6.536804188	6.4186985	6.65490985	5.928916513	6.74495349	7.560990466
100131017	8.889567996	6.0628006	7.476184293	1.371545024	1.437859363	1.305230684
100131067	5.948609857	6.3414989	6.734388006	6.55718572	5.56182082	7.55255062
100131089	6.726175503	6.8469245	6.967673497	1.117721484	1.305230684	0.930212283
100131096	5.277347693	4.7935692	5.035458467	2.878545696	2.507535085	2.693040391
100131107	-0.352108471	-0.352108	-0.352108471	4.662527092	5.859617369	7.056707646
100131205	5.347774631	5.7155573	4.979991919	0.542875446	-0.352108471	1.437859363
100131211	-0.352108471	-0.352108	-0.352108471	5.869665514	5.893442277	5.845888751
100131257	12.68803241	14.378806	10.9972591	13.50973311	11.56558005	9.621426987
100131262	1.66985715	-0.352108	3.69182277	3.420123827	4.456886199	5.493648572
100131315	-0.352108471	-0.352108	-0.352108471	5.080554094	5.529311366	4.631796822
100131320	-0.352108471	-0.352108	-0.352108471	5.095423988	5.124543452	5.066304524
100131347	3.75254278	4.3859368	3.119148782	1.74784442	2.686833264	0.808855576
100131496	6.535909516	6.7454347	6.326384314	3.668632064	6.014452276	1.322811852
100131564	8.43039751	7.4288242	7.92961084	6.995794281	7.152887698	7.309981114
100131691	5.149020242	6.3964328	3.901607702	3.533307748	0.534864438	2.034086093
100131801	-0.352108471	-0.352108	-0.352108471	6.502035182	5.048101249	5.775068216
100131897	-0.352108471	-0.352108	-0.352108471	2.191584826	2.313651598	2.252618212
100132057	6.573968999	6.9723174	6.175620628	-0.352108471	-0.352108471	-0.352108471
100132077	5.256484155	6.9128905	8.569296897	5.504186002	5.315258837	5.693113166
100132101	4.513304033	7.2765395	1.750068586	3.857445023	4.578801146	5.300157269
100132154	8.881348136	7.9746271	8.427987642	7.624230206	7.505494249	7.386758292
100132169	4.245214285	1.0745472	7.415881363	5.105431891	5.504902093	5.904372294
100132249	8.321094611	8.0744353	8.567753944	5.506611151	6.903819455	8.301027759
100132295	5.749402114	5.7752316	5.723572646	-0.352108471	-0.352108471	-0.352108471
100132354	7.909679438	5.9611669	4.012654293	-0.352108471	-0.352108471	-0.352108471
100132526	5.860022834	5.8615274	5.858518226	6.051012296	4.209125924	7.892898669
100132594	4.803618005	2.735462	6.871774048	-0.352108471	-0.352108471	-0.352108471
100132619	3.862940367	2.3925354	3.127737875	4.041902295	4.80208491	5.562267525
100132677	7.208142848	6.1333361	5.058529434	1.12335747	0.808855576	1.437859363
100132774	5.712678347	6.4453648	4.979991919	3.90093566	5.804293869	1.997577452
100132815	6.520799499	6.1414231	6.900175883	4.456886199	4.482692559	4.43107984
100132832	-0.352108471	-0.352108	-0.352108471	2.121209733	2.191310813	2.051108653
100132874	-0.352108471	-0.352108	-0.352108471	3.591645958	0.650773508	2.121209733
100132911	6.331936898	7.0611019	5.602771915	6.540477509	6.353906971	6.167336433
100133036	-0.352108471	-0.352108	-0.352108471	0.95025487	-0.352108471	2.252618212
100133121	5.195685681	5.5202832	5.357984451	5.178880455	4.48140857	5.87635234

100133331	3.931977972	4.6173824	5.3027868	-0.352108471	-0.352108471	-0.352108471
100133920	6.97258325	4.2184161	5.595499658	-0.352108471	-0.352108471	-0.352108471
100133941	7.037781708	8.1576667	5.917896697	6.755846282	6.813225323	6.698467242
100134040	-0.352108471	-0.352108	-0.352108471	1.581393954	2.232575625	0.930212283
100134444	-0.352108471	-0.352108	-0.352108471	2.168105043	4.688318557	-0.352108471
100134868	5.107302037	5.8692224	6.63114281	0.228373553	0.808855576	-0.352108471
100142659	5.354685649	5.3835036	5.325867653	-0.352108471	-0.352108471	-0.352108471
100144595	6.697650533	6.0584885	6.378069496	8.175722907	5.939256449	3.702789991
100144748	6.930096189	6.9037827	6.956409727	6.77688455	6.750862801	6.802906299
100147773	5.656289537	5.7155573	5.597021732	0.228373553	-0.352108471	0.808855576
100188917	-0.352108471	-0.352108	-0.352108471	4.80208491	5.374947902	4.229221918
100188947	6.136795257	6.3152311	5.958359385	5.23177507	4.688318557	5.775231583
100188953	5.91063331	5.6532998	6.167966808	1.437859363	1.437859363	1.437859363
100188954	6.475834528	7.0002884	5.951380647	1.223721097	0.861671934	1.58577026
100190938	6.236695219	6.0524358	6.420954684	5.655380401	7.482915129	6.569147765
100190940	3.183399768	6.8286042	5.006001962	6.251703356	4.943908594	3.636113833
100190986	6.551531362	8.2669293	4.836133438	6.518776878	7.302381905	8.085986931
100191040	6.28106638	6.1995956	6.118124884	-0.352108471	-0.352108471	-0.352108471
100192420	10.10926989	6.0299969	1.95072392	0.791292145	0.440372779	1.14221151
100216479	4.984393465	4.2452143	5.723572646	1.727216326	1.646479888	1.807952763
100233209	4.795188378	5.0805541	5.36591981	1.043426334	1.646479888	0.440372779
100268168	8.107324794	6.7648161	5.422307458	5.509057391	5.482709348	5.495883369
100271715	5.284974356	5.3152588	5.254689875	-0.352108471	-0.352108471	-0.352108471
100271836	3.591652	2.0145989	2.803125457	-0.352108471	-0.352108471	-0.352108471
100272147	-0.352108471	-0.352108	-0.352108471	3.850724089	5.958359385	8.06599468
100286844	4.758263834	4.7359333	4.780594366	1.223721097	1.305230684	1.14221151
100287015	6.606671778	7.8867459	5.326597619	2.350520741	1.761670631	1.172820521
100287036	-0.352108471	-0.352108	-0.352108471	8.042215924	6.311659063	4.581102202
100287042	4.363953422	3.3234906	5.404416235	7.43611685	6.272179388	8.600054313
100287082	-0.352108471	-0.352108	-0.352108471	2.911033875	5.596916991	8.282800107
100287226	-0.352108471	-0.352108	-0.352108471	1.383520155	3.119148782	-0.352108471
100287270	5.546108263	4.8304259	6.261790604	5.284974356	5.315258837	5.254689875
100287290	6.504249638	6.5970178	6.550633696	6.445401299	5.847710919	5.25002054
100287314	6.605482243	6.2479641	5.890445911	4.481413792	5.868531014	7.255648236
100287387	5.217320325	6.1476709	4.286969732	1.405996421	3.576708353	5.747420285
100287616	2.245600013	3.3053943	1.185805728	6.204656258	7.449796053	4.959516463
100287639	2.631634217	1.4021941	2.016914154	3.880309919	2.439072038	0.997834158
100287722	8.81650169	7.0871828	5.35786397	4.772383248	6.106446694	7.44051014
100287792	8.339229013	6.5710539	4.802878743	5.431542975	6.297453798	7.16336462
100287823	3.86984729	6.9853317	5.42758951	-0.352108471	-0.352108471	-0.352108471
100287846	4.908926167	6.1368079	7.364689543	4.09062522	2.022080686	6.159169754
100287898	-0.352108471	-0.352108	-0.352108471	4.927679215	4.954218546	4.901139884
100287932	2.15990226	1.5108201	2.808984446	2.53101606	3.919505936	5.307995811
100288077	4.616975419	1.8734952	3.245235305	5.821410838	4.025622598	7.617199078
100288079	3.726804584	1.7944329	2.760618758	-0.352108471	-0.352108471	-0.352108471
100288142	5.852937462	4.5710473	7.134827656	-0.352108471	-0.352108471	-0.352108471
100288181	5.50819537	5.9991789	5.017211832	2.035970564	4.732995071	3.384482817

100288254	6.536514975	7.0300312	6.042998786	6.804723141	4.97854774	8.630898543
100288527	6.121528769	5.178749	7.064308531	-0.352108471	-0.352108471	-0.352108471
100288615	6.427319214	5.4483354	5.937827329	-0.352108471	-0.352108471	-0.352108471
100288748	4.115678136	6.231676	5.173677083	1.14221151	1.14221151	1.14221151
100288778	1.065336338	2.3139417	3.56254711	-0.352108471	-0.352108471	-0.352108471
100288846	5.688622647	5.7155573	5.661687951	-0.352108471	-0.352108471	-0.352108471
100289019	6.709235363	6.8088907	6.908546041	7.712812022	6.091591095	4.470370168
100289097	3.792256394	6.3604577	5.07635705	1.223721097	1.305230684	1.14221151
100289124	4.596401634	5.289714	3.903089269	-0.049510209	4.241874486	2.096182139
100289137	6.223836723	6.4453648	6.002308672	-0.352108471	-0.352108471	-0.352108471
100289341	6.957178318	8.0271653	5.887191311	6.058226619	1.488244727	3.773235673
100289580	6.268384805	6.8018138	7.335242742	-0.352108471	-0.352108471	-0.352108471
100289650	-0.352108471	-0.352108	-0.352108471	2.43016825	-0.352108471	5.212444972
100289678	4.890484718	5.9092789	5.399881815	1.70048971	1.556591723	1.412693737
100292680	4.640942554	4.1925858	4.416764197	1.454895716	0.808855576	2.100935855
100294362	4.381407216	3.9195059	4.843308497	2.929626001	1.939746065	3.919505936
100302401	5.401515054	5.6310304	5.516272736	7.361599114	7.480110924	7.243087303
100302692	4.947007189	5.5168937	4.377120644	3.820309957	4.665843474	2.97477644
100302739	3.663903369	1.8645479	2.764225616	-0.352108471	-0.352108471	-0.352108471
100302740	2.201036472	3.7787594	0.62331358	-0.352108471	-0.352108471	-0.352108471
100302746	6.305685134	7.197895	5.413475306	8.946835015	5.611212266	2.275589517
100306975	5.370483827	5.6886226	6.006761467	-0.352108471	-0.352108471	-0.352108471
100309464	4.412824513	4.6756983	4.544261391	0.624614178	0.440372779	0.808855576
100313796	7.384321494	4.3215534	5.852937462	4.797006422	3.042005449	3.919505936
100313865	6.276131817	7.1577076	8.039283402	-0.352108471	-0.352108471	-0.352108471
100316904	-0.352108471	-0.352108	-0.352108471	5.983736712	5.436324997	6.531148427
100335030	7.112913835	6.4125415	5.712169066	5.170565066	6.671548634	8.172532203
100379571	3.597748589	4.4336808	5.26961299	5.680349863	3.820978157	1.96160645
100419583	4.552679654	2.5530733	0.553466939	-0.352108471	-0.352108471	-0.352108471
100419743	8.934815581	7.6894359	10.18019526	6.811762296	8.597145682	7.704453989
100420587	8.710776712	5.8275854	7.269181038	9.310773304	4.468703989	6.889738646
100462977	8.973010505	9.3530434	9.163026968	9.022833616	8.645133758	8.833983687
100462981	10.80677072	13.057697	8.555844046	10.97038763	10.46808925	9.965790881
100462983	3.07615903	7.054009	5.065084027	3.597628112	3.762355996	3.679992054
100463289	6.037807317	5.4397245	4.841641627	4.76431248	3.07604078	1.38776908
100463482	8.166673639	8.535703	8.904732444	7.146480757	7.723482864	6.569478649
100463486	11.00559925	10.606781	10.80619016	9.507655551	8.710935774	10.30437533
100463487	7.964028563	9.3643842	8.664206359	7.120490641	9.772620848	8.446555745
100463488	5.830939431	5.5882244	6.073654487	2.221964512	2.191310813	2.252618212
100499466	5.980186278	5.4364282	5.708307224	5.525865331	4.930663214	6.121067448
100499483	5.463922025	6.7666215	4.161222578	4.512821737	-0.176611651	2.168105043
100505477	4.169848031	5.9953913	5.082619672	-0.352108471	-0.352108471	-0.352108471
100505478	-0.352108471	-0.352108	-0.352108471	2.38621749	6.167503983	-1.395069002
100505495	4.424692132	5.549469	4.987080576	3.923203137	3.99316708	3.853239194
100505532	-0.352108471	-0.352108	-0.352108471	7.472116188	3.814964051	5.64354012
100505534	4.819685928	5.5438096	5.181747782	-0.352108471	-0.352108471	-0.352108471
100505547	6.273832259	6.5780759	5.969588655	-0.352108471	-0.352108471	-0.352108471

100505566	4.013028315	7.2157186	0.810338079	4.407187888	2.627398558	0.847609227
100505573	-0.352108471	-0.352108	-0.352108471	3.99074184	6.347573811	5.169157826
100505576	7.60286746	7.2013529	8.00438199	3.098679711	5.522284255	7.945888799
100505583	6.056053999	6.7490886	5.363019386	-0.352108471	-0.352108471	-0.352108471
100505609	6.009680338	5.6251608	5.240641204	-0.352108471	-0.352108471	-0.352108471
100505621	8.964272546	9.380969	8.547576081	8.551289593	8.474204208	8.628374978
100505633	6.551825805	6.3152311	6.788420481	4.655759405	4.245214285	5.066304524
100505641	4.178899946	4.2452143	4.112585606	1.218975116	0.440372779	1.997577452
100505650	7.675801096	7.5842785	7.767323737	7.061166384	6.672196951	7.450135817
100505659	5.468819781	5.3910182	5.313216548	3.923814016	5.95428886	7.984763704
100505664	-0.352108471	-0.352108	-0.352108471	4.233855154	7.73361827	5.983736712
100505676	7.92672776	5.3767238	6.651725767	1.997577452	-1.794744538	5.789899442
100505727	-0.352108471	-0.352108	-0.352108471	2.204376426	1.273088389	3.135664463
100505728	5.876276997	6.1945795	5.557974474	-0.352108471	-0.352108471	-0.352108471
100505741	-0.352108471	-0.352108	-0.352108471	3.981228639	4.843308497	3.119148782
100505746	-0.352108471	-0.352108	-0.352108471	2.469725738	2.836168613	2.103282862
100505768	-0.352108471	-0.352108	-0.352108471	6.165870606	6.468860513	5.862880699
100505776	4.214046665	6.6583352	5.436190922	0.854785337	6.55475994	3.704772639
100505795	-0.352108471	-0.352108	-0.352108471	6.501802117	3.618783359	5.060292738
100505806	6.242048279	6.2558778	6.228218771	6.890254017	6.907463027	6.873045007
100505811	5.599077984	3.874921	7.323234927	7.655347763	5.47633612	3.297324477
100505812	4.433680789	3.6854445	5.181917044	1.290035436	1.14221151	1.437859363
100505817	6.170281192	4.0890016	5.129641382	-0.352108471	-0.352108471	-0.352108471
100505876	7.585390785	7.5821735	7.588608089	6.683949098	6.617035395	6.750862801
100505881	6.413173056	7.4427409	5.383605256	4.326502696	4.736526515	3.916478878
100505915	7.050880463	7.0683905	7.033370399	-0.352108471	-0.352108471	-0.352108471
100505989	5.704193438	7.1675494	8.630905282	6.00998441	6.371856746	5.648112075
100505994	1.383520155	-0.352108	3.119148782	1.454895716	2.100935855	0.808855576
100506007	7.624244847	7.4792768	7.334308775	-0.352108471	-0.352108471	-0.352108471
100506012	3.733128669	5.1529298	6.572730904	-0.352108471	-0.352108471	-0.352108471
100506013	7.30256871	6.2420483	5.181527848	-0.352108471	-0.352108471	-0.352108471
100506022	4.317298305	4.803618	5.289937705	4.065642901	3.064586224	2.063529546
100506023	6.073694796	6.5712473	6.32247105	5.964157879	6.004373062	5.984265471
100506033	6.354439508	5.5985342	7.110344824	2.02034096	2.231655729	1.809026191
100506060	7.241080306	8.1332833	9.025486343	6.877892015	7.195390655	7.036641335
100506083	4.851734675	3.9346201	5.76884929	3.880482573	7.178501171	5.529491872
100506085	6.720916484	6.7174473	6.724385635	0.440372779	0.440372779	0.440372779
100506119	6.65994771	4.9704738	3.280999913	-0.352108471	-0.352108471	-0.352108471
100506123	6.061443125	4.4306707	5.246056936	-0.352108471	-0.352108471	-0.352108471
100506124	-0.352108471	-0.352108	-0.352108471	5.529311366	5.529311366	5.529311366
100506127	8.162935329	9.4415973	6.884273359	6.677377896	5.438642631	6.058010264
100506178	3.41960233	3.1277379	2.835873421	4.670941474	3.402425854	4.036683664
100506190	6.434445821	9.1538721	3.715019519	2.064739793	2.252618212	1.876861374
100506211	4.21321388	5.7429572	7.272700451	6.752332956	5.17119118	5.961762068
100506215	7.618125877	9.7470534	5.489198398	5.712263427	5.731413688	5.693113166
100506217	-0.352108471	-0.352108	-0.352108471	6.28946978	4.964541288	5.627005534
100506248	-0.352108471	-0.352108	-0.352108471	3.081409479	1.731739118	4.43107984

100506305	5.021893812	8.4738855	6.747889644	3.47770936	5.354454372	4.416081866
100506334	10.18208261	9.7845569	9.983319737	7.896677475	9.208129949	10.51958242
100506376	7.735551336	5.500886	6.618218658	1.374300821	0.808855576	1.939746065
100506385	6.840029989	5.5425257	8.137534304	5.54524521	5.624920123	5.465570297
100506392	5.919459543	5.0916698	6.747249254	-0.352108471	-0.352108471	-0.352108471
100506394	7.846085362	7.428576	7.637330686	5.698930132	5.665251835	5.631573539
100506444	4.149549778	6.0481973	2.250902235	3.222881898	4.312084516	3.767483207
100506469	3.87583822	4.0310378	4.18623739	2.738485171	1.509536442	0.280587712
100506472	4.878665478	2.3521284	7.405202581	5.573019814	6.670158794	6.121589304
100506476	6.731869951	3.0765924	4.904231184	1.060446687	1.727216326	2.393985965
100506492	0.775109013	1.5010176	2.226926156	1.337894877	2.212189733	3.086484589
100506495	5.357984451	5.283117	5.432851874	0.228373553	0.808855576	-0.352108471
100506504	9.121866293	4.7051115	6.913488898	-0.352108471	-0.352108471	-0.352108471
100506532	6.433318728	5.2420678	7.624569647	-0.352108471	-0.352108471	-0.352108471
100506548	4.162220877	6.1404366	5.151328716	7.637458303	4.880328202	6.258893253
100506551	7.692413376	7.7313381	7.653488636	7.035244821	7.3720224	6.698467242
100506555	6.074913188	6.8524366	6.463674915	-0.352108471	-0.352108471	-0.352108471
100506596	4.117696434	1.9715532	6.263839711	2.618057952	5.588224375	-0.352108471
100506599	4.736638248	4.9140225	4.559254036	3.196376711	4.245214285	2.147539136
100506603	6.141253736	6.0734373	6.005620908	3.592345606	5.842426786	8.092507965
100506621	6.689815331	6.1039214	6.396868383	-0.352108471	-0.352108471	-0.352108471
100506627	5.485331377	6.2752044	5.880267911	0.721578631	1.14221151	1.562844388
100506634	8.879682209	9.8042101	10.72873809	9.083144505	9.045726342	9.008308179
100506637	9.417455405	7.4264413	8.421948362	9.217664463	5.390634204	7.304149333
100506658	6.12526732	8.1806123	7.152939819	4.174472381	2.098181557	3.136326969
100506679	2.972455137	4.1818679	5.391280624	2.460720591	3.253150981	1.668290201
100506688	7.295688755	7.4239334	7.167444067	4.450744817	3.337746555	5.563743079
100506697	4.150643996	3.0982686	3.624456288	6.660249617	5.884416135	7.436083099
100506710	-0.352108471	-0.352108	-0.352108471	5.886425029	10.53963486	1.233215197
100506714	4.463716617	6.3034612	8.143205873	5.859617369	6.359615552	5.359619186
100506730	8.712413858	1.3637643	5.038089054	1.05704313	2.352760652	-0.238674391
100506736	6.183964016	7.1243353	5.243592684	-0.352108471	-0.352108471	-0.352108471
100506742	5.493783215	2.9014243	8.086142146	-0.352108471	-0.352108471	-0.352108471
100506746	6.033596974	5.873346	6.193847962	1.814585088	1.437859363	2.191310813
100506749	-0.352108471	-0.352108	-0.352108471	6.416697544	6.087644454	5.758591365
100506757	1.392109249	3.136327	-0.352108471	5.704335255	5.308347618	6.100322892
100506759	4.739268142	3.5151412	2.29101433	5.800199788	6.607682849	4.992716726
100506769	1.891775157	2.6505957	3.409416183	-0.352108471	-0.352108471	-0.352108471
100506783	5.391018164	5.3835036	5.398532684	-0.352108471	-0.352108471	-0.352108471
100506801	-0.352108471	-0.352108	-0.352108471	7.692954228	4.66005769	1.627161151
100506826	-0.352108471	-0.352108	-0.352108471	2.38621749	5.124543452	-0.352108471
100506834	6.664657419	7.0526396	6.85864849	1.657360368	1.876861374	1.437859363
100506835	6.579604628	7.0871828	7.594761031	-0.352108471	-0.352108471	-0.352108471
100506858	4.161208001	6.5373837	8.913559448	-0.352108471	-0.352108471	-0.352108471
100506881	6.081810127	6.2432352	6.40466022	5.10037386	4.567936616	4.834155238
100506901	-0.352108471	-0.352108	-0.352108471	6.270222109	7.376601672	5.163842545
100506930	6.779094686	7.8857032	8.992311811	5.608062679	4.485523326	5.046793002

100506990	2.977745599	6.9162688	4.947007189	0.044132154	-0.352108471	0.440372779
100506994	5.851448667	7.568737	6.710092826	5.925900831	5.473956012	6.37784565
100507003	-0.352108471	-0.352108	-0.352108471	4.66005769	4.688318557	4.631796822
100507032	6.038177363	6.479333	5.597021732	1.697414861	1.14221151	2.252618212
100507043	1.383520155	-0.352108	3.119148782	4.583433279	5.054280952	4.112585606
100507050	-0.352108471	-0.352108	-0.352108471	4.927679215	4.954218546	4.901139884
100507056	3.41407487	3.3694434	3.458706305	4.018998419	6.204656258	8.390314097
100507099	8.083335993	8.484805	8.284070521	5.863122202	6.333759457	6.098440829
100507117	4.750181605	9.1014762	6.925828885	4.110546098	4.73755266	5.364559222
100507131	6.267656397	7.8014657	9.335274935	6.926938623	4.989564878	8.864312367
100507156	4.028491804	4.1460235	4.087257665	2.12839562	2.791013268	2.459704444
100507171	2.032840233	5.1296414	8.226442532	3.94669085	0.499242608	7.394139091
100507173	5.224957289	5.417548	5.321252663	4.535401412	7.083611396	5.809506404
100507175	5.557045014	5.7155573	5.398532684	1.371545024	1.305230684	1.437859363
100507178	7.815126942	4.9808044	10.64944948	6.774540384	6.538191011	7.010889757
100507217	-0.352108471	-0.352108	-0.352108471	4.456886199	4.482692559	4.43107984
100507236	7.218664846	6.6632761	6.107887289	7.25758336	6.316270247	5.374957134
100507246	8.221025114	4.1730525	6.197038815	5.217592704	7.644891787	6.431242245
100507290	6.71500546	6.0404998	6.377752623	7.001809086	5.791927679	6.396868383
100507321	7.276703885	7.1385499	7.00039595	6.650500242	7.249321749	6.051678734
100507336	-0.352108471	-0.352108	-0.352108471	4.846943929	5.915408166	6.983872402
100507373	6.735779686	6.3070145	7.164544881	-0.352108471	-0.352108471	-0.352108471
100507395	6.369156591	6.3935345	6.344778649	-0.352108471	-0.352108471	-0.352108471
100507412	10.22759617	10.583313	9.87187898	11.31710026	9.029935741	6.742771225
100507424	-0.352108471	-0.352108	-0.352108471	5.165434699	4.926927932	5.046181316
100507428	6.970773049	7.2698636	7.568954175	6.262318958	6.629118201	6.445718579
100507431	6.808720513	7.2622793	6.355161685	3.820525905	4.954218546	2.686833264
100507436	2.760618758	5.873346	-0.352108471	1.117721484	1.305230684	0.930212283
100507460	6.364461518	7.533337	8.702212427	4.80208491	4.843308497	4.760861323
100507501	3.059467309	4.4333835	5.807299712	5.523239719	7.25482461	3.791654829
100507507	6.801813774	6.8152071	6.788420481	-0.352108471	-0.352108471	-0.352108471
100507513	5.749402114	5.7752316	5.723572646	-0.352108471	-0.352108471	-0.352108471
100507516	7.305341479	8.1372473	6.473435614	0.872801732	0.440372779	1.305230684
100507524	10.8705125	10.870512	10.8705125	9.632852697	9.535687614	9.73001778
100507533	6.939808856	7.4494081	7.194608474	-0.352108471	-0.352108471	-0.352108471
100507577	5.526269952	6.3324667	7.138663385	2.018703435	5.709342649	3.864023042
100507582	5.357984451	5.3507587	5.365210183	3.687748495	4.182695441	4.677642388
100507584	5.308716024	5.2396395	5.17056307	5.839632047	5.945858472	5.733405623
100507588	4.025829494	6.5441192	5.284974356	-0.352108471	-0.352108471	-0.352108471
100507616	9.308742826	5.2639065	7.286324647	-0.352108471	-0.352108471	-0.352108471
100507629	6.456494764	6.7357797	7.015064609	-0.352108471	-0.352108471	-0.352108471
100507632	4.119761196	6.2420483	8.364335362	-0.352108471	-0.352108471	-0.352108471
100507639	7.544161386	5.3141829	3.084204395	-0.352108471	-0.352108471	-0.352108471
100507642	6.640019393	6.517941	6.762097775	-0.352108471	-0.352108471	-0.352108471
100507651	-0.352108471	-0.352108	-0.352108471	5.223343677	5.095423988	4.967504298
100507679	7.18462267	7.1464808	7.222764584	0.395051519	1.14221151	-0.352108471
100509894	5.493095576	6.916299	8.339502466	4.53499238	3.045613449	6.024371311

100526783	0.500398845	6.5554341	3.527916452	2.359319907	1.388378921	1.873849414
100526830	-0.352108471	-0.352108	-0.352108471	6.152314334	4.842884858	3.533455382
100527964	6.484281017	6.4636749	6.443068813	-0.352108471	-0.352108471	-0.352108471
100528007	6.45963463	5.9258255	6.192730084	4.233779363	2.313289664	0.392799966
100528022	4.182087776	7.7785803	5.980334028	2.481575183	5.315258837	-0.352108471
100528030	5.729853661	4.4868934	3.243933226	1.657360368	1.876861374	1.437859363
100529209	-0.352108471	-0.352108	-0.352108471	7.004646402	4.767082708	2.529519015
100529239	4.692371316	5.3889268	6.085482282	5.951161712	4.866065849	7.036257576
100533105	5.168704884	3.4624462	4.315575532	1.918895351	2.191310813	1.646479888
100533177	-0.352108471	-0.352108	-0.352108471	4.970473812	6.4346232	3.506324423
100533182	7.878030571	6.3072687	4.736506872	5.2185599	5.957888849	5.588224375
100533789	10.34243084	8.3981147	6.453798619	6.574085782	7.514364071	7.044224926
100533970	8.203621941	8.4092908	8.306456348	6.677703761	6.260622078	7.094785444
100534012	6.294300225	6.6959853	6.495142767	5.804562573	7.79988811	6.802225342
100616668	2.379164056	0.6228711	1.501017585	9.922559147	5.909748525	1.896937903
100652739	-0.352108471	-0.352108	-0.352108471	2.313941724	-0.352108471	4.979991919
100652748	6.233801298	4.6590193	3.084237322	4.004921698	6.60492871	5.304925204
100652759	-0.352108471	-0.352108	-0.352108471	0.874413692	2.100935855	-0.352108471
100652772	6.668893685	6.0023087	7.335478698	3.44626434	3.337746555	3.554782125
100652833	5.852937462	5.873346	5.832528937	-0.352108471	-0.352108471	-0.352108471
100652846	-0.352108471	-0.352108	-0.352108471	3.180447167	1.90830372	0.636160272
100652901	10.44157452	10.165379	10.71776974	7.942682924	11.11822571	9.530454315
100652929	7.392944949	6.2231429	8.562747011	7.050965989	6.242392959	5.433819929
100653061	2.201036472	-0.352108	4.754181416	1.179289842	1.549724107	0.808855576
100653233	5.201288977	5.1847699	5.168250812	-0.352108471	-0.352108471	-0.352108471
100750225	5.92524845	5.6613207	5.397393041	3.200243148	5.243942167	7.287641187
100775104	-0.352108471	-0.352108	-0.352108471	5.88700099	5.893442277	5.880559702
100775107	6.408050094	7.6087389	5.207361299	0.872801732	0.440372779	1.305230684
100820709	5.658783754	7.1941471	4.123420449	4.591397191	5.525865331	6.460333472
100846978	14.26064462	13.408044	12.55544429	13.65479255	13.16129636	13.40804445
100852408	7.087105625	2.8069088	4.947007189	-0.352108471	-0.352108471	-0.352108471
100859921	2.764225616	-0.352108	5.880559702	2.634592538	-0.352108471	5.621293547
100861412	5.311892488	4.5821219	4.947007189	0.927192453	1.807952763	2.688713073
100861437	-0.352108471	-0.352108	-0.352108471	4.113585595	5.511864271	6.910142947
100861504	5.217491139	4.5465207	5.888461528	-0.352108471	-0.352108471	-0.352108471
100861532	12.14765807	11.283307	13.01200934	13.0724719	9.667599279	11.37003559
100861542	1.392109249	3.136327	-0.352108471	4.571042117	5.095423988	5.619805859
100861549	6.98777334	8.1525971	7.570185228	6.000135948	6.917282891	6.45870942
100861555	-0.352108471	-0.352108	-0.352108471	6.406486527	6.054765778	6.758207276
100862680	9.469543183	11.302183	7.636902938	8.603890228	8.526024375	8.681756082
100862704	2.681724436	5.7155573	-0.352108471	0.044132154	0.440372779	-0.352108471
100873165	1.924048886	1.0779863	1.501017585	7.905324643	6.235921161	4.566517679
100873964	4.58739024	3.136327	6.038453511	-0.352108471	-0.352108471	-0.352108471
100873974	-0.352108471	-0.352108	-0.352108471	2.301055037	4.954218546	-0.352108471
100873979	6.056053999	6.0736545	6.038453511	-0.352108471	-0.352108471	-0.352108471
100874019	9.497739328	8.785672	8.073604611	6.798638809	7.812123768	8.825608726
100874058	5.871254895	3.7394027	8.003107131	2.958541694	3.919505936	1.997577452

100874079	6.394537636	5.7940988	6.994976515	3.727794797	5.24599209	4.486893444
100874083	-0.352108471	-0.352108	-0.352108471	1.631676003	-0.44942765	3.712779657
100874095	3.527916452	5.152872	1.902960944	0.570011001	4.899219042	2.734615022
100874123	8.633374294	9.4213439	9.027359077	7.438805957	7.419406972	7.458204942
100874126	-0.352108471	-0.352108	-0.352108471	1.793112977	1.646479888	1.939746065
100874151	1.383520155	-0.352108	3.119148782	2.53473567	5.421579811	-0.352108471
100874167	-0.352108471	-0.352108	-0.352108471	5.133308083	5.438960411	5.744612738
100874198	5.156684974	5.3130538	5.234869379	1.631676003	3.615460478	-0.352108471
100874211	-0.352108471	-0.352108	-0.352108471	3.366285451	2.252618212	1.138950972
100874214	3.761131874	5.7925837	1.729680021	2.379793093	6.863760789	4.621776941
100874243	9.580558577	7.9505586	6.32055856	7.883223243	6.682822443	5.482421642
100874278	-0.352108471	-0.352108	-0.352108471	6.111249662	3.923023209	5.017136436
100874300	5.04287137	4.0495704	3.056269331	-0.352108471	-0.352108471	-0.352108471
100874370	-0.352108471	-0.352108	-0.352108471	4.557059531	6.717533827	5.637296679
100885776	11.43752948	15.037546	13.23753795	12.31098812	13.22493522	12.76796167
100885779	5.117879811	5.6641565	5.391018164	5.002357752	5.984600755	5.493479254
100885795	4.782630369	8.6110951	6.696862741	3.957701248	4.66152415	5.365347052
100885848	5.6678354	4.8505978	4.033360215	1.731739118	1.731739118	1.731739118
100886964	7.069285813	8.1481099	9.226934059	5.587484762	10.07320548	7.830345121
100887750	6.074601444	5.3089485	4.543295563	4.200472614	3.401368727	3.80092067
100996249	-0.352108471	-0.352108	-0.352108471	4.247194404	5.253374746	6.259555088
100996251	8.044216423	8.4413207	7.647112169	6.573293172	7.10166618	7.630039188
100996273	5.869175118	9.9939326	7.931553871	4.529904824	8.008689914	6.269297369
100996279	6.519673958	4.1228842	8.916463723	6.413268661	4.792646314	3.172023967
100996291	10.22675527	4.2776579	7.252206581	2.553539015	7.050630806	4.80208491
100996295	5.032668174	6.1515856	7.270503083	5.479933443	5.899220491	5.060646394
100996307	5.284974356	5.7539737	4.815974971	-0.352108471	-0.352108471	-0.352108471
100996338	-0.352108471	-0.352108	-0.352108471	5.821410838	5.23896625	6.403855425
100996447	6.074121915	5.2110495	6.937194367	5.411542568	5.388370172	5.365197775
100996521	-0.352108471	-0.352108	-0.352108471	3.903344077	-0.700670423	1.601336827
100996541	4.121846117	3.4216545	4.822037705	0.975533543	0.808855576	1.14221151
100996573	5.888869469	5.8617144	5.834559354	1.5207156	2.232575625	0.808855576
100996582	6.191190925	7.6165571	4.765824798	4.156393209	7.040995723	9.925598237
100996598	5.613270467	6.8461728	6.229721613	1.72397541	3.198539975	4.673104541
100996624	-0.352108471	-0.352108	-0.352108471	4.188977125	5.169157826	6.149338526
100996681	5.268109931	4.7139222	4.159734512	0.228373553	0.808855576	-0.352108471
100996693	-0.352108471	-0.352108	-0.352108471	4.39174529	0.017007562	2.204376426
100996702	7.678496069	6.4023916	8.954600531	5.312246344	6.12823701	6.944227676
100996713	-0.352108471	-0.352108	-0.352108471	4.91770573	4.324959533	5.510451928
100996717	4.186705969	5.2174911	6.24827631	-0.352108471	-0.352108471	-0.352108471
100996724	-0.352108471	-0.352108	-0.352108471	6.074134525	5.776694563	5.479254601
100996738	5.611850338	5.2068303	6.01687034	-0.352108471	-0.352108471	-0.352108471
100996930	-0.352108471	-0.352108	-0.352108471	6.532441983	3.660133932	9.404750034
101059936	4.975875769	4.5788011	4.181726524	-0.352108471	-0.352108471	-0.352108471
101060017	1.392109249	3.136327	-0.352108471	2.039485684	-0.352108471	4.43107984
101060097	6.214743625	7.4900233	4.939463991	0.044132154	-0.352108471	0.440372779
101060177	3.660099283	5.7677452	4.713922221	-0.352108471	-0.352108471	-0.352108471

101060187	7.150675788	5.7494021	4.348128441	-0.352108471	-0.352108471	-0.352108471
101060226	-0.352108471	-0.352108	-0.352108471	2.169424975	1.23880364	3.100046309
101060254	5.688622647	6.3217343	5.055511004	-0.352108471	-0.352108471	-0.352108471
101060275	6.448663762	6.1995956	5.950527502	0.044132154	0.440372779	-0.352108471
101060322	7.459870921	3.2436936	5.351782282	1.58479924	1.437859363	1.731739118
101060328	3.127737875	5.0059245	1.249551269	0.24978	2.605817304	4.961854607
101060373	-0.352108471	-0.352108	-0.352108471	1.880238567	-0.352108471	4.112585606
101060398	-0.352108471	-0.352108	-0.352108471	5.64354012	5.023044807	6.264035433
101060429	6.571654576	5.3085806	4.045506658	5.86372365	5.881326627	5.898929603
101060445	6.962165162	10.855734	8.908949447	7.118409184	4.811572158	9.42524621
101060542	3.124254617	4.7970985	3.960676558	3.828080288	0.663999426	6.99216115
101060604	3.41407487	3.136327	3.69182277	0.82273449	1.997577452	-0.352108471
101060691	3.245235305	3.136327	3.354143641	2.301055037	4.954218546	-0.352108471
101060825	2.065292044	-0.352108	4.482692559	0.793818797	1.939746065	-0.352108471
101101775	-0.352108471	-0.352108	-0.352108471	5.287421216	5.116246503	5.201833859
101243545	-0.352108471	-0.352108	-0.352108471	2.577576529	2.796089998	2.686833264
101243555	7.133445996	4.8711713	6.002308672	-0.352108471	-0.352108471	-0.352108471
101409256	7.428721334	7.889817	6.967625619	10.35776112	3.599227082	6.978494102
101409258	4.814358965	3.9997511	5.628966845	7.448532665	6.635453109	5.822373554
101410538	-0.352108471	-0.352108	-0.352108471	3.543647175	8.958859847	6.251253511
101410541	3.414598071	8.9979527	6.206275376	4.219673638	9.013654677	6.616664157
101669764	4.731111372	4.8304259	4.631796822	1.640731613	1.731739118	1.549724107
101669767	7.457846733	7.3046105	7.151374206	7.697567401	7.129654683	6.561741966
101752400	-0.352108471	-0.352108	-0.352108471	3.416955113	-0.153603106	1.631676003
101805492	7.9900573	7.9448756	8.035239038	7.846725922	7.837892631	7.855559213
101867536	10.49227718	9.2732507	8.054224215	4.552376253	10.70604843	7.629212341
101926889	9.907133664	10.788949	11.67076398	7.650460345	9.214229956	10.77799957
101926890	-0.352108471	-0.352108	-0.352108471	1.901473368	2.250571141	2.076022254
101926897	5.32386623	4.1838641	4.753865155	8.201527569	2.706462118	5.453994843
101926898	13.53861591	10.184414	11.86151496	10.65900951	10.63610231	10.6131951
101926907	6.644956305	7.7592588	8.873561387	6.528404389	9.534341235	3.522467543
101926908	3.926167062	7.7315299	5.828848464	-0.352108471	-0.352108471	-0.352108471
101926911	2.718924682	5.6884362	8.657947775	1.518484901	1.731739118	1.305230684
101926915	6.86259906	6.6215439	7.103654244	4.918926546	4.482692559	5.355160532
101926918	-0.352108471	-0.352108	-0.352108471	4.66005769	3.701594535	5.618520844
101926928	7.535667186	5.1195655	9.951768838	6.113029435	5.647333551	6.57872532
101926930	5.198808809	5.4836661	5.768523368	-0.352108471	-0.352108471	-0.352108471
101926935	7.00268	7.7974494	6.207910571	8.481573421	6.165870606	3.850167791
101926953	2.65478974	-0.352108	5.661687951	-0.352108471	-0.352108471	-0.352108471
101926955	4.940612622	3.2746177	6.606607529	4.645563652	6.298295008	5.47192933
101926958	-0.352108471	-0.352108	-0.352108471	3.264579603	6.926268373	5.095423988
101926960	3.209772045	8.7675562	5.988664101	1.981180367	0.75077525	3.211585483
101926969	6.966795335	8.3284377	7.64761654	-0.352108471	-0.352108471	-0.352108471
101926984	-0.352108471	-0.352108	-0.352108471	5.88700099	7.295806839	4.478195141
101926989	-0.352108471	-0.352108	-0.352108471	2.397492216	-0.352108471	5.147092904
101926996	8.47548589	7.7187703	9.232201477	6.06683161	7.007197297	7.947562984
101927009	5.677239679	5.825168	5.529311366	1.640731613	1.731739118	1.549724107

101927016	4.600159022	6.3691566	8.138154159	-0.352108471	-0.352108471	-0.352108471
101927021	1.501017585	-0.352108	3.354143641	8.06546056	6.796814879	5.528169199
101927030	-0.352108471	-0.352108	-0.352108471	10.96152105	2.113246397	6.537383725
101927032	1.737439106	8.6417942	5.189616664	0.624614178	0.440372779	0.808855576
101927034	10.86368975	13.051971	8.675408313	9.153716668	9.546259259	9.349987964
101927040	3.636824788	3.9195059	3.354143641	1.218975116	0.440372779	1.997577452
101927045	5.522595798	4.976516	6.068675629	-0.352108471	-0.352108471	-0.352108471
101927074	6.017940363	6.5297242	6.273832259	-0.352108471	-0.352108471	-0.352108471
101927088	-0.352108471	-0.352108	-0.352108471	6.396868383	6.410585269	6.383151496
101927094	7.709644288	7.2146729	6.719701551	5.615175082	6.610868188	7.606561295
101927131	6.290659851	4.8020746	5.546367231	6.238407109	7.360256897	5.116557322
101927132	5.569305896	8.0049622	6.787134073	3.656418144	1.997577452	5.315258837
101927136	6.211223246	6.5021238	6.793024285	8.33904221	4.726070399	6.532556305
101927150	5.953091712	7.1319092	4.774274269	6.214551203	5.474812883	6.954289524
101927152	6.199130262	7.5522806	4.845979904	5.009972817	2.388028312	3.699000565
101927157	7.76039587	3.3664563	5.563426062	8.867439673	5.552726144	2.238012615
101927166	5.027756401	6.2848227	5.656289537	1.427477396	-0.202941035	3.057895826
101927178	1.703814606	2.4868796	3.269944576	1.624492476	3.698088569	5.771684661
101927181	6.522346096	4.6973571	8.347335051	6.795027168	4.641917167	8.948137169
101927190	-0.352108471	-0.352108	-0.352108471	4.745251399	5.017136436	5.289021472
101927228	1.383520155	-0.352108	3.119148782	2.19617771	4.559699198	6.923220687
101927257	8.442468668	7.1258056	5.809142469	6.050474908	4.186838295	5.118656602
101927274	6.67322099	7.9295946	5.416847378	1.82202867	1.646479888	1.997577452
101927279	6.809538378	8.7559248	10.70231129	6.201175754	7.469697771	8.738219789
101927292	2.740210233	3.0290686	2.451351903	-0.352108471	-0.352108471	-0.352108471
101927325	-0.352108471	-0.352108	-0.352108471	2.397492216	-0.352108471	5.147092904
101927334	2.419557138	-0.352108	5.191222747	-0.352108471	-0.352108471	-0.352108471
101927337	12.05978117	12.059781	12.05978117	12.4424511	12.41516276	12.46973944
101927339	6.589691005	5.6691055	7.510276468	3.480869363	1.646479888	5.315258837
101927340	6.867028007	6.9515466	6.78250938	3.38836593	0.63603458	2.012200255
101927357	6.355926573	5.5909139	4.825901136	3.447009639	3.680662938	3.914316237
101927359	4.755240115	5.7903812	3.720099057	1.640731613	1.549724107	1.731739118
101927374	11.50148523	11.470359	11.53261106	10.44579859	10.45249577	10.4391014
101927378	-0.352108471	-0.352108	-0.352108471	4.625102937	5.230255493	4.927679215
101927401	6.573968999	6.5884653	6.559472732	-0.352108471	-0.352108471	-0.352108471
101927415	8.862492035	6.2680756	7.56528383	9.605988581	2.861935094	6.233961838
101927419	5.990131758	5.0635146	5.526823203	5.219123492	6.737999171	3.700247812
101927423	5.876327432	6.552591	6.214459212	1.14221151	1.14221151	1.14221151
101927446	1.863947334	1.3921092	0.920271164	4.116820349	1.845238787	-0.426342774
101927447	5.344042808	6.1509366	6.95783045	0.843916758	3.035138754	1.939527756
101927452	6.788420481	6.4667543	7.110086689	6.536811004	4.984263142	5.760537073
101927476	6.708652355	9.4527399	8.080696104	7.616827828	7.411978511	7.207129195
101927479	6.434445821	6.3439771	6.524914536	2.602306905	2.051108653	3.153505157
101927522	6.625642623	4.7909718	5.708307224	-0.352108471	-0.352108471	-0.352108471
101927526	-0.352108471	-0.352108	-0.352108471	2.065292044	2.727751458	1.40283263
101927528	9.653471396	7.0872306	4.520989756	4.755602447	6.863410361	5.809506404
101927550	8.93296241	2.0059169	5.469439637	0.044132154	-0.352108471	0.440372779

101927556	3.127737875	3.136327	3.119148782	5.494417177	2.714559209	8.274275145
101927559	3.594841197	5.0363099	4.315575532	7.91322227	6.900899666	5.888577061
101927562	4.323396426	5.2849744	6.246552286	-0.352108471	-0.352108471	-0.352108471
101927575	7.164717728	7.4734848	7.319101259	6.0669142	6.721284839	7.375655477
101927579	-0.352108471	-0.352108	-0.352108471	3.169772494	2.187592805	2.678682649
101927580	8.174508714	7.007949	5.841389267	6.351126145	6.42302191	6.279230379
101927585	-0.352108471	-0.352108	-0.352108471	5.017136436	3.039297998	6.994974873
101927587	1.356017291	2.874763	4.39350866	0.624614178	0.440372779	0.808855576
101927588	5.157610748	4.4826926	5.832528937	1.218975116	1.997577452	0.440372779
101927595	-0.352108471	-0.352108	-0.352108471	1.336474202	2.232575625	0.440372779
101927598	6.002308672	5.7573212	6.247296179	2.121209733	2.742246626	1.50017284
101927606	7.231153032	4.9504636	6.090808341	-0.352108471	-0.352108471	-0.352108471
101927610	4.582371975	6.4450275	8.307682927	4.72458994	5.335500499	4.113679381
101927615	-0.352108471	-0.352108	-0.352108471	1.968661759	1.997577452	1.939746065
101927619	2.567643063	0.1993972	1.383520155	1.239592382	2.847531327	4.455470273
101927623	2.413921152	-0.211251	5.039093469	4.036683664	2.560176267	5.51319106
101927652	6.677060206	3.8928885	5.284974356	-0.352108471	-0.352108471	-0.352108471
101927657	3.779740629	3.5846224	3.682181533	5.810583651	4.942702387	4.074821124
101927666	6.851407316	7.3180325	7.784657702	3.123303237	6.215206307	9.307109378
101927667	6.640019393	7.2961404	5.983898352	2.886671685	6.027100714	4.456886199
101927671	2.201036472	-0.352108	4.754181416	1.727216326	1.807952763	1.646479888
101927678	7.149902426	2.6064378	4.878170137	5.258845516	1.772255056	3.515550286
101927691	8.104788488	6.7629397	7.433864099	-0.352108471	-0.352108471	-0.352108471
101927693	-0.352108471	-0.352108	-0.352108471	1.268079384	2.299318081	1.783698732
101927720	-0.352108471	-0.352108	-0.352108471	6.188308708	6.47267644	6.757044173
101927723	5.038089054	4.617923	5.458255064	1.646479888	1.646479888	1.646479888
101927740	8.399519058	7.4572022	9.341835904	7.789279214	7.351399985	8.227158443
101927741	-0.352108471	-0.352108	-0.352108471	2.521016528	5.317995343	3.919505936
101927750	4.805894371	5.4144052	4.197383579	-0.352108471	-0.352108471	-0.352108471
101927752	-0.352108471	-0.352108	-0.352108471	2.371379612	1.90830372	1.445227827
101927760	-0.352108471	-0.352108	-0.352108471	3.245625856	3.337746555	3.153505157
101927767	-0.352108471	-0.352108	-0.352108471	5.017136436	3.57759874	6.456674131
101927770	7.121123024	3.3358418	5.228482407	3.61935986	0.009810316	1.814585088
101927774	6.035394867	6.5688561	7.102317432	3.119148782	2.758457771	3.479839792
101927788	-0.352108471	-0.352108	-0.352108471	7.230662479	4.57685853	5.903760504
101927789	-0.352108471	-0.352108	-0.352108471	1.939639127	2.147539136	1.731739118
101927793	-0.352108471	-0.352108	-0.352108471	4.330998279	6.320433877	5.325716078
101927796	7.108246235	10.942575	9.02541086	7.532837054	5.317328239	9.748345868
101927797	7.439907027	6.3369206	6.888413829	6.543375401	3.554622972	5.048999186
101927806	-0.352108471	-0.352108	-0.352108471	7.554020345	2.958515671	5.256268008
101927824	-0.352108471	-0.352108	-0.352108471	4.08236011	7.38205614	0.782664081
101927837	-0.352108471	-0.352108	-0.352108471	2.770666903	5.893442277	-0.352108471
101927841	5.852937462	6.0671629	5.638712004	-0.352108471	-0.352108471	-0.352108471
101927877	1.904923171	3.2722797	2.588601447	0.228373553	0.808855576	-0.352108471
101927881	8.430581625	8.4455387	8.415624566	1.631676003	3.615460478	-0.352108471
101927884	5.877498426	7.3947613	8.912024117	6.709520631	5.708761154	7.710280107
101927889	-0.352108471	-0.352108	-0.352108471	2.481575183	5.315258837	-0.352108471

101927896	4.107432644	5.3211461	6.534859572	2.209286191	1.049448818	3.369123563
101927901	4.757610744	6.8526205	2.662600944	2.342793532	4.245214285	0.440372779
101927902	5.529311366	4.8148641	6.243758652	-0.352108471	-0.352108471	-0.352108471
101927911	-0.352108471	-0.352108	-0.352108471	1.783698732	-0.352108471	3.919505936
101927914	5.089412202	6.5161245	3.662699872	-0.352108471	-0.352108471	-0.352108471
101927915	-0.352108471	-0.352108	-0.352108471	1.997577452	1.44635305	2.548801854
101927924	4.215160124	7.0148176	1.415502606	5.048433266	8.532601271	1.564265262
101927944	6.001725177	7.816155	4.18729534	5.468276192	4.075630311	6.860922073
101927952	4.404049874	5.3546856	6.305321424	-0.352108471	-0.352108471	-0.352108471
101927954	6.803513645	9.0988796	4.508147684	4.312962502	6.868687233	5.590824867
101927964	6.061329067	6.8991656	7.73700222	-0.352108471	-0.352108471	-0.352108471
101927973	7.049086746	4.7049241	5.877005427	9.442431921	5.687745613	1.933059306
101927980	2.687757291	4.0160458	5.344334251	6.380291785	5.704335255	5.028378725
101927994	6.177699785	6.3755257	6.276612754	5.82826691	3.336476406	8.320057414
101927998	5.413210729	7.380526	6.396868383	-0.352108471	-0.352108471	-0.352108471
101928002	-0.352108471	-0.352108	-0.352108471	5.369332089	5.383503645	5.355160532
101928004	5.548815533	5.692967	5.620891261	4.09347548	5.673482525	7.25348957
101928020	0.783465188	3.7077348	2.245600013	1.946552907	3.54819548	0.344910334
101928026	5.428393874	5.4582551	5.398532684	-0.352108471	-0.352108471	-0.352108471
101928033	5.976439646	2.4538806	4.215160124	0.044132154	0.440372779	-0.352108471
101928045	-0.352108471	-0.352108	-0.352108471	5.075427365	7.396414957	6.235921161
101928053	4.842468271	5.5957787	5.219123492	-0.352108471	-0.352108471	-0.352108471
101928068	5.698464481	5.4498875	5.201310554	3.482176795	4.153941194	3.818058994
101928069	3.555243815	5.3121178	4.433680789	-0.352108471	-0.352108471	-0.352108471
101928079	9.376792143	7.8957769	6.414761735	6.43602752	8.120827043	4.751227997
101928081	9.60662593	3.8608111	6.733718494	6.102475591	5.300187985	4.497900379
101928099	8.061120123	6.6395097	7.35031491	3.751349834	2.147539136	5.355160532
101928101	5.444754735	5.0677813	5.256268008	2.61236831	1.305230684	3.919505936
101928103	7.496618137	4.77306	10.22017626	6.710150397	6.913091351	6.811620874
101928104	6.846957097	4.2158087	9.478105461	4.963223552	5.72472801	6.486232468
101928113	3.486766	4.2597237	5.032681328	-0.352108471	-0.352108471	-0.352108471
101928118	9.812832381	9.8716874	9.753977346	9.340245741	9.11942662	8.898607499
101928120	-0.352108471	-0.352108	-0.352108471	4.950307598	6.089864204	7.229420811
101928123	3.744544946	6.1494694	4.947007189	-0.352108471	-0.352108471	-0.352108471
101928131	2.803125457	5.9583594	-0.352108471	1.540978788	1.14221151	1.939746065
101928137	9.712905877	9.8306575	9.771781667	7.933420402	7.837847273	7.885633837
101928144	5.810306839	7.5460452	9.28178347	7.330596441	5.599098969	3.867601498
101928150	7.566164052	7.5166011	7.467038146	5.091220567	7.926103816	2.256337317
101928151	7.288433404	6.684971	6.986702195	7.360533746	5.945032052	6.652782899
101928158	0.388235613	2.4815752	4.574914752	-0.352108471	-0.352108471	-0.352108471
101928169	-0.352108471	-0.352108	-0.352108471	2.501526031	3.182714307	1.820337755
101928174	6.735151691	2.4194539	4.577302775	5.687600059	6.711928096	4.663272021
101928176	-0.352108471	-0.352108	-0.352108471	2.670502348	-0.352108471	5.693113166
101928189	5.656120661	4.0177946	4.836957653	6.295633508	5.271165901	7.320101116
101928201	-0.352108471	-0.352108	-0.352108471	5.67093521	6.149337851	6.627740491
101928202	-0.352108471	-0.352108	-0.352108471	5.958359385	5.958359385	5.958359385
101928228	5.372241581	4.4826926	6.261790604	5.511864271	5.494417177	5.529311366

101928231	1.383520155	0.2535657	2.513474608	3.883156074	6.190193949	1.576118198
101928241	5.376563746	7.96909	2.784037471	0.808855576	0.808855576	0.808855576
101928254	3.902204188	4.1125856	3.69182277	5.819619791	5.191222747	6.448016836
101928255	8.116007685	5.8188936	6.967450619	4.449748927	4.102036274	4.79746158
101928295	5.852491963	2.3149862	9.389997758	2.868155262	2.518741013	3.217569511
101928303	6.50351982	5.2529092	4.002298608	5.523239719	6.172398222	4.874081216
101928309	3.945254192	3.136327	4.754181416	0.228373553	-0.352108471	0.808855576
101928318	6.537383725	5.6396255	7.435141923	4.883715006	9.764015434	7.32386522
101928331	-0.352108471	-0.352108	-0.352108471	5.923493182	8.01270237	3.834283995
101928336	10.30683545	8.14045	5.97406461	6.828129341	7.681857993	5.974400688
101928344	6.991795048	8.3031412	5.680448889	-0.352108471	-0.352108471	-0.352108471
101928368	-0.352108471	-0.352108	-0.352108471	3.119148782	3.348032409	2.890265154
101928372	5.196577314	5.4960088	4.897145843	8.559174957	6.382707331	4.206239706
101928381	5.111021431	4.28869	4.699855738	0.044132154	-0.352108471	0.440372779
101928391	6.104835944	5.193245	5.649040476	0.044132154	0.440372779	-0.352108471
101928401	7.623288714	9.5714111	5.67516629	5.780173853	6.223773372	6.66737289
101928423	4.249261192	2.6341643	5.864358109	1.744484008	2.051108653	1.437859363
101928435	-0.352108471	-0.352108	-0.352108471	4.456886199	5.592695754	3.321076645
101928440	5.59960682	5.6803006	5.518913025	-0.352108471	-0.352108471	-0.352108471
101928446	4.543008221	5.5936428	6.644277383	0.782103376	1.478197356	2.174291337
101928453	6.235994735	3.5213362	4.878665478	-0.352108471	-0.352108471	-0.352108471
101928483	5.743835375	5.5293114	5.958359385	-0.352108471	-0.352108471	-0.352108471
101928495	2.560163873	2.7115616	2.862959239	0.044132154	-0.352108471	0.440372779
101928496	6.263611604	7.6163863	4.910836907	0.986043316	7.694323943	4.340183629
101928514	10.12910836	9.0782351	8.027361906	7.983005582	8.018349163	7.947662002
101928525	2.711464377	5.1146406	7.517816919	-0.352108471	-0.352108471	-0.352108471
101928526	5.835210261	6.1131757	5.557244839	6.977708203	7.919034787	6.036381618
101928527	4.113141693	6.7474099	5.430275811	7.859486648	5.511044393	6.685265521
101928545	-0.352108471	-0.352108	-0.352108471	5.88700099	5.089621259	6.68438072
101928550	6.844298182	9.2277515	4.460844902	8.059855992	7.400550537	6.741245083
101928555	4.821115482	7.0783904	5.949752937	6.856794704	0.24275261	3.549773657
101928565	7.12950969	8.0787243	6.180295037	6.088371026	7.957752778	4.218989274
101928567	4.07445589	5.7536871	4.914071475	5.64753996	5.460434222	5.273328484
101928574	6.780548039	3.2359077	5.008227864	1.842407068	1.876861374	1.807952763
101928578	4.433680789	3.6761388	5.191222747	0.044132154	-0.352108471	0.440372779
101928580	4.156475292	4.2452143	4.333953278	1.979475812	2.011378906	1.995427359
101928583	7.037798976	8.3124918	5.763106103	0.044132154	-0.352108471	0.440372779
101928597	5.586919941	4.9799919	6.193847962	1.591046029	1.305230684	1.876861374
101928600	10.25478152	11.54303	8.966533538	9.326288657	9.845835113	8.8067422
101928625	1.392109249	2.3571814	0.42703708	2.124237496	3.703340207	0.545134784
101928632	7.561981411	5.4582551	3.354528717	-0.352108471	-0.352108471	-0.352108471
101928669	6.020327111	6.055636	6.03798158	5.903760504	8.494417256	3.313103753
101928684	8.747825592	6.8454429	7.796634271	5.203968979	6.905740203	8.607511426
101928694	4.297639083	6.9925329	1.602745308	5.855547981	6.232107317	5.478988644
101928704	2.44582553	1.5597239	3.331927181	-0.352108471	-0.352108471	-0.352108471
101928710	4.141031582	8.4658909	6.303461245	-0.352108471	-0.352108471	-0.352108471
101928716	7.452723153	7.352529	7.552917276	5.141833898	4.245214285	6.038453511

101928720	7.946182748	4.7461416	6.346162167	-0.352108471	-0.352108471	-0.352108471
101928725	5.544862015	2.3456464	3.945254192	1.668505151	3.03366733	2.351086241
101928726	6.517963851	6.531646	6.504281698	0.727922146	1.807952763	-0.352108471
101928733	1.392109249	2.8925343	-0.108315788	2.887607814	3.11247051	3.000039162
101928738	5.110010598	4.7518672	5.468153966	0.805486251	1.769397609	2.733308968
101928752	5.657493881	5.6532998	5.661687951	-0.352108471	-0.352108471	-0.352108471
101928760	4.072205287	5.2849744	6.497743425	-0.352108471	-0.352108471	-0.352108471
101928766	5.321252663	5.5882244	5.054280952	6.12990004	6.135204448	6.124595632
101928786	4.864842617	6.1215288	7.378214921	0.812748712	2.432228037	1.622488375
101928792	-0.352108471	-0.352108	-0.352108471	2.124237496	2.147539136	2.100935855
101928794	8.459617393	4.7551375	6.607377459	-0.352108471	-0.352108471	-0.352108471
101928795	6.304886683	6.5896867	6.874486772	1.704340373	3.27996028	4.855580186
101928796	-0.352108471	-0.352108	-0.352108471	5.017136436	5.054280952	4.979991919
101928808	7.363560518	5.5869199	3.810279363	3.488784686	1.997577452	4.979991919
101928809	5.089412202	5.1245435	5.054280952	6.378900911	6.532588736	6.225213087
101928818	-0.352108471	-0.352108	-0.352108471	5.284974356	4.868036798	5.701911914
101928841	7.018254481	6.7066286	7.329880387	6.328089748	3.446196717	9.209982779
101928844	2.483048095	1.277429	1.880238567	3.46613827	4.569815273	2.362461267
101928845	5.937827329	5.873346	6.002308672	0.440372779	0.440372779	0.440372779
101928846	8.831681515	4.9422224	6.886951948	4.537270288	7.459806893	5.99853859
101928847	6.537146105	6.8840608	6.190231414	0.737349958	3.721385883	2.22936792
101928848	6.005023479	5.1125123	5.55876787	-0.352108471	-0.352108471	-0.352108471
101928855	3.715642943	7.3672599	5.541451418	1.831534952	3.668632064	5.505729177
101928857	6.184348037	5.6656669	5.925007489	3.196038955	4.954218546	1.437859363
101928868	9.209884796	4.0047484	6.607316614	5.420684135	4.445477722	3.470271309
101928875	-0.352108471	-0.352108	-0.352108471	5.980334028	6.904792035	5.055876022
101928876	-0.352108471	-0.352108	-0.352108471	6.458036567	2.571154353	-1.315727861
101928879	5.939192221	7.1110404	4.767344022	-0.352108471	-0.352108471	-0.352108471
101928885	6.090808341	6.5188701	5.662746571	-0.352108471	-0.352108471	-0.352108471
101928887	8.051116643	6.0558619	7.053489274	5.449524003	2.887004166	0.324484329
101928891	-0.352108471	-0.352108	-0.352108471	3.475323526	6.862992125	5.169157826
101928906	-0.352108471	-0.352108	-0.352108471	3.402206991	0.06583992	6.738574063
101928933	6.40767526	5.2788202	7.536530271	7.661993031	6.871609123	6.081225215
101928936	11.79921455	10.815781	9.832348147	8.815571768	7.728884677	9.90225886
101928946	4.834101937	2.7058213	6.96238253	3.563451397	2.732871542	3.148161469
101928962	4.649747619	5.054281	4.245214285	6.065196634	6.355161685	5.775231583
101928969	3.530238889	5.6641722	7.798105474	-0.352108471	-0.352108471	-0.352108471
101928978	2.84317252	2.995873	2.690472086	0.975533543	1.14221151	0.808855576
101928992	6.22851478	5.0087546	3.78899432	2.913739289	3.922118404	3.417928847
101928998	-0.352108471	-0.352108	-0.352108471	0.622639271	5.978059164	3.300349217
101928999	5.112273114	7.1110017	9.109730209	-0.352108471	-0.352108471	-0.352108471
101929010	6.494190852	6.1999612	6.788420481	3.027377867	5.124543452	0.930212283
101929025	2.981302042	3.5151412	4.04898043	1.873849414	1.807952763	1.939746065
101929057	3.601141313	6.4889106	5.045025943	0.791292145	1.14221151	0.440372779
101929059	7.072509339	6.9037827	7.241236027	6.218511826	6.101634422	6.33538923
101929066	2.981526684	4.5220247	6.062522703	1.981180367	0.808855576	3.153505157
101929083	5.493783215	5.5293114	5.458255064	-0.352108471	-0.352108471	-0.352108471

101929086	8.076681806	8.1193837	8.162085602	6.072053441	6.985674563	7.899295684
101929112	-0.352108471	-0.352108	-0.352108471	6.200126558	4.456886199	2.713645841
101929123	-0.352108471	-0.352108	-0.352108471	6.001809131	6.08127149	5.922346773
101929130	7.860152705	7.3590339	7.609593315	7.310661511	5.574700788	3.838740065
101929144	6.395056727	5.9414478	5.487838919	0.791292145	0.440372779	1.14221151
101929147	7.044455835	6.9037827	7.18512902	0.044132154	0.440372779	-0.352108471
101929152	8.507400971	7.4746577	6.441914365	6.072606262	7.006051904	7.939497545
101929153	4.421820181	5.4283939	6.434967567	-0.352108471	-0.352108471	-0.352108471
101929159	1.190868475	4.5497284	7.908588236	0.326055216	2.528899575	1.427477396
101929165	8.423783094	6.648528	7.536155555	5.256221592	7.571823439	9.887425286
101929181	6.074755128	6.7518845	7.429013781	1.981180367	0.808855576	3.153505157
101929184	-0.352108471	-0.352108	-0.352108471	0.874413692	-0.352108471	2.100935855
101929194	-0.352108471	-0.352108	-0.352108471	6.801058014	4.486022226	5.64354012
101929199	8.186101763	7.9701803	7.754258871	7.406278031	11.54073697	3.271819091
101929200	-0.352108471	-0.352108	-0.352108471	4.774498542	4.482692559	5.066304524
101929206	-0.352108471	-0.352108	-0.352108471	0.849500091	-0.352108471	2.051108653
101929216	3.805664353	3.4358082	4.175520484	2.049256654	2.100935855	1.997577452
101929224	5.91576742	5.7770771	6.05445777	1.58479924	1.731739118	1.437859363
101929227	6.263974257	6.9076731	5.620275462	4.155185764	6.562251917	1.748119611
101929230	5.656289537	5.9103134	5.402265645	-0.352108471	-0.352108471	-0.352108471
101929259	5.804767386	7.6044747	6.704621053	5.658097137	8.227591779	6.942844458
101929260	5.933381578	6.3324667	6.731551759	-0.352108471	-0.352108471	-0.352108471
101929279	3.511586554	6.6672378	5.089412202	1.30840417	1.807952763	0.808855576
101929295	6.666109932	6.5150311	6.817188811	2.179939357	0.440372779	3.919505936
101929301	3.662141459	1.3842828	2.523212107	3.073227812	4.32549163	3.699359721
101929329	4.20130528	6.6073775	9.013449638	-0.352108471	-0.352108471	-0.352108471
101929333	8.363953381	10.081332	11.79870992	9.187054383	8.313629059	8.750341721
101929352	5.986643059	5.0554016	6.917884546	4.481844932	7.337535782	1.626154082
101929372	4.618564529	3.7280709	5.509058121	1.80041638	2.051108653	1.549724107
101929378	4.372191214	2.627595	6.116787462	2.211943219	2.232575625	2.191310813
101929380	5.226406818	5.054281	5.398532684	2.655229797	2.191310813	3.119148782
101929390	7.075097789	7.848492	8.621886177	5.544866226	6.432145005	7.319423784
101929398	5.974855592	6.8917819	6.433318728	-0.352108471	-0.352108471	-0.352108471
101929400	4.549728355	6.2861288	2.8133279	-0.352108471	-0.352108471	-0.352108471
101929402	7.077963787	6.5644614	6.050959007	6.571028909	6.398570076	6.743487742
101929415	9.488445266	9.3131537	9.663736784	8.698996908	8.759587648	8.638406169
101929418	5.331510862	4.4868934	3.642276026	0.689815323	-0.352108471	1.731739118
101929425	2.588601447	2.8524293	2.32477359	-0.352108471	-0.352108471	-0.352108471
101929426	7.667941511	6.6002756	5.532609778	4.609641414	3.245625856	1.881610298
101929431	8.198489481	6.4727957	9.92418329	8.018068611	7.023663373	7.520865992
101929439	5.0702182	6.5301814	5.800199788	-0.352108471	-0.352108471	-0.352108471
101929445	-0.352108471	-0.352108	-0.352108471	5.169157826	6.60418997	3.734125681
101929450	5.967012314	6.4772569	6.222134608	3.308014316	1.549724107	5.066304524
101929482	3.682035383	6.5472459	5.114640648	-0.352108471	-0.352108471	-0.352108471
101929486	3.127737875	3.136327	3.119148782	4.288701923	5.458255064	3.119148782
101929497	0.736119179	3.9660533	2.351086241	0.044132154	-0.352108471	0.440372779
101929511	5.95664934	6.5341125	7.111575689	5.615586825	7.054970771	6.335278798

101929519	-0.352108471	-0.352108	-0.352108471	2.221964512	2.252618212	2.191310813
101929526	-0.352108471	-0.352108	-0.352108471	6.453264303	4.73755266	3.021841017
101929528	6.369156591	7.955428	4.782885208	-0.352108471	-0.352108471	-0.352108471
101929540	2.277882245	4.5174054	3.397643806	3.304563757	2.681724436	2.058885116
101929542	7.204955222	4.3455079	5.775231583	-0.352108471	-0.352108471	-0.352108471
101929550	5.648033597	5.3324918	5.490262688	2.893883306	6.992603391	4.943243349
101929567	12.14051218	11.177295	11.65890366	9.078360757	10.34897984	11.61959892
101929586	6.783937768	9.0963346	7.940136183	5.892315348	4.416882669	7.367748028
101929614	-0.352108471	-0.352108	-0.352108471	3.919505936	3.919505936	3.919505936
101929634	7.914178495	2.3983766	5.156277572	3.350303694	1.939746065	4.760861323
101929645	5.326927409	5.1384716	5.515383174	0.791292145	1.14221151	0.440372779
101929660	2.668345013	5.1227518	7.577158686	6.181883301	7.613507454	4.750259147
101929670	-0.352108471	-0.352108	-0.352108471	5.1471442	4.175292888	3.203441576
101929680	6.538498835	7.4209894	5.656008285	6.661679876	6.330059492	5.998439109
101929681	6.09184854	5.1800301	5.635939335	-0.352108471	-0.352108471	-0.352108471
101929688	-0.352108471	-0.352108	-0.352108471	3.756942025	4.91770573	6.078469436
101929690	6.640019393	7.0361566	6.243882221	-0.352108471	-0.352108471	-0.352108471
101929693	-0.352108471	-0.352108	-0.352108471	4.66005769	5.672236747	3.647878632
101929694	4.335919821	5.4066305	3.265209189	2.653997961	3.254329098	3.854660235
101929698	7.578270771	7.9883565	7.168185076	4.969163726	2.361348104	7.576979348
101929713	5.386535448	5.2437595	5.529311366	3.878699875	1.939746065	5.817653684
101929716	6.905153245	6.1515856	5.398018012	-0.352108471	-0.352108471	-0.352108471
101929717	5.951809919	6.3763797	5.527240124	-0.352108471	-0.352108471	-0.352108471
101929754	3.098457124	3.3920135	3.245235305	6.697849457	5.529311366	4.360773274
101929762	6.158865923	7.2654723	5.052259592	2.051108653	1.548142771	2.554074536
101929774	4.04402614	5.9945398	5.019282955	-0.352108471	-0.352108471	-0.352108471
101929796	6.89992346	6.9268019	6.873045007	6.580724432	6.759061079	6.402387785
101929806	-0.352108471	-0.352108	-0.352108471	5.44897977	7.587771569	3.310187972
101929814	5.664088163	6.0023087	5.325867653	-0.352108471	-0.352108471	-0.352108471
101929892	5.858161648	5.5755178	5.292873997	1.651404068	2.396536258	0.906271878
101929927	4.305750079	0.7406741	2.523212107	-0.352108471	-0.352108471	-0.352108471
101929941	7.485720019	6.7206966	5.955673123	7.120271332	5.492661445	3.865051557
101929946	5.742285457	4.863372	5.302828753	1.437859363	-0.024004841	2.899723567
101929959	6.020666958	6.8312892	7.641911457	-0.352108471	-0.352108471	-0.352108471
101929977	3.127737875	3.136327	3.119148782	4.201099247	4.482692559	3.919505936
101929988	-0.352108471	-0.352108	-0.352108471	4.178899946	4.245214285	4.112585606
101929998	-0.352108471	-0.352108	-0.352108471	3.725166081	3.337746555	4.112585606
101930028	-0.352108471	-0.352108	-0.352108471	0.793818797	1.939746065	-0.352108471
101930031	6.903443506	6.6708246	6.787134073	-0.352108471	-0.352108471	-0.352108471
101930047	1.950932319	7.241871	4.596401634	0.149726728	4.129961623	2.139844176
101930059	3.495206597	6.2621244	4.878665478	-0.352108471	-0.352108471	-0.352108471
101930067	6.020381091	7.2906614	4.750100767	0.440372779	0.440372779	0.440372779
101930075	5.073619131	8.8317955	6.952707338	-0.352108471	-0.352108471	-0.352108471
101930085	6.230614498	7.5626216	8.894628665	7.760375585	6.614103346	5.467831107
101930091	5.525038609	6.502263	7.479487447	-0.352108471	-0.352108471	-0.352108471
101930117	7.326578711	7.7375008	8.148422839	7.237837929	6.849035557	7.043436743
101930119	7.207838162	7.2305473	7.18512902	-0.352108471	-0.352108471	-0.352108471

101930123	5.085607333	3.1253464	7.045868219	4.027343717	2.879532747	1.731721778
101930125	-0.352108471	-0.352108	-0.352108471	8.531340208	5.939192221	3.347044235
101930129	4.296089635	2.6817244	1.067359237	-0.352108471	-0.352108471	-0.352108471
101930151	3.127737875	3.136327	3.119148782	4.439864799	5.315258837	6.190652875
101930164	8.20435406	5.2917962	2.379238258	-0.352108471	-0.352108471	-0.352108471
101930168	2.991761457	1.9108199	2.451290702	-0.352108471	-0.352108471	-0.352108471
101930452	-0.352108471	-0.352108	-0.352108471	0.854670495	2.124237496	3.393804497
101978719	5.029080614	8.4980293	6.763554971	7.016082108	6.543126016	6.070169923
101978785	6.526543088	7.2579542	7.989365282	6.785488968	4.561476082	5.673482525
102060414	-0.352108471	-0.352108	-0.352108471	1.77976078	3.119148782	0.440372779
102157401	-0.352108471	-0.352108	-0.352108471	1.400698343	-0.352108471	3.153505157
102157402	-0.352108471	-0.352108	-0.352108471	6.804723141	7.308739603	6.300706679
102467081	6.418225735	7.6458482	7.032036953	5.870213399	6.16990339	6.469593382
102578074	6.739878825	7.0629266	6.41683103	0.044132154	-0.352108471	0.440372779
102606463	2.718593827	3.2452353	3.771876783	6.474985635	2.63489202	4.554938827
102659353	4.161342354	7.0144707	5.587906531	1.988898614	2.100935855	1.876861374
102723099	8.71290153	6.4975644	7.605232944	6.679108481	7.564375751	7.121742116
102723166	6.149285363	3.7077382	4.92851177	1.190059422	0.440372779	1.939746065
102723318	-0.352108471	-0.352108	-0.352108471	6.682822443	6.471635949	6.894008936
102723338	-0.352108471	-0.352108	-0.352108471	3.384482817	2.805970333	3.962995302
102723344	-0.352108471	-0.352108	-0.352108471	1.873849414	1.939746065	1.807952763
102723381	6.754635196	6.9079432	6.831289207	-0.352108471	-0.352108471	-0.352108471
102723382	-0.352108471	-0.352108	-0.352108471	4.950122184	3.919505936	2.888889687
102723393	7.245527112	7.1770317	7.108536371	2.5476814	1.046196536	1.796938968
102723395	-0.352108471	-0.352108	-0.352108471	7.050486941	5.596916991	4.143347041
102723412	-0.352108471	-0.352108	-0.352108471	4.292853281	6.569835415	8.846817549
102723423	5.876276997	5.9200251	5.832528937	3.80092067	4.482692559	3.119148782
102723426	3.280386814	4.2393435	5.198300279	-0.352108471	-0.352108471	-0.352108471
102723442	4.947007189	1.0914165	8.802597845	-0.352108471	-0.352108471	-0.352108471
102723446	3.279414369	3.5279165	3.776418535	6.378374162	6.935614535	5.821133789
102723449	6.836731419	7.6083864	8.38004137	5.380773408	7.996908999	6.688841204
102723490	4.503064341	-0.18326	2.15990226	4.872800634	6.846434104	5.859617369
102723506	5.173677083	2.7976612	7.549692921	4.996759282	-1.001604379	1.997577452
102723508	6.009358019	6.7354978	7.461637667	7.381481781	8.784840195	5.978123368
102723510	5.185586761	5.1799508	5.191222747	-0.352108471	-0.352108471	-0.352108471
102723521	7.798666424	6.8453896	5.892112784	5.774127426	3.625085775	7.923169078
102723529	-0.352108471	-0.352108	-0.352108471	2.810398723	1.438076269	2.124237496
102723543	5.26355452	6.1515856	7.039616737	-0.352108471	-0.352108471	-0.352108471
102723546	4.579618372	4.2452143	4.914022459	1.744484008	1.437859363	2.051108653
102723548	-0.352108471	-0.352108	-0.352108471	0.940233577	2.232575625	-0.352108471
102723549	6.537383725	6.8492425	6.225524967	-0.352108471	-0.352108471	-0.352108471
102723564	4.550307802	2.6980379	6.402577662	3.027377867	5.124543452	0.930212283
102723567	5.016270388	5.8529375	6.689604536	-0.352108471	-0.352108471	-0.352108471
102723578	-0.352108471	-0.352108	-0.352108471	3.73520442	5.017136436	6.299068451
102723582	4.335320611	5.4731418	4.904231184	-0.352108471	-0.352108471	-0.352108471
102723597	5.683431529	6.8018138	7.920196019	-0.352108471	-0.352108471	-0.352108471
102723603	11.11699173	6.7032984	8.910145059	7.345603024	7.821824499	8.298045973

102723623	5.110693474	6.2615598	5.686126657	3.916963084	1.210357624	6.623568543
102723656	5.931811387	5.7347435	5.537675535	1.345967809	1.14221151	1.549724107
102723678	4.677204034	7.5923972	6.134800615	-0.352108471	-0.352108471	-0.352108471
102723708	-0.352108471	-0.352108	-0.352108471	5.760537073	6.040166126	5.48090802
102723709	-0.352108471	-0.352108	-0.352108471	3.3765591	3.394991108	3.358127092
102723739	5.824288785	5.873346	5.775231583	-0.352108471	-0.352108471	-0.352108471
102723743	-0.352108471	-0.352108	-0.352108471	1.946552907	4.245214285	-0.352108471
102723750	7.43264761	7.1363903	7.728904916	2.745079935	6.139361673	9.533643411
102723769	6.66237111	6.8118473	6.512894899	6.099125059	6.345148158	5.853101961
102723780	4.772909641	4.9140225	4.631796822	-0.352108471	-0.352108471	-0.352108471
102723794	-0.352108471	-0.352108	-0.352108471	6.740064213	5.435224696	6.087644454
102723795	7.714431553	5.5926231	3.470814553	-0.352108471	-0.352108471	-0.352108471
102723802	-0.352108471	-0.352108	-0.352108471	5.095423988	5.124543452	5.066304524
102723832	4.792303669	4.8304259	4.754181416	-0.352108471	-0.352108471	-0.352108471
102723847	-0.352108471	-0.352108	-0.352108471	6.710896273	5.881191458	6.296043865
102723897	-0.352108471	-0.352108	-0.352108471	2.763418119	3.727833592	3.245625856
102723908	4.01645024	4.9470072	5.877564138	6.540502679	8.397076465	4.683928894
102723912	6.505122864	6.7549619	7.004801016	-0.352108471	-0.352108471	-0.352108471
102723968	4.117696434	3.51541	4.719982836	2.501526031	2.809020727	2.194031335
102723981	5.493783215	3.9239422	7.063624187	-0.352108471	-0.352108471	-0.352108471
102724023	-0.352108471	-0.352108	-0.352108471	9.7643434	2.707498922	6.235921161
102724030	6.689537938	7.0781647	6.300911206	4.65132759	5.775320426	5.213324008
102724036	-0.352108471	-0.352108	-0.352108471	1.167362396	2.686833264	-0.352108471
102724046	-0.352108471	-0.352108	-0.352108471	7.293225942	6.450302837	5.607379733
102724081	5.55876787	5.6506315	5.466904248	-0.352108471	-0.352108471	-0.352108471
102724094	-0.352108471	-0.352108	-0.352108471	4.265501071	0.785932822	2.525716947
102724148	4.843038887	5.054281	4.631796822	-0.352108471	-0.352108471	-0.352108471
102724190	-0.352108471	-0.352108	-0.352108471	2.024343053	1.689662294	2.359023812
102724211	6.253538778	3.8511786	8.655899005	1.569894481	1.997577452	1.14221151
102724220	-0.352108471	-0.352108	-0.352108471	2.02034096	2.100935855	1.939746065
102724225	-0.352108471	-0.352108	-0.352108471	3.722730865	6.46811711	5.095423988
102724229	-0.352108471	-0.352108	-0.352108471	6.640800069	5.810136601	6.225468335
102724235	2.681724436	0.8066625	4.556786355	-0.352108471	-0.352108471	-0.352108471
102724250	6.502263028	6.5668081	6.437717923	-0.352108471	-0.352108471	-0.352108471
102724252	4.895653759	8.452604	6.674128879	0.228373553	0.808855576	-0.352108471
102724273	-0.352108471	-0.352108	-0.352108471	5.637296679	3.692628542	7.581964815
102724292	-0.352108471	-0.352108	-0.352108471	6.246160577	6.215464554	6.276856601
102724339	-0.352108471	-0.352108	-0.352108471	6.039651413	9.610460608	2.468842218
102724344	6.77374078	6.7884205	6.759061079	-0.352108471	-0.352108471	-0.352108471
102724363	7.068980452	7.2865734	7.17777694	-0.352108471	-0.352108471	-0.352108471
102724380	-0.352108471	-0.352108	-0.352108471	4.313211605	3.085507837	3.699359721
102724423	-0.352108471	-0.352108	-0.352108471	0.874413692	-0.352108471	2.100935855
102724444	-0.352108471	-0.352108	-0.352108471	5.712263427	8.280447609	3.144079246
102724455	6.888648371	6.0908083	5.29296831	0.808855576	0.808855576	0.808855576
102724457	-0.352108471	-0.352108	-0.352108471	5.095423988	5.124543452	5.066304524
102724475	-0.352108471	-0.352108	-0.352108471	3.77417806	3.585121301	3.396064542
102724488	3.127737875	3.136327	3.119148782	6.165870606	6.174080069	6.157661143

102724507	-0.352108471	-0.352108	-0.352108471	6.266367194	6.276856601	6.255877787
102724532	6.541696368	5.8379872	5.134278016	8.30117402	5.205647422	6.753410721
102724594	5.77115932	7.9196199	6.845389604	-0.352108471	-0.352108471	-0.352108471
102724621	7.278768713	7.7991004	6.758436988	-0.352108471	-0.352108471	-0.352108471
102724634	5.152247113	5.7003494	4.604144794	-0.352108471	-0.352108471	-0.352108471
102724637	6.221877384	5.0931104	5.657493881	0.849500091	2.051108653	-0.352108471
102724646	-0.352108471	-0.352108	-0.352108471	5.204720742	2.508581108	7.900860376
102724657	2.803125457	5.9583594	-0.352108471	-0.352108471	-0.352108471	-0.352108471
102724659	5.249747311	6.3273021	5.78852472	1.852386493	0.63909494	1.245740716
102724668	8.502557848	5.4860477	6.994302779	-0.352108471	-0.352108471	-0.352108471
102724676	7.831268604	5.2736756	6.552472077	1.928436693	3.724381058	2.826408875
102724689	5.609572595	3.1036821	0.597791666	-0.352108471	-0.352108471	-0.352108471
102724699	10.62495013	10.906804	10.34309655	9.518627019	7.881578866	11.15567517
102724711	1.378672347	1.3835202	1.388367963	4.739675848	5.183235465	5.626795082
102724734	-0.352108471	-0.352108	-0.352108471	4.977710418	5.054280952	4.901139884
102724738	7.307826811	6.6235409	5.939255055	2.169424975	2.191310813	2.147539136
102724858	3.931850748	0.5464667	2.239158726	-0.352108471	-0.352108471	-0.352108471
102724859	-0.352108471	-0.352108	-0.352108471	4.08236011	4.245214285	3.919505936
102724872	-0.352108471	-0.352108	-0.352108471	7.007692314	6.305519736	5.603347158
102724877	6.238827807	6.4994854	6.369156591	-0.352108471	-0.352108471	-0.352108471
102724904	7.325892999	5.7743703	4.222847639	-0.352108471	-0.352108471	-0.352108471
102724927	7.739518189	7.1946085	6.649698759	3.844270002	5.118703215	6.393136428
102724938	5.831998311	6.0023087	5.661687951	-0.352108471	-0.352108471	-0.352108471
102724950	-0.352108471	-0.352108	-0.352108471	4.569567949	5.37748771	4.97352783
102724968	-0.352108471	-0.352108	-0.352108471	4.110067659	5.88700099	7.66393432
102724983	4.550307802	4.617923	4.482692559	6.131855599	6.383151496	5.880559702
102724984	3.340221245	6.2443861	4.792303669	-0.352108471	-0.352108471	-0.352108471
102724985	3.805664353	3.9195059	3.69182277	5.704335255	5.715557343	5.693113166
102725013	5.65589851	5.8319126	5.479884449	0.843352133	4.530314394	2.686833264
102725019	-0.352108471	-0.352108	-0.352108471	4.573795337	5.112282437	4.843038887
102725068	4.508443336	4.5740709	4.442815789	2.841536824	4.245214285	1.437859363
102725086	5.421457125	4.2150349	6.627879354	0.791292145	1.14221151	0.440372779
102725126	7.530950267	3.7840375	5.657493881	0.598807818	1.549724107	-0.352108471
102725186	4.918756329	9.2417413	7.080248828	8.07981804	7.149914465	7.614866253
102725195	-0.352108471	-0.352108	-0.352108471	2.076022254	2.100935855	2.051108653
102725210	6.533789593	5.1720853	5.852937462	1.245740716	2.051108653	0.440372779
102725247	-0.352108471	-0.352108	-0.352108471	4.73755266	3.712735384	5.762369936
102725509	2.065292044	-0.352108	4.482692559	2.313941724	-0.352108471	4.979991919
102800447	-0.352108471	-0.352108	-0.352108471	1.946552907	4.245214285	-0.352108471
103021294	6.661997756	6.3425283	6.502263028	-0.352108471	-0.352108471	-0.352108471
103106903	4.312701608	5.3910182	6.469334721	-0.352108471	-0.352108471	-0.352108471
103157000	5.105598647	5.1452963	5.065900975	3.793986574	0.800422977	6.78755017
103344929	4.617567339	6.4681519	8.318736396	0.044132154	0.440372779	-0.352108471
103344931	6.888173949	6.9567177	7.025261518	6.537165199	6.184463472	6.889866926
103352539	4.105225259	3.9268663	4.016045771	3.298817978	3.342978832	3.387139686
103352670	6.903030097	6.1401472	6.521588638	7.585865076	3.91931171	5.752588393
103611081	5.625160771	5.0798157	6.170505845	-0.352108471	-0.352108471	-0.352108471

103625681	5.04930029	5.9308246	6.812348884	4.666623264	1.939715935	3.3031696
103724386	4.467791151	4.3196312	4.615951059	-0.352108471	-0.352108471	-0.352108471
104054148	-0.352108471	-0.352108	-0.352108471	3.585121301	4.641495008	2.528747594
104169670	1.383520155	-0.352108	3.119148782	2.124237496	2.147539136	2.100935855
104266956	2.667523647	4.5870979	6.506672164	-0.352108471	-0.352108471	-0.352108471
104326051	-0.352108471	-0.352108	-0.352108471	0.874413692	2.100935855	-0.352108471
104355147	6.089329939	6.5468574	6.318093681	1.631676003	3.615460478	-0.352108471
104355149	6.047360751	5.9545903	5.861819855	1.117721484	1.305230684	0.930212283
104355287	5.845888183	4.453057	7.238719402	0.228373553	-0.352108471	0.808855576
104355295	4.297639083	4.6063879	3.98889025	2.753338652	0.440372779	5.066304524
104472714	6.753869352	7.9976037	9.241338116	5.632438617	6.802395966	4.462481268
104472716	8.194652846	10.051336	6.337969354	4.074835099	3.867799132	4.281871066
104472718	4.921054744	4.9167193	4.912383916	0.542875446	-0.352108471	1.437859363
104472848	2.132907287	4.617923	-0.352108471	6.217258872	6.276856601	6.157661143
104502416	4.217203809	5.3152588	3.119148782	1.293955958	2.147539136	0.440372779
104533120	-0.352108471	-0.352108	-0.352108471	3.119890661	1.167362396	-0.785165869
104548971	5.354685649	5.2241579	5.485213359	-0.352108471	-0.352108471	-0.352108471
104548972	2.016914154	-0.352108	4.385936778	6.128988904	6.735141444	7.341293985
104644205	7.743546215	8.5504282	6.936664241	8.046450095	7.493134535	6.939818975
104797538	5.393591769	7.079259	8.764926247	2.351086241	5.054280952	-0.352108471
105221694	4.19282617	6.0203811	7.847936012	4.501740506	6.819587433	2.18389358
105274304	-0.352108471	-0.352108	-0.352108471	5.017136436	3.392382825	6.641890046
105369140	6.083899313	5.7743703	5.464841325	-0.352108471	-0.352108471	-0.352108471
105369152	6.606671778	6.6538708	6.559472732	-0.352108471	-0.352108471	-0.352108471
105369153	5.454020722	6.6400194	7.826018063	-0.352108471	-0.352108471	-0.352108471
105369154	6.623540933	5.8725175	7.374564332	4.73755266	6.290023024	3.185082296
105369164	5.526823203	4.7479185	6.305727907	2.111907176	7.517878287	4.814892732
105369166	-0.352108471	-0.352108	-0.352108471	6.285554459	6.31523113	6.255877787
105369171	4.615143732	6.1675828	7.72002194	2.463480868	7.140688952	4.80208491
105369172	6.861057494	6.8767254	6.845389604	-0.352108471	-0.352108471	-0.352108471
105369175	4.947007189	4.9799919	4.914022459	-0.352108471	-0.352108471	-0.352108471
105369189	-0.352108471	-0.352108	-0.352108471	3.447208575	4.843308497	2.051108653
105369197	2.65478974	-0.759232	6.068811703	3.851666316	3.50082021	3.149974105
105369204	-0.352108471	-0.352108	-0.352108471	4.120822635	1.510772892	2.815797763
105369210	-0.352108471	-0.352108	-0.352108471	2.211943219	2.232575625	2.191310813
105369216	-0.352108471	-0.352108	-0.352108471	2.788781971	5.397214192	8.005646412
105369223	-0.352108471	-0.352108	-0.352108471	7.851917613	5.983736712	4.11555581
105369243	6.95059639	3.6190308	5.284813592	-0.352108471	-0.352108471	-0.352108471
105369244	5.359437143	7.1546555	6.257046343	1.598101998	1.646479888	1.549724107
105369271	2.376845906	2.5530733	2.729300687	3.919505936	1.164053417	6.674958454
105369276	4.510705466	5.4937832	6.476860964	3.189677368	5.075519558	6.961361749
105369282	4.925514366	4.7541814	4.582848465	-0.352108471	-0.352108471	-0.352108471
105369292	-0.352108471	-0.352108	-0.352108471	7.495200179	6.415928975	5.33665777
105369309	5.330618856	4.8482055	5.089412202	-0.352108471	-0.352108471	-0.352108471
105369314	-0.352108471	-0.352108	-0.352108471	4.186919329	2.339377742	6.034460915
105369327	-0.352108471	-0.352108	-0.352108471	5.279187466	5.663737322	6.048287177
105369328	-0.352108471	-0.352108	-0.352108471	4.00368964	3.572764679	3.141839719

105369344	6.183964016	6.1999612	6.167966808	-0.352108471	-0.352108471	-0.352108471
105369360	3.515141236	3.6761388	3.354143641	5.588224375	5.588224375	5.588224375
105369361	4.869177831	5.8001998	6.731221744	-0.352108471	-0.352108471	-0.352108471
105369364	3.716216518	2.5392592	3.127737875	0.897715333	2.147539136	-0.352108471
105369391	-0.352108471	-0.352108	-0.352108471	5.369332089	5.383503645	5.355160532
105369395	5.299765723	6.1515856	7.003405534	-0.352108471	-0.352108471	-0.352108471
105369419	5.800199788	6.6993531	4.901046517	-0.352108471	-0.352108471	-0.352108471
105369428	-0.352108471	-0.352108	-0.352108471	7.255774566	2.285024916	4.770399741
105369436	5.775231583	5.7752316	5.775231583	5.458255064	5.458255064	5.458255064
105369455	5.688622647	3.8706949	7.506550388	-0.352108471	-0.352108471	-0.352108471
105369486	5.420879354	5.7406872	5.101071532	-0.352108471	-0.352108471	-0.352108471
105369494	4.079415695	4.7439526	3.414878831	2.748587067	4.688318557	0.808855576
105369495	6.823036432	6.4570024	6.640019393	-0.352108471	-0.352108471	-0.352108471
105369498	-0.352108471	-0.352108	-0.352108471	6.266367194	6.276856601	6.255877787
105369516	-0.352108471	-0.352108	-0.352108471	6.783009163	5.575983727	4.36895829
105369526	-0.352108471	-0.352108	-0.352108471	6.211291876	2.90810652	4.559699198
105369546	4.553444075	5.9450387	5.249241373	-0.352108471	-0.352108471	-0.352108471
105369570	-0.352108471	-0.352108	-0.352108471	6.113115027	6.58346007	5.642769984
105369583	-0.352108471	-0.352108	-0.352108471	0.240492277	4.183394161	2.211943219
105369589	6.513563088	6.4002632	6.286963372	-0.352108471	-0.352108471	-0.352108471
105369591	-0.352108471	-0.352108	-0.352108471	4.953070076	2.686833264	0.420596451
105369605	6.369156591	5.0202775	7.718035661	-0.352108471	-0.352108471	-0.352108471
105369620	6.519673958	5.3533517	7.685996238	-0.352108471	-0.352108471	-0.352108471
105369626	-0.352108471	-0.352108	-0.352108471	5.23117587	5.315258837	5.147092904
105369632	8.082395191	5.7820025	3.481609878	1.429982115	0.256335003	2.603629227
105369633	-0.352108471	-0.352108	-0.352108471	5.712263427	5.731413688	5.693113166
105369662	-0.352108471	-0.352108	-0.352108471	3.23244152	5.681330879	4.456886199
105369671	-0.352108471	-0.352108	-0.352108471	6.885805372	3.452510279	5.169157826
105369687	-0.352108471	-0.352108	-0.352108471	6.369156591	5.689149611	7.04916357
105369699	-0.352108471	-0.352108	-0.352108471	5.745394463	7.795099455	3.695689471
105369703	5.20192326	5.7815511	6.361179011	3.973412303	6.937874907	1.008949699
105369718	-0.352108471	-0.352108	-0.352108471	4.12362865	3.615460478	4.631796822
105369727	5.876276997	5.5727089	6.179845051	-0.352108471	-0.352108471	-0.352108471
105369734	-0.352108471	-0.352108	-0.352108471	4.296218215	4.204267206	4.388169225
105369746	1.650498549	2.6817244	3.712950323	0.762376451	1.876861374	-0.352108471
105369750	-0.352108471	-0.352108	-0.352108471	5.233567424	5.254689875	5.212444972
105369765	5.906842547	5.385765	6.427920101	3.50103787	3.690969633	3.311106106
105369771	-0.352108471	-0.352108	-0.352108471	2.605817304	-0.352108471	5.563743079
105369796	1.456851574	2.3510862	3.245320908	0.044132154	-0.352108471	0.440372779
105369823	5.90029238	8.0046148	3.795969972	0.624614178	0.440372779	0.808855576
105369862	-0.352108471	-0.352108	-0.352108471	6.056053999	5.290590346	6.821517652
105369865	9.11648662	1.4592238	5.287855205	0.985062086	1.873707872	2.762353657
105369879	5.498944719	6.5527278	4.445161664	3.570927212	3.034828093	2.498728974
105369882	8.211514833	6.6905929	7.451053859	0.289051906	-0.352108471	0.930212283
105369884	7.08718283	6.4338331	7.740532544	-0.352108471	-0.352108471	-0.352108471
105369899	-0.352108471	-0.352108	-0.352108471	1.383520155	-0.352108471	3.119148782
105369900	6.561670144	7.2691536	7.976637098	-0.352108471	-0.352108471	-0.352108471

105369954	6.224862874	6.2558778	6.193847962	-0.352108471	-0.352108471	-0.352108471
105369963	1.946552907	-0.352108	4.245214285	2.072558294	1.997577452	2.147539136
105369966	-0.352108471	-0.352108	-0.352108471	5.233567424	5.254689875	5.212444972
105369981	7.211492803	3.3669907	5.28924173	-0.352108471	-0.352108471	-0.352108471
105370015	6.537383725	5.9402987	7.134468793	-0.352108471	-0.352108471	-0.352108471
105370033	6.573968999	6.9811491	6.166788899	-0.352108471	-0.352108471	-0.352108471
105370039	5.360782592	5.1245435	5.597021732	3.012289909	3.337746555	2.686833264
105370042	7.981521756	8.8469093	7.116134233	5.666392586	7.795117688	6.730755137
105370097	3.945254192	3.7021369	4.188371499	2.739781894	1.77365078	0.807519665
105370102	5.428393874	5.4582551	5.398532684	-0.352108471	-0.352108471	-0.352108471
105370109	5.345008441	6.8838848	6.114446624	5.567478026	5.366715977	5.768240076
105370110	5.900075821	3.4966392	4.698357509	-0.352108471	-0.352108471	-0.352108471
105370123	-0.352108471	-0.352108	-0.352108471	5.353955426	2.43016825	-0.493618925
105370141	-0.352108471	-0.352108	-0.352108471	6.12990004	5.138205193	7.121594888
105370182	-0.352108471	-0.352108	-0.352108471	5.547554998	1.622687605	3.585121301
105370205	1.383520155	-0.352108	3.119148782	2.826769934	-0.352108471	6.005648339
105370218	-0.352108471	-0.352108	-0.352108471	2.065292044	4.482692559	-0.352108471
105370230	2.553073296	3.819188	1.286958599	-0.352108471	-0.352108471	-0.352108471
105370241	5.284974356	3.7413293	6.828619428	-0.352108471	-0.352108471	-0.352108471
105370253	5.386535448	2.687836	8.085234854	2.685302075	1.688802714	0.692303353
105370294	5.625160771	5.6452218	5.605099774	-0.352108471	-0.352108471	-0.352108471
105370312	6.720370627	4.8469335	8.593807752	-0.352108471	-0.352108471	-0.352108471
105370324	5.284974356	5.3152588	5.254689875	-0.352108471	-0.352108471	-0.352108471
105370326	5.521606291	3.8643719	4.692989119	8.336642039	4.457094726	6.396868383
105370333	4.7505581	5.3865354	6.022512797	4.573434619	-0.59563739	1.988898614
105370360	5.354685649	5.3835036	5.325867653	-0.352108471	-0.352108471	-0.352108471
105370361	3.805664353	6.7638559	0.847472855	5.369332089	6.146249101	4.592415076
105370363	-0.352108471	-0.352108	-0.352108471	7.527394437	5.017136436	2.506878434
105370374	5.15068911	5.4788907	5.314789901	7.158526143	-0.423215372	3.367655385
105370378	5.939875572	5.3920614	4.844247146	-0.352108471	-0.352108471	-0.352108471
105370380	-0.352108471	-0.352108	-0.352108471	5.88700099	7.421106124	4.352895856
105370382	7.788898454	6.1165162	6.952707338	-0.352108471	-0.352108471	-0.352108471
105370389	6.44985595	5.7494021	5.048948278	2.766560492	5.424788855	8.083017217
105370408	-0.352108471	-0.352108	-0.352108471	2.633343959	3.119148782	2.147539136
105370409	-0.352108471	-0.352108	-0.352108471	5.169157826	5.191222747	5.147092904
105370475	3.683980801	4.6696054	2.698356187	2.103550565	2.673599798	3.24364903
105370546	7.122936951	4.528176	1.933415076	-0.352108471	-0.352108471	-0.352108471
105370548	8.364243563	6.5384988	4.712754107	-0.352108471	-0.352108471	-0.352108471
105370554	2.534485787	2.6224566	2.710427473	-0.352108471	-0.352108471	-0.352108471
105370596	5.749402114	5.7752316	5.723572646	-0.352108471	-0.352108471	-0.352108471
105370622	4.882738629	6.0736545	3.69182277	-0.352108471	-0.352108471	-0.352108471
105370624	5.126394779	5.6251608	6.123926763	-0.352108471	-0.352108471	-0.352108471
105370639	4.724330734	5.2849744	5.845617978	-0.352108471	-0.352108471	-0.352108471
105370671	5.625160771	7.1885684	4.06175311	-0.352108471	-0.352108471	-0.352108471
105370676	-0.352108471	-0.352108	-0.352108471	3.3942846	9.107785088	6.251034844
105370680	5.55876787	5.5882244	5.529311366	-0.352108471	-0.352108471	-0.352108471
105370687	4.038879774	2.1183088	5.959450749	3.448534433	2.615011019	3.031772726

105370697	2.681724436	0.6027528	4.760696067	-0.352108471	-0.352108471	-0.352108471
105370700	6.607377459	6.6215439	6.593211042	2.553073296	5.458255064	-0.352108471
105370703	2.740210233	-0.352108	5.832528937	-0.352108471	-0.352108471	-0.352108471
105370710	-0.352108471	-0.352108	-0.352108471	0.938538833	2.139844176	3.341149518
105370712	-0.352108471	-0.352108	-0.352108471	3.245625856	4.735168769	1.756082943
105370713	-0.352108471	-0.352108	-0.352108471	0.874413692	-0.352108471	2.100935855
105370720	2.446540028	2.5530733	2.659606565	-0.352108471	-0.352108471	-0.352108471
105370737	7.924465353	5.8529375	3.78140957	-0.352108471	-0.352108471	-0.352108471
105370748	2.486879591	-0.352108	5.325867653	-0.352108471	-0.352108471	-0.352108471
105370761	1.66201518	1.2718791	2.052151305	5.284974356	6.196741249	4.373207463
105370767	6.084059696	5.5587679	5.033476045	-0.352108471	-0.352108471	-0.352108471
105370772	5.939192221	5.9200251	5.958359385	-0.352108471	-0.352108471	-0.352108471
105370811	4.878665478	1.513975	8.24335596	-0.352108471	-0.352108471	-0.352108471
105370816	6.452765963	4.1300326	5.291399258	-0.352108471	-0.352108471	-0.352108471
105370836	-0.352108471	-0.352108	-0.352108471	4.410462234	5.575983727	6.741505219
105370839	5.354685649	3.678796	7.030575321	-0.352108471	-0.352108471	-0.352108471
105370847	3.416881325	5.4283939	7.439906423	-0.352108471	-0.352108471	-0.352108471
105370849	3.708209828	4.8786655	6.049121129	9.720359308	2.688953208	6.204656258
105370859	4.01477596	5.7757352	7.536694493	-0.352108471	-0.352108471	-0.352108471
105370872	5.152247113	6.2366294	4.067864783	4.927679215	3.740631438	6.114726992
105370899	-0.352108471	-0.352108	-0.352108471	1.446816307	4.178899946	6.910983584
105370900	-0.352108471	-0.352108	-0.352108471	2.926285844	2.656839111	2.387392378
105370928	3.684126867	8.5589307	6.121528769	-0.352108471	-0.352108471	-0.352108471
105370938	4.923818571	8.3592709	6.641544748	-0.352108471	-0.352108471	-0.352108471
105370949	-0.352108471	-0.352108	-0.352108471	1.631676003	3.615460478	-0.352108471
105370951	5.543977057	6.1079622	4.979991919	-0.352108471	-0.352108471	-0.352108471
105370958	6.396868383	7.5974815	5.196255244	-0.352108471	-0.352108471	-0.352108471
105371009	6.107769369	5.6251608	5.142552174	-0.352108471	-0.352108471	-0.352108471
105371011	-0.352108471	-0.352108	-0.352108471	7.330612862	5.171894159	6.251253511
105371017	-0.352108471	-0.352108	-0.352108471	5.052267685	5.124543452	4.979991919
105371022	6.857691475	4.5299463	9.185436682	4.881364164	7.293924745	6.087644454
105371028	-0.352108471	-0.352108	-0.352108471	5.760537073	4.408741859	7.112332286
105371052	-0.352108471	-0.352108	-0.352108471	3.919505936	3.919505936	3.919505936
105371060	5.714944975	5.7483543	5.781763599	4.851734675	4.245214285	5.458255064
105371063	3.906200071	5.76581	7.625419967	8.392081185	6.493532752	4.59498432
105371096	-0.352108471	-0.352108	-0.352108471	3.010220895	2.100935855	3.919505936
105371100	-0.352108471	-0.352108	-0.352108471	2.47165961	1.783698732	1.095737855
105371145	5.217491139	5.7771751	4.657807177	-0.352108471	-0.352108471	-0.352108471
105371146	3.38245853	6.3748724	4.878665478	6.982905827	3.207942148	5.095423988
105371156	3.255864286	2.3943359	2.8251001	-0.352108471	-0.352108471	-0.352108471
105371168	-0.352108471	-0.352108	-0.352108471	4.178899946	4.245214285	4.112585606
105371192	3.805664353	2.1763337	5.434995008	1.744735086	1.939746065	1.549724107
105371201	-0.352108471	-0.352108	-0.352108471	5.918181819	5.169157826	4.420133832
105371220	5.656289537	5.7155573	5.597021732	0.044132154	0.440372779	-0.352108471
105371227	5.941596889	4.0364862	7.846707608	-0.352108471	-0.352108471	-0.352108471
105371232	-0.352108471	-0.352108	-0.352108471	5.679743465	5.923493182	6.167242899
105371237	-0.352108471	-0.352108	-0.352108471	5.958359385	5.958359385	5.958359385

105371240	-0.352108471	-0.352108	-0.352108471	8.786760551	3.325347447	6.056053999
105371248	-0.352108471	-0.352108	-0.352108471	5.995240547	5.017136436	4.039032324
105371267	5.800199788	5.6817489	5.918650679	-0.352108471	-0.352108471	-0.352108471
105371297	7.506390201	7.1571706	7.331780412	7.056595924	7.008908203	7.032752064
105371305	5.556768734	4.7311114	3.905454011	0.362879868	3.677648519	2.020264194
105371320	6.480446832	5.3495577	7.611336002	1.509536442	1.876861374	1.14221151
105371330	5.749130652	5.4246336	5.586882134	-0.352108471	-0.352108471	-0.352108471
105371384	-0.352108471	-0.352108	-0.352108471	3.919505936	4.274024238	3.564987633
105371385	2.588601447	5.5293114	-0.352108471	-0.352108471	-0.352108471	-0.352108471
105371388	5.980334028	9.008187	2.952481073	-0.352108471	-0.352108471	-0.352108471
105371394	6.071788357	5.6249562	5.178123968	-0.352108471	-0.352108471	-0.352108471
105371400	-0.352108471	-0.352108	-0.352108471	5.013656289	5.324659362	5.169157826
105371401	3.749085012	6.940169	5.344627021	2.981195473	4.105704177	3.543449825
105371414	-0.352108471	-0.352108	-0.352108471	5.941434187	5.629960393	6.252907981
105371430	-0.352108471	-0.352108	-0.352108471	5.851193391	5.095423988	4.339654585
105371433	2.661898887	4.5220247	6.382150501	0.044132154	0.440372779	-0.352108471
105371451	5.89084209	7.7025584	4.07912574	6.459304058	6.00972091	6.234512484
105371455	-0.352108471	-0.352108	-0.352108471	2.553073296	-0.352108471	5.458255064
105371471	2.803125457	5.9583594	-0.352108471	-0.352108471	-0.352108471	-0.352108471
105371473	2.588601447	5.5293114	-0.352108471	2.630970183	3.615460478	1.646479888
105371476	5.980334028	5.8556276	6.105040413	-0.352108471	-0.352108471	-0.352108471
105371499	9.602057506	6.3324667	3.062875832	-0.352108471	-0.352108471	-0.352108471
105371510	4.095303961	5.2861808	2.904427076	-0.352108471	-0.352108471	-0.352108471
105371519	6.93466021	4.6173824	2.300104562	3.628626245	3.337746555	3.919505936
105371543	-0.352108471	-0.352108	-0.352108471	10.10584183	2.30347069	6.204656258
105371551	3.028785297	5.5557774	8.082769578	5.385284682	4.873169338	4.361053995
105371553	-0.352108471	-0.352108	-0.352108471	2.039485684	-0.352108471	4.43107984
105371554	-0.352108471	-0.352108	-0.352108471	3.682181533	4.245214285	3.119148782
105371556	-0.352108471	-0.352108	-0.352108471	6.202626522	4.927679215	3.652731908
105371565	5.392061359	3.7512273	7.032895429	-0.352108471	-0.352108471	-0.352108471
105371566	-0.352108471	-0.352108	-0.352108471	5.704335255	5.352550662	6.056119848
105371571	-0.352108471	-0.352108	-0.352108471	2.200078674	2.798770262	1.601387086
105371575	2.711561556	5.0747636	0.348359519	4.287786813	7.893491169	6.090638991
105371586	6.509913372	7.9438486	5.075978185	1.3309757	1.731739118	0.930212283
105371589	9.403855162	6.9675112	4.531167328	-0.352108471	-0.352108471	-0.352108471
105371595	4.867086667	4.9799919	4.754181416	-0.352108471	-0.352108471	-0.352108471
105371602	4.087257665	3.7993632	4.375152081	1.32702005	2.721666055	2.024343053
105371606	5.493783215	5.084979	5.902587387	-0.352108471	-0.352108471	-0.352108471
105371634	2.783958294	3.821987	1.745929632	-0.352108471	-0.352108471	-0.352108471
105371649	-0.352108471	-0.352108	-0.352108471	5.710172012	5.596916991	5.48366197
105371654	6.03798158	5.369033	6.7069302	6.529856559	5.821410838	5.112965116
105371676	-0.352108471	-0.352108	-0.352108471	6.897035646	6.061485107	6.479260377
105371692	5.895550985	5.5293114	6.261790604	6.9068292	7.146480757	6.667177643
105371695	-0.352108471	-0.352108	-0.352108471	6.266367194	6.276856601	6.255877787
105371743	3.580473277	8.2905092	5.935491224	4.645260436	4.020996898	3.39673336
105371748	6.152309613	5.5535653	5.852937462	-0.352108471	-0.352108471	-0.352108471
105371774	5.55876787	6.0780648	5.039470984	0.044132154	-0.352108471	0.440372779

105371783	-0.352108471	-0.352108	-0.352108471	6.396868383	6.410585269	6.383151496
105371787	2.201036472	0.1521352	4.249937703	-0.352108471	-0.352108471	-0.352108471
105371794	6.637460404	5.6657109	6.151585628	-0.352108471	-0.352108471	-0.352108471
105371804	7.258982997	8.1557272	6.362238773	6.938652084	6.056053999	5.173455914
105371828	6.703005628	7.3432278	7.983450005	4.337329384	4.490576065	4.413952725
105371838	4.939235113	4.6518549	4.364474699	0.95479973	2.612597735	1.783698732
105371839	6.396868383	5.2841754	7.509561332	4.66005769	3.805518094	5.514597285
105371850	4.621079993	4.9470072	5.272934386	-0.352108471	-0.352108471	-0.352108471
105371860	-0.352108471	-0.352108	-0.352108471	3.742557626	7.7482313	5.745394463
105371912	8.706411595	8.6820009	8.730822261	7.09730403	7.314125625	6.880482436
105371913	2.803125457	2.3941612	3.212089674	-0.352108471	-0.352108471	-0.352108471
105371919	5.958359385	3.6800839	8.236634836	-0.352108471	-0.352108471	-0.352108471
105371920	3.545346924	5.3381081	4.441727503	3.649440074	1.906323156	5.392556992
105371925	5.504664173	5.9941401	5.749402114	5.959308615	5.859617369	5.759926123
105371941	-0.352108471	-0.352108	-0.352108471	3.683183745	2.586774247	4.779593243
105371943	-0.352108471	-0.352108	-0.352108471	2.748265035	3.919505936	5.090746836
105371946	3.92027207	6.1140008	5.017136436	-0.352108471	-0.352108471	-0.352108471
105371984	6.537383725	7.4727585	5.602008994	-0.352108471	-0.352108471	-0.352108471
105371998	5.440881004	5.6890856	5.19267643	6.19018895	4.405759666	5.297974308
105372000	-0.352108471	-0.352108	-0.352108471	2.686833264	1.897647301	3.476019226
105372007	-0.352108471	-0.352108	-0.352108471	5.841772725	4.456886199	3.071999674
105372013	7.160036637	5.7532474	4.346458241	-0.352108471	-0.352108471	-0.352108471
105372019	-0.352108471	-0.352108	-0.352108471	3.897396099	7.745425577	5.821410838
105372056	-0.352108471	-0.352108	-0.352108471	6.12990004	6.883222067	5.376578013
105372092	-0.352108471	-0.352108	-0.352108471	7.478388974	1.435383425	4.456886199
105372109	7.623320807	4.1558905	5.889605647	3.337001686	4.482692559	2.191310813
105372115	-0.476070528	5.103954	2.313941724	-0.352108471	-0.352108471	-0.352108471
105372126	6.242048279	6.2558778	6.228218771	0.044132154	-0.352108471	0.440372779
105372143	-0.352108471	-0.352108	-0.352108471	5.169157826	5.750484729	4.587830922
105372160	2.553073296	1.4056176	3.700528987	-0.352108471	-0.352108471	-0.352108471
105372176	5.393115701	6.9219153	3.864316134	-0.352108471	-0.352108471	-0.352108471
105372190	-0.352108471	-0.352108	-0.352108471	5.233567424	6.458152413	4.008982434
105372195	4.560468605	4.9470072	5.333545773	-0.352108471	-0.352108471	-0.352108471
105372197	3.212513362	7.9050224	5.55876787	-0.352108471	-0.352108471	-0.352108471
105372201	5.089412202	5.1245435	5.054280952	-0.352108471	-0.352108471	-0.352108471
105372202	-0.352108471	-0.352108	-0.352108471	3.706243326	6.328029545	5.017136436
105372209	5.90029238	5.9200251	5.880559702	-0.352108471	-0.352108471	-0.352108471
105372218	3.805664353	6.1925657	1.418763015	2.185115095	3.245625856	4.306136617
105372231	-0.352108471	-0.352108	-0.352108471	5.258845866	6.31523113	7.371616395
105372263	7.979998603	6.5373837	5.094768846	8.772235414	6.479260377	4.186285339
105372269	6.059993141	6.1999612	5.920025058	0.044132154	-0.352108471	0.440372779
105372272	-0.352108471	-0.352108	-0.352108471	2.765800232	3.77959546	4.793390687
105372275	2.351086241	0.6479807	4.05419183	3.670176582	5.804928738	4.73755266
105372277	7.413456664	4.0256733	5.719564994	3.85262404	1.206270807	2.529447424
105372294	5.607291125	5.8001998	5.99310845	-0.352108471	-0.352108471	-0.352108471
105372300	-0.352108471	-0.352108	-0.352108471	2.525716947	-0.352108471	5.403542364
105372305	-0.352108471	-0.352108	-0.352108471	6.124301981	5.893442277	6.355161685

105372306	4.248783913	6.9338273	5.591305588	1.223721097	1.305230684	1.14221151
105372310	7.0366128	7.352529	6.720696571	-0.352108471	-0.352108471	-0.352108471
105372315	8.122667381	6.9398718	5.757076216	-0.352108471	-0.352108471	-0.352108471
105372318	-0.352108471	-0.352108	-0.352108471	6.676169033	6.698467242	6.653870824
105372325	6.910176716	6.816905	6.723633369	1.968661759	1.997577452	1.939746065
105372327	-0.352108471	-0.352108	-0.352108471	3.259218329	4.539984809	1.978451848
105372331	5.939010806	6.6235409	7.30807106	-0.352108471	-0.352108471	-0.352108471
105372337	-0.352108471	-0.352108	-0.352108471	3.086062074	3.919505936	2.252618212
105372372	-0.352108471	-0.352108	-0.352108471	6.434644758	5.327736786	7.54155273
105372386	5.484614182	2.7762562	4.13043521	4.590089232	4.518591297	4.554340264
105372387	6.757030794	4.4339685	5.595499658	4.300487042	7.826417261	6.063452152
105372389	6.007374565	8.1050157	7.05619511	6.423769798	6.689878453	6.157661143
105372401	-0.352108471	-0.352108	-0.352108471	5.69326252	6.174080069	5.212444972
105372416	-0.352108471	-0.352108	-0.352108471	5.150781781	8.187775524	2.113788037
105372436	6.123913494	6.8251815	7.526449601	6.56915909	5.648630776	7.489687404
105372448	4.792303669	4.8304259	4.754181416	-0.352108471	-0.352108471	-0.352108471
105372453	2.685732087	1.2722316	4.099232606	-0.352108471	-0.352108471	-0.352108471
105372474	5.277506698	5.463922	5.650337352	0.86953393	0.808855576	0.930212283
105372479	1.173067317	1.5010176	1.828967852	2.036185252	1.315841796	0.59549834
105372480	-0.352108471	-0.352108	-0.352108471	7.145752419	6.235921161	5.326089903
105372481	6.657100227	6.0078331	6.332466669	-0.352108471	-0.352108471	-0.352108471
105372487	-0.352108471	-0.352108	-0.352108471	1.968661759	1.997577452	1.939746065
105372488	-0.161866399	5.0644478	2.451290702	3.400229289	2.82785899	2.255488691
105372489	-0.352108471	-0.352108	-0.352108471	6.070043966	6.101634422	6.038453511
105372490	3.754972794	6.0023582	4.878665478	-0.352108471	-0.352108471	-0.352108471
105372500	-0.352108471	-0.352108	-0.352108471	3.211896338	8.367629114	5.789762726
105372553	7.779049226	6.183964	4.588878805	-0.352108471	-0.352108471	-0.352108471
105372564	-0.352108471	-0.352108	-0.352108471	7.294486055	5.923493182	4.552500309
105372569	-0.352108471	-0.352108	-0.352108471	5.821410838	5.825167992	5.817653684
105372580	6.463513944	6.1679668	6.759061079	2.858198167	3.615460478	2.100935855
105372583	-0.352108471	-0.352108	-0.352108471	2.127314251	3.119148782	4.110983312
105372584	7.914061853	7.11166	6.309258102	1.308941151	6.165346909	3.73714403
105372585	5.625160771	5.1687474	6.081574139	-0.352108471	-0.352108471	-0.352108471
105372590	-0.352108471	-0.352108	-0.352108471	5.904005051	7.04134783	6.47267644
105372603	-0.352108471	-0.352108	-0.352108471	5.575983727	6.137731367	5.014236086
105372607	6.081742358	7.6121718	6.846957097	2.33707729	0.021502393	1.179289842
105372630	-0.352108471	-0.352108	-0.352108471	6.345275458	5.982103371	6.708447544
105372647	5.937827329	6.2083007	5.667353966	6.750286666	6.038179856	6.394233261
105372657	-0.352108471	-0.352108	-0.352108471	6.194352735	6.802965135	7.411577535
105372659	5.952184013	6.8415528	6.396868383	0.940233577	2.232575625	-0.352108471
105372669	-0.352108471	-0.352108	-0.352108471	1.167362396	2.686833264	-0.352108471
105372683	-0.352108471	-0.352108	-0.352108471	5.116484326	7.29282819	6.204656258
105372699	4.000751846	5.7336454	7.466538923	-0.352108471	-0.352108471	-0.352108471
105372700	-0.352108471	-0.352108	-0.352108471	4.456886199	4.60565593	4.308116469
105372703	4.927238735	4.9667756	4.947007189	-0.352108471	-0.352108471	-0.352108471
105372705	5.74446732	4.5406293	5.142548317	-0.352108471	-0.352108471	-0.352108471
105372706	2.553073296	-0.352108	5.458255064	-0.352108471	-0.352108471	-0.352108471

105372708	6.241862871	5.5553643	5.898613566	6.439574147	3.818058994	1.196543842
105372716	6.414258136	6.4453648	6.383151496	-0.352108471	-0.352108471	-0.352108471
105372722	-0.352108471	-0.352108	-0.352108471	3.585121301	2.861039218	4.309203384
105372729	10.99664675	9.7527641	8.508881449	8.737511829	5.503380275	7.120446052
105372731	-0.352108471	-0.352108	-0.352108471	3.847979261	0.947005172	2.397492216
105372733	7.251757684	7.2622793	7.241236027	6.47267644	6.497336045	6.448016836
105372734	-0.352108471	-0.352108	-0.352108471	6.266367194	8.836261014	3.696473373
105372759	5.381666778	4.2755511	6.487782447	-0.352108471	-0.352108471	-0.352108471
105372760	5.184151491	5.2437595	5.124543452	-0.352108471	-0.352108471	-0.352108471
105372764	-0.352108471	-0.352108	-0.352108471	2.881499807	3.615460478	2.147539136
105372790	4.372287533	5.0171364	5.661985339	-0.352108471	-0.352108471	-0.352108471
105372794	4.194550202	5.0522677	5.909985168	-0.352108471	-0.352108471	-0.352108471
105372802	-0.352108471	-0.352108	-0.352108471	2.430691558	5.073786662	3.75223911
105372808	-0.352108471	-0.352108	-0.352108471	4.120814639	5.734543791	4.927679215
105372815	6.495254774	6.056054	5.616853224	-0.352108471	-0.352108471	-0.352108471
105372837	6.349569396	7.5408057	5.158333044	0.845664482	2.85116063	4.856656779
105372843	5.770788164	5.8001998	5.829611411	-0.352108471	-0.352108471	-0.352108471
105372844	5.187052848	5.3965395	5.291796159	1.842407068	1.876861374	1.807952763
105372855	6.38339657	5.8242888	5.265181	-0.352108471	-0.352108471	-0.352108471
105372857	-0.352108471	-0.352108	-0.352108471	4.11944322	3.337746555	4.901139884
105372858	4.400341142	3.6447708	5.155911468	3.607229804	6.888507299	5.247868551
105372866	5.758523395	5.2641585	6.252888256	-0.352108471	-0.352108471	-0.352108471
105372870	6.265560412	6.0703732	6.167966808	-0.352108471	-0.352108471	-0.352108471
105372872	3.527916452	4.3462168	2.709616062	1.825906298	3.539517737	5.253129175
105372877	4.792303669	4.8304259	4.754181416	-0.352108471	-0.352108471	-0.352108471
105372884	-0.352108471	-0.352108	-0.352108471	6.735779686	6.750862801	6.720696571
105372888	-0.352108471	-0.352108	-0.352108471	6.056053999	6.073654487	6.038453511
105372909	4.511889403	4.3368437	4.161797949	2.893678875	2.232575625	3.554782125
105372939	6.297476393	6.7070497	6.502263028	1.411051924	8.623220948	5.017136436
105372952	7.77831314	5.5657607	6.672036922	5.816428737	9.821148092	7.818788415
105372959	4.792303669	4.8304259	4.754181416	-0.352108471	-0.352108471	-0.352108471
105372960	7.215218797	4.7015	5.958359385	2.458228148	1.043426334	-0.37137548
105372965	6.808340183	5.3961231	8.22055723	5.608521445	3.004191963	8.212850927
105372978	-0.352108471	-0.352108	-0.352108471	1.789985844	2.301055037	2.812124231
105373005	6.455749259	5.5698028	7.341695675	5.350030384	4.239428057	4.79472922
105373025	-0.352108471	-0.352108	-0.352108471	2.212189733	3.427687301	0.996692165
105373060	2.659360865	2.4868796	2.314398317	-0.352108471	-0.352108471	-0.352108471
105373064	2.351086241	5.054281	-0.352108471	-0.352108471	-0.352108471	-0.352108471
105373072	-0.352108471	-0.352108	-0.352108471	5.477983694	4.071013389	4.774498542
105373085	5.073466497	6.6400194	8.206572288	3.276328907	2.22936792	1.182406934
105373092	6.64651405	6.6191582	6.67386988	6.635453109	9.069520854	4.201385365
105373097	5.688622647	7.9377564	3.439488919	-0.352108471	-0.352108471	-0.352108471
105373107	6.2094349	7.2011401	6.705287512	-0.352108471	-0.352108471	-0.352108471
105373116	-0.352108471	-0.352108	-0.352108471	0.077277834	1.891423885	3.705569937
105373132	-0.352108471	-0.352108	-0.352108471	2.124237496	0.949252571	3.299222421
105373154	7.727652175	6.9134889	6.099325622	-0.352108471	-0.352108471	-0.352108471
105373159	-0.352108471	-0.352108	-0.352108471	6.163080188	6.235352281	6.307624374

105373172	4.51172187	4.9735278	5.435333789	-0.352108471	-0.352108471	-0.352108471
105373195	5.132275089	6.1368079	7.141340621	3.073599906	1.744484008	0.41536811
105373203	3.505168771	1.1970037	2.351086241	-0.352108471	-0.352108471	-0.352108471
105373244	5.876276997	5.982996	5.769557961	-0.352108471	-0.352108471	-0.352108471
105373247	2.451247688	2.4512907	2.451333716	-0.352108471	-0.352108471	-0.352108471
105373249	-0.352108471	-0.352108	-0.352108471	5.609138735	3.995031086	4.80208491
105373254	5.626442006	7.522329	3.730555035	-0.352108471	-0.352108471	-0.352108471
105373263	-0.352108471	-0.352108	-0.352108471	5.089169987	4.456886199	3.824602411
105373273	-0.352108471	-0.352108	-0.352108471	4.088118542	4.314079953	4.201099247
105373289	-0.352108471	-0.352108	-0.352108471	6.660779387	5.670961825	6.165870606
105373292	-0.352108471	-0.352108	-0.352108471	3.684880912	3.245625856	2.8063708
105373297	-0.352108471	-0.352108	-0.352108471	3.990917638	6.579031074	5.284974356
105373306	6.878577877	5.5207125	4.16284712	-0.352108471	-0.352108471	-0.352108471
105373316	8.340617406	11.831005	10.08581113	9.883770033	6.34922329	8.116496661
105373318	6.12250122	4.0563232	5.089412202	-0.352108471	-0.352108471	-0.352108471
105373329	6.778521655	6.3657707	5.953019844	-0.352108471	-0.352108471	-0.352108471
105373356	-0.352108471	-0.352108	-0.352108471	6.374965413	6.165870606	5.956775799
105373362	-0.352108471	-0.352108	-0.352108471	5.369332089	5.383503645	5.355160532
105373365	7.11396904	8.0292243	6.198713791	2.166656576	1.646479888	2.686833264
105373369	6.353573297	6.2858739	6.421272681	-0.352108471	-0.352108471	-0.352108471
105373377	2.313941724	4.9799919	-0.352108471	-0.352108471	-0.352108471	-0.352108471
105373378	4.6689304	6.377549	5.523239719	-0.352108471	-0.352108471	-0.352108471
105373414	-0.352108471	-0.352108	-0.352108471	3.245625856	3.337746555	3.153505157
105373424	6.155981145	5.1398055	4.12362985	-0.352108471	-0.352108471	-0.352108471
105373425	5.688622647	6.4261829	4.951062387	-0.352108471	-0.352108471	-0.352108471
105373457	1.392109249	3.136327	-0.352108471	2.685883497	3.119148782	2.252618212
105373476	5.353790381	4.126404	6.581176774	-0.352108471	-0.352108471	-0.352108471
105373479	2.407299168	4.8786655	7.350031788	-0.352108471	-0.352108471	-0.352108471
105373487	-0.352108471	-0.352108	-0.352108471	5.723485516	5.731413688	5.715557343
105373488	2.618057952	5.5882244	-0.352108471	-0.352108471	-0.352108471	-0.352108471
105373490	5.852937462	6.7867168	4.919158158	-0.352108471	-0.352108471	-0.352108471
105373495	-0.352108471	-0.352108	-0.352108471	2.451290702	5.254689875	-0.352108471
105373498	5.709671152	4.5948231	5.152247113	-0.352108471	-0.352108471	-0.352108471
105373537	-0.352108471	-0.352108	-0.352108471	5.859617369	5.873345987	5.845888751
105373557	6.224581073	5.5420741	5.88332757	-0.352108471	-0.352108471	-0.352108471
105373583	-0.352108471	-0.352108	-0.352108471	5.501776916	4.76985944	5.135818178
105373594	2.588601447	-0.352108	5.529311366	-0.352108471	-0.352108471	-0.352108471
105373604	4.539878369	5.3537904	6.167702393	-0.352108471	-0.352108471	-0.352108471
105373606	-0.352108471	-0.352108	-0.352108471	7.302158403	2.717145811	5.009652107
105373622	-0.352108471	-0.352108	-0.352108471	6.874660259	6.946414218	6.802906299
105373651	2.38621749	6.5803415	-1.807906506	-0.352108471	-0.352108471	-0.352108471
105373673	1.392109249	-0.723524	3.507742701	1.832717481	1.852096656	1.842407068
105373674	6.724469766	5.8452988	7.60364074	-0.352108471	-0.352108471	-0.352108471
105373681	5.449551011	6.183964	6.918377021	-0.352108471	-0.352108471	-0.352108471
105373714	5.55876787	6.3740208	4.743514957	-0.352108471	-0.352108471	-0.352108471
105373726	-0.352108471	-0.352108	-0.352108471	6.020506301	6.056053999	6.091601696
105373738	7.196698817	7.352529	7.040868605	6.658968677	6.515031054	6.802906299

105373761	-0.352108471	-0.352108	-0.352108471	5.284974356	6.573338478	3.996610234
105373774	5.350056358	6.056054	6.76205164	5.742889092	6.087644454	6.432399817
105373810	2.515697587	5.3835036	-0.352108471	-0.352108471	-0.352108471	-0.352108471
105373816	2.486879591	2.4619273	2.511831882	-0.352108471	-0.352108471	-0.352108471
105373824	7.972954947	6.502263	5.031571109	-0.352108471	-0.352108471	-0.352108471
105373844	-0.352108471	-0.352108	-0.352108471	6.296043865	5.085644905	7.506442826
105373874	-0.352108471	-0.352108	-0.352108471	4.744220006	5.169157826	5.594095646
105373895	-0.352108471	-0.352108	-0.352108471	-0.366252974	2.605817304	5.577887581
105373904	-0.352108471	-0.352108	-0.352108471	4.871635595	4.178899946	3.486164297
105373916	5.42626078	3.3070879	4.36667435	0.228373553	-0.352108471	0.808855576
105373926	6.816905042	6.7884205	6.845389604	3.012289909	3.337746555	2.686833264
105373943	5.080584181	6.4487689	3.712399503	5.056085501	3.886404517	2.716723534
105373950	-0.352108471	-0.352108	-0.352108471	5.889433849	5.859617369	5.829800889
105373954	-0.352108471	-0.352108	-0.352108471	6.545950789	6.020474238	7.071427339
105373956	5.88206096	6.2125362	6.543011457	-0.352108471	-0.352108471	-0.352108471
105373958	5.375612014	5.7494021	6.123192214	-0.352108471	-0.352108471	-0.352108471
105373959	-0.352108471	-0.352108	-0.352108471	2.024343053	2.051108653	1.997577452
105373968	-0.352108471	-0.352108	-0.352108471	5.923493182	4.877154119	6.969832246
105373981	-0.352108471	-0.352108	-0.352108471	2.419557138	5.191222747	-0.352108471
105374013	5.562159617	3.3537661	7.770553139	5.321820691	4.199045311	3.076269931
105374047	7.149852634	4.5057558	5.827804241	2.207172287	1.598101998	0.989031709
105374066	4.716750566	3.345325	4.031037805	1.783698732	2.315101139	1.252296326
105374073	6.656125064	6.6694769	6.642773206	-0.352108471	-0.352108471	-0.352108471
105374084	5.469175652	5.9583594	4.979991919	6.041477508	5.958359385	6.124595632
105374089	6.45166524	5.1522471	3.852828986	-0.352108471	-0.352108471	-0.352108471
105374104	6.853089993	6.6333781	7.072801884	4.648570438	5.709190472	5.178880455
105374105	4.736544651	6.004054	7.271563325	-0.352108471	-0.352108471	-0.352108471
105374112	-0.352108471	-0.352108	-0.352108471	5.017136436	5.054280952	4.979991919
105374114	6.414258136	8.6260328	4.2024835	-0.352108471	-0.352108471	-0.352108471
105374117	7.795211269	3.9106637	5.852937462	-0.352108471	-0.352108471	-0.352108471
105374125	5.552021843	6.0483777	5.800199788	0.044132154	0.440372779	-0.352108471
105374127	-0.352108471	-0.352108	-0.352108471	6.463674915	5.228894993	7.698454837
105374129	-0.352108471	-0.352108	-0.352108471	5.859617369	5.587617059	6.131617679
105374158	6.058072194	7.7728008	6.915436509	-0.352108471	-0.352108471	-0.352108471
105374166	4.720108865	3.1133419	6.326875794	0.542875446	1.437859363	-0.352108471
105374181	8.285755715	4.3046829	6.295219325	4.599436265	4.435020481	4.270604696
105374187	-0.352108471	-0.352108	-0.352108471	2.076022254	2.100935855	2.051108653
105374226	0.308208442	2.6505957	4.992982898	-0.352108471	-0.352108471	-0.352108471
105374239	-0.352108471	-0.352108	-0.352108471	4.80208491	6.005916655	3.598253165
105374247	-0.352108471	-0.352108	-0.352108471	2.854007231	7.321798494	5.087902863
105374267	-0.352108471	-0.352108	-0.352108471	1.590060026	2.024343053	2.458626079
105374298	5.428393874	5.4582551	5.398532684	-0.352108471	-0.352108471	-0.352108471
105374301	5.019282955	2.9706358	7.067930135	7.346440078	2.506219572	4.926329825
105374303	-0.352108471	-0.352108	-0.352108471	5.325716078	5.084610622	5.566821534
105374304	5.995385515	5.8829989	5.939192221	-0.352108471	-0.352108471	-0.352108471
105374335	-0.276880308	4.1699861	1.946552907	4.878170137	4.545205492	5.211134782
105374338	5.925007489	6.5117733	5.338241658	6.809477782	5.704935534	4.600393286

105374339	8.44419954	5.5587679	2.6733362	-0.352108471	-0.352108471	-0.352108471
105374340	5.249224703	7.8760826	2.622366844	-0.352108471	-0.352108471	-0.352108471
105374349	2.62245663	3.4586652	1.786248034	-0.352108471	-0.352108471	-0.352108471
105374350	5.354536482	5.8887701	6.423003772	4.83789099	5.688622647	6.539354304
105374357	6.331393632	7.2863246	8.241255661	-0.352108471	-0.352108471	-0.352108471
105374358	-0.352108471	-0.352108	-0.352108471	7.872707178	4.785420335	1.698133492
105374363	10.32122726	8.6310674	6.940907504	11.33167175	7.945175188	4.558678625
105374414	5.532069877	5.1771923	5.35463107	-0.352108471	-0.352108471	-0.352108471
105374421	-0.352108471	-0.352108	-0.352108471	5.249134474	6.32531018	7.401485887
105374426	-0.352108471	-0.352108	-0.352108471	4.532283971	3.82551592	4.178899946
105374439	6.295386969	4.8267197	7.764054204	-0.352108471	-0.352108471	-0.352108471
105374462	-0.352108471	-0.352108	-0.352108471	6.454311268	0.715931335	3.585121301
105374477	-0.352108471	-0.352108	-0.352108471	4.98893646	5.169157826	5.349379192
105374494	4.963168291	5.8162788	5.389723561	-0.352108471	-0.352108471	-0.352108471
105374507	2.44582553	5.2437595	-0.352108471	-0.352108471	-0.352108471	-0.352108471
105374509	-0.352108471	-0.352108	-0.352108471	2.626750568	5.605609608	-0.352108471
105374533	-0.352108471	-0.352108	-0.352108471	2.881499807	3.615460478	2.147539136
105374558	3.916593774	7.6314925	5.77404314	4.820948486	7.853436899	1.788460073
105374582	-0.352108471	-0.352108	-0.352108471	1.601336827	2.486284199	0.716389454
105374585	-0.352108471	-0.352108	-0.352108471	2.211943219	2.232575625	2.191310813
105374587	4.901506784	5.2437595	4.559254036	5.962630825	5.731413688	6.193847962
105374602	5.722600146	5.5587679	5.394935595	5.409753312	6.730334621	6.070043966
105374637	3.851786382	7.0760577	5.463922025	-0.352108471	-0.352108471	-0.352108471
105374650	-0.352108471	-0.352108	-0.352108471	3.245625856	3.337746555	3.153505157
105374708	2.588601447	-0.352108	5.529311366	-0.352108471	-0.352108471	-0.352108471
105374712	-0.352108471	-0.352108	-0.352108471	2.787426417	-1.244678345	6.81953118
105374713	-0.352108471	-0.352108	-0.352108471	-0.007541461	2.274515706	4.556572874
105374767	-0.352108471	-0.352108	-0.352108471	2.40503459	2.886513546	2.645774068
105374771	6.412437211	6.4277085	6.420072833	0.623678479	1.540978788	2.458279096
105374775	-0.352108471	-0.352108	-0.352108471	3.859631539	8.934105226	6.396868383
105374778	-0.352108471	-0.352108	-0.352108471	6.089675752	3.585121301	1.08056685
105374790	-0.352108471	-0.352108	-0.352108471	4.569831023	4.178899946	3.787968868
105374795	6.860920727	3.5740616	5.217491139	-0.352108471	-0.352108471	-0.352108471
105374799	-0.352108471	-0.352108	-0.352108471	5.821410838	6.470665102	5.172156573
105374800	7.666631757	4.133953	5.90029238	-0.352108471	-0.352108471	-0.352108471
105374812	3.919505936	3.9195059	3.919505936	0.793818797	-0.352108471	1.939746065
105374835	-0.352108471	-0.352108	-0.352108471	5.233567424	6.902108188	3.565026659
105374838	-0.352108471	-0.352108	-0.352108471	7.316768464	5.369332089	3.421895714
105374858	6.395996971	6.3439771	6.448016836	-0.352108471	-0.352108471	-0.352108471
105374866	5.291399258	6.9857424	3.597056073	-0.352108471	-0.352108471	-0.352108471
105374871	-0.352108471	-0.352108	-0.352108471	5.789762726	5.629207848	5.950317604
105374872	-0.352108471	-0.352108	-0.352108471	5.972621945	5.821410838	5.670199731
105374879	6.242048279	8.4159232	4.068173401	-0.352108471	-0.352108471	-0.352108471
105374892	1.6561563	2.9239264	4.191696452	-0.352108471	-0.352108471	-0.352108471
105374932	1.985287245	5.0894122	8.193537159	-0.352108471	-0.352108471	-0.352108471
105374970	3.228477782	5.3920006	7.555523459	-0.352108471	-0.352108471	-0.352108471
105374983	4.754181416	6.5199701	2.988392724	-0.352108471	-0.352108471	-0.352108471

105374984	7.217668718	4.2811355	5.749402114	-0.352108471	-0.352108471	-0.352108471
105374986	6.914839026	6.1675828	5.420326646	-0.352108471	-0.352108471	-0.352108471
105374988	9.303741924	7.5281072	5.752472553	0.440372779	0.440372779	0.440372779
105374989	5.054280952	4.3557095	5.752852389	1.761670631	1.646479888	1.876861374
105375021	-0.352108471	-0.352108	-0.352108471	4.80208491	4.843308497	4.760861323
105375032	5.625160771	6.7628703	4.487451286	-0.352108471	-0.352108471	-0.352108471
105375043	5.640043677	6.8233885	8.006733384	7.115785866	3.332849856	5.224317861
105375054	5.029645164	5.7945685	4.264721793	2.19321233	0.348096305	1.270654317
105375056	-0.352108471	-0.352108	-0.352108471	3.119148782	5.106236715	1.132060848
105375063	5.638684911	6.295387	6.952089027	0.624614178	0.440372779	0.808855576
105375064	8.7701777	3.6548947	6.212536209	-0.352108471	-0.352108471	-0.352108471
105375085	-0.352108471	-0.352108	-0.352108471	6.12990004	6.135204448	6.124595632
105375087	-0.352108471	-0.352108	-0.352108471	3.558956552	2.686833264	4.43107984
105375099	-0.352108471	-0.352108	-0.352108471	2.351086241	5.054280952	-0.352108471
105375122	-0.352108471	-0.352108	-0.352108471	3.808273748	5.369332089	6.930390429
105375128	2.62245663	-0.352108	5.597021732	-0.352108471	-0.352108471	-0.352108471
105375132	-0.352108471	-0.352108	-0.352108471	2.98156133	6.31523113	-0.352108471
105375135	-0.352108471	-0.352108	-0.352108471	6.194496374	5.772977049	5.983736712
105375137	-0.352108471	-0.352108	-0.352108471	5.931958596	5.933157898	5.9343572
105375170	5.410363371	-0.174247	2.618057952	-0.352108471	-0.352108471	-0.352108471
105375213	-0.352108471	-0.352108	-0.352108471	5.369332089	5.383503645	5.355160532
105375217	4.163774858	2.5002886	5.827261103	-0.352108471	-0.352108471	-0.352108471
105375229	-0.352108471	-0.352108	-0.352108471	3.919505936	3.919505936	3.919505936
105375249	-0.352108471	-0.352108	-0.352108471	6.296043865	6.31523113	6.276856601
105375256	5.800199788	5.825168	5.775231583	-0.352108471	-0.352108471	-0.352108471
105375275	-0.352108471	-0.352108	-0.352108471	5.494417177	5.107178892	5.881655462
105375282	6.257046343	6.0921498	6.421942902	-0.352108471	-0.352108471	-0.352108471
105375291	-0.352108471	-0.352108	-0.352108471	2.515697587	5.383503645	-0.352108471
105375301	5.754124716	5.5882244	5.920025058	5.588224375	5.588224375	5.588224375
105375310	-0.352108471	-0.352108	-0.352108471	5.412561087	3.961798016	6.863324159
105375320	-0.352108471	-0.352108	-0.352108471	5.697771913	6.450302837	7.202833762
105375328	8.142000074	6.413437	4.684873917	-0.352108471	-0.352108471	-0.352108471
105375341	5.019618295	8.4654695	1.573767134	2.942215415	1.08477577	4.799655059
105375383	-0.352108471	-0.352108	-0.352108471	8.275958535	5.663737322	3.051516109
105375384	-0.352108471	-0.352108	-0.352108471	2.536084801	0.440372779	4.631796822
105375388	4.64626819	3.0491097	6.243426649	3.416337965	7.30326637	5.359802168
105375402	2.188261915	2.3862175	2.584173065	-0.352108471	-0.352108471	-0.352108471
105375439	-0.352108471	-0.352108	-0.352108471	2.039485684	-0.352108471	4.43107984
105375470	5.939192221	5.1550092	6.723375244	-0.352108471	-0.352108471	-0.352108471
105375513	5.895550985	5.3877976	6.403304371	2.328607617	4.576277023	3.45244232
105375521	5.089412202	5.5578442	4.620980171	3.68757591	4.688318557	2.686833264
105375531	7.316116953	4.282377	5.799246954	5.317240836	4.044062205	4.68065152
105375539	8.09195325	6.8018138	5.511674297	-0.352108471	-0.352108471	-0.352108471
105375551	-0.352108471	-0.352108	-0.352108471	2.168105043	4.688318557	-0.352108471
105375562	5.688622647	5.7155573	5.661687951	-0.352108471	-0.352108471	-0.352108471
105375564	7.194061177	6.8884138	6.58276648	-0.352108471	-0.352108471	-0.352108471
105375566	5.555777437	5.6532998	5.458255064	-0.352108471	-0.352108471	-0.352108471

105375567	-0.352108471	-0.352108	-0.352108471	4.716916862	7.542883219	6.12990004
105375569	7.525541673	5.9460177	6.735779686	-0.352108471	-0.352108471	-0.352108471
105375578	6.607377459	8.7841726	4.430582314	-0.352108471	-0.352108471	-0.352108471
105375587	-0.352108471	-0.352108	-0.352108471	2.962374065	2.31593942	3.60880871
105375594	5.453985405	5.1159633	5.284974356	-0.352108471	-0.352108471	-0.352108471
105375620	3.977317262	1.3835202	-1.210276951	6.204656258	10.00068093	2.408631584
105375622	-0.352108471	-0.352108	-0.352108471	4.457568814	2.660344289	3.558956552
105375631	5.530255239	7.7841866	3.276323907	-0.352108471	-0.352108471	-0.352108471
105375649	5.59738215	5.5738939	5.550405623	5.920076488	5.133155767	6.706997208
105375655	-0.352108471	-0.352108	-0.352108471	5.406707798	5.151189269	5.662226327
105375656	5.601099126	4.6461319	6.556066371	1.599335848	1.583494647	1.591415247
105375683	-0.352108471	-0.352108	-0.352108471	1.524736735	2.575541304	3.626345874
105375686	8.663779949	3.2968881	5.980334028	-0.352108471	-0.352108471	-0.352108471
105375687	-0.352108471	-0.352108	-0.352108471	4.414759241	5.169157826	5.92355641
105375707	8.106040581	5.7980585	3.490076465	5.042189825	5.243015607	4.841364044
105375713	5.290506345	4.603508	4.947007189	2.461532669	4.444204054	0.478861285
105375720	-0.352108471	-0.352108	-0.352108471	2.560258305	2.88624358	3.212228856
105375740	5.278213198	8.9629091	7.120561127	5.8849336	5.613072698	6.156794501
105375742	-0.352108471	-0.352108	-0.352108471	5.284974356	5.315258837	5.254689875
105375755	4.106210896	9.3653485	6.735779686	-0.352108471	-0.352108471	-0.352108471
105375757	6.6237638	7.9019849	7.262874357	0.440372779	0.440372779	0.440372779
105375758	7.164235655	5.3963175	3.628399272	1.848631622	1.549724107	2.147539136
105375774	6.433318728	7.93051	4.936127502	-0.352108471	-0.352108471	-0.352108471
105375780	-0.352108471	-0.352108	-0.352108471	4.80208491	4.843308497	4.760861323
105375786	-0.352108471	-0.352108	-0.352108471	5.359952855	4.226062513	4.793007684
105375793	5.852937462	7.0942851	4.611589834	-0.352108471	-0.352108471	-0.352108471
105375808	4.967066095	5.1799508	4.754181416	-0.352108471	-0.352108471	-0.352108471
105375811	2.711561556	5.7752316	-0.352108471	3.883156074	5.958359385	1.807952763
105375813	6.172790294	3.2168897	4.694839979	-0.352108471	-0.352108471	-0.352108471
105375816	2.074063343	3.0320832	2.553073296	-0.352108471	-0.352108471	-0.352108471
105375829	2.588601447	-0.352108	5.529311366	-0.352108471	-0.352108471	-0.352108471
105375835	-0.352108471	-0.352108	-0.352108471	3.794327429	4.199045311	4.603763193
105375855	-0.352108471	-0.352108	-0.352108471	7.483893437	1.429878962	4.456886199
105375882	3.955096794	5.9389176	4.947007189	-0.352108471	-0.352108471	-0.352108471
105375898	-0.352108471	-0.352108	-0.352108471	0.514115655	3.245625856	5.977136057
105375904	-0.352108471	-0.352108	-0.352108471	3.077965061	5.715557343	0.440372779
105375911	-0.352108471	-0.352108	-0.352108471	1.968661759	1.997577452	1.939746065
105375912	4.783573432	8.5646843	6.674128879	-0.352108471	-0.352108471	-0.352108471
105375917	7.729712101	4.6953603	6.212536209	-0.352108471	-0.352108471	-0.352108471
105375940	1.47406176	8.8971118	5.185586761	1.906998677	3.66091791	2.783958294
105375941	-0.352108471	-0.352108	-0.352108471	2.38621749	2.512372412	2.260062568
105375945	8.139272684	6.216547	4.293821333	4.971274502	2.596602547	3.783938524
105375950	-0.352108471	-0.352108	-0.352108471	5.575983727	5.588224375	5.563743079
105375962	5.217491139	4.5792903	5.855691969	-0.352108471	-0.352108471	-0.352108471
105376019	-0.352108471	-0.352108	-0.352108471	1.968661759	1.997577452	1.939746065
105376023	5.012258842	5.9984064	6.984554053	-0.352108471	-0.352108471	-0.352108471
105376025	-0.352108471	-0.352108	-0.352108471	5.458255064	2.338521244	8.577988884

105376032	-0.352108471	-0.352108	-0.352108471	5.20089139	5.254689875	5.147092904
105376038	-0.352108471	-0.352108	-0.352108471	3.245625856	1.671067963	4.820183749
105376043	-0.352108471	-0.352108	-0.352108471	2.313941724	-0.352108471	4.979991919
105376047	3.787792627	4.5503078	5.312822976	1.914522387	1.14221151	2.686833264
105376054	6.77374078	6.7884205	6.759061079	-0.352108471	-0.352108471	-0.352108471
105376060	2.481697	2.8278825	2.65478974	-0.352108471	-0.352108471	-0.352108471
105376066	4.628663253	4.4660231	4.547343177	0.598807818	1.549724107	-0.352108471
105376069	-0.352108471	-0.352108	-0.352108471	0.849500091	2.051108653	-0.352108471
105376104	5.861579664	5.5293114	6.193847962	1.14221151	1.14221151	1.14221151
105376111	5.409204555	6.2966704	5.852937462	-0.352108471	-0.352108471	-0.352108471
105376117	3.630849757	6.183964	8.737078274	-0.352108471	-0.352108471	-0.352108471
105376128	8.492738743	10.664143	6.321334015	8.321040554	5.23198494	6.776512747
105376146	6.861057494	6.8767254	6.845389604	-0.352108471	-0.352108471	-0.352108471
105376148	-0.352108471	-0.352108	-0.352108471	5.826557872	5.325409581	5.575983727
105376158	-0.352108471	-0.352108	-0.352108471	4.106651958	7.443484474	5.775068216
105376161	-0.352108471	-0.352108	-0.352108471	5.627005534	4.45056856	6.803442508
105376162	-0.352108471	-0.352108	-0.352108471	3.585121301	5.334128418	1.836114185
105376165	1.792110488	8.580167	5.186138762	-0.352108471	-0.352108471	-0.352108471
105376166	-0.352108471	-0.352108	-0.352108471	4.268504401	5.383503645	3.153505157
105376195	3.993556661	9.8337441	6.913650398	5.127162765	2.945847087	0.764531408
105376201	-0.352108471	-0.352108	-0.352108471	7.731292938	4.409837064	6.070565001
105376209	5.661212991	8.060902	6.861057494	-0.352108471	-0.352108471	-0.352108471
105376225	6.735779686	9.0567866	4.414772801	-0.352108471	-0.352108471	-0.352108471
105376228	-0.352108471	-0.352108	-0.352108471	2.492032424	5.336173319	-0.352108471
105376238	8.496542827	5.3832008	6.939871798	-0.352108471	-0.352108471	-0.352108471
105376239	4.295080645	5.0007509	3.589410353	0.395051519	-0.352108471	1.14221151
105376243	-0.352108471	-0.352108	-0.352108471	8.244273989	5.958359385	3.67244478
105376256	5.53725808	5.2849744	5.032690632	-0.352108471	-0.352108471	-0.352108471
105376257	-0.352108471	-0.352108	-0.352108471	1.400698343	-0.352108471	3.153505157
105376266	-0.352108471	-0.352108	-0.352108471	5.637296679	5.653299811	5.621293547
105376272	5.4641134	5.5599715	5.655829615	2.881499807	3.615460478	2.147539136
105376290	5.402061931	5.5587679	5.715473809	-0.352108471	-0.352108471	-0.352108471
105376312	3.70869183	5.5124423	4.610567068	-0.352108471	-0.352108471	-0.352108471
105376317	-0.352108471	-0.352108	-0.352108471	1.946552907	4.245214285	-0.352108471
105376318	-0.352108471	-0.352108	-0.352108471	8.340490145	6.434644758	4.528799372
105376322	-0.352108471	-0.352108	-0.352108471	2.124237496	2.028091952	2.22038304
105376324	5.284974356	3.7809281	6.789020613	-0.352108471	-0.352108471	-0.352108471
105376326	7.581497581	3.8180246	5.69976107	2.43572631	0.440372779	4.43107984
105376338	4.792303669	4.8304259	4.754181416	-0.352108471	-0.352108471	-0.352108471
105376384	8.216975399	5.8242888	3.431602171	2.100935855	2.100935855	2.100935855
105376387	5.493783215	6.2532684	4.73429806	1.223721097	1.14221151	1.305230684
105376388	-0.352108471	-0.352108	-0.352108471	3.058133858	5.760537073	8.462940288
105376394	-0.352108471	-0.352108	-0.352108471	1.90830372	1.939746065	1.876861374
105376425	-0.352108471	-0.352108	-0.352108471	5.907948707	5.233567424	4.55918614
105376427	5.813110936	5.1065363	6.519685616	3.997152479	2.18436845	3.090760465
105376434	-0.352108471	-0.352108	-0.352108471	7.735227386	4.797507002	6.266367194
105376436	-0.352108471	-0.352108	-0.352108471	4.879761376	5.663737322	6.447713268

105376464	2.618057952	1.6808649	3.555250981	-0.352108471	-0.352108471	-0.352108471
105376474	-0.352108471	-0.352108	-0.352108471	2.974588569	7.059684302	5.017136436
105376491	5.825318483	4.3257206	5.075519558	2.496320744	0.792032047	4.20060944
105376513	5.152247113	3.4244886	6.880005656	-0.352108471	-0.352108471	-0.352108471
105376516	-0.352108471	-0.352108	-0.352108471	3.73714403	3.919505936	3.554782125
105376546	-0.352108471	-0.352108	-0.352108471	5.412561087	5.421579811	5.403542364
105376554	6.136667708	6.6378887	7.139109629	3.699359721	3.762236766	3.636482675
105376560	-0.352108471	-0.352108	-0.352108471	2.761826744	4.007138891	3.384482817
105376562	6.738649524	6.8312892	6.923928891	-0.352108471	-0.352108471	-0.352108471
105376591	2.764225616	0.2585353	5.269915917	-0.352108471	-0.352108471	-0.352108471
105376601	-0.352108471	-0.352108	-0.352108471	2.686833264	2.686833264	2.686833264
105376612	4.854844895	6.3954766	5.625160771	-0.352108471	-0.352108471	-0.352108471
105376620	-0.352108471	-0.352108	-0.352108471	2.501526031	-0.352108471	5.355160532
105376630	6.64818489	5.7494021	4.850619339	-0.352108471	-0.352108471	-0.352108471
105376632	-0.352108471	-0.352108	-0.352108471	1.631676003	3.615460478	-0.352108471
105376677	5.246056936	4.256114	6.235999889	7.343465972	3.664906032	5.504186002
105376681	3.449995986	4.9774847	6.504973388	0.476561106	1.305230684	-0.352108471
105376684	4.537335758	5.366581	3.708090482	-0.352108471	-0.352108471	-0.352108471
105376685	-0.352108471	-0.352108	-0.352108471	1.880238567	-0.352108471	4.112585606
105376705	2.523212107	-0.352108	5.398532684	-0.352108471	-0.352108471	-0.352108471
105376716	9.127181534	3.3266592	6.226920361	0.735127777	2.571154353	4.407180928
105376735	2.197246788	3.1742174	2.685732087	-0.352108471	-0.352108471	-0.352108471
105376737	-0.352108471	-0.352108	-0.352108471	1.400698343	4.051124344	-1.249727659
105376743	6.034554801	5.2157667	5.625160771	-0.352108471	-0.352108471	-0.352108471
105376776	4.161423262	6.8850562	5.523239719	-0.352108471	-0.352108471	-0.352108471
105376779	6.967511245	6.9786128	6.956409727	-0.352108471	-0.352108471	-0.352108471
105376782	-0.352108471	-0.352108	-0.352108471	2.577819651	2.793732842	3.009646032
105376785	-0.352108471	-0.352108	-0.352108471	3.119148782	3.119148782	3.119148782
105376794	4.201099247	3.9195059	4.482692559	2.039485684	-0.352108471	4.43107984
105376807	-0.352108471	-0.352108	-0.352108471	6.3862009	5.939192221	5.492183543
105376813	-0.352108471	-0.352108	-0.352108471	5.789762726	5.804293869	5.775231583
105376831	6.674128879	6.797202	6.551055783	-0.352108471	-0.352108471	-0.352108471
105376832	-0.352108471	-0.352108	-0.352108471	0.874413692	-0.352108471	2.100935855
105376851	-0.352108471	-0.352108	-0.352108471	3.716380147	2.221964512	0.727548877
105376856	6.276782173	5.3236174	5.800199788	-0.352108471	-0.352108471	-0.352108471
105376857	5.688622647	7.457536	3.919709244	-0.352108471	-0.352108471	-0.352108471
105376868	-0.352108471	-0.352108	-0.352108471	3.168357904	8.875743946	6.022050925
105376869	2.8251001	-0.352108	6.002308672	-0.352108471	-0.352108471	-0.352108471
105376875	5.435636528	5.6818992	5.55876787	-0.352108471	-0.352108471	-0.352108471
105376879	-0.352108471	-0.352108	-0.352108471	2.19530121	3.875472802	5.555644394
105376896	6.181464618	7.8378304	7.009647498	4.394233843	4.705448172	4.083019514
105376900	6.621016522	6.3152311	6.926801914	3.899998205	4.245214285	3.554782125
105376908	-0.352108471	-0.352108	-0.352108471	3.998406827	5.686783237	2.310030417
105376909	-0.352108471	-0.352108	-0.352108471	0.392735488	3.500370326	1.946552907
105376911	5.801482926	6.4033455	5.199620343	2.176777034	2.252618212	2.100935855
105376916	6.333874251	7.4387432	6.886308748	-0.352108471	-0.352108471	-0.352108471
105376918	4.22804496	4.0798907	4.153967827	1.77365078	1.220653946	2.326647613

105376927	1.048715362	2.7402102	4.431705103	-0.352108471	-0.352108471	-0.352108471
105376934	7.758360446	5.0353763	6.396868383	-0.352108471	-0.352108471	-0.352108471
105376938	-0.352108471	-0.352108	-0.352108471	1.346495496	2.252618212	0.440372779
105376942	1.920459576	2.5156976	3.110935598	-0.352108471	-0.352108471	-0.352108471
105376956	1.149715101	1.6345034	1.392109249	6.088761404	6.285554459	6.482347513
105376973	4.757610744	2.5701985	6.945022943	3.434816223	7.365017787	11.29521935
105376980	5.855297211	6.6737632	7.492229135	0.542875446	-0.352108471	1.437859363
105376984	1.383520155	0.0593603	2.707679997	2.241824978	5.219123492	8.196422005
105376994	7.137727412	7.6319432	6.643511666	-0.352108471	-0.352108471	-0.352108471
105377006	-0.352108471	-0.352108	-0.352108471	5.596916991	5.605609608	5.588224375
105377040	-0.352108471	-0.352108	-0.352108471	4.279062203	6.410585269	2.147539136
105377050	3.41407487	3.6660236	3.1621261	3.730639943	6.910870138	5.32075504
105377053	-0.352108471	-0.352108	-0.352108471	2.252618212	1.630384959	2.874851464
105377057	5.140914746	4.3639534	3.586992098	-0.512397393	2.062346313	4.63709002
105377059	-0.352108471	-0.352108	-0.352108471	0.95025487	-0.352108471	2.252618212
105377077	-0.352108471	-0.352108	-0.352108471	5.231098346	2.024343053	-1.182412241
105377082	5.2243927	5.632395	5.428393874	-0.352108471	-0.352108471	-0.352108471
105377095	-0.352108471	-0.352108	-0.352108471	2.039485684	-0.352108471	4.43107984
105377100	5.416362038	5.8339595	5.625160771	-0.352108471	-0.352108471	-0.352108471
105377135	-0.352108471	-0.352108	-0.352108471	5.591132762	5.494417177	5.397701591
105377167	3.993171587	4.8902834	4.441727503	0.647185709	-0.352108471	1.646479888
105377170	2.201036472	4.7541814	-0.352108471	5.789762726	5.804293869	5.775231583
105377177	-0.352108471	-0.352108	-0.352108471	6.266367194	8.402117206	4.130617182
105377195	5.420390631	5.0021813	5.838599938	1.995427359	2.947319917	1.043534802
105377198	3.892576082	5.719565	7.546553907	-0.352108471	-0.352108471	-0.352108471
105377225	5.689140061	5.8243239	5.553956231	-0.352108471	-0.352108471	-0.352108471
105377251	-0.352108471	-0.352108	-0.352108471	3.806529484	1.880238567	-0.046052349
105377265	4.01084683	2.5530733	1.095299763	3.382913164	3.784559474	4.186205783
105377267	1.783698732	3.9195059	-0.352108471	3.919505936	3.919505936	3.919505936
105377292	10.95949515	3.3467648	7.153129997	1.58479924	0.736841864	2.432756617
105377294	6.243962761	6.5546628	6.865362884	-0.352108471	-0.352108471	-0.352108471
105377295	4.947007189	5.2901056	4.603908745	4.282459364	5.958359385	7.634259405
105377310	-0.352108471	-0.352108	-0.352108471	4.495751253	6.149433787	7.803116321
105377319	5.620891261	4.8651455	6.376637049	0.476561106	-0.352108471	1.305230684
105377320	-0.352108471	-0.352108	-0.352108471	5.723485516	6.86786121	4.579109821
105377328	7.82455912	5.7790684	6.801813774	-0.352108471	-0.352108471	-0.352108471
105377352	1.709904749	2.1035728	2.497240816	6.001809131	4.868750345	7.134867918
105377360	-0.352108471	-0.352108	-0.352108471	5.596916991	6.193634888	5.000199094
105377362	4.197208934	2.6857321	1.174255241	3.773917231	4.129105482	3.418728979
105377378	-0.352108471	-0.352108	-0.352108471	6.149337851	8.792934731	3.50574097
105377400	7.138933258	6.3968684	5.654803507	-0.352108471	-0.352108471	-0.352108471
105377421	5.253240792	6.3193075	4.187174113	-0.352108471	-0.352108471	-0.352108471
105377425	-0.352108471	-0.352108	-0.352108471	4.73755266	4.945386437	4.529718883
105377436	-0.352108471	-0.352108	-0.352108471	4.54785697	5.723779385	5.135818178
105377442	7.297470204	8.7416345	8.019552374	6.713858687	3.999320952	9.428396423
105377445	2.201036472	1.6469568	2.755116138	-0.352108471	-0.352108471	-0.352108471
105377458	5.021401806	3.1277379	1.234073944	1.601336827	-0.352108471	3.554782125

105377464	6.467824574	5.6886226	4.90942072	-0.352108471	-0.352108471	-0.352108471
105377466	-0.352108471	-0.352108	-0.352108471	6.750362314	5.388882507	6.06962241
105377508	5.152247113	5.0776438	5.226850463	-0.352108471	-0.352108471	-0.352108471
105377533	5.514819679	5.5587679	5.602716062	-0.352108471	-0.352108471	-0.352108471
105377567	6.183964016	6.1999612	6.167966808	-0.352108471	-0.352108471	-0.352108471
105377575	-0.352108471	-0.352108	-0.352108471	7.968841277	1.334967077	4.651904177
105377582	-0.352108471	-0.352108	-0.352108471	2.43016825	-0.352108471	5.212444972
105377597	-0.352108471	-0.352108	-0.352108471	5.169157826	4.818593164	5.519722487
105377600	5.634592072	5.5460431	5.723141053	0.624614178	0.808855576	0.440372779
105377616	4.878992132	6.283251	5.581121573	-0.352108471	-0.352108471	-0.352108471
105377638	6.090808341	6.1079622	6.073654487	4.716557081	3.615460478	5.817653684
105377639	4.736638248	1.8973409	7.575935553	1.30840417	3.170669557	-0.553861217
105377650	-0.352108471	-0.352108	-0.352108471	5.412561087	5.421579811	5.403542364
105377690	-0.352108471	-0.352108	-0.352108471	5.847512993	4.891151184	5.369332089
105377712	6.303461245	6.5974928	6.009429738	-0.352108471	-0.352108471	-0.352108471
105377716	-0.352108471	-0.352108	-0.352108471	1.601517443	2.888273049	4.175028656
105377730	5.688622647	5.7155573	5.661687951	-0.352108471	-0.352108471	-0.352108471
105377732	2.740210233	-0.352108	5.832528937	-0.352108471	-0.352108471	-0.352108471
105377737	-0.352108471	-0.352108	-0.352108471	5.412561087	4.869345447	5.955776728
105377740	-0.352108471	-0.352108	-0.352108471	5.128763636	6.145629444	4.111897827
105377757	6.020381091	6.4097672	5.630995022	-0.352108471	-0.352108471	-0.352108471
105377776	5.354685649	5.4498053	5.259566032	-0.352108471	-0.352108471	-0.352108471
105377780	-0.352108471	-0.352108	-0.352108471	0.191417513	5.614564532	2.902991023
105377782	-0.352108471	-0.352108	-0.352108471	5.511864271	3.805197278	7.218531264
105377813	5.765810019	6.0023087	5.529311366	7.667232662	7.692669739	7.641795584
105377817	5.748354287	7.9533982	3.543310417	-0.352108471	-0.352108471	-0.352108471
105377819	-0.352108471	-0.352108	-0.352108471	4.80208491	4.843308497	4.760861323
105377856	-0.352108471	-0.352108	-0.352108471	5.426606128	6.235921161	7.045236194
105377859	5.493783215	6.5851193	4.402447165	-0.352108471	-0.352108471	-0.352108471
105377861	5.87518164	6.3968684	6.918555125	-0.352108471	-0.352108471	-0.352108471
105377894	-0.352108471	-0.352108	-0.352108471	5.117340897	5.254689875	4.979991919
105377896	5.118418872	6.9223433	6.020381091	-0.352108471	-0.352108471	-0.352108471
105377907	1.946552907	0.8679093	3.025196512	4.942702387	6.588043781	3.297360993
105377908	4.46380013	4.8786655	5.293530827	-0.352108471	-0.352108471	-0.352108471
105377919	-0.352108471	-0.352108	-0.352108471	4.005546737	7.441424294	5.723485516
105377928	-0.352108471	-0.352108	-0.352108471	7.894586032	4.899150733	6.396868383
105377937	5.181747782	5.8046669	4.558828652	1.190059422	0.440372779	1.939746065
105377940	6.686076964	5.0547547	3.42343246	-0.352108471	-0.352108471	-0.352108471
105377958	-0.352108471	-0.352108	-0.352108471	5.149872953	5.095423988	5.040975023
105377986	6.303461245	7.9362493	4.670673235	-0.352108471	-0.352108471	-0.352108471
105378027	-0.352108471	-0.352108	-0.352108471	2.274515706	3.30547384	1.243557573
105378031	4.928960211	4.211158	4.570059097	3.151146871	3.615460478	2.686833264
105378049	-0.352108471	-0.352108	-0.352108471	4.178899946	4.245214285	4.112585606
105378090	-0.352108471	-0.352108	-0.352108471	5.760537073	5.775231583	5.745842563
105378095	-0.352108471	-0.352108	-0.352108471	6.296043865	6.31523113	6.276856601
105378098	8.694180684	6.7389281	4.783675536	6.404996167	6.280301159	6.15560615
105378131	-0.352108471	-0.352108	-0.352108471	5.425370388	6.087644454	6.74991852

105378141	-0.352108471	-0.352108	-0.352108471	5.473547957	5.458255064	5.442962171
105378146	-0.352108471	-0.352108	-0.352108471	0.82273449	-0.352108471	1.997577452
105378147	5.55876787	5.5882244	5.529311366	-0.352108471	-0.352108471	-0.352108471
105378150	-0.352108471	-0.352108	-0.352108471	4.174760939	6.016087037	5.095423988
105378151	-0.352108471	-0.352108	-0.352108471	4.29127799	4.66005769	5.028837389
105378152	5.428393874	5.015001	5.841786766	-0.352108471	-0.352108471	-0.352108471
105378155	-0.352108471	-0.352108	-0.352108471	6.639034896	4.807936135	5.723485516
105378173	-0.352108471	-0.352108	-0.352108471	4.650211429	4.66990395	4.66005769
105378176	5.595499658	5.2761208	5.914878506	0.624614178	0.808855576	0.440372779
105378185	6.507456839	6.2125362	5.917615578	2.481575183	2.934478494	2.028671872
105378190	7.000225237	5.6162528	4.232280409	-0.352108471	-0.352108471	-0.352108471
105378193	-0.352108471	-0.352108	-0.352108471	3.077569294	0.489828171	1.783698732
105378238	6.831289207	9.7248101	3.937768275	-0.352108471	-0.352108471	-0.352108471
105378275	2.62245663	0.5388276	4.706085695	-0.352108471	-0.352108471	-0.352108471
105378308	5.742914374	7.9228776	3.562951127	-0.352108471	-0.352108471	-0.352108471
105378309	-0.352108471	-0.352108	-0.352108471	2.397492216	-0.352108471	5.147092904
105378315	5.043534014	3.2891072	6.797960846	1.493791735	1.549724107	1.437859363
105378341	1.392109249	3.136327	-0.352108471	1.902765107	1.997577452	1.807952763
105378390	-0.352108471	-0.352108	-0.352108471	4.453901852	4.456886199	4.459870547
105378395	6.107538801	6.1414231	6.073654487	-0.352108471	-0.352108471	-0.352108471
105378415	2.992608695	4.8786655	6.764722261	-0.352108471	-0.352108471	-0.352108471
105378427	7.387935135	4.652827	6.020381091	-0.352108471	-0.352108471	-0.352108471
105378432	5.947824374	5.6251608	5.302497169	-0.352108471	-0.352108471	-0.352108471
105378451	-0.352108471	-0.352108	-0.352108471	2.065292044	4.482692559	-0.352108471
105378469	6.433318728	5.4078516	7.458785844	-0.352108471	-0.352108471	-0.352108471
105378488	8.397137724	5.1522471	1.907356502	-0.352108471	-0.352108471	-0.352108471
105378506	-0.352108471	-0.352108	-0.352108471	6.5207905	5.017136436	3.513482371
105378512	7.496427806	7.5676522	7.425203426	-0.352108471	-0.352108471	-0.352108471
105378517	-0.352108471	-0.352108	-0.352108471	4.380566524	5.439917437	6.499268351
105378525	5.625160771	5.6532998	5.597021732	4.927679215	4.954218546	4.901139884
105378526	-0.352108471	-0.352108	-0.352108471	6.291947162	5.575983727	4.860020291
105378527	-0.352108471	-0.352108	-0.352108471	5.233567424	5.254689875	5.212444972
105378538	6.03798158	6.0736545	6.002308672	-0.352108471	-0.352108471	-0.352108471
105378550	5.926519726	5.0171364	4.107753145	-0.352108471	-0.352108471	-0.352108471
105378565	-0.352108471	-0.352108	-0.352108471	7.528868501	6.039651413	4.550434326
105378579	-0.352108471	-0.352108	-0.352108471	2.419557138	0.978447378	3.860666899
105378586	4.496247384	5.873346	3.119148782	-0.352108471	-0.352108471	-0.352108471
105378598	8.452104911	9.6471416	7.25706822	1.697414861	1.14221151	2.252618212
105378643	6.311337777	6.2363267	6.273832259	-0.352108471	-0.352108471	-0.352108471
105378669	5.529311366	5.5293114	5.529311366	2.931568264	5.054280952	0.808855576
105378670	4.583433279	4.1125856	5.054280952	0.044132154	-0.352108471	0.440372779
105378683	5.749402114	5.0236502	6.47515398	-0.352108471	-0.352108471	-0.352108471
105378695	-0.352108471	-0.352108	-0.352108471	5.494417177	3.70798208	7.280852273
105378720	5.284974356	5.3152588	5.254689875	-0.352108471	-0.352108471	-0.352108471
105378725	-0.352108471	-0.352108	-0.352108471	4.172619225	4.175292888	4.17796655
105378726	4.220681375	5.8154729	7.410264413	-0.352108471	-0.352108471	-0.352108471
105378742	-0.352108471	-0.352108	-0.352108471	4.66005769	5.17533982	4.144775559

105378753	-0.352108471	-0.352108	-0.352108471	8.124937532	3.98234523	6.053641381
105378759	7.527177696	7.6875967	7.366758653	2.854586112	0.460134624	1.657360368
105378775	7.477981313	5.9391922	4.40040313	-0.352108471	-0.352108471	-0.352108471
105378805	6.539661058	4.2126095	8.866712594	-0.352108471	-0.352108471	-0.352108471
105378813	4.878665478	4.9140225	4.843308497	-0.352108471	-0.352108471	-0.352108471
105378816	-0.352108471	-0.352108	-0.352108471	4.602445978	3.755353914	4.178899946
105378841	-0.352108471	-0.352108	-0.352108471	2.271463724	3.102202803	2.686833264
105378842	1.383520155	-0.352108	3.119148782	3.196516302	4.843308497	1.549724107
105378849	1.989646424	5.6574939	9.325341338	-0.352108471	-0.352108471	-0.352108471
105378857	4.292030912	5.0082279	5.724424816	6.397752545	9.73622985	8.066991197
105378863	-0.352108471	-0.352108	-0.352108471	2.091873588	6.821898811	4.456886199
105378902	-0.352108471	-0.352108	-0.352108471	6.335196408	7.679732575	4.99066024
105378910	6.090808341	7.2841592	4.897457494	-0.352108471	-0.352108471	-0.352108471
105378913	-0.352108471	-0.352108	-0.352108471	4.176881732	0.595553249	2.38621749
105378938	1.383520155	-0.352108	3.119148782	4.864757091	4.617382386	4.370007682
105378939	-0.352108471	-0.352108	-0.352108471	5.104454211	4.726111306	5.482797115
105378951	-0.352108471	-0.352108	-0.352108471	0.940233577	2.232575625	-0.352108471
105378957	5.223934733	4.3218845	4.772909641	5.230387232	5.812486125	5.521436679
105378958	9.146664356	4.9099316	7.028297964	2.92016921	1.510721577	4.329616843
105378978	3.969195455	3.8943622	3.819528982	-0.3795483	6.508720747	3.064586224
105378979	5.90029238	5.9200251	5.880559702	-0.352108471	-0.352108471	-0.352108471
105379005	5.287855205	5.0531527	5.522557694	2.147539136	2.147539136	2.147539136
105379010	6.369156591	6.3831515	6.355161685	-0.352108471	-0.352108471	-0.352108471
105379016	6.832218283	4.0245695	5.428393874	4.178899946	3.878191396	4.479608495
105379021	-0.352108471	-0.352108	-0.352108471	2.252618212	2.252618212	2.252618212
105379060	-0.352108471	-0.352108	-0.352108471	6.056053999	4.82877742	7.283330577
105379072	5.656289537	5.1945146	6.118064495	0.872801732	0.440372779	1.305230684
105379090	3.539717369	6.183964	8.828210662	0.044132154	-0.352108471	0.440372779
105379100	-0.352108471	-0.352108	-0.352108471	2.411083697	2.546625611	2.682167526
105379117	-0.352108471	-0.352108	-0.352108471	5.529311366	6.815591889	4.243030843
105379119	-0.352108471	-0.352108	-0.352108471	4.354889986	4.79472922	5.234568455
105379126	-0.352108471	-0.352108	-0.352108471	3.119148782	3.219709646	3.018587918
105379137	-0.352108471	-0.352108	-0.352108471	2.515697587	5.383503645	-0.352108471
105379146	-0.352108471	-0.352108	-0.352108471	5.095423988	3.696255077	6.494592899
105379165	7.711121501	8.2047969	7.217446092	5.07328681	4.688318557	5.458255064
105379167	-0.352108471	-0.352108	-0.352108471	0.510474327	2.481575183	4.452676039
105379177	6.870096176	7.041464	6.698728325	3.8113611	5.506320168	7.201279237
105379180	-0.352108471	-0.352108	-0.352108471	2.625545239	1.929530708	1.233516178
105379183	-0.352108471	-0.352108	-0.352108471	0.842360745	3.119148782	5.395936818
105379200	7.129322625	7.0425231	6.955723541	-0.352108471	-0.352108471	-0.352108471
105379205	6.090808341	7.8336728	4.347943883	-0.352108471	-0.352108471	-0.352108471
105379206	-0.352108471	-0.352108	-0.352108471	5.923493182	5.926961306	5.920025058
105379223	8.545455107	9.4775239	10.40959273	8.825256836	8.498729794	8.661993315
105379227	5.492104853	5.2171573	5.35463107	-0.352108471	-0.352108471	-0.352108471
105379250	7.491501957	6.2125362	4.93357046	-0.352108471	-0.352108471	-0.352108471
105379251	-0.352108471	-0.352108	-0.352108471	5.197821579	8.60946277	6.903642175
105379252	-0.352108471	-0.352108	-0.352108471	5.390930934	5.715557343	5.066304524

105379256	-0.352108471	-0.352108	-0.352108471	4.353126573	5.27665889	4.814892732
105379279	2.642407329	6.5944666	4.618436987	-0.352108471	-0.352108471	-0.352108471
105379288	-0.352108471	-0.352108	-0.352108471	6.433682579	6.918655486	6.676169033
105379291	-0.352108471	-0.352108	-0.352108471	3.024357264	3.213940299	3.119148782
105379301	2.588601447	5.5293114	-0.352108471	-0.352108471	-0.352108471	-0.352108471
105379302	5.523239719	5.6573174	5.389162088	-0.352108471	-0.352108471	-0.352108471
105379311	6.242048279	6.2558778	6.228218771	-0.352108471	-0.352108471	-0.352108471
105379356	7.990771225	5.4344397	2.878108208	-0.352108471	-0.352108471	-0.352108471
105379361	6.96369403	5.4851236	4.006553188	-0.352108471	-0.352108471	-0.352108471
105379364	7.003358054	6.1555609	6.579459461	0.86953393	0.808855576	0.930212283
105379365	-0.352108471	-0.352108	-0.352108471	4.613083451	5.210216965	4.911650208
105379369	-0.352108471	-0.352108	-0.352108471	7.803705017	4.971504216	2.139303415
105379377	-0.352108471	-0.352108	-0.352108471	8.849990258	6.022050925	3.194111592
105379398	-0.352108471	-0.352108	-0.352108471	2.492032424	5.336173319	-0.352108471
105379405	2.351086241	-0.352108	5.054280952	-0.352108471	-0.352108471	-0.352108471
105379406	-0.352108471	-0.352108	-0.352108471	5.662971356	5.97985032	5.821410838
105379408	4.730615771	4.5722278	4.88900371	3.228447668	3.337746555	3.119148782
105379413	5.049130657	7.1924906	6.120810648	-0.352108471	-0.352108471	-0.352108471
105379418	-0.352108471	-0.352108	-0.352108471	5.465839294	4.123619146	4.79472922
105379420	7.450449055	7.4583733	7.442524773	-0.352108471	-0.352108471	-0.352108471
105379422	4.493470537	6.0203811	7.547291646	3.239272365	5.637738742	4.438505554
105379423	5.633937955	7.6532016	9.67246524	6.54435867	6.630837149	6.717315627
105379431	-0.352108471	-0.352108	-0.352108471	4.456886199	4.482692559	4.43107984
105379440	2.84317252	-0.352108	6.038453511	-0.352108471	-0.352108471	-0.352108471
105379443	5.38967036	5.9875749	5.688622647	-0.352108471	-0.352108471	-0.352108471
105379448	7.506677825	7.508442	7.504913637	-0.352108471	-0.352108471	-0.352108471
105379467	-0.352108471	-0.352108	-0.352108471	2.43016825	2.110900477	2.749436024
105379477	5.509495557	5.2437595	5.775231583	3.046836295	5.653299811	0.440372779
105379494	5.871254895	6.3439771	5.398532684	-0.352108471	-0.352108471	-0.352108471
105379498	-0.352108471	-0.352108	-0.352108471	2.501526031	-0.352108471	5.355160532
105379516	6.151585628	7.7119342	4.59123706	2.076022254	1.300422222	2.851622287
105379520	6.739149467	6.6898785	6.788420481	6.147741192	6.101634422	6.193847962
105379523	7.664080745	6.1848209	4.705561142	8.305949299	4.97239442	6.63917186
105379527	3.582547382	5.0799713	6.577395312	0.044132154	-0.352108471	0.440372779
105379536	5.522595798	5.0342263	6.010965254	-0.352108471	-0.352108471	-0.352108471
105379547	5.745480655	6.9166391	6.331059877	6.571174876	5.233893299	5.902534088
105379549	6.294619744	5.6024012	6.986838314	5.695475335	6.087644454	6.479813574
105379551	7.155086519	6.448541	6.801813774	-0.352108471	-0.352108471	-0.352108471
105379554	-0.352108471	-0.352108	-0.352108471	1.842407068	1.807952763	1.876861374
105379555	-0.352108471	-0.352108	-0.352108471	5.322153946	6.086516564	5.704335255
105379557	2.934655352	2.4868796	2.03910383	-0.352108471	-0.352108471	-0.352108471
105379564	6.216915331	6.9675112	7.718107158	-0.352108471	-0.352108471	-0.352108471
105379581	-0.352108471	-0.352108	-0.352108471	1.979372981	2.444513457	2.211943219
105379587	5.652271474	6.6593365	4.645206473	7.09094637	5.784960503	4.478974636
105379615	4.97352783	5.3152588	4.631796822	-0.352108471	-0.352108471	-0.352108471
105379629	4.744327548	4.4173531	4.090378577	-0.352108471	-0.352108471	-0.352108471
105379661	-0.352108471	-0.352108	-0.352108471	6.105008867	4.66005769	3.215106512

105379676	5.428393874	6.0814598	4.775327955	-0.352108471	-0.352108471	-0.352108471
105379679	-0.352108471	-0.352108	-0.352108471	3.182489739	6.13762564	4.66005769
105379680	4.367830071	3.6642615	4.016045771	5.579094101	6.533013897	6.056053999
105379687	1.551874638	1.2151657	1.383520155	4.811096552	4.706961556	4.60282656
105379689	5.493783215	6.9577196	4.029846782	5.245661134	2.919059086	4.08236011
105379702	6.293623131	4.3347012	2.375779346	-0.352108471	-0.352108471	-0.352108471
105379715	-0.352108471	-0.352108	-0.352108471	5.295078103	5.712273256	4.87788295
105379724	7.882101328	6.9163731	8.84782955	7.178690343	5.453994843	3.729299344
105379728	5.937827329	7.842619	4.033035626	-0.352108471	-0.352108471	-0.352108471
105379734	4.287526266	4.2109961	4.249261192	8.228687422	4.363400309	6.296043865
105379742	0.425291339	4.537859	2.481575183	3.03914168	2.397492216	1.755842753
105379748	7.688088734	5.980334	4.272579322	-0.352108471	-0.352108471	-0.352108471
105379752	-0.352108471	-0.352108	-0.352108471	3.119148782	3.893819184	2.344478379
105379756	6.572931339	5.8001998	5.027468236	2.094444132	1.997577452	2.191310813
105379760	6.416357783	7.7695198	5.063195737	-0.352108471	-0.352108471	-0.352108471
105379767	5.899362839	5.8369584	5.774553889	-0.352108471	-0.352108471	-0.352108471
105379768	2.558401675	6.3410962	4.449748927	6.223209964	3.963030129	5.093120047
105379792	6.720370627	6.7508628	6.689878453	-0.352108471	-0.352108471	-0.352108471
105379799	-0.352108471	-0.352108	-0.352108471	4.66005769	4.688318557	4.631796822
105379803	-0.352108471	-0.352108	-0.352108471	5.077914249	4.477947052	3.877979854
105379806	7.650334664	6.8469571	6.04357953	3.73714403	5.042654972	2.431633088
105379807	1.492879131	4.1573211	2.8251001	-0.352108471	-0.352108471	-0.352108471
105379808	-0.352108471	-0.352108	-0.352108471	5.88700099	6.943528322	4.830473658
105379809	7.184663343	5.5765095	8.792817219	5.11458226	4.600497609	4.857539934
105379816	2.588601447	-0.352108	5.529311366	1.76984594	1.807952763	1.731739118
105379818	4.992568101	5.4424142	5.217491139	5.993376219	8.812718376	7.403047298
105379819	10.59963574	9.1599607	7.720285641	8.092037136	6.77374078	5.455444424
105379822	-0.352108471	-0.352108	-0.352108471	0.949604797	4.637194169	8.32478354
105379826	-0.352108471	-0.352108	-0.352108471	5.458255064	7.859500965	3.057009163
105379833	-0.352108471	-0.352108	-0.352108471	4.456886199	6.055883089	2.85788931
105379834	5.836275906	4.5750389	3.313801928	4.66005769	6.667586145	2.652529234
105379838	4.598204327	4.6565592	4.714914154	3.245625856	3.337746555	3.153505157
105379848	5.689216459	5.980334	6.271451597	4.976042035	1.969230181	7.982853889
105379853	4.943335066	6.519674	8.096012849	0.395051519	1.14221151	-0.352108471
105379854	-0.352108471	-0.352108	-0.352108471	5.44897977	5.494417177	5.403542364
105379861	5.800199788	5.3020742	6.298325346	-0.352108471	-0.352108471	-0.352108471
105379862	6.720370627	5.8221214	7.618619828	6.101164691	5.284974356	4.468784021
105379880	6.77374078	6.7884205	6.759061079	6.105896993	6.246629235	5.965164752
105379881	-0.352108471	-0.352108	-0.352108471	4.617117504	5.417155367	5.017136436
105747689	5.564591206	4.4504318	6.678750612	8.729582432	6.795027168	4.860471904
105748977	7.136372074	6.1796743	6.658023207	-0.352108471	-0.352108471	-0.352108471
106182249	4.731111372	4.8304259	4.631796822	4.977710418	5.054280952	4.901139884
106478957	4.455779276	4.9170441	3.994514455	0.689815323	-0.352108471	1.731739118
106478991	5.718716224	4.0451438	7.392288636	5.305586236	5.326967336	5.316276786
106614088	5.915852686	6.6003588	5.231346598	5.284336357	6.114004174	4.45466854
106635544	7.112826218	1.051894	4.08236011	4.973858514	5.222210236	5.470561958
106660613	-0.352108471	-0.352108	-0.352108471	4.777811646	-0.352108471	1.930583232
