# Supplementary material for: Cytogenetic and Transcriptomic Analysis of Human Endometrial MSC Retaining Proliferative Activity after Sublethal Heat Shock
Source: Cells. 2018 Oct 25;7(11):184. doi: 10.3390/cells7110184 (PMC6262560; doi:10.3390/cells7110184)
Supplement: Supplementary file 1 [file cells-07-00184-s001.zip › cells-363926-supp/Supplementary Table 2.pdf]

Supplementary Table 2. Chromosomal changes revealed by the G-banding method in control human eMSCs and in SHS-SP.

A -Chromosomal changes revealed by the G-banding method in human eMSCs (Control cells) at passage 13 - 10% cells with changes

Chromosome №	1	2	3	4	5	6	7	8	9	10	11	12	13	14	15	16	17	18	19	20	21	22	x	n
1																								46
2							m					m									m	m	m	41
3	m														iso								m	44
4																								46
5																								46
6																								46
7							t						13:14					m						46
8																								46
9																								46
10																								46
11																								46
12																								46
13																								46
14																								46
15																								46
16																								46
17																								46
18																								46
19																								46
20																								46
21																								46
22																								46
23																								46
24																								46
25																								46

**21**																								**46**
**22**																								**46**
**23**																								**46**
**24**																								**46**
**25**																								**46**

B -Chromosomal changes revealed by the G-banding method in SHS-SP at passage 13 (6 passage after HS) – 80% cells with changes

**Chromosome №**	**1**	**2**	**3**	**4**	**5**	**6**	**7**	**8**	**9**	**10**	**11**	**12**	**13**	**14**	**15**	**16**	**17**	**18**	**19**	**20**	**21**	**22**	**x**	**n**
**1**	b(add)		t		b(del)					m	m+b(add)			t						m				**45**
**2**			t							m		b(add)	m		m				t			t		**46**
**3**	t+b(del)		t+b(add)								b(del)	m					m							**46**
**4**								b																**46**
**5**		b(del)			b(del)	b			m		m		m	m				m	m	m			m	**37**
**6**			b(del)								b(del)													**46**
**7**																								**46**
**8**																								**46**
**9**	t+b(del)																							**47**
**10**	t+b(add)	t+b(del)	b(add)							b(del)			a										a	**44**
**11**		b(del)	b(del)	b(del)				m			m											a		**42**
**12**																								**46**
**13**								b(del)							m									**45**
**14**				b(del)	b(del)	m												b(del)						**45**
**15**		m	m				m			m			m				m							**40**
**16**		b(del)																		m			m	**44**
**17**	t+b(del)	b(add)		m						b(del)		a				m						m		**42**
**18**				b(del)																				**46**
**19**						b(del)																		**46**
**20**		b(del)		b(del)																				**46**

**21**		b(add)		b(del)				b(del)			m	b(add)					m			t		m	a	**42**
**22**																								**46**
**23**																								**46**
**24**																								**46**
**25**	t+b(del)																					m		**46**
**26**	b(del)	t+b(del)	t+b(del)						m		m	m												**45**
**27**					b(del)					b(del)	a					m			m		m	m	t	**41**
**28**					m		t								m	m				m				**43**
**29**		b(del)	t+b(del)			m				m	m												m	**43**

m - monosomy;

t - trisomy;

b(del) – break of chromosome with deletion;

b(add) – break of chromosome with preservation of genetic material;

iso – isochromosome;

13:14 - Ectopic conjugation between chromosomes 13 and 14

t+b – trisomy and chromosomal break

m+b - monosomy and chromosomal break
