# Supplementary material for: Cytogenetic and Transcriptomic Analysis of Human Endometrial MSC Retaining Proliferative Activity after Sublethal Heat Shock
Source: Cells. 2018 Oct 25;7(11):184. doi: 10.3390/cells7110184 (PMC6262560; doi:10.3390/cells7110184)
Supplement: Supplementary file 1 [file cells-07-00184-s001.zip › cells-363926-supp/Supplementary Table 3.pdf]

Supplementary Table 3. DNA recombination and repair related gene modules enriching for genes significantly deregulated in SHS-SP vs. control MSC.

[*]GO module or BioSystems pathway accession number.

[**]GO module or BioSystems pathway name.

[@] Average fold difference of expression between SHS-SP vs. control MSC.

[#] Gene number in a gene module.

[♦]p-value for pathway enrichment of tested gene sample.

[♥] q-value for pathway enrichment of tested gene sample (please see footnote).

[&] Genes are ranked according to the effect strength (induction for induced, inhibition for inhibited).

Pathway* number	Pathway name**	Fold difference@	Gene number #	p-value♦	q-value♥	Gene Symbols&
**DNA repair related gene modules that are induced in SHS-SP vs. control MSC**						
GO:0006289	Nucleotide-excision repair	2.841	74	1.15E-04	0.07	PARP1; POLR2A; COPS6; SUMO3; RAD23A; RFC1; POLR2H; POLL; POLR2J; ERCC2; UBE2I; UBE2V2; POLD3; COPS3; POLE; COPS8; NEIL3; POLR2K; CHD1L; PNKP; HMGN1; SUMO2; COPS2; RPA2; TCEA1; SUMO1; POLR2F; ERCC3; RAD23B; POLR2E; RBBP8; TP53; MMS19; RPA1; POLR2L; PRPF19; PCNA; CETN2; RPS27A; GPS1; CUL4A; UBA52; POLR2I; COPS7A; UBB; UBC; POLR2D; NEIL2; POLD2; USP7; CUL4B; DDB1; XAB2; GTF2H5; UBE2N; RBX1; RFC2; POLR2C; COPS4; GTF2H3; COPS7B; POLD4; COPS5; ATXN3; HUS1; SLX4; ERCC4; ISY1; SIRT1; AQR; GTF2H4; CCNH; POLK; CDK7
GO:0006281	DNA repair	1.87	273	1.18E-04	0.05	PARP1; POLR2A; SFPQ; PPP5C; RUVBL2; COPS6; SUMO3; UFD1; KIF22; RAD23A; HELB; UBE2L6; RFC1; POLR2H; TRIP12; USP47; POLL; UBE2A; POLR2J; CIB1; ERCC2; UBE2I; TRIM25; UBE2V2; FOXM1; UPF1; POLD3; COPS3; MC1R; USP28; POLE; COPS8; SEM1; NEIL3; TP53BP1; CHAF1B; POLR2K; XRCC4; TDG; ASCC2; PNKP; CHD1L; TDP1; MRNIP; NEXMIF; CDC45; REV1; KDM2A; HMGN1; UHRF1; RMI2; INO80C; PAGR1; NSMCE1; CENPS; DTL; GINS2; SUMO2; FANCI; COPS2; ALKBH1; PALB2; RECQL4; NCOA6; RPA2; WDR48; FAAP20; RPAIN; YY1; ABRAXAS1; TCEA1; HELQ; SUMO1; EYA2; SETD2; MEN1; EYA4; FBXO18; HMGB2; TOPBP1; POLR2F; SMC1A; ERCC3; RUVBL1; NUDT1; RAD21; POLM; ALKBH5; UBE2V1; ASCC1; RHNO1; FAAP100; PTTG1; ATRX; MUS81; TTC5; RBM14; SWI5; AP5S1; MUM1; SSRP1; SMC3; GEN1; POLG;

						RAD23B; NUCKS1; POLR2E; WRNIP1; TRIP13; MORF4L1; IGHMBP2; RBBP8; UBR5; INTS3; TP53; DCLRE1C; SUPT16H; MMS19; UIMC1; UCHL5; TFPT; NABP2; KAT5; RPA1; POLR2L; PRPF19; PCNA; OTUB1; CETN2; RPS27A; XRCC5; GPS1; CUL4A; SMC5; UBE2B; UBA52; ISG15; RPS27L; POLR2I; HUWE1; HSF1; ZBTB1; BABAM1; APEX1; HMGB1; XRCC6; RAD54L; NONO; PIAS4; COPS7A; CDCA5; PRKDC; UBB; RPS3; UBC; TREX1; H2AFX; TOP3A; MORF4L2; VCP; POLR2D; NEIL2; NPM1; HMGA1; USP1; POLD2; USP7; PCLAF; DDX1; CUL4B; BAZ1B; TAOK1; DDB1; SFR1; XAB2; PARPBP; GTF2H5; UBE2N; RBX1; DOT1L; POLH; XRCC2; MPG; RFC2; POLR2C; MCRS1; TRIM28; TONSL; DTX3L; SMG1; HERC2; COPS4; MAD2L2; SETMAR; GTF2H3; COPS7B; USP10; FTO; BRIP1; POLD4; KIN; RAD1; MMS22L; FBXO6; PSMD14; COPS5; ANKLE1; UBE2T; BRCA1; FANCF; SWSAP1; CLSPN; ATXN3; TDP2; GINS4; AP5Z1; HUS1; FAAP24; FANCG; FEN1; ATR; INO80E; INIP; RAD54B; ZSWIM7; RAD9A; MTA1; SLX4; RNF168; HMGA2; ERCC4; MSH3; ATM; NPLOC4; CHEK1; ISY1; MCMDC2; NHEJ1; SETX; STUB1; RAD17; CENPX; CEP164; APBB1; NSMCE2; SIRT1; TNKS1BP1; CHRNA4; AQR; GADD45A; NSD2; FZR1; RAD18; RNF8; GTF2H4; NSMCE3; PARP4; CCNH; MARF1; USP43; UBE2D3; APLF; REV3L; SMARCB1; POLK; ZFYVE26; CHAF1A; APTX; RNF138; PDS5A; REC8; CDK7; MLH3
GO:0006284	Base-excision repair	6.09	19	1.04E-03	0.05	USP47; POLL; POLE; NEIL3; TDG; RECQL4; RPA2; POLG; TP53; RPA1; HUWE1; APEX1; NEIL2; HMGA1; MPG; HMGA2; HMGA1; DNA2; NEIL1
GO:0051106	DNA ligation involved in DNA repair	27.7	5	1.24E-03	0.08	PARP1; XRCC4; HMGB2; HMGB1; LIG4
1309112__REACTOME__R-HSA-5696399	Global Genome Nucleotide Excision Repair (GG-NER)	4.19	56	1.26E-03	0.08	PARP1; COPS6; POLE4; SUMO3; RAD23A; RFC1; ERCC2; UBE2I; UBE2V2; POLD3; COPS3; POLE; COPS8; CHD1L; INO80C; SUMO2; COPS2; RPA2; YY1; SUMO1; ERCC3; RUVBL1; RAD23B; TFPT; RPA1; PCNA; POLE3; CETN2; RPS27A; GPS1; ACTB; CUL4A; UBA52; PIAS3; COPS7A; UBB; UBC; POLD2; CUL4B; DDB1; GTF2H5; UBE2N; RBX1; RFC2; MCRS1; COPS4; GTF2H3; COPS7B; POLD4; COPS5; INO80E; ERCC4; GTF2H4; CCNH; POLK; CDK7
1309111__REACTOME__R-HSA-5696398	Nucleotide Excision Repair	3.31	73	1.31E-03	0.06	PARP1; POLR2A; COPS6; POLE4; SUMO3; RAD23A; RFC1; POLR2H; POLR2J; ERCC2; UBE2I; UBE2V2; POLD3; COPS3; POLE; COPS8; POLR2K; CHD1L; HMGN1; INO80C; SUMO2; COPS2; RPA2; YY1;

						TCEA1; SUMO1; POLR2F; ERCC3; RUVBL1; RAD23B; POLR2E; TFPT; RPA1; POLR2L; PRPF19; PCNA; POLE3; CETN2; RPS27A; GPS1; ACTB; CUL4A; UBA52; PIAS3; POLR2I; COPS7A; UBB; UBC; POLR2D; POLD2; USP7; CUL4B; DDB1; XAB2; GTF2H5; UBE2N; RBX1; RFC2; POLR2C; MCRS1; COPS4; GTF2H3; COPS7B; POLD4; COPS5; INO80E; ERCC4; ISY1; AQR; GTF2H4; CCNH; POLK; CDK7
1270350__REACTOME__R-HSA-73894	DNA Repair	2.15	164	1.36E-03	0.07	PARP1; POLR2A; PPP5C; COPS6; POLE4; SUMO3; UFD1; RAD23A; UBE2L6; RFC1; POLR2H; PPP4C; POLL; POLR2J; ERCC2; UBE2I; TRIM25; UBE2V2; POLD3; COPS3; POLE; COPS8; TP53BP1; POLR2K; TDG; XRCC4; ASCC2; PNKP; CHD1L; TDP1; REV1; HMGN1; RMI2; INO80C; CENPS; DTL; BAP1; SUMO2; FANCI; COPS2; PALB2; RPA2; FAAP20; YY1; ABRAXAS1; TCEA1; SUMO1; EYA2; EYA4; TOPBP1; POLR2F; ERCC3; RNF4; RUVBL1; POLM; ALKBH5; ASCC1; RHNO1; FAAP100; MUS81; KPNA2; GEN1; RAD23B; POLR2E; RBBP8; TP53; DCLRE1C; UIMC1; TFPT; KAT5; RPA1; POLR2L; PRPF19; PCNA; POLE3; CETN2; RPS27A; XRCC5; GPS1; ACTB; CUL4A; UBE2B; UBA52; ISG15; PIAS3; POLR2I; CCNA2; BABAM1; APEX1; XRCC6; PIAS4; COPS7A; PRKDC; UBB; UBC; H2AFX; TOP3A; VCP; POLR2D; NEIL2; TIMELESS; USP1; POLD2; USP7; PCLAF; CUL4B; BAZ1B; DDB1; XAB2; GTF2H5; UBE2N; RBX1; POLH; XRCC2; MPG; RFC2; POLR2C; MCRS1; HERC2; COPS4; MAD2L2; GTF2H3; KDM4B; COPS7B; USP10; FTO; BRIP1; POLD4; RAD1; COPS5; UBE2T; BRCA1; FANCF; CLSPN; TDP2; HUS1; FAAP24; FANCG; FEN1; ATR; INO80E; RAD9A; SLX4; RNF168; ERCC4; MSH3; ATM; NPLOC4; CHEK1; ISY1; NHEJ1; RAD17; CENPX; APBB1; AQR; NSD2; RAD18; RNF8; GTF2H4; CCNH; USP43; REV3L; POLK; CDK7
1270351__REACTOME__R-HSA-73884	Base Excision Repair; Resolution of Abasic Sites (AP sites)	6.22	18	1.42E-03	0.07	PARP1; POLE4; RFC1; POLD3; POLE; TDG; PNKP; RPA2; RPA1; PCNA; POLE3; APEX1; NEIL2; POLD2; MPG; RFC2; POLD4; FEN1
1268744__REACTOME__R-HSA-3108214	SUMOylation of DNA damage response and repair proteins	2.93	47	1.54E-03	0.07	PARP1; SUMO3; RING1; PML; PHC1; UBE2I; POM121C; TPR; TDG; XRCC4; RANBP2; NUP153; CDKN2A; NSMCE1; SCMH1; SUMO2; NUP85; PHC2; NDC1; SUMO1; NUP88; SMC1A; NUP98; RAD21; SMC3; NUP62; RPA1; CETN2; SMC5; PIAS4; NUP58; HDAC7; NUP93; HERC2; PIAS2; PHC3; CBX4; BRCA1; RNF168; NSMCE2; NUP35; SEH1L; NUP50; NSMCE3; NUP210; NUP188; CBX8
83043__KEGG__hsa03410	Base excision repair	4.24	17	1.62E-03	0.08	PARP1; POLE4; POLL; POLD3; POLE; NEIL3; TDG; PCNA; POLE3; APEX1;

						HMGB1; NEIL2; POLD2; MPG; POLD4; FEN1; PARP4
83047__KEGG __hsa03450	Non-homologous end-joining	2.92	11	1.73E-03	0.08	POLL; XRCC4; DNTT; POLM; DCLRE1C; XRCC5; XRCC6; PRKDC; FEN1; NHEJ1; LIG4
**DNA recombination and repair related gene modules that are inhibited in SHS-SP vs control**						
GO:0016444	Somatic cell DNA recombination	-4.96	19	1.21E-03	0.06	EXOSC3; RNF8; RAG2; ATM; EXO1; MLH11; HSPD1; EXOSC6; RNF168; AICDA; TCF3; BCL11B; CCR6; HSPD1; SWAP70; PRKDC; HMGB1; DCLRE1C; HMGB2
GO:0007131	Reciprocal meiotic recombination	-4.35	21	2.21E-03	0.07	MLH3; REC8; CENPX; MCMDC2; ATM; ERCC4; SLX4; KLHDC3; RAD54B; BRIP1; XRCC2; TOP2A; UBE2B; TOP2B; TRIP13; MUS81; RAD21; CENPS; DMC1; MRE11A; MEIOB
GO:0035825	Reciprocal DNA recombination	-4.35	21	2.21E-03	0.07	MLH3; REC8; CENPX; MCMDC2; ATM; ERCC4; SLX4; KLHDC3; RAD54B; BRIP1; XRCC2; TOP2A; UBE2B; TOP2B; TRIP13; MUS81; RAD21; CENPS; DMC1; MRE11A; MEIOB
GO:0045911	Positive regulation of DNA recombination	-4.89	13	2.29E-03	0.07	EXOSC3; IL2; NSD2; TFRC; TNFSF4; EXOSC6; TGFB1; UBE2B; STAT6; MRNIP; IL4; WHSC1; TNFSF13
GO:0015074	DNA integration	-11.28	7	2.65E-03	0.09	SMARCB1; NYNRIN; SETMAR; BANF1; RLF; ZBED9; THAP9
GO:0010792	DNA double-strand break processing involved in repair via single-strand annealing	-12.06	5	3.89E-03	0.09	RNF138; SLX4; RBBP8; RAD52; SLX1B
GO:0045002	double-strand break repair via single-strand annealing	-12.06	5	3.89E-03	0.09	RNF138; SLX4; RBBP8; RAD52; SLX1B
1309097__REACTOME__R-HSA-5693548	Sensing of DNA Double Strand Breaks	-2.46	5	4.21E-03	0.09	ATM; KAT5; KPNA2; MRE11A; RAD50
198890__WikiPathways__WP186	Homologous recombination	-1.85	8	6.32E-03	0.09	ATM; RAD54B; POLD4; POLD2; RPA1; POLD3; MRE11A; RAD54L; MUS81; NBN; EME1
homolog_DNArep_NC'14_Down	Homologous recombination DNA repair signature (Peng et al 2014)	-1.48	71	3.28E-03	0.06	SHCBP1; CSE1L; SUV39H1; CHAF1A; AURKB; AADAT; NCAPD3; HELLS; GJB2; TK1; KIF11; FDPS; CHEK1; HMGB3; ANLN; RAD54B; DEPDC1; FEN1; MTBP; CHRNA5; AHSA1; GINS4; BRCA1; POLA2; E2F2; KIF14; C1QTNF6; EFHD2; DHFR; MSX1; TIMELESS; WDHD1; CDCA5; MCM2; RAD54L; CDCA3; CCNA2; TMEM158; DPYSL3; TYMS; PCNA; RRM2; MRTO4; DNMT1; CTSC; TRIP13; POLR3K; DUT; TACC3; RECQL4; SLC25A10; MCM3; CCNB1; FANCI; GINS2; INSIG1; UHRF1; C1orf112; PRPF38A; CHAF1B; NEIL3; MCM5; TUBA4A; NETO2; MCM7; SFPQ
1108230__KEGG__M00691	DNA damage-induced cell cycle checkpoints	-4.92	5	7.63E-03	0.09	CHEK1; ATM; ATR; CDC25C; CDC25A

Footnote. We used q<0.1. In the literature q-value up to 0.15 is used (e.g., Seemann et al. 2012; Koo et al. 2014; Riester et al. 2014; Morow et al. 2015; Vazquez-Martin et al. 2016; Coarfa et al. 2017).

Coarfa C., et al. 2017. Sexual dimorphism of the pulmonary transcriptome in neonatal hyperoxic lung injury: identification of angiogenesis as a key pathway. Am. J. Physiol. 313: L991-L1005.

Koo I. et al. 2014. Comparative analysis of false discovery rate methods in constructing metabolic association networks. J Bioinform. Comput. Biol. 12: 1450018.

Morrow J.D. et al. 2015. Identifying a gene expression signature of frequent COPD exacerbations in peripheral blood using network methods. BMC Med. Genomics. 8: 1.

Riester M. et al. 2014. Integrative analysis of 1q23.3 copy-number gain in metastatic urothelial carcinoma. Clin. Cancer Res. 20: 1873-83.

Seemann S.E. et al. 2012. Transcripts with in silico predicted RNA structure are enriched everywhere in the mouse brain. BMC Genomics 13: 214.

Vazquez-Martin A. et al. 2016. Somatic polyploidy is associated with the upregulation of c-MYC interacting genes and EMT-like signature. Oncotarget 7: 75235–75260.
