# Supplementary material for: Cytogenetic and Transcriptomic Analysis of Human Endometrial MSC Retaining Proliferative Activity after Sublethal Heat Shock
Source: Cells. 2018 Oct 25;7(11):184. doi: 10.3390/cells7110184 (PMC6262560; doi:10.3390/cells7110184)
Supplement: Supplementary file 1 [file cells-07-00184-s001.zip › cells-363926-supp/Supplementary Table 4.pdf]

Supplementary Table 4. The pathways enriched in switched-on DNA repair genes.

GeneN - the observed number of genes belonging to a pathway in the switched-on genes.
Ratio - the ratio of observed to expected numbers of genes belonging to a pathway in the switched-on genes.

Pathway ID	Pathway Name	GeneN	Ratio	p-value	q-value	Gene list
1309111__REACTOME__R-HSA-5696398	Nucleotide Excision Repair	34	29.87	1.69E-43	2.60E-40	PARP1; POLR2A; COPS6; POLE4; SUMO3; RAD23A; RFC1; POLR2H; POLR2J; ERCC2; UBE2I; UBE2V2; POLD3; COPS3; POLE; COPS8; POLR2K; CHD1L; HMGN1; SUMO2; COPS2; RPA2; YY1; TCEA1; SUMO1; POLR2F; ERCC3; RUVBL1; TFPT; RPA1; PCNA; PIAS3; CCNH; POLK
1268743__REACTOME__R-HSA-3108232	SUMO E3 ligases SUMOylate target proteins	29	27.35	1.26E-35	9.70E-33	PARP1; SUMO3; RING1; PML; PHC1; UBE2I; POM121C; TP53BP1; TPR; TDG; XRCC4; RANBP2; NUP153; CDKN2A; SCMH1; SUMO2; NUP85; PHC2; NDC1; SUMO1; NUP88; SMC1A; RAD21; SMC3; NUP62; RPA1; PCNA; PIAS3; PIAS2
1268744__REACTOME__R-HSA-3108214	SUMOylation of DNA damage response and repair proteins	26	34.73	1.59E-35	9.80E-33	PARP1; SUMO3; RING1; PML; PHC1; UBE2I; POM121C; TPR; TDG; XRCC4; RANBP2; NUP153; CDKN2A; SCMH1; SUMO2; NUP85; PHC2; NDC1; SUMO1; NUP88; SMC1A; RAD21; SMC3; NUP62; RPA1; PIAS2
1309112__REACTOME__R-HSA-5696399	Global Genome Nucleotide Excision Repair (GG-NER)	27	30.92	4.95E-35	2.54E-32	PARP1; COPS6; POLE4; SUMO3; RAD23A; RFC1; ERCC2; UBE2I; UBE2V2; POLD3; COPS3; POLE; COPS8; CHD1L; SUMO2; COPS2; RPA2; YY1; SUMO1; ERCC3; RUVBL1; TFPT; RPA1; PCNA; PIAS3; CCNH; POLK
1268738__REACTOME__R-HSA-2990846	SUMOylation	29	25.83	9.99E-35	4.40E-32	PARP1; SUMO3; RING1; PML; PHC1; UBE2I; POM121C; TP53BP1; TPR; TDG; XRCC4; RANBP2; NUP153; CDKN2A; SCMH1; SUMO2; NUP85; PHC2; NDC1; SUMO1; NUP88; SMC1A; RAD21; SMC3; NUP62; RPA1; PCNA; PIAS3; PIAS2
1309117__REACTOME__R-HSA-6781827	Transcription-Coupled Nucleotide Excision Repair (TC-NER)	22	26.12	1.46E-26	5.01E-24	POLR2A; COPS6; POLE4; RFC1; POLR2H; POLR2J; ERCC2; POLD3; COPS3; POLE; COPS8; POLR2K; HMGN1; COPS2; RPA2; TCEA1; POLR2F; ERCC3; RPA1; PCNA; CCNH; POLK
1269741__REACTOME__R-HSA-1640170	Cell Cycle	41	7.34	3.74E-25	1.15E-22	RUVBL2; POLE4; RFC1; MCM7; TUBA4A; MCM5; UBE2I; UBE2V2; FOXM1; POM121C; POLD3; CDC25C; POLE; TP53BP1; TPR; RANBP2; NUP153; CDKN2A; CDC45; RMI2; MCM3; NUP85; RPA2; NDC1; SUMO1; NUP88; TOPBP1; SMC1A; RAD21; RUVBL1; RHNO1; PTTG1; SMC3; NUP62; RBBP8; UIMC1; KAT5; RPA1; PCNA; CDCA5; CCNH
1309120__REACTOME__R-HSA-6782210	Gap-filling DNA repair synthesis and ligation in TC-NER	18	26.23	6.57E-22	1.56E-19	POLR2A; POLE4; RFC1; POLR2H; POLR2J; ERCC2; POLD3; POLE; POLR2K; HMGN1; RPA2; TCEA1; POLR2F; ERCC3; RPA1; PCNA; CCNH; POLK
1309119__REACTOME__R-HSA-6782135	Dual incision in TC-NER	18	25.65	1.08E-21	2.38E-19	POLR2A; POLE4; RFC1; POLR2H; POLR2J; ERCC2; POLD3; POLE; POLR2K; HMGN1; RPA2; TCEA1; POLR2F; ERCC3; RPA1; PCNA; CCNH; POLK

1339112__REACTOME__R-HSA-4570464	SUMOylation of RNA binding proteins	15	33.17	2.23E-20	4.31E-18	RING1; PHC1; UBE2I; POM121C; TPR; RANBP2; NUP153; SCMH1; SUMO2; NUP85; PHC2; NDC1; SUMO1; NUP88; NUP62
1309114__REACTOME__R-HSA-5696395	Formation of Incision Complex in GG-NER	14	29.92	2.92E-18	5.30E-16	PARP1; SUMO3; RAD23A; ERCC2; UBE2I; UBE2V2; CHD1L; SUMO2; RPA2; SUMO1; ERCC3; RPA1; PIAS3; CCNH
1339113__REACTOME__R-HSA-4615885	SUMOylation of DNA replication proteins	14	28.96	5.26E-18	9.01E-16	SUMO3; UBE2I; POM121C; TPR; RANBP2; NUP153; SUMO2; NUP85; NDC1; SUMO1; NUP88; NUP62; PCNA; PIAS3
1269058__REACTOME__R-HSA-162587	HIV Life Cycle	20	12.95	2.52E-17	4.09E-15	POLR2A; POLR2H; POLR2J; ERCC2; POM121C; POLR2K; TPR; XRCC4; RANBP2; NUP153; NUP85; NDC1; TCEA1; NUP88; POLR2F; ERCC3; SSRP1; NUP62; SUPT16H; CCNH
1269117__REACTOME__R-HSA-168325	Viral Messenger RNA Synthesis	13	29.77	5.41E-17	8.16E-15	POLR2A; POLR2H; POLR2J; POM121C; POLR2K; TPR; RANBP2; NUP153; NUP85; NDC1; NUP88; POLR2F; NUP62
1269069__REACTOME__R-HSA-162599	Late Phase of HIV Life Cycle	19	13.69	5.56E-17	8.16E-15	POLR2A; POLR2H; POLR2J; ERCC2; POM121C; POLR2K; TPR; RANBP2; NUP153; NUP85; NDC1; TCEA1; NUP88; POLR2F; ERCC3; SSRP1; NUP62; SUPT16H; CCNH
470351__KEGG__hsa_M00427; 890636__KEGG__M00427	Nuclear pore complex	12	34.98	7.03E-17	9.87E-15	SUMO3; UBE2I; POM121C; TPR; RANBP2; NUP153; SUMO2; NUP85; NDC1; SUMO1; NUP88; NUP62
1309101__REACTOME__R-HSA-5693607	Processing of DNA double-strand break ends	15	20.91	1.18E-16	1.58E-14	PPP4C; UBE2I; UBE2V2; TP53BP1; RMI2; SUMO2; RPA2; SUMO1; TOPBP1; RNF4; RHNO1; RBBP8; UIMC1; KAT5; RPA1
1309118__REACTOME__R-HSA-6781823	Formation of TC-NER Pre-Incision Complex	14	21.90	6.13E-16	7.56E-14	POLR2A; COPS6; POLR2H; POLR2J; ERCC2; COPS3; COPS8; POLR2K; HMGN1; COPS2; TCEA1; POLR2F; ERCC3; CCNH
1269763__REACTOME__R-HSA-69278	Cell Cycle, Mitotic	29	6.30	6.84E-16	8.11E-14	POLE4; RFC1; MCM7; TUBA4A; MCM5; FOXM1; POM121C; POLD3; CDC25C; POLE; TPR; RANBP2; NUP153; CDKN2A; CDC45; MCM3; NUP85; RPA2; NDC1; NUP88; SMC1A; RAD21; PTTG1; SMC3; NUP62; RPA1; PCNA; CDCA5; CCNH
1268701__REACTOME__R-HSA-597592	Post-translational protein modification	39	4.30	1.36E-15	1.56E-13	PARP1; SUMO3; RING1; RAD23A; PML; USP47; UBE2A; PHC1; UBE2I; TRIM25; POM121C; USP28; TP53BP1; TPR; TDG; XRCC4; RANBP2; NUP153; CDKN2A; SCMH1; BAP1; SUMO2; NUP85; PHC2; NDC1; YY1; SUMO1; NUP88; SMC1A; RAD21; RUVBL1; SMC3; NUP62; UIMC1; TFPT; RPA1; PCNA; PIAS3; PIAS2
1269723__REACTOME__R-HSA-5578749	Transcriptional regulation by small RNAs	13	23.82	1.95E-15	2.15E-13	POLR2A; POLR2H; POLR2J; POM121C; POLR2K; TPR; RANBP2; NUP153; NUP85; NDC1; NUP88; POLR2F; NUP62
1309115__REACTOME__R-HSA-5696400	Dual Incision in GG-NER	12	27.48	3.06E-15	3.25E-13	PARP1; POLE4; RFC1; ERCC2; POLD3; POLE; CHD1L; RPA2; ERCC3; RPA1; PCNA; POLK
1270374__REACTOME__R-HSA-5656169	Termination of translesion DNA synthesis	11	29.39	1.82E-14	1.88E-12	POLE4; UBE2L6; RFC1; TRIM25; POLD3; POLE; REV1; RPA2; RPA1; PCNA; POLK

1270351__REACTOME__R-HSA-73884; 1270359__REACTOME__R-HSA-73933	Base Excision Repair; Resolution of Abasic Sites (AP sites)	10	35.62	2.37E-14	2.36E-12	PARP1; POLE4; RFC1; POLD3; POLE; TDG; PNKP; RPA2; RPA1; PCNA
452__KEGG__ko03420; 83044__KEGG__hsa03420	Nucleotide excision repair	11	27.13	5.50E-14	5.14E-12	POLE4; RAD23A; RFC1; ERCC2; POLD3; POLE; RPA2; ERCC3; RPA1; PCNA; CCNH
1269777__REACTOME__R-HSA-69242	S Phase	16	12.07	1.53E-13	1.39E-11	POLE4; RFC1; MCM7; MCM5; POLD3; POLE; CDC45; MCM3; RPA2; SMC1A; RAD21; SMC3; RPA1; PCNA; CDCA5; CCNH
1269719__REACTOME__R-HSA-211000	Gene Silencing by RNA	13	17.74	1.58E-13	1.40E-11	POLR2A; POLR2H; POLR2J; POM121C; POLR2K; TPR; RANBP2; NUP153; NUP85; NDC1; NUP88; POLR2F; NUP62
1270367__REACTOME__R-HSA-73893	DNA Damage Bypass	12	20.80	1.65E-13	1.42E-11	POLE4; UBE2L6; RFC1; TRIM25; POLD3; POLE; REV1; DTL; RPA2; RPA1; PCNA; POLK
444__KEGG__ko03030; 83039__KEGG__hsa03030	DNA replication	10	30.53	1.84E-13	1.53E-11	POLE4; RFC1; MCM7; MCM5; POLD3; POLE; MCM3; RPA2; RPA1; PCNA
1269654__REACTOME__R-HSA-3700989	Transcriptional Regulation by TP53	24	6.39	2.28E-13	1.85E-11	POLR2A; PML; POLR2H; POLR2J; ERCC2; CDC25C; POLR2K; CDKN2A; RMI2; FANCI; RPA2; TCEA1; TOPBP1; POLR2F; ERCC3; RHNO1; TTC5; SSRP1; RBBP8; SUPT16H; KAT5; RPA1; PCNA; CCNH
1270369__REACTOME__R-HSA-110313	Translesion synthesis by Y family DNA polymerases bypasses lesions on DNA template	11	24.32	2.37E-13	1.87E-11	POLE4; UBE2L6; RFC1; TRIM25; POLD3; POLE; REV1; RPA2; RPA1; PCNA; POLK
1269057__REACTOME__R-HSA-162906	HIV Infection	20	8.07	3.43E-13	2.65E-11	POLR2A; POLR2H; POLR2J; ERCC2; POM121C; POLR2K; TPR; XRCC4; RANBP2; NUP153; NUP85; NDC1; TCEA1; NUP88; POLR2F; ERCC3; SSRP1; NUP62; SUPT16H; CCNH
1269857__REACTOME__R-HSA-180786	Extension of Telomeres	9	33.95	9.05E-13	6.81E-11	RUVBL2; POLE4; RFC1; POLD3; POLE; RPA2; RUVBL1; RPA1; PCNA
1269074__REACTOME__R-HSA-167169; 1269076__REACTOME__R-HSA-167246; 1269077__REACTOME__R-HSA-167200	HIV Transcription Elongation; Tat-mediated elongation of the HIV-1 transcript; Formation of HIV-1 elongation complex containing HIV-1 Tat	11	21.37	1.26E-12	9.22E-11	POLR2A; POLR2H; POLR2J; ERCC2; POLR2K; TCEA1; POLR2F; ERCC3; SSRP1; SUPT16H; CCNH
1383075__REACTOME__R-HSA-6796648	TP53 Regulates Transcription of DNA Repair Genes	12	17.49	1.72E-12	1.20E-10	POLR2A; POLR2H; POLR2J; ERCC2; POLR2K; FANCI; TCEA1; POLR2F; ERCC3; SSRP1; SUPT16H; CCNH

1269080__REACTOME__R-HSA-167152	Formation of HIV elongation complex in the absence of HIV Tat	11	20.75	1.83E-12	1.26E-10	POLR2A; POLR2H; POLR2J; ERCC2; POLR2K; TCEA1; POLR2F; ERCC3; SSRP1; SUPT16H; CCNH
1269754__REACTOME__R-HSA-69473	G2/M DNA damage checkpoint	12	17.10	2.31E-12	1.55E-10	UBE2V2; CDC25C; TP53BP1; RMI2; RPA2; SUMO1; TOPBP1; RHNO1; RBBP8; UIMC1; KAT5; RPA1
198771__WikiPathways__WP466	DNA Replication	10	24.66	2.59E-12	1.70E-10	RFC1; MCM7; MCM5; POLD3; POLE; CDC45; MCM3; RPA2; RPA1; PCNA
1269753__REACTOME__R-HSA-69481	G2/M Checkpoints	16	10.06	2.96E-12	1.90E-10	MCM7; MCM5; UBE2V2; CDC25C; TP53BP1; CDC45; RMI2; MCM3; RPA2; SUMO1; TOPBP1; RHNO1; RBBP8; UIMC1; KAT5; RPA1
1269784__REACTOME__R-HSA-69190	DNA strand elongation	9	30.37	3.35E-12	2.11E-10	RFC1; MCM7; MCM5; POLD3; CDC45; MCM3; RPA2; RPA1; PCNA
1270364__REACTOME__R-HSA-110373	Resolution of AP sites via the multiple-nucleotide patch replacement pathway	8	36.64	7.84E-12	4.74E-10	PARP1; POLE4; RFC1; POLD3; POLE; RPA2; RPA1; PCNA
1269682__REACTOME__R-HSA-75955; 1269684__REACTOME__R-HSA-112382	RNA Polymerase II Transcription Elongation; Formation of RNA Pol II elongation complex	11	17.20	1.85E-11	1.10E-09	POLR2A; POLR2H; POLR2J; ERCC2; POLR2K; TCEA1; POLR2F; ERCC3; SSRP1; SUPT16H; CCNH
1309116__REACTOME__R-HSA-5696397	Gap-filling DNA repair synthesis and ligation in GG-NER	8	32.06	3.27E-11	1.87E-09	POLE4; RFC1; POLD3; POLE; RPA2; RPA1; PCNA; POLK
1269922__REACTOME__R-HSA-170822	Regulation of Glucokinase by Glucokinase Regulatory Protein	8	32.06	3.27E-11	1.87E-09	POM121C; TPR; RANBP2; NUP153; NUP85; NDC1; NUP88; NUP62
1309098__REACTOME__R-HSA-5693565	Recruitment and ATM-mediated phosphorylation of repair and signaling proteins at DNA double strand breaks	10	19.43	4.11E-11	2.31E-09	PPP5C; UBE2I; UBE2V2; TP53BP1; BAP1; SUMO1; EYA2; EYA4; UIMC1; KAT5
1269742__REACTOME__R-HSA-69620	Cell Cycle Checkpoints	16	8.48	4.35E-11	2.40E-09	MCM7; MCM5; UBE2V2; CDC25C; TP53BP1; CDC45; RMI2; MCM3; RPA2; SUMO1; TOPBP1; RHNO1; RBBP8; UIMC1; KAT5; RPA1
1269856__REACTOME__R-HSA-157579	Telomere Maintenance	9	24.05	4.44E-11	2.40E-09	RUVBL2; POLE4; RFC1; POLD3; POLE; RPA2; RUVBL1; RPA1; PCNA
1309096__REACTOME__R-HSA-5693606	DNA Double Strand Break Response	10	18.86	5.75E-11	3.06E-09	PPP5C; UBE2I; UBE2V2; TP53BP1; BAP1; SUMO1; EYA2; EYA4; UIMC1; KAT5

1268677__REACTOME__R-HSA-392499	Metabolism of proteins	42	2.91	5.85E-11	3.06E-09	PARP1; SUMO3; RING1; RAD23A; UBE2L6; PML; USP47; UBE2A; TUBA4A; PHC1; UBE2I; TRIM25; POM121C; USP28; TP53BP1; TPR; TDG; XRCC4; RANBP2; NUP153; CDKN2A; SCMH1; BAP1; SUMO2; NUP85; PHC2; NDC1; YY1; SUMO1; NUP88; SMC1A; RAD21; RUVBL1; SMC3; NUP62; UIMC1; TFPT; RPA1; PCNA; PIAS3; PIAS2; FBXO6
451__KEGG__ko03410; 83043__KEGG__hsa03410	Base excision repair	8	30.18	6.10E-11	3.14E-09	PARP1; POLE4; POLL; POLD3; POLE; NEIL3; TDG; PCNA
198917__WikiPathways__WP405	Eukaryotic Transcription Initiation	9	23.08	6.85E-11	3.46E-09	POLR2A; POLR2H; POLR2J; ERCC2; POLR2K; POLR2F; ERCC3; POLR3K; CCNH
1269650__REACTOME__R-HSA-212436	Generic Transcription Pathway	29	3.98	8.61E-11	4.29E-09	PARP1; POLR2A; PML; POLR2H; POLR2J; ERCC2; UBE2I; CDC25C; POLR2K; CDKN2A; RMI2; FANCI; RPA2; YY1; TCEA1; SUMO1; MEN1; TOPBP1; POLR2F; ERCC3; RHNO1; TTC5; SSRP1; RBBP8; SUPT16H; KAT5; RPA1; PCNA; CCNH
1269070__REACTOME__R-HSA-167172	Transcription of the HIV genome	11	15.01	9.40E-11	4.60E-09	POLR2A; POLR2H; POLR2J; ERCC2; POLR2K; TCEA1; POLR2F; ERCC3; SSRP1; SUPT16H; CCNH
1269123__REACTOME__R-HSA-168333	NEP/NS2 Interacts with the Cellular Export Machinery	8	28.50	1.08E-10	5.07E-09	POM121C; TPR; RANBP2; NUP153; NUP85; NDC1; NUP88; NUP62
1269114__REACTOME__R-HSA-168271	Transport of Ribonucleoproteins into the Host Nucleus	8	28.50	1.08E-10	5.07E-09	POM121C; TPR; RANBP2; NUP153; NUP85; NDC1; NUP88; NUP62
1269121__REACTOME__R-HSA-168274	Export of Viral Ribonucleoproteins from Nucleus	8	28.50	1.08E-10	5.07E-09	POM121C; TPR; RANBP2; NUP153; NUP85; NDC1; NUP88; NUP62
1309113__REACTOME__R-HSA-5696394	DNA Damage Recognition in GG-NER	9	20.61	2.23E-10	1.01E-08	PARP1; COPS6; RAD23A; COPS3; COPS8; COPS2; YY1; RUVBL1; TFPT
1270366__REACTOME__R-HSA-5651801	PCNA-Dependent Long Patch Base Excision Repair	7	34.53	3.03E-10	1.22E-08	POLE4; RFC1; POLD3; POLE; RPA2; RPA1; PCNA
1269859__REACTOME__R-HSA-174417	Telomere C-strand (Lagging Strand) Synthesis	7	34.53	3.03E-10	1.22E-08	POLE4; RFC1; POLD3; POLE; RPA2; RPA1; PCNA
1339110__REACTOME__R-HSA-3232118	SUMOylation of transcription factors	7	34.53	3.03E-10	1.22E-08	SUMO3; UBE2I; TP53BP1; CDKN2A; SUMO2; SUMO1; PIAS3
1269817__REACTOME__R-HSA-3301854	Nuclear Pore Complex (NPC) Disassembly	8	25.65	3.04E-10	1.22E-08	POM121C; TPR; RANBP2; NUP153; NUP85; NDC1; NUP88; NUP62
1269773__REACTOME__R-HSA-68962	Activation of the pre-replicative complex	8	25.65	3.04E-10	1.22E-08	POLE4; MCM7; MCM5; POLE; CDC45; MCM3; RPA2; RPA1

1269073__REACTOME__R-HSA-167160; 1269681__REACTOME__R-HSA-77075	RNA Pol II CTD phosphorylation and interaction with CE during HIV infection; RNA Pol II CTD phosphorylation and interaction with CE	8	25.65	3.04E-10	1.22E-08	POLR2A; POLR2H; POLR2J; ERCC2; POLR2K; POLR2F; ERCC3; CCNH
1269094__REACTOME__R-HSA-180910	Vpr-mediated nuclear import of PICs	8	25.65	3.04E-10	1.22E-08	POM121C; TPR; RANBP2; NUP153; NUP85; NDC1; NUP88; NUP62
1269096__REACTOME__R-HSA-180746	Nuclear import of Rev protein	8	25.65	3.04E-10	1.22E-08	POM121C; TPR; RANBP2; NUP153; NUP85; NDC1; NUP88; NUP62
1269697__REACTOME__R-HSA-159227	Transport of the SLBP independent Mature mRNA	8	25.65	3.04E-10	1.22E-08	POM121C; TPR; RANBP2; NUP153; NUP85; NDC1; NUP88; NUP62
175229__KEGG__ko03013; 177876__KEGG__hsa03013	RNA transport	14	8.98	3.54E-10	1.40E-08	SUMO3; UBE2I; UPF1; POM121C; TPR; RANBP2; NUP153; SUMO2; NUP85; NDC1; SUMO1; NUP88; TACC3; NUP62
1269676__REACTOME__R-HSA-674695	RNA Polymerase II Pre-transcription Events	11	13.06	4.71E-10	1.82E-08	POLR2A; POLR2H; POLR2J; ERCC2; POLR2K; TCEA1; POLR2F; ERCC3; SSRP1; SUPT16H; CCNH
1269085__REACTOME__R-HSA-165054	Rev-mediated nuclear export of HIV RNA	8	24.43	4.85E-10	1.82E-08	POM121C; TPR; RANBP2; NUP153; NUP85; NDC1; NUP88; NUP62
1269687__REACTOME__R-HSA-72086	mRNA Capping	8	24.43	4.85E-10	1.82E-08	POLR2A; POLR2H; POLR2J; ERCC2; POLR2K; POLR2F; ERCC3; CCNH
1269696__REACTOME__R-HSA-159230	Transport of the SLBP Dependant Mature mRNA	8	24.43	4.85E-10	1.82E-08	POM121C; TPR; RANBP2; NUP153; NUP85; NDC1; NUP88; NUP62
198805__WikiPathways__WP45	G1 to S cell cycle control	10	14.57	9.53E-10	3.54E-08	MCM7; MCM5; POLE; CDKN2A; CDC45; MCM3; RPA2; RPA1; PCNA; CCNH
1269092__REACTOME__R-HSA-176033	Interactions of Vpr with host cellular proteins	8	22.30	1.14E-09	4.03E-08	POM121C; TPR; RANBP2; NUP153; NUP85; NDC1; NUP88; NUP62
1270368__REACTOME__R-HSA-110314	Recognition of DNA damage by PCNA-containing replication complex	8	22.30	1.14E-09	4.03E-08	POLE4; RFC1; POLD3; POLE; DTL; RPA2; RPA1; PCNA
1269131__REACTOME__R-HSA-168276	NS1 Mediated Effects on Host Pathways	8	22.30	1.14E-09	4.03E-08	POM121C; TPR; RANBP2; NUP153; NUP85; NDC1; NUP88; NUP62
1269095__REACTOME__R-HSA-177243	Interactions of Rev with host cellular proteins	8	22.30	1.14E-09	4.03E-08	POM121C; TPR; RANBP2; NUP153; NUP85; NDC1; NUP88; NUP62

1269649__REACTOME__R-HSA-74160	Gene Expression	43	2.60	1.15E-09	4.03E-08	PARP1; POLR2A; PML; POLR2H; POLR2J; ERCC2; UBE2I; UPF1; POM121C; CDC25C; POLR2K; TPR; TDG; PRPF38A; RANBP2; NUP153; CDKN2A; UHRF1; RMI2; FANCI; NUP85; RPA2; NDC1; YY1; TCEA1; SUMO1; MEN1; NUP88; TOPBP1; POLR2F; ERCC3; RHNO1; TTC5; POLR3K; SSRP1; NUP62; RBBP8; INTS3; SUPT16H; KAT5; RPA1; PCNA; CCNH
1269853__REACTOME__R-HSA-73886	Chromosome Maintenance	9	17.49	1.16E-09	4.03E-08	RUVBL2; POLE4; RFC1; POLD3; POLE; RPA2; RUVBL1; RPA1; PCNA
920977__WikiPathways__WP2446	Retinoblastoma (RB) in Cancer	11	11.95	1.29E-09	4.42E-08	MCM7; POLD3; POLE; CDC45; MCM3; RPA2; HMGB2; SMC1A; SMC3; RPA1; PCNA
1269698__REACTOME__R-HSA-159231	Transport of Mature mRNA Derived from an Intronless Transcript	8	21.37	1.68E-09	5.71E-08	POM121C; TPR; RANBP2; NUP153; NUP85; NDC1; NUP88; NUP62
1270425__REACTOME__R-HSA-3371453	Regulation of HSF1-mediated heat shock response	10	13.64	1.91E-09	6.41E-08	POM121C; TPR; RANBP2; NUP153; NUP85; RPA2; NDC1; NUP88; NUP62; RPA1
1269316__REACTOME__R-HSA-1169410; 1269317__REACTOME__R-HSA-1169408	Antiviral mechanism by IFN-stimulated genes; ISG15 antiviral mechanism	10	13.36	2.38E-09	7.77E-08	UBE2L6; TRIM25; POM121C; TPR; RANBP2; NUP153; NUP85; NDC1; NUP88; NUP62
1269082__REACTOME__R-HSA-167243; 1269083__REACTOME__R-HSA-167238	Tat-mediated HIV elongation arrest and recovery; Pausing and recovery of Tat-mediated HIV elongation	8	20.52	2.44E-09	7.77E-08	POLR2A; POLR2H; POLR2J; POLR2K; TCEA1; POLR2F; SSRP1; SUPT16H
1269130__REACTOME__R-HSA-168253	Host Interactions with Influenza Factors	8	20.52	2.44E-09	7.77E-08	POM121C; TPR; RANBP2; NUP153; NUP85; NDC1; NUP88; NUP62
1269921__REACTOME__R-HSA-70153	Glucose transport	8	20.52	2.44E-09	7.77E-08	POM121C; TPR; RANBP2; NUP153; NUP85; NDC1; NUP88; NUP62
1269695__REACTOME__R-HSA-159234	Transport of Mature mRNAs Derived from Intronless Transcripts	8	20.52	2.44E-09	7.77E-08	POM121C; TPR; RANBP2; NUP153; NUP85; NDC1; NUP88; NUP62
463__KEGG__ko04110; 83054__KEGG__hsa04110	Cell cycle	12	9.62	3.05E-09	9.50E-08	MCM7; MCM5; CDC25C; CDKN2A; CDC45; MCM3; SMC1A; RAD21; PTTG1; SMC3; PCNA; CCNH

1269075__REACTOME__R-HSA-167158; 1269683__REACTOME__R-HSA-113418	Formation of the HIV-1 Early Elongation Complex; Formation of the Early Elongation Complex	8	19.73	3.48E-09	1.06E-07	POLR2A; POLR2H; POLR2J; ERCC2; POLR2K; POLR2F; ERCC3; CCNH
1269079__REACTOME__R-HSA-167287; 1269081__REACTOME__R-HSA-167290	HIV elongation arrest and recovery; Pausing and recovery of HIV elongation	8	19.73	3.48E-09	1.06E-07	POLR2A; POLR2H; POLR2J; POLR2K; TCEA1; POLR2F; SSRP1; SUPT16H
198811__WikiPathways__WP179	Cell Cycle	11	10.85	3.81E-09	1.15E-07	MCM7; MCM5; CDC25C; CDKN2A; CDC45; MCM3; MEN1; SMC1A; PTTG1; PCNA; CCNH
1269920__REACTOME__R-HSA-189200	Hexose transport	8	18.32	6.75E-09	2.00E-07	POM121C; TPR; RANBP2; NUP153; NUP85; NDC1; NUP88; NUP62
1309110__REACTOME__R-HSA-5693571	Nonhomologous End-Joining (NHEJ)	8	17.69	9.20E-09	2.70E-07	POLL; UBE2V2; TP53BP1; XRCC4; TDP1; SUMO1; UIMC1; KAT5
1269779__REACTOME__R-HSA-69239	Synthesis of DNA	11	9.66	1.36E-08	3.96E-07	POLE4; RFC1; MCM7; MCM5; POLD3; POLE; CDC45; MCM3; RPA2; RPA1; PCNA
1269056__REACTOME__R-HSA-5663205	Infectious disease	20	4.48	1.48E-08	4.26E-07	POLR2A; POLR2H; POLR2J; ERCC2; POM121C; POLR2K; TPR; XRCC4; RANBP2; NUP153; NUP85; NDC1; TCEA1; NUP88; POLR2F; ERCC3; SSRP1; NUP62; SUPT16H; CCNH
1269731__REACTOME__R-HSA-194441; 1269732__REACTOME__R-HSA-191859	Metabolism of non-coding RNA; snRNP Assembly	8	16.55	1.65E-08	4.71E-07	POM121C; TPR; RANBP2; NUP153; NUP85; NDC1; NUP88; NUP62
1270372__REACTOME__R-HSA-5655862	Translesion synthesis by POLK	6	29.59	2.01E-08	5.70E-07	RFC1; REV1; RPA2; RPA1; PCNA; POLK
1309089__REACTOME__R-HSA-6784531	tRNA processing in the nucleus	8	16.03	2.17E-08	6.03E-07	POM121C; TPR; RANBP2; NUP153; NUP85; NDC1; NUP88; NUP62

1269071__REACTOME__R-HSA-167161; 1269072__REACTOME__R-HSA-167162; 1269677__REACTOME__R-HSA-73779; 1269678__REACTOME__R-HSA-76042; 1269679__REACTOME__R-HSA-75953; 1269680__REACTOME__R-HSA-73776	HIV Transcription Initiation; RNA Polymerase II HIV Promoter Escape; RNA Polymerase II Transcription Pre-Initiation And Promoter Opening; RNA Polymerase II Transcription Initiation And Promoter Clearance; RNA Polymerase II Transcription Initiation; RNA Polymerase II Promoter Escape	8	16.03	2.17E-08	6.03E-07	POLR2A; POLR2H; POLR2J; ERCC2; POLR2K; POLR2F; ERCC3; CCNH
1269115__REACTOME__R-HSA-168273	Influenza Viral RNA Transcription and Replication	13	7.25	2.24E-08	6.18E-07	POLR2A; POLR2H; POLR2J; POM121C; POLR2K; TPR; RANBP2; NUP153; NUP85; NDC1; NUP88; POLR2F; NUP62
1269875__REACTOME__R-HSA-69306	DNA Replication	11	9.16	2.43E-08	6.62E-07	POLE4; RFC1; MCM7; MCM5; POLD3; POLE; CDC45; MCM3; RPA2; RPA1; PCNA
1270421__REACTOME__R-HSA-3371556	Cellular response to heat stress	10	10.51	2.77E-08	7.49E-07	POM121C; TPR; RANBP2; NUP153; NUP85; RPA2; NDC1; NUP88; NUP62; RPA1
1269815__REACTOME__R-HSA-2980766	Nuclear Envelope Breakdown	8	15.09	3.65E-08	9.78E-07	POM121C; TPR; RANBP2; NUP153; NUP85; NDC1; NUP88; NUP62
1309108__REACTOME__R-HSA-5685938	HDR through Single Strand Annealing (SSA)	7	19.52	3.81E-08	1.01E-06	RMI2; RPA2; TOPBP1; RHNO1; RBBP8; KAT5; RPA1
309__KEGG__ko00240; 82946__KEGG__hsa00240	Pyrimidine metabolism	10	10.18	3.82E-08	1.01E-06	POLR2A; POLE4; POLR2H; POLR2J; POLD3; POLE; POLR2K; POLR2F; DUT; POLR3K
1269109__REACTOME__R-HSA-168255	Influenza Life Cycle	13	6.78	5.07E-08	1.32E-06	POLR2A; POLR2H; POLR2J; POM121C; POLR2K; TPR; RANBP2; NUP153; NUP85; NDC1; NUP88; POLR2F; NUP62
1269311__REACTOME__R-HSA-913531	Interferon Signaling	12	7.54	5.11E-08	1.32E-06	UBE2L6; PML; TRIM25; POM121C; TPR; RANBP2; NUP153; NUP85; NDC1; SUMO1; NUP88; NUP62
1269757__REACTOME__R-HSA-176187	Activation of ATR in response to replication stress	7	18.70	5.30E-08	1.35E-06	MCM7; MCM5; CDC25C; CDC45; MCM3; RPA2; RPA1
1309104__REACTOME__R-HSA-5693616	Presynaptic phase of homologous DNA pairing and strand exchange	7	17.95	7.27E-08	1.84E-06	RMI2; RPA2; TOPBP1; RHNO1; RBBP8; KAT5; RPA1

1383076__REACTOME__R-HSA-5633007	Regulation of TP53 Activity	12	7.26	7.90E-08	1.98E-06	PML; CDKN2A; RMI2; RPA2; TOPBP1; RHNO1; TTC5; SSRP1; RBBP8; SUPT16H; KAT5; RPA1
1309103__REACTOME__R-HSA-5693579	Homologous DNA Pairing and Strand Exchange	7	17.26	9.82E-08	2.43E-06	RMI2; RPA2; TOPBP1; RHNO1; RBBP8; KAT5; RPA1
1269108__REACTOME__R-HSA-168254	Influenza Infection	13	6.41	9.85E-08	2.43E-06	POLR2A; POLR2H; POLR2J; POM121C; POLR2K; TPR; RANBP2; NUP153; NUP85; NDC1; NUP88; POLR2F; NUP62
377128__KEGG__ko03460; 377262__KEGG__hsa03460	Fanconi anemia pathway	7	16.62	1.31E-07	3.18E-06	REV1; RMI2; FANCI; RPA2; FAAP100; RPA1; POLK
1269788__REACTOME__R-HSA-69186	Lagging Strand Synthesis	5	32.06	2.02E-07	4.88E-06	RFC1; POLD3; RPA2; RPA1; PCNA
1269688__REACTOME__R-HSA-72203	Processing of Capped Intron-Containing Pre-mRNA	14	5.51	2.11E-07	5.04E-06	POLR2A; POLR2H; POLR2J; POM121C; POLR2K; TPR; PRPF38A; RANBP2; NUP153; NUP85; NDC1; NUP88; POLR2F; NUP62
1339139__REACTOME__R-HSA-5619507; 1339140__REACTOME__R-HSA-5617472	Activation of HOX genes during differentiation; Activation of anterior HOX genes in hindbrain development during early embryogenesis	8	12.21	2.13E-07	5.07E-06	POLR2A; POLR2H; POLR2J; POLR2K; PAGR1; YY1; POLR2F; PIAS2
1269793__REACTOME__R-HSA-2468052	Establishment of Sister Chromatid Cohesion	4	51.30	2.79E-07	6.58E-06	SMC1A; RAD21; SMC3; CDCA5
442__KEGG__ko03020; 83037__KEGG__hsa03020	RNA polymerase	6	20.25	2.95E-07	6.89E-06	POLR2A; POLR2H; POLR2J; POLR2K; POLR2F; POLR3K
1383080__REACTOME__R-HSA-6804756	Regulation of TP53 Activity through Phosphorylation	9	9.62	3.13E-07	7.25E-06	RMI2; RPA2; TOPBP1; RHNO1; SSRP1; RBBP8; SUPT16H; KAT5; RPA1
1269675__REACTOME__R-HSA-73857	RNA Polymerase II Transcription	11	7.12	3.42E-07	7.88E-06	POLR2A; POLR2H; POLR2J; ERCC2; POLR2K; TCEA1; POLR2F; ERCC3; SSRP1; SUPT16H; CCNH
453__KEGG__ko03430; 83045__KEGG__hsa03430	Mismatch repair	5	29.15	3.66E-07	8.31E-06	RFC1; POLD3; RPA2; RPA1; PCNA
413415__KEGG__hsa_M00180; 890456__KEGG__M00180	RNA polymerase II, eukaryotes	5	29.15	3.66E-07	8.31E-06	POLR2A; POLR2H; POLR2J; POLR2K; POLR2F
1269694__REACTOME__R-HSA-159236	Transport of Mature mRNA derived from an Intron-Containing Transcript	8	11.40	3.75E-07	8.44E-06	POM121C; TPR; RANBP2; NUP153; NUP85; NDC1; NUP88; NUP62

1269764__REACTOME__R-HSA-453279	Mitotic G1-G1/S phases	11	7.05	3.79E-07	8.48E-06	POLE4; MCM7; MCM5; POLE; CDKN2A; CDC45; MCM3; RPA2; RPA1; PCNA; CCNH
1270373__REACTOME__R-HSA-5656121	Translesion synthesis by POLI	5	26.72	6.20E-07	1.37E-05	RFC1; REV1; RPA2; RPA1; PCNA
1270370__REACTOME__R-HSA-110312	Translesion synthesis by REV1	5	26.72	6.20E-07	1.37E-05	RFC1; REV1; RPA2; RPA1; PCNA
1269768__REACTOME__R-HSA-69206	G1/S Transition	10	7.54	7.00E-07	1.53E-05	POLE4; MCM7; MCM5; POLE; CDC45; MCM3; RPA2; RPA1; PCNA; CCNH
1269865__REACTOME__R-HSA-1500620	Meiosis	7	13.20	7.24E-07	1.57E-05	UBE2I; RPA2; SMC1A; RAD21; SMC3; RBBP8; RPA1
1269661__REACTOME__R-HSA-73772	RNA Polymerase I Promoter Escape	6	17.49	7.80E-07	1.67E-05	POLR2H; ERCC2; POLR2K; POLR2F; ERCC3; CCNH
1269663__REACTOME__R-HSA-73863	RNA Polymerase I Transcription Termination	6	17.49	7.80E-07	1.67E-05	POLR2H; ERCC2; POLR2K; POLR2F; ERCC3; CCNH
1270412__REACTOME__R-HSA-5358565	Mismatch repair (MMR) directed by MSH2:MSH6 (MutSalpha)	4	42.75	8.27E-07	1.75E-05	POLD3; RPA2; RPA1; PCNA
1268742__REACTOME__R-HSA-3065678	SUMO is transferred from E1 to E2 (UBE2I, UBC9)	4	42.75	8.27E-07	1.75E-05	SUMO3; UBE2I; SUMO2; SUMO1
1269693__REACTOME__R-HSA-72202	Transport of Mature Transcript to Cytoplasm	8	10.06	1.02E-06	2.15E-05	POM121C; TPR; RANBP2; NUP153; NUP85; NDC1; NUP88; NUP62
1383045__REACTOME__R-HSA-8851708	Signaling by FGFR2 IIIa TM	5	22.90	1.53E-06	3.15E-05	POLR2A; POLR2H; POLR2J; POLR2K; POLR2F
1269724__REACTOME__R-HSA-5601884	PIWI-interacting RNA (piRNA) biogenesis	5	22.90	1.53E-06	3.15E-05	POLR2A; POLR2H; POLR2J; POLR2K; POLR2F
1270413__REACTOME__R-HSA-5358606	Mismatch repair (MMR) directed by MSH2:MSH3 (MutSbeta)	4	36.64	1.91E-06	3.82E-05	POLD3; RPA2; RPA1; PCNA
1269863__REACTOME__R-HSA-174437	Removal of the Flap Intermediate from the C-strand	4	36.64	1.91E-06	3.82E-05	POLD3; RPA2; RPA1; PCNA
1270411__REACTOME__R-HSA-5358508	Mismatch Repair	4	36.64	1.91E-06	3.82E-05	POLD3; RPA2; RPA1; PCNA
1269862__REACTOME__R-HSA-174414	Processive synthesis on the C-strand of the telomere	4	36.64	1.91E-06	3.82E-05	POLD3; RPA2; RPA1; PCNA

454__KEGG__ko03440; 83046__KEGG__hsa03440	Homologous recombination	6	14.80	2.29E-06	4.55E-05	POLD3; RPA2; TOPBP1; RBBP8; UIMC1; RPA1
1269810__REACTOME__R-HSA-68886	M Phase	13	4.85	2.52E-06	4.98E-05	POM121C; TPR; RANBP2; NUP153; NUP85; NDC1; NUP88; SMC1A; RAD21; PTTG1; SMC3; NUP62; CDCA5
1270414__REACTOME__R-HSA-2262752	Cellular responses to stress	16	3.92	2.82E-06	5.54E-05	RING1; PHC1; POM121C; TPR; RANBP2; NUP153; CDKN2A; SCMH1; NUP85; PHC2; RPA2; NDC1; NUP88; NUP62; KAT5; RPA1
1269662__REACTOME__R-HSA-73777	RNA Polymerase I Chain Elongation	6	14.25	2.90E-06	5.67E-05	POLR2H; ERCC2; POLR2K; POLR2F; ERCC3; CCNH
1309088__REACTOME__R-HSA-72306	tRNA processing	8	8.69	3.21E-06	6.23E-05	POM121C; TPR; RANBP2; NUP153; NUP85; NDC1; NUP88; NUP62
1269734__REACTOME__R-HSA-212165	Epigenetic regulation of gene expression	8	8.55	3.66E-06	7.05E-05	POLR2H; ERCC2; POLR2K; TDG; UHRF1; POLR2F; ERCC3; CCNH
198875__WikiPathways__WP531	Mismatch repair	3	64.12	3.71E-06	7.09E-05	RFC1; RPA1; PCNA
1269790__REACTOME__R-HSA-69166	Removal of the Flap Intermediate	4	32.06	3.77E-06	7.09E-05	POLD3; RPA2; RPA1; PCNA
1268739__REACTOME__R-HSA-3215018	Processing and activation of SUMO	4	32.06	3.77E-06	7.09E-05	SUMO3; UBE2I; SUMO2; SUMO1
1269789__REACTOME__R-HSA-69183	Processive synthesis on the lagging strand	4	32.06	3.77E-06	7.09E-05	POLD3; RPA2; RPA1; PCNA
1269720__REACTOME__R-HSA-203927	MicroRNA (miRNA) biogenesis	5	18.86	4.55E-06	8.51E-05	POLR2A; POLR2H; POLR2J; POLR2K; POLR2F
1269831__REACTOME__R-HSA-68874; 1269832__REACTOME__R-HSA-69002	M/G1 Transition; DNA Replication Pre-Initiation	8	8.02	6.00E-06	1.12E-04	POLE4; MCM7; MCM5; POLE; CDC45; MCM3; RPA2; RPA1
138029__Pathway Interaction Database__ranbp2pathway	Sumoylation by RanBP2 regulates transcriptional repression	4	28.50	6.70E-06	1.23E-04	UBE2I; RANBP2; SUMO1; PIAS2
1269785__REACTOME__R-HSA-176974	Unwinding of DNA	4	28.50	6.70E-06	1.23E-04	MCM7; MCM5; CDC45; MCM3
1269811__REACTOME__R-HSA-68875	Mitotic Prophase	8	7.89	6.76E-06	1.23E-04	POM121C; TPR; RANBP2; NUP153; NUP85; NDC1; NUP88; NUP62
1268870__REACTOME__R-HSA-1839126	FGFR2 mutant receptor activation	5	16.87	8.34E-06	1.51E-04	POLR2A; POLR2H; POLR2J; POLR2K; POLR2F

1269078__REACTOME__R-HSA-167242	Abortive elongation of HIV-1 transcript in the absence of Tat	5	16.87	8.34E-06	1.51E-04	POLR2A; POLR2H; POLR2J; POLR2K; POLR2F
1309121__REACTOME__R-HSA-6783310	Fanconi Anemia Pathway	5	16.03	1.10E-05	1.94E-04	FANCI; RPA2; FAAP20; FAAP100; RPA1
1383022__REACTOME__R-HSA-6803529	FGFR2 alternative splicing	5	16.03	1.10E-05	1.94E-04	POLR2A; POLR2H; POLR2J; POLR2K; POLR2F
413416__KEGG__hsa_M00181; 890457__KEGG__M00181	RNA polymerase III, eukaryotes	4	25.65	1.10E-05	1.94E-04	POLR2H; POLR2K; POLR2F; POLR3K
1269660__REACTOME__R-HSA-73762	RNA Polymerase I Transcription Initiation	6	11.32	1.21E-05	2.10E-04	POLR2H; ERCC2; POLR2K; POLR2F; ERCC3; CCNH
1458202__WikiPathways__WP3878	ATM Signaling Network in Development and Disease	6	10.99	1.44E-05	2.49E-04	PPP5C; TP53BP1; HMGN1; SMC1A; RBBP8; KAT5
1269739__REACTOME__R-HSA-427413	NoRC negatively regulates rRNA expression	6	10.69	1.70E-05	2.93E-04	POLR2H; ERCC2; POLR2K; POLR2F; ERCC3; CCNH
1269670__REACTOME__R-HSA-73780	RNA Polymerase III Chain Elongation	4	23.32	1.71E-05	2.94E-04	POLR2H; POLR2K; POLR2F; POLR3K
1269737__REACTOME__R-HSA-5250941	Negative epigenetic regulation of rRNA expression	6	10.12	2.35E-05	4.01E-04	POLR2H; ERCC2; POLR2K; POLR2F; ERCC3; CCNH
307__KEGG__ko00230; 82944__KEGG__hsa00230	Purine metabolism	9	5.77	2.38E-05	4.03E-04	POLR2A; POLE4; POLR2H; POLR2J; POLD3; POLE; POLR2K; POLR2F; POLR3K
1268869__REACTOME__R-HSA-5655253	Signaling by FGFR2 in disease	5	12.82	3.53E-05	5.93E-04	POLR2A; POLR2H; POLR2J; POLR2K; POLR2F
1270371__REACTOME__R-HSA-110320	Translesion Synthesis by POLH	4	19.73	3.62E-05	5.95E-04	RFC1; RPA2; RPA1; PCNA
1269671__REACTOME__R-HSA-73980	RNA Polymerase III Transcription Termination	4	19.73	3.62E-05	5.95E-04	POLR2H; POLR2K; POLR2F; POLR3K
1268741__REACTOME__R-HSA-3065676	SUMO is conjugated to E1 (UBA2:SAE1)	3	38.47	3.62E-05	5.95E-04	SUMO3; SUMO2; SUMO1
1268740__REACTOME__R-HSA-3065679	SUMO is proteolytically processed	3	38.47	3.62E-05	5.95E-04	SUMO3; SUMO2; SUMO1
1269658__REACTOME__R-HSA-73854	RNA Polymerase I Promoter Clearance	6	9.38	3.69E-05	6.02E-04	POLR2H; ERCC2; POLR2K; POLR2F; ERCC3; CCNH

1269656__REACTOME__R-HSA-504046	RNA Polymerase I, RNA Polymerase III, and Mitochondrial Transcription	7	7.48	3.71E-05	6.03E-04	POLR2H; ERCC2; POLR2K; POLR2F; ERCC3; POLR3K; CCNH
1383088__REACTOME__R-HSA-6807505	RNA polymerase II transcribes snRNA genes	6	9.16	4.25E-05	6.86E-04	POLR2A; POLR2H; POLR2J; POLR2K; POLR2F; INTS3
1270346__REACTOME__R-HSA-452723	Transcriptional regulation of pluripotent stem cells	5	12.33	4.32E-05	6.95E-04	POLR2A; POLR2H; POLR2J; POLR2K; POLR2F
1269657__REACTOME__R-HSA-73864	RNA Polymerase I Transcription	6	8.95	4.87E-05	7.79E-04	POLR2H; ERCC2; POLR2K; POLR2F; ERCC3; CCNH
466__KEGG__ko04120; 83056__KEGG__hsa04120	Ubiquitin mediated proteolysis	8	5.90	5.87E-05	9.34E-04	UBE2L6; PML; TRIP12; UBE2A; UBE2I; UBR5; PIAS3; PIAS2
1268854__REACTOME__R-HSA-1643685	Disease	21	2.54	6.61E-05	1.04E-03	POLR2A; POLR2H; POLR2J; ERCC2; POM121C; CDC25C; POLR2K; TPR; XRCC4; RANBP2; NUP153; NUP85; NDC1; TCEA1; NUP88; POLR2F; ERCC3; SSRP1; NUP62; SUPT16H; CCNH
1269907__REACTOME__R-HSA-425407	SLC-mediated transmembrane transport	9	5.06	6.75E-05	1.06E-03	POM121C; TPR; RANBP2; NUP153; SLC25A10; NUP85; NDC1; NUP88; NUP62
413421__KEGG__hsa_M00285; 890547__KEGG__M00285	MCM complex	3	32.06	7.16E-05	1.11E-03	MCM7; MCM5; MCM3
1269830__REACTOME__R-HSA-2470946	Cohesin Loading onto Chromatin	3	32.06	7.16E-05	1.11E-03	SMC1A; RAD21; SMC3
1269667__REACTOME__R-HSA-76061	RNA Polymerase III Transcription Initiation From Type 1 Promoter	4	16.03	8.89E-05	1.36E-03	POLR2H; POLR2K; POLR2F; POLR3K
1269669__REACTOME__R-HSA-76071	RNA Polymerase III Transcription Initiation From Type 3 Promoter	4	16.03	8.89E-05	1.36E-03	POLR2H; POLR2K; POLR2F; POLR3K
1269668__REACTOME__R-HSA-76066	RNA Polymerase III Transcription Initiation From Type 2 Promoter	4	16.03	8.89E-05	1.36E-03	POLR2H; POLR2K; POLR2F; POLR3K
920989__WikiPathways__WP1530	miRNA Regulation of DNA Damage Response	6	8.02	9.20E-05	1.40E-03	RFC1; PML; MCM7; CDC25C; RPA2; SMC1A
1269091__REACTOME__R-HSA-162909	Host Interactions of HIV factors	8	5.52	9.46E-05	1.43E-03	POM121C; TPR; RANBP2; NUP153; NUP85; NDC1; NUP88; NUP62

1269786__REACTOME__R-HSA-69109; 1269787__REACTOME__R-HSA-69091; 1269861__REACTOME__R-HSA-174411	Leading Strand Synthesis; Polymerase switching; Polymerase switching on the C-strand of the telomere	3	27.48	1.24E-04	1.86E-03	RFC1; POLD3; PCNA
413425__KEGG__hsa_M00290; 890551__KEGG__M00290	Holo-TFIIH complex	3	27.48	1.24E-04	1.86E-03	ERCC2; ERCC3; CCNH
1268863__REACTOME__R-HSA-1226099	Signaling by FGFR in disease	5	9.16	1.91E-04	2.84E-03	POLR2A; POLR2H; POLR2J; POLR2K; POLR2F
1269821__REACTOME__R-HSA-2500257	Resolution of Sister Chromatid Cohesion	6	6.87	2.20E-04	3.25E-03	RANBP2; NUP85; SMC1A; RAD21; SMC3; CDCA5
1269666__REACTOME__R-HSA-76046	RNA Polymerase III Transcription Initiation	4	12.82	2.26E-04	3.31E-03	POLR2H; POLR2K; POLR2F; POLR3K
413423__KEGG__hsa_M00288; 468376__KEGG__M00288	RPA complex	2	64.12	2.41E-04	3.53E-03	RPA2; RPA1
1269867__REACTOME__R-HSA-1221632	Meiotic synapsis	4	12.21	2.75E-04	4.01E-03	UBE2I; SMC1A; RAD21; SMC3
413479__KEGG__hsa_M00182; 890458__KEGG__M00182	RNA polymerase I, eukaryotes	3	21.37	2.91E-04	4.19E-03	POLR2H; POLR2K; POLR2F
1269829__REACTOME__R-HSA-68884	Mitotic Telophase/Cytokinesis	3	21.37	2.91E-04	4.19E-03	SMC1A; RAD21; SMC3
1269664__REACTOME__R-HSA-74158; 1269665__REACTOME__R-HSA-749476	RNA Polymerase III Transcription; RNA Polymerase III Abortive And Retractive Initiation	4	11.66	3.32E-04	4.77E-03	POLR2H; POLR2K; POLR2F; POLR3K
1269691__REACTOME__R-HSA-72165	mRNA Splicing - Minor Pathway	5	7.82	4.08E-04	5.83E-03	POLR2A; POLR2H; POLR2J; POLR2K; POLR2F
1269820__REACTOME__R-HSA-68877	Mitotic Prometaphase	6	6.11	4.21E-04	5.98E-03	RANBP2; NUP85; SMC1A; RAD21; SMC3; CDCA5
198806__WikiPathways__WP138	Androgen receptor signaling pathway	6	5.92	4.99E-04	7.03E-03	UBE2I; SUMO1; RNF4; KAT5; PIAS3; PIAS2
