# Supplementary material for: Cytogenetic and Transcriptomic Analysis of Human Endometrial MSC Retaining Proliferative Activity after Sublethal Heat Shock
Source: Cells. 2018 Oct 25;7(11):184. doi: 10.3390/cells7110184 (PMC6262560; doi:10.3390/cells7110184)
Supplement: Supplementary file 1 [file cells-07-00184-s001.zip › cells-363926-supp/Supplementary Table 5.pdf]

**Supplementary Table 5. The pathways enriched in switched-off DNA repair genes.**

GeneN - the observed number of genes belonging to a pathway in the switched-off genes.
Ratio - the ratio of observed to expected numbers of genes belonging to a pathway in the switched-off genes.

| Pathway ID | Pathway Name | GeneN | Ratio | p-value | q-value | Gene list |
|---|---|---|---|---|---|---|
| 1270350__REACTOME__R-HSA-73894 | DNA Repair | 24 | 14.55 | 4.26E-22 | 4.38E-19 | REV3L; NSD2; MRE11; FANCG; MUS81; RAD50; RAD9A; RNF8; BRCA2; AQR; RNF168; POLM; FEN1; RAD17; FAAP24; APBB1; CDK7; CHEK1; NBN; USP43; GTF2H4; PALB2; RAD18; CHEK2 |
| 1269741__REACTOME__R-HSA-1640170 | Cell Cycle | 28 | 8.11 | 8.53E-19 | 6.58E-16 | GINS4; NUP35; NSD2; MRE11; NUP210; REC8; GINS2; AURKB; RAD50; MCPH1; RAD9A; RNF8; BRCA2; PDS5A; MLH3; CEP164; RNF168; FEN1; RAD17; NUP50; NCAPD3; CDK7; CHEK1; SEH1L; NUP188; NBN; FZR1; CHEK2 |
| 1309095__REACTOME__R-HSA-5693532 | DNA Double-Strand Break Repair | 16 | 19.29 | 6.71E-17 | 3.45E-14 | NSD2; MRE11; MUS81; RAD50; RAD9A; RNF8; BRCA2; POLM; RNF168; FEN1; RAD17; APBB1; CHEK1; NBN; CHEK2; PALB2 |
| 1309099__REACTOME__R-HSA-5693538 | Homology Directed Repair | 13 | 20.43 | 3.12E-14 | 1.37E-11 | RNF168; FEN1; RAD17; NSD2; MRE11; MUS81; RAD50; CHEK1; RAD9A; RNF8; BRCA2; NBN; PALB2 |
| 1309100__REACTOME__R-HSA-5693567 | HDR through Homologous Recombination (HR) or Single Strand Annealing (SSA) | 12 | 19.44 | 6.11E-13 | 2.36E-10 | RNF168; RAD17; NSD2; MRE11; MUS81; RAD50; CHEK1; RAD9A; BRCA2; RNF8; NBN; PALB2 |
| 1268743__REACTOME__R-HSA-3108232 | SUMO E3 ligases SUMOylate target proteins | 12 | 18.30 | 1.31E-12 | 4.51E-10 | RNF168; NUP35; TOP2B; NUP50; NUP210; AURKB; NSMCE1; CBX8; MTA1; NUP188; SEH1L; NSMCE3 |
| 1268738__REACTOME__R-HSA-2990846 | SUMOylation | 12 | 17.28 | 2.69E-12 | 8.30E-10 | RNF168; NUP35; TOP2B; NUP50; NUP210; AURKB; NSMCE1; CBX8; MTA1; NUP188; SEH1L; NSMCE3 |
| 1269754__REACTOME__R-HSA-69473 | G2/M DNA damage checkpoint | 10 | 23.05 | 9.54E-12 | 2.68E-09 | RNF168; RAD17; NSD2; MRE11; CHEK1; RAD50; RAD9A; RNF8; NBN; CHEK2 |
| 1309103__REACTOME__R-HSA-5693579 | Homologous DNA Pairing and Strand Exchange | 8 | 31.91 | 7.13E-11 | 1.83E-08 | RAD50; CHEK1; RAD9A; RAD17; BRCA2; NBN; MRE11; PALB2 |
| 1309102__REACTOME__R-HSA-5685942 | HDR through Homologous Recombination (HRR) | 9 | 21.71 | 1.98E-10 | 4.70E-08 | RAD17; MRE11; MUS81; CHEK1; RAD50; RAD9A; BRCA2; NBN; PALB2 |
| 1309101__REACTOME__R-HSA-5693607 | Processing of DNA double-strand break ends | 9 | 20.29 | 3.78E-10 | 7.77E-08 | RNF168; RAD17; NSD2; MRE11; CHEK1; RAD50; RAD9A; RNF8; NBN |
| 1268744__REACTOME__R-HSA-3108214 | SUMOylation of DNA damage response and repair proteins | 9 | 19.44 | 5.66E-10 | 1.09E-07 | RNF168; NUP35; NUP50; NUP210; NSMCE1; CBX8; NUP188; SEH1L; NSMCE3 |

| | | | | | | |
|---|---|---|---|---|---|---|
| 1309098__REACTOME__R-HSA-5693565 | Recruitment and ATM-mediated phosphorylation of repair and signaling proteins at DNA double strand breaks | 8 | 25.14 | 6.00E-10 | 1.09E-07 | RNF168; NSD2; MRE11; APBB1; RAD50; RNF8; NBN; CHEK2 |
| 1309096__REACTOME__R-HSA-5693606 | DNA Double Strand Break Response | 8 | 24.40 | 7.79E-10 | 1.34E-07 | RNF168; NSD2; MRE11; APBB1; RAD50; RNF8; NBN; CHEK2 |
| 1458202__WikiPathways__WP3878 | ATM Signaling Network in Development and Disease | 8 | 23.70 | 1.00E-09 | 1.63E-07 | RNF168; MRE11; AURKB; CHEK1; RAD50; RNF8; NBN; CHEK2 |
| 1309104__REACTOME__R-HSA-5693616 | Presynaptic phase of homologous DNA pairing and strand exchange | 7 | 29.04 | 2.49E-09 | 3.84E-07 | RAD50; CHEK1; RAD9A; RAD17; BRCA2; NBN; MRE11 |
| 454__KEGG__ko03440; 83046__KEGG__hsa03440 | Homologous recombination | 7 | 27.92 | 3.38E-09 | 4.97E-07 | RAD50; BRCA2; RAD54L; NBN; MRE11; MUS81; PALB2 |
| 377128__KEGG__ko03460; 377262__KEGG__hsa03460 | Fanconi anemia pathway | 7 | 26.89 | 4.53E-09 | 6.35E-07 | WDR48; REV3L; BRCA2; FAAP24; FANCG; MUS81; PALB2 |
| 920969__WikiPathways__WP2516 | ATM Signaling Pathway | 7 | 25.03 | 7.84E-09 | 1.01E-06 | RAD50; CHEK1; RAD9A; NBN; MRE11; CHEK2; GADD45A |
| 1309110__REACTOME__R-HSA-5693571 | Nonhomologous End-Joining (NHEJ) | 7 | 25.03 | 7.84E-09 | 1.01E-06 | RNF168; POLM; RAD50; RNF8; NSD2; NBN; MRE11 |
| 198788__WikiPathways__WP707 | DNA Damage Response | 8 | 18.44 | 8.49E-09 | 1.05E-06 | RAD17; MRE11; GADD45A; CHEK1; RAD50; RAD9A; NBN; CHEK2 |
| 1309105__REACTOME__R-HSA-5693537 | Resolution of D-Loop Structures | 6 | 34.57 | 1.13E-08 | 1.29E-06 | RAD50; BRCA2; NBN; MRE11; MUS81; PALB2 |
| 1309106__REACTOME__R-HSA-5693568 | Resolution of D-loop Structures through Holliday Junction Intermediates | 6 | 34.57 | 1.13E-08 | 1.29E-06 | RAD50; BRCA2; NBN; MRE11; MUS81; PALB2 |
| 1339113__REACTOME__R-HSA-4615885 | SUMOylation of DNA replication proteins | 7 | 23.42 | 1.30E-08 | 1.43E-06 | SEH1L; NUP188; NUP35; TOP2B; NUP50; NUP210; AURKB |
| 920989__WikiPathways__WP1530 | miRNA Regulation of DNA Damage Response | 8 | 17.28 | 1.45E-08 | 1.55E-06 | RAD17; MRE11; GADD45A; CHEK1; RAD50; RAD9A; NBN; CHEK2 |
| 219801__WikiPathways__WP1984 | Integrated Breast Cancer Pathway | 10 | 10.69 | 2.51E-08 | 2.59E-06 | PTEN; SIRT1; RAD54L; MRE11; CDK7; GADD45A; CHEK1; RAD50; BRCA2; CHEK2 |
| 1269753__REACTOME__R-HSA-69481 | G2/M Checkpoints | 10 | 10.17 | 4.10E-08 | 3.95E-06 | RNF168; RAD17; NSD2; MRE11; CHEK1; RAD50; RAD9A; RNF8; NBN; CHEK2 |
| 1383080__REACTOME__R-HSA-6804756 | Regulation of TP53 Activity through Phosphorylation | 8 | 13.83 | 8.97E-08 | 8.39E-06 | RAD17; MRE11; AURKB; CHEK1; RAD50; RAD9A; NBN; CHEK2 |

| | | | | | | |
|---|---|---|---|---|---|---|
| 1269763__REACTOME__R-HSA-69278 | Cell Cycle, Mitotic | 15 | 5.27 | 1.08E-07 | 9.76E-06 | GINS4; FEN1; NUP35; NUP50; NCAPD3; NUP210; CDK7; GINS2; AURKB; SEH1L; MCPH1; NUP188; FZR1; PDS5A; CEP164 |
| 1309109__REACTOME__R-HSA-5685939 | HDR through MMEJ (alt-NHEJ) | 4 | 69.14 | 1.19E-07 | 1.05E-05 | RAD50; FEN1; NBN; MRE11 |
| 1309107__REACTOME__R-HSA-5693554 | Resolution of D-loop Structures through Synthesis-Dependent Strand Annealing (SDSA) | 5 | 34.57 | 2.05E-07 | 1.75E-05 | RAD50; BRCA2; NBN; MRE11; PALB2 |
| 1269742__REACTOME__R-HSA-69620 | Cell Cycle Checkpoints | 10 | 8.57 | 2.10E-07 | 1.75E-05 | RNF168; RAD17; NSD2; MRE11; CHEK1; RAD50; RAD9A; RNF8; NBN; CHEK2 |
| 1339112__REACTOME__R-HSA-4570464 | SUMOylation of RNA binding proteins | 6 | 21.46 | 2.65E-07 | 2.15E-05 | CBX8; SEH1L; NUP188; NUP35; NUP50; NUP210 |
| 1269922__REACTOME__R-HSA-170822 | Regulation of Glucokinase by Glucokinase Regulatory Protein | 5 | 32.41 | 2.96E-07 | 2.34E-05 | SEH1L; NUP188; NUP35; NUP50; NUP210 |
| 1269654__REACTOME__R-HSA-3700989 | Transcriptional Regulation by TP53 | 13 | 5.59 | 4.28E-07 | 3.22E-05 | PTEN; RAD17; MRE11; CDK7; AURKB; GADD45A; RAD50; CHEK1; RAD9A; NBN; GTF2H4; TNKS1BP1; CHEK2 |
| 1269123__REACTOME__R-HSA-168333 | NEP/NS2 Interacts with the Cellular Export Machinery | 5 | 28.81 | 5.72E-07 | 3.92E-05 | SEH1L; NUP188; NUP35; NUP50; NUP210 |
| 1269866__REACTOME__R-HSA-912446 | Meiotic recombination | 5 | 28.81 | 5.72E-07 | 3.92E-05 | RAD50; BRCA2; NBN; MRE11; MLH3 |
| 1269114__REACTOME__R-HSA-168271 | Transport of Ribonucleoproteins into the Host Nucleus | 5 | 28.81 | 5.72E-07 | 3.92E-05 | SEH1L; NUP188; NUP35; NUP50; NUP210 |
| 1269121__REACTOME__R-HSA-168274 | Export of Viral Ribonucleoproteins from Nucleus | 5 | 28.81 | 5.72E-07 | 3.92E-05 | SEH1L; NUP188; NUP35; NUP50; NUP210 |
| 1269865__REACTOME__R-HSA-1500620 | Meiosis | 6 | 18.30 | 7.23E-07 | 4.80E-05 | RAD50; BRCA2; NBN; MRE11; REC8; MLH3 |
| 413426__KEGG__hsa_M00291; 890552__KEGG__M00291 | MRN complex | 3 | 103.70 | 8.64E-07 | 5.56E-05 | RAD50; NBN; MRE11 |
| 1269817__REACTOME__R-HSA-3301854 | Nuclear Pore Complex (NPC) Disassembly | 5 | 25.93 | 1.02E-06 | 6.05E-05 | SEH1L; NUP188; NUP35; NUP50; NUP210 |
| 1269094__REACTOME__R-HSA-180910 | Vpr-mediated nuclear import of PICs | 5 | 25.93 | 1.02E-06 | 6.05E-05 | SEH1L; NUP188; NUP35; NUP50; NUP210 |
| 1269096__REACTOME__R-HSA-180746 | Nuclear import of Rev protein | 5 | 25.93 | 1.02E-06 | 6.05E-05 | SEH1L; NUP188; NUP35; NUP50; NUP210 |
| 1269697__REACTOME__R-HSA-159227 | Transport of the SLBP independent Mature mRNA | 5 | 25.93 | 1.02E-06 | 6.05E-05 | SEH1L; NUP188; NUP35; NUP50; NUP210 |

| | | | | | | |
|---|---|---|---|---|---|---|
| 1269085__REACTOME__R-HSA-165054 | Rev-mediated nuclear export of HIV RNA | 5 | 24.69 | 1.33E-06 | 7.45E-05 | SEH1L; NUP188; NUP35; NUP50; NUP210 |
| 1269696__REACTOME__R-HSA-159230 | Transport of the SLBP Dependant Mature mRNA | 5 | 24.69 | 1.33E-06 | 7.45E-05 | SEH1L; NUP188; NUP35; NUP50; NUP210 |
| 470351__KEGG__hsa_M00427; 890636__KEGG__M00427 | Nuclear pore complex | 5 | 23.57 | 1.70E-06 | 9.39E-05 | SEH1L; NUP188; NUP35; NUP50; NUP210 |
| 1269092__REACTOME__R-HSA-176033 | Interactions of Vpr with host cellular proteins | 5 | 22.54 | 2.16E-06 | 1.11E-04 | SEH1L; NUP188; NUP35; NUP50; NUP210 |
| 1269131__REACTOME__R-HSA-168276 | NS1 Mediated Effects on Host Pathways | 5 | 22.54 | 2.16E-06 | 1.11E-04 | SEH1L; NUP188; NUP35; NUP50; NUP210 |
| 1309108__REACTOME__R-HSA-5685938 | HDR through Single Strand Annealing (SSA) | 5 | 22.54 | 2.16E-06 | 1.11E-04 | RAD50; RAD9A; RAD17; NBN; MRE11 |
| 1269095__REACTOME__R-HSA-177243 | Interactions of Rev with host cellular proteins | 5 | 22.54 | 2.16E-06 | 1.11E-04 | SEH1L; NUP188; NUP35; NUP50; NUP210 |
| 198890__WikiPathways__WP186 | Homologous recombination | 4 | 37.71 | 2.52E-06 | 1.27E-04 | RAD50; BRCA2; NBN; MRE11 |
| 1269811__REACTOME__R-HSA-68875 | Mitotic Prophase | 7 | 11.17 | 2.65E-06 | 1.32E-04 | SEH1L; MCPH1; NUP188; NUP35; NUP50; NCAPD3; NUP210 |
| 1269698__REACTOME__R-HSA-159231 | Transport of Mature mRNA Derived from an Intronless Transcript | 5 | 21.60 | 2.71E-06 | 1.33E-04 | SEH1L; NUP188; NUP35; NUP50; NUP210 |
| 1269130__REACTOME__R-HSA-168253 | Host Interactions with Influenza Factors | 5 | 20.74 | 3.36E-06 | 1.57E-04 | SEH1L; NUP188; NUP35; NUP50; NUP210 |
| 1269921__REACTOME__R-HSA-70153 | Glucose transport | 5 | 20.74 | 3.36E-06 | 1.57E-04 | SEH1L; NUP188; NUP35; NUP50; NUP210 |
| 1269695__REACTOME__R-HSA-159234 | Transport of Mature mRNAs Derived from Intronless Transcripts | 5 | 20.74 | 3.36E-06 | 1.57E-04 | SEH1L; NUP188; NUP35; NUP50; NUP210 |
| 455__KEGG__ko03450; 83047__KEGG__hsa03450 | Non-homologous end-joining | 4 | 34.57 | 3.75E-06 | 1.73E-04 | POLM; RAD50; FEN1; MRE11 |
| 1269058__REACTOME__R-HSA-162587 | HIV Life Cycle | 8 | 8.38 | 4.43E-06 | 1.98E-04 | SEH1L; NUP188; FEN1; NUP35; NUP50; GTF2H4; NUP210; CDK7 |
| 672450__WikiPathways__WP1971 | Integrated Cancer Pathway | 5 | 19.20 | 5.03E-06 | 2.22E-04 | PTEN; RAD50; CHEK1; MRE11; CHEK2 |
| 1270425__REACTOME__R-HSA-3371453 | Regulation of HSF1-mediated heat shock response | 6 | 13.24 | 5.22E-06 | 2.27E-04 | SIRT1; SEH1L; NUP188; NUP35; NUP50; NUP210 |

| | | | | | | |
|---|---|---|---|---|---|---|
| 1269117__REACTOME__R-HSA-168325 | Viral Messenger RNA Synthesis | 5 | 18.52 | 6.08E-06 | 2.54E-04 | SEH1L; NUP188; NUP35; NUP50; NUP210 |
| 1269920__REACTOME__R-HSA-189200 | Hexose transport | 5 | 18.52 | 6.08E-06 | 2.54E-04 | SEH1L; NUP188; NUP35; NUP50; NUP210 |
| 1383076__REACTOME__R-HSA-5633007 | Regulation of TP53 Activity | 8 | 7.83 | 7.40E-06 | 3.04E-04 | RAD17; MRE11; AURKB; CHEK1; RAD50; RAD9A; NBN; CHEK2 |
| 1269731__REACTOME__R-HSA-194441; 1269732__REACTOME__R-HSA-191859 | Metabolism of non-coding RNA; snRNP Assembly | 5 | 16.73 | 1.03E-05 | 4.12E-04 | SEH1L; NUP188; NUP35; NUP50; NUP210 |
| 1309089__REACTOME__R-HSA-6784531 | tRNA processing in the nucleus | 5 | 16.20 | 1.21E-05 | 4.78E-04 | SEH1L; NUP188; NUP35; NUP50; NUP210 |
| 711360__WikiPathways__WP2377 | Integrated Pancreatic Cancer Pathway | 8 | 7.09 | 1.54E-05 | 6.00E-04 | PTEN; SIRT1; MRE11; GADD45A; CHEK1; RAD50; BRCA2; CHEK2 |
| 1269815__REACTOME__R-HSA-2980766 | Nuclear Envelope Breakdown | 5 | 15.25 | 1.65E-05 | 6.35E-04 | SEH1L; NUP188; NUP35; NUP50; NUP210 |
| 1309097__REACTOME__R-HSA-5693548 | Sensing of DNA Double Strand Breaks | 3 | 51.85 | 1.69E-05 | 6.37E-04 | RAD50; NBN; MRE11 |
| 1458264__WikiPathways__WP2942 | DDX1 as a regulatory component of the Drosha microprocessor | 3 | 51.85 | 1.69E-05 | 6.37E-04 | RAD50; NBN; MRE11 |
| 1269723__REACTOME__R-HSA-5578749 | Transcriptional regulation by small RNAs | 5 | 14.81 | 1.91E-05 | 7.00E-04 | SEH1L; NUP188; NUP35; NUP50; NUP210 |
| 1269069__REACTOME__R-HSA-162599 | Late Phase of HIV Life Cycle | 7 | 8.16 | 2.18E-05 | 7.90E-04 | SEH1L; NUP188; NUP35; NUP50; GTF2H4; NUP210; CDK7 |
| 1270421__REACTOME__R-HSA-3371556 | Cellular response to heat stress | 6 | 10.20 | 2.43E-05 | 8.71E-04 | SIRT1; SEH1L; NUP188; NUP35; NUP50; NUP210 |
| 1269810__REACTOME__R-HSA-68886 | M Phase | 9 | 5.43 | 3.78E-05 | 1.34E-03 | NUP35; NUP50; NCAPD3; NUP210; AURKB; NUP188; MCPH1; SEH1L; PDS5A |
| 1270414__REACTOME__R-HSA-2262752 | Cellular responses to stress | 11 | 4.35 | 3.86E-05 | 1.35E-03 | SIRT1; NUP35; NUP50; MRE11; NUP210; CBX8; RAD50; SEH1L; NUP188; NBN; FZR1 |
| 137959__Pathway Interaction Database__bard1pathway | BARD1 signaling events | 4 | 19.75 | 4.24E-05 | 1.47E-03 | RAD50; NBN; MRE11; FANCG |

| | | | | | | |
|---|---|---|---|---|---|---|
| 413428__KEGG__hsa_M00295; 890555__KEGG__M00295 | BRCA1-associated genome surveillance complex (BASC) | 3 | 38.89 | 4.67E-05 | 1.60E-03 | RAD50; NBN; MRE11 |
| 1269694__REACTOME__R-HSA-159236 | Transport of Mature mRNA derived from an Intron-Containing Transcript | 5 | 11.52 | 6.65E-05 | 2.23E-03 | SEH1L; NUP188; NUP35; NUP50; NUP210 |
| 1269719__REACTOME__R-HSA-211000 | Gene Silencing by RNA | 5 | 11.03 | 8.22E-05 | 2.73E-03 | SEH1L; NUP188; NUP35; NUP50; NUP210 |
| 1269316__REACTOME__R-HSA-1169410; 1269317__REACTOME__R-HSA-1169408 | Antiviral mechanism by IFN-stimulated genes; ISG15 antiviral mechanism | 5 | 10.80 | 9.11E-05 | 2.98E-03 | SEH1L; NUP188; NUP35; NUP50; NUP210 |
| 413422__KEGG__hsa_M00286; 890548__KEGG__M00286 | GINS complex | 2 | 103.70 | 9.19E-05 | 2.98E-03 | GINS4; GINS2 |
| 1269649__REACTOME__R-HSA-74160 | Gene Expression | 23 | 2.25 | 1.13E-04 | 3.61E-03 | NUP35; MRE11; NUP210; AURKB; GADD45A; RAD50; RAD9A; AQR; PTEN; SIRT1; NCOA6; RAD17; NUP50; CDK7; CHEK1; SEH1L; NUP188; MTA1; SUV39H1; NBN; GTF2H4; TNKS1BP1; CHEK2 |
| 1269693__REACTOME__R-HSA-72202 | Transport of Mature Transcript to Cytoplasm | 5 | 10.17 | 1.22E-04 | 3.85E-03 | SEH1L; NUP188; NUP35; NUP50; NUP210 |
| 1269650__REACTOME__R-HSA-212436 | Generic Transcription Pathway | 14 | 3.11 | 1.30E-04 | 4.05E-03 | PTEN; NCOA6; RAD17; MRE11; CDK7; AURKB; GADD45A; RAD50; CHEK1; RAD9A; NBN; GTF2H4; TNKS1BP1; CHEK2 |
| 1269057__REACTOME__R-HSA-162906 | HIV Infection | 8 | 5.22 | 1.38E-04 | 4.25E-03 | SEH1L; NUP188; FEN1; NUP35; NUP50; GTF2H4; NUP210; CDK7 |
| 1269777__REACTOME__R-HSA-69242 | S Phase | 6 | 7.32 | 1.59E-04 | 4.86E-03 | GINS4; FEN1; FZR1; PDS5A; CDK7; GINS2 |
| 1309088__REACTOME__R-HSA-72306 | tRNA processing | 5 | 8.79 | 2.45E-04 | 7.42E-03 | SEH1L; NUP188; NUP35; NUP50; NUP210 |
| 1269734__REACTOME__R-HSA-212165 | Epigenetic regulation of gene expression | 5 | 8.64 | 2.65E-04 | 7.95E-03 | SIRT1; MTA1; SUV39H1; GTF2H4; CDK7 |
| 1268701__REACTOME__R-HSA-597592 | Post-translational protein modification | 15 | 2.68 | 3.75E-04 | 1.11E-02 | PTEN; RNF168; WDR48; NUP35; TOP2B; NUP50; NUP210; AURKB; NSMCE1; CBX8; SEH1L; MTA1; NUP188; NSMCE3; UCHL5 |
| 1269737__REACTOME__R-HSA-5250941 | Negative epigenetic regulation of rRNA expression | 4 | 10.92 | 4.62E-04 | 1.36E-02 | SIRT1; SUV39H1; GTF2H4; CDK7 |

| | | | | | | |
|---|---|---|---|---|---|---|
| 413476__KEGG__hsa_M00413; 890628__KEGG__M00413 | FA core complex | 2 | 51.85 | 5.44E-04 | 1.58E-02 | FAAP24; FANCG |
| 1269784__REACTOME__R-HSA-69190 | DNA strand elongation | 3 | 16.37 | 7.49E-04 | 2.16E-02 | GINS4; FEN1; GINS2 |
| 1309121__REACTOME__R-HSA-6783310 | Fanconi Anemia Pathway | 3 | 15.56 | 8.75E-04 | 2.50E-02 | FAAP24; FANCG; MUS81 |
| 465__KEGG__ko04115; 83055__KEGG__hsa04115 | p53 signaling pathway | 4 | 9.22 | 8.85E-04 | 2.51E-02 | PTEN; CHEK1; CHEK2; GADD45A |
| 1084767__KEGG__hsa_M00691; 1108230__KEGG__M00691 | DNA damage-induced cell cycle checkpoints | 2 | 41.48 | 9.01E-04 | 2.53E-02 | CHEK1; CHEK2 |
| 463__KEGG__ko04110; 83054__KEGG__hsa04110 | Cell cycle | 5 | 6.48 | 1.01E-03 | 2.80E-02 | CHEK1; FZR1; CDK7; CHEK2; GADD45A |
| 137987__Pathway Interaction Database__telomerasepathway | Regulation of Telomerase | 4 | 8.83 | 1.05E-03 | 2.88E-02 | RAD50; RAD9A; NBN; MRE11 |
| 137935__Pathway Interaction Database__foxm1pathway | FOXM1 transcription factor network | 3 | 13.53 | 1.33E-03 | 3.64E-02 | BRCA2; CHEK2; AURKB |
| 1270429__REACTOME__R-HSA-2559586 | DNA Damage/Telomere Stress Induced Senescence | 3 | 12.96 | 1.51E-03 | 4.05E-02 | RAD50; NBN; MRE11 |
| 1269757__REACTOME__R-HSA-176187 | Activation of ATR in response to replication stress | 3 | 12.96 | 1.51E-03 | 4.05E-02 | CHEK1; RAD9A; RAD17 |
| 137925__Pathway Interaction Database__aurora_a_pathway | Aurora A signaling | 3 | 12.44 | 1.71E-03 | 4.54E-02 | FZR1; AURKB; GADD45A |
| 413425__KEGG__hsa_M00290; 890551__KEGG__M00290 | Holo-TFIIH complex | 2 | 29.63 | 1.87E-03 | 4.85E-02 | GTF2H4; CDK7 |

| | | | | | | |
|---|---|---|---|---|---|---|
| 198895__WikiPathways__ WP438 | Non-homologous end joining | 2 | 29.63 | 1.87E-03 | 4.85E-02 | RAD50; MRE11 |
| 1458190__WikiPathways__ WP3875 | ATR Signaling | 2 | 29.63 | 1.87E-03 | 4.85E-02 | CHEK1; RAD9A |
